# Collective asymmetric synthesis of the *Strychnos* alkaloids via thiophene *S,S*-dioxide cycloadditions

**DOI:** 10.1038/s41557-025-02041-1

**Published:** 2026-01-23

**Authors:** Kun Ho ‘Kenny’ Park, Jisook Park, Nils Frank, Hanwen Zhang, Peilin Tian, Yasmine Biddick, Fernanda Duarte, Edward A. Anderson

**Affiliations:** Chemistry Research Laboratory, Department of Chemistry, University of Oxford, Oxford, UK

**Keywords:** Natural product synthesis, Synthetic chemistry methodology, Computational chemistry, Asymmetric synthesis

## Abstract

The *Strychnos* alkaloids have long been regarded as landmark targets for chemical synthesis due to their captivating architectures and notorious biological properties. However, the design of approaches that access multiple family members in an asymmetric, concise and atom-economical fashion remains an important challenge. Here we show that thiophene *S,S*-dioxides (TDOs) offer a modular, rapid entry to *Strychnos* natural products via inverse electron demand Diels–Alder cascades. We demonstrate that exceptional levels of stereocontrol can be achieved in asymmetric TDO cycloadditions, affording tricyclic indolines of utility in medicinal chemistry research and enabling the stereoselective synthesis of eight *Strychnos* alkaloids by the shortest routes described so far, including a synthesis of the iconic family member brucine. Using a machine-learning approach, computational studies provide insight into the source of stereoinduction and reveal an intriguing and unexpected spontaneous cheletropic extrusion of SO_2_.

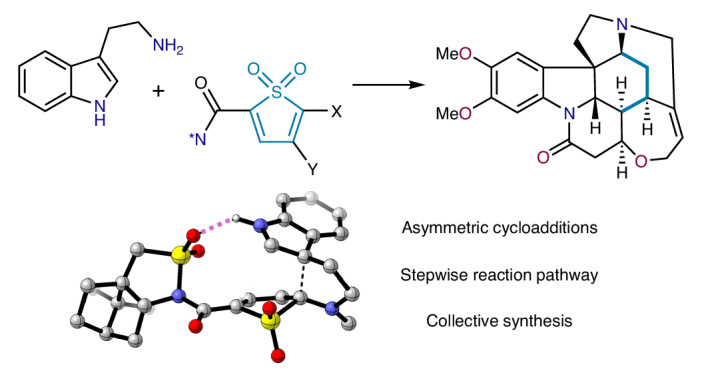

## Abstract (web summary)

Thiophene *S*,*S*-dioxides are excellent substrates for cycloaddition reactions, but underused in target-oriented synthesis. Here the authors show how these heterocycles enable the asymmetric synthesis of tricyclic indolines, as well as the collective synthesis of the iconic *Strychnos* alkaloids. Computational studies rationalize the source of asymmetry and reveal a spontaneous SO_2_ extrusion pathway.

## Main

**Fig. 1:**
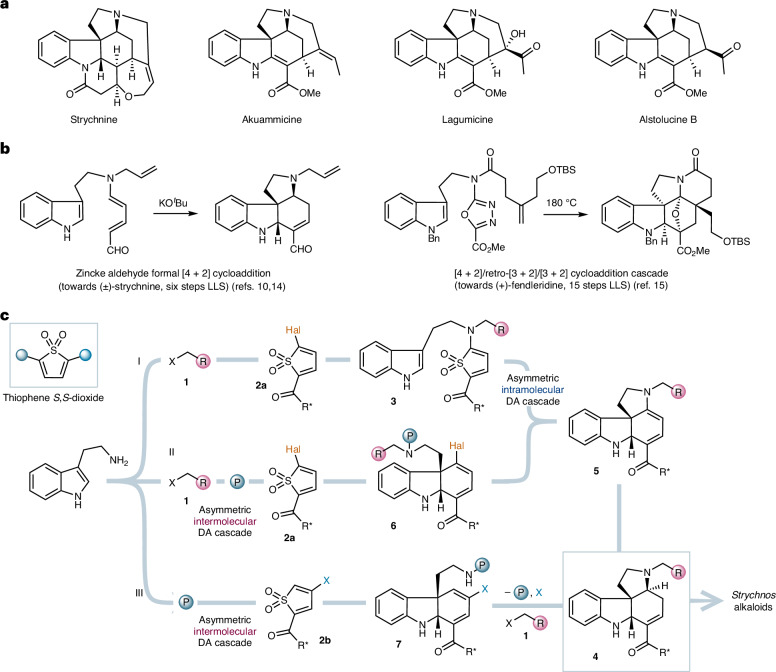
Representative *Strychnos* alkaloids, selected previous cycloaddition approaches to indole alkaloids and the synthesis blueprint in this work. **a**, Examples of *Strychnos* alkaloids include strychnine, akuammicine, lagumicine and alstolucine B. **b**, Previous inverse electron demand cycloaddition approaches to indole alkaloids include the use of Zincke aldehydes^10,14^ and oxadiazoles^15^. **c**, This work: the three modular strategies that were explored to access tetracycle **4**, a key precursor for the collective synthesis of eight *Strychnos* alkaloids, via asymmetric cycloaddition cascades of TDOs. X, substituent; TBS, SiMe_2_^*t*^ Bu; R, substituent; Hal, halide; R*, chiral substituent; P, protecting group; DA, Diels–Alder.

The *Strychnos* indole alkaloid natural products have long excited the scientific community, not least because of their potent biological properties and architectural complexity (Fig. 1a). Robinson infamously said of the flagship member of the family, strychnine, that “for its molecular size, it is the most complex substance known”^1^, and the landmark synthesis of this natural product by Woodward et al.^2,3^ inspired the design of many synthetic approaches to these alkaloids^4–6^. In addition to the structural challenges, such endeavours have also been stimulated by their potential biological applications, such as the identification of alstolucines B and F as candidates for the re-sensitization of taxanes in multidrug-resistant cancers^7^. However, synthetic approaches that are asymmetric, sufficiently concise to produce useful quantities of natural product and also sufficiently flexible to enable the ‘collective’ synthesis of multiple family members are rare. Additional demands of synthesis ideality, such as the avoidance of protecting groups and the minimization of waste^8,9^, impose further challenges that continue to limit the wider exploitation of the family. In this context, the six-step racemic synthesis of strychnine by the Vanderwal group^10^ and the collective asymmetric synthesis of six indole alkaloids by MacMillan et al. (including a twelve-step asymmetric approach to strychnine)^11^ represent pioneering contributions, with recent elegant approaches having also been described by the Zhang and Snaddon groups^12,13^.

We questioned whether an entry to the *Strychnos* family might be designed to meet the stringent demands of contemporary synthetic chemistry, specifically a modular, asymmetric strategy that would enable the construction of the *Strychnos* core in a handful of steps, and thereby access multiple natural products in a scalable, straightforward manner. We recognized that the polycyclic indoline core of the alkaloids is an attractive motif for ring formation via cycloaddition, in which an indole precursor would serve as an electron-rich 2*π* component. This concept was exploited by Vanderwal and co-workers using tethered Zincke aldehydes (Fig. 1b)^10,14^, and also by the Boger and Padwa groups via [3 + 2] cycloadditions with tethered carbonyl ylides^15,16^. However, these elegant strategies suffer from drawbacks, including the need for harsh reaction conditions when employing aromatic cycloaddition partners such as oxadiazoles (for example, heating at 180 °C for 48 h (ref. ^15^)), the use of hazardous diazo compounds for carbonyl ylide synthesis^16^ or the formation of racemic products^10^ that necessitate late-stage chromatographic resolution^15^.

We hypothesized that thiophene *S*,*S*-dioxides (TDOs, Fig. 1c)^17^ could address these limitations. These heterocycles are in many ways ‘ideal’ diene substrates for inverse electron demand Diels–Alder (IEDDA) reactions as they are non-aromatic and should therefore exhibit enhanced reactivity compared with traditional substrates such as pyrones or tetrazines, while still having the benefits of electron deficiency, an intrinsically s-*cis* constrained diene and a powerful entropic driving force that renders the cycloaddition irreversible (that is, the loss of SO_2_ through cheletropic extrusion). However, the deployment of TDOs in target-oriented synthesis is surprisingly rare, with applications so far being restricted to aromatic carbocycles such as the relatively simple illudalane triterpenes, the antiplatelet agent beraprost and the indoline core of the dictyodendrins^18–20^. We questioned whether these underexploited dienes could also be used to assemble the saturated carbocycles found in the *Strychnos* core, and whether we might simultaneously develop asymmetric TDO cycloadditions, which would render the collective synthesis enantioselective.

Three complementary TDO-based approaches were envisaged to access the *Strychnos* core, which vary strategically in the order of synthetic events (Fig. 1c). First (Path I), tryptamine could undergo alkylation with side chain **1** suitable for downstream completion of the targeted natural products, followed by addition–elimination (substitution) reaction with a halogenated, chiral TDO **2a** to give intermediate **3**. The TDO in **3** would react with the tethered indole via a stereoselective intramolecular IEDDA–SO_2_ cheletropic extrusion pathway to form tetracycle **4** after partial reduction of the intermediate dienamine cycloadduct **5**. Further functionalization of **4** would afford the *Strychnos* natural products. As an alternative, we considered intermolecular approaches such as Path II, in which temporary protection of the tryptamine side-chain nitrogen atom precedes intermolecular cycloaddition with TDO **2a** to give diene **6**; cleavage of the nitrogen atom protecting group should then lead to intermediate **5** by intramolecular substitution of the TDO-derived halogen atom, thereby converging on Path I. Finally (Path III), we considered the direct cycloaddition of N-protected tryptamine with TDO **2b** to form adduct **7**. TDO **2b** would necessarily feature a disposable substituent (X), which is essential to prevent heterodimerization of the thiophene ring system in the course of its oxidation^17^. Subsequent to the cycloaddition cascade, deprotection and cyclization of the tryptamine side chain in **7**, cleavage of the superfluous substituent X and *N*-alkylation with side chain **1** would also afford advanced intermediate **4**. Here we describe the realization of all three strategies, which culminated in a collective asymmetric synthesis of eight natural products of the *Strychnos* family. In doing so, we also established asymmetric intermolecular cycloadditions of TDOs, accessing tricyclic indoline scaffolds that are of particular interest for medicinal chemistry applications. Computational studies revealed the source of the stereoselectivity observed in both the intra- and intermolecular IEDDA reactions, and suggested that the intramolecular TDO cycloadditions may benefit from an entropic driving force, where cheletropic extrusion of SO_2_ occurs in a spontaneous manner along the cycloaddition pathway without the formation of a discrete intermediate [4 + 2] cycloadduct.

## Results

### Asymmetric thiophene *S*,*S-*dioxide cycloaddition reactions

With a view to developing an asymmetric route to the *Strychnos* family, we first addressed the development of hitherto unrealized asymmetric TDO cycloadditions. Investigations began with intermolecular [4 + 2] cycloadditions between indoles and enantioenriched thiophene *S*,*S*-dioxides (prepared by peroxyacid oxidation of the corresponding thiophene^21^) equipped with inexpensive, readily available chiral substituents. We found that the reaction of indole (**8a**, Table 1, R^1^, R^2^, R^3^ = H) with a TDO substituted with an acyl camphorsultam group^22^ (**9a**, R^4^ = 5-Cl) in CH_2_Cl_2_ at 0 °C afforded tricycle **10a** as a single diastereomer in high yield. The stereochemistry of **10a** was established by single-crystal X-ray diffraction analysis, which revealed the indoline ring junction to possess the absolute stereochemistry required for the natural stereoisomers of the *Strychnos* alkaloids. This method was tested on a range of substituted indoles and showed broad functional group tolerance of both electron-rich (for example, methoxy) and electron-poor substituents (for example, ester, nitro and nitrile) at various positions around the indole ring, as well as useful functionalities such as boronic esters and halides and substitution of the nitrogen atom. All tricyclic products were isolated as single diastereomers in good-to-excellent yields (**10b**–**10m**, 68–93%). Variation of the thiophene *S*,*S*-dioxide was also well tolerated, including a bicyclic TDO (**10n**–**10p**, 54–68%). With a view towards the proposed intermolecular cycloaddition approach to the *Strychnos* alkaloids, we also evaluated the use of 3-substituted indoles in this chemistry. These reactions were also successful, albeit requiring higher temperatures to effect the cycloaddition due to the steric demand imposed by the additional indole substituent, affording cycloadducts **10q** and **10r** in 90% and 91% yields, respectively, again as single diastereomers.

**Table 1:**
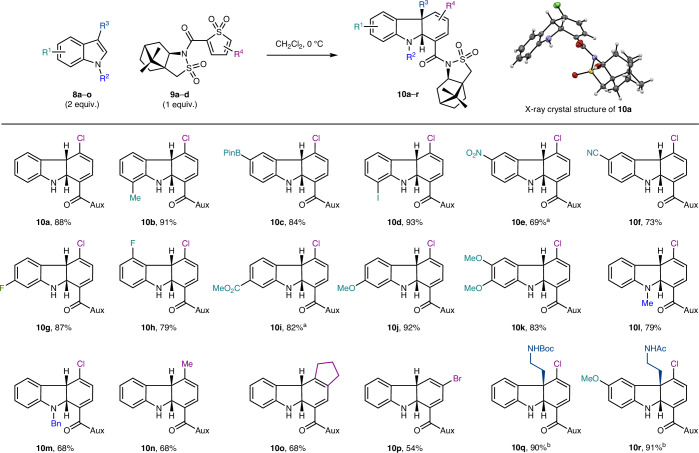
Development of stereoselective TDO cycloaddition reactions

The inset shows the X-ray crystallographic structure of **10a**. Aux, camphorsultam auxiliary group; PinB, pinacolboryl. ^a^Compounds **10e** and **10i** were synthesized at room temperature. ^b^Compounds **10q** and **10r** were synthesized at 80 °C in CHCl_3_.

### Synthesis of the *Strychnos* alkaloids

**Fig. 2:**
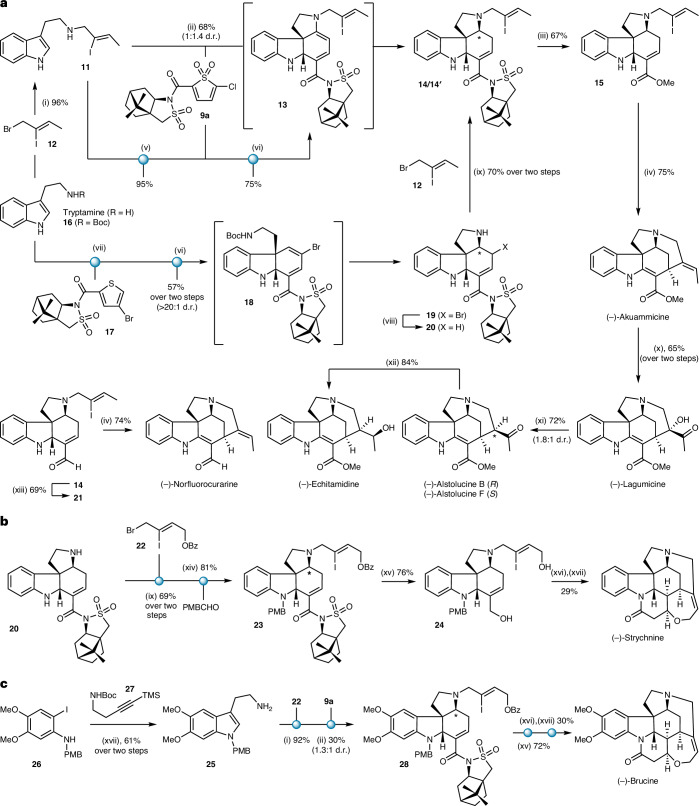
Collective asymmetric syntheses of the *Strychnos* alkaloids. **a**, Syntheses of akuammicine, norfluorocurarine, lagumicine, alstolucines B and F, and echitamidine. **b**, Synthesis of strychnine. **c**, Synthesis of brucine. Reagents and conditions: (i) MeCN, room temperature (r.t.); (ii) MeCN, r.t. to 80 °C (70 °C for **28**); then AcOH, NaBH_3_CN, 65 °C (55 °C for **28**); (iii) NaOMe, dimethyl carbonate, CH_2_Cl_2_, 0 °C; (iv) Pd(OAc)_2_, PPh_3_, Et_3_N, 70 °C; (v) Boc_2_O, Et_3_N, CH_2_Cl_2_; (vi) CHCl_3_, 80 °C; TFA, r.t.; (vii) trifluoroacetic anhydride, H_2_O_2_, 0 °C to r.t.; (viii) H_2_ (1 atm), Pd/C, MeOH–EtOAc, r.t.; (ix) Na_2_CO_3_, MeCN, r.t.; (x) K_2_OsO_4_·2H_2_O, NMO·H_2_O, ^*t*^BuOH–H_2_O, r.t.; then Dess–Martin periodinane, ^*t*^BuOH, CH_2_Cl_2_, r.t.; (xi) SmI_2_, THF–MeOH, 0 °C to r.t.; (xii) NaBH_4_, MeOH, 0 °C to r.t.; (xiii) DIBALH, CH_2_Cl_2_, −78 °C; (xiv) *p*-anisaldehyde, NaBH(OAc)_3_, AcOH, 1,2-dichloroethane, 0 °C to r.t.; (xv) DIBALH, CH_2_Cl_2_, −78 to 0 °C; (xvi) Pd(OAc)_2_, Bu_4_NCl, NaHCO_3_, EtOAc, r.t.; (xvii) PhSH, TFA, 45 °C (50 °C for brucine); then NaOAc, Ac_2_O, AcOH, malonic acid, 120 °C; (xviii) Pd(OAc)_2_, LiCl, K_2_CO_3_, *N*,*N*-dimethylformamide, 105 °C; then (±)-camphorsulfonic acid, ^*i*^PrOH, 50 °C.

Having established highly selective, asymmetric intermolecular TDO cycloaddition reactions, we turned to apply them to the synthesis of the *Strychnos* alkaloids, exploring both the intra- and intermolecular routes. We first tested an intramolecular (tethered) cascade towards akuammicine (Fig. 2a). A key issue was whether the excellent stereocontrol imparted by the camphorsultam group in the intermolecular cycloadditions would be maintained in an intramolecular setting. In the event, the reaction of TDO **9a** with amine **11** (prepared by the N alkylation of tryptamine with known side chain **12**^23^) at room temperature, followed by warming to 80 °C, afforded a single diastereomer of the IEDDA cycloadduct **13**. This intermediate dienamine was reduced in situ using acetic acid and sodium cyanoborohydride^24–26^ to afford diastereomers **14** and **14′** in a 1:1.4 ratio and 68% overall yield from **9a** (see Supplementary Table 1 for details). The adverse selectivity of this step likely derives from the preferred delivery of hydride to the less-hindered concave face of the intermediate iminium ion^24–26^. To complete the synthesis of akuammicine, the camphorsultam amide was subjected to methanolysis^27^ to give the corresponding ester **15** (67%), with the camphorsultam moiety recovered in high yield. Heck cyclization^28,29^ of **15** proceeded to afford (–)-akuammicine in 75% yield in four steps from tryptamine (six steps in the longest linear sequence (LLS) that includes assembly of the side chain **12**). This represents the most concise and atom-economical assembly of akuammicine described so far. An intermolecular cycloaddition approach to **13** (corresponding to Path II in Fig. 1c) could also be achieved by *tert*-butoxycarbonyl (Boc) protection of the side-chain amine in **11** (95%). Reaction of the resulting carbamate-protected indole with TDO **9a** gave an intermediate cycloadduct that upon treatment with trifluoroacetic acid (TFA), followed by basic work-up to trigger cyclization of the free amine onto the dienoyl chloride, also afforded dienamine **13** in 75% yield (see Supplementary Section 2.2 for details).

The poor selectivity observed in the reduction of the dienamine cycloadduct **13** prompted us to test the alternative intermolecular asymmetric cycloaddition strategy (Path III in Fig. 1c). We identified 4-bromo-TDO **9d** (as used in the synthesis of **10p**, Table 1) as a potential candidate for this route as its bromine substituent would protect the corresponding thiophene from unwanted dimerization during oxidation^17^ and would also likely be removed under mild conditions later in the synthesis. We found that intermolecular cycloaddition of *N-*Boc-tryptamine **16** with **9d** (used directly from the oxidation of thiophene **17**) afforded a single diastereomer of dihydrocarbazole **18**. In situ addition of TFA effected Boc deprotection, and subsequent addition of the tryptamine side-chain amine onto the dienoyl motif upon basic work-up gave a single diastereomer of the pyrrolidine product **19** in 57% yield (from **17**). We found that the allylic bromide resident in **19** could then be selectively cleaved by hydrogenolysis without reduction of the adjacent alkene; attachment of the vinyl iodide side chain **12** to the debrominated product **20** smoothly afforded **14**, thus converging on the intramolecular cycloaddition route, but now with complete control of the stereochemistry at the three contiguous stereocentres within the tetracycle. Including the previously implemented endgame, this alternative synthesis of (–)-akuammicine proceeded in seven steps in the LLS (20% overall yield). Four additional natural products (lagumicine, alstolucines B and F, and echitamidine) were synthesized from akuammicine using established chemistry^30^, while (–)-norfluorocurarine was prepared from **14** by initial reduction to aldehyde **21** with diisobutylaluminium hydride (DIBALH) (69%), followed by Heck cyclization (74%).

Pyrrolidine **20**, prepared via the intermolecular cycloaddition route, also provided access to strychnine (Fig. 2b). The alkylation of **20** with side chain **22** (69% yield from **19**), followed by *para-*methoxybenzyl (PMB) protection^11^ of the indoline nitrogen atom gave tetracycle **23** (81%). From here, exhaustive reduction with DIBALH afforded diol **24** (76%), Heck cyclization–lactol formation of which proceeded smoothly to afford the PMB-protected Wieland–Gumlich aldehyde (57% yield). This was converted into (–)-strychnine via deprotection of the PMB group, followed by treatment with malonic acid, acetic anhydride and sodium acetate (50% over two steps)^11^. Overall, the synthesis of (–)-strychnine was completed in ten steps via this intermolecular cycloaddition route (LLS, including the synthesis of side chain **22**), which represents the most concise asymmetric approach reported so far (see Supplementary Information for details of the intramolecular TDO cycloaddition approach, which proceeded in seven steps LLS from tryptamine, but with inferior stereocontrol).

Despite the rich history of the *Strychnos* alkaloids, there is one family member that has thus far eluded chemical synthesis: brucine. This dimethylcatechol analogue of strychnine has been known for over 200 years, and has been employed by chemists as a chiral resolving agent since Fischer’s seminal report in 1899^31^; its catechol derivative has been used as a chiral ligand in asymmetric catalysis^32^. The challenge for any synthesis of brucine relates to the highly electron-rich nature of the indoline ring, which confers sensitivity towards oxidation and electrophilic degradation. To implement the TDO cascade approach to brucine, we first required *N-*PMB-5,6-dimethoxytryptamine **25** (Fig. 2c). Due to the aforementioned instability issues, precedents for the synthesis of highly oxidized tryptamines are not well documented, but after extensive investigation we found that **25** could be accessed by Larock indole synthesis^33,34^ from iodoaniline **26** and alkyne **27**, which afforded **25** in good yield (61% over two steps). The successful progression of **25** towards brucine proved possible only via the intramolecular TDO cascade: alkylation of **25** with side chain **22** proceeded uneventfully (92%), but due to the reduced stability of the 5,6-dimethoxyindole, the cycloaddition with **9a** benefited from a lower reaction temperature (70 °C) and a longer reaction time, affording **28** after reduction of the intermediate dienamine (28% yield from **25**, 1.3:1 diastereomeric ratio (d.r.)). A similar endgame sequence to that employed in the synthesis of strychnine then accomplished the total synthesis of (–)-brucine in nine steps LLS from commercial 2-iodo-4,5-dimethoxyaniline. Attempts to implement the alternative intermolecular TDO cascade proved unsuccessful, with complex mixtures observed under the cycloaddition conditions, presumably reflecting the challenge of deploying a highly electron-rich indole.

### Theoretical investigations

To better understand the basis of the remarkable levels of asymmetric induction imparted by the TDO camphorsultam side chain in the inter- and intramolecular cycloadditions, we performed quantum mechanics (QM) calculations and molecular dynamics (MD) simulations driven by MACE machine learning interatomic potentials (MLIPs) to model the cycloaddition step^35^. MLIPs ‘learn’ the high-dimensional potential energy surfaces (PES) from QM calculations, effectively mapping atomic positions to energies and, often, forces with an accuracy comparable to QM and efficiency comparable to simple empirical force fields^36,37^.

**Fig. 3:**
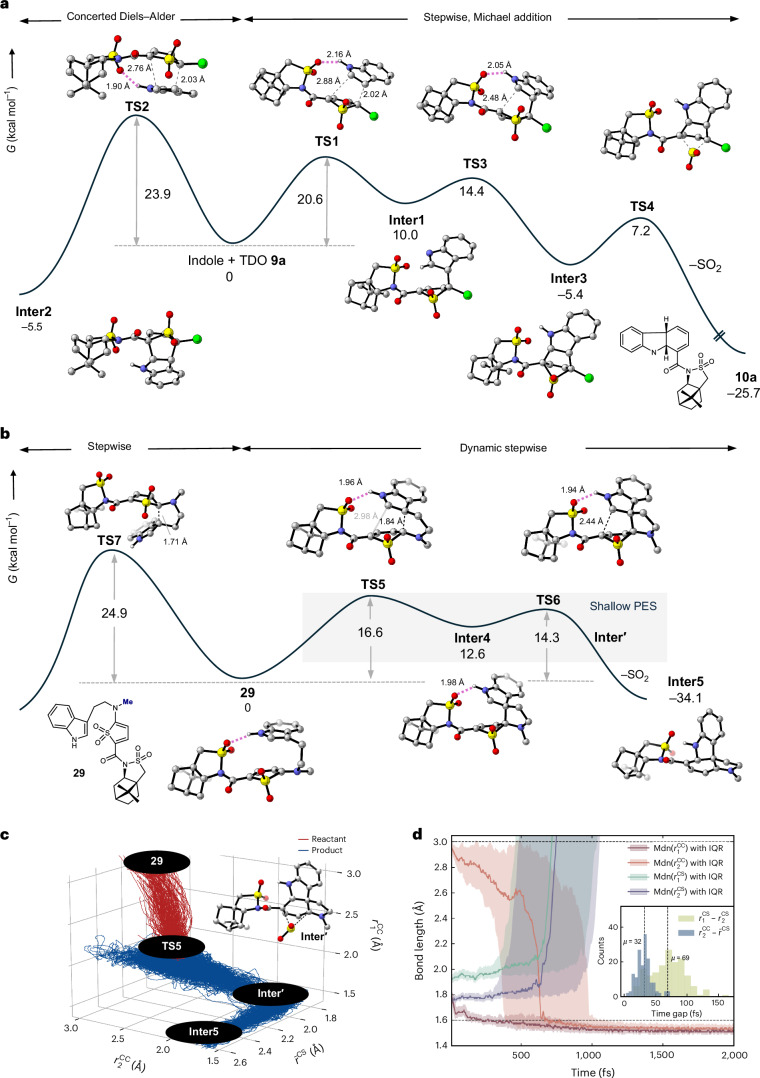
Computational mechanistic exploration. **a**,**b**, Intermolecular IEDDA cycloaddition of TDO **9a** with indole (**a**) and intramolecular IEDDA cycloaddition of *N-*methyltryptamine-derived TDO **29**. The free-energy profiles were computed at the CPCM(MeCN)-DLPNO-CCSD(T)/def2-TZVP//CPCM(MeCN)-B2PLYP-D3BJ/def2-SVP level of theory (353 K/1 M). **c**, MLIP-MD simulations revealed a concerted asynchronous [4 + 2] process involving a short-lived intermediate (**Inter′**) and a dynamic stepwise extrusion of SO_2_. ${\bar{{{r}}}}_{\mathrm{CS}}$, mean of the two C–S bonds. **d**, Time evolution of the C–C and C–S distances after **Inter4**. Median (Mdn) values of the C–C and C–S bond lengths (${r}_{1}^{\mathrm{CC}}$, ${r}_{2}^{\mathrm{CC}}$, ${r}_{1}^{\mathrm{CS}}$ and ${r}_{2}^{\mathrm{CS}}$) are shown as solid lines, with the shaded regions representing the interquartile range (IQR; that is, the first to the third quartile) across trajectories. Inset: distributions of time gaps between the formation of ${r}_{1}^{\mathrm{CS}}$ and ${r}_{2}^{\mathrm{CS}}$, and between ${r}_{2}^{\mathrm{CC}}$ and ${\bar{r}}_{\mathrm{CS}}$. The dashed lines indicate mean values of 32 and 69 fs, respectively. MLIP-MD was trained using the MACE architecture^35^, with the CPCM(MeCN)-B2PLYP-D3BJ/def2-SVP level of theory used as the ground-truth method (Supplementary Section 4.1.2).

We first explored the facial selectivity of the intermolecular cycloaddition reaction of TDO **9a** with indole at the CPCM(MeCN)-DLPNO-CCSD(T)/def2-TZVP//CPCM(MeCN)-B2PLYP-D3BJ/def2-SVP level of theory (353 K/1 M, Fig. 3a). Interestingly, we found that the preferred pathway involves a stepwise cycloaddition via **TS1**, proceeding through a series of shallow energy minima before yielding the experimentally observed product diastereomer **10a** (Fig. 3a, right). In contrast, the alternative diastereomer was found to form via a concerted cycloaddition pathway, in which the transition state (**TS2**) is 3.3 kcal mol^−1^ higher in energy (Fig. 3a, left). The favoured pathway via **TS1** initially leads to **Inter1** (Gibbs free energy of reaction Δ*G*° = +10 kcal mol^−1^), which undergoes a low-barrier cyclization (**TS3**, activation energy Δ*G*^‡^ = 14.4 kcal mol^−1^) to form the [4 + 2] cycloadduct **Inter3**. Exergonic extrusion of SO_2_ via **TS4** (Δ*G*^‡^ = 12.6 kcal mol^−1^) affords the observed product **10a**.

We were curious to understand the energy difference between **TS1** and **TS2** (2.7 kcal mol^−1^) as both exhibit stabilizing hydrogen bonding and no obvious steric hindrance. Distortion interaction analysis^38^ indicated that the higher energy of **TS2** compared with **TS1** arises from the greater distortion of the TDO **9a** (3.2 kcal mol^−1^ higher in **TS2**, see Supplementary Section 4.6, and Supplementary Fig. 11). Although this increased distortion is partially compensated by a stronger interaction energy in **TS2**, the net result is that the electronic activation energy of **TS2** remains 2.7 kcal mol^−1^ higher than **TS1**.

The intramolecular cycloaddition cascade of the tryptamine–TDO adduct **29**, which features a simplified *N*-methyltryptamine side chain, was found to proceed via a shallower stepwise pathway (Fig. 3b). This held true regardless of which face of the TDO the tethered indole occupies, with an intramolecular hydrogen bond forming between the indole N–H and the sultam SO_2_ group seeming to favour **TS5**, which leads to the experimentally observed diastereomer, over **TS7** (difference in activation energies ΔΔ*G*^‡^ = 8.3 kcal mol^−1^). The PES of the resulting intermediate **Inter4** is shallow, such that the second C–C bond formation occurs via a low-energy transition state (**TS6**, Δ*G*^‡^ = 1.7 kcal mol^−1^). Beyond **TS6**, an unexpected, spontaneous extrusion of SO_2_ leads directly to the dienamine product **Inter5** (Fig. 3b). Attempts to locate the expected SO_2_-bridged [4 + 2] cycloadduct (analogous to **Inter3**) were unsuccessful. Intrigued by this finding, we explored the dynamics of the [4 + 2] mechanism through downhill MD simulations using an MLIP (Fig. 3c and Supplementary Section 4.5 for further details). For this study, 500 trajectories were initialized from **TS5** at 353 K and propagated downhill for 5 ps in the direction of either the reactant or product states. Half of the trajectories reverted to the reactant state, while the remainder progressed towards the product state (Supplementary Fig. 9b). Among the latter, 159 trajectories successfully reached the product, while the remaining 91 were trapped at **Inter4**. These trajectories revealed a highly asynchronous dynamic stepwise process for the cycloaddition reaction, with one C–C bond substantially advanced in the **TS5** region (${r}_{1}^{\mathrm{CC}}$ ≈ 1.84 Å) compared with the other C–C bond (${r}_{2}^{\mathrm{CC}}$ ≈ 3.0 Å). At **TS6**, the second C–C bond formation has progressed (${r}_{2}^{\mathrm{CC}}$ ≈ 2.44 Å) and a transient sulfur-bridged [4 + 2] cycloadduct (**Inter′**) is observed in which the C–S bonds remain intact. However, as the system progresses towards the product state, the C–S bonds elongate and SO_2_ is extruded. The asynchronicity of this process is evidenced in the evolution of the relevant C–C and C–S distances with time (Fig. 3d, *t* = 0 being **TS5**). The average time gap between the formation of the two C–C bonds (defined as bond lengths <1.6 Å) is 748 ± 491 fs, ranging from 109–2,459 fs. At around 700 fs, as the second C–C bond starts to form (${r}_{2}^{\mathrm{CC}}$ shortening to 1.6 Å), the C–S bonds begin to elongate, with ${r}_{1}^{\mathrm{CS}}$ lengthening more than ${r}_{2}^{\mathrm{CS}}$. The time gap between the cleavage of the C–S bonds averages 33 ± 13 fs (ranging from 4–80 fs); this elongation continues until SO_2_ is extruded.

## Discussion

Cascade (or domino) reactions and telescoped synthetic procedures offer useful means to increase the efficiency of target-oriented synthesis. Thiophene *S*,*S*-dioxides offer an excellent opportunity to engineer such events as their ability to undergo cycloadditions that are rendered irreversible by the in situ extrusion of SO_2_ offers a direct means to construct cyclohexadienes. However, TDOs have rarely been exploited in synthetic contexts, and their use in asymmetric cycloadditions has not been reported. Here, TDOs equipped with a cheap, common chiral camphorsultam side chain have been shown to offer efficient, stereoselective and general access to polycyclic indolines, where the cycloaddition step proceeds under remarkably mild conditions (0 °C) for indoles that are unsubstituted at the 2- and 3-positions. The observation that these cycloadditions proceed with exceptional stercocontrol (>20:1 d.r.) suggests that TDOs offer a mild and general method for polycyclic indoline synthesis. Application of this chemistry to various tryptamine derivatives provides an entry to the *Strychnos* alkaloid framework, which enabled the asymmetric synthesis of eight *Strychnos* alkaloids via the most concise routes reported so far. This includes the historic natural product brucine which, despite being known since the early 1800s, had yet to succumb to total synthesis.

Key to the wider application of TDOs is an understanding of the basis of their reactivity and selectivity in asymmetric cycloaddition reactions. Computational studies using QM calculations and MD simulations driven by MLIPs provided insight into the source of stereocontrol imparted by the camphorsultam side chain in both inter- and intramolecular cycloaddition pathways, and also revealed highly asynchronous cycloaddition pathways or even stepwise ‘Michael addition’ mechanisms for the reaction of the TDO with the indole substrate, along with spontaneous extrusion of SO_2_. This results in sound principles for the design of further reactions exploiting TDOs as dienes. Overall, these studies demonstrate that TDOs offer broad potential for application in the synthesis of structurally complex architectures across synthetic and medicinal chemistry.

## Methods

### Representative example of oxidation of thiophene to thiophene *S*,*S*-dioxide 9a

To a stirred solution of trifluoroacetic anhydride (64.3 ml, 463 mmol, 10.2 equiv.) at 0 °C was added H_2_O_2_ (30 wt% in H_2_O, 16.3 ml, 160 mmol, 3.5 equiv.) dropwise. The resulting mixture was warmed to room temperature and stirred for 15 min. To the resulting mixture at 0 °C was added (5-chlorothiophen-2-yl)[(3a*S*,6*R*,7a*R*)-8,8-dimethyl-2,2-dioxidotetrahydro-3*H*-3a,6-methanobenzo[*c*]isothiazol-1(4*H*)-yl]methanone (16.4 g, 45.6 mmol, 1.0 equiv.). The resulting mixture was warmed to room temperature and stirred for 14 h before it was concentrated under reduced pressure. The crude residue was recrystallized from CHCl_3_–MeCN (3:1) to afford compound **9a** (15.7 g, 40.1 mmol, 88%) as a light-yellow solid.

### Representative example of intramolecular cycloaddition–reduction

To a stirred solution of **11** (217 mg, 0.64 mmol, 1.0 equiv.) in MeCN (26 ml) at room temperature was added **9a** (250 mg, 0.64 mmol, 1.0 equiv.). The resulting mixture was stirred for 14 h before additional **11** (261 mg, 0.77 mmol, 1.2 equiv.) was added. The resulting mixture was warmed to 80 °C and stirred for 40 h before it was cooled to 65 °C and AcOH (0.55 ml, 9.61 mmol, 15.0 equiv.) was added. The resulting mixture was stirred for 15 min at 65 °C, and then NaBH_3_CN (401 mg, 6.38 mmol, 10.0 equiv.) in MeOH (6.5 ml) was added. The resulting mixture was stirred at 65 °C for 30 min before it was cooled to room temperature, quenched by the slow addition of a saturated aqueous solution of NaHCO_3_ (20 ml) and then diluted with water (10 ml) and CH_2_Cl_2_ (20 ml). The layers were separated and the aqueous layer was extracted with CH_2_Cl_2_ (3 × 30 ml). The combined organic layers were washed with water (50 ml) and brine (50 ml), dried (Na_2_SO_4_) and concentrated under reduced pressure. Flash column chromatography (silica gel, pentane–Et_2_O 9:1→3:1) afforded compound **14** and its C3a epimer **14′** (276 mg combined mass, 0.44 mmol, 68%, 1:1.4 d.r.) as a brown foam, along with recovered tryptamine **11** (240 mg, 0.71 mmol). These diastereomers could be separated for the purposes of characterization and the subsequent reduction of **14**.

### Representative example of intermolecular cycloaddition–Boc cleavage–cyclization

To a stirred solution of Boc-tryptamine **16** (1.00 g, 3.84 mmol, 2.0 equiv.) in CHCl_3_ (19.0 ml) at room temperature was added **9d**. The resulting mixture was warmed to 80 °C and stirred for 23 h, before it was cooled to 0 °C and TFA (9.0 ml) was added. The resulting mixture was warmed to room temperature and stirred for 2.5 h before it was cooled to 0 °C, quenched by the slow addition of a saturated aqueous solution of Na_2_CO_3_ (200 ml) and then stirred for 20 h. The layers were separated and the aqueous layer was extracted with CH_2_Cl_2_ (3 × 50 ml), and then the combined organic layers were dried (Na_2_SO_4_) and concentrated under reduced pressure. Flash column chromatography (silica gel, pentane–EtOAc 9:1→4:6) afforded compound **19** (585 mg, 1.10 mmol, 57% over two steps) as a yellow foam.

### General procedure for the intermolecular cycloaddition of indoles with thiophene *S*,*S*-dioxides

To a stirred solution of indole (0.2 mmol) in CH_2_Cl_2_ (0.1 M) at 0 °C was added thiophene *S*,*S*-dioxide (0.1 mmol). The resulting mixture was stirred for 38 h before it was concentrated under reduced pressure to afford the crude material, which was purified by flash column chromatography using pentane–EtOAc eluent.

## Online content

Any methods, additional references, Nature Portfolio reporting summaries, source data, extended data, supplementary information, acknowledgements, peer review information; details of author contributions and competing interests; and statements of data and code availability are available at 10.1038/s41557-025-02041-1.

## Supplementary information

Supplementary Information Supplementary Figs. 1–11, Schemes 1–5, Tables 1–18, experimental procedures, computational details and copies of NMR spectra.

Supplementary Data 1 The *xyz* coordinates for all computed structures.

## Data Availability

The data generated in this study, including details concerning experimental procedures and characterization data, are available in the Supplementary Information. Crystallographic data for structure **10a** reported in this Article are available in the Supplementary Information, and have been deposited at the Cambridge Crystallographic Data Centre (CCDC 2408040). Copies of this data can be obtained free of charge at https://www.ccdc.cam.ac.uk/structures/.
