# Supplementary material for: Collective asymmetric synthesis of the Strychnos alkaloids via thiophene S,S-dioxide cycloadditions
Source: Nat Chem. 2026 Jan 23;18(4):782–9. doi: 10.1038/s41557-025-02041-1 (PMC13061625; doi:10.1038/s41557-025-02041-1)

nature chemistry

Supplementary information https://doi.org/10.1038/s41557-025-02041-1

Collective asymmetric synthesis of the *Strychnos* alkaloids via thiophene *S,S*-dioxide cycloadditions

In the format provided by the authors and unedited

Table of contents

1. Materials and Methods S2

2. 2.1 Experimental Procedures and Characterisation Data (*Strychnos* alkaloid synthesis) Including Synthesis of (±)-Akuammicine S4

   2.2 Mechanistic Investigations (*Strychnos* alkaloid synthesis) S47

3. Experimental Procedures and Characterisation Data (Intermolecular TDO / indole cycloadditions) S55

4. Computational Details S68

5. Coordinates S87

6. X-ray crystallographic data S120

7. References S126

8. Copies of NMR Spectra S129

1. Materials and Methods

NMR Spectroscopy. ^{1}H NMR spectra were recorded at 400, 500 MHz on Bruker AVIII HD 400 and AVII 500 spectrometers. ^{13}C NMR spectra were recorded at 101, 126, or 151 MHz on Bruker AVIII HD 400, AVII 500 and NEO 600 spectrometers. Chemical shifts (δ_H and δ_C) are expressed in parts per million (ppm), referenced to the residual solvent peak of $CDCl_3$. Coupling constants (*J*) are reported to the nearest 0.1 Hz. Spectra are assigned based on chemical shift, coupling constants, COSY, HSQC and HMBC data and / or comparison with similar compounds. Splitting patterns are described using the following abbreviations: br (broad), s (singlet), d (doublet), t (triplet), q (quartet), quin. (quintet), sept. (septet).

Infrared Spectroscopy. Infrared spectra were recorder on a Bruker Tensor 27 Fourier transform spectrometer, as a thin film on a diamond ATR module. Absorption maxima (ν_{max}) are quoted in wavenumbers (cm^{-1}).

Polarimetry. Optical rotations were recorded using Perkin Elmer 241 Polarimeter (using the sodium D line, 589 nm) with a path length of 1 dm at 25 °C. $[\alpha]_D^{25}$ are reported in units of 10^{-1} deg cm^2 g^{-1} and the concentrations (c) are reported in g/100 mL

Mass Spectrometry. Low resolution mass spectra were recorded on a Micromass LCT Premier Open Access using electrospray ionisation (ESI). Accurate mass (HRMS) data was determined under conditions of ESI, EI and CI on a Bruker MicroTOF. High resolution values are calculated to 4 decimal places from the molecular formula, and all values are within a tolerance of 5 ppm.

X-ray Diffraction. Low temperature single crystal X-ray diffraction data for **15a** was collected using a Rigaku Oxford SuperNova diffractometer. See page S94 for details.

Reagents, solvents and techniques:

Solvents. Dichloromethane, tetrahydrofuran, *N,N*-dimethylformamide, and toluene were dried by passing through an activated alumina column under argon in a solvent dispenser. All other reagents were used as received. Brine refers to a saturated aqueous solution of NaCl. $NaHCO_3$, NH_4Cl and $Na_2S_2O_3$

solutions refer to saturated aqueous solutions. HCl was also used as an aqueous solution at the specified molarity.

Reactions. All reactions were carried out under argon or nitrogen unless otherwise stated. Oven-dried glassware was used for reactions requiring anhydrous conditions.

Heating. For reactions that require heating, a DrySyn heating block or sand bath was employed. The temperature was monitored via a temperature probe plugged into the stirrer plate.

Chromatography. Thin-layer chromatography was performed on Merck aluminium-backed DC 60 F254 0.2 mm precoated plates, which were visualised with UV fluorescence and staining with potassium (VII) manganate or vanillin. Flash column chromatography was performed on MN Kieselgel 60M (particle size 40-63 μm), under a positive pressure of nitrogen, with the solvent system used in parentheses.

2.1 Experimental Procedures and Characterisation Data (*Strychnos* alkaloid synthesis)

Synthesis of (±)-Akuammicine

Before addressing the asymmetric syntheses of the *Strychnos* alkaloids as described in the main text, the feasibility of the proposed intramolecular cascade was first tested in the setting of a non-asymmetric synthesis of akuammicine (**Scheme S1**). Thiophene *S,S*-dioxide **9e** was synthesised from commercially-available 2-chlorothiophene ester *via* peroxyacid oxidation[1]. The indole dienophile **11** was prepared as described in the main text. Reaction of TDO **9e** with amine **11** at room temperature, followed by warming to 75 °C, efficiently furnished the cycloaddition cascade product **S1**. Reduction of the enamine in **S1** proved challenging; the combination of acetic acid and sodium cyanoborohydride at 65 °C was found to afford the desired diastereomer **S2** and its epimer **S2'** in 63% overall yield (1:2.4 *dr*), in favour of **S2'**[2,3]. As with the auxiliary-equipped product, the adverse selectivity of this step likely derives from the preferred delivery of hydride to the less-hindered concave face of the intermediate iminium ion. We nonetheless found that this three step sequence could be streamlined by performing the enamine reduction in the same reaction flask immediately after the cycloaddition, such that the synthesis of **S2/S2'** could be achieved in 62% combined yield from **11**. An intramolecular Heck reaction[4,5] of the desired isomer **S2** (81%) completed the synthesis of akuammicine in three steps from tryptamine (five steps in the longest linear sequence (LLS) that includes assembly of the sidechain **12**). This represents the most concise and atom-economical assembly of (±)-akuammicine to date.

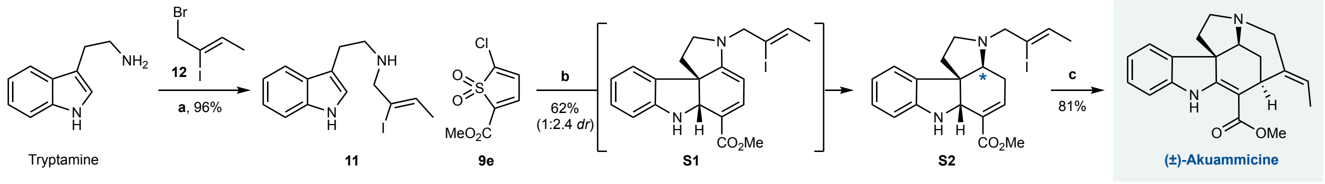

Scheme S1. Synthesis of (±)-akuammicine. a) MeCN, r.t.; b) MeCN, r.t. to 75 °C; then AcOH, $NaBH_3CN$, 65 °C; c) $Pd(OAc)_2$, PPh_3, Et_3N, 70 °C.

(*Z*)-1-Bromo-2-iodobut-2-ene, 12

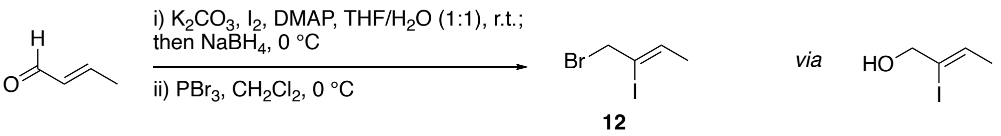

According to a modified literature procedure.[6] (i) To a stirred solution of *trans*-crotonaldehyde (8.40 g,

120 mmol, 1.0 equiv.) in THF (300 mL) and water (300 mL) was added potassium carbonate (20.0 g, 145 mmol, 1.2 equiv.) followed by iodine (46.0 g, 181 mmol, 1.5 equiv.) and *N,N*-dimethylaminopyridine (2.94 g, 24.1 mmol, 0.2 equiv.). The resulting mixture was stirred at room temperature for 2 h before it was cooled to 0 °C. Sodium borohydride (5.00 g, 132 mmol, 1.1 equiv.) was then added batchwise. The resulting solution was warmed to room temperature and stirred for 1 h before it was quenched with $Na_2S_2O_3$ (200 mL, sat. aq.). The layers were separated and the aqueous layer was extracted with Et_2O (3 × 150 mL). The combined organic layers were washed with water (200 mL), brine (200 mL), dried (Na_2SO_4) and concentrated under reduced pressure. The resulting (*Z*)-2-iodobut-2-en-1-ol was used directly in the next step without further purification.

(ii) To a stirred solution of crude (*Z*)-2-iodobut-2-en-1-ol (obtained above, 18.5 g, 93.4 mmol, 1.0 equiv.) in CH_2Cl_2 (250 mL) at 0 °C was added phosphorous tribromide (10.5 mL, 111 mmol, 1.2 equiv.) dropwise. The resulting mixture was warmed to room temperature and stirred for 2.5 h before it was quenched with $NaHCO_3$ (150 mL, sat. aq.). The layers were separated and the aqueous layer was extracted CH_2Cl_2 (3 × 100 mL), the combined organic layers were washed with water (200 mL), brine (200 mL), dried (Na_2SO_4) and concentrated under reduced pressure. Flash column chromatography (silica gel, pentane:Et_2O 1:0→10:1) afforded compound **12** (26.3 g, 101 mmol, 84% over two steps) as a yellow oil. All physical characteristics of **12** are identical to those reported in literature.[6]

(*Z*)-*N*-(2-(1*H*-indol-3-yl)ethyl)-2-iodobut-2-en-1-amine, 11

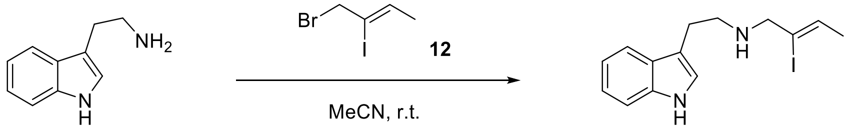

Tryptamine **11**

To a stirred solution of tryptamine (21.3 g, 133 mmol, 3.0 equiv.) in MeCN (450 mL) at room temperature was added **12** (11.6 g, 44.5 mmol, 1.0 equiv.) and the mixture was stirred for 18 h before it was concentrated under reduced pressure. Flash column chromatography (silica gel, pentane:Et_2O 4:1→0:1) afforded compound **11** (14.5 g, 42.6 mmol, 96%) as a brown sticky solid. **11**: R_f = 0.24 (silica gel, pentane:EtOAc 1:1); IR (film) ν_{max} 3417, 2914, 2849, 1456, 1352, 1108, 740 cm^{-1}; ^1H NMR (400 MHz, $CDCl_3$): δ 8.12 (s, 1H), 7.62 (ddt, *J* = 7.8, 1.4, 0.7 Hz, 1H), 7.36 (dt, *J* = 8.1, 0.9 Hz, 1H), 7.20 (ddd, *J* = 8.1, 7.1, 1.0 Hz, 1H), 7.13 (ddd, *J* = 8.0, 7.1, 1.1 Hz, 1H), 7.05 (d, *J* = 2.3 Hz, 1H), 5.77 (qt, *J* = 6.4, 1.2 Hz, 1H), 3.50 (s, 2H), 3.05-2.94 (m, 2H), 2.94-2.82 (m, 2H), 1.76 ppm (dt, *J* = 6.4, 1.1 Hz, 3H); ^{13}C

NMR (101 MHz, $CDCl_3$): δ 136.5, 131.5, 127.6, 122.1, 122.0, 119.4, 119.1, 114.1, 111.6, 111.3, 61.1, 47.7, 25.9, 21.8 ppm; HRMS calcd. For $C_{14}H_{18}IN_2^+$ $[M + H]^+$ 341.0509, found 341.0504.

Methyl 5-chlorothiophene-2-carboxylate 1,1-dioxide, 9e

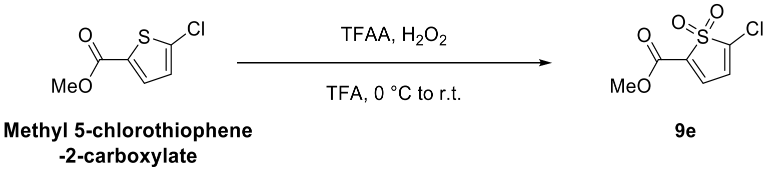

According to a modified literature procedure.[1] To a stirred solution of TFAA (11.2 mL, 80.5 mmol, 10.1 equiv.) and TFA (0.1 mL) at 0 °C was added H_2O_2 (30 wt% in H_2O, 3.22 mL, 31.5 mmol, 4.0 equiv.) dropwise. The resulting mixture was warmed to room temperature and stirred for 15 min. To this mixture at 0 °C was added a solution of methyl 5-chlorothiophene-2-carboxylate (1.40 g, 7.93 mmol, 1.0 equiv.) in TFA (1.5 mL) dropwise. The resulting mixture was warmed to room temperature and stirred for 18 h before it was concentrated under reduced pressure. The crude residue was recrystallised in $CHCl_3$:MeCN (4:1) to afford compound **9e** (1.21 g, 5.80 mmol, 73%) as a light yellow sticky solid. **9e**: R_f = 0.48 (silica gel, pentane:EtOAc 5:1); IR (film) ν_{max} 1720, 1549, 1438, 1321, 1258, 1155, 749 cm^{-1}; ^1H NMR (500 MHz, $CDCl_3$): δ 7.63 (d, J = 5.1 Hz, 1H), 6.75 (d, J = 5.1 Hz, 1H), 3.95 ppm (s, 3H); ^{13}C NMR (126 MHz, $CDCl_3$): δ 157.6, 138.7, 137.1, 132.7, 120.8, 53.5 ppm; HRMS calcd. For $C_6H_6ClO_4S^+$ $[M + H]^+$ 208.9670, found 208.9672.

Methyl (3a*S*,6a*R*)-3-((*Z*)-2-iodobut-2-en-1-yl)-2,3,3a,4,6a,7-hexahydro-1*H*-pyrrolo[2,3-*d*]carbazole-6-carboxylate, S2 and S2'

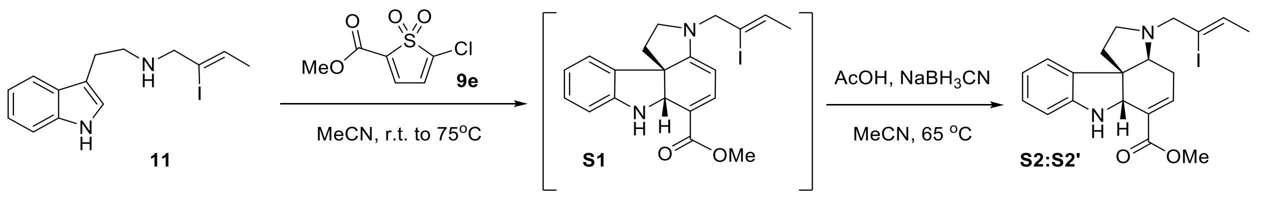

To a stirred solution of **9e** (120 mg, 0.58 mmol, 1.0 equiv.) in MeCN (23 mL) at room temperature was added **11** (200 mg, 0.59 mmol, 1.0 equiv.). The resulting mixture was stirred for 14 h before additional **11** (240 mg, 0.71 mmol, 1.2 equiv.) was added. The resulting mixture was warmed to 75 °C and stirred

for 47 h before it was cooled to 65 °C, and AcOH (0.49 mL, 8.56 mmol, 14.8 equiv.) was added. The resulting mixture was stirred for 15 min at 65 °C followed by addition of $NaBH_3CN$ (360 mg, 5.73 mmol, 9.9 equiv.) in MeOH (10 mL). The resulting mixture was stirred at 65 °C for 30 min before it was cooled to room temperature and quenched by slow addition of $NaHCO_3$ (40 mL, sat. aq.), then diluted with water (10 mL) and CH_2Cl_2 (50 mL). The layers were separated and the aqueous layer was extracted with CH_2Cl_2 (3 × 30 mL), the combined organic layers were washed with water (75 mL), brine (75 mL), dried (Na_2SO_4) and concentrated under reduced pressure. Flash column chromatography (silica gel, pentane:Et_2O 20:1→3:1) afforded compounds **S2** and its C3a-epimer **S2'** (160 mg combined mass, 0.36 mmol, 62%, 1:2.4 *dr*) as a brown foam. These diastereomers could be separated for the purposes of characterisation, and for the subsequent Heck cyclisation of **S2**.

S2 (desired): R_f = 0.84 (silica gel, pentane:Et_2O 1:1); IR (film) ν_{max} 3399, 2922, 1704, 1485, 1250, 742 cm^{-1}; ^1H NMR (400 MHz, $CDCl_3$): δ 7.11 (d, *J* = 7.3 Hz, 1H), 7.06-7.00 (m, 2H), 6.71 (t, *J* = 7.4 Hz, 1H), 6.57 (d, *J* = 7.7 Hz, 1H), 5.86 (q, *J* = 6.4 Hz, 1H), 4.57 (s, 1H), 4.31 (s, *J* = 1.6 Hz, 1H), 3.78 (s, 3H), 3.58 (d, *J* = 14.3 Hz, 1H), 3.30 (d, *J* = 14.2 Hz, 1H), 3.17-3.13 (m, 1H), 3.10 (ddd, *J* = 9.8, 8.6, 6.6 Hz, 1H), 2.69 (td, *J* = 9.8, 4.7 Hz, 1H), 2.42-2.34 (m, 1H), 2.29-2.22 (m, 1H), 2.16 (ddd, *J* = 13.1, 8.6, 4.7 Hz, 1H), 1.97 (ddd, *J* = 12.8, 9.8, 6.6 Hz, 1H), 1.77 ppm (d, *J* = 6.4 Hz, 3H); ^{13}C NMR (126 MHz, $CDCl_3$): δ 167.3, 150.0, 139.2, 132.5, 130.6, 129.9, 128.0, 123.1, 118.4, 109.1, 108.9, 65.2, 62.3, 61.1, 53.4, 51.6, 50.2, 37.5, 25.1, 21.6 ppm; HRMS calcd. For $C_{20}H_{24}IN_2O_2^+$ $[M + H]^+$ 451.0877, found 451.0875.

S2' (undesired): R_f = 0.80 (silica gel, pentane: Et_2O 1:1); IR (film) ν_{max} 3394, 2918, 1700, 1477, 1248, 739 cm^{-1}; ^1H NMR (400 MHz, $CDCl_3$): δ 7.79 (d, *J* = 8.0 Hz, 1H), 7.08-6.99 (m, 2H), 6.74 (t, *J* = 7.0 Hz, 1H), 6.61 (d, *J* = 7.7 Hz, 1H), 5.93 (q, *J* = 6.3 Hz, 1H), 4.61 (s, 1H), 4.28 (s, 1H), 3.75 (s, 3H), 3.71 (d, *J* = 13.5 Hz, 1H), 3.27 (dt, *J* = 10.0, 8.1 Hz, 1H), 3.16 (d, *J* = 13.5 Hz, 1H), 2.81 (dd, *J* = 10.7, 4.5 Hz, 1H), 2.47-2.37 (m, 2H), 2.28 (ddt, *J* = 18.0, 10.7, 2.3 Hz, 1H), 2.10-2.00 (m, 1H), 1.98-1.90 (m, 1H), 1.84 ppm (d, *J* = 6.3 Hz, 3H); ^{13}C NMR (101 MHz, $CDCl_3$): δ 167.4, 149.1, 141.4, 132.2, 131.9, 131.2, 127.9, 126.4, 119.0, 110.3, 110.2, 66.6, 64.7, 63.9, 54.7, 51.9, 50.6, 36.3, 28.5, 21.8 ppm; HRMS calcd. For $C_{20}H_{24}IN_2O_2^+$ $[M + H]^+$ 451.0877, found 451.0886.

(±)-Akuammicine

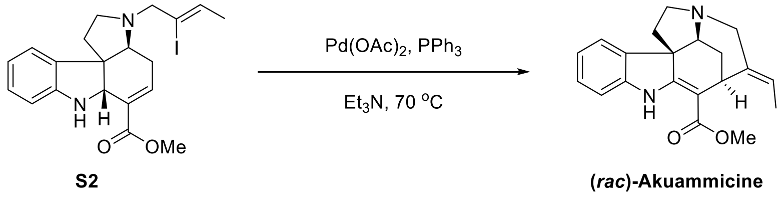

To a stirred solution of **S2** (144 mg, 0.32 mmol, 1.0 equiv.) in Et_3N (10.0 mL) at room temperature was added PPh_3 (33.5 mg, 0.13 mmol, 0.4 equiv.) and $Pd(OAc)_2$ (14.4 mg, 64.1 μmol, 0.2 equiv.). The resulting mixture was warmed to 70 °C and stirred for 5 h before it was concentrated under reduced pressure. Flash column chromatography (silica gel, CH_2Cl_2:MeOH:NH_4OH 20:1:1) afforded **(±)-akuammicine** (83.6 mg, 0.26 mmol, 81%) as a tan waxy solid. All physical characteristics of (*rac*)-akuammicine are identical to those reported for the asymmetric synthesis below (see page S19).

(5-Chlorothiophen-2-yl)((3a*S*,6*R*,7a*R*)-8,8-dimethyl-2,2-dioxidotetrahydro-3*H*-3a,6-methanobenzo[*c*]isothiazol-1(4*H*)-yl)methanone, S3

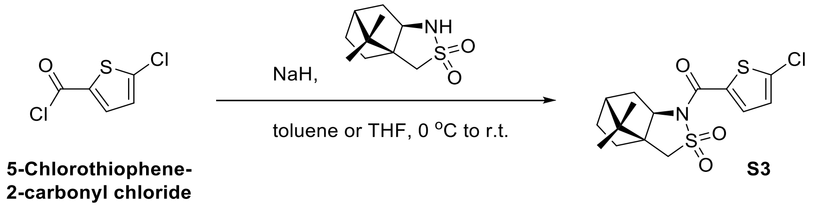

To a stirred solution of (1*S*)-(–)-2,10-camphorsultam (10.0 g, 46.4 mmol, 1.0 equiv.) in toluene or THF (200 mL) at 0 °C was added NaH (60% in oil, 2.79 g, 69.8 mmol, 1.5 equiv.). The resulting mixture was stirred for 1 h. To the resulting mixture at 0 °C was added 5-chlorothiophene-2-carbonyl chloride (10.9 mL, 90.3 mmol, 2.0 equiv.). The resulting mixture was warmed to room temperature and stirred for 14 h before it was quenched slowly with water (100 mL). The layers were separated and the aqueous layer was extracted with Et_2O (3 × 80 mL), the combined organic layers were washed with water (175 mL), brine (175 mL), dried (Na_2SO_4) concentrated under reduced pressure. The residue was washed with hot pentane (50 mL) to afford compound **S3** (16.4 g, 45.6 mmol, 98%) as a white solid. **S1**: R_f = 0.18 (silica gel, pentane:EtOAc 9:1); $[\alpha]_D^{25}$ = –107 (*c* = 0.66, $CHCl_3$); IR (film) ν_{max} 2958, 1660, 1425, 1341, 752 cm^{-1}; ^1H NMR (500 MHz, $CDCl_3$): δ 7.87 (d, *J* = 4.2 Hz, 1H), 6.93 (d, *J* = 4.2 Hz, 1H), 4.20 (dd, *J* = 7.7, 4.7 Hz, 1H), 3.57 (d, *J* = 13.6 Hz, 1H), 3.48 (d, *J* = 13.7 Hz, 1H), 2.08 (dd, *J* = 13.7, 7.7 Hz, 1H), 2.04-

1.95 (m, 2H), 1.95-1.87 (m, 2H), 1.49-1.43 (m, 1H), 1.43-1.35 (m, 1H), 1.28 (s, 3H), 1.02 ppm (s, 3H); ^{13}C NMR (126 MHz, $CDCl_3$): δ 161.5, 139.3, 135.4, 134.3, 127.4, 66.6, 53.9, 48.4, 48.0, 45.3, 38.4, 33.4, 26.6, 21.4, 20.1 pm; HRMS calcd. For $C_{15}H_{19}ClNO_3S_2^+$ [M + H]$^+$ 360.0489, found 360.0480.

(5-Chloro-1,1-dioxidothiophen-2-yl)((3a*S*,6*R*,7a*R*)-8,8-dimethyl-2,2-dioxidotetrahydro-3*H*-3a,6-methanobenzo[*c*]isothiazol-1(4*H*)-yl)methanone, 9a

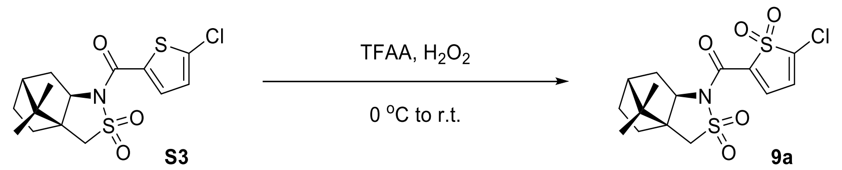

According to a modified literature procedure.[1] To a stirred solution of TFAA (64.3 mL, 463 mmol, 10.2 equiv.) at 0 °C was added H_2O_2 (30 wt% in H_2O, 16.3 mL, 160 mmol, 3.5 equiv.) dropwise. The resulting mixture was warmed to room temperature and stirred for 15 min. To the resulting mixture at 0 °C was added **S3** (16.4 g, 45.6 mmol, 1.0 equiv.). The resulting mixture was warmed to room temperature and stirred for 14 h before it was concentrated under reduced pressure. The crude residue was recrystallised in $CHCl_3$:MeCN (3:1) to afford compound **9a** (15.7 g, 40.1 mmol, 88%) as a light yellow solid. **9a**: R_f = 0.38 (silica gel, pentane:EtOAc 3:1); $[\alpha]_D^{25}$ = –124 (c = 0.93, $CHCl_3$); IR (film) ν_{max} 2965, 1661, 1425, 1338, 737 cm^{-1}; ^{1}H NMR (400 MHz, $CDCl_3$): δ 7.92 (d, J = 5.3 Hz, 1H), 6.75 (d, J = 5.3 Hz, 1H), 4.15 (dd, J = 7.6, 4.9 Hz, 1H), 3.59 (d, J = 13.7 Hz, 1H), 3.52 (d, J = 13.7 Hz, 1H), 2.13 (dd, J = 13.9, 7.7 Hz, 1H), 2.09-1.85 (m, 4H), 1.50-1.36 (m, 2H), 1.20 (s, 3H), 1.01 ppm (s, 3H); ^{13}C NMR (101 MHz, $CDCl_3$): δ 157.1, 139.3, 135.1, 133.2, 121.0, 66.5, 53.8, 48.8, 48.0, 45.2, 38.5, 33.4, 26.5, 21.3, 20.0 ppm; HRMS calcd. For $C_{15}H_{19}ClNO_5S_2^+$ [M + H]$^+$ 392.0388, found 392.0375.

((3a*S*,6*R*,7a*R*)-8,8-Dimethyl-2,2-dioxidotetrahydro-3*H*-3a,6-methanobenzo[*c*]isothiazol-1(4*H*)-yl)((3a*S*,6a*R*,11b*S*)-3-((*Z*)-2-iodobut-2-en-1-yl)-2,3,3a,4,6a,7-hexahydro-1*H*-pyrrolo[2,3-*d*]carbazol-6-yl)methanone, 14 and 14'

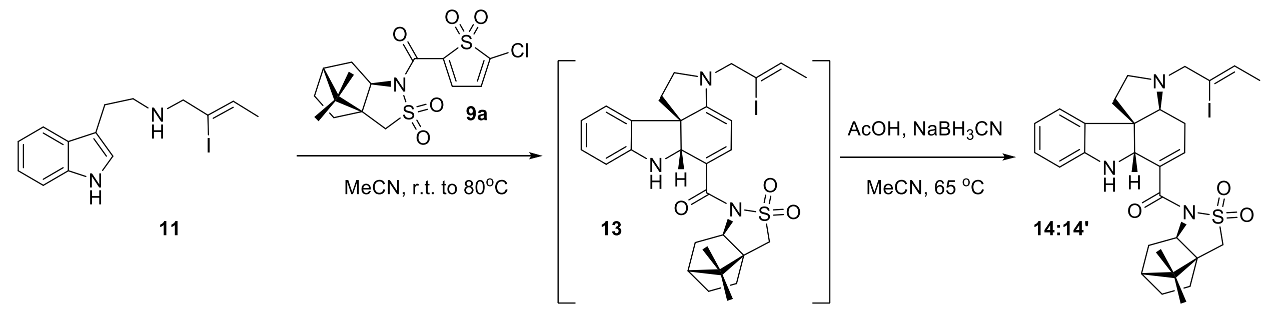

To a stirred solution of **11** (217 mg, 0.64 mmol, 1.0 equiv.) in MeCN (26 mL) at room temperature was added **9a** (250 mg, 0.64 mmol, 1.0 equiv.). The resulting mixture was stirred for 14 h before additional **11** (261 mg, 0.77 mmol, 1.2 equiv.) was added. The resulting mixture was warmed to 80 °C and stirred for 40 h before it was cooled to 65 °C, and AcOH (0.55 mL, 9.61 mmol, 15.0 equiv.) was added. The resulting mixture was stirred for 15 min at 65 °C followed by addition of $NaBH_3CN$ (401 mg, 6.38 mmol, 10.0 equiv.) in MeOH (6.5 mL). The resulting mixture was stirred at 65 °C for 30 min before it was cooled to room temperature and quenched by slow addition of $NaHCO_3$ (20 mL, sat. aq.), then diluted with water (10 mL) and CH_2Cl_2 (20 mL). The layers were separated and the aqueous layer was extracted with CH_2Cl_2 (3 × 30 mL), the combined organic layers were washed with water (50 mL), brine (50 mL), dried (Na_2SO_4) and concentrated under reduced pressure. Flash column chromatography (silica gel, pentane:Et_2O 9:1→3:1) afforded compounds **14** and its C3a-epimer **14'** (276 mg combined mass, 0.44 mmol, 68%, 1:1.4 *dr*) as a brown foam, along with recovered tryptamine **11** (240 mg, 0.71 mmol). These diastereomers could be separated for the purposes of characterisation, and for the subsequent reduction of **14**.

^{1}H NMR (400 MHz, $CHCl_3$) of semi-purified dienamine 13

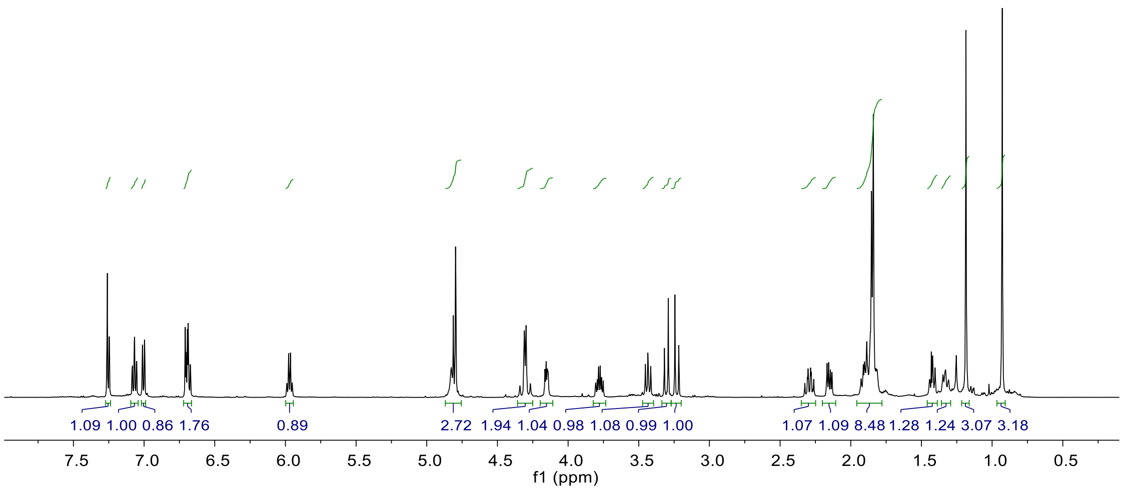

^{13}C NMR (101 MHz, $CHCl_3$) of semi-purified dienamine 13

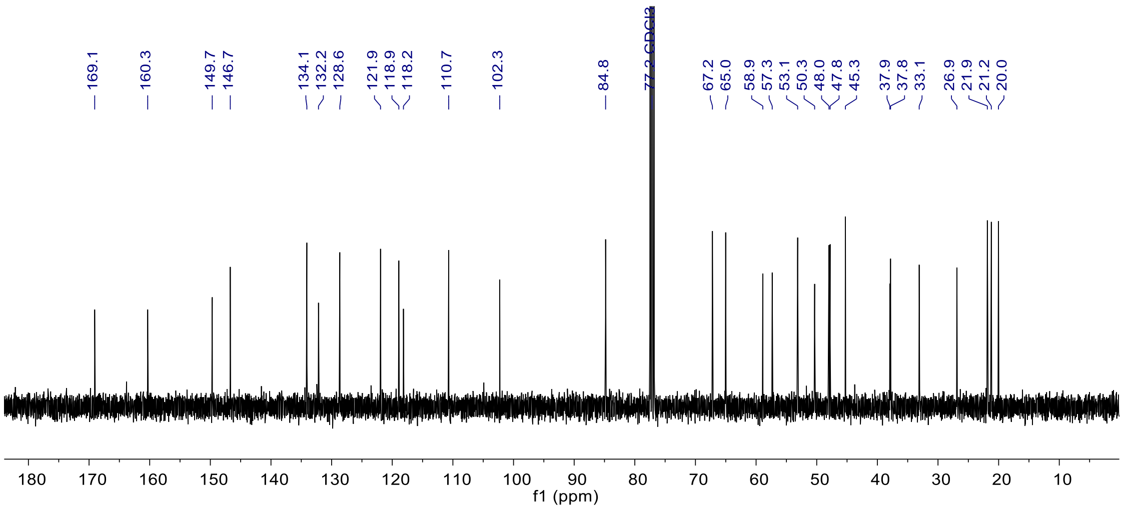

14 (desired): R_f = 0.54 (silica gel, pentane:CH_2Cl_2:Et_2O 2:1:1); $[\alpha]_D^{25}$ = –54.7 (*c* = 1.20, $CHCl_3$); IR (film) ν_{max} 2981, 2889, 1683, 1487, 1382, 913, 738 cm^{-1}; ^{1}H NMR (400 MHz, $CDCl_3$): δ 7.08 (d, *J* = 7.4 Hz, 1H), 7.01 (t, *J* = 7.6 Hz, 1H), 6.80 (t, *J* = 4.7 Hz, 1H), 6.71 (t, *J* = 7.4 Hz, 1H), 6.57 (d, *J* = 7.7 Hz, 1H), 5.85 (q, *J* = 6.4 Hz, 1H), 4.52 (s, 1H), 4.13 (dd, *J* = 7.6, 4.3 Hz, 1H), 4.02 (s, 1H), 3.55 (d, *J* = 14.4 Hz, 1H), 3.48 (d, *J* = 13.6 Hz, 1H), 3.38 (d, *J* = 13.6 Hz, 1H), 3.24 (d, *J* = 14.4 Hz, 1H), 3.19-3.10 (m, 1H), 3.04 (t, *J* = 3.6 Hz, 1H), 2.59 (td, *J* = 9.1, 6.5 Hz, 1H), 2.38 (t, *J* = 3.9 Hz, 2H), 2.17-2.00 (m, 3H), 1.98-1.87 (m, 4H), 1.76 (d, *J* = 6.4 Hz, 3H), 1.49-1.36 (m, 2H), 1.23 (s, 3H), 0.99 ppm (s, 3H); ^{13}C NMR (101 MHz, $CDCl_3$): δ 170.7, 150.7, 141.4, 133.9, 133.4, 130.7, 128.0, 123.1, 118.8, 109.8, 109.5, 66.0, 65.4, 62.3, 54.4, 53.8, 51.6, 48.0, 47.8, 45.3, 38.3, 38.2, 33.2, 26.7, 25.6, 21.7, 21.3, 20.0 ppm; HRMS calcd. For $C_{29}H_{37}IN_3O_3S^+$ $[M + H]^+$ 634.1595, found 634.1566.

14' (undesired): R_f = 0.50 (silica gel, pentane:CH_2Cl_2:Et_2O 2:1:1); $[\alpha]_D^{25}$ = –168 (*c* = 1.10, $CHCl_3$); IR (film) ν_{max} 2989, 2920, 1670, 1480, 1337, 912, 737 cm^{-1}; ^{1}H NMR (400 MHz, $CDCl_3$): δ 7.77 (d, *J* = 7.5 Hz, 1H), 7.04 (d, *J* = 7.5 Hz, 1H), 6.76 (t, *J* = 7.9 Hz, 1H), 6.64-6.58 (m, 2H), 5.92 (q, *J* = 6.3 Hz, 1H), 4.51 (s, 1H), 4.10-4.04 (m, 1H), 3.92 (s, 1H), 3.68 (d, *J* = 16.5 Hz, 1H), 3.39 (d, *J* = 13.6 Hz, 1H), 3.33 (d, *J* = 13.7 Hz, 1H), 3.30-3.23 (m, 1H), 3.16 (d, *J* = 13.5 Hz, 1H), 2.81 (dd, *J* = 10.1, 4.9 Hz, 1H), 2.46-2.29 (m, 3H), 2.08 (d, *J* = 12.0 Hz, 2H), 1.98-1.85 (m, 5H), 1.82 (d, *J* = 6.3 Hz, 3H), 1.49-1.31 (m, 2H), 1.16 (s, 3H), 0.96 ppm (s, 3H); ^{13}C NMR (126 MHz, $CDCl_3$): δ 170.8, 149.6, 140.0, 136.6, 131.9, 131.2, 127.9, 126.2, 119.6, 111.6, 110.3, 66.7, 65.3, 64.8, 64.6, 54.5, 53.8, 50.8, 48.2, 48.0, 45.1, 38.2, 36.4, 33.2, 28.1, 26.7, 21.9, 21.2, 20.0 ppm; HRMS calcd. For $C_{29}H_{37}IN_3O_3S^+$ $[M + H]^+$ 634.1595, found 634.1568.

Table S1. Optimisation studies for reduction of dienamine 13.

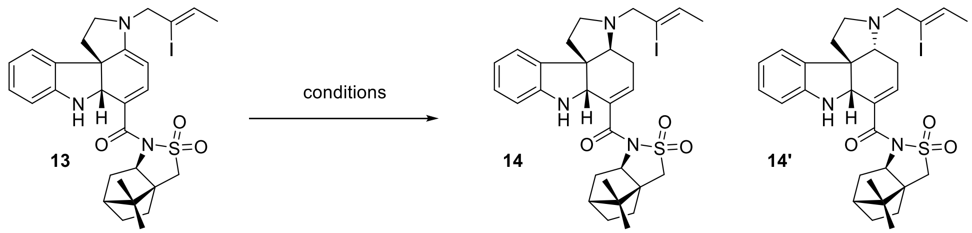

Entry	conditions[a]	14:14'[b]
1	AcOH (15 equiv.), $NaBH_3CN$ (10 equiv.), MeCN:MeOH, rt	N.R.
2	AcOH (15 equiv.), $NaBH_3CN$ (10 equiv.), MeCN:MeOH, 65 °C	1:1.4
3	HCl (15 equiv.), $NaBH_3CN$ (10 equiv.), MeCN:MeOH, 65 °C	decomposed
4	HCOOH (15 equiv.), $NaBH_3CN$ (10 equiv.), MeCN:MeOH, 65 °C	decomposed
5	TFA (15 equiv.), $NaBH_3CN$ (10 equiv.), MeCN:MeOH, 65 °C	decomposed
6	CSA (15 equiv.), $NaBH_3CN$ (10 equiv.), MeCN:MeOH, 65 °C	decomposed
7	PTSA (15 equiv.), $NaBH_3CN$ (10 equiv.), MeCN:MeOH, 65 °C	decomposed
8	PhCOOH (15 equiv.), $NaBH_3CN$ (10 equiv.), MeCN:MeOH, 65 °C	1:1.2
9	3-Chlorobenzoic acid (15 equiv.), $NaBH_3CN$ (10 equiv.), MeCN:MeOH, 65 °C	1:1.5
10	4-Bromophenyl acetic acid (15 equiv.), $NaBH_3CN$ (10 equiv.), MeCN:MeOH, 65 °C	1:1.2
11	2-Picolinic acid (15 equiv.), $NaBH_3CN$ (10 equiv.), MeCN:MeOH, 65 °C	1:2
12	Isonicotinic acid (15 equiv.), $NaBH_3CN$ (10 equiv.), MeCN:MeOH, 65 °C	1:1.3
13	PhCOOH (15 equiv.), $NaBH_4$ (10 equiv.), MeCN:MeOH, 65 °C	N.R.
14	PhCOOH (15 equiv.), $LiBH_4$ (10 equiv.), MeCN:MeOH, 65 °C	decomposed
15	PhCOOH (15 equiv.), $NaH(OAc)_3$ (10 equiv.), MeCN:MeOH, 65 °C	1:9
16	PhCOOH (15 equiv.), 2-Picoline Borane (10 equiv.), MeCN:MeOH, 65 °C	1:9
17[c]	PhCOOH (15 equiv.), $NaBH_3CN$ (10 equiv.), MeCN:MeOH, 65 °C	1:2

a. Reactions were performed on 0.03 mmol scale; *b*. Determined by ^{1}H NMR analysis of crude mixture; *c* 3.83 mmol scale

(4-Bromothiophen-2-yl)((3a*S*,6*R*,7a*R*)-8,8-dimethyl-2,2-dioxidotetrahydro-3*H*-3a,6-methanobenzo[*c*]isothiazol-1(4*H*)-yl)methanone, 17

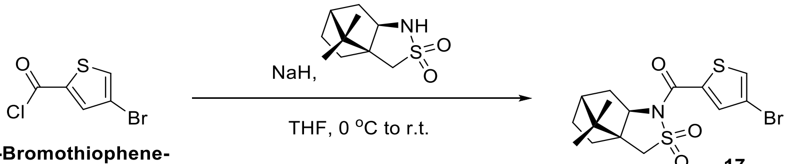

4-Bromothiophene-2-carbonyl chloride **17**

To a solution of (1*S*)-(–)-2,10-camphorsultam (1.79 g, 8.31 mmol, 1.0 equiv.) in THF (60 mL) at 0 °C was added NaH (60% in oil, 483 mg, 12.1 mmol, 1.46 equiv.). The resulting mixture was stirred for 15 min at 0 °C, then a solution of 4-bromothiophene-2-carbonyl chloride (2.72 g, 12.1 mmol, 1.46 equiv.) in THF (20.0 mL) was added. The resulting mixture was warmed to room temperature and stirred for 16 h before it was diluted slowly with water (50 mL). The layers were separated and the aqueous layer was extracted with EtOAc (3 × 50 mL), the combined organic layers were washed with brine (100 mL), dried (Na_2SO_4) concentrated under reduced pressure. Flash column chromatography (silica gel, pentane:Et_2O 9:1→1:1) afforded compound **17** (3.33 g, 8.24 mmol, 99%) as a white foam. **17**: R_f = 0.18 (silica gel, pentane:EtOAc 6:1); $[\alpha]_D^{25}$ = –137 (*c* = 0.44, $CHCl_3$); IR (film) ν_{max} 3112, 2962, 1660, 1407, 1403, 1336, 1292, 734 cm^{-1}; ^{1}H NMR (400 MHz, $CDCl_3$): δ 7.96 (d, *J* = 1.4 Hz, 1H), 7.52 (d, *J* = 1.4 Hz, 1H), 4.20 (dd, *J* = 7.7, 4.7 Hz, 1H), 3.59 (d, *J* = 13.7 Hz, 1H), 3.48 (d, *J* = 13.6 Hz, 1H), 2.09 (dd, *J* = 13.7, 7.7 Hz, 1H), 2.06-1.85 (m, 4H), 1.50-1.35 (m, 2H), 1.28 (s, 3H), 1.02 ppm (s, 3H); ^{13}C NMR (101 MHz, $CDCl_3$): δ 161.3, 137.6, 136.1, 130.8, 110.5, 66.6, 53.8, 48.4, 48.0, 45.3, 38.5, 33.3, 26.6, 21.4, 20.1 ppm; HRMS calcd. For $C_{15}H_{19}BrNO_3S_2^+$ $[M + H]^+$ 403.9984, found 403.9978.

(4-Bromo-1,1-dioxidothiophen-2-yl)((3a*S*,6*R*,7a*R*)-8,8-dimethyl-2,2-dioxidotetrahydro-3*H*-3a,6-methanobenzo[*c*]isothiazol-1(4*H*)-yl)methanone, 9d

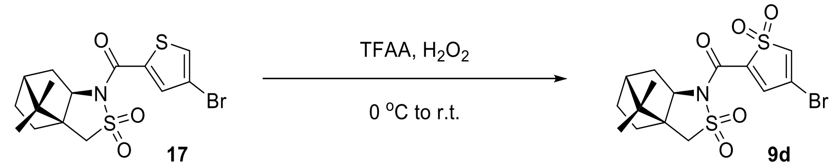

17 **9d**

According to a modified literature procedure.[1] To a stirred solution of TFAA (2.68 mL, 19.2 mmol, 10.0 equiv.) at 0 °C was added H_2O_2 (30 wt % in H_2O, 0.69 mL, 6.73 mmol, 3.5 equiv.) dropwise. The

resulting mixture was stirred for 15 min, then **17** (778 mg, 1.92 mmol, 1.0 equiv.) was added. The resulting mixture was warmed to room temperature and stirred for 64 h before it was concentrated under reduced pressure. The resulting residue was azeotroped with toluene (3 × 5 mL) to afford compound **9d** as brown foam, which was used directly in the next step without further purification.

^{1}H NMR (400 MHz, $CHCl_3$) of 4-bromothiophene *S*,*S*-dioxide (crude), 9d

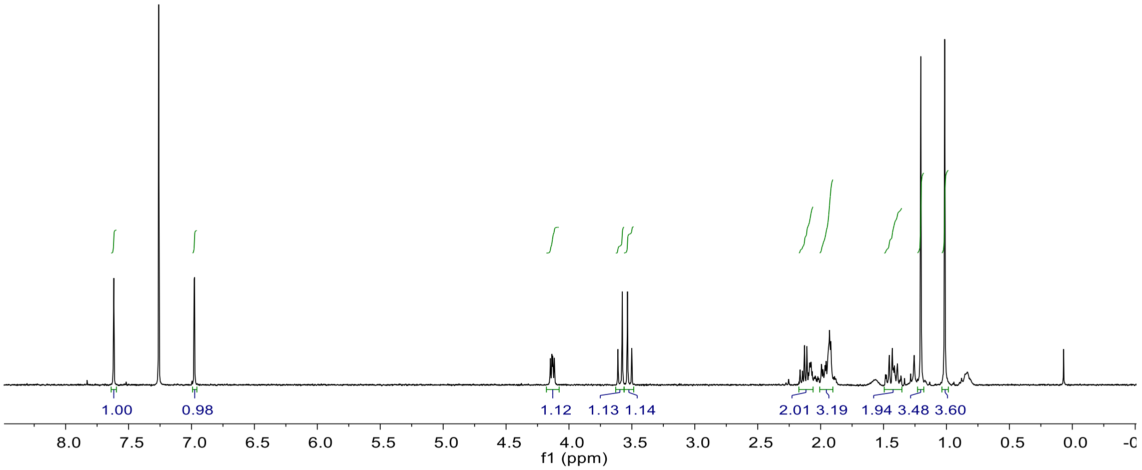

((6a*R*,11b*S*)-4-Bromo-2,3,3a,4,6a,7-hexahydro-1*H*-pyrrolo[2,3-*d*]carbazol-6-yl)((3a*S*,6*R*,7a*R*)-8,8-dimethyl-2,2-dioxidotetrahydro-3*H*-3a,6-methanobenzo[*c*]isothiazol-1(4*H*)-yl)methanone, 19

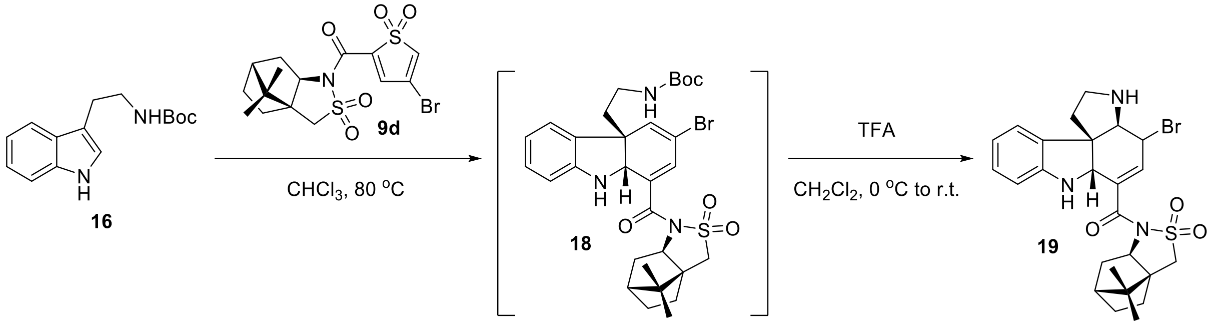

To a stirred solution of Boc-tryptamine **16**[7] (1.00 g, 3.84 mmol, 2.0 equiv.) in $CHCl_3$ (19.0 mL) at room temperature was added **9d** (crude, obtained above). The resulting mixture was warmed to 80 °C and stirred for 23 h, before it was cooled to 0 °C and added TFA (9.0 mL) (to the intermediate diene **18**). The resulting mixture was warmed to room temperature and stirred for 2.5 h before it was cooled to 0 °C and quenched by slow addition of Na_2CO_3 (200 mL, sat. aq.), and stirred for 20 h. The layers were separated and the aqueous layer was extracted with CH_2Cl_2 (3 × 50 mL), then the combined organic layers were dried (Na_2SO_4) and concentrated under reduced pressure. Flash column chromatography (silica gel,

pentane:EtOAc 9:1→4:6) afforded compound **19** (585 mg, 1.10 mmol, 57% over two steps) as a yellow foam. **19**: R_f = 0.22 (silica gel, pentane:EtOAc 4:6); $[\alpha]_D^{25}$ = –59.5 (c = 0.38, $CHCl_3$); IR (film) ν_{max} 3367, 2957, 2362, 1675, 1466, 1330, 742 cm^{-1}; ^1H NMR (400 MHz, $CDCl_3$): δ 7.07-7.01 (m, 2H), 6.76-6.70 (m, 2H), 6.57 (dt, J = 7.7, 0.8 Hz, 1H), 4.93 (ddd, J = 3.9, 2.3, 1.4 Hz, 1H), 4.54 (d, J = 1.4 Hz, 1H), 4.11 (br s, 1H), 4.08 (dd, J = 7.5, 4.8 Hz, 1H), 3.85 (dd, J = 4.1, 1.3 Hz, 1H), 3.49 (d, J = 13.7 Hz, 1H), 3.39 (d, J = 13.7 Hz, 1H), 3.28-3.16 (m, 2H), 2.37 (ddd, J = 12.3, 7.1, 5.0 Hz, 1H), 2.30 (br s, 1H), 2.23 (ddd, J = 12.7, 8.8, 7.4 Hz, 1H), 2.09-1.84 (m, 6H), 1.47-1.32 (m, 2H), 1.19 (s, 3H), 0.97 ppm (s, 3H); ^{13}C NMR (101 MHz, $CDCl_3$): δ 169.4, 150.6, 140.0, 135.0, 130.4, 128.6, 122.6, 119.2, 110.0, 66.9, 65.5, 61.1, 55.0, 53.8, 48.5, 48.2, 47.9, 45.2, 44.0, 41.7, 38.2, 33.2, 26.6, 21.2, 19.9 ppm; HRMS calcd. For $C_{25}H_{31}BrN_3O_3S^+$ $[M + H]^+$ 532.1248, found 532.1264.

^1H NMR (400 MHz, $CHCl_3$) of diene 18

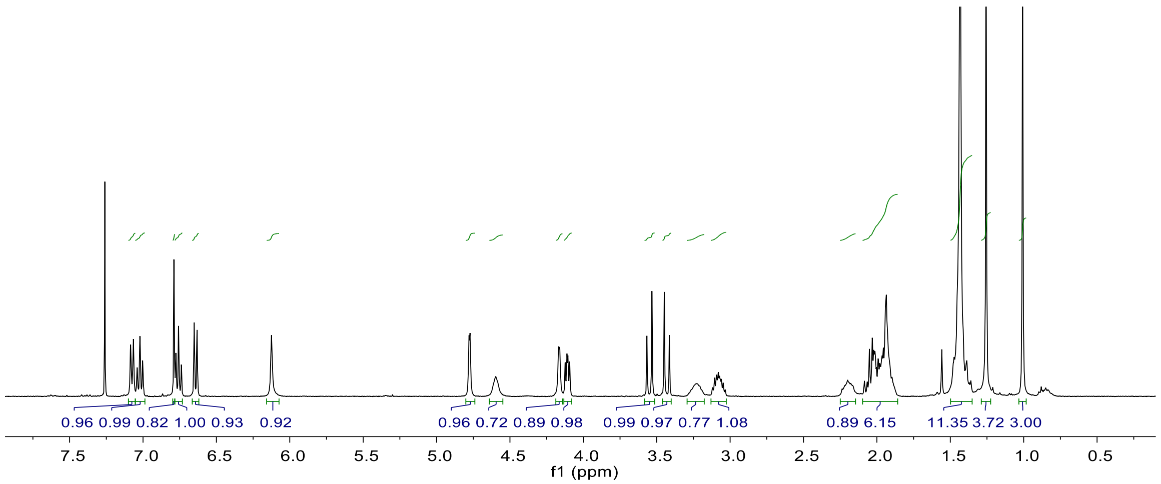

^{13}C NMR (101 MHz, $CHCl_3$) of diene 18

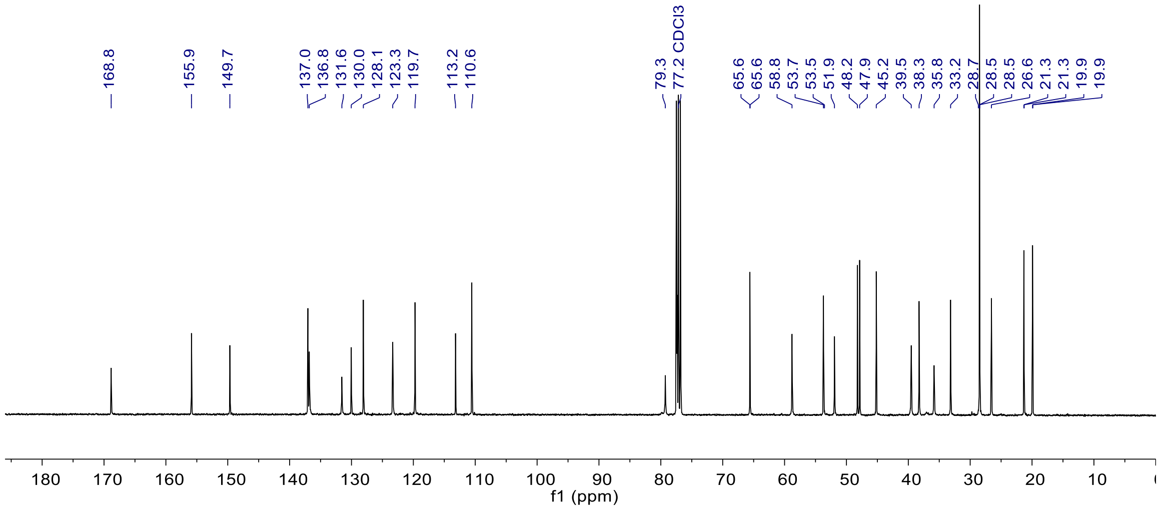

((3a*S*,6*R*,7a*R*)-8,8-dimethyl-2,2-dioxidotetrahydro-3*H*-3a,6-methanobenzo[*c*]isothiazol-1(4*H*)-yl)((3a*S*,6a*R*,11b*S*)-3-((*Z*)-2-iodobut-2-en-1-yl)-2,3,3a,4,6a,7-hexahydro-1*H*-pyrrolo[2,3-*d*]carbazol-6-yl)methanone, 14

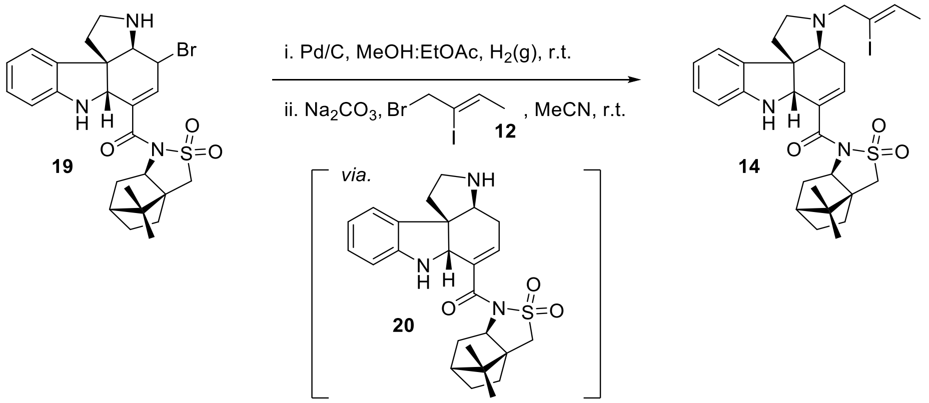

(i) To a solution of **19** (107 mg, 0.20 mmol, 1.0 equiv.) in MeOH:EtOAc (9:1, 2.0 mL) at room temperature was added Pd/C (10 wt% on carbon, 21.4 mg, 0.02 mmol, 0.1 equiv.). The resulting mixture was purged with 3 cycles of H_2/vacuum and stirred for a further 3 h under an H_2 atmosphere (balloon) before it was filtered through a pad of Celite®, eluting with EtOAc (5 mL). The filtrate was concentrated under reduced pressure to afford compound **20** as yellow foam, which was used directly in the next step without further purification.

(ii) To a solution of **20** (crude, obtained above) in MeCN (5.0 mL) at room temperature were added sodium carbonate (53.5 mg, 0.50 mmol, 2.5 equiv.) and **12** (52.2 mg, 0.20 mmol, 1.0 equiv.) in MeCN (1.5 mL). The resulting mixture was stirred for 17.5 h, then concentrated to half volume using a stream of nitrogen (*if a rotary evaporator is used the bath temperature must be kept at or below 20 °C to avoid alkylation of the indoline nitrogen with residual bromide **12***). The solution was directly purified by flash column chromatography (silica gel, pentane:EtOAc 9:1→7:3) which afforded compound **14** (87.0 mg, 0.14 mmol, 70% over two steps) as a light yellow foam. All the physical data of **14** are identical to those obtained from the reaction of **11** and **9a**, see page S11.

^{1}H NMR (500 MHz, $CDCl_3$) of semi-purified diamine 20

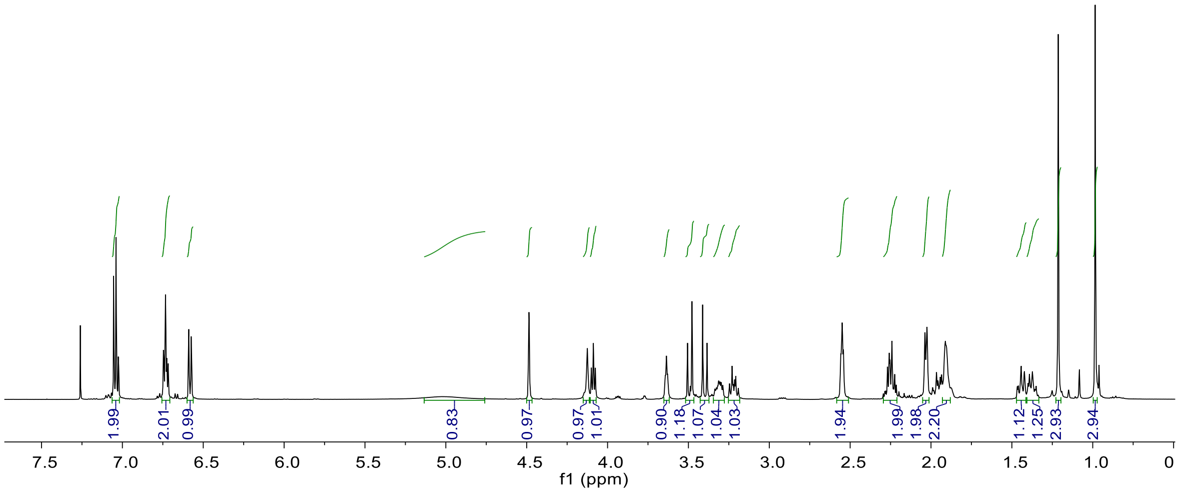

^{13}C NMR (126 MHz, $CDCl_3$) of semi-purified diamine 20

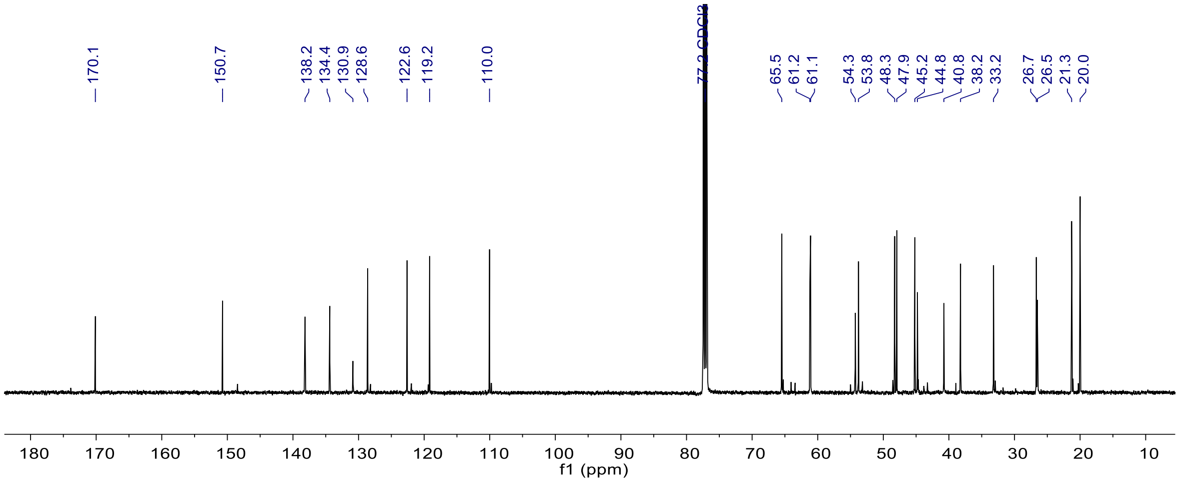

Methyl (3a*S*,6a*R*,11b*S*)-3-((*Z*)-2-iodobut-2-en-1-yl)-2,3,3a,4,6a,7-hexahydro-1*H*-pyrrolo[2,3-*d*]carbazole-6-carboxylate, 15

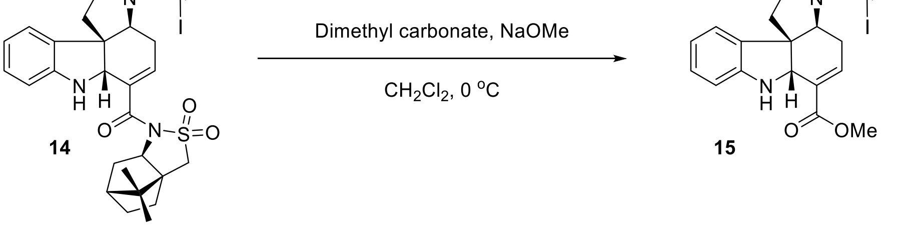

To a solution of **14** (302 mg, 0.48 mmol, 1.0 equiv.) in CH_2Cl_2 (9.50 mL) at 0 °C was added dimethyl carbonate (1.0 mL, 11.9 mmol, 25 equiv.) and sodium methoxide (25 wt% solution in MeOH, 0.30 mL,

1.31 mmol, 2.7 equiv.). The resulting mixture was stirred for 2 h at 0 °C, before it was quenched with H_2O (10 mL) and warmed to room temperature. The layers were separated and the aqueous layer was CH_2Cl_2 (3 × 10 mL), then the combined organic layers were dried (Na_2SO_4) and concentrated under reduced pressure. Flash chromatography (silica gel, pentane:Et_2O 4:1) afforded compound **15** (146 mg, 0.32 mmol, 67%) as yellow foam. All physical characteristics of **15** are identical to those reported for the racemic synthesis (see page S7). $[\alpha]_D^{25}$ = –110 (c = 0.48, $CHCl_3$).

Chiral HPLC: ChiralPak® IC, Flow: 1.0 mL/min; isocratic hexane:isopropanol 90:10

Racemic 15

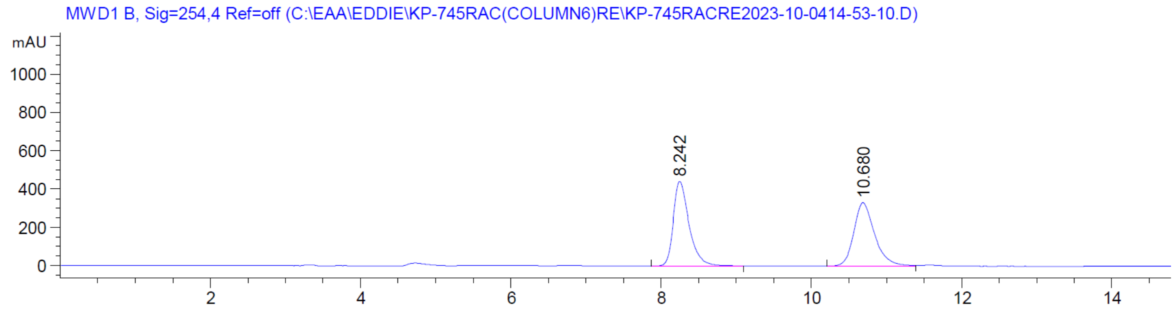

Eantiopure 15

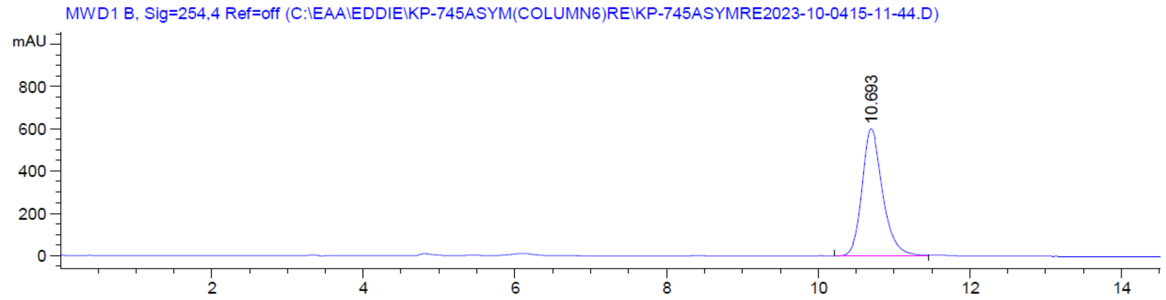

(–)-Akuammicine

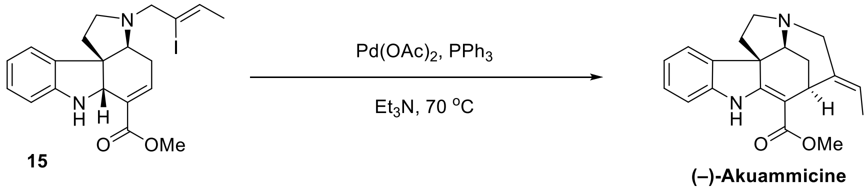

To a stirred solution of **15** (705 mg, 1.57 mmol, 1.0 equiv.) in Et_3N (50.0 mL) at room temperature was added PPh_3 (164 mg, 0.63 mmol, 0.4 equiv.) and $Pd(OAc)_2$ (70.3 mg, 0.31 mmol, 0.2 equiv.). The resulting mixture was warmed to 70 °C and stirred for 5 h before it was concentrated under reduced

pressure. Flash column chromatography (silica gel, CH_2Cl_2:MeOH:NH_4OH 20:1:1) afforded the **(–)-akuammicine** (376 mg, 1.17 mmol, 75%) as a tan waxy solid. **(–)-Akuammicine:** R_f = 0.26 (silica gel CH_2Cl_2:MeOH:NH_4OH 12:1:1); $[\alpha]_D^{25}$ = –736 (c = 0.30, $CHCl_3$); IR (film) ν_{max} 3362, 2946, 2861, 1670, 1603, 1238, 1101 cm^{-1}; ^1H NMR (500 MHz, $CDCl_3$): δ 9.00 (s, 1H), 7.23 (d, J = 7.4 Hz, 1H), 7.14 (td, J = 7.7, 1.2 Hz, 1H), 6.89 (td, J = 7.5, 1.0 Hz, 1H), 6.82 (d, J = 7.8 Hz, 1H), 5.35 (q, J = 6.8 Hz, 1H), 4.08-4.02 (m, 1H), 3.98-3.92 (m, 1H), 3.89 (d, J = 14.9 Hz, 1H), 3.80 (s, 3H), 3.27 (td, J = 12.6, 5.6 Hz, 1H), 3.02 (dd, J = 12.3, 6.6 Hz, 1H), 2.95 (d, J = 15.1 Hz, 1H), 2.51 (td, J = 12.6, 6.8 Hz, 1H), 2.41 (ddd, J = 13.4, 4.0, 2.2 Hz, 1H), 1.82 (dd, J = 12.4, 5.5 Hz, 1H), 1.60 (d, J = 7.0 Hz, 3H), 1.30 ppm (dt, J = 13.2, 3.0 Hz, 1H); ^{13}C NMR (126 MHz, $CDCl_3$): δ 168.1, 168.0, 143.6, 139.2, 137.1, 127.9, 121.1, 121.0, 120.9, 109.6, 101.4, 62.1, 57.7, 57.1, 56.4, 51.2, 46.4, 31.0, 29.9, 13.0 ppm; HRMS calcd. For $C_{20}H_{23}N_2O_2^+$ $[M + H]^+$ 323.1754, found 323.1753.

Table S2. ^1H and ^{13}C NMR ($CDCl_3$, ppm) comparison for akuammicine with that obtained from the MacMillan synthesis[8]

Macmillan (^1H, 500 MHz, ppm)	This work (^1H, 500 MHz, ppm)	Macmillan (^{13}C, 125 MHz, ppm)	This work (^{13}C, 126 MHz, ppm)
9.01 (s, 1H)	9.00 (s, 1H)	168.2	168.1
7.24 (d, J = 7.3 Hz, 1H)	7.23 (d, J = 7.4 Hz, 1H)	168.1	168.0
7.15 (td, J = 7.6, 0.9 Hz, 1H)	7.14 (td, J = 7.7, 1.2 Hz, 1H)	143.4	143.6
6.90 (t, J = 7.5 Hz, 1H)	6.89 (td, J = 7.5, 1.0 Hz, 1H)	139.4	139.2
6.83 (d, J = 7.8 Hz, 1H)	6.82 (d, J = 7.7 Hz, 1H)	137.0	137.1
5.34 (q, J = 6.7 Hz, 1H)	5.35 (q, J = 6.8 Hz, 1H)	127.7	127.9
4.04-4.02 (m, 1H)	4.08-4.02 (m, 1H)	120.9	121.1
3.95-3.92 (m, 1H)	3.98-3.92 (m, 1H)	120.8	121.0
3.89 (d, J = 15.0 Hz, 1H)	3.89 (d, J = 15.1 Hz, 1H)	120.6	121.0
3.81 (s, 3H)	3.80 (s, 3H)	109.4	109.6
3.27 (ddd, J = 12.6, 12.6, 5.6 Hz, 1H)	3.27 (td, J = 12.6, 5.7 Hz, 1H)	101.2	101.4
3.03 (dd, J = 12.4, 6.7 Hz, 1H)	3.02 (dd, J = 12.3, 6.6 Hz, 1H)	62.0	62.1
2.95 (d, J = 15.1 Hz, 1H)	2.95 (d, J = 15.1 Hz, 1H)	57.6	57.7
2.51 (ddd, J = 12.6, 12.6, 6.7 Hz, 1H)	2.51 (td, J = 12.6, 6.8 Hz, 1H)	57.0	57.1
2.43 (ddd, J = 13.4, 3.9, 2.2 Hz, 1H)	2.41 (ddd, J = 13.4, 4.0, 2.2 Hz, 1H)	56.4	56.4
1.82 (dd, J = 12.3, 5.5 Hz, 1H)	1.82 (dd, J = 12.4, 5.4 Hz, 1H)	51.0	51.2
1.61 (d, J = 6.9 Hz, 3H)	1.60 (d, J = 6.9 Hz, 3H)	46.4	46.4
1.30 (ddd, J = 13.1, 2.8, 2.8 Hz, 1H)	1.30 (dt, J = 13.4, 2.9 Hz, 1H)	30.9	31.0
		29.8	29.9
		12.9	13.0

(3a*S*,6a*R*,11b*S*)-3-((*Z*)-2-iodobut-2-en-1-yl)-2,3,3a,4,6a,7-hexahydro-1*H*-pyrrolo[2,3-*d*]carbazole-6-carbaldehyde, 21

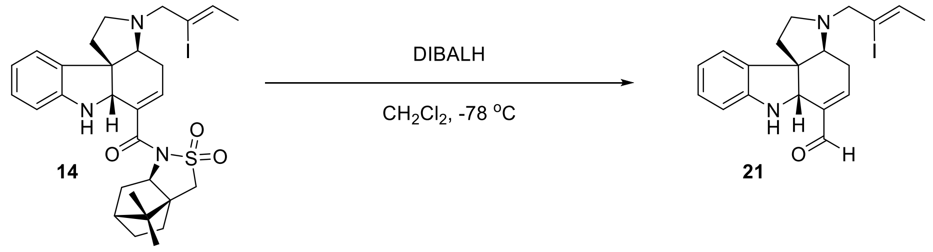

To a stirred solution of **14** (91.0 mg, 0.14 mmol, 1.0 equiv.) in CH_2Cl_2 (3.0 mL) at -78 °C was added DIBALH (1.0 M solution in hexane, 0.16 mL, 0.16 mmol, 1.1 equiv.). The resulting mixture was stirred for 1 h before it was quenched with Rochelle's salt (5 mL, sat. aq.) and stirred vigorously for 1 h. The layers were separated and the aqueous layer was extracted with CH_2Cl_2 (3 × 5 mL), the combined organic layers were washed with water (15 mL), brine (15 mL), dried (Na_2SO_4) and concentrated under reduced pressure. Flash column chromatography (silica gel, pentane:Et_2O 10:1→5:1) afforded compound **21** (40.5 mg, 96 μmol, 69%) as a yellow oil. **21**: R_f = 0.70 (silica gel, pentane:EtOAc 1:2); $[\alpha]_D^{25}$ = –126 (*c* = 1.22, $CHCl_3$); IR (film) ν_{max} 3397, 2920, 2819, 1678, 1651, 1607, 1484, 744 cm^{-1}; ^1H NMR (400 MHz, $CDCl_3$): δ 9.49 (s, 1H), 7.07 (d, *J* = 8.1 Hz, 1H), 7.02 (td, *J* = 7.6, 1.2 Hz, 1H), 6.81 (dd, *J* = 5.4, 2.9 Hz, 1H), 6.70 (td, *J* = 7.4, 1.0 Hz, 1H), 6.55 (d, *J* = 7.8 Hz, 1H), 5.85 (q, *J* = 6.4 Hz, 1H), 4.51 (s, 1H), 4.31 (s, 1H), 3.61 (dt, *J* = 14.2, 1.6 Hz, 1H), 3.30-3.22 (m, 2H), 3.14 (dd, *J* = 18.2, 7.0 Hz, 1H), 2.62 (td, *J* = 10.0, 4.4 Hz, 1H), 2.59-2.51 (m, 1H), 2.43-2.33 (m, 1H), 2.19 (ddd, *J* = 12.7, 8.4, 4.4 Hz, 1H), 1.94 (ddd, *J* = 12.8, 10.1, 6.9 Hz, 1H), 1.78 ppm (d, *J* = 6.4 Hz, 3H); ^{13}C NMR (126 MHz, $CDCl_3$): δ 195.0, 150.6, 150.5, 141.0, 131.7, 131.0, 128.4, 123.1, 118.7, 109.5, 109.3, 65.6, 63.0, 59.7, 53.6, 50.7, 37.7, 26.2, 21.8 ppm; HRMS calcd. For $C_{19}H_{22}IN_2O^+$ $[M + H]^+$ 421.0771, found 421.0758.

(–)-Norfluorocurarine

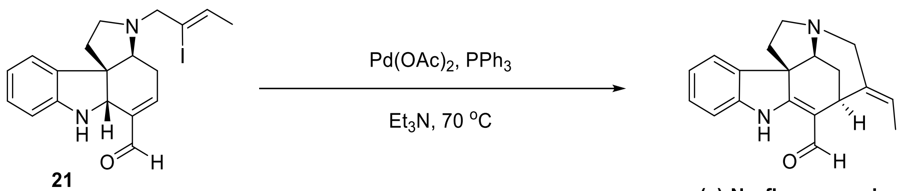

To a stirred solution of **21** (12.2 mg, 29.0 μmol, 1.0 equiv.) in Et_3N (3.0 mL) at room temperature was added PPh_3 (4.6 mg, 17.5 μmol, 0.6 equiv.) and $Pd(OAc)_2$ (2.0 mg, 8.9 μmol, 0.3 equiv.). The resulting mixture was purged with nitrogen for 20 min before it was warmed to 70 °C and stirred for 5.5 h. The resulting mixture was cooled to room temperature and concentrated under reduced pressure. Flash column chromatography (silica gel, CH_2Cl_2:MeOH:NH_4OH 20:1:1) afforded **(–)-norfluorocurarine** (6.3 mg, 21.5 μmol, 74%) as a yellow waxy solid. **(–)-Norfluorocurarine:** R_f = 0.26 (silica gel CH_2Cl_2:MeOH:NH_4OH 12:1:1); $[\alpha]_D^{25} = -1080$ (c = 0.12, $CHCl_3$); IR (film) ν_{max} 3350, 2900, 1648, 1561, 1466, 1380, 1107, 761 cm^{-1}; ^1H NMR (500 MHz, $CDCl_3$): δ 10.32 (br s, 1H), 9.36 (s, 1H), 7.29 (d, J = 7.4 Hz, 1H), 7.20 (t, J = 7.4 Hz, 1H), 6.98 (t, J = 7.5 Hz, 1H), 6.92 (d, J = 7.8 Hz, 1H), 5.40 (q, J = 6.7 Hz, 1H), 4.11 (s, 1H), 4.00 (d, J = 17.3 Hz, 1H), 3.71 (s, 1H), 3.32 (td, J = 12.4, 5.4 Hz, 1H), 3.08 (dd, J = 12.3, 6.4 Hz, 1H), 2.94 (d, J = 15.7 Hz, 1H), 2.58 (dq, J = 13.7, 2.2 Hz, 1H), 2.39 (td, J = 12.4, 6.6 Hz, 1H), 1.84 (dd, J = 12.2, 5.0 Hz, 1H), 1.60 (d, J = 6.8 Hz, 3H), 1.29 ppm (dt, J = 13.4, 2.6 Hz, 1H); ^{13}C NMR (126 MHz, $CDCl_3$): δ 189.0, 169.3, 143.1, 139.8, 137.3, 128.1, 122.3, 121.3, 121.0, 111.5, 110.7, 62.1, 58.6, 57.1, 57.0, 46.7, 31.6, 31.2, 13.2 ppm; HRMS calcd. For $C_{19}H_{21}N_2O^+$ $[M + H]^+$ 293.1648, found 293.1648.

Table S3. ^{1}H and ^{13}C NMR ($CDCl_3$, ppm) comparison for norfluorocurarine with that obtained from the Vanderwal synthesis[9]

Vanderwal (^{1}H, 500 MHz, ppm)	This work (^{1}H, 500 MHz, ppm)	Vanderwal (^{13}C, 125 MHz, ppm)	This work (^{13}C, 126 MHz, ppm)
10.33 (br s, 1H)	10.32 (br s, 1H)	188.9	189.0
9.37 (s, 1H)	9.36 (s, 1H)	169.1	169.3
7.31 (d, *J* = 7.6 Hz, 1H)	7.29 (d, *J* = 7.4 Hz, 1H)	143.0	143.1
7.21 (t, *J* = 7.6 Hz, 1H)	7.20 (t, *J* = 7.4 Hz, 1H)	139.5	139.8
6.99 (t, *J* = 7.6 Hz, 1H)	6.98 (t, *J* = 7.5 Hz, 1H)	137.1	137.3
6.93 (d, *J* = 7.6 Hz, 1H)	6.92 (d, *J* = 7.8 Hz, 1H)	128.1	128.1
5.42 (q, *J* = 6.8 Hz, 1H)	5.40 (q, *J* = 6.7 Hz, 1H)	122.3	122.3
4.13 (s, 1H)	4.11 (s, 1H)	121.2	121.3
4.02 (d, *J* = 15.6 Hz, 1H)	4.00 (d, *J* = 17.3 Hz, 1H)	121.1	121.0
3.72 (s, 1H)	3.71 (s, 1H)	111.4	111.5
3.34 (td, *J* = 12.4, 5.3 Hz, 1H)	3.32 (td, *J* = 12.4, 5.4 Hz, 1H)	110.6	110.7
3.09 (dd, *J* = 12.4, 6.5 Hz, 1H)	3.08 (dd, *J* = 12.4, 6.4 Hz, 1H)	62.0	62.1
2.96 (d, *J* = 15.6 Hz, 1H)	2.94 (d, *J* = 15.7 Hz, 1H)	58.4	58.6
2.59 (ddd, *J* = 13.4, 3.5, 2.3 Hz, 1H)	2.58 (dq, *J* = 13.7, 2.2 Hz, 1H)	56.9	57.1
2.40 (td, *J* = 12.4, 6.5 Hz, 1H)	2.39 (td, *J* = 12.4, 6.6 Hz, 1H)	56.8	57.0
1.85 (dd, *J* = 12.4, 5.3 Hz, 1H)	1.84 (dd, *J* = 12.2, 5.0 Hz, 1H)	46.6	46.7
1.62 (d, *J* = 6.8 Hz, 3H)	1.60 (d, *J* = 6.8 Hz, 3H)	31.5	31.6
1.30 (dt, *J* = 7.4, 2.8 Hz, 1H)	1.29 (dt, *J* = 13.4, 2.6 Hz, 1H))	31.1	31.2
		13.1	13.2

(–)-Lagumicine

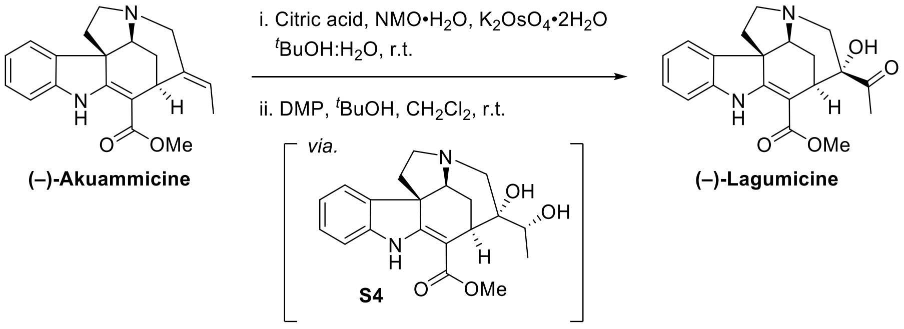

(i) To a solution of (–)-akuammicine (83.6 mg, 0.26 mmol, 1.0 equiv.) in ^{t}BuOH:H_2O (1:1, 6.0 mL) at room temperature was added anhydrous citric acid (100 mg, 0.52 mmol, 2.0 equiv.) and NMO monohydrate (77 mg, 0.57 mmol, 2.2 equiv.) and potassium osmate dihydrate (9.6 mg, 26 μmol, 0.1 equiv.). The resulting mixture was stirred for 14 h, before it was quenched with Na_2SO_3 (10 mL, sat. aq.). The layers were separated and the aqueous layer was extracted with EtOAc (3 × 10 mL), the combined organic layers were dried (Na_2SO_4) and concentrated under reduced pressure to afford compound **S4**, which was used directly in the next step without further purification.

(ii) To a solution of **S4** (crude, obtained above) in CH_2Cl_2 (5.0 mL) at room temperature was added ^{t}BuOH (0.75 mL, 7.90 mmol, 30 equiv.) and Dess-martin periodinane (165 mg, 0.39 mmol, 1.50 equiv.). The resulting mixture was stirred for 5 h, before it was quenched with $Na_2S_2O_3$ (5 mL, 50% sat.) and NaOH (5 mL, 1 M aq.). The layers were separated and the aqueous layer was CH_2Cl_2 (3 × 10 mL), the combined organic layers were dried (Na_2SO_4) and concentrated under reduced pressure. Flash column chromatography (silica gel, CH_2Cl_2:MeOH 20:1→CH_2Cl_2:MeOH:NH_4OH 12:1:1) afforded **(–)-lagumicine** (61.6 mg, 0.17 mmol, 65% over two steps) as a yellow sticky oil. **(–)-Lagumicine:** R_f = 0.30 (silica gel CH_2Cl_2:MeOH:NH_4OH 12:1:1); $[\alpha]_D^{25}$ = –578 (*c* = 0.10, $CHCl_3$); IR (film) ν_{max} 2963, 1707, 1679, 1604, 1464, 1380, 1243, 760 cm^{-1}; ^{1}H NMR (400 MHz, $CDCl_3$): δ 8.95 (br s, 1H), 7.18 (d, *J* = 7.3 Hz, 1H), 7.14 (td, *J* = 7.7, 1.2 Hz, 1H), 6.91 (td, *J* = 7.5, 1.0 Hz, 1H), 6.81 (d, *J* = 7.7 Hz, 1H), 3.95 (br s, 1H), 3.69 (s, 3H), 3.15 (d, *J* = 13.3 Hz, 1H), 3.09 (br s, 1H), 3.06-2.93 (m, 2H), 2.90 (dt, *J* = 13.5, 3.2 Hz, 1H), 2.80-2.73 (m, 1H), 2.61 (d, *J* = 13.2 Hz, 1H), 2.38 (s, 3H), 1.97-1.88 (m, 1H), 1.31-1.19 ppm (m, 1H); ^{13}C NMR (101 MHz, $CDCl_3$): δ 211.9, 170.9, 167.6, 144.3, 134.8, 128.1, 121.5, 119.8, 110.1, 99.0, 60.3, 56.4, 53.3, 51.1, 50.3, 42.6, 35.3, 25.4, 24.9 ppm; HRMS calcd. For $C_{20}H_{23}N_2O_4^+$ $[M + H]^+$ 355.1652, found 355.1648.

Table S4. ^{1}H and ^{13}C NMR ($CDCl_3$, ppm) comparison for lagumicine with that obtained from the Vanderwal synthesis[10]

Vanderwal (^{1}H, 500 MHz, ppm)	This work (^{1}H, 400 MHz, ppm)	Vanderwal (^{13}C, 125 MHz, ppm)	This work (^{13}C, 101 MHz, ppm)
8.97 (br s, 1H)	8.95 (br s, 1H)	211.7	211.9
7.18 (d, *J* = 7.3 Hz, 1H)	7.18 (d, *J* = 7.3 Hz, 1H)	170.9	170.9
7.14 (ddd, *J* = 7.7, 7,7, 1.2 Hz, 1H)	7.14 (td, *J* = 7.7, 1.2 Hz, 1H)	167.6	167.6
6.91 (dd, *J* = 7.4, 7.4 Hz, 1H)	6.91 (td, *J* = 7.5, 1.0 Hz, 1H)	144.3	144.3
6.81 (d, *J* = 7.8 Hz, 1H)	6.81 (d, *J* = 7.7 Hz, 1H)	134.8	134.8
3.95 – 3.87 (m, 1H)	3.95 (br s, 1H)	128.1	128.1
3.69 (s, 3H)	3.69 (s, 3H)	121.4	121.5
3.12 (d, *J* = 13.1 Hz, 1H)	3.15 (d, *J* = 13.3 Hz, 1H)	119.8	119.8
3.08 – 2.92 (m, 3H)	3.09 (br s, 1H)	110.0	110.1
	3.06 – 2.93 (m, 2H)	99.0	99.0
2.83 (ddd, *J* = 13.8, 3.4, 3.4 Hz, 1H)	2.90 (dt, *J* = 13.5, 3.2, 1H)	77.1	76.9
2.78 (dd, *J* = 10.7, 6.1 Hz, 1H)	2.80 – 2.73 (m, 1H)	60.3	60.3
2.62 (d, *J* = 13.0 Hz, 1H)	2.61 (d, *J* = 13.2 Hz, 1H)	56.5	56.4
2.36 (s, 3H)	2.38 (s, 3H)	53.4	53.3
1.95 – 1.88 (m, 1H)	1.97 – 1.88 (m, 1H)	51.1	51.1
1.25 (ddd, *J* = 13.2, 3.1, 3.1 Hz, 1H)	1.31 – 1.19 (m, 1H)	50.4	50.3
		42.6	42.6
		35.3	35.3
		25.4	25.4
		25.0	24.9

(–)-Alstolucines B & F

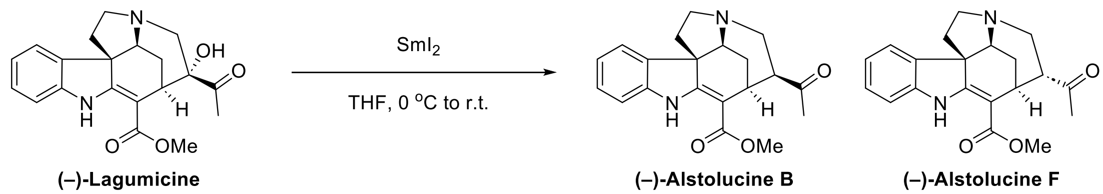

According to a modified literature procedure.[10] To a stirred solution of (–)-lagumicine (25.4 mg, 71.7 μmol, 1.00 equiv) in THF/MeOH (2:1, 1.5 mL) at 0 °C was added SmI_2 (0.1 M solution in THF, 1.8 mL, ~ 0.18 mmol, 2.50 equiv) dropwise. Additional SmI_2 was added at 5 min intervals (x 3) until the starting material was consumed as indicated by TLC. The resulting mixture was quenched with brine (3 mL, 50% sat.). The layers were separated and the aqueous layer was extracted with EtOAc (3 × 5 mL), the combined organic layers were dried (Na_2SO_4) and concentrated under reduced pressure. Flash column chromatography (silica gel, CH_2Cl_2:MeOH 9.8:0.2→CH_2Cl_2:MeOH 9:1) afforded **(–)-alstolucine B** (11.2 mg, 33.1 μmol, 46%) and **(–)-alstolucine F** (6.2 mg, 18.3 μmol, 26%) as a yellow sticky solid. **(–)-Alstolucine B:** R_f = 0.26 (silica gel CH_2Cl_2:MeOH:NH_4OH 12:1:1); $[\alpha]_D^{25}$ = –498 (c = 0.27, $CHCl_3$); IR (film) ν_{max} 3362, 2953, 2929, 2855, 1707, 1605, 1465, 1436, 1278, 758 cm^{-1}; ^1H NMR (400 MHz, $CDCl_3$): δ 8.93 (br s, 1H), 7.18-7.10 (m, 2H), 6.90 (td, J = 7.5, 1.0 Hz, 1H), 6.80 (d, J = 7.7 Hz, 1H), 3.88 (br s, 1H), 3.68 (s, 3H), 3.47 (br s, 1H), 3.10-3.01 (m, 2H), 2.92-2.80 (m, 3H), 2.64 (t, J = 12.3 Hz, 1H), 2.29 (s, 3H), 2.13 (dt, J = 13.0, 3.1 Hz, 1H), 1.91-1.80 (m, 1H), 1.48 ppm (dt, J = 12.9, 3.3 Hz, 1H); ^{13}C NMR (101 MHz, $CDCl_3$): δ 208.6, 172.3, 167.8, 144.4, 135.5, 127.8, 121.3, 119.8, 109.9, 96.7, 60.7, 56.8, 54.1, 51.1, 50.1, 45.8, 43.6, 31.9, 31.0, 29.4 ppm; HRMS calcd. For $C_{20}H_{23}N_2O_3^+$ $[M + H]^+$ 339.1703, found 339.1696.

Table S5. ^{1}H and ^{13}C NMR ($CDCl_3$, ppm) comparison for alstolucine B with that obtained from the Vanderwal synthesis[10]

Vanderwal (^{1}H, 500 MHz, ppm)	This work (^{1}H, 400 MHz, ppm)	Vanderwal (^{13}C, 125 MHz, ppm)	This work (^{13}C, 101 MHz, ppm)
8.93 (br s, 1H)	8.93 (br s, 1H)	208.7	208.6
7.15 (d, *J* = 7.14 Hz, 1H)	7.18 – 7.10 (m, 2H)	172.4	172.3
7.12 (ddd, *J* = 7.6, 7.6, 1.3 Hz, 1H)		167.8	167.8
6.89 (ddd, *J* = 7.5, 7.5, 1.0 Hz, 1H)	6.90 (td, *J* = 7.5, 1.0 Hz, 1H)	144.4	144.4
6.80 (d, *J* = 7.7 Hz, 1H)	6.80 (d, *J* = 7.7 Hz, 1H)	135.6	135.5
3.91 – 3.80 (m, 1H)	3.88 (br s, 1H)	127.7	127.8
3.68 (s, 3H)	3.68 (s, 3H)	121.3	121.3
3.51 – 3.39 (m, 1H)	3.47 (br s, 1H)	119.8	119.8
3.10 – 2.98 (m, 2H)	3.10 – 3.01 (m, 2H)	109.8	109.9
2.92 – 2.80 (m, 2H)	2.92 – 2.80 (m, 3H)	96.6	96.7
2.80 (dd, *J* = 12.1, 4.3, 1H)		60.8	60.7
2.62 (dd, *J* = 12.4, 12.4 Hz, 1H)	2.64 (t, *J* = 12.3 Hz, 1H)	56.9	56.8
2.29 (s, 3H)	2.29 (s, 3H)	54.2	54.1
2.11 (ddd, *J* = 12.9, 3.0, 3.0 Hz, 1H)	2.13 (dt, *J* = 13.0, 3.1 Hz, 1H)	51.0	51.1
1.90 – 1.78 (m, 1H)	1.91 – 1.80 (m, 1H)	50.3	50.1
1.47 (dd, *J* = 13.0, 3.2, 3.2 Hz, 1H)	1.48 (dt, *J* = 12.9, 3.3 Hz, 1H)	45.8	45.8
		43.7	43.6
		32.0	31.9
		31.1	31.0
		29.4	29.4

(–)-Alstolucine F: R_f = 0.39 (silica gel CH_2Cl_2:MeOH:NH_4OH 12:1:1); $[\alpha]_D^{25}$ = –361 (*c* = 0.18, $CHCl_3$); IR (film) ν_{max} 2951, 2926, 2858, 1708, 1680, 1608, 1478, 1241, 762 cm^{-1}; ^{1}H NMR (500 MHz, $CDCl_3$): δ 8.81 (br s, 1H), 7.19 (d, *J* = 7.4 Hz, 1H), 7.15 (td, *J* = 7.7, 1.2 Hz, 1H), 6.91 (td, *J* = 7.5, 0.9 Hz, 1H), 6.83 (d, *J* = 7.7 Hz, 1H), 4.06 (s, 1H), 3.77 (s, 3H), 3.37 (qd, *J* = 3.1, 1.7 Hz, 1H), 3.28 (dd, *J* = 14.0, 10.1 Hz, 1H), 3.17 (dt, *J* = 11.3, 7.4 Hz, 1H), 3.01 (ddd, *J* = 9.7, 5.9, 2.8 Hz, 1H), 2.96 (ddd, *J* = 11.5, 6.7, 5.4 Hz, 1H), 2.81 (dd, *J* = 14.0, 5.9 Hz, 1H), 2.34 (ddd, *J* = 12.8, 8.0, 6.7 Hz, 1H), 2.25 (s, 3H), 2.19 (dt, *J* = 13.7, 3.5 Hz, 1H), 2.02 (ddd, *J* = 12.5, 6.6, 5.4 Hz, 1H), 1.19 ppm (dt, *J* = 13.8, 2.8 Hz, 1H); ^{13}C NMR (126 MHz, $CDCl_3$): δ 210.0, 168.8, 168.0, 144.3, 135.1, 128.1, 121.3, 120.9, 109.9, 102.7, 58.6, 58.4, 53.1, 51.3, 49.5, 47.1, 45.2, 29.5, 27.5, 26.7 ppm; HRMS calcd. For $C_{20}H_{23}N_2O_3^+$ $[M + H]^+$ 339.1703, found 339.1695.

Table S6. ^{1}H and ^{13}C NMR ($CDCl_3$, ppm) comparison for alstolucine F with that obtained from the Vanderwal synthesis[10]

Vanderwal (^{1}H, 500 MHz, ppm)	This work (^{1}H, 500 MHz, ppm)	Vanderwal (^{13}C, 125 MHz, ppm)	This work (^{13}C, 126 MHz, ppm)
8.81 (br s, 1H)	8.81 (br s, 1H)	210.0	210.0
7.18 (d, *J* = 7.4 Hz, 1H)	7.19 (d, *J* = 7.4 Hz, 1H)	168.8	168.8
7.14 (ddd, *J* = 7.7, 7.7, 1.3 Hz, 1H)	7.15 (td, *J* = 7.7, 1.2 Hz, 1H)	168.0	168.0
6.90 (ddd, *J* = 7.4, 7.4, 1.0 Hz, 1H)	6.91 (td, *J* = 7.5, 0.9 Hz, 1H)	144.3	144.3
6.83 (d, *J* = 7.7 Hz, 1H)	6.83 (d, *J* = 7.7 Hz, 1H)	135.1	135.1
4.09 – 4.02 (m, 1H)	4.06 (s, 1H)	128.0	128.1
3.76 (s, 3H)	3.77 (s, 3H)	121.3	121.3
3.40 – 3.34 (m, 1H)	3.37 (qd, *J* = 3.1, 1.7 Hz, 1H)	120.8	120.9
3.28 (dd, *J* = 14.0, 10.1 Hz, 1H)	3.28 (dd, *J* = 14.0, 10.1 Hz, 1H)	109.9	109.9
3.16 (ddd, *J* = 11.2, 8.0, 6.7 Hz, 1H)	3.17 (dt, *J* = 11.3, 7.4 Hz, 1H)	102.7	102.7
3.01 (ddd, *J* = 9.6, 5.9, 2.7 Hz, 1H)	3.01 (ddd, *J* = 9.7, 5.9, 2.8 Hz, 1H)	58.7	58.6
2.96 (ddd, *J* = 11.6, 6.7, 5.4 Hz, 1H)	2.96 (ddd, *J* = 11.5, 6.7, 5.4 Hz, 1H)	58.3	58.4
2.80 (dd, *J* = 14.0, 5.9 Hz, 1H)	2.81 (dd, *J* = 14.0, 5.9 Hz, 1H)	53.1	53.1
2.34 (ddd, *J* = 12.6, 8.1, 6.7 Hz, 1H)	2.34 (ddd, *J* = 12.8, 8.0, 6.7 Hz, 1H)	51.3	51.3
2.25 (s, 3H)	2.25 (s, 3H)	49.5	49.5
2.18 (ddd, *J* = 13.7, 3.4, 3.4 Hz, 1H)	2.19 (dt, *J* = 13.7, 3.5 Hz, 1H)	47.1	47.1
2.00 (ddd, *J* = 12.6, 6.7, 5.4 Hz, 1H)	2.02 (ddd, *J* = 12.5, 6.6, 5.4 Hz, 1H)	45.2	45.2
1.18 (ddd, *J* = 13.4, 2.7, 2.7 Hz, 1H)	1.19 (dt, J = 13.8, 2.8 Hz, 1H)	29.5	29.5
		27.5	27.5
		26.7	26.7

(–)-Echitamidine

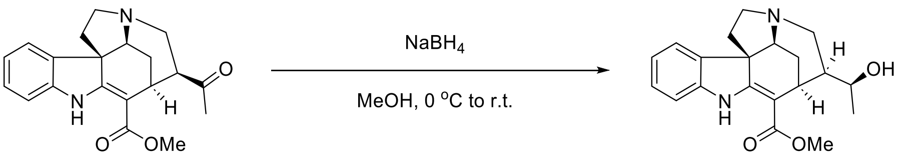

(–)-Alstolucine B **(–)-Echitamidine**

To a solution of (–)-alstolucine B (5.3 mg, 16 μmol, 1.0 equiv.) in MeOH (1.0 mL) at 0 °C was added sodium borohydride (1.2 mg, 32 μmol, 2.0 equiv.). The resulting mixture was stirred for 2 h, before it was quenched with brine (3 mL, 50% sat.). The layers were separated and the aqueous layer was EtOAc (3 × 5 mL), the combined organic layers were dried (Na_2SO_4) and concentrated under reduced pressure. Flash column chromatography (silica gel, CH_2Cl_2:MeOH 9.8:0.2→CH_2Cl_2:MeOH 9:1) afforded **(–)-**

echitamidine (4.5 mg, 13 μmol, 84%) as a white sticky solid. **(–)-Echitamidine:** R_f = 0.26 (silica gel CH_2Cl_2:MeOH:NH_4OH 12:1:1); $[\alpha]_D^{25}$ = –496 (*c* = 0.40, $CHCl_3$); IR (film) ν_{max} 3424, 2951, 2930, 1666, 1596, 1476, 1232, 760 cm^{-1}; ^{1}H NMR (400 MHz, $CDCl_3$): δ 8.64 (br s, 1H), 7.19 (d, *J* = 7.8 Hz, 1H), 7.15 (td, *J* = 7.7, 1.3 Hz, 1H), 6.93 (td, *J* = 7.5, 1.0 Hz, 1H), 6.84 (dt, *J* = 7.8, 0.8 Hz, 1H), 4.44 (br s, 1H), 3.95-3.90 (m, 1H), 3.87 (s, 3H), 3.35-3.31 (m, 1H), 3.26 (dq, *J* = 9.3, 6.2 Hz, 1H), 3.14-3.06 (m, 1H), 2.95-2.80 (m, 3H), 2.04 (dt, *J* = 13.0, 3.1 Hz, 1H), 1.94 (dd, *J* = 12.8, 11.3 Hz, 1H), 1.85 (dd, *J* = 12.4, 6.6 Hz, 1H), 1.81-1.72 (m, 1H), 1.41 (ddd, *J* = 13.1, 4.1, 2.6 Hz, 1H), 1.16 ppm (d, *J* = 6.2 Hz, 3H); ^{13}C NMR (101 MHz, $CDCl_3$): δ 172.5, 169.0, 143.9, 135.7, 127.8, 121.6, 120.0, 109.8, 97.1, 68.6, 61.0, 57.3, 54.2, 52.1, 48.3, 46.0, 43.6, 31.2, 29.0, 20.0 ppm; HRMS calcd. For $C_{20}H_{25}N_2O_3^+$ $[M + H]^+$ 341.1860, found 341.1852.

Table S7. ^{1}H and ^{13}C NMR ($CDCl_3$, ppm) comparison for echitamidine with that obtained from the Vanderwal synthesis[10]

Vanderwal (^{1}H, 500 MHz, ppm)	This work (^{1}H, 400 MHz, ppm)	Vanderwal (^{13}C, 125 MHz, ppm)	This work (^{13}C, 101 MHz, ppm)
8.63 (br s, 1H)	8.64 (br s, 1H)	172.6	172.5
7.18 (d, *J* = 7.3 Hz, 1H)	7.19 (d, *J* = 7.8 Hz, 1H)	169.0	169.0
7.14 (dd, *J* = 7.7, 7,7 Hz, 1H)	7.15 (td, *J* = 7.7, 1.3 Hz, 1H)	143.9	143.9
6.92 (dd, *J* = 7.5, 7.5 Hz, 1H)	6.93 (td, *J* = 7.5, 1.0 Hz, 1H)	135.8	135.7
6.84 (d, *J* = 7.7 Hz, 1H)	6.84 (dt, *J* = 7.8, 0.8 Hz, 1H)	127.8	127.8
4.55 - 4.35 (m, 1H)	4.44 (br s, 1H)	121.6	121.6
3.91 - 3.84 (m, 1H)	3.95 – 3.90 (m, 1H)	120.0	120.0
3.87 (s, 3H)	3.87 (s, 3H)	109.8	109.8
3.36 – 3.29 (m, 1H)	3.35 – 3.31 (m, 1H)	97.0	97.1
3.31 – 3.19 (m, 1H)	3.26 (dq, *J* = 9.3, 6.2 Hz, 1H)	68.6	68.6
3.15 – 3.02 (m, 1H)	3.14 – 3.06 (m, 1H)	61.1	61.0
2.94 – 2.78 (m, 3H)	2.95 – 2.80 (m, 3H)	57.3	57.3
2.03 (br d, *J* = 13.2 Hz, 1H)	2.04 (dt, *J* = 13.0, 3.1 Hz, 1H)	54.3	54.2
1.93 (dd, *J* = 12.1, 12.1 Hz, 1H)	1.94 (dd, *J* = 12.8, 11.3 Hz, 1H)	52.1	52.1
1.84 (dd, *J* = 12.9, 6.6 Hz, 1H)	1.85 (dd, *J* = 12.4, 6.6 Hz, 1H)	48.3	48.3
1.79 – 1.70 (m, 1H)	1.81 – 1.72 (m, 1H)	46.0	46.0
1.40 (br d, *J* = 13.2 Hz, 1H)	1.41 (ddd, *J* = 13.1, 4.1, 2.6 Hz, 1H)	43.7	43.6
1.15 (br d, *J* = 6.1 Hz, 3H)	1.16 (br d, *J* = 6.2 Hz, 3H)	31.2	31.2
		29.0	29.0
		19.9	20.0

(*Z*)-4-Bromo-3-iodobut-2-en-1-yl benzoate, 22

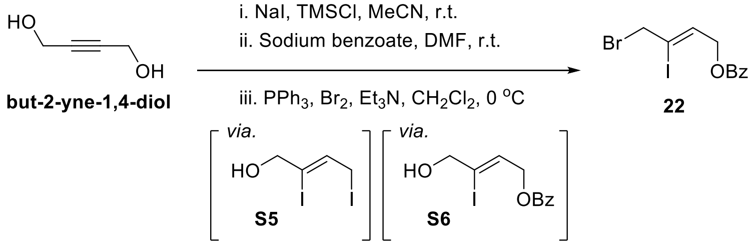

According to a modified literature procedure.[11] (i) To a stirred solution of sodium iodide (40.0 g, 267 mmol, 2.3 equiv.) in MeCN (100 mL) at room temperature was added TMSCl (33.8 mL, 266 mmol, 2.3 equiv.). The resulting mixture was stirred for 10 min before a solution of but-2-yne-1,4-diol (10.0 g, 116 mmol, 1.0 equiv.) in MeCN (40.0 mL) was added. The resulting mixture was stirred for 30 min before it was quenched with $Na_2S_2O_3$ (175 mL, 10% aq.) and diluted with Et_2O (100 mL). The layers were separated and the aqueous layer was extracted with Et_2O (3 × 100 mL), the combined organic layers were washed with $NaHCO_3$ (200 mL, sat. aq.), brine (200 mL), dried (Na_2SO_4) and concentrated under reduced pressure. The resulting compound **S5** was used directly in the next step without further purification.

(ii) To a stirred solution of **S5** (crude, obtained above) in DMF (100 mL) at room temperature was added sodium benzoate (50.1 g, 348 mol, 3.0 equiv.). The resulting mixture was stirred for 18 h before it was quenched with citric acid (100 mL, 10% aq.). The layers were separated and the aqueous layer was extracted with Et_2O (3 × 100 mL), the combined organic layers were washed with brine (100 mL), dried (Na_2SO_4) and concentrated under reduced pressure. The resulting residue was passed through a thin layer of silica (silica gel, hexanes:EtOAc 12:1, sintered glass filtration) to afford compound **S6** (30.7 g, 96.5 mmol 83% over two steps) as a pale yellow oil.

(iii) To a stirred solution of PPh_3 (13.6 g, 51.9 mmol, 1.1 equiv.) in CH_2Cl_2 (50 mL) at 0 °C was added Br_2 (2.66 mL, 51.9 mol, 1.1 equiv.) followed by Et_3N (9.87 mL, 70.8 mol, 1.5 equiv.). The resulting mixture was stirred for 10 min before a solution of **S6** (obtained above, 15.0 g, 47.2 mmol, 1.0 equiv.) in CH_2Cl_2 (20 mL) was added. The resulting mixture was stirred for 10 min before it was concentrated under reduced pressure. The resulting residue was purified by column chromatography (silica gel, pentane:EtOAc 1:0→15:1) to afford compound **22** (14.4 g, 37.8 mmol, 80%) as a fine white solid. **22:** R_f = 0.54 (silica gel, pentane:EtOAc 18:1); IR (film) ν_{max} 3025, 1705, 1424, 1212, 1182, 1127, 710 cm^{-1}; ^{1}H NMR (400 MHz, $CDCl_3$): δ 8.06 (d, *J* = 8.4 Hz, 2H), 7.58 (t, *J* = 7.4 Hz, 1H), 7.45 (t, *J* = 7.7 Hz, 2H), 6.41 (t, *J* = 5.7 Hz, 1H), 4.89 (d, *J* = 5.7 Hz, 2H), 4.36 ppm (s, 2H); ^{13}C NMR (101 MHz, $CDCl_3$): δ

166.3, 134.9, 133.4, 129.9, 129.7, 128.6, 103.3, 68.7, 41.9 ppm; HRMS calcd. For $C_{11}H_{11}BrIO_2^+$ [M + H]$^+$ 380.8982, found 380.8981.

Intramolecular route to Strychnine:

In the intramolecular context, indole **S7** underwent alkylation with sidechain **22** (96%), followed by the cycloaddition cascade with TDO **9a**. A single diastereomer of the diene cycloadduct **S9** was observed, which following *in situ* reduction gave the tetracyclic product **23**/**23'** in 50% overall yield (1:1 *dr*).

(*Z*)-3-Iodo-4-((2-(1-(4-methoxybenzyl)-1*H*-indol-3-yl)ethyl)amino)but-2-en-1-yl benzoate, S8

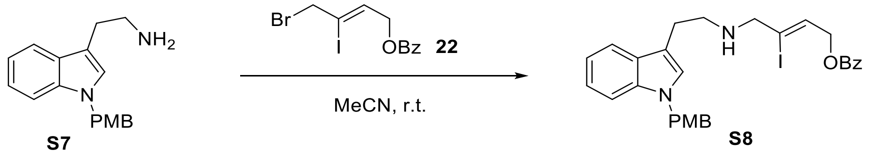

To a stirred solution of PMB-tryptamine **S7**[12] (1.35 g, 4.82 mmol, 3.0 equiv.) in MeCN (16.0 mL) at room temperature was added **22** (610 mg, 1.60 mmol, 1.0 equiv.). The mixture was stirred for 18 h before it was concentrated under reduced pressure. Flash column chromatography (silica gel, pentane:EtOAc 4:1→1:2) afforded compound **S8** (0.89 g, 1.53 mmol, 96%) as a brown syrup. **S8**: R_f = 0.64 (silica gel, EtOAc); IR (film) ν_{max} 3417, 2908, 2835, 1720, 1514, 1482, 1271, 1112, 742 cm^{-1}; ^{1}H NMR (400 MHz, $CDCl_3$): δ 8.05 (d, J = 7.1 Hz, 2H), 7.62 (d, J = 7.8 Hz, 1H), 7.57 (t, J = 7.4 Hz, 1H), 7.44 (t, J = 7.7 Hz, 2H), 7.28 (d, J = 8.2 Hz, 1H), 7.20-7.14 (m, 1H), 7.12-7.05 (m, 3H), 6.98 (s, 1H), 6.82 (d, J = 8.7 Hz, 2H), 6.17 (t, J = 5.8 Hz, 1H), 5.20 (s, 2H), 4.88 (d, J = 5.8 Hz, 2H), 3.76 (s, 3H), 3.54 (s, 2H), 2.98 (t, J = 6.8 Hz, 2H), 2.87 ppm (t, J = 7.0 Hz, 2H); ^{13}C NMR (101 MHz, $CDCl_3$): δ 166.4, 159.2, 136.8, 133.2, 130.6, 130.0, 129.8, 129.8, 128.5, 128.4, 128.3, 126.1, 121.9, 119.2, 119.1, 114.2, 113.0, 113.0, 109.8, 68.7, 61.1, 55.4, 49.5, 48.0, 25.9 ppm; HRMS calcd. For $C_{29}H_{30}IN_2O_3^+$ [M + H]$^+$ 581.1296, found 581.1288.

(*Z*)-4-((3a*S*,6a*R*,11b*R*)-6-((3a*S*,6*R*,7a*R*)-8,8-Dimethyl-2,2-dioxidohexahydro-3*H*-3a,6-methanobenzo[*c*]isothiazole-1-carbonyl)-7-(4-methoxybenzyl)-1,2,3a,4,6a,7-hexahydro-3*H*-pyrrolo[2,3-*d*]carbazol-3-yl)-3-iodobut-2-en-1-yl benzoate, 23 and 23'

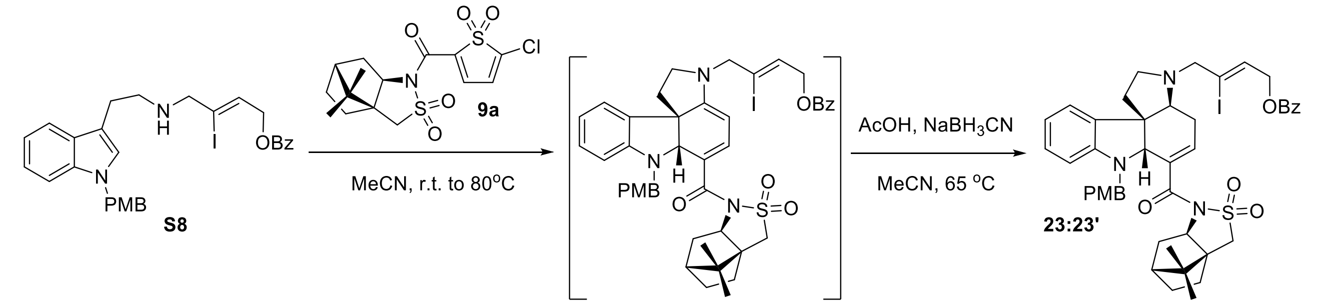

To a stirred solution of **S8** (222 mg, 0.38 mmol, 1.0 equiv.) in MeCN (20.0 mL) at room temperature was added **9a** (150 mg, 0.38 mmol, 1.0 equiv.). The resulting mixture was stirred for 19 h before additional **S8** (267 mg, 0.46 mmol, 1.2 equiv.) was added. The resulting mixture was warmed to 80 °C and stirred for 46 h before it was cooled to 65 °C, and AcOH (0.33 mL, 5.77 mmol, 15.2 equiv.) was added. The resulting mixture was stirred for 15 min, then a solution of $NaBH_3CN$ (241 mg, 3.84 mmol, 10.1 equiv.) in MeOH (3.8 mL) was added. The resulting mixture was stirred for 30 min before it was cooled to room temperature and quenched by slow addition of $NaHCO_3$ (20 mL, sat. aq.), diluted with water (10 mL), and CH_2Cl_2 (50 mL). The layers were separated and the aqueous layer was extracted with CH_2Cl_2 (3 × 30 mL), the combined organic layers were washed with water (50 mL), brine (50 mL), dried (Na_2SO_4) and concentrated under reduced pressure. Flash column chromatography (silica gel, pentane:Et_2O 10:1→2:1) afforded compound **23** and its C3a-epimer **23'** (169 mg combined mass, 0.19 mmol, 50%, 1:1 *dr*) as a brown foam. These diastereomers could be separated for the purposes of characterisation, and for the subsequent reduction of **23**.

23 (desired): R_f = 0.48 (silica gel, pentane:Et_2O:CH_2Cl_2 4:1:1); $[\alpha]_D^{25}$ = –58.1 (*c* = 0.74, $CHCl_3$); IR (film) ν_{max} 3008, 2958, 1721, 1671, 1512, 1483, 1270, 736 cm^{-1}; ^1H NMR (500 MHz, $CDCl_3$): δ 8.05 (d, *J* = 8.3 Hz, 2H), 7.57 (t, *J* = 7.4 Hz, 1H), 7.49-7.41 (m, 3H), 7.19 (d, *J* = 8.7 Hz, 2H), 7.05 (d, *J* = 7.3 Hz, 1H), 6.95 (t, *J* = 8.2 Hz, 1H), 6.80 (d, *J* = 8.9 Hz, 2H), 6.68 (t, *J* = 6.9 Hz, 1H), 6.32 (d, *J* = 7.5 Hz, 1H), 6.18 (t, *J* = 5.9 Hz, 1H), 4.89 (d, *J* = 6.1 Hz, 2H), 4.52 (s, 1H), 4.40 (d, *J* = 15.1 Hz, 1H), 4.13 (dd, *J* = 7.6, 4.5 Hz, 1H), 3.94 (d, *J* = 15.3 Hz, 1H), 3.76 (s, 3H), 3.52 (d, *J* = 14.2 Hz, 1H), 3.47 (d, *J* = 13.6 Hz, 1H), 3.39 (d, *J* = 13.7 Hz, 1H), 3.11-3.03 (m, 2H), 2.80 (s, 1H), 2.67 (dt, *J* = 15.5, 3.7 Hz, 1H), 2.60 (dd, *J* = 15.4, 7.8 Hz, 1H), 2.45-2.36 (m, 1H), 2.25 (dd, *J* = 13.0, 7.0, 1.7 Hz, 1H), 2.00 (dd, *J* = 13.6, 7.8

Hz, 1H), 1.98-1.80 (m, 5H), 1.48-1.41 (m, 1H), 1.38-1.31 (m, 1H), 1.23 (s, 3H), 0.97 ppm (s, 3H); ^{13}C NMR (126 MHz, $CDCl_3$): δ 170.0, 166.4, 158.5, 152.2, 149.8, 136.1, 133.3, 132.6, 131.0, 130.5, 130.0, 129.9, 129.4, 128.6, 127.9, 123.0, 118.5, 113.8, 111.8, 108.7, 72.8, 68.7, 68.3, 65.9, 64.9, 55.3, 54.8, 53.8, 51.3, 48.2, 47.9, 45.1, 38.5, 38.2, 33.1, 26.9, 26.8, 21.3, 20.0 ppm; HRMS calcd. For $C_{44}H_{49}IN_3O_6S^+$ $[M + H]^+$ 874.2381, found 874.2365.

23' (undesired): R_f = 0.26 (silica gel, pentane:Et_2O:CH_2Cl_2 4:1:1); $[\alpha]_D^{25}$ = –101 (*c* = 0.50, $CHCl_3$); IR (film) ν_{max} 2936, 1722, 1668, 1511, 1482, 1271, 739 cm^{-1}; ^{1}H NMR (400 MHz, $CDCl_3$): δ 8.06 (d, *J* = 7.1 Hz, 2H), 7.69 (d, *J* = 7.3 Hz, 1H), 7.57 (t, *J* = 7.4 Hz, 1H), 7.45 (t, *J* = 7.6 Hz, 2H), 7.18 (d, *J* = 8.7 Hz, 2H), 7.01 (td, *J* = 7.7, 1.3 Hz, 1H), 6.79 (d, *J* = 8.7 Hz, 2H), 6.63 (t, *J* = 7.8 Hz, 1H), 6.52 (s, 1H), 6.37 (d, *J* = 7.8 Hz, 1H), 6.30 (t, *J* = 5.7 Hz, 1H), 4.96 (d, *J* = 5.7 Hz, 2H), 4.56 (s, 1H), 4.44 (d, *J* = 15.5 Hz, 1H), 4.18 (d, *J* = 15.5 Hz, 1H), 4.02 (t, *J* = 6.1 Hz, 1H), 3.76 (s, 3H), 3.70 (d, *J* = 12.7 Hz, 1H), 3.42 (d, *J* = 13.7 Hz, 1H), 3.37 (d, *J* = 13.7 Hz, 1H), 3.24 (q, *J* = 8.3 Hz, 1H), 3.18 (d, *J* = 14.0 Hz, 1H), 2.88-2.80 (m, 1H), 2.42-2.29 (m, 3H), 1.99-1.76 (m, 6H), 1.72-1.63 (m, 1H), 1.45 (t, *J* = 10.6 Hz, 1H), 1.38-1.30 (m, 1H), 1.17 (s, 3H), 0.96 ppm (s, 3H); ^{13}C NMR (101 MHz, $CDCl_3$): δ 170.5, 166.5, 158.7, 149.2, 138.6, 135.8, 133.3, 132.8, 131.4, 130.8, 130.1, 129.9, 128.9, 128.6, 128.0, 125.9, 117.7, 113.8, 111.7, 108.9, 68.8, 67.9, 66.6, 65.8, 64.6, 55.4, 54.8, 53.5, 50.6, 50.3, 48.3, 47.9, 45.2, 38.5, 37.0, 33.3, 27.9, 26.6, 21.2, 20.0 ppm; HRMS calcd. For $C_{44}H_{49}IN_3O_6S^+$ $[M + H]^+$ 874.2381, found 874.2371.

(*Z*)-4-((3a*S*,6a*R*,11b*S*)-6-((3a*S*,6*R*,7a*R*)-8,8-Dimethyl-2,2-dioxidohexahydro-3*H*-3a,6-methanobenzo[*c*]isothiazole-1-carbonyl)-1,2,3a,4,6a,7-hexahydro-3*H*-pyrrolo[2,3-*d*]carbazol-3-yl)-3-iodobut-2-en-1-yl benzoate, S9

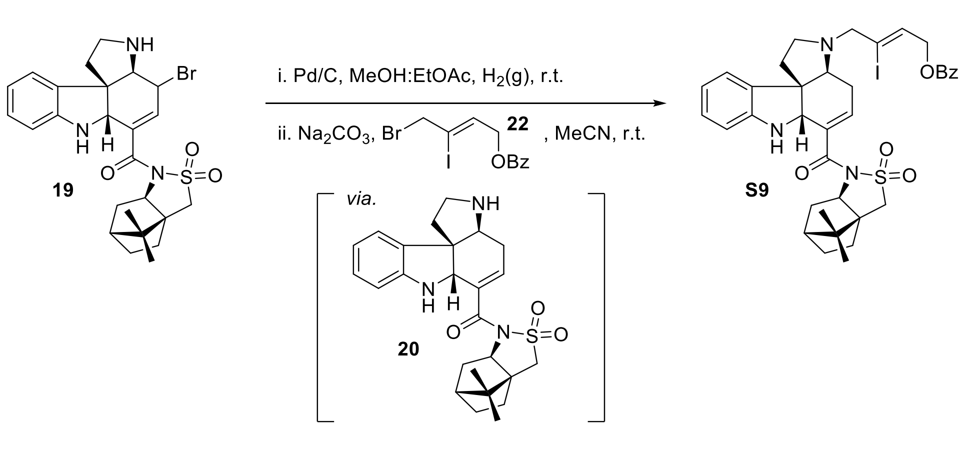

(i) To a solution of **19** (51.0 mg, 95.8 μmol, 1.0 equiv.) in MeOH:EtOAc (9:1, 1.0 mL) at room

temperature was added Pd/C (10 wt% on activated carbon, 10.2 mg, 9.58 μmol, 0.1 equiv.). The resulting mixture was purged with 3 cycles of H_2/vacuum and stirred under an H_2 atmosphere (balloon) for a further 5 h, before it was filtered through a pad of Celite®, eluted with EtOAc (10 mL). The filtrate was concentrated under reduced pressure to afford compound **20** as yellow foam, which was used directly in the next step without further purification.

(ii) To a solution of **20** (crude, obtained above) in MeCN (2.0 mL) at room temperature were added sodium carbonate (25.4 mg, 0.24 mmol, 2.4 equiv.) and **22** (38.1 mg, 0.1 mmol, 1.0 equiv.). The resulting mixture was stirred for 48 h before it was concentrated to half volume using a stream of nitrogen (if a rotary evaporator is used the bath temperature must be kept at or below 20 °C to avoid alkylation of the indoline nitrogen with residual bromide **22**). This solution was directly purified by flash column chromatography (silica gel, pentane:EtOAc 9:1→7:3) which afforded compound **S9** (49.7 mg, 65.9 μmol, 69% over two steps) as a light yellow foam. **S9**: R_f = 0.39 (silica gel, pentane:EtOAc 7:3); $[\alpha]_D^{25}$ = –65.0 (c = 0.3, $CHCl_3$); IR (film) ν_{max} 3369, 2987, 2362, 1720, 1676, 1273, 750 cm^{-1}; ^1H NMR (500 MHz, $CDCl_3$): δ 8.06 (d, J = 8.3 Hz, 2H), 7.57 (t, J = 7.4 Hz, 1H), 7.48-7.42 (m, 2H), 7.07 (dd, J = 7.4, 1.2 Hz, 1H), 7.01 (td, J = 7.6, 1.3 Hz, 1H), 6.78 (dd, J = 5.9, 3.5 Hz, 1H), 6.71 (td, J = 7.4, 1.0 Hz, 1H), 6.57 (d, J = 7.7 Hz, 1H), 6.26 (t, J = 5.8 Hz, 1H), 4.91 (d, J = 5.8 Hz, 2H), 4.53 (s, 1H), 4.11 (dd, J = 7.7, 4.3 Hz, 1H), 4.03 (br s, 1H), 3.60 (dq, J = 14.9, 1.7 Hz, 1H), 3.46 (d, J = 13.6 Hz, 1H), 3.37 (d, J = 13.7 Hz, 1H), 3.30 (d, J = 14.9 Hz, 1H), 3.20 (ddd, J = 9.5, 8.0, 5.4 Hz, 1H), 3.08 (t, J = 3.6 Hz, 1H), 2.62 (td, J = 9.2, 6.3 Hz, 1H), 2.45-2.33 (m, 2H), 2.16 (ddd, J = 12.7, 8.0, 6.2 Hz, 1H), 2.08 (ddd, J = 12.7, 8.9, 5.4 Hz, 1H), 1.99 (dd, J = 13.4, 7.7 Hz, 1H), 1.94-1.84 (m, 4H), 1.46-1.40 (m, 1H), 1.39-1.31 (m, 1H), 1.20 (s, 3H), 0.97 ppm (s, 3H); ^{13}C NMR (126 MHz, $CDCl_3$): δ 170.7, 166.4, 150.7, 141.0, 134.1, 133.2, 130.2, 130.1, 129.9, 128.5, 128.2, 123.1, 118.9, 111.1, 109.9, 68.8, 66.0, 65.5, 65.5, 62.3, 54.3, 53.8, 51.8, 48.1, 47.9, 45.3, 38.4, 38.2, 33.2, 26.7, 25.7, 21.3, 20.0 ppm; HRMS calcd. For $C_{36}H_{40}IN_3O_5SNa^+$ $[M + Na]^+$ 776.1603, found 776.1626.

(*Z*)-4-((3a*S*,6a*R*,11b*R*)-6-((3a*S*,6*R*,7a*R*)-8,8-Dimethyl-2,2-dioxidohexahydro-3*H*-3a,6-methanobenzo[*c*]isothiazole-1-carbonyl)-7-(4-methoxybenzyl)-1,2,3a,4,6a,7-hexahydro-3*H*-pyrrolo[2,3-*d*]carbazol-3-yl)-3-iodobut-2-en-1-yl benzoate, 23

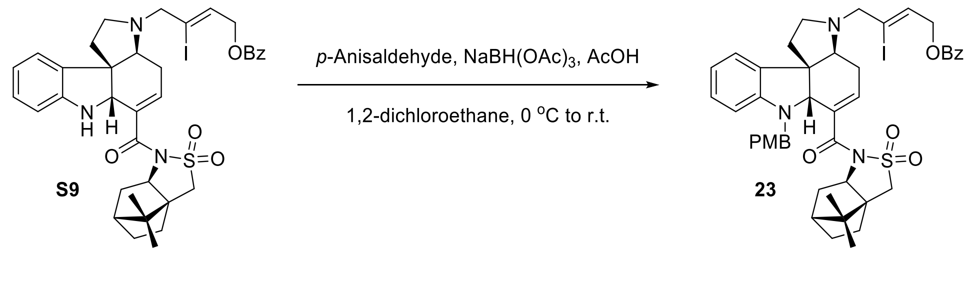

To a solution of **S9** (28.4 mg, 37.7 μmol, 1.0 equiv.) in 1,2-dichloroethane (1.0 mL) at 0 °C were added *p*-anisaldehyde (18 μL, 0.15 mmol, 4.0 equiv.) and sodium triacetoxyborohydride (64 mg, 0.30 mmol, 8.0 equiv.), followed by acetic acid (17.3 μL, 0.30 mmol, 8.0 equiv.). The resulting mixture was warmed to room temperature and stirred for 2 d before it was diluted with Na_2CO_3 (sat. aq. 2 mL). The layers were separated and the aqueous layer was extracted with CH_2Cl_2 (3 × 3 mL), the combined organic layers were dried (Na_2SO_4) and concentrated under reduced pressure. Flash column chromatography (silica gel, pentane:Et_2O 95:5→7:3) afforded compound **23** (26.7 mg, 30.6 μmol, 81%) as a white foam. All physical data of **23** are identical to those obtained from the intramolecular cycloaddition route (see page S31).

(*Z*)-4-((3a*S*,6a*R*,11b*R*)-6-(hydroxymethyl)-7-(4-methoxybenzyl)-1,2,3a,4,6a,7-hexahydro-3*H*-pyrrolo[2,3-*d*]carbazol-3-yl)-3-iodobut-2-en-1-ol, 24

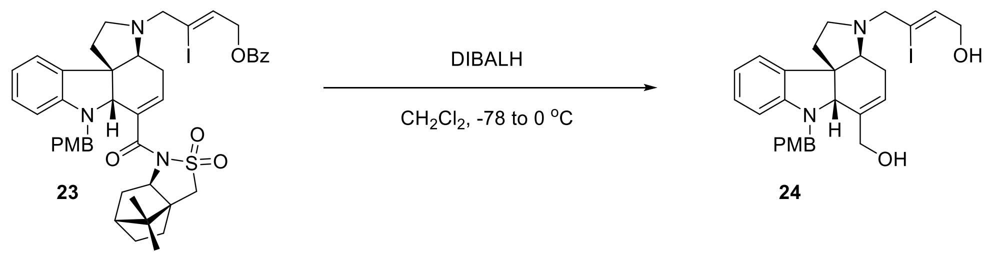

To a stirred solution of **23** (71.2 mg, 81.5 μmol, 1.0 equiv.) in CH_2Cl_2 (2.0 mL) at-78 °C was added DIBALH (1.0 M solution in hexane, 0.33 mL, 0.33 mmol, 4.0 equiv.). The resulting mixture was stirred for 30 min before it was warmed to 0 °C and stirred for additional 2 h. The resulting mixture again cooled to -78 °C and additional DIBALH (1.0 M solution hexane, 0.16 mL, 0.16 mmol, 2.0 equiv) was added.

The resulting mixture was warmed to 0 °C and stirred for 1 h before it was quenched with Rochelle's salt (5 mL, sat. aq.), then EtOAc was added (1 mL) and the mixture was stirred vigorously for 1 h. The layers were separated and the aqueous layer was extracted with CH_2Cl_2 (3 × 8 mL), the combined organic layers were washed with water (15 mL), brine (15 mL), dried (Na_2SO_4) and concentrated under reduced pressure. Flash column chromatography (silica gel, pentane:EtOAc 4:1→0:1) afforded compound **24** (34.6 mg, 62.0 μmol, 76%) as a yellow oil. **24:** R_f = 0.70 (silica gel, EtOAc); $[\alpha]_D^{25}$ = –22.2 (*c* = 0.33, $CHCl_3$); IR (film) ν_{max} 3120, 1723, 1708, 1605, 1485, 1273, 1218, 760 cm^{-1}; ^1H NMR (400 MHz, $CDCl_3$): δ 7.16 (d, *J* = 8.7 Hz, 2H), 7.08 (d, *J* = 8.2 Hz, 1H), 7.01 (t, *J* = 8.3 Hz, 1H), 6.82 (d, *J* = 8.7 Hz, 2H), 6.71 (t, *J* = 7.8 Hz, 1H), 6.35 (d, *J* = 7.9 Hz, 1H), 6.20 (t, *J* = 5.2 Hz, 1H), 6.06-5.97 (m, 1H), 4.38 (d, *J* = 15.8 Hz, 1H), 4.24 (d, *J* = 5.4 Hz, 2H), 4.17-4.08 (m, 2H), 4.04 (d, *J* = 12.5 Hz, 1H), 3.92 (s, 1H), 3.78 (s, 3H), 3.53 (d, *J* = 14.8 Hz, 1H), 3.25-3.07 (m, 2H), 2.75 (s, 1H), 2.56-2.43 (m, 2H), 2.40-2.18 (m, 4H), 1.77 ppm (q, *J* = 10.3, 9.2 Hz, 1H); ^{13}C NMR (101 MHz, $CDCl_3$): δ 158.7, 152.4, 137.9, 136.4, 135.6, 131.0, 128.7, 128.5, 128.0, 123.0, 118.4, 114.0, 107.9, 106.9, 71.8, 69.8, 67.0, 65.6, 55.4, 54.7, 53.9, 52.1, 39.1, 25.5 ppm; HRMS calcd. For $C_{27}H_{32}IN_2O_3^+$ $[M + H]^+$ 559.1452, found 559.1443.

PMB-protected Wieland-Gumlich aldehyde, S10

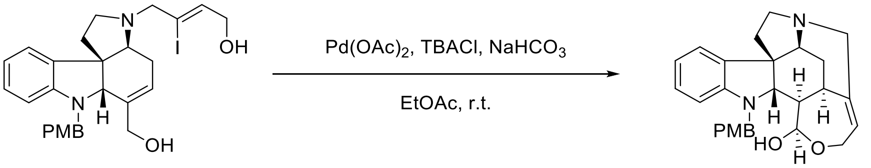

24 **S10**

According to a modified literature procedure.[8] To a stirred solution of **24** (22.7 mg, 40.6 μmol, 1.0 equiv.) in EtOAc (3.0 mL) at room temperature was added tetrabutylammonium chloride (33.9 mg, 0.12 mmol, 3.0 equiv.), $NaHCO_3$ (34.2 mg, 0.41 mmol, 10.1 equiv.) and $Pd(OAc)_2$ (2.7 mg, 12.0 μmol, 0.3 equiv.). The resulting mixture was stirred for 20 h before it was concentrated under reduced pressure. Flash column chromatography (silica gel, CH_2Cl_2:MeOH:NH_4OH 20:1:1) afforded compound **S10** (9.9 mg, 23.0 μmol, 57%) as a yellow sticky solid. **S10:** R_f = 0.26 (silica gel, pentane:EtOAc 1:1); $[\alpha]_D^{25}$ = –21.7 (*c* = 0.49, $CHCl_3$); IR (film) ν_{max} 2951, 2869, 1606, 1512, 1484, 1249, 912, 736 cm^{-1}; ^1H NMR (400 MHz, $CDCl_3$, major diastereoisomer): δ 7.17 (d, *J* = 8.6 Hz, 2H), 7.06 (d, *J* = 7.7 Hz, 1H), 6.92 (d, *J* = 7.3 Hz, 1H), 6.81 (d, *J* = 8.7 Hz, 2H), 6.69 (t, *J* = 7.8 Hz, 1H), 6.50 (d, *J* = 7.8 Hz, 1H), 5.66 (s, 1H), 5.38

(s, 1H), 4.79 (d, J = 16.6 Hz, 1H), 4.64 (d, J = 15.4 Hz, 1H), 4.25 (d, J = 15.4 Hz, 1H), 3.85 (s, 1H), 3.76 (s, 4H), 3.64 (d, J = 14.5 Hz, 1H), 3.42 (d, J = 10.6 Hz, 1H), 3.30 (s, 1H), 3.01 (t, J = 9.3 Hz, 1H), 2.66-2.51 (m, 2H), 2.19 (dd, J = 14.2, 4.0 Hz, 1H), 1.68 (d, J = 10.6 Hz, 1H), 1.51-1.33 (m, 3H), 1.22 (dd, J = 12.5, 6.1 Hz, 1H), 1.06-0.99 ppm (m, 1H); ^{13}C NMR (126 MHz, $CDCl_3$, mixture of diastereomers): δ 159.6, 159.0, 150.8, 150.4, 141.9, 140.8, 137.7, 133.5, 131.4, 130.2, 129.1, 128.5, 128.3, 127.7, 126.0, 125.4, 123.9, 122.4, 121.9, 118.7, 118.5, 114.0, 113.9, 109.8, 106.3, 96.4, 66.7, 65.6, 62.8, 60.3, 60.2, 59.2, 59.0, 57.3, 55.4, 55.4, 54.7, 54.6, 54.6, 53.9, 53.6, 51.1, 50.6, 46.8, 46.3, 41.9, 41.2, 33.3, 28.6, 26.5, 26.0, 24.3 ppm; HRMS calcd. For $C_{27}H_{31}N_2O_3^+$ $[M + H]^+$ 431.2329, found 431.2326.

(–)-Strychnine

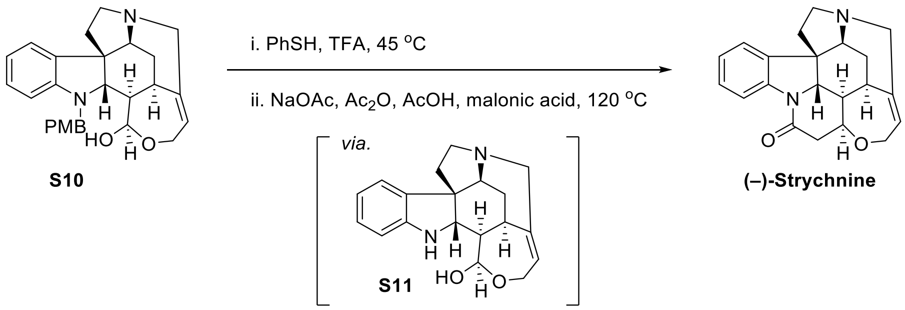

According to a modified literature procedure.[8] (i) To a stirred solution of **S10** (8.5 mg, 19.7 µmol, 1.0 equiv.) in TFA (1.0 mL) at room temperature was added thiophenol (20 µL, 0.19 mmol, 9.6 equiv.). The resulting mixture was warmed to 45 °C and stirred for 18 h before it was concentrated under reduced pressure. The residue was diluted with CH_2Cl_2 (2 mL) and the solution was stirred vigorously with 1 N HCl (2 mL) for 10 min. The aqueous layer was washed with CH_2Cl_2 (3×5 mL) and then basified with ammonium hydroxide solution (0.5 mL, 35 wt%). The resulting solution was extracted with CH_2Cl_2 (5×5 mL), the combined organic layers were dried (Na_2SO_4) and concentrated under reduced pressure. The resulting compound (containing the Wieland-Gumlich aldehyde **S11**) was used directly in the next step without further purification.

(ii) To a stirred solution of **S11** (4.4 mg, 14.2 µmol, 1.0 equiv.) in AcOH (1.0 mL) at room temperature were added sodium acetate (48.0 mg, 0.59 mmol, 41.5 equiv.), acetic anhydride (16 µL, 0.17 mmol, 12.0 equiv.) and malonic acid (48.0 mg, 0.46 mmol, 32.4 equiv.). The resulting mixture was warmed to 120 °C and stirred for 3 h. The resulting residue was cooled to room temperature and diluted with water (3 mL)

and basified with NaOH (1M solution, 1 mL). The resulting mixture was extracted with CH_2Cl_2 (3 × 5 mL), the combined organic layers were dried (Na_2SO_4) and concentrated under reduced pressure. Flash column chromatography (silica gel, CH_2Cl_2:MeOH:NH_4OH 20:1:1) afforded **(–)-strychnine** (3.3 mg, 9.9 µmol, 50% over two steps) as a clear waxy solid. **(–)-Strychnine:** R_f = 0.26 (silica gel, CH_2Cl_2:MeOH:NH_4OH 16:1:0.5); $[\alpha]_D^{25} = -112$ (c = 0.07, $CHCl_3$); IR (film) ν_{max} 2933, 2849, 1668, 1600, 1488, 1397, 1249, 1114, 1038, 753, 742 cm^{-1}; ^1H NMR (500 MHz, $CDCl_3$): δ 8.09 (d, J = 8.0 Hz, 1H), 7.25 (t, J = 7.7 Hz, 1H), 7.16 (d, J = 7.5 Hz, 1H), 7.10 (t, J = 7.9 Hz, 1H), 5.90 (t, J = 5.6 Hz, 1H), 4.28 (dt, J = 8.4, 3.3 Hz, 1H), 4.15 (dd, J = 13.8, 7.0 Hz, 1H), 4.06 (dd, J = 13.5, 6.2 Hz, 1H), 3.94 (s, 1H), 3.86 (d, J = 10.5 Hz, 1H), 3.71 (d, J = 14.8 Hz, 1H), 3.23-3.09 (m, 3H), 2.87 (q, J = 10.0 Hz, 1H), 2.73 (d, J = 14.8 Hz, 1H), 2.67 (dd, J = 17.4, 3.3 Hz, 1H), 2.36 (dt, J = 14.4, 4.3 Hz, 1H), 1.92-1.87 (m, 2H), 1.46 (d, J = 14.4 Hz, 1H), 1.31-1.26 ppm (m, 1H); ^{13}C NMR (151 MHz, $CDCl_3$): δ 169.5, 142.4, 140.8, 132.9, 128.7, 127.4, 124.4, 122.4, 116.4, 77.8, 64.8, 60.4, 60.3, 52.8, 52.1, 50.5, 48.4, 43.0, 42.7, 31.8, 27.0 ppm; HRMS calcd. For $C_{21}H_{23}N_2O_2^+$ $[M + H]^+$ 335.1754, found 335.1752.

Table S8. ^{1}H and ^{13}C NMR ($CDCl_3$, ppm) comparison for strychnine with that obtained from the MacMillan synthesis[8]

Macmillan (^{1}H, 500 MHz, ppm)	This work (^{1}H, 500 MHz, ppm)	Macmillan (^{13}C, 125 MHz, ppm)	This work (^{13}C, 151 MHz, ppm)
8.10 (d, *J* = 8.0 Hz, 1H)	8.09 (d, *J* = 8.0 Hz, 1H)	169.3	169.5
7.26 (t, *J* = 7.2 Hz, 1H)	7.25 (t, *J* = 7.7 Hz, 1H)	142.2	142.4
7.18 (d, *J* = 7.3 Hz, 1H)	7.16 (d, *J* = 7.5 Hz, 1H)	140.4	140.8
7.11 (t, *J* = 7.2 Hz, 1H)	7.10 (t, *J* = 7.9 Hz, 1H)	132.6	132.9
5.95 – 5.89 (m, 1H)	5.90 (t, *J* = 5.6 Hz, 1H)	128.7	128.7
4.30 (ddd, *J* = 8.2, 3.2, 3.2 Hz, 1H)	4.28 (dt, *J* = 8.4, 3.3 Hz, 1H)	127.6	127.4
4.16 (dd, *J* = 13.8, 7.0 Hz, 1H)	4.15 (dd, *J* = 13.8, 7.0 Hz, 1H)	124.3	124.4
4.07 (dd, *J* = 13.7, 6.0 Hz, 1H)	4.06 (dd, *J* = 13.5, 6.2 Hz, 1H)	122.3	122.4
4.01 – 3.94 (m, 1H)	3.94 (s, 1H)	116.3	116.4
3.87 (d, *J* = 10.5 Hz, 1H)	3.86 (d, *J* = 10.5 Hz, 1H)	77.6	77.8
3.73 (d, *J* = 15.0 Hz, 1H)	3.71 (d, *J* = 14.8 Hz, 1H)	64.6	64.8
3.26 – 3.20 (m, 1H)	3.23 – 3.09 (m, 3H)	60.2	60.4
3.17 – 3.15 (m, 1H)		60.1	60.3
3.15 (dd, *J* = 17.3, 8.5 Hz, 1H)		52.6	52.8
2.89 (dd, *J* = 18.9, 9.9 Hz, 1H)	2.87 (q, *J* = 10.0 Hz, 1H)	51.9	52.1
2.75 (d, *J* = 14.8 Hz, 1H)	2.73 (d, *J* = 14.8 Hz, 1H)	50.4	50.5
2.68 (dd, *J* = 17.4, 3.2 Hz, 1H)	2.67 (dd, *J* = 17.4, 3.3 Hz, 1H)	48.2	48.4
2.39 (ddd, *J* = 14.3, 4.3, 4.3 Hz, 1H)	2.36 (dt, *J* = 14.4, 4.3 Hz, 1H)	42.8	43.0
1.94 – 1.92 (m, 1H)	1.92 – 1.87 (m, 2H)	42.5	42.7
1.92 – 1.89 (m, 1H)		31.6	31.8
1.48 (d, *J* = 14.3 Hz, 1H)	1.46 (d, *J* = 14.4 Hz, 1H)	26.8	27.0
1.30 (ddd, *J* = 10.5, 3.2, 3.2 Hz, 1H)	1.31 – 1.26 (m, 1H)		

2-Iodo-4,5-dimethoxy-*N*-(4-methoxybenzyl)aniline, 26

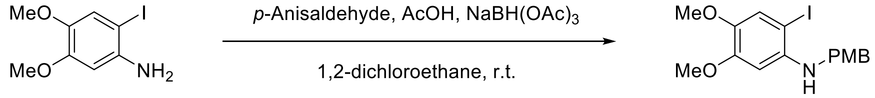

2-iodo-4,5-dimethoxyaniline **26**

To a stirred solution of 2-iodo-4,5-dimethoxyaniline (1.20 g, 4.30 mmol, 1.0 equiv.) in 1,2-dichloroethane (45 mL) at room temperature was added *p*-anisaldehyde (0.58 mL, 4.77 mmol, 1.1 equiv.), AcOH (0.5 mL, 8.74 mmol, 2.0 equiv.) and $NaBH(OAc)_3$ (3.65 g, 17.2 mmol, 4.0 equiv.). The resulting

mixture was stirred for 18 h before it was quenched slowly with $KHCO_3$ (150 mL, sat. aq.). The layers were separated and the aqueous layer was extracted with CH_2Cl_2 (3 × 75 mL), the combined organic layers were washed with water (150 mL), brine (150 mL), dried (Na_2SO_4) and concentrated under reduced pressure. Flash column chromatography (silica gel, pentane:EtOAc 15:1→1:1) afforded compound **26** (1.42 g, 3.56 mmol, 83%) as a yellow amorphous solid. **26:** R_f = 0.68 (silica gel, pentane:Et_2O 1:1); IR (film) ν_{max} 3001, 2835, 1612, 1514, 1465, 1251, 1033, 735 cm^{-1}; ^1H NMR (400 MHz, $CDCl_3$): δ 7.29 (d, J = 8.8 Hz, 2H), 7.16 (s, 1H), 6.89 (d, J = 8.8 Hz, 2H), 6.23 (s, 1H), 4.30 (s, 2H), 3.80 (s, 3H), 3.78 (s, 3H), 3.75 ppm (s, 3H); ^{13}C NMR (101 MHz, $CDCl_3$): δ 159.0, 150.7, 142.6, 141.8, 130.9, 128.7, 122.7, 114.2, 97.1, 72.0, 57.1, 55.9, 55.4, 48.9 ppm; HRMS calcd. For $C_{16}H_{19}INO_3^+$ $[M + H]^+$ 400.0404, found 400.0399.

***tert*-Butyl (4-(trimethylsilyl)but-3-yn-1-yl)carbamate, 27**

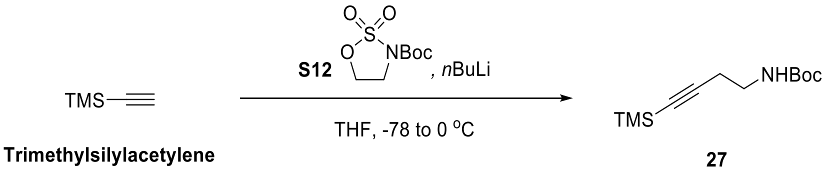

To a stirred solution of trimethylsilyl acetylene (3.0 mL, 21.7 mmol, 1.5 equiv.) in THF (100 mL) at –78 °C was added *n*BuLi (1.6 M solution in hexanes, 14.6 ml, 23.4 mmol, 1.6 equiv.). The reaction mixture was stirred for 15 min before it was warmed to 0 °C and stirred for additional 15 min. The resulting mixture at –78 °C was added *tert*-butyl 1,2,3-oxathiazolidine-3-carboxylate 2,2-dioxide **S12** (3.25 g, 14.6 mmol, 1.0 equiv.) in THF (24 mL). The reaction mixture was stirred for 30 min before it was warmed 0 °C and stirred for additional 1 h. The reaction was quenched with 1 N citric acid (125 mL) and the mixture was stirred vigorously for 30 min. The layers were separated and the aqueous layer was extracted with EtOAc (3 x 100 mL), the combined organic layers were washed with brine (100 mL), dried (Na_2SO_4) and concentrated under reduced pressure. Flash column chromatography (silica gel, pentane:EtOAc 10:1→1:2) afforded compound **27** (2.67 g, 11.1 mmol, 76%) as a white amorphous solid. **27:** R_f = 0.38 (silica gel, petane:Et_2O 10:1); IR (film) ν_{max} 3374, 2980, 1719, 1523, 1252, 847, 762 cm^{-1}; ^1H NMR (400 MHz, $CDCl_3$): δ 4.81 (s, 1H), 3.25 (q, J = 6.5 Hz, 2H), 2.40 (t, J = 6.5 Hz, 2H), 1.44 (s, 9H), 0.14 ppm (s, 9H); ^{13}C NMR (101 MHz, $CDCl_3$): δ 155.7, 104.1, 86.1, 79.2, 39.4, 28.4, 21.3, 0.1 ppm; HRMS calcd. For $C_{12}H_{24}NO_2Si^+$ $[M + H]^+$ 242.1571, found 242.1569.

2-(5,6-Dimethoxy-1-(4-methoxybenzyl)-1*H*-indol-3-yl)ethan-1-amine, 25

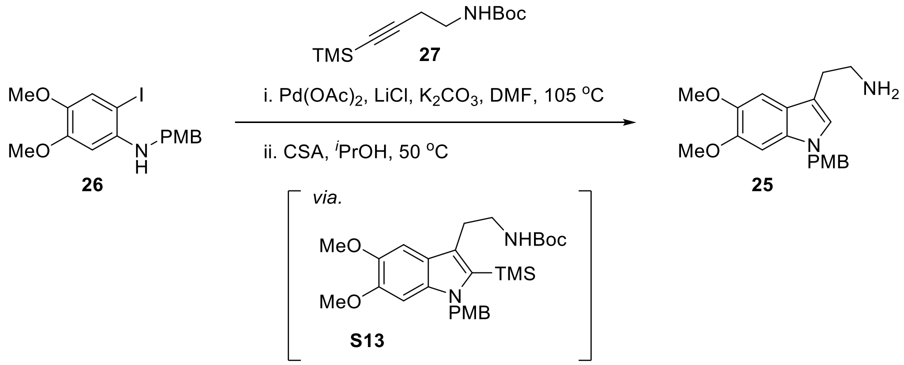

(i) To a solution of aniline **26** (1.67 g, 4.18 mmol, 1.0 equiv.), alkyne **27** (1.40 g, 5.80 mmol, 1.4 equiv.) and LiCl (177 mg, 4.18 mmol, 1.0 equiv.) in DMF (60 mL) at room temperature was added K_2CO_3 (1.73 g, 12.5 mmol, 3.0 equiv.) and $Pd(OAc)_2$ (94.0 mg, 0.42 mmol, 0.1 equiv.). The resulting mixture was warmed to 105 °C and stirred for 15 h before it was cooled to room temperature and quenched with H_2O (50 mL). The layers were separated and the aqueous layer was extracted with EtOAc (3 × 50 mL), the combined organic layers were washed with water (3 × 100 mL), brine (100 mL), dried (Na_2SO_4) and concentrated under reduced pressure. The resulting compound **S13** was used directly in the next step without further purification.

(ii) To a stirred solution of **S13** (crude, obtained above) in 2-isopropanol (65.0 mL) at room temperature was added camphorsulfonic acid (3.10 g, 13.3 mmol, 3.2 equiv.). The resulting mixture was warmed to 50 °C and stirred for 17 h, before being cooled to room temperature and quenched with $NaHCO_3$ (50 mL, sat. aq.). The layers were separated and the aqueous layer was extracted with CH_2Cl_2 (3 × 50 mL), the combined organic layers were dried (Na_2SO_4) and concentrated under reduced pressure. Flash column chromatography (silica gel, CH_2Cl_2:MeOH:NH_4OH 1:0:0→16:1:1) afforded compound **25** (873 mg, 2.56 mmol, 61% over two steps) as a yellow foam. **25**: R_f = 0.25 (silica gel, CH_2Cl_2:MeOH:NH_4OH 16:1:1); IR (film) ν_{max} 3067, 2998, 1613, 1514, 1490, 1249, 1036, 741 cm^{-1}; ^1H NMR (400 MHz, $CDCl_3$): δ 7.05 (d, J = 8.7 Hz, 2H), 7.03 (s, 1H), 6.83 (d, J = 8.7 Hz, 2H), 6.81 (s, 1H), 6.73 (s, 1H), 5.15 (s, 2H), 3.93 (s, 3H), 3.86 (s, 3H), 3.78 (s, 3H), 3.00 (t, J = 6.7 Hz, 2H), 2.85 (t, J = 6.7 Hz, 2H), 1.40 ppm (br s, 2H); ^{13}C NMR (101 MHz, $CDCl_3$): δ 159.2, 147.1, 144.9, 131.3, 129.8, 128.3, 124.6, 121.0, 114.3, 112.7, 101.1, 93.5, 56.6, 56.4, 55.4, 49.7, 42.7, 29.8 ppm; HRMS calcd. For $C_{20}H_{25}N_2O_3^+$ $[M + H]^+$ 341.1860, found 341.1855.

(*Z*)-4-((2-(5,6-dimethoxy-1-(4-methoxybenzyl)-1*H*-indol-3-yl)ethyl)amino)-3-iodobut-2-en-1-yl benzoate, S14

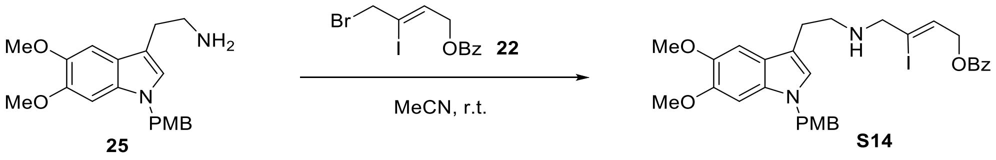

To a stirred solution of **25** (873 mg, 2.56 mmol, 3.0 equiv.) in MeCN (50 mL) at room temperature was added **22** (326 mg, 0.86 mmol, 1.0 equiv.), and the mixture was stirred for 18 h before it was concentrated under reduced pressure. Flash column chromatography (silica gel, pentane:Et_2O 8:1→0:1) afforded compound **S14** (504 mg, 0.79 mmol, 92%) as a brown syrup. **S14**: R_f = 0.51 (silica gel, EtOAc); IR (film) ν_{max} 2951, 2907, 2835, 1720, 1514, 1489, 1271, 1252, 714 cm^{-1}; ^1H NMR (500 MHz, $CDCl_3$): δ 8.04 (d, *J* = 7.0 Hz, 2H), 7.56 (t, *J* = 7.4 Hz, 1H), 7.43 (t, *J* = 7.8 Hz, 2H), 7.06 (s, 1H), 7.04 (d, *J* = 2.5 Hz, 2H), 6.84 (d, *J* = 3.6 Hz, 2H), 6.81 (s, 1H), 6.72 (s, 1H), 6.18 (t, *J* = 5.8 Hz, 1H), 5.14 (s, 2H), 4.88 (d, *J* = 5.7 Hz, 2H), 3.92 (s, 3H), 3.85 (s, 3H), 3.77 (s, 3H), 3.54 (s, 2H), 2.93 (t, *J* = 6.8 Hz, 2H), 2.84 (t, *J* = 6.7 Hz, 2H), 1.70 ppm (s, 1H); ^{13}C NMR (126 MHz, $CDCl_3$): δ 166.4, 159.2, 147.1, 144.8, 133.3, 131.2, 130.7, 130.0, 129.8, 128.5, 128.3, 124.5, 121.0, 114.2, 113.0, 112.5, 101.0, 93.5, 68.7, 61.1, 56.6, 56.4, 55.4, 49.7, 48.1, 25.9 ppm; HRMS calcd. For $C_{31}H_{34}IN_2O_5^+$ $[M + H]^+$ 641.1507, found 641.1497.

(*Z*)-4-((3a*S*,6a*R*,11b*R*)-6-((3a*S*,6*R*,7a*R*)-8,8-dimethyl-2,2-dioxidohexahydro-3*H*-3a,6-methanobenzo[*c*]isothiazole-1-carbonyl)-9,10-dimethoxy-7-(4-methoxybenzyl)-1,2,3a,4,6a,7-hexahydro-3*H*-pyrrolo[2,3-*d*]carbazol-3-yl)-3-iodobut-2-en-1-yl benzoate, 28 and 28'

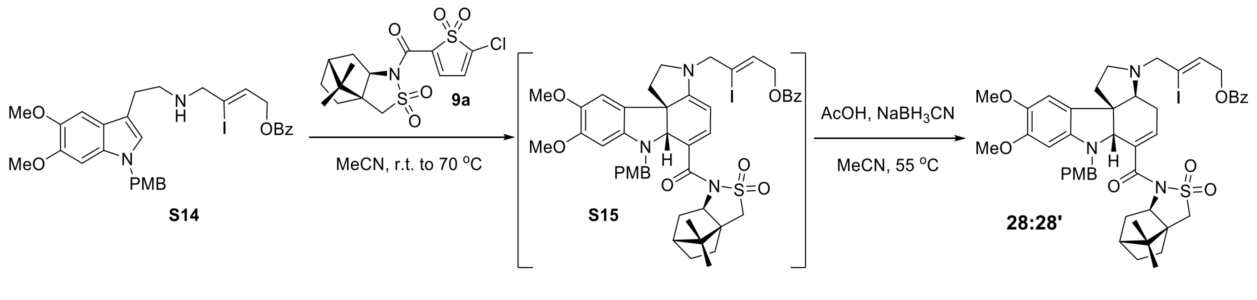

To a stirred solution of tryptamine derivative **S14** (295 mg, 0.46 mmol, 1.0 equiv.) in MeCN (19 mL) at room temperature was added **9a** (181 mg, 0.46 mmol, 1.0 equiv.). The resulting mixture was stirred for

17 h before additional **S14** (354 mg, 0.55 mmol, 1.2 equiv.) was added. The resulting mixture was warmed to 70 °C and stirred for 75 h before it was cooled to 55 °C. AcOH (0.40 mL, 6.99 mmol, 15.2 equiv.) was added, and the resulting mixture was stirred for 40 min at 55 °C. A solution of $NaBH_3CN$ (290 mg, 4.61 mmol, 10.0 equiv.) in MeOH (4.60 mL) was added, and the resulting mixture was stirred at 55 °C for 1 h before it was cooled to room temperature and quenched bey slow addition of $NaHCO_3$ (30 mL, sat. aq.), and then CH_2Cl_2 (30 mL). The layers were separated and the aqueous layer was extracted with CH_2Cl_2 (3 × 30 mL), the combined organic layers were washed with brine (50 mL), dried (Na_2SO_4) and concentrated under reduced pressure. Flash column chromatography (silica gel, pentane:EtOAc 9:1→8:2) afforded compound **28** and its C3a-epimer **28'** (127 mg combined mass, 0.14 mmol, 30%, 1.3:1 *dr*) as a yellow foam. These diastereomers could be separated for the purposes of characterisation, and for the subsequent reduction of **28**.

28 (desired): R_f = 0.39 (silica gel, pentane:EtOAc 1:1); $[\alpha]_D^{25}$ = –58.8 (*c* = 0.17, $CHCl_3$); IR (film) ν_{max} 3008, 2961, 1723, 1672, 1512, 1500, 1272, 762 cm^{-1}; ^1H NMR (400 MHz, $CDCl_3$): 8.05 (d, *J* = 7.2 Hz, 2H), 7.60-7.54 (m, 1H), 7.48-7.39 (m, 3H), 7.22 (d, *J* = 8.6 Hz, 2H), 6.81 (d, *J* = 8.6 Hz, 2H), 6.66 (s, 1H), 6.18 (t, *J* = 5.9 Hz, 1H), 5.98 (s, 1H), 4.89 (d, *J* = 5.8 Hz, 2H), 4.43 (s, 1H), 4.36 (d, *J* = 14.8 Hz, 1H), 4.13 (dt, *J* = 7.0, 2.9 Hz, 1H), 3.85 (d, *J* = 14.0 Hz, 1H), 3.79 (s, 3H), 3.76 (s, 3H), 3.62 (s, 3H), 3.52 (d, *J* = 13.7 Hz, 1H), 3.48 (d, *J* = 13.7 Hz, 1H), 3.38 (d, *J* = 13.7 Hz, 1H), 3.12-3.00 (m, 2H), 2.75 (br s, 1H), 2.71-2.54 (m, 2H), 2.38 (q, *J* = 9.0 Hz, 1H), 2.24-2.17 (m, 1H), 2.04-1.80 (m, 7H), 1.44 (t, *J* = 10.7 Hz, 1H), 1.39-1.31 (m, 1H), 1.23 (s, 3H), 0.97 ppm (s, 3H); ^{13}C NMR (101 MHz, $CDCl_3$): δ 170.0, 166.4, 158.6, 149.6, 149.5, 147.0, 142.4, 133.3, 132.8, 131.3, 130.5, 129.9, 129.6, 128.6, 126.6, 113.8, 111.7, 109.0, 95.3, 72.4, 69.2, 68.7, 65.9, 64.9, 57.6, 56.0, 55.3, 54.7, 53.7, 53.6, 52.7, 48.1, 47.9, 45.1, 38.5, 38.0, 33.1, 26.8, 21.3, 20.0 ppm; HRMS calcd. For $C_{46}H_{53}IN_3O_8S^+$ $[M + H]^+$ 934.2593, found 934.2567.

28' (undesired): R_f = 0.26 (silica gel, pentane:EtOAc 1:1); $[\alpha]_D^{25}$ = –121 (*c* = 1.10, $CHCl_3$); IR (film) ν_{max} 3016, 2958, 2937, 1721, 1667, 1511, 1496, 1338, 1270, 757 cm^{-1}; ^1H NMR (500 MHz, $CDCl_3$): δ 8.04 (d, *J* = 8.1 Hz, 2H), 7.56 (t, *J* = 7.4 Hz, 1H), 7.44 (t, *J* = 7.8 Hz, 2H), 7.34 (s, 1H), 7.15 (d, *J* = 8.6 Hz, 2H), 6.78 (d, *J* = 8.6 Hz, 2H), 6.39 (s, 1H), 6.28 (t, *J* = 5.7 Hz, 1H), 6.08 (s, 1H), 4.94 (d, *J* = 5.6 Hz, 2H), 4.50 (s, 1H), 4.38 (d, *J* = 14.8 Hz, 1H), 4.08 (d, *J* = 14.8 Hz, 1H), 4.04-3.96 (m, 1H), 3.81 (s, 3H), 3.75 (s, 3H), 3.71 (s, 3H), 3.67 (s, 1H), 3.43 (d, *J* = 13.6 Hz, 1H), 3.37 (d, *J* = 13.7 Hz, 1H), 3.16 (q, *J* = 8.3 Hz, 1H), 3.09 (d, *J* = 13.8 Hz, 1H), 2.75 (dd, *J* = 10.6, 4.5 Hz, 1H), 2.38-2.20 (m, 3H), 1.97-1.88 (m, 2H), 1.89-1.74 (m, 3H), 1.63 (q, *J* = 11.0, 9.5 Hz, 2H), 1.44-1.27 (m, 3H), 1.19 (s, 3H), 0.96 ppm (s, 3H); ^{13}C NMR (101 MHz, $CDCl_3$): δ 170.0, 166.4, 158.9, 149.0, 143.6, 142.6, 136.5, 133.3, 131.3, 131.0,

129.9, 129.8, 129.7, 128.6, 125.3, 113.7, 111.5, 110.2, 97.4, 68.8, 68.7, 66.7, 65.8, 64.7, 57.2, 56.0, 55.4, 54.9, 53.9, 53.3, 50.6, 48.4, 47.8, 45.2, 38.5, 37.2, 33.2, 27.3, 26.6, 21.2, 20.0 ppm; HRMS calcd. For $C_{46}H_{53}IN_3O_8S^+$ $[M + H]^+$ 934.2593, found 934.2557.

(*Z*)-4-((3a*S*,6a*R*,11b*R*)-6-(hydroxymethyl)-9,10-dimethoxy-7-(4-methoxybenzyl)-1,2,3a,4,6a,7-hexahydro-3*H*-pyrrolo[2,3-*d*]carbazol-3-yl)-3-iodobut-2-en-1-ol, S16

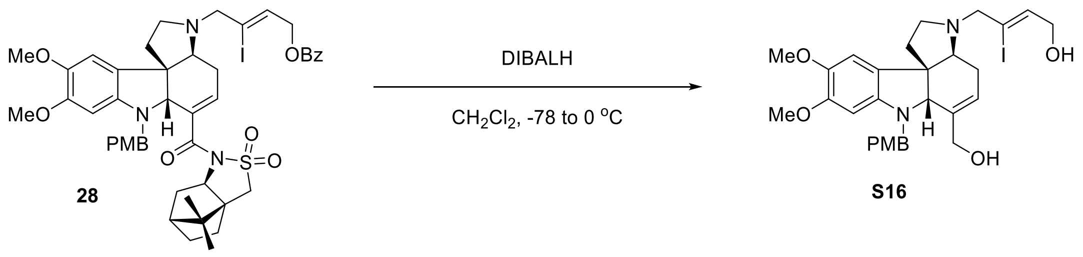

To a stirred solution of **28** (72.5 mg, 77.6 µmol, 1.0 equiv.) in CH_2Cl_2 (3.5 mL) at-78 °C was added DIBALH (1.0 M solution in hexane, 0.31 mL, 0.31 mmol, 4.0 equiv.). The resulting mixture was stirred for 30 min before it was warmed to 0 °C and stirred for additional 2 h. The resulting mixture again cooled to -78 °C and additional DIBALH (0.16 mL, 0.16 mmol, 2.1 equiv) was added. The resulting mixture was warmed to 0 °C and stirred for 1 h before it was quenched with Rochelle's salt (6 mL, sat. aq.). EtOAc (1 mL) was added and the mixture was stirred vigorously for 1 h. The layers were separated and the aqueous layer was extracted with CH_2Cl_2 (3 × 8 mL), the combined organic layers were washed with water (15 mL), brine (15 mL), dried (Na_2SO_4) and concentrated under reduced pressure. Flash column chromatography (silica gel, pentane:EtOAc 4:1→EtOAc:Et_3N 16:1) afforded compound **S16** (34.6 mg, 55.9 µmol, 72%) as a yellow oil. **S16:** R_f = 0.67 (silica gel, EtOAc); $[\alpha]_D^{25}$ = –18.5 (*c* = 0.39, $CHCl_3$); IR (film) ν_{max} 3329, 3006, 2935, 2835, 1511, 1499, 1248, 1222, 1037, 761 cm^{-1}; ^1H NMR (400 MHz, $CDCl_3$): δ 7.18 (d, *J* = 8.6 Hz, 2H), 6.82 (d, *J* = 8.7 Hz, 2H), 6.69 (s, 1H), 6.20 (t, *J* = 5.4 Hz, 1H), 6.05 (s, 1H), 5.98-5.91 (m, 1H), 4.35 (d, *J* = 15.3 Hz, 1H), 4.23 (d, *J* = 5.4 Hz, 2H), 4.16-4.01 (m, 3H), 3.86-3.81 (m, 4H), 3.78 (s, 4H), 3.70 (s, 3H), 3.53 (d, *J* = 14.7 Hz, 1H), 3.21 (d, *J* = 14.8 Hz, 1H), 3.08 (t, *J* = 8.1 Hz, 1H), 2.76 (s, 1H), 2.50 (q, *J* = 8.7 Hz, 1H), 2.31 (d, *J* = 16.2 Hz, 1H), 2.28-2.19 (m, 1H), 2.12-2.05 (m, 1H), 1.74-1.63 ppm (m, 1H); ^{13}C NMR (101 MHz, $CDCl_3$): δ 158.8, 149.7, 146.8, 142.7, 138.1, 135.6, 131.1, 129.0, 127.7, 127.1, 114.0, 108.9, 106.9, 95.3, 70.3, 70.2, 67.0, 67.0, 65.5, 57.6, 56.1, 55.4, 54.5, 54.3, 53.3, 39.3, 25.1 ppm; HRMS calcd. For $C_{29}H_{36}IN_2O_5^+$ $[M + H]^+$ 619.1664, found 619.1656.

(–)-Brucine

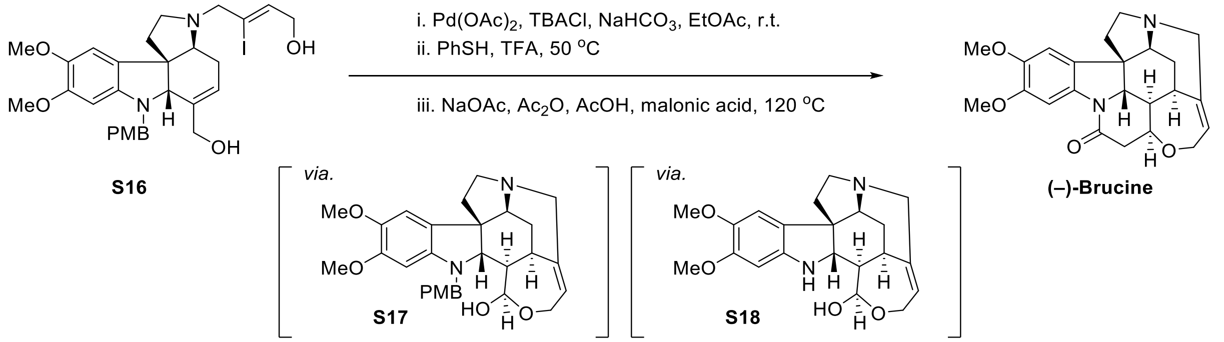

According to a modified literature procedure.[8] (i) To a stirred solution of **S16** (23.6 mg, 38.2 μmol, 1.0 equiv.) in EtOAc (3.0 mL) at room temperature was added tetra-*n*-butylammonium chloride (31.7 mg, 0.11 mmol, 2.9 equiv.), $NaHCO_3$ (31.7 mg, 0.38 mmol, 10.0 equiv.) and $Pd(OAc)_2$ (2.6 mg, 11.6 μmol, 0.3 equiv.). The resulting mixture was stirred for 20 h before it was concentrated under reduced pressure. Flash column chromatography (silica gel, CH_2Cl_2:MeOH:NH_4OH 20:1:1) afforded compound **S17** (11.2 mg, 22.8 μmol, 60%) as a tan sticky solid.

(ii) To a stirred solution of **S17** (11.2 mg, 22.8 μmol, 1.0 equiv.) in TFA (1.5 mL) at room temperature was added thiophenol (24 μL, 0.23 mmol, 10.1 equiv.). The resulting mixture was warmed to 50 °C and stirred for 18 h before it was concentrated under reduced pressure. The residue was diluted with CH_2Cl_2 (3 mL) and the solution was stirred vigorously with 1 N HCl (3 mL) for 10 min. The aqueous layer was washed with CH_2Cl_2 (3×5 mL) and then basified with ammonium hydroxide solution (1.5 mL, 35 wt%). The resulting solution was extracted with CH_2Cl_2 (5×5 mL), the combined organic layers were dried (Na_2SO_4) and concentrated under reduced pressure. The resulting compound (the brucine-Wieland-Gumlich aldehyde **S18**) was used directly in the next step without further purification.

^{1}H NMR spectrum (400 MHz, $CDCl_3$) of crude brucine-Wieland-Gumlich aldehyde, S18

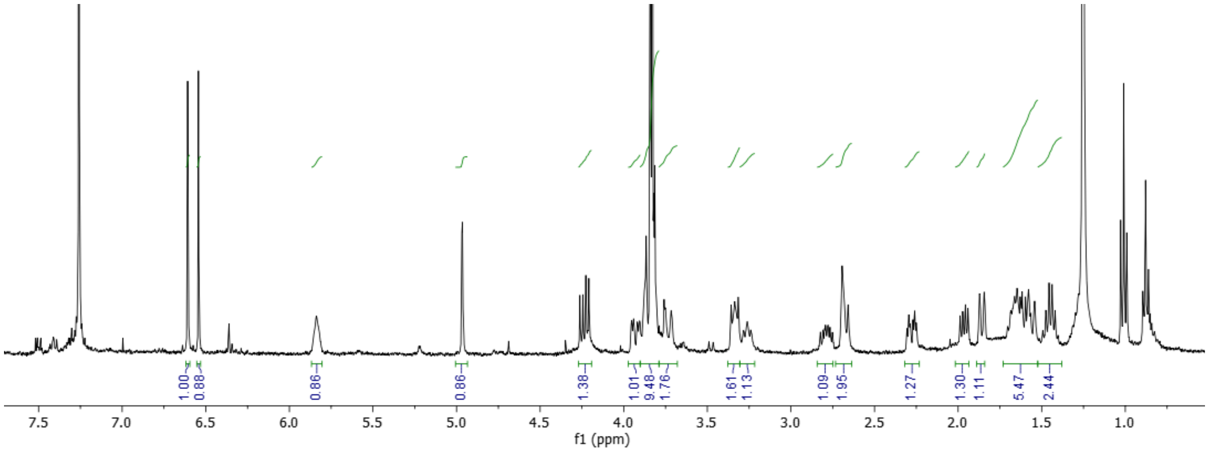

(iii) To a stirred solution of **S18** (6.0 mg, 16 μmol, 1.0 equiv.) in AcOH (1.0 mL) at room temperature was added sodium acetate (40 mg, 0.49 mmol, 30 equiv.), acetic anhydride (15 μL, 0.16 mmol, 10 equiv.) and malonic acid (40 mg, 0.38 mmol, 24 equiv.). The resulting mixture was warmed to 120 °C and stirred for 3 h. The resulting residue was cooled to room temperature and diluted with water (3 mL) and basified with NaOH (1 mL, 1N aq.). The resulting mixture was extracted with CH_2Cl_2 (3 × 5 mL), the combined organic layers were dried (Na_2SO_4) and concentrated under reduced pressure. Flash column chromatography (silica gel, CH_2Cl_2:MeOH:NH_4OH 20:1:1) afforded **(–)-brucine** (4.5 mg, 12 μmol, 30% over three steps) as a clear sticky solid. **(–)-Brucine**: R_f = 0.26 (silica gel, CH_2Cl_2:MeOH:NH_4OH 12:1:1); $[\alpha]_D^{25}$ = –113 (c = 0.03, $CHCl_3$); IR (film) ν_{max} 2960, 2923, 1665, 1502, 1467, 1403, 1285, 1263, 1118, 806, 750 cm^{-1}; ^{1}H NMR (500 MHz, $CDCl_3$): δ 7.81 (s, 1H), 6.67 (s, 1H), 5.89 (t, J = 6.1 Hz, 1H), 4.28 (dt, J = 8.2, 3.2 Hz, 1H), 4.14 (dd, J = 13.8, 7.0 Hz, 1H), 4.06 (dd, J = 13.9, 5.9 Hz, 1H), 3.90 (s, 3H), 3.88-3.84 (m, 4H), 3.82 (d, J = 10.5 Hz, 1H), 3.70 (d, J = 14.7 Hz, 1H), 3.21-3.06 (m, 3H), 2.88-2.80 (m, 1H), 2.71 (d, J = 14.8 Hz, 1H), 2.65 (dd, J = 20.6, 3.2 Hz, 1H), 2.40-2.32 (m, 1H), 1.93-1.82 (m, 2H), 1.46 (d, J = 14.3 Hz, 1H), 1.30-1.25 ppm (m, 1H); ^{13}C NMR (126 MHz, $CDCl_3$): δ 169.1, 149.4, 146.4, 140.8, 136.1, 127.4, 123.7, 105.7, 101.2, 78.0, 64.8, 60.6, 60.1, 56.6, 56.4, 52.9, 52.1, 50.4, 48.5, 42.6, 42.6, 31.7, 27.0 ppm; HRMS calcd. For $C_{23}H_{27}N_2O_4^+$ $[M + H]^+$ 395.1965, found 395.1962.

Table S9. ^{1}H and ^{13}C NMR ($CDCl_3$, ppm) comparison for brucine with commercial material

Commercial Brucine (^{1}H, 500 MHz, ppm)	This work (^{1}H, 500 MHz, ppm)	Commercial Brucine (^{13}C, 126 MHz, ppm)	This work (^{13}C, 126 MHz, ppm)
7.81 (s, 1H)	7.81 (s, 1H)	169.1	169.1
6.66 (s, 1H)	6.67 (s, 1H)	149.3	149.4
5.93 – 5.84 (m, 1H)	5.89 (t, J = 6.1 Hz, 1H)	146.3	146.4
4.28 (dt, J = 8.5, 3.3 Hz, 1H)	4.28 (dt, J = 8.2, 3.2 Hz, 1H)	140.8	140.8
4.14 (dd, J = 13.8, 7.0 Hz, 1H)	4.14 (dd, J = 13.8, 7.0 Hz, 1H)	136.1	136.1
4.06 (dd, J = 13.8, 6.1 Hz, 1H)	4.06 (dd, J = 13.9, 5.9 Hz, 1H)	127.3	127.4
3.90 (s, 3H)	3.90 (s, 3H)	123.7	123.7
3.85 (s, 4H)	3.88 – 3.84 (m, 4H)	105.7	105.7
3.82 (d, J = 10.4 Hz, 1H)	3.82 (d, J = 10.5 Hz, 1H)	101.1	101.2
3.69 (d, J = 14.7 Hz, 1H)	3.70 (d, J = 14.7 Hz, 1H)	77.9	78.0
3.20 – 3.06 (m, 3H)	3.21 – 3.06 (m, 3H)	64.8	64.8
2.84 (ddd, J = 12.3, 10.0, 6.7 Hz, 1H)	2.88 – 2.80 (m, 1H)	60.5	60.6
2.71 (d, J = 14.8 Hz, 1H)	2.71 (d, J = 14.8 Hz, 1H)	60.1	60.1
2.65 (dd, J = 17.4, 3.4 Hz, 1H)	2.65 (dd, J = 20.6, 3.2 Hz, 1H)	56.6	56.6
2.35 (dt, J = 14.3, 4.4 Hz, 1H)	2.40 – 2.32 (m, 1H)	56.4	56.4
1.93 – 1.81 (m, 2H)	1.93 – 1.82 (m, 2H)	52.9	52.9
1.46 (dt, J = 14.3, 2.2 Hz, 1H)	1.46 (d, J = 14.3 Hz, 1H)	52.1	52.1
1.26 (dt, J = 10.4, 3.2 Hz, 1H)	1.30 – 1.25 ppm (m, 1H)	50.4	50.4
		48.5	48.5
		42.6	42.6
		42.6	42.6
		31.7	31.7
		27.0	27.0

2.2 Mechanistic Investigation

To explore the reaction pathway of the planned intramolecular cascade, we first treated *N*-benzyl tryptamine with ester-substituted thiophene dioxide **9e** (see **Scheme S2**). This enabled the isolation and full characterisation of intermediate **S19**. On heating to 80 °C in MeCN, **S19** was converted into the expected cycloadduct **S20** via the intramolecular IEDDA cascade.

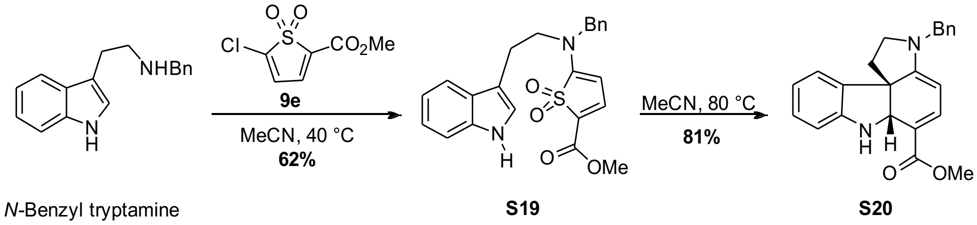

Scheme S2. Validation of the substitution / IMDA cascade for ester-substituted TDO **8**.

On repeating this experiment using the sultam-substituted TDO **9a** and tryptamine derivative **11**, we observed solely the aza-Michael adduct **S23** (**Scheme S3**). This was the major product observed after reaction at room temperature, or at 40 °C. We suspect that trace amounts of the expected adduct **S24** were detected in the NMR spectrum of the crude reaction mixture, but this could not be isolated or characterised with certainty.

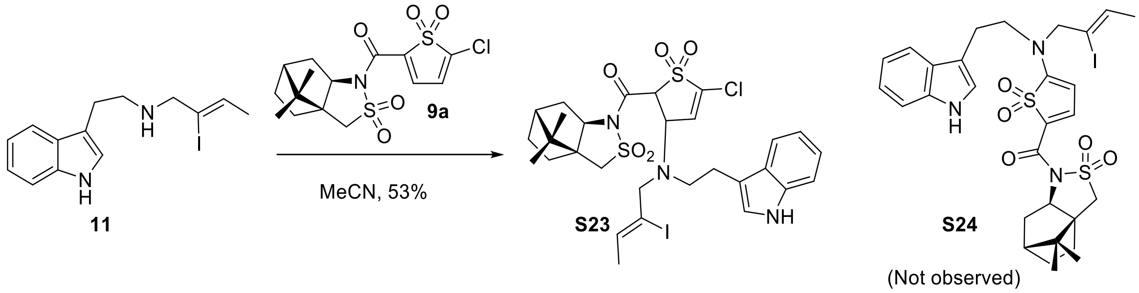

Scheme S3. Formation of aza-Michael adduct **S23** from TDO **9a** and tryptamine **11**.

To further explore the mechanism, compound **S23** was submitted to thermal conditions in the presence of additional **11** and we found that **S23** was converted to the desired cycloadduct **13** (**Scheme S4**). No *N*-substituted thiophene *S,S*-dioxide intermediate (**S24**, above) could be observed in this process. Later on, VT NMR (In CD_3CN at 65 °C, using CH_2Br_2 as internal standard) studies were carried out and clearly showed the consumption of **S23** and formation of **13**. We believe that the additional equivalent of **11** that is added to the reaction mediates the retro-aza-Michael process to reform compound **9a.** See additional conclusions below on possible reaction pathways from **Scheme 4**.

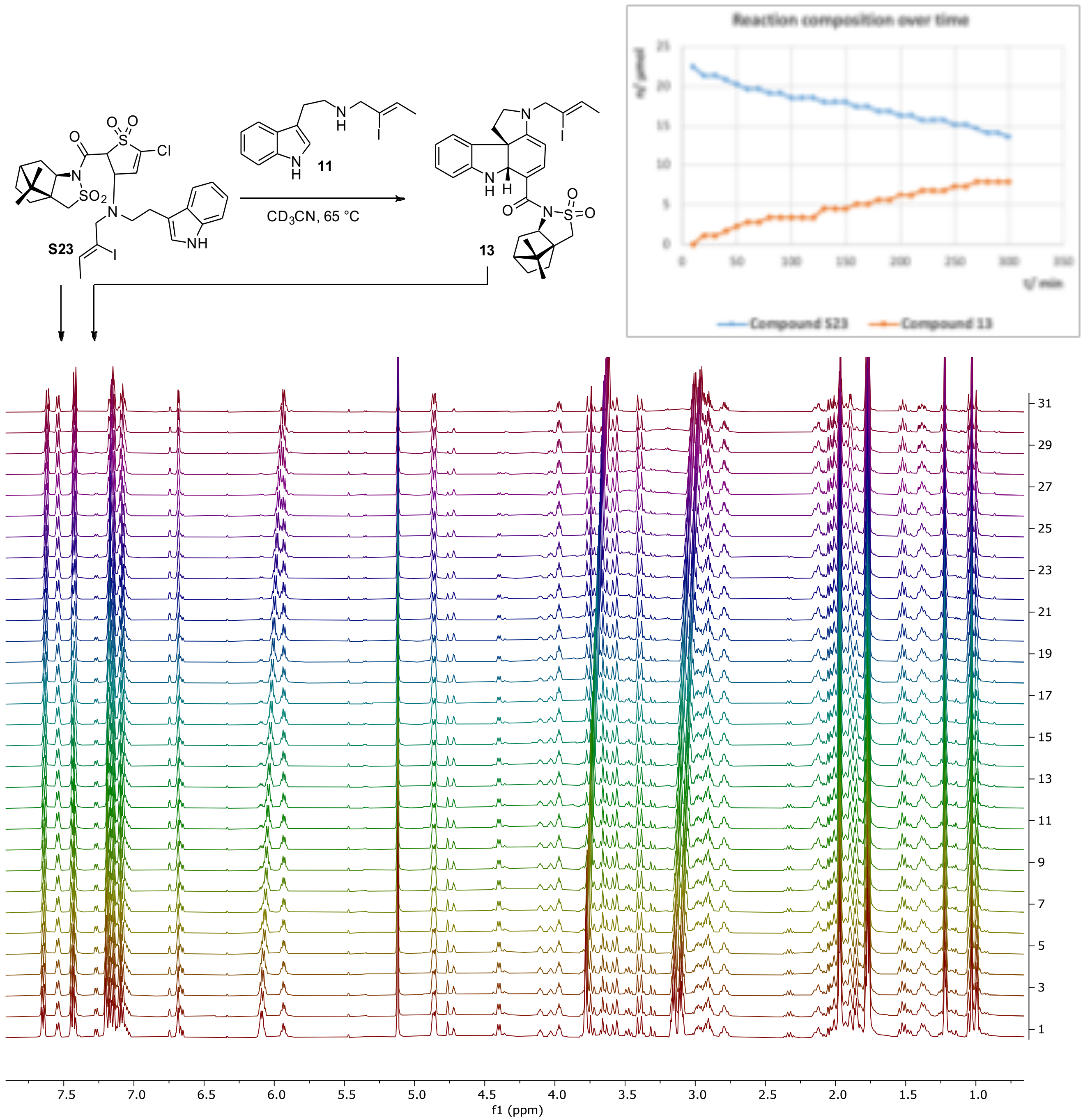

Scheme S4. Conversion of aza-Michael adduct **S23** to cycloadduct **13** and VT NMR experiment

As an intermolecular pathway may be responsible for the formation of **13**, we also studied the reaction of **9a** with compound **S21**, (**Scheme S5**). This substrate is unable to effect chloride substitution, and so must react via intermolecular Diels–Alder reaction. We were pleased to find that **S21** reacted smoothly with **9a** to deliver adduct **S22** in 86% isolated yield. TFA deprotection and basification afforded **13**, which was identical in all respects to that prepared by the presumed tethered, intramolecular route. This

result shows that the intermolecular DA reaction can indeed compete with the intramolecular process, if it operates.

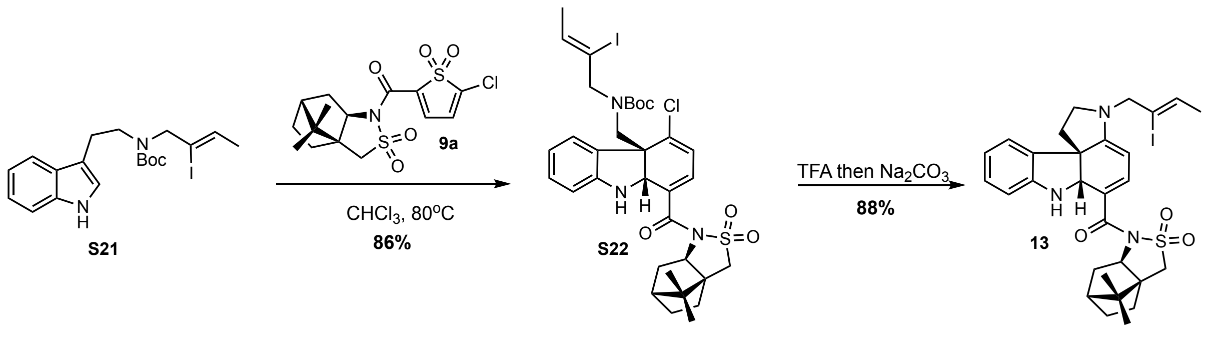

Scheme S5. Intramolecular DA sequence / Boc removal / cyclisation to **13**.

Data for mechanistic study:

Methyl 5-((2-(1H-indol-3-yl)ethyl)(benzyl)amino)thiophene-2-carboxylate 1,1-dioxide, S19

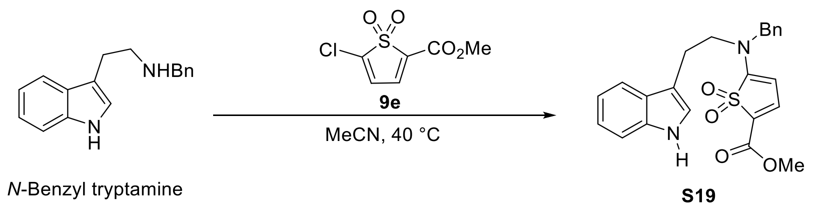

To a stirred solution of ***N*-Benzyl tryptamine** (38 mg, 0.15 mmol, 2.0 equiv.) in MeCN (0.75 mL) at room temperature was added **9e** (15.6 mg, 0.075 mmol, 1.0 equiv.). The reaction mixture was heated at 40 °C for 18 h. Then the reaction mixture was concentrated in vacuo. Flash column chromatography (silica gel, pentane:EtOAc 10:1→3:1) afforded compound **S19** (19.7 mg, 46.6 µmol, 62%) as a red oil. **S19:** R_f = 0.62 (silica gel, pentane:EtOAc 1:1); IR (film) ν_{max} 2980, 1672, 1541, 1458, 1367, 1249, 1162, 955, 806, 740 cm^{-1}; ^1H NMR (500 MHz, $CDCl_3$) δ 8.10 (s, 1H), 7.68 (d, J = 6.1 Hz, 1H), 7.48 (d, J = 7.9 Hz, 1H), 7.39-7.31 (m, 4H), 7.26-7.18 (m, 3H), 7.13 (t, J = 7.5 Hz, 1H), 7.03 (s, 1H), 5.00 (d, J = 6.1 Hz, 1H), 4.61 (s, 2H), 3.86 (s, 3H), 3.76-3.66 (m, 2H), 3.10 ppm (t, J = 7.6 Hz, 2H).; ^{13}C NMR (126 MHz, $CDCl_3$) δ 159.4, 155.8, 146.9, 136.4, 134.6, 129.2, 128.6, 128.2, 127.1, 122.7, 122.5, 119.9, 118.5, 115.3, 111.6, 111.6, 86.4, 54.8, 52.2, 51.2, 23.8 ppm; HRMS calcd. For $C_{23}H_{23}N_2O_4S^+$ $[M + H]^+$ 423.1373, found 423.1369.

Methyl (6a*R*,11b*S*)-3-benzyl-2,3,6a,7-tetrahydro-1H-pyrrolo[2,3-d]carbazole-6-carboxylate, S20

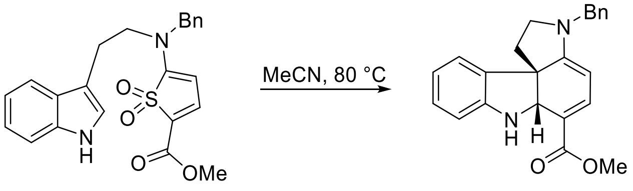

S19 **S20**

A solution of **S19** (12 mg, 28.4 μmol, 1.0 equiv.) in MeCN (1.0 mL) was heated to 80 °C for 16 h. Then the reaction mixture was concentrated in vacuo. Flash column chromatography (silica gel, pentane:EtOAc 10:1→3:1) afforded compound **S20** (8.2 mg, 22.9 μmol, 81%) as a yellow foam. **S20:** R_f = 0.72 (silica gel, pentane:EtOAc 1:1); IR (film) ν_{max} 2958, 1647, 1522, 1477, 1325, 1272, 1120, 946, 758 cm^{-1}; ^{1}H NMR (500 MHz, $CDCl_3$) δ 7.44-7.38 (m, 2H), 7.35 (t, J = 6.5 Hz, 3H), 7.30 (d, J = 7.2 Hz, 1H), 7.04 (t, J = 7.6 Hz, 1H), 6.69 (t, J = 8.1 Hz, 2H), 6.60 (t, J = 7.4 Hz, 1H), 5.50 (s, 1H), 4.80 (d, J = 7.1 Hz, 1H), 4.71 (s, 1H), 4.58-4.45 (m, 2H), 3.67 (s, 3H), 3.64 (dd, J = 10.5, 5.8 Hz, 1H), 3.41-3.31 (m, 1H), 2.31-2.21 (m, 1H), 2.12 ppm (dd, J = 11.8, 5.8 Hz, 1H); ^{13}C NMR (126 MHz, $CDCl_3$) δ 168.4, 159.0, 149.6, 142.3, 136.4, 133.4, 129.1, 128.7, 128.2, 128.1, 121.6, 118.6, 110.8, 110.6, 82.7, 66.8, 57.4, 51.0, 50.7, 49.7, 37.9 ppm; HRMS calcd. For $C_{23}H_{22}N_2NaO_2^+$ $[M + Na]^+$ 381.1573, found 381.1575.

***tert*-Butyl (*Z*)-(2-(1H-indol-3-yl)ethyl)(2-iodobut-2-en-1-yl)carbamate, S21**

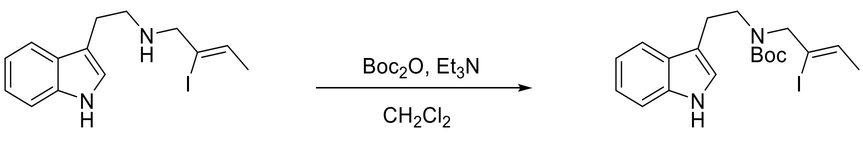

11 **S21**

To a stirred solution of **11** (500 mg, 1.5 mmol, 1.0 equiv.) in CH_2Cl_2 (10 mL) at room temperature was added Et_3N (0.41 mL, 3.0 mmol, 2.0 equiv.) and Boc_2O (0.3 mL, 1.7 mmol, 1.1 equiv.). The mixture was stirred for 3 h before it was concentrated under reduced pressure. Flash column chromatography (silica gel, pentane:Et_2O 5:1→2:1) afforded compound **S21** (618 mg, 1.4 mmol, 95%) as a white solid, which exhibited rotamers in its NMR spectra. **S21**: R_f = 0.61 (silica gel, pentane:EtOAc 1:3); IR (film) ν_{max} 2920, 2842, 1519, 1354, 1140, 962, 742 cm^{-1}; ^{1}H NMR (500 MHz, $CDCl_3$) δ 8.23 (s, 1H), 7.60 (d, J = 7.8 Hz, 1H), 7.36 (t, J = 6.5 Hz, 1H), 7.19 (d, J = 8.0 Hz, 1H), 7.13 (d, J = 7.7 Hz, 1H), 6.98 (d, J = 11.9

Hz, 1H), 5.66 (d, J = 6.7 Hz, 1H), 4.07 (d, J = 39.7 Hz, 2H), 3.59-3.40 (m, 2H), 2.99 (dt, J = 14.8, 7.5 Hz, 2H), 1.77 (d, J = 6.3 Hz, 3H), 1.49 ppm (d, J = 21.8 Hz, 9H); ^{13}C NMR (126 MHz, $CDCl_3$) δ 155.8, 155.3, 136.5, 131.7, 131.4, 127.6, 127.5, 122.1, 122.0, 119.3, 118.9, 118.8, 113.4, 113.3, 111.4, 111.3, 106.7, 106.5, 80.1, 80.0, 58.7, 57.7, 47.0, 46.7, 28.5, 24.5, 23.9, 21.7, 21.7 ppm; HRMS calcd. For $C_{19}H_{26}IN_2O_2^+$ $[M + H]^+$ 441.1033, found 441.1028.

tert-Butyl (2-((4a*S*,9a*R*)-1-((3a*S*,6*R*,7a*R*)-8,8-dimethyl-2,2-dioxidohexahydro-3H-3a,6 methano-benzo[c]isothiazole-1-carbonyl)-9,9a-dihydro-4a*H*-carbazol-4a-yl)ethyl)((*Z*)-2-iodobut-2-en-1-yl)carbamate, S22

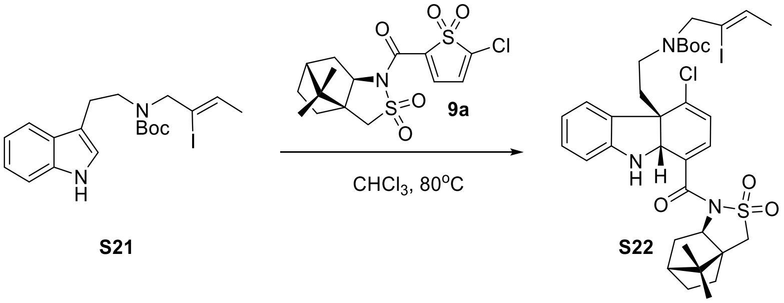

To a stirred solution of **S21** (270 mg, 0.62 mmol, 2.0 equiv.) in $CHCl_3$ (3 mL) at room temperature was added compound **9a** (120 mg, 0.31 mmol, 1.0 equiv.) and the mixture was heated to 80 °C for 16 h, before it was concentrated under reduced pressure. Flash column chromatography (silica gel, pentane:EtOAc 6:1→3:1) afforded compound **S22** (202 mg, 306 µmol, 86%) as a yellow oil, which exhibited rotamers in its NMR spectra. **S22**: R_f = 0.32 (silica gel, pentane:EtOAc 1:3); $[\alpha]_D^{25}$ = –218 (*c* = 1.0, $CHCl_3$); IR (film) ν_{max} 2981, 2888, 1697, 1686, 1541, 1377, 1337, 1220, 1071, 954, 760 cm^{-1}; ^{1}H NMR (500 MHz, $CDCl_3$) δ 7.35 (dd, J = 22.7, 7.6 Hz, 1H), 7.06 (d, J = 7.7 Hz, 1H), 6.78 (dd, J = 9.6, 6.8 Hz, 2H), 6.69-6.61 (m, 1H), 6.31 (d, J = 6.4 Hz, 1H), 5.89-5.77 (m, 1H), 4.82 (d, J = 13.6 Hz, 1H), 4.26-3.95 (m, 4H), 3.57-3.41 (m, 2H), 3.14-3.09 (m, 1H), 2.43 (p, J = 7.5 Hz, 1H), 2.05-1.87 (m, 6H), 1.78 (d, J = 6.2 Hz, 3H), 1.48 (d, J = 22.6 Hz, 9H), 1.25 (s, 3H), 1.00 ppm (s, 3H); ^{13}C NMR (101 MHz, $CDCl_3$) δ 169.1, 161.3, 155.4, 154.8, 149.4, 144.8, 144.4, 136.0, 133.4, 133.2, 132.7, 131.5, 129.9, 129.8, 128.5, 127.4, 126.5, 126.4, 121.4, 119.3, 110.1, 106.2, 106.0, 80.3, 80.1, 74.7, 67.2, 66.5, 65.9, 65.6, 65.5, 62.6, 58.3, 57.7, 53.8, 53.3, 48.2, 48.1, 47.9, 45.3, 44.5, 40.8, 38.3, 35.5, 34.2, 33.3, 32.9, 28.6, 26.6, 26.6, 22.4, 21.9, 21.4, 20.5, 20.1, 20.0, 15.4, 15.1, 14.2 ppm; HRMS calcd. For $C_{34}H_{43}ClIN_3NaO_5S^+$ $[M + Na]^+$ 790.1549, found 790.1533.

((3a*S*,6*R*,7a*R*)-8,8-dimethyl-2,2-dioxidotetrahydro-3*H*-3a,6-methanobenzo[c]isothiazol-1(4*H*)-yl)((6a*R*,11b*S*)-3-((*Z*)-2-iodobut-2-en-1-yl)-2,3,6a,7-tetrahydro-1*H*-pyrrolo[2,3-d]carbazol-6-yl)methanone, 13

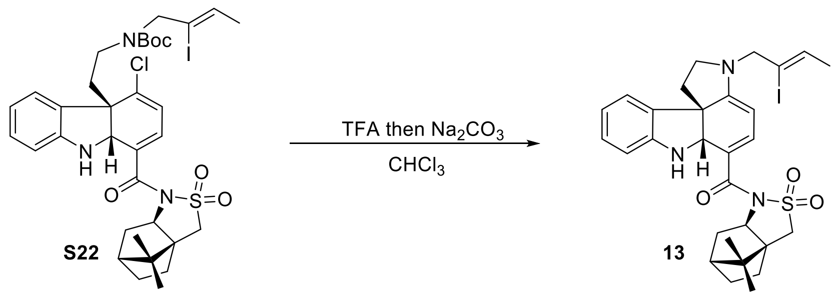

To a stirred solution of **S22** (150 mg, 0.2 mmol, 1.0 equiv.) in $CHCl_3$ (3.0 mL) at room temperature was added TFA (1.0 mL). The resulting mixture was stirred for 2.5 h before it was cooled to 0 °C and quenched by slow addition of Na_2CO_3 (20 mL, sat. aq.), and stirred for 20 h. The layers were separated and the aqueous layer was extracted with CH_2Cl_2 (3 × 10 mL), then the combined organic layers were dried (Na_2SO_4) and concentrated under reduced pressure. Flash column chromatography (silica gel, pentane:EtOAc 5:1→2:1) afforded compound **13** (106 mg, 168 μmol, 88%) as a yellow foam. **13**: R_f = 0.55 (silica gel, pentane:EtOAc 1:1); $[\alpha]_D^{25}$ = 541.9 (*c* = 1.0, $CHCl_3$); IR (film) ν_{max} 2981, 2889, 1676, 1617, 1458, 1386, 1153, 1084, 955, 765 cm^{-1}; ^1H NMR (400 MHz, $CDCl_3$) δ 7.25 (dd, J = 7.3, 1.3 Hz, 1H), 7.06 (td, J = 7.6, 1.3 Hz, 1H), 7.00 (d, J = 7.1 Hz, 1H), 6.68 (ddd, J = 8.4, 7.5, 1.4 Hz, 2H), 6.00-5.92 (m, 1H), 4.90-4.73 (m, 3H), 4.35-4.24 (m, 2H), 4.18-4.11 (m, 1H), 3.77 (td, J = 10.6, 5.8 Hz, 1H), 3.42 (dd, J = 10.3, 8.6 Hz, 1H), 3.34-3.19 (m, 2H), 2.29 (td, J = 11.3, 8.6 Hz, 1H), 2.14 (dd, J = 11.8, 5.7 Hz, 1H), 1.95-1.79 (m, 8H), 1.45-1.30 (m, 2H), 1.18 (s, 3H), 0.92 ppm (s, 3H); ^{13}C NMR (101 MHz, $CDCl_3$): δ 169.0, 160.3, 149.7, 146.7, 134.1, 132.2, 128.6, 121.9, 118.9, 118.1, 110.7, 102.3, 84.8, 67.2, 65.0, 58.9, 57.3, 53.1, 50.3, 48.0, 47.8, 45.3, 37.9, 37.8, 33.1, 26.9, 21.9, 21.2, 20.0. ppm; HRMS calcd. For $C_{29}H_{35}IN_3O_3S^+$ $[M + H]^+$ 632.1438, found 632.1426.

((3a*S*,6*R*,7a*R*)-8,8-dimethyl-2,2-dioxidotetrahydro-3*H*-3a,6-methanobenzo[c]isothiazol-1(4*H*)-yl)((6a*R*,11b*S*)-3-((*Z*)-2-iodobut-2-en-1-yl)-2,3,6a,7-tetrahydro-1*H*-pyrrolo[2,3-d]carbazol-6-yl)methanone, 13 (One pot procedure)

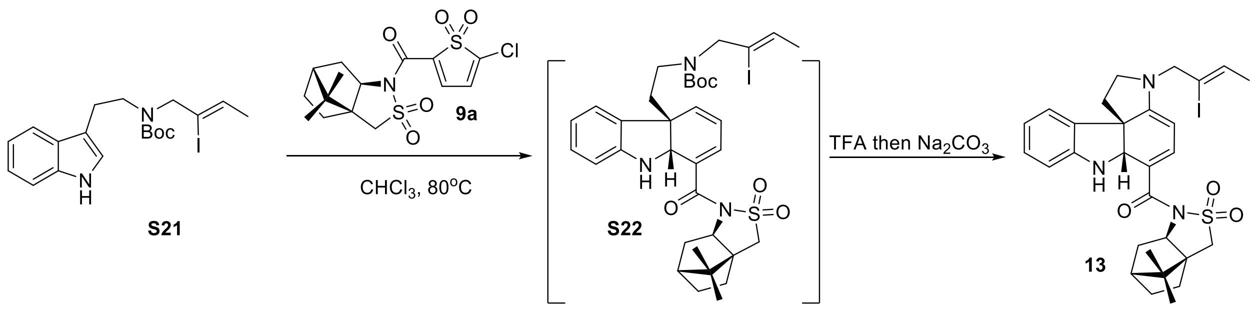

To a stirred solution of **S21** (351 mg, 0.8 mmol, 2.0 equiv.) in $CHCl_3$ (4 mL) at room temperature was added compound **9a** (156 mg, 0.4 mmol, 1.0 equiv.) The resulting mixture was warmed to 80 °C and stirred for 23 h, before it was cooled to 0 °C. TFA (1.3 mL) was added (to the intermediate diene **S22**). The resulting mixture was warmed to room temperature and stirred for 2.5 h before it was cooled to 0 °C and quenched by slow addition of Na_2CO_3 (26 mL, sat. aq.), and stirred for 20 h. The layers were separated and the aqueous layer was extracted with CH_2Cl_2 (3 × 15 mL), then the combined organic layers were dried (Na_2SO_4) and concentrated under reduced pressure. Flash column chromatography (silica gel, pentane:EtOAc 5:1→2:1) afforded compound **13** (196 mg, 0.31 mmol, 78%) as a yellow foam. All the physical data of **13** are identical to those obtained from the two-step procedure.

(3-((2-(1*H*-indol-3-yl)ethyl)((*Z*)-2-iodobut-2-en-1-yl)amino)-5-chloro-1,1-dioxido2,3-dihydrothiop-hen-2-yl)((3a*S*,6*R*,7a*R*)-8,8-dimethyl-2,2-dioxidotetrahydro-3*H*-3a,6 methanobenzo [c] isothiazol - 1(4*H*) yl)methanone, S23

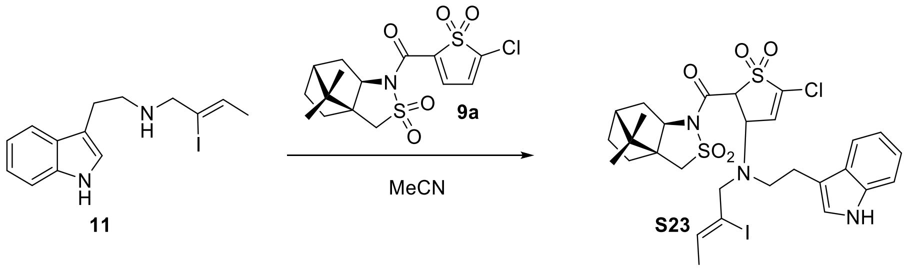

To a stirred solution of **11** (34 mg, 0.1 mmol, 1.0 equiv.) in MeCN (1 mL) at room temperature was added compound **9a** (39 mg, 0.1 mmol, 1.0 equiv.) The mixture was stirred for 16 h before it was concentrated

under reduced pressure. Flash column chromatography (silica gel, pentane:Et_2O 5:1→1:1) afforded compound **S23** (39 mg, 0.053 mmol, 53%) as a yellow oil. **S23**: R_f = 0.78 (silica gel, pentane:EtOAc 1:1); $[\alpha]_D^{25}$ = –67.5 (c = 1.0, $CHCl_3$); IR (film) ν_{max} 3382, 2918, 1608, 1522, 1325, 1166, 1053, 946, 874, 669 cm^{-1}; ^1H NMR (400 MHz, $CDCl_3$) δ 8.02 (s, 1H), 7.54 (dd, J = 7.9, 1.1 Hz, 1H), 7.35 (dt, J = 8.0, 1.0 Hz, 1H), 7.22-7.14 (m, 1H), 7.13-7.06 (m, 2H), 6.43 (d, J = 3.5 Hz, 1H), 5.82 (q, J = 6.2 Hz, 1H), 4.86 (dt, J = 5.7, 2.5 Hz, 2H), 3.86 (dd, J = 7.8, 4.8 Hz, 1H), 3.63-3.44 (m, 3H), 3.28 (d, J = 14.1 Hz, 1H), 3.03-2.85 (m, 3H), 2.85-2.73 (m, 1H), 2.21 (ddd, J = 10.6, 4.7, 2.3 Hz, 1H), 2.02 (dd, J = 13.9, 7.9 Hz, 1H), 1.99-1.86 (m, 3H), 1.75 (dd, J = 6.3, 1.3 Hz, 3H), 1.48-1.31 (m, 2H), 1.25 (s, 3H), 0.97 ppm (s, 3H). ^{13}C NMR (101 MHz, $CDCl_3$) δ 161.6, 136.4, 134.7, 134.7, 133.4, 127.5, 122.5, 122.1, 119.5, 119.0, 113.5, 111.3, 108.2, 65.6, 63.2, 61.9, 60.2, 53.1, 50.6, 49.0, 48.2, 44.6, 37.7, 32.9, 26.7, 24.6, 22.0, 20.5, 20.1 ppm; HRMS calcd. For $C_{29}H_{35}ClIN_3NaO_5S_2^+$ $[M + Na]^+$ 754.0644, found 754.0620.

3. Experimental Procedures and Characterisation Data (Intermolecular TDO / indole cycloadditions)

General Procedure

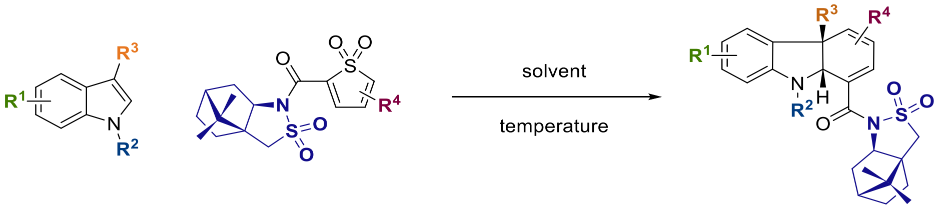

To a stirred solution of indole (0.2 mmol) in CH_2Cl_2 (0.1 M) at 0 °C was added thiophene *S,S*-dioxide (0.1 mmol). The resulting mixture was stirred for 38 h before it was concentrated under reduced pressure to afford the crude material, which was purified by flash column chromatography. Yields stated are based on thiophene *S,S*-dioxide.

Some substrates required the use of chloroform as a solvent and/or higher reaction temperature, which is noted below for those specific cases. Stereochemistry was assigned by analogy to the cycloaddition of thiophene S,S-dioxide ***9d*** *with* ***17****, which was converted to the (–)-series of natural products.*

((4a*S*,9a*R*)-4-Chloro-4a,9a-dihydro-9*H*-carbazol-1-yl)((3a*S*,6*R*,7a*R*)-8,8-dimethyl-2,2-dioxidotetrahydro-3*H*-3a,6-methanobenzo[*c*]isothiazol-1(4*H*)-yl)methanone, 10a

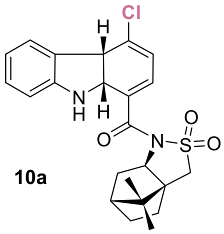

10a

Flash column chromatography (silica gel, pentane:EtOAc 20:1→4:1) afforded compound **10a** (39.2 mg, 88.1 μmol, 88%) as a yellow foam. **10a**: R_f = 0.37 (silica gel, pentane:EtOAc 4:1); $[\alpha]_D^{25}$ = –267 (*c* = 1.00, $CHCl_3$); IR (film) ν_{max} 3377, 3055, 3000, 2960, 1670, 1577, 1332, 1284, 737 cm^{-1}; ^1H NMR (500 MHz, $CDCl_3$): δ 7.45 (d, *J* = 7.5 Hz, 1H), 7.08 (td, *J* = 7.6, 1.2 Hz, 1H), 6.81 (d, *J* = 6.6 Hz, 1H), 6.79-6.74 (m, 1H), 6.67 (d, *J* = 7.8 Hz, 1H), 6.24 (dd, *J* = 6.6, 1.7 Hz, 1H), 5.20 (d, *J* = 11.4 Hz, 1H), 4.24 (d, *J* = 11.4 Hz, 1H), 4.14 (dd, *J* = 7.7, 4.3 Hz, 1H), 3.51 (d, *J* = 13.6 Hz, 1H), 3.40 (d, *J* = 13.7 Hz, 1H), 2.04 (dd, *J* = 13.2, 7.8 Hz, 1H), 2.00-1.88 (m, 4H), 1.48-1.35 (m, 2H), 1.23 (s, 3H), 0.99 ppm (s, 3H); ^{13}C NMR (101 MHz, $CDCl_3$): δ 169.9, 149.4, 141.8, 133.8, 128.6, 128.2, 127.2, 126.5, 120.0, 119.3, 110.2, 65.6, 58.5, 53.8, 48.2, 48.1, 47.9, 45.3, 38.3, 33.3, 26.7, 21.4, 20.0 ppm; HRMS calcd. For $C_{23}H_{26}ClN_2O_3S^+$ $[M + H]^+$ 445.1347, found 445.1338.

((4a*S*,9a*R*)-4-Chloro-8-methyl-4a,9a-dihydro-9*H*-carbazol-1-yl)((3a*S*,6*R*,7a*R*)-8,8-dimethyl-2,2-dioxidotetrahydro-3*H*-3a,6-methanobenzo[*c*]isothiazol-1(4*H*)-yl)methanone, 10b

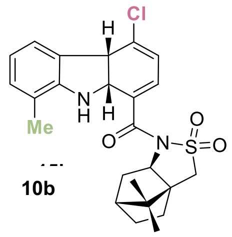

10b

Flash column chromatography (silica gel, pentane:EtOAc 20:1→4:1) afforded compound **10b** (41.9 mg, 91.3 µmol, 91%) as a yellow foam. **10b**: R_f = 0.51 (silica gel, pentane:EtOAc 4:1); $[\alpha]_D^{25}$ = –260 (c = 1.00, $CHCl_3$); IR (film) ν_{max} 3390, 3076, 2993, 2960, 1671, 1580, 1333, 1273, 737 cm^{-1}; ^{1}H NMR (400 MHz, $CDCl_3$): δ 7.32 (d, J = 7.5 Hz, 1H), 6.92 (d, J = 7.4 Hz, 1H), 6.78 (d, J = 6.6 Hz, 1H), 6.71 (t, J = 7.5 Hz, 1H), 6.22 (dd, J = 6.6, 1.7 Hz, 1H), 5.24 (d, J = 11.6 Hz, 1H), 4.28 (d, J = 11.6 Hz, 1H), 4.15 (dd, J = 7.7, 4.3 Hz, 1H), 3.99 (d, J = 14.5 Hz, 1H), 3.51 (d, J = 13.6 Hz, 1H), 3.40 (d, J = 13.7 Hz, 1H), 2.11 (s, 3H), 2.05 (dd, J = 13.2, 7.7 Hz, 1H), 2.01-1.86 (m, 4H), 1.50-1.33 (m, 2H), 1.23 (s, 3H), 0.99 ppm (s, 3H); ^{13}C NMR (101 MHz, $CDCl_3$): δ 169.9, 148.0, 141.9, 133.5, 129.4, 128.1, 126.6, 123.8, 119.8, 119.7, 119.3, 65.5, 58.2, 53.7, 48.4, 48.2, 47.9, 45.2, 38.2, 33.2, 26.6, 21.3, 19.9, 16.9 ppm; HRMS calcd. For $C_{24}H_{28}ClN_2O_3S^+$ [M + H]$^+$ 459.1504, found 459.1494.

((4a*S*,9a*R*)-4-Chloro-6-(4,4,5,5-tetramethyl-1,3,2-dioxaborolan-2-yl)-4a,9a-dihydro-9*H*-carbazol-1-yl)((3a*S*,6*R*,7a*R*)-8,8-dimethyl-2,2-dioxidotetrahydro-3*H*-3a,6-methanobenzo[*c*]isothiazol-1(4*H*)-yl)methanone, 10c

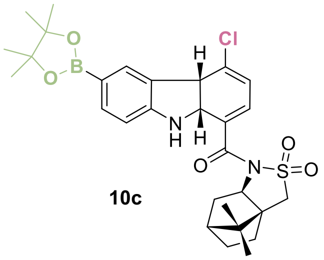

10c

Flash column chromatography (silica gel, pentane:EtOAc 10:1→2:1) afforded compound **10c** (48.2 mg, 84.4 µmol, 84%) as a yellow foam. **10c**: R_f = 0.18 (silica gel, pentane:EtOAc 4:1); $[\alpha]_D^{25}$ = –258 (c = 1.00, $CHCl_3$); IR (film) ν_{max} 3402, 3056, 2991, 2979, 1672, 1606, 1354, 1273, 737 cm^{-1}; ^{1}H NMR (400 MHz, $CDCl_3$): δ 7.86 (s, 1H), 7.56 (d, J = 7.9 Hz, 1H), 6.81 (d, J = 6.6 Hz, 1H), 6.61 (d, J = 7.8 Hz, 1H), 6.23 (dd, J = 6.6, 1.6 Hz, 1H), 5.19 (dd, J = 11.4, 1.6 Hz, 1H), 4.37 (s, 1H), 4.22 (d, J = 11.3 Hz, 1H), 4.13 (dd, J = 7.7, 3.8 Hz, 1H), 3.50 (d, J = 13.7 Hz, 1H), 3.39 (d, J = 13.7 Hz, 1H), 2.08-1.85 (m, 5H), 1.48-1.35 (m, 2H), 1.32 (d, J = 2.3 Hz, 12H), 1.23 (s, 3H), 0.99 ppm (s, 3H); ^{13}C NMR (101 MHz, $CDCl_3$): δ 169.9, 152.2, 142.0, 136.3, 134.1, 132.8, 127.9, 126.3, 119.9, 108.9, 83.4, 65.6, 58.6, 53.8, 48.2, 47.9, 47.6, 45.3, 38.2, 33.3, 26.7, 25.1, 25.0, 21.4, 20.0 ppm; HRMS calcd. For $C_{29}H_{37}BClN_2O_5S^+$ [M + H]$^+$ 571.2199, found 571.2191.

((4a*S*,9a*R*)-4-Chloro-8-iodo-4a,9a-dihydro-9*H*-carbazol-1-yl)((3a*S*,6*R*,7a*R*)-8,8-dimethyl-2,2-dioxidotetrahydro-3*H*-3a,6-methanobenzo[*c*]isothiazol-1(4*H*)-yl)methanone, 10d

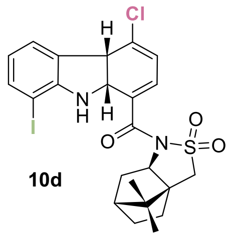

10d

Flash column chromatography (silica gel, pentane:EtOAc 20:1→5:1) afforded compound **10d** (53.3 mg, 93.4 μmol, 93%) as a yellow foam. **10d**: R_f = 0.60 (silica gel, pentane:EtOAc 3:1); $[\alpha]_D^{25}$ = –167 (*c* = 1.00, $CHCl_3$); IR (film) ν_{max} 3383, 3060, 3001, 2960, 1671, 1598, 1374, 1274, 737 cm^{-1}; ^1H NMR (400 MHz, $CDCl_3$): δ 7.40 (dd, *J* = 9.7, 7.6 Hz, 2H), 6.78 (d, *J* = 6.6 Hz, 1H), 6.48 (t, *J* = 7.7 Hz, 1H), 6.25 (dd, *J* = 6.6, 1.7 Hz, 1H), 5.24 (dd, *J* = 11.5, 2.1 Hz, 1H), 4.38 (d, *J* = 12.0 Hz, 2H), 4.15 (dd, *J* = 7.7, 4.3 Hz, 1H), 3.51 (d, *J* = 13.7 Hz, 1H), 3.44 (d, *J* = 13.7 Hz, 1H), 2.06 (dd, *J* = 13.2, 7.8 Hz, 1H), 2.02-1.85 (m, 4H), 1.51-1.34 (m, 2H), 1.24 (s, 3H), 1.00 ppm (s, 3H); ^{13}C NMR (101 MHz, $CDCl_3$): δ 169.8, 151.3, 140.9, 137.1, 133.6, 127.7, 126.6, 126.2, 120.5, 120.4, 75.2, 65.5, 57.4, 53.8, 49.4, 48.3, 47.9, 45.3, 38.3, 33.3, 26.7, 21.4, 20.0 ppm; HRMS calcd. For $C_{23}H_{25}ClIN_2O_3S^+$ $[M + H]^+$ 571.0314, found 571.0303.

((4a*S*,9a*R*)-4-Chloro-6-nitro-4a,9a-dihydro-9*H*-carbazol-1-yl)((3a*S*,6*R*,7a*R*)-8,8-dimethyl-2,2-dioxidotetrahydro-3*H*-3a,6-methanobenzo[*c*]isothiazol-1(4*H*)-yl)methanone, 10e

(Reaction was performed at room temperature)

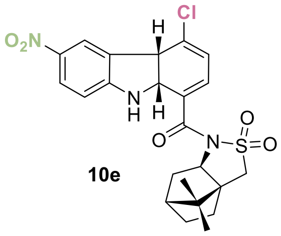

10e

Flash column chromatography (silica gel, pentane:EtOAc 20:1→5:1) afforded compound **10e** (33.6 mg, 68.6 μmol, 69%) as a yellow foam. **10e**: R_f = 0.30 (silica gel, pentane:EtOAc 3:1); $[\alpha]_D^{25}$ = –205 (*c* = 1.00, $CHCl_3$); IR (film) ν_{max} 3375, 3061, 2999, 2960, 1672, 1580, 1326, 1264, 737 cm^{-1}; ^1H NMR (400 MHz, $CDCl_3$): δ 8.32 (dt, *J* = 1.9, 0.8 Hz, 1H), 8.06 (dd, *J* = 8.7, 2.3 Hz, 1H), 6.82 (d, *J* = 6.6 Hz, 1H), 6.53 (d, *J* = 8.8 Hz, 1H), 6.29 (dd, *J* = 6.6, 1.6 Hz, 1H), 5.38 (dd, *J* = 11.8, 1.2 Hz, 1H), 4.91 (s, 1H), 4.32 (dt, *J* = 11.9, 1.5 Hz, 1H), 4.12 (dd, *J* = 7.8, 4.0 Hz, 1H), 3.52 (d, *J* = 13.7 Hz, 1H), 3.40 (d, *J* = 13.7 Hz, 1H), 2.10-1.86 (m, 5H), 1.50-1.35 (m, 2H), 1.23 (s, 3H), 1.00 ppm (s, 3H); ^{13}C NMR (101 MHz, $CDCl_3$): δ 169.7, 154.8, 140.1, 139.7, 134.1, 127.3, 127.0, 126.8, 122.9, 120.5, 107.2, 65.5, 59.2, 53.9, 48.3, 48.0, 46.8, 45.3, 38.2, 33.3, 26.7, 21.3, 20.0 ppm; HRMS calcd. For $C_{23}H_{25}ClN_3O_5S^+$ $[M + H]^+$ 490.1198, found 490.1192.

(4b*S*,8a*R*)-5-Chloro-8-((3a*S*,6*R*,7a*R*)-8,8-dimethyl-2,2-dioxidohexahydro-3*H*-3a,6-methanobenzo[*c*]isothiazole-1-carbonyl)-4b,8a-dihydro-9*H*-carbazole-3-carbonitrile, 10f

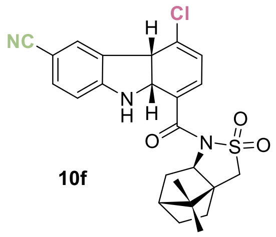

10f

Flash column chromatography (silica gel, pentane:EtOAc 10:1→2:1) afforded compound **10f** (34.1 mg, 72.6 μmol, 73%) as a yellow foam. **10f**: R_f = 0.38 (silica gel, pentane:EtOAc 2:1); $[\alpha]_D^{25}$ = –206 (c = 1.00, $CHCl_3$); IR (film) ν_{max} 3400, 3005, 2960, 1669, 1578, 1336, 1245, 737 cm^{-1}; ^1H NMR (400 MHz, $CDCl_3$): δ 7.67 (d, J = 1.4 Hz, 1H), 7.36 (dd, J = 8.2, 1.7 Hz, 1H), 6.80 (d, J = 6.6 Hz, 1H), 6.57 (d, J = 8.2 Hz, 1H), 6.27 (dd, J = 6.6, 1.6 Hz, 1H), 5.30 (d, J = 11.8 Hz, 1H), 4.69 (s, 1H), 4.27 (d, J = 11.7 Hz, 1H), 4.12 (dd, J = 7.8, 4.0 Hz, 1H), 3.52 (d, J = 13.5 Hz, 1H), 3.39 (d, J = 13.7 Hz, 1H), 2.08-1.87 (m, 5H), 1.49-1.35 (m, 2H), 1.22 (s, 3H), 1.00 ppm (s, 3H); ^{13}C NMR (126 MHz, $CDCl_3$): δ 169.8, 153.0, 140.2, 134.1, 133.9, 130.0, 127.6, 127.2, 120.5, 108.9, 100.5, 65.5, 58.7, 53.8, 48.3, 48.0, 47.1, 45.3, 38.2, 33.3, 26.7, 21.3, 20.0 ppm; HRMS calcd. For $C_{24}H_{25}ClN_3O_3S^+$ $[M + H]^+$ 470.1300, found 470.1293.

((4a*S*,9a*R*)-4-Chloro-7-fluoro-4a,9a-dihydro-9*H*-carbazol-1-yl)((3a*S*,6*R*,7a*R*)-8,8-dimethyl-2,2-dioxidotetrahydro-3*H*-3a,6-methanobenzo[*c*]isothiazol-1(4*H*)-yl)methanone, 10g

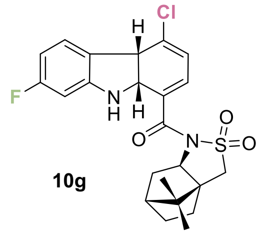

10g

Flash column chromatography (silica gel, pentane:EtOAc 15:1→4:1) afforded compound **10g** (40.4 mg, 87.3 μmol, 87%) as a yellow foam. **10g**: R_f = 0.43 (silica gel, pentane:EtOAc 4:1); $[\alpha]_D^{25}$ = –228 (c = 1.00, $CHCl_3$); IR (film) ν_{max} 3400, 2992, 2961, 1671, 1579, 1457, 1285, 738 cm^{-1}; ^1H NMR (500 MHz, $CDCl_3$): δ 7.34 (ddd, J = 8.3, 5.6, 1.0 Hz, 1H), 6.81 (d, J = 6.5 Hz, 1H), 6.41 (ddd, J = 9.4, 8.2, 2.4 Hz, 1H), 6.33 (dd, J = 9.7, 2.3 Hz, 1H), 6.24 (dd, J = 6.6, 1.6 Hz, 1H), 5.23 (dd, J = 11.4, 1.9 Hz, 1H), 4.34-4.23 (m, 1H), 4.18 (dd, J = 11.5, 1.5 Hz, 1H), 4.13 (dd, J = 7.8, 3.9 Hz, 1H), 3.51 (d, J = 13.6 Hz, 1H), 3.41 (d, J = 13.7 Hz, 1H), 2.09-1.90 (m, 5H), 1.50-1.34 (m, 2H), 1.23 (s, 3H), 1.00 ppm (s, 3H); ^{13}C NMR (126 MHz, $CDCl_3$): δ 169.82, 164.87, 162.94, 151.0 (d, J = 12.0 Hz), 141.6, 133.9, 127.8, 127.1 (d, J = 10.5 Hz), 122.5 (d, J = 2.3 Hz), 120.0, 105.4 (d, J = 22.9 Hz), 97.6 (d, J = 26.4 Hz), 65.6, 59.1, 53.8, 48.1 (d, J = 39.7 Hz), 47.2, 45.3, 38.2, 33.3, 26.7, 21.4, 20.0 ppm; ^{19}F NMR (376 MHz, $CDCl_3$): δ -114.4 ppm; HRMS calcd. For $C_{23}H_{25}ClFN_2O_3S^+$ $[M + H]^+$ 463.1253, found 463.1246.

((4a*S*,9a*R*)-4-Chloro-5-fluoro-4a,9a-dihydro-9*H*-carbazol-1-yl)((3a*S*,6*R*,7a*R*)-8,8-dimethyl-2,2-dioxidotetrahydro-3*H*-3a,6-methanobenzo[*c*]isothiazol-1(4*H*)-yl)methanone, 10h

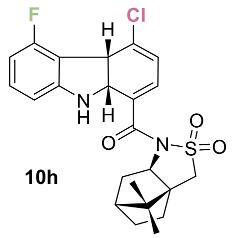

10h

Flash column chromatography (silica gel, pentane:EtOAc 15:1→4:1) afforded compound **10h** (36.5 mg, 78.8 μmol, 79%) as a yellow foam. **10h**: R_f = 0.46 (silica gel, pentane:EtOAc 3:1); $[\alpha]_D^{25}$ = –437 (*c* = 0.25, $CHCl_3$); IR (film) ν_{max} 2992, 2962, 1672, 1575, 1469, 1283, 763 cm^{-1}; ^{1}H NMR (500 MHz, $CDCl_3$): δ 7.04 (td, *J* = 8.0, 5.5 Hz, 1H), 6.87 (d, *J* = 6.5 Hz, 1H), 6.48 (t, *J* = 8.7 Hz, 1H), 6.44 (d, *J* = 7.8 Hz, 1H), 6.33 (dd, *J* = 6.5, 2.2 Hz, 1H), 5.01 (dd, *J* = 10.0, 3.2 Hz, 1H), 4.36 (dd, *J* = 10.0, 2.1 Hz, 1H), 4.33-4.28 (m, 1H), 4.13 (dd, *J* = 7.8, 4.4 Hz, 1H), 3.54 (d, *J* = 13.7 Hz, 1H), 3.43 (d, *J* = 13.7 Hz, 1H), 2.04 (dd, *J* = 13.3, 7.8 Hz, 1H), 2.01-1.87 (m, 4H), 1.48-1.33 (m, 2H), 1.24 (s, 3H), 1.00 ppm (s, 3H); ^{13}C NMR (126 MHz, $CDCl_3$): δ 169.6, 161.8, 159.8, 152.3 (d, *J* = 7.4 Hz), 141.3, 134.5, 130.4 (d, *J* = 8.9 Hz), 126.8, 121.1, 112.7 (d, *J* = 19.1 Hz), 106.8 (d, *J* = 21.6 Hz), 105.8 (d, *J* = 2.8 Hz), 65.6, 58.6, 53.8, 48.1 (d, *J* = 39.1 Hz), 45.6 (d, *J* = 2.2 Hz), 45.3, 38.2, 33.3, 26.7, 21.3, 20.0 ppm; ^{19}F NMR (376 MHz, $CDCl_3$): δ -114.4 ppm; HRMS calcd. For $C_{23}H_{25}ClFN_2O_3S^+$ $[M + H]^+$ 463.1253, found 463.1246.

Methyl (4b*S*,8a*R*)-5-chloro-8-((3a*S*,6*R*,7a*R*)-8,8-dimethyl-2,2-dioxidohexahydro-3*H*-3a,6-methanobenzo[*c*]isothiazole-1-carbonyl)-4b,8a-dihydro-9*H*-carbazole-2-carboxylate, 10i

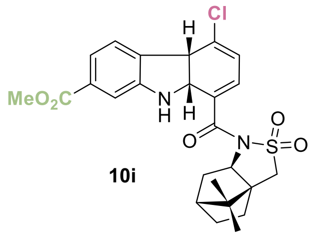

10i

(Reaction was performed at room temperature)

Flash column chromatography (silica gel, pentane:EtOAc 10:1→3:1) afforded compound **10i** (41.1 mg, 81.7 μmol, 82%) as a yellow foam. **10i**: R_f = 0.38 (silica gel, pentane:EtOAc 4:1); $[\alpha]_D^{25}$ = –250 (*c* = 1.00, $CHCl_3$); IR (film) ν_{max} 3408, 3004, 2958, 1718, 1669, 1579, 1336, 1286, 736 cm^{-1}; ^{1}H NMR (400 MHz, $CDCl_3$): δ 7.50-7.43 (m, 2H), 7.27 (d, *J* = 1.4 Hz, 1H), 6.80 (d, *J* = 6.6 Hz, 1H), 6.26 (dd, *J* = 6.6, 1.6 Hz, 1H), 5.25 (d, *J* = 11.5 Hz, 1H), 4.27 (dt, *J* = 11.5, 1.1 Hz, 1H), 4.17-4.09 (m, 1H), 3.86 (s, 3H), 3.50 (d, *J* = 13.7 Hz, 1H), 3.40 (d, *J* = 13.7 Hz, 1H), 2.08-1.85 (m, 5H), 1.49-1.34 (m, 2H), 1.22 (s, 3H), 0.99 ppm (s, 3H); ^{13}C NMR (101 MHz, $CDCl_3$): δ 169.8, 167.4, 149.6, 140.5, 133.6, 132.4, 130.7, 128.1, 126.0, 121.0, 120.5, 110.4, 65.6, 58.8, 53.8, 52.1, 48.3, 48.0, 47.9, 45.3, 38.3, 33.3, 26.7, 21.4, 20.0 ppm; HRMS calcd. For $C_{25}H_{28}ClN_2O_5S^+$ $[M + H]^+$ 503.1402, found 503.1395.

((4a*S*,9a*R*)-4-Chloro-7-methoxy-4a,9a-dihydro-9*H*-carbazol-1-yl)((3a*S*,6*R*,7a*R*)-8,8-dimethyl-2,2-dioxidotetrahydro-3*H*-3a,6-methanobenzo[*c*]isothiazol-1(4*H*)-yl)methanone, 10j

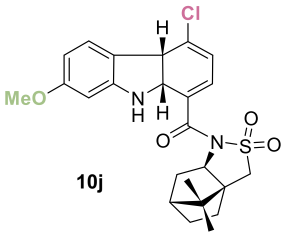

Flash column chromatography (silica gel, pentane:EtOAc 15:1→4:1) afforded compound **10j** (43.7 mg, 92.0 µmol, 92%) as a yellow foam. **10j**: R_f = 0.50 (silica gel, pentane:EtOAc 3:1); $[\alpha]_D^{25}$ = –322 (*c* = 1.00, $CHCl_3$); IR (film) ν_{max} 3383, 2994, 2958, 1670, 1581, 1452, 1291, 735 cm^{-1}; ^{1}H NMR (400 MHz, $CDCl_3$): δ 7.33 (dd, *J* = 8.3, 1.0 Hz, 1H), 6.81 (d, *J* = 6.8 Hz, 1H), 6.31 (dd, *J* = 8.3, 2.3 Hz, 1H), 6.24 (d, *J* = 2.4 Hz, 1H), 6.21 (dd, *J* = 6.6, 1.8 Hz, 1H), 5.19 (d, *J* = 11.4 Hz, 1H), 4.27-4.06 (m, 3H), 3.73 (s, 3H), 3.52 (d, *J* = 13.7 Hz, 1H), 3.41 (d, *J* = 13.7 Hz, 1H), 2.04 (dd, *J* = 12.8, 7.7 Hz, 1H), 2.00-1.85 (m, 4H), 1.50-1.35 (m, 2H), 1.23 (s, 3H), 1.00 ppm (s, 3H); ^{13}C NMR (101 MHz, $CDCl_3$): δ 169.9, 160.8, 150.7, 142.4, 133.9, 127.9, 127.0, 119.7, 119.4, 104.6, 96.7, 65.6, 58.7, 55.5, 53.8, 48.2, 47.9, 47.3, 45.3, 38.3, 33.3, 26.7, 21.4, 20.0 ppm; HRMS calcd. For $C_{24}H_{28}ClN_2O_4S^+$ [M + H]$^+$ 475.1453, found 475.1446.

((4a*S*,9a*R*)-4-Chloro-6,7-dimethoxy-4a,9a-dihydro-9*H*-carbazol-1-yl)((3a*S*,6*R*,7a*R*)-8,8-dimethyl-2,2-dioxidotetrahydro-3*H*-3a,6-methanobenzo[*c*]isothiazol-1(4*H*)-yl)methanone, 10k

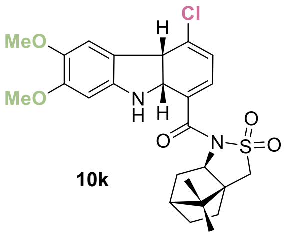

Flash column chromatography (silica gel, pentane:EtOAc 15:1→1:1) afforded compound **10k** (41.7 mg, 82.6 µmol, 83%) as a yellow foam. **10k**: R_f = 0.60 (silica gel, pentane:EtOAc 1:1); $[\alpha]_D^{25}$ = –288 (*c* = 1.00, $CHCl_3$); IR (film) ν_{max} 3392, 2993, 2960, 1664, 1579, 1444, 1285, 748 cm^{-1}; ^{1}H NMR (400 MHz, $CDCl_3$): δ 7.06 (d, *J* = 0.8 Hz, 1H), 6.78 (d, *J* = 6.6 Hz, 1H), 6.37 (s, 1H), 6.20 (dd, *J* = 6.6, 1.8 Hz, 1H), 5.18 (d, *J* = 11.6 Hz, 1H), 4.18 (dd, *J* = 11.6, 1.8 Hz, 1H), 4.13 (dd, *J* = 7.7, 4.3 Hz, 1H), 3.83 (s, 3H), 3.79 (s, 3H), 3.51 (d, *J* = 13.6 Hz, 1H), 3.41 (d, *J* = 13.7 Hz, 1H), 2.04 (dd, *J* = 13.1, 7.7 Hz, 1H), 2.01-1.84 (m, 4H), 1.47-1.33 (m, 2H), 1.23 (s, 3H), 0.99 ppm (s, 3H); ^{13}C NMR (101 MHz, $CDCl_3$): δ 169.7, 150.0, 143.6, 143.2, 142.0, 133.5, 128.1, 119.7, 118.4, 111.4, 96.7, 65.7, 58.7, 57.1, 56.1, 53.8, 48.4, 48.2, 47.9, 45.3, 38.3, 33.3, 26.6, 21.4, 20.0 ppm; HRMS calcd. For $C_{25}H_{30}ClN_2O_5S^+$ [M + H]$^+$ 505.1559, found 505.1548.

((4a*S*,9a*R*)-4-Chloro-9-methyl-4a,9a-dihydro-9*H*-carbazol-1-yl)((3a*S*,6*R*,7a*R*)-8,8-dimethyl-2,2-dioxidotetrahydro-3*H*-3a,6-methanobenzo[*c*]isothiazol-1(4*H*)-yl)methanone, 10l

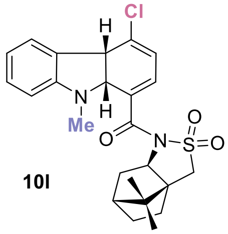

10l

Flash column chromatography (silica gel, pentane:EtOAc 20:1→5:1) afforded compound **10l** (36.1 mg, 78.6 μmol, 79%) as a yellow foam. **10l**: R_f = 0.50 (silica gel, pentane:EtOAc 5:1); $[\alpha]_D^{25}$ = –461 (*c* = 1.00, $CHCl_3$); IR (film) ν_{max} 3094, 2992, 2960, 1668, 1573, 1484, 1288, 738 cm^{-1}; ^1H NMR (400 MHz, $CDCl_3$): δ 7.45 (d, J = 7.4 Hz, 1H), 7.18 (td, J = 7.7, 1.3 Hz, 1H), 7.14 (d, J = 6.5 Hz, 1H), 6.78 (td, J = 7.4, 1.0 Hz, 1H), 6.56 (d, J = 7.8 Hz, 1H), 6.34 (dd, J = 6.5, 2.3 Hz, 1H), 4.66-4.57 (m, 1H), 4.22-4.11 (m, 2H), 3.54 (d, J = 13.7 Hz, 1H), 3.46 (d, J = 13.7 Hz, 1H), 2.69 (s, 3H), 2.07 (dd, J = 12.8, 7.8 Hz, 1H), 2.01-1.91 (m, 4H), 1.56-1.45 (m, 1H), 1.44-1.34 (m, 1H), 1.25 (s, 3H), 1.01 ppm (s, 3H); ^{13}C NMR (101 MHz, $CDCl_3$): δ 169.8, 152.1, 142.9, 137.2, 128.8, 127.6, 126.7, 126.6, 120.8, 118.7, 108.1, 66.1, 64.7, 53.8, 48.2, 48.0, 47.0, 45.1, 38.3, 34.9, 33.2, 26.8, 21.3, 20.0 ppm; HRMS calcd. For $C_{24}H_{28}ClN_2O_3S^+$ $[M + H]^+$ 459.1504, found 459.1495.

((4a*S*,9a*R*)-9-Benzyl-4-chloro-4a,9a-dihydro-9*H*-carbazol-1-yl)((3a*S*,6*R*,7a*R*)-8,8-dimethyl-2,2-dioxidotetrahydro-3*H*-3a,6-methanobenzo[*c*]isothiazol-1(4*H*)-yl)methanone, 10m

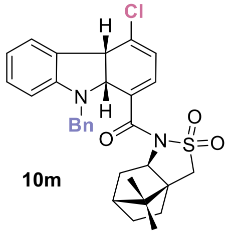

10m

Flash column chromatography (silica gel, pentane:EtOAc 20:1→5:1) afforded compound **10m** (36.4 mg, 68.0 μmol, 68%) as a brown foam. **10m**: R_f = 0.53 (silica gel, pentane:EtOAc 4:1); $[\alpha]_D^{25}$ = –338 (*c* = 1.00, $CHCl_3$); IR (film) ν_{max} 3083, 3006, 2960, 1667, 1571, 1483, 1337, 1273, 1242, 740 cm^{-1}; ^1H NMR (400 MHz, $CDCl_3$): δ 7.43 (d, *J* = 7.4 Hz, 1H), 7.25 (s, 3H), 7.22-7.18 (m, 1H), 7.11 (d, *J* = 6.6 Hz, 1H), 7.00 (td, *J* = 7.8, 1.4 Hz, 1H), 6.72 (td, *J* = 7.5, 1.0 Hz, 1H), 6.38 (dd, *J* = 6.6, 2.1 Hz, 1H), 6.30 (d, *J* = 8.0 Hz, 1H), 5.03 (d, *J* = 10.2 Hz, 1H), 4.48 (d, *J* = 15.9 Hz, 1H), 4.16 (dd, *J* = 10.2, 2.1 Hz, 1H), 4.10-4.02 (m, 2H), 3.44 (d, *J* = 13.7 Hz, 1H), 3.37 (d, *J* = 13.7 Hz, 1H), 1.98 (dd, *J* = 13.3, 7.8 Hz, 1H), 1.93-1.77 (m, 4H), 1.47-1.38 (m, 1H), 1.37-1.29 (m, 1H), 1.14 (s, 3H), 0.96 ppm (s, 3H); ^{13}C NMR (101 MHz, $CDCl_3$): δ 169.6, 151.3, 142.7, 138.4, 137.2, 128.7, 128.4, 127.8, 127.4, 126.9, 126.7, 126.5, 120.8, 118.3, 108.4, 65.8, 62.5, 53.5, 50.9, 48.2, 47.9, 47.3, 45.0, 38.1, 33.0, 26.7, 21.1, 20.0 ppm; HRMS calcd. For $C_{30}H_{32}ClN_2O_3S^+$ $[M + H]^+$ 535.1817, found 535.1810.

((3a*S*,6*R*,7a*R*)-8,8-Dimethyl-2,2-dioxidotetrahydro-3*H*-3a,6-methanobenzo[*c*]isothiazol-1(4*H*)-yl)(5-methylthiophen-2-yl)methanone, S25

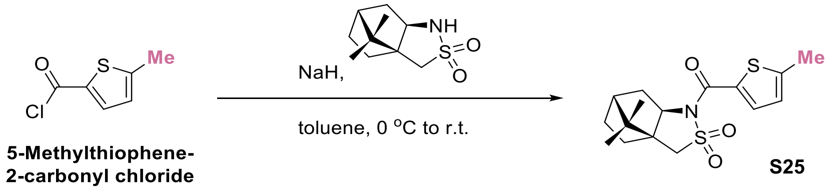

To a stirred solution of (1*S*)-(–)-2,10-camphorsultam (137 mg, 0.64 mmol, 1.0 equiv.) in toluene (6.5 mL) at 0 °C was added NaH (60% in oil, 38.4 mg, 0.96 mmol, 1.5 equiv.). The resulting mixture was stirred for 1 h. To the resulting mixture at 0 °C was added 5-Methylthiophene-2-carbonyl chloride (204 mg, 1.27 mmol, 2.0 equiv.). The resulting mixture was warmed to room temperature and stirred for 14 h before it was quenched slowly with water (10 mL). The layers were separated and the aqueous layer was extracted with Et_2O (3 × 10 mL), the combined organic layers were washed with water (30 mL), brine (30 mL), dried (Na_2SO_4) concentrated under reduced pressure. Flash column chromatography (silica gel, pentane:EtOAc 15:1→4:1) afforded compound **S25** (354 mg, 1.04 mmol, 82%) as a yellow flaky solid.

S25: R_f = 0.20 (silica gel, pentane:EtOAc 6:1); $[\alpha]_D^{25}$ = –113 (*c* = 0.43, $CHCl_3$); IR (film) ν_{max} 3005, 2958, 1657, 1458, 1336, 1295, 768 cm^{-1}; ^1H NMR (400 MHz, $CDCl_3$): δ 7.87 (d, *J* = 3.8 Hz, 1H), 6.78 (dq, *J* = 3.9, 1.0 Hz, 1H), 4.22 (dd, *J* = 7.6, 4.7 Hz, 1H), 3.56 (d, *J* = 13.6 Hz, 1H), 3.46 (d, *J* = 13.6 Hz, 1H), 2.52 (d, *J* = 1.0 Hz, 3H), 2.08 (dd, *J* = 13.6, 7.6 Hz, 1H), 2.04-1.85 (m, 4H), 1.51-1.35 (m, 2H), 1.29 (s, 3H), 1.01 ppm (s, 3H); ^{13}C NMR (101 MHz, $CDCl_3$): δ 162.3, 149.7, 135.3, 134.5, 126.7, 66.5, 53.8, 48.2, 47.9, 45.4, 38.5, 33.4, 26.7, 21.4, 20.1, 16.0 ppm; HRMS calcd. For $C_{16}H_{22}NO_3S_2^+$ $[M + H]^+$ 340.1036, found 340.1032.

((3a*S*,6*R*,7a*R*)-8,8-Dimethyl-2,2-dioxidotetrahydro-3*H*-3a,6-methanobenzo[*c*]isothiazol-1(4*H*)-yl)(5-methyl-1,1-dioxidothiophen-2-yl)methanone, 9b

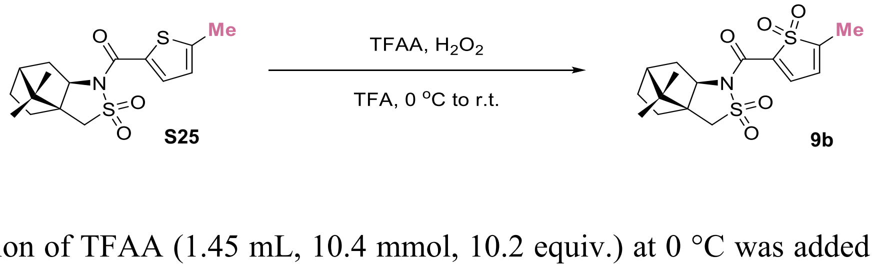

To a stirred solution of TFAA (1.45 mL, 10.4 mmol, 10.2 equiv.) at 0 °C was added H_2O_2 (30 wt% in H_2O, 0.37 mL, 3.64 mmol, 3.5 equiv.) dropwise. The resulting mixture was warmed to room temperature

and stirred for 15 min. To the resulting mixture at 0 °C was added **S25** (354 mg, 1.04 mmol, 1.0 equiv.). The resulting mixture was warmed to room temperature and stirred for 14 h before it was concentrated under reduced pressure. The residue was washed with hot pentane (30 mL) to afford compound **9b** (302 mg, 0.81 mmol, 78%) as a bright yellow solid. **9b**: R_f = 0.18 (silica gel, pentane:EtOAc 3:1); $[\alpha]_D^{25}$ = –111 (*c* = 0.14, $CHCl_3$); IR (film) ν_{max} 2993, 2947, 1669, 1340, 1316, 749 cm^{-1}; ^1H NMR (400 MHz, $CDCl_3$): δ 7.78 (d, *J* = 4.8 Hz, 1H), 6.48 (dt, *J* = 5.3, 1.9 Hz, 1H), 4.12 (dd, *J* = 7.6, 4.8 Hz, 1H), 3.55 (d, *J* = 13.7 Hz, 1H), 3.49 (d, *J* = 13.7 Hz, 1H), 2.18 (d, *J* = 2.0 Hz, 3H), 2.09 (dd, *J* = 13.9, 7.9 Hz, 1H), 2.00-1.82 (m, 4H), 1.48-1.34 (m, 2H), 1.17 (s, 3H), 0.98 ppm (s, 3H); ^{13}C NMR (101 MHz, $CDCl_3$): δ 157.6, 146.2, 136.5, 133.6, 120.6, 66.2, 53.6, 48.5, 47.8, 45.1, 38.4, 33.1, 26.4, 21.2, 19.9, 10.0 ppm; HRMS calcd. For $C_{16}H_{22}NO_5S_2^+$ $[M + H]^+$ 372.0934, found 372.0929.

((3a*S*,6*R*,7a*R*)-8,8-Dimethyl-2,2-dioxidotetrahydro-3*H*-3a,6-methanobenzo[*c*]isothiazol-1(4*H*)-yl)((5a*R*,10b*S*)-1,2,3,5a,6,10b-hexahydrocyclopenta[*c*]carbazol-5-yl)methanone, 10n

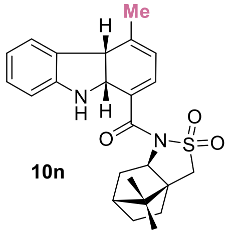

10n

Flash column chromatography (silica gel, pentane:EtOAc 13:1→1:1) afforded compound **10n** (29.0 mg, 68.3 μmol, 68%) as a yellow foam. **10n**: R_f = 0.55 (silica gel, pentane:EtOAc 2:1); $[\alpha]_D^{25}$ = –351 (*c* = 0.39, $CHCl_3$); IR (film) ν_{max} 2991, 2958, 1667, 1643, 1465, 1333, 1286, 1240, 737 cm^{-1}; ^1H NMR (400 MHz, $CDCl_3$): δ 7.21 (d, *J* = 7.4 Hz, 1H), 7.03 (t, *J* = 7.6 Hz, 1H), 6.92 (d, *J* = 6.1 Hz, 1H), 6.72 (t, *J* = 7.4 Hz, 1H), 6.64 (d, *J* = 7.8 Hz, 1H), 5.93 (dt, *J* = 6.1, 1.8 Hz, 1H), 5.04 (d, *J* = 10.8 Hz, 1H), 4.15 (dd, *J* = 7.7, 4.2 Hz, 1H), 3.91 (d, *J* = 10.9 Hz, 1H), 3.50 (d, *J* = 13.6 Hz, 1H), 3.39 (d, *J* = 13.7 Hz, 1H), 2.03 (dd, *J* = 13.0, 7.7 Hz, 1H), 1.98-1.86 (m, 7H), 1.48-1.33 (m, 2H), 1.25 (s, 3H), 0.99 ppm (s, 3H); ^{13}C NMR (101 MHz, $CDCl_3$): δ 170.6, 149.7, 144.3, 136.4, 129.1, 127.9, 127.0, 125.6, 118.9, 118.4, 110.1, 65.6, 57.0, 53.8, 48.1, 47.9, 46.8, 45.3, 38.2, 33.3, 26.7, 22.5, 21.4, 20.0 ppm; HRMS calcd. For $C_{24}H_{29}N_2O_3S^+$ $[M + H]^+$ 425.1893, found 425.1886.

(5,6-Dihydro-4*H*-cyclopenta[*b*]thiophen-2-yl)((3a*S*,6*R*,7a*R*)-8,8-dimethyl-2,2-dioxidotetrahydro-3*H*-3a,6-methanobenzo[*c*]isothiazol-1(4*H*)-yl)methanone, S26

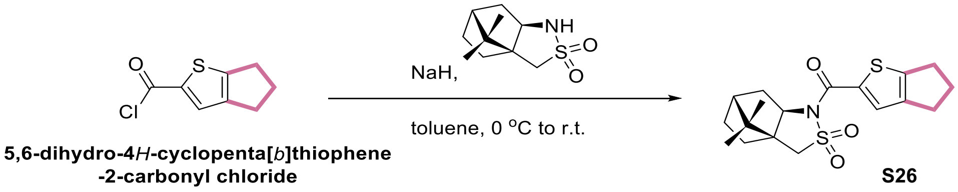

To a stirred solution of (1*S*)-(–)-2,10-camphorsultam (123 mg, 0.57 mmol, 1.0 equiv.) in toluene (5.7 mL) at 0 °C was added NaH (60% in oil, 34.2 mg, 0.86 mmol, 1.5 equiv.). The resulting mixture was stirred for 1 h. To the resulting mixture at 0 °C was added 5,6-dihydro-4H-cyclopenta[*b*]thiophene-2-carbonyl chloride (213 mg, 1.14 mmol, 2.0 equiv.). The resulting mixture was warmed to room temperature and stirred for 14 h before it was quenched slowly with water (10 mL). The layers were separated and the aqueous layer was extracted with Et_2O (3 × 10 mL), the combined organic layers were washed with water (30 mL), brine (30 mL), dried (Na_2SO_4) concentrated under reduced pressure. Flash column chromatography (silica gel, pentane:EtOAc 15:1→4:1) afforded compound **S26** (350 mg, 0.96 mmol, 84%) as a tan flaky solid. **S26**: R_f = 0.55 (silica gel, pentane:EtOAc 4:1); $[\alpha]_D^{25}$ = –116 (*c* = 0.33, $CHCl_3$); IR (film) ν_{max} 2960, 1653, 1476, 1403, 1332, 1293, 761 cm^{-1}; ^1H NMR (500 MHz, $CDCl_3$): δ 7.80 (s, 1H), 4.24 (dd, *J* = 7.7, 4.7 Hz, 1H), 3.56 (d, *J* = 13.7 Hz, 1H), 3.47 (d, *J* = 13.6 Hz, 1H), 2.92 (t, *J* = 7.2 Hz, 2H), 2.80 (dt, *J* = 14.8, 7.3 Hz, 1H), 2.72 (dt, *J* = 14.8, 7.2 Hz, 1H), 2.50-2.39 (m, 2H), 2.07 (dd, *J* = 13.7, 7.7 Hz, 1H), 2.04-1.94 (m, 2H), 1.94-1.87 (m, 2H), 1.49-1.36 (m, 2H), 1.30 (s, 3H), 1.01 ppm (s, 3H); ^{13}C NMR (126 MHz, $CDCl_3$): δ 162.5, 153.0, 147.6, 139.2, 130.4, 66.6, 53.9, 48.2, 47.9, 45.4, 38.5, 33.4, 29.7, 29.3, 28.3, 26.7, 21.4, 20.1 ppm; HRMS calcd. For $C_{18}H_{24}NO_3S_2^+$ [M + H]$^+$ 366.1192, found 366.1187.

((3a*S*,6*R*,7a*R*)-8,8-dimethyl-2,2-dioxidotetrahydro-3*H*-3a,6-methanobenzo[*c*]isothiazol-1(4*H*)-yl)(1,1-dioxido-5,6-dihydro-4*H*-cyclopenta[*b*]thiophen-2-yl)methanone, 9c

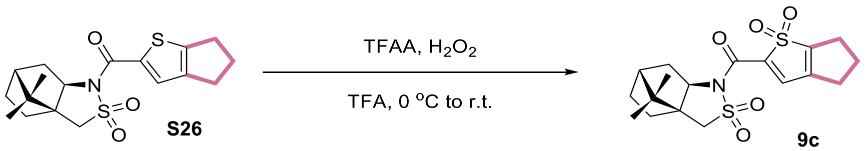

To a stirred solution of TFAA (1.33 mL, 9.58 mmol, 10.0 equiv.) at 0 °C was added H_2O_2 (30 wt% in H_2O, 0.34 mL, 3.35 mmol, 3.5 equiv.) dropwise. The resulting mixture was warmed to room temperature and stirred for 15 min. To the resulting mixture at 0 °C was added **S26** (350 mg, 0.96 mmol, 1.0 equiv.). The resulting mixture was warmed to room temperature and stirred for 14 h before it was concentrated under reduced pressure. Flash column chromatography (silica gel, pentane:EtOAc 10:1→2:1) afforded compound **9c** (282 mg, 0.71 mmol, 74%) as a white foam. **9c**: R_f = 0.18 (silica gel, pentane:EtOAc 3:1);

$[\alpha]_D^{25}$ = –70.2 (*c* = 0.60, $CHCl_3$); IR (film) ν_{max} 2962, 1668, 1561, 1315, 1298, 736 cm^{-1}; ^1H NMR (500 MHz, $CDCl_3$): δ 7.74 (s, 1H), 4.15 (dd, *J* = 7.8, 4.8 Hz, 1H), 3.57 (d, *J* = 13.7 Hz, 1H), 3.50 (d, *J* = 13.7 Hz, 1H), 2.83-2.76 (m, 2H), 2.73-2.59 (m, 2H), 2.43 (p, *J* = 7.9 Hz, 2H), 2.12 (dd, *J* = 13.9, 7.7 Hz, 1H), 2.08-2.02 (m, 1H), 1.98-1.87 (m, 4H), 1.48-1.35 (m, 2H), 1.20 (s, 3H), 1.00 ppm (s, 3H); ^{13}C NMR (126 MHz, $CDCl_3$): δ 157.7, 147.2, 146.5, 139.4, 133.4, 66.5, 53.8, 48.6, 48.0, 45.2, 38.6, 33.4, 29.4, 26.8, 26.7, 26.5, 21.4, 20.0 ppm; HRMS calcd. For $C_{18}H_{24}NO_5S_2^+$ $[M + H]^+$ 398.1090, found 398.1085.

((3a*S*,6*R*,7a*R*)-8,8-Dimethyl-2,2-dioxidotetrahydro-3*H*-3a,6-methanobenzo[*c*]isothiazol-1(4H)-yl)((5a*R*,10b*S*)-1,2,3,5a,6,10b-hexahydrocyclopenta[*c*]carbazol-5-yl)methanone, 10o

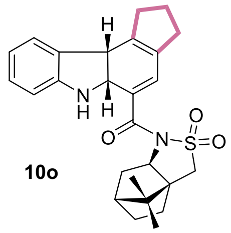

10o

Flash column chromatography (silica gel, pentane:EtOAc 15:1→3:1) afforded compound **10o** (30.5 mg, 67.7 μmol, 68%) as a yellow foam. **10o**: R_f = 0.57 (silica gel, pentane:EtOAc 3:1); $[\alpha]_D^{25}$ = –179 (*c* = 0.31, $CHCl_3$); IR (film) ν_{max} 2987, 2963, 1671, 1642, 1484, 1333, 1285, 1242, 742 cm^{-1}; ^1H NMR (400 MHz, $CDCl_3$): δ 7.17 (d, *J* = 7.3 Hz, 1H), 7.01 (t, *J* = 7.6 Hz, 1H), 6.97 (s, 1H), 6.71 (t, *J* = 7.4 Hz, 1H), 6.64 (d, *J* = 7.8 Hz, 1H), 5.15 (d, *J* = 11.2 Hz, 1H), 4.22-4.12 (m, 2H), 4.07 (d, *J* = 11.3 Hz, 1H), 3.52 (d, *J* = 13.6 Hz, 1H), 3.42 (d, *J* = 13.6 Hz, 1H), 2.63-2.53 (m, 1H), 2.46 (t, *J* = 7.8 Hz, 3H), 2.05 (dd, *J* = 13.2, 7.7 Hz, 1H), 2.00-1.84 (tt, *J* = 16.8, 6.3 Hz, 6H), 1.50-1.34 (m, 2H), 1.27 (s, 3H), 1.00 ppm (s, 3H); ^{13}C NMR (101 MHz, $CDCl_3$): δ 170.7, 149.5, 145.5, 135.6, 131.4, 129.1, 128.1, 127.8, 125.3, 119.0, 110.1, 65.7, 57.0, 53.9, 48.1, 47.9, 45.4, 44.3, 38.3, 34.0, 33.4, 26.8, 22.3, 21.5, 20.1 ppm; HRMS calcd. For $C_{26}H_{31}N_2O_3S^+$ $[M + H]^+$ 451.2050, found 451.2040.

((4a*S*,9a*R*)-3-Bromo-4a,9a-dihydro-9*H*-carbazol-1-yl)((3a*S*,6*R*,7a*R*)-8,8-dimethyl-2,2-dioxidotetrahydro-3*H*-3a,6-methanobenzo[*c*]isothiazol-1(4*H*)-yl)methanone, 10p

(Crude thiophene *S,*S-dioxide **9d** used for the cycloaddition)

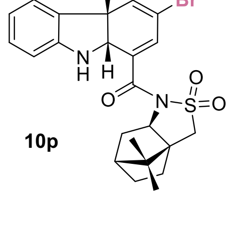

10p

Flash column chromatography (silica gel, pentane:EtOAc 15:1→3:1) afforded compound **10p** (26.6 mg, 54.3 μmol, 54%) as a dark yellow foam. **10p**: R_f = 0.57 (silica gel, pentane:EtOAc 3:1); $[\alpha]_D^{25}$ = –245 (*c* = 0.52, $CHCl_3$); IR (film) ν_{max} 3009, 2959, 1671, 1631, 1484, 1335, 1289, 1244, 763 cm^{-1}; ^1H NMR (500 MHz, $CDCl_3$): δ 7.14 (d, *J* = 7.4 Hz, 1H), 7.04 (t, *J* = 7.6 Hz, 1H), 6.79 (br s, 1H), 6.75 (t, *J* = 7.4 Hz, 1H), 6.67 (d, *J* = 7.8 Hz, 1H), 6.30 (dd, *J* = 3.9, 1.3 Hz,

1H), 5.05 (d, J = 11.7 Hz, 1H), 4.25 (dd, J = 11.7, 3.9 Hz, 1H), 4.15 (s, 1H), 4.11 (dd, J = 7.8, 4.5 Hz, 1H), 3.53 (d, J = 13.7 Hz, 1H), 3.42 (d, J = 13.7 Hz, 1H), 2.06 (dd, J = 13.6, 7.8 Hz, 1H), 2.00-1.89 (m, 4H), 1.48-1.35 (m, 2H), 1.24 (s, 3H), 1.00 ppm (s, 3H); ^{13}C NMR (126 MHz, $CDCl_3$): δ 169.1, 149.5, 137.0, 133.5, 130.7, 128.7, 128.3, 124.4, 119.7, 113.7, 110.8, 65.6, 53.8, 53.7, 48.3, 48.0, 45.2, 45.0, 38.3, 33.3, 26.7, 21.4, 20.0 ppm; HRMS calcd. For $C_{23}H_{26}BrN_2O_3S^+$ $[M + H]^+$ 489.0842, found 489.0833.

***tert*-Butyl (2-((4a*S*,9a*S*)-4-chloro-1-((3a*S*,6*R*,7a*R*)-8,8-dimethyl-2,2-dioxidohexahydro-3*H*-3a,6-methanobenzo[*c*]isothiazole-1-carbonyl)-9,9a-dihydro-4a*H*-carbazol-4a-yl)ethyl)carbamate, 10q**

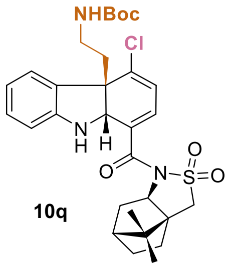

10q

(Reaction was performed in $CHCl_3$ at 80 °C)

Flash column chromatography (silica gel, pentane:EtOAc 5:1→3:1) afforded compound **10q** (52.9 mg, 0.09 mmol, 90%) as a yellow foam. **10q**: R_f = 0.41 (silica gel, pentane:EtOAc 3:1); $[\alpha]_D^{25}$ = –285 (c = 0.31, $CHCl_3$); IR (film) ν_{max} 3372, 2970, 1706, 1670, 1332, 1282, 1247, 1168, 742 cm^{-1}; ^{1}H NMR (400 MHz, $CDCl_3$): δ 7.35 (dd, J = 7.7, 1.1 Hz, 1H), 7.07 (td, J = 7.6, 1.2 Hz, 1H), 6.79 (dd, J = 7.0, 5.4 Hz, 2H), 6.65 (d, J = 7.8 Hz, 1H), 6.34 (d, J = 6.5 Hz, 1H), 4.86 (s, 1H), 4.61 (br s, 1H), 4.18 (br s, 1H), 4.13 (dd, J = 7.3, 4.9 Hz, 1H), 3.55 (d, J = 13.6 Hz, 1H), 3.43 (d, J = 13.6 Hz, 1H), 3.22 (br s, 1H), 3.14-3.03 (m, 1H), 2.53-2.42 (m, 1H), 2.09-1.84 (m, 6H), 1.43 (s, 11H), 1.25 (s, 3H), 1.01 ppm (s, 3H); ^{13}C NMR (101 MHz, $CDCl_3$): δ 169.4, 155.8, 149.4, 144.6, 133.4, 129.9, 128.4, 127.3, 126.4, 121.5, 119.3, 110.1, 79.2, 65.7, 62.7, 53.8, 53.5, 48.2, 47.9, 45.3, 38.3, 37.9, 35.8, 33.2, 28.5, 26.6, 21.4, 19.9 ppm; HRMS calcd. For $C_{30}H_{39}ClN_3O_5S^+$ $[M + H]^+$ 588.2275, found 588.2294.

***N*-(2-((4aS,9aS)-4-Chloro-1-((3a*S*,6*R*,7a*R*)-8,8-dimethyl-2,2-dioxidohexahydro-3*H*-3a,6-methanobenzo[*c*]isothiazole-1-carbonyl)-6-methoxy-9,9a-dihydro-4a*H*-carbazol-4a-yl)ethyl)acetamide, 10r**

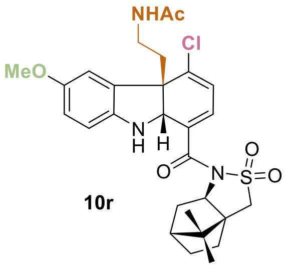

10r

(Reaction was performed in $CHCl_3$ at 80 °C)

Flash column chromatography (silica gel, pentane:EtOAc 1:1→1:3) afforded compound **10r** (50.9 mg, 0.09 mmol, 91%) as a red foam. **10r**: R_f = 0.29 (silica gel, EtOAc); $[\alpha]_D^{25}$ = –366 (c = 0.33, $CHCl_3$); IR (film) ν_{max} 3334, 2997, 1664, 1490, 1332, 1275, 742 cm^{-1}; ^{1}H NMR (400 MHz, $CDCl_3$): δ 6.92 (d, J = 2.1 Hz, 1H), 6.79 (d, J = 6.4 Hz, 1H), 6.66 (d, J = 2.2 Hz, 2H), 6.36 (d, J = 6.4 Hz, 1H), 5.98 (br s, 1H), 4.82 (s, 1H), 4.12 (dd, J = 7.5, 4.7 Hz, 1H), 3.92 (br s, 1H), 3.76 (s,

3H), 3.57 (d, *J* = 13.7 Hz, 1H), 3.50 (dd, *J* = 13.8, 6.7 Hz, 1H), 3.45 (d, *J* = 13.6 Hz, 1H), 3.08 (dq, *J* = 13.0, 6.5 Hz, 1H), 2.46 (dt, *J* = 14.7, 6.3 Hz, 1H), 2.10-1.96 (m, 3H), 1.96-1.87 (m, 6H), 1.48-1.35 (m, 2H), 1.26 (s, 3H), 1.01 ppm (s, 3H) ; ^{13}C NMR (101 MHz, $CDCl_3$): δ 170.2, 169.8, 154.1, 144.3, 143.2, 133.5, 132.2, 127.3, 121.7, 113.7, 112.9, 111.5, 65.7, 63.2, 56.1, 54.4, 53.7, 48.2, 47.8, 45.3, 38.3, 36.9, 34.8, 33.3, 26.5, 23.2, 21.4, 19.9 ppm; HRMS calcd. For $C_{28}H_{34}ClN_3O_5SK^+$ $[M + K]^+$ 598.1529, found 598.1539.

4. Computational Details

4.1.1 Electronic Structure Calculations

Quantum chemical calculations were run using the ORCA suite of programs (version 5.0.3 and 6.0).[13,14] All calculations used the resolution of the identity approximation (RIJCOSX),[15]with the appropriate auxiliary basis sets.[16] “VeryTight” optimisation criteria (10^{-9} Ha tolerance for SCF, $2 \cdot 10^{-7}$ Ha tolerance for optimisation step) were employed using the default grid.

Geometry optimisations were carried out using the Minnesota DFT-functional M06-2X[17] and double-hybrid functional B2PLYP,[18] both of which have been shown to provide reliable predictions of geometries, rotational spectroscopic parameters, and vibrational properties.[19-22] The def2-TZVP/C and def2-SVP/C correlation integrals were used as auxiliary basis set for the def2-TZVP and def2-SVP basis sets, respectively.[23]

Initial conformers for minima were identified using the software package CREST[24] and optimised using GFN2-xTB in combination with the GBSA(MeCN) solvent model[25] implemented in xTB.[26] Relevant conformers were reoptimised at CPCM(MeCN)-M06-2X/def2-SVP[27] and CPCM(MeCN)-B2PLYP-D3BJ/def2-SVP[28] levels of theory. The Domain-based Local Pair Natural Orbital coupled cluster method with singles, doubles and perturbative triples (DLPNO-CCSD(T)) was used as a final single point providing reliable electronic energies using the def2-TZVP basis set.[23] DLPNO-CCSD(T) calculations were run using “NormalPNO” cut-offs.[29]

Vibrational frequencies were computed at the optimisation level of theory to confirm whether the structures correspond to minima or transition states. All intermediate structures were verified to be minima by the absence of imaginary frequencies upon calculation of the Hessian. Grimme’s quasi RRHO approach was used to calculate free energies at 353 K.[30] A standard state correction from 1 atm to 1 M was applied by adding RTln(1/24.5) (T = 353 K) to the calculated free energy of each species. For calculating thermodynamic data, the python-script *OTherm.py* was used with ω_0 = 100 cm^{-1}, replacing harmonic oscillators with free-rotors below ω_0.[30,31]

Throughout this analysis, the vinyl iodide substituent of **30** was found not to influence the conformational state of the rest of the molecule (see Table S15 for **30**, Appendix), with various minima corresponding simply to different rotameric forms of this sidechain. Therefore, this vinyl iodide sidechain was then simplified to a Me group (i.e., corresponding to model compound **29** to reduce computational cost, (Figure S1 and Figure 3a in the main text).

The 13 lowest energy conformers of **29**, representing each of the features were reoptimised at the CPCM(MeCN)-M06-2X/def2-SVP level of theory, with 'conformer **2**' (corresponding to **29** in main text, see Table S12) identified as the lowest-energy conformer at both CPCM(MeCN)-M06-2X/def2-SVP and at CPCM(MeCN)-M06-2X/def2-TZVP//CPCM(MeCN)-M06-2X/def2-SVP level of theory.

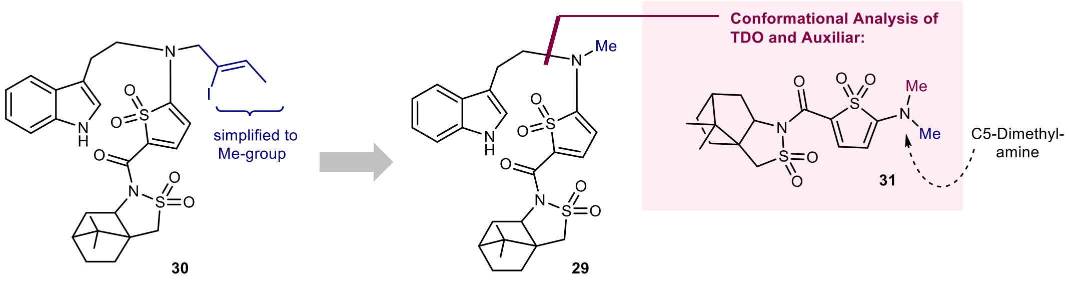

Figure S1. Overview of the structure used during the computational analysis

4.1.2 Training of Machine Learning Interatomic Potential (MLIP): To investigate the conformational flexibility of the model TDO system (**31**), as well as the mechanism of the intramolecular Diels Alder reaction and the subsequent SO_2 extrusion step, connecting conformation **29** and the product **Inter2** (main text Figure 3a), we trained a machine learning interatomic potential (MLIP).

The MLIP was trained using the MACE architecture,[32] a graph neural network-based MLIP, where the chemical environments are described using the atomic cluster expansion (ACE) descriptor. The ground-truth method employed was The CPCM (MeCN)-B2PLYP-D3BJ/def2-SVP. Training was conducted using MACE v0.3.4[32,33] within the *mlp-train* package.[34] An on-the-fly training strategy combining active learning (AL) and well-tempered metadynamics (WTMetaD)[35] was employed using the averaged bond length of the two C-C bond being formed ($\bar{r} = \frac{1}{2} \times (r_1^{CC} + r_2^{CC})$) as the collective variable. Hyperparameters of MACE, selectors and WTMetaD bias, used in training MACE MLIPs are listed in Table S11. The idea behind the AL strategy is to systematically improve the performance of the MLIP as new data is collected (Figure S2a). The initial MLIP is built using configurations generated by random displacement of atomic coordinates; in this case, starting from **TS5** with the opposite enantiomer of the auxiliary from which five configurations were generated (Figure S2b). This first MLIP is then used to propagate dynamics and collect new data points, which are selected if their kernel similarity vector, derived from the Smooth Overlap of Atomic Positions (SOAP) descriptor,[36] to the existing training data is below 0.9993. For more details on the AL strategy and selector, readers are referred to Ref. 33.

In this case, the initial MLIP was used to propagate five independent molecular dynamics (MLIP-MD) simulations in the NVT ensemble at 353 K with WTMetaD bias using the PLUMED plugin,[37,38] integrated with the Atomic Simulation Environment (ASE) v3.23.0b1.[39] These simulations employed Langevin dynamics with a friction coefficient of 0.02 in atomic units and a timestep of 0.5 fs. Velocities were initialised using the Maxwell-Boltzmann distribution at the corresponding temperature. During the AL workflow, 432 configurations were collected, with 389 configurations randomly selected for training and the remaining 43 used for validation. Additionally, 16 configurations of a simplified TDO (**31**, shown in Figure S2c), with dihedral angles α and β spanning the range α = –140° to 130° and β = 0 ° to 270, were included in the training set to improve conformer space representation (Figure S1). The validation set revealed an energy error of 0.93 kcal mol^{-1} and a force error of 0.77 kcal mol^{-1} compared to the ground truth.

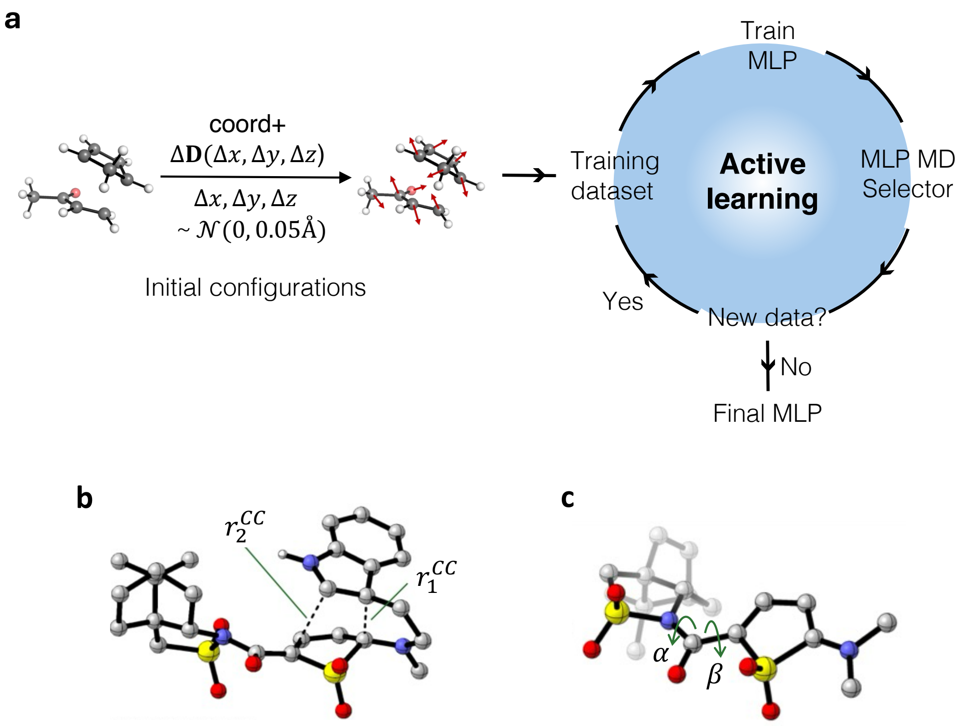

Figure S2. **a.** Active learning (AL) strategy used to train the machine learning interatomic potential (MLIP), modified from Figure 1a in Ref. 33. **b**. Starting configuration used for AL, corresponding to **TS5** with the opposite enantiomer of the auxiliary optimised at the CPCM (MeCN)-B2PLYP-D3BJ/def2-SVP level of theory; the C-C bonds used within the collective variable $\bar{r} = \frac{1}{2} \times (r_1^{CC} + r_2^{CC})$ for WTMetaD simulations are highlighted in dashed black lines. **c.** simplified TDO geometry (**31)**, featuring a C5-dimethylamine substituent, used to generate the 16 additional configurations for MLIP training. These configurations were generated by varying the dihedral angles α and β, covering the range α = –140º to 130º and β = 0º to 270º.

Table S11. Hyperparameter setting for MACE potential, selectors and WTMetaD in training MLIP.

Type	Parameter	Description	Value
MACE	ν	Maximum correlation order	3
	r_{cut}	Cut-off radius	5.0 Å
	hidden_irrps	Irreducible representations	128x0e+128x1o
	batch_size	Batch size in training potential	5
	valid_fraction	Fraction of the dataset used for validation during the training	0.1
SOAP descriptor	σ_{at}^{SOAP}	Spread of the Gaussian added to atomic density	1.0 Å
	n_{max}, l_{max}	The maximum number and degree for the radial (n) and angular (l) basis	6
	r_{cut}	Cut-off radius	5.0 Å

similarity selector	k_T	Selection threshold	0.9993
WTMetaD	σ	Width, standard deviation of the placed Gaussian	0.05
	τ_G	Pace, interval at which a new Gaussian is placed	10 fs
	ω	Height, initial height of placed Gaussian	$5k_BT$
	γ	Bias factor, describe how quickly Gaussian shrink	70

4.2. Results

4.2.1 Validation of the DFT level of theory

Note: This segment of the study was conducted with the opposite enantiomer of the auxiliary. This is inconsequential, as two opposite enantiomers possess the same electronic properties and energies.

We evaluated the influence of both basis set and DFT functional on the geometries and energies of the ground state conformer **30** and its associated transition state. For the ground state, increasing the basis set from def2-SVP to def2-TZVP resulted in a minor heavy-atom root mean square deviation (RMSD) change of 0.24 Å and a slight increase in the hydrogen bond distance between sultam and indole from 2.09 Å to 2.35 Å for def2-SVP to def2-TZV basis sets (Figure S3).

Using the def2-TZVP basis set, we evaluated various DFT functionals, including PBE0-D3BJ (GGA hybrid), M06-2X (meta-GGA), and B2PLYP (double hybrid, with and without dispersion correction D3BJ), were evaluated. The heavy-atom RMSD differences across these functional were negligible (0.10 Å, 0.10 Å and 0.04 Å for PBE0-D3BJ, M06-2X and B2PLYP relative to B2PLYP-D3BJ). However, the hydrogen bond distances between sultam and indole varied more significantly, especially when considering or not dispersion, yielding distances of 2.36 Å, 2.35 Å, 2.13 Å, and 2.74 Å, for PBE0-D3BJ, M06-2X, B2PLYP-D3BJ and B2PLYP, respectively. The transition state had a heavy-atom RMSD of 0.34 Å between B2PLYP-D3BJ and M06-2X, with larger discrepancies in the aforementioned H-bond distance, 2.4 and 4.6 Å, respectively. The generally small RMSD difference between these functionals and basis sets led us to use M06-2X with the def2-TZVP basis set for initial geometry optimisation and pre-filtering of (transition state) conformers.

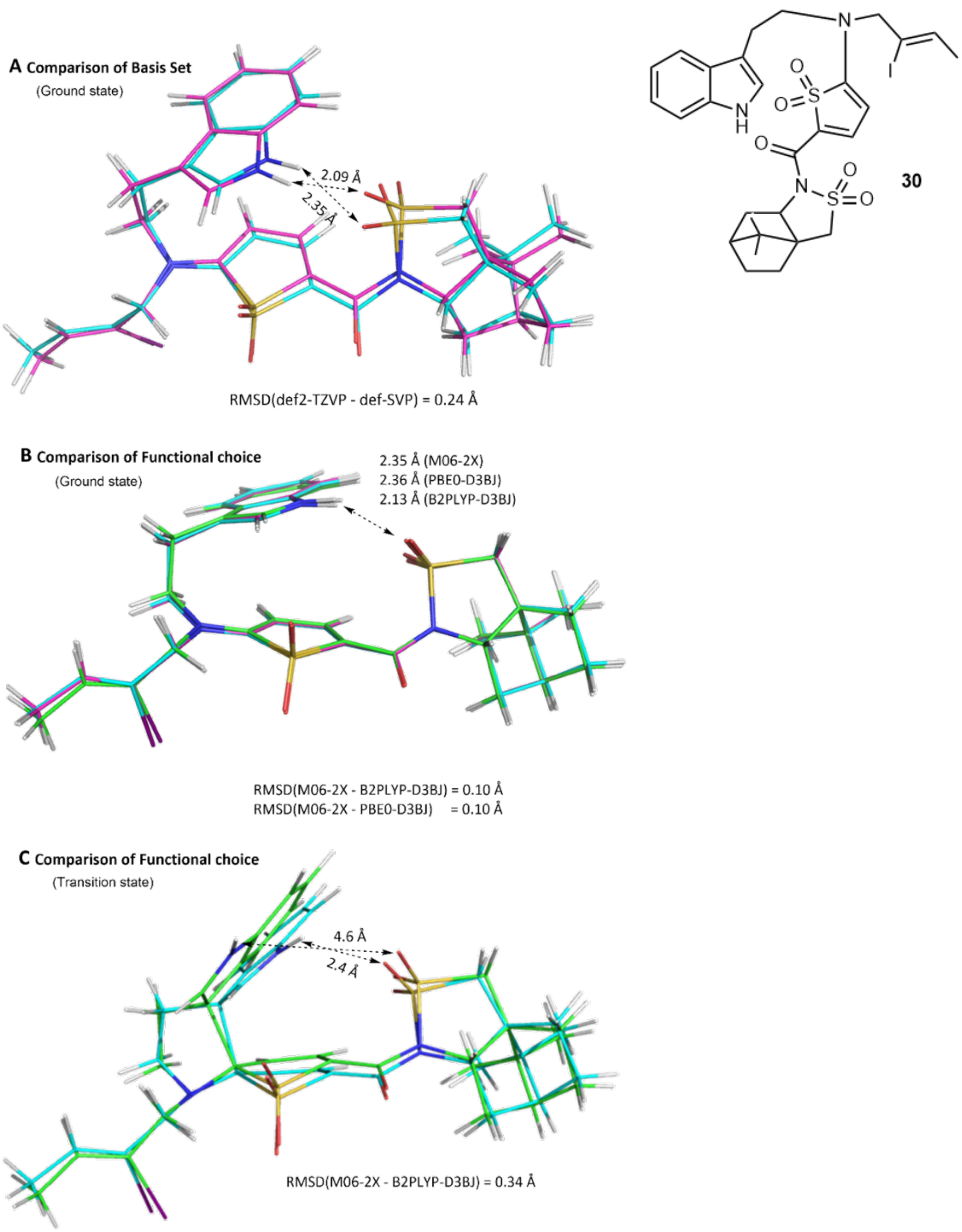

Figure S3. Effect of Basis set and functional on geometry. Effect of basis set on the ground state geometry of **30**. Geometries optimised at the CPCM(MeCN)-M06-2X/def2-SVP (pink structure) and CPCM(MeCN)-M06-2X/def2-TZVP (blue structure) level of theory. **b**. Comparison of different DFT functionals on the ground state geometry of **30** computed at the CPCM(MeCN)-**Functional**/def2-TZVP, where **Functional** corresponds to PBE0-D3BJ (pink structure), M06-2X (green structure), and B2PLYP-D3BJ (blue structure) functionals. **c**. Overlay of TS optimised geometries at the CPCM(MeCN)-M06-2X/def2-SVP (blue structure) and CPCM(MeCN)-B2PLYP-D3BJ/def2-SVP (green structure) level of theory. Structures were aligned using the *align* command in PyMOL 3.0, followed by heavy-atom RMSD calculations.

4.3. Conformational sampling

Conformational sampling of **30** using CREST/xtB with the GBSA(MeCN) solvent model resulted in 320 unique conformers, which were manually categorised based on features ① to ④, as depicted in Figure S4 and Table S12 – accounting for the orientation of the indole attack (*exo*- or *endo*) and the different orientations of the amide and sultam bicycle along the z-axis. CREST/xtB yielded only 13 of the 16 (2^4) possible conformers, as some fell outside the 6 kcal mol^{-1} selection window (Table S12).

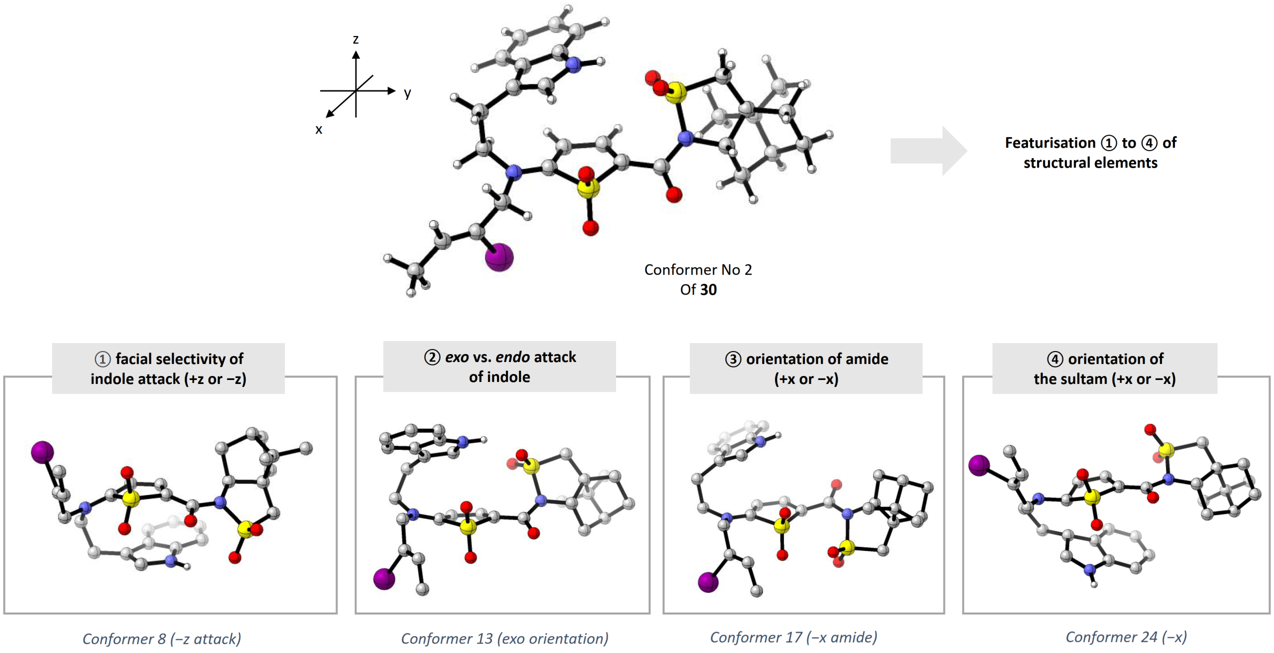

Figure S4. Relevant features of the diastereoselectivity of cycloaddition of **30**: ① The indole can attack from -z or +z direction, dictating the facial selectivity, in an *exo*- or *endo*-fashion ②, relative to the indole. Further, the amide ③, as well as the sultam ④, can be oriented to the front (+x) or back (-x).

4.3.1 Lowest lying conformer

Throughout this analysis, the vinyl iodide substituent of **30** was found not to influence the conformational state of the rest of the molecule (see Table S15 for **30**, Appendix), with various minima corresponding simply to different rotameric forms of this sidechain. Therefore, this vinyl iodide sidechain was then simplified to a Me group corresponding to model compound **29** to reduce computational cost (Figure S1 and Figure 5a in the main text).

The 13 lowest energy conformers of **29**, representing each of the features were reoptimised at the CPCM(MeCN)-M06-2X/def2-SVP level of theory, with 'conformer **2**' (corresponding to **29** in main text, see Table S12) identified as the lowest-energy conformer at both CPCM(MeCN)-M06-2X/def2-SVP and at CPCM(MeCN)-M06-2X/def2-TZVP//CPCM(MeCN)-M06-2X/def2-SVP level of theory.

Table S12. Re-optimised conformers of **30** generated by CREST and classified based on features ① to ④ as described in **Figure S4**. Entries with same features but different energy values are rotamers of vinyl iodide sidechain, e.g. entry No. 1-5 in Table S15 (Appendix).

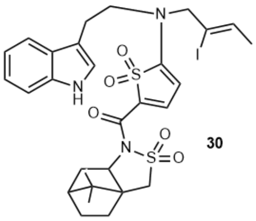

30

CREST rank	1) face of indole attack	2) endo/exo	3) Amide	4) Sultam	E_{el} at M06-2X/ def2-SVP [Ha]	Relative E_{el} [kcal mol^{-1}]
2	+z	*endo*	+x	-x	-2757.61698	0.0
6	+z	*endo*	-x	+x	-2757.60397	8.2
18	+z	*exo*	+x	-x	-2757.61205	3.1
15	-z	*endo*	+x	+x	-2757.60518	7.4
60	-z	*endo*	+x	-x	-2757.61054	4.0
253	-z	*endo*	-x	+x	-2757.59357	14.7
301	-z	*endo*	-x	-x	-2757.60773	5.8
34	-z	*endo*	-x	+x	-2757.60628	6.7
100	-z	*endo*	-x	-x	-2757.60861	5.3
148	-z	*endo*	+x	-x	-2757.60009	10.6
28	-z	*exo*	+x	-x	-2757.61088	3.8
81	-z	*exo*	-x	+x	-2757.60481	7.6
29		*Unoriented conformer*			-2757.60425	8.0

Model thiophene *S,S*-dioxides (TDOs) conformation: 29 was further simplified to analyse in more detail the conformational preference of the camphorsultam group relative to the TDO ring, beside the CREST-suggested lowest lying **29**.

Using the simplified TDO **31**, featuring a C5-dimethylamine substituent (Figure S1), a 2D energy profile was constructed as a function of the torsion angles around the (TDO)C–C(=O)(β) and C(=O)–N(sultam)(α) bonds. For efficient sampling of the PES, we used the MLIP trained at the CPCM(MeCN)-B2PLYP-D3BJ/def2-SVP level of theory. In line with the CREST-sampling, Conformer **A** was identified as the lowest energy state, followed by conformer **B** (ΔG = 0.7 kcal mol^{-1}). In conformer **A**, the C2 carbonyl group is oriented *syn* to the TDO sulfone, with the C=O bond bisecting the two S=O bonds. This arrangement allows for coplanarity between the carbonyl and the C5-dimethylamine, with the sultam N–S bond positioned *anti*- to the carbonyl group to minimize steric clash with the TDO ring.

Natural bond orbital (NBO) analysis conducted at CPCM(MeCN)-B2PLYP-D3BJ/def2-SVP level indicated that although conformer **B** exhibits a stronger stabilisation due to the donation of the nitrogen lone pair to the C=O π^* orbital, with second order perturbation theory interaction energies (E(2)) of 46.3 and 31.0 kcal mol^{-1} for conformer **B** and conformer **A**, respectively (Table S13, highlighted in light blue**)**, conformers **B** is destabilised by steric clashes between the bicyclic sultam auxiliary moiety and the thiophene aromatic core. This steric hindrance is absent in conformer **A**, resulting in similar energy between both conformers.

Further, two additional local minima were identified on the MLP-PES of **31**: Conformer **C** and **D**. Both are significantly less stabilised than Conformer A, ΔG = 4.6 kcal mol^{-1} and ΔG = 3.6 kcal mol$^{-1,}$ respectively. This is due to the conjugaition across the amide and sultam is less prevalent in those structures.

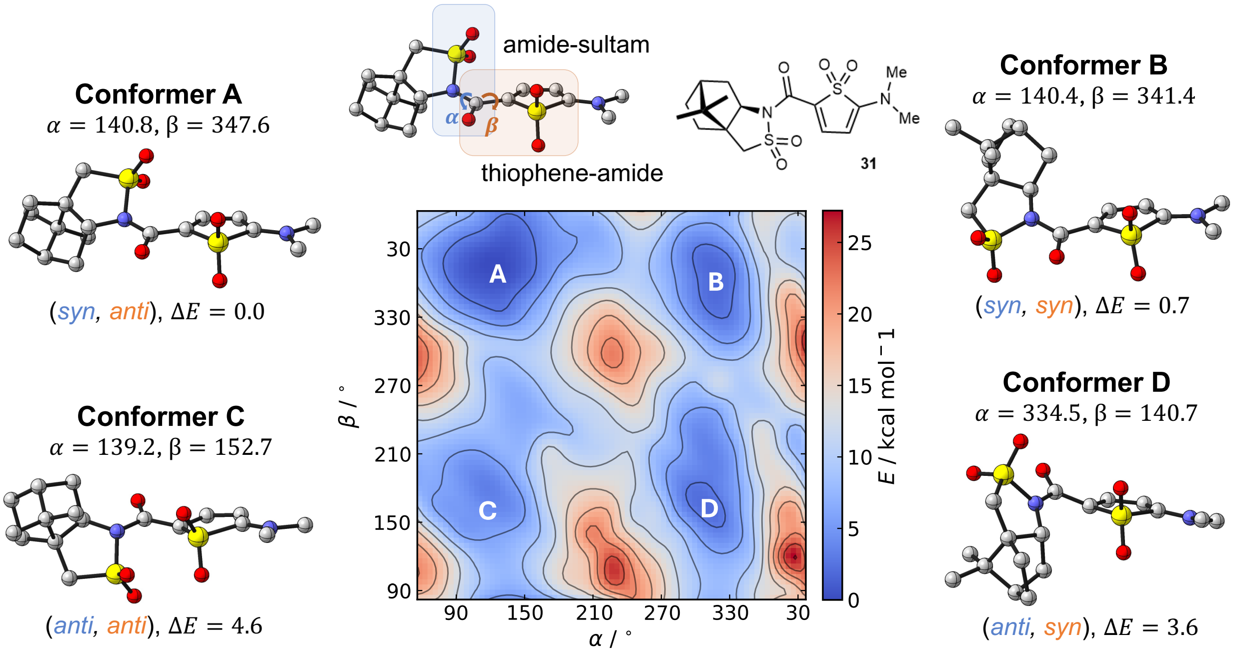

Figure S5. 2D energy profile (kcal mol^{-1}) of **31** as a function of torsion angles around the C–C and C–N bonds flanking the carbonyl generated using a machine learning interatomic potential (MLIP) trained at CPCM (MeCN)-B2PLYP-D3BJ/def2-SVP level of theory using the MACE architecture.[39,40] Each minimum was further characterised at the CPCM(MeCN)-DLPNO-CCSD(T)/def2-TZVP//CPCM(MeCN)-B2PLYP-D3BJ/def2-SVP level of theory (353 K / 1 M).

Table S13. Relevant second order perturbation theory values of NBO analysis. Calculations were carried out at CPCM(MeCN)-B2PLYP-D3BJ/def2-SVP level of theory. All values are given in kcal/mol.

Conformer A			Conformer B		
**Donor (L) NBO**	**Acceptor (NL) NBO**	**E(2)**	**Donor (L) NBO**	**Acceptor (NL) NBO**	**E(2)**
35. LP (1) O 1	107. BD*(1) O 1- C 2	1.25	35. LP (1) O 1	107. BD*(1) O 1- C 2	1.27
35. LP (1) O 1	109. BD*(1) C 2- N 3	0.89	35. LP (1) O 1	109. BD*(1) C 2- N 3	1.03
36. LP (2) O 1	109. BD*(1) C 2- N 3	23.75	36. LP (2) O 1	109. BD*(1) C 2- N 3	23.17

37. LP (1) N 3	107. BD*(1) O 1- C 2	4.61	37. LP (1) N 3	107. BD*(1) O 1- C 2	1.69
37. LP (1) N 3	108. BD*(2) O 1- C 2	26.37	37. LP (1) N 3	108. BD*(2) O 1- C 2	44.65

4.4. Mechanistic Analysis

4.4.1 Transition state Conformational analysis

Using the protocol described in Section 4.3, we identified 13 unique ground-state conformers of **29** (Table S12 and Figure S4). From each of these structures, TSs were searched *via* a constrained scan (Table S14 and Figure S5). The lowest lying transition state was found to be **TS5**, which was also found in a manual TS space exploration:

We evaluated the two lowest energy **Conformers A** and **B** discussed in Section 4.4. We considered attack by the indole unit from either the top or lower face, resulting in four transition states: **TS5**, **TS7**, **TS8**, and **TS9** (Figure S6). The upper face attack TS derived from Conformer **A** (**TS5**) is favored by 8.3 kcal mol^{-1} compared to **TS7**. This is due to the presence of an H-bond interaction stabilizing the transition state (see Figure S6). For the two facial transition states derived from Conformer **B**, the bornyl-unit similarly blocks the concerted attack of the indole unit in **TS9**, leading to a free activation energy of 21.1 kcal mol^{-1}. Interestingly, the H-bond in **TS8,** rather than stabilising the TS**,** limits a concerted attack ($\Delta G^{\ddagger}$ = 28.7 kcal mol^{-1}).

Table S14. Electronic activation energies relative to **2** (highlighted in grey) at CPCM(MeCN)-M06-2X/def2-SVP level of theory.

Ground state CREST rank	Activation ΔE_{el} rel. to 2 [kcal mol^{-1}]
100	36.8
148	42.0
15	37.4
18	31.8
19	
24	35.9
253	42.6
27	32.0
301	
34	
60*	26.6*
81	31.4
86	36.2
41	39.4
2	35.1

†19, 34 and 301 could not be optimised due to convergence issues.

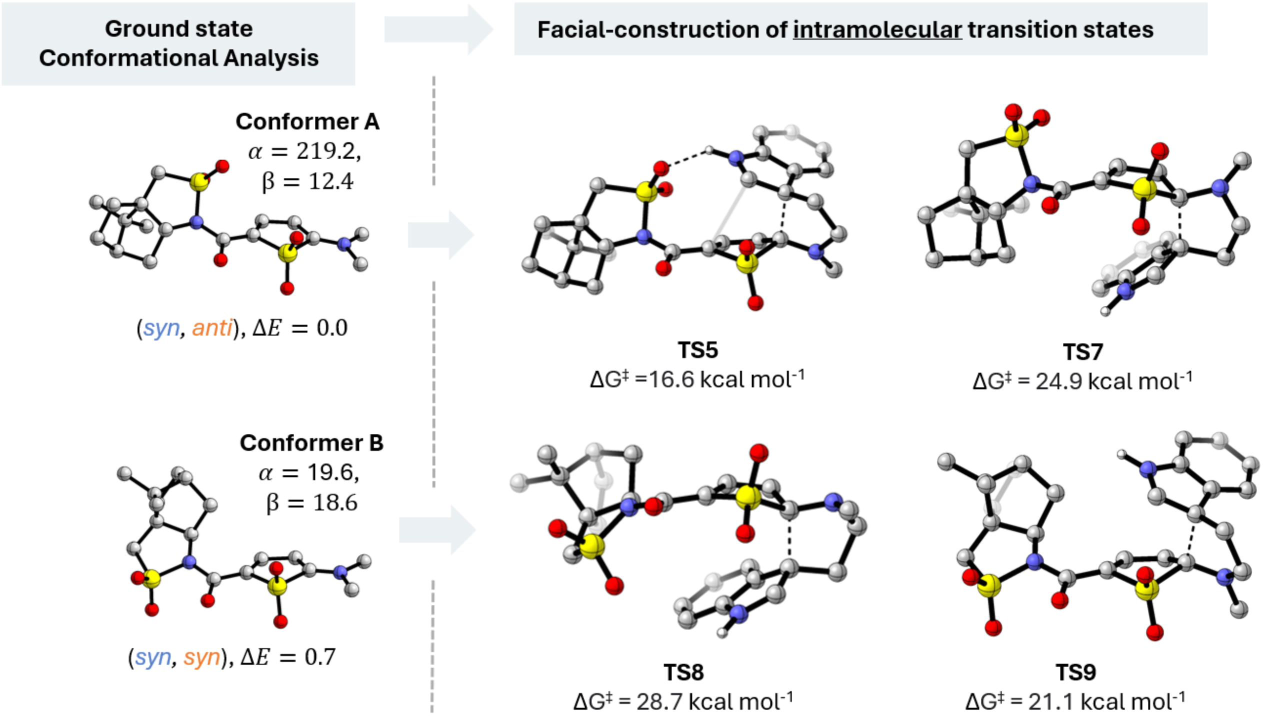

Figure S6. Analysis of the four potential transition states **TS5** – **TS9** computed at the CPCM(MeCN)-DLPNO-CCSD(T)/def2-TZVP//CPCM(MeCN)-B2PLYP-D3BJ/def2-SVP level of theory (353 K / 1 M). Activation energies in kcal mol^{-1}.

4.4.2. SO_2 extrusion

As discussed in the main text, the C–C bond formation process occurst via a stepwise mechanism. Once the second C-C bond is formed (**TS6)**, an unexpected, spontaneous extrusion of SO_2 leads directly to the dienamine product **Inter5** (ΔG= −34.1 kcal mol^{-1}). Attempts to identify an SO_2-bridged intermediate were unsuccessful (Figure S7a). In contrast, **TS5** corresponding to a Michael addition reaction, leading to the formation of a high-energy zwitterionic intermediate **Inter4** (12.6 kcal mol^{-1}). This contrasts with our previous results for furan/thiophene *S,S*-dioxide cycloadditions, where a stable SO_2-bridged intermediate **E** (Figure S7b) was found computationally as a local minimum.[40] We hypothesise that the electron donation ability of the tryptamine sidechain amine in **F** (Figure S7b) may promote the direct expulsion of SO_2, while in the electronically unbiased, fused carbocycle **E**, containing no electron-donating +M-substituent, this driving force is absent. In contrast, a -Chloro-substituent in the intermolecular case exhibits not enough +M effect to extrude SO_2, therefore **Inter3** could be located as a stable minimum. When the –Cl group in **Inter3** is computationally replaced with a strongly electron-donating –NMe_2 group, geometry optimisation leads to spontaneous SO_2 loss, yielding the final product without a stable intermediate. This process could be explored in more detail using MLIPs, *vide infra* (section 4.5.3)

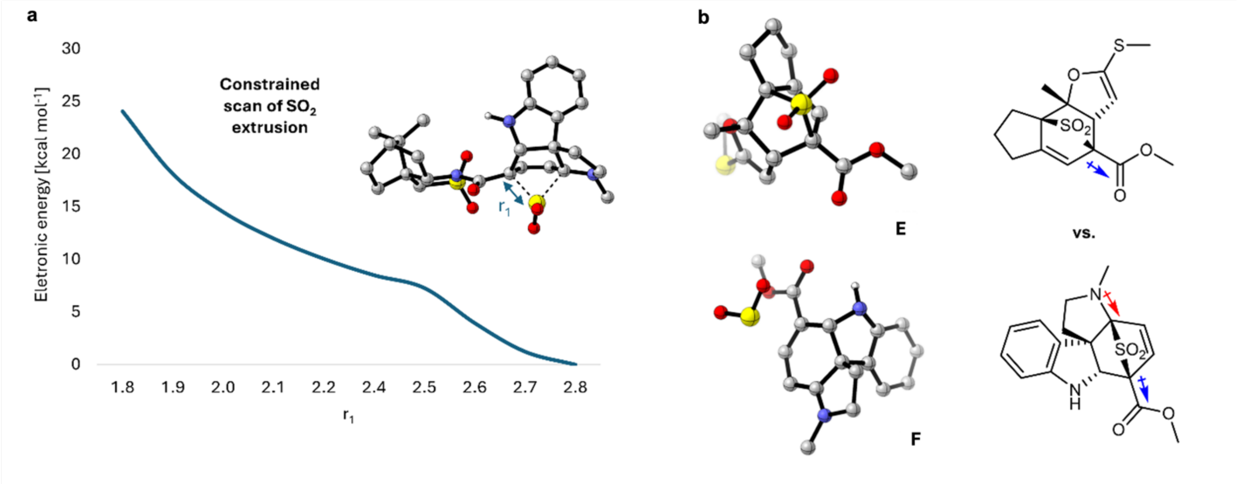

Figure S7. **a**. Constrained scan of r_1 showing no stationary point of a bicyclic SO_2-bridged intermediate at CPCM(MeCN)-B2PLYP-D3BJ/def2-SVP level of theory. No intermediate **F** was found, in contrast to **E** of from our previous work.[40] **b.** Schematic representation of the electron-donating (red arrow)- and electron withdrawing (blue arrow)-effects in intermediate cycloadduct (**E**) and spontaneous SO_2 extrusion (**F**).

4.5. Dynamics of the intramolecular cycloaddition reaction with MLIPs

4.5.1 Validation: The MLIP trained to model the intramolecular reaction between **29** and **Inter5** (Figure 4b main text) was validated using a dataset generated by two independent MLIP-MD simulations initialised from **TS5**. The simulation at 500 K in the NVT ensemble was propagated for 5 picoseconds with a timestep of 0.5 fs towards the reactant state (**29**) and product state (**Inter5**), generating 1002 configurations. Validation showed high accuracy through a point-to-point comparison of ground truth and predicted energies and forces, with force errors of 0.8 and 0.9 kcal mol^{-1} (Figure S7).

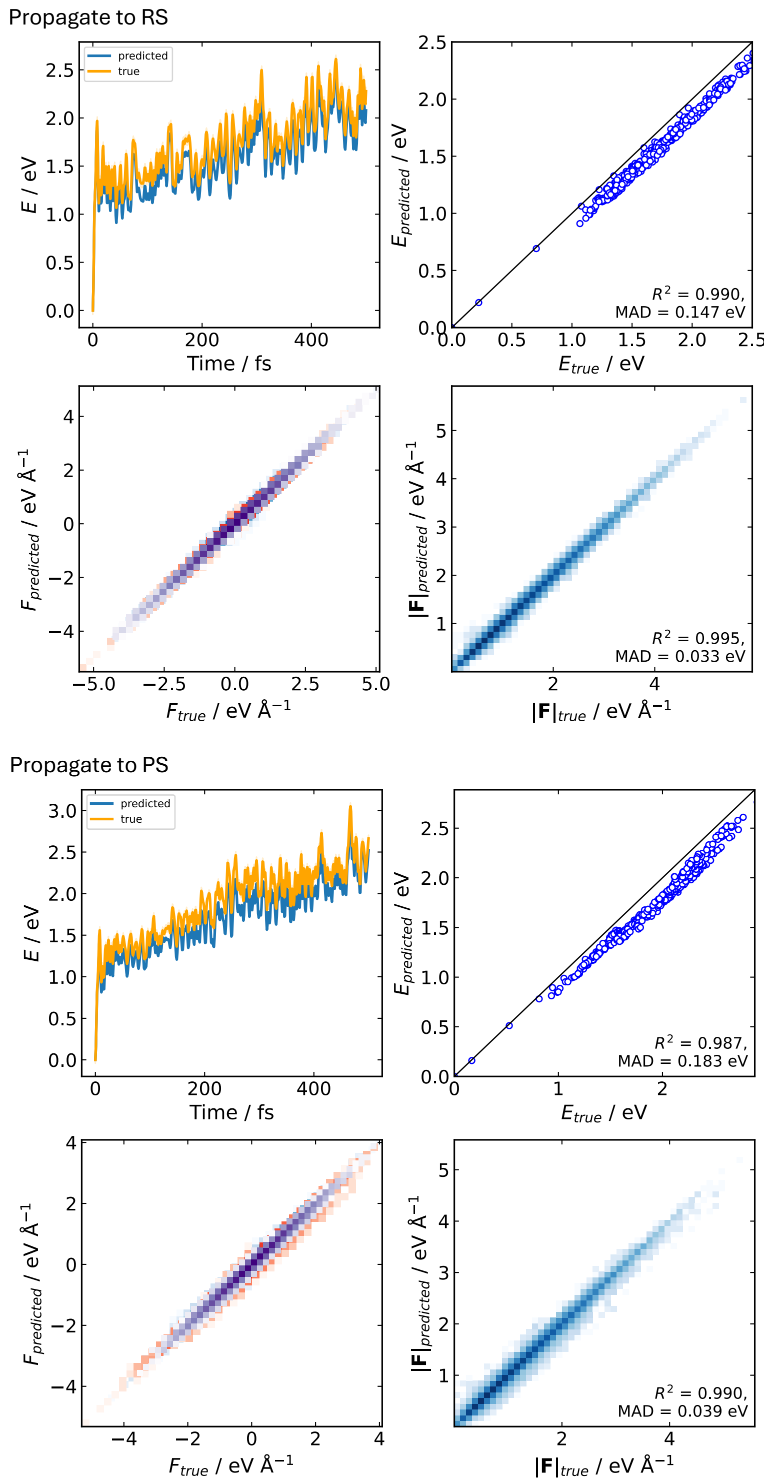

Figure S8. Comparisons of ground-truth (CPCM (MeCN)-B2PLYP-D3BJ/def2-SVP) and predicted (MLIP) energies and forces for modelling intramolecular cycloaddition substrate. Values were obtained during 5-ps MLP-MD trajectory initialised from **TS5** (500 K, time step = 0.5 fs), propagating to reactant state (RS) shown in upper panel and to product state (PS) illustrated at the bottom panel.

4.5.2 Downhill dynamics. 500 downhill MLP-MD trajectories were initiated from **TS5** (shown in Figure 4b) at 353 K and propagated downhill either towards the product or the reactant state for five picoseconds. Product formation was identified by both bond distances r_1^{CC} with r_2^{CC} being less than 1.6 Å, whereas distances over 3.0 Å indicated reactant (Figure S9a). Of these trajectories, 250 (50%) went to the reactant state and the remaining 250 (50%) toward the product state (Figure S9b), but only 159 of the latter reached the product state. For further discussion, see the main text, Figure 4.

The time gap between the formation of the two C-C bonds, reflecting the (a)synchronicity of the reaction, averaged 748 fs, ranging from 109 fs to 2459 fs. Such a long time gap supports the observation of **Inter4**, indicating a stepwise mechanism. The first C-C bond (r_1^{CC} < 1.5 Å) forms at around 20 fs, with the second (r_2^{CC}) only after 100 fs (Figures S9c and S9d) and main text (Figure 4). Notably, around 91 forward trajectories did not form the second C-C bond even after 5 ps, remaining at the intermediate stage, **Inter4** (Figure 4b).

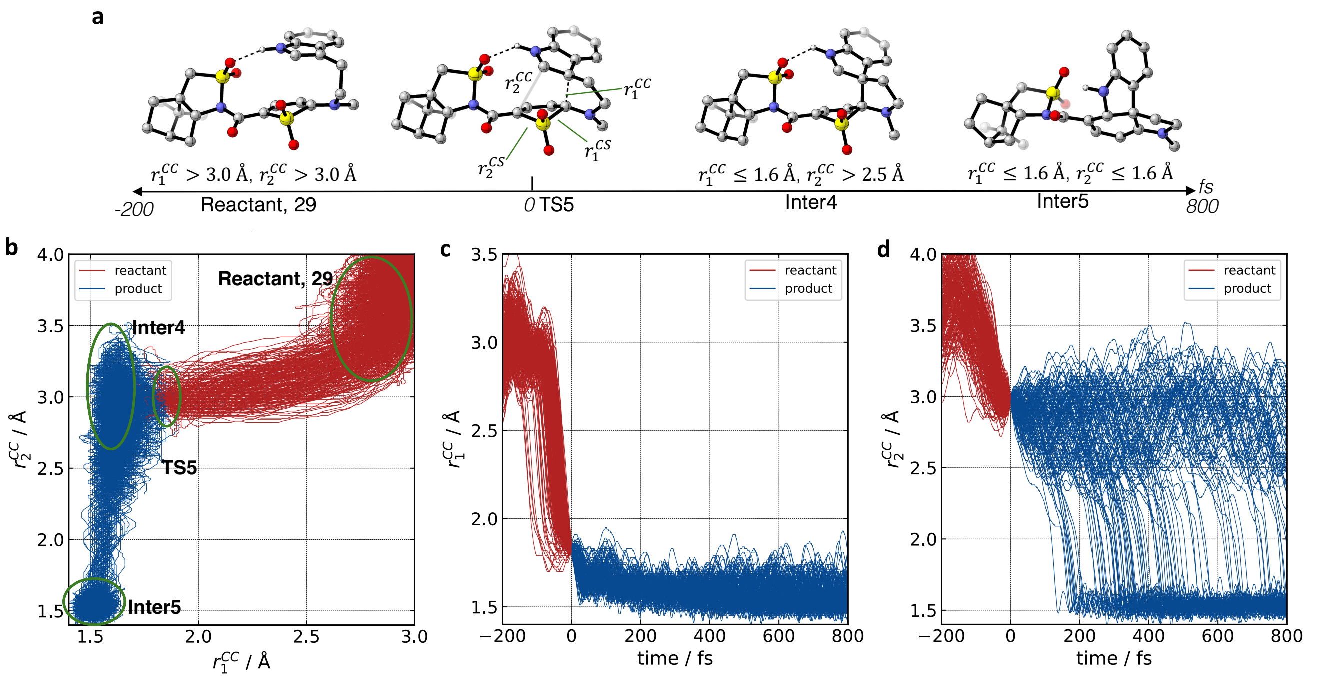

Figure S9. Downhill MLP-MD dynamics for intramolecular cycloaddition, initialised from **TS5,** with ground truth of CPCM(MeCN)-B2PLYP-D3BJ/def2-SVP level of theory under an NVT ensemble at 353 K. **a.** Representative geometries along the forward and a backward downhill dynamic highlighting key bond distances in Ångström. **b.** Change of r_1^{CC} with r_2^{CC} for 500 trajectories. Evolution of (c) r_1^{CC} and (d) r_2^{CC} over time, black dashed line at 1.6 Å indicates the C-C bond length at the product state.

The absence of an SO_2-bridged intermediate employing DFT calculations (section 4.4.2), was further supported through downhill MLIP-MD dynamics. These trajectories reveal the instantaneous breaking of C–S bonds, supporting the absence of a bicyclic SO_2-bridged intermediate, as illustrated in Figure S6a. Notably, only when

r_2^{CC} reaches 1.5 Å the C-S bonds break occurs (Figure S10), evidenced by r_1^{CS} and r_2^{CS} values > 2.5 Å, illustrating the high asynchronicity of the process. The average time gap between the two C-S bonds being broken is only 32 fs, confirming SO_2 release is a concerted mechanism.

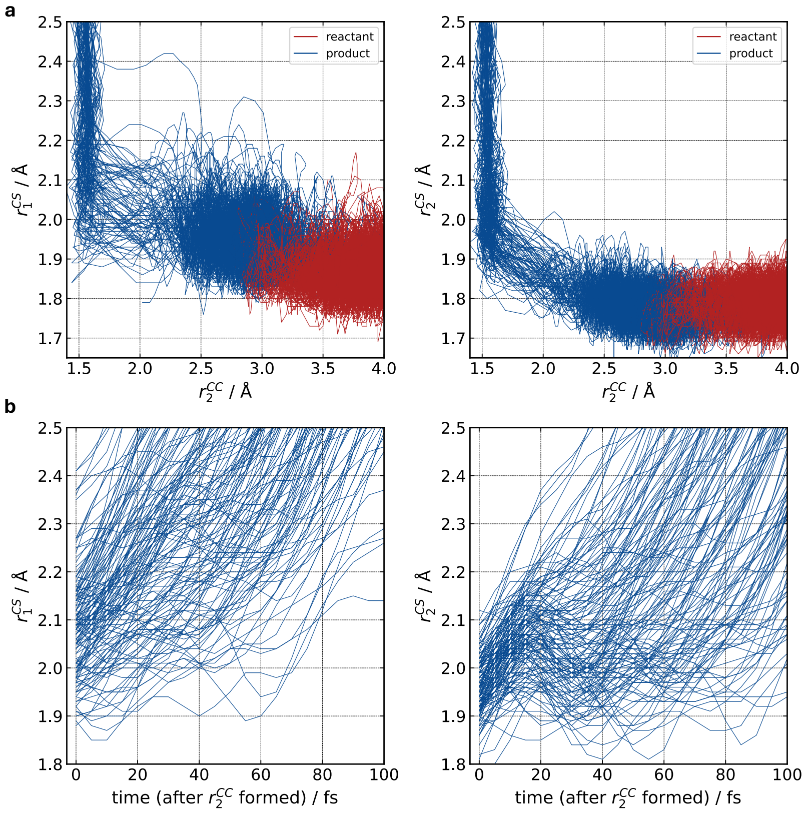

Figure S10. Downhill dynamics conducted by MLIP with ground truth of CPCM (MeCN)-B2PLYP-D3BJ/def2-SVP level of theory under an NVT ensemble at 353 K. **a**. Change of r_1^{CS} (right panel) and r_2^{CS} with r_2^{CC} for 500 trajectories **b**. Evolution of r_1^{CS} (right panel) and r_2^{CS} with time, where t = 0 defined as when $r_2^{CC} < 1.6$ Å.

4.6 Mechanistic Analysis for Intermolecular reaction

Conformational analysis and facial selectivity modelling were first carried out for the intramolecular reaction. For the intermolecular case, the TSs were obtained by optimising geometries starting from the lowest-energy intramolecular TS structures at the CPCM(MeCN)-B2PLYP-D3BJ/def2-SVP level of theory, yielding **TS1** and **TS2**—with calculated $\Delta G^{\ddagger}$ of 20.6 and 23.9 kcal mol^{-1}, respectively (see Figure 4a in main text).

To rationalize the energy difference between **TS1** and **TS2**, a distortion–interaction analysis (DIA) was performed (Figure S11). While DIA could not be extended to the intramolecular case due to challenges in defining appropriate fragments, the analysis of the intermolecular case revealed that the higher activation barrier of **TS2** primarily arises from greater distortion of the TDO fragment, which contributes 3.2 kcal mol^{-1} more distortion energy than observed

in **TS1**. Although this increased distortion is partially compensated by a stronger interaction energy in **TS2**, the net result is that **TS2** remains 2.7 kcal mol^{-1} higher in activation energy relative to **TS1**.

NBO analysis reveals that while **TS2** benefits from stronger lone pair N → C=O π^* delocalisation in the TDO fragment compared to **TS1**: E(2) of 51.7 vs 28.6 kcal mol^{-1} for **TS2** and **TS1**, respectively (**Table S15**, highlighted in light blue**)**, **TS1** benefits from having a more preorganized ground state (Figure S5, conformer **A** with α=140.8° β° =347.6°), suggesting that **TS2** experiences larger structural distortion to reach the TS.

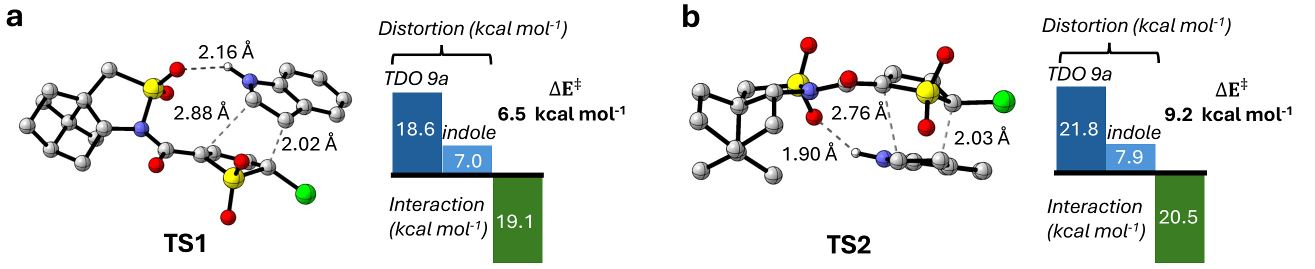

Figure S11. Distortion interaction analysis (DIA) in kcal mol^{-1} for **a. TS1** and **b. TS2.** The dark blue represents the distortion energy of TDO **9a**, the light blue represents the distortion energy of indole, and the green shows the interaction energy in the respective structures.

Table S15. Relevant second-order perturbation theory values from NBO analysis. Calculations were carried out at CPCM(MeCN)-B2PLYP-D3BJ/def2-SVP level of theory. All values are given in kcal mol^{-1} .

Donor (L) NBO	Acceptor (NL) NBO	E(2)	Donor (L) NBO	Acceptor (NL) NBO	E(2)
	**TS1**			**TS2**	
47. LP (2) O 1	136. BD*(1) C 2- N 3	30.64	47. LP (2) O 1	136. BD*(1) C 2- N 3	28.87
48. LP (1) N 3	135. BD*(2) O 1- C 2	28.62	48. LP (1) N 3	135. BD*(2) O 1- C 2	51.71
51. LP (3) O 15	139. BD*(1) N 3- S 14	34.27	51. LP (3) O 15	139. BD*(1) N 3- S 14	32.34
55. LP (1) C 19	171. BD*(2) C 17- C 18	183.16	55. LP (1) C 19	171. BD*(2) C 17- C 18	184.38
55. LP (1) C 19	178. BD*(1) C 20- C 24	88.07	55. LP (1) C 19	178. BD*(1) C 20- C 24	80.77
58. LP (3) O 22	177. BD*(1) C 20- S 21	22.12	58. LP (3) O 22	177. BD*(1) C 20- S 21	27.26
58. LP (3) O 22	181. BD*(1) S 21- O 23	15.19	58. LP (3) O 22	181. BD*(1) S 21- O 23	6.02
61. LP (3) O 23	177. BD*(1) C 20- S 21	27.69	61. LP (3) O 23	177. BD*(1) C 20- S 21	24.67

4.7 Appendix

Table S15. Reoptimised conformers generated by CREST of **30**. Entries with same features but different energy values are rotamers of vinyl iodide sidechain, e.g. entry No. 1-5.

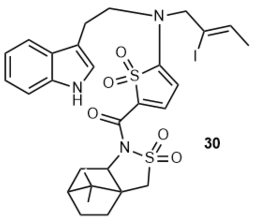

30

No.	1) face of indole attack	*2) endo/exo*	4) Amide	5) Sulfonamide	E_{el} at M06-2X/ def2-SVP [Ha]	Relative E_{el} [kcal mol^{-1}]
1	+z	*endo*	+x	-x	-2757.61457	1.5
2	+z	*endo*	+x	-x	-2757.61698	0.0
3	+z	*endo*	+x	-x	-2757.61594	0.7
4	+z	*endo*	+x	-x	-2757.61316	2.4
5	+z	*endo*	+x	-x	-2757.61316	2.4
6	+z	*endo*	-x	+x	-2757.60397	8.2
8	-z	*endo*	+x	+x	-2757.60342	8.5
11	+z	*endo*	-x	+x	-2757.60279	8.9
13	+z	*endo*	+x	-x	-2757.60832	5.4
15	-z	*endo*	+x	+x	-2757.60518	7.4
17	+z	*exo*	+x	-x	-2757.60922	4.9
18	+z	*exo*	+x	-x	-2757.61205	3.1
19	+z	*endo*	-x	+x	-2757.60397	8.2
24	-z	*exo*	+x	-x	-2757.60855	5.3
27	+z	*exo*	+x	-x	-2757.60857	5.3
28	-z	*exo*	+x	-x	-2757.61088	3.8
29		*unoriented conformer*			-2757.60425	8.0
31	-z	*endo*	+x	+x	-2757.6029	8.8
32	-z	*endo*	+x	-x	-2757.60955	4.7
34	-z	*endo*	-x	+x	-2757.60628	6.7
41	-z	*exo*	+x	-x	-2757.60857	5.3
60	-z	*endo*	+x	-x	-2757.61054	4.0
81	-z	*exo*	-x	+x	-2757.60481	7.6
86	+z	*endo*	-x	-x	-2757.60468	7.7
100	-z	*endo*	-x	-x	-2757.60861	5.3
148	-z	*endo*	+x	-x	-2757.60009	10.6
253	-z	*endo*	-x	+x	-2757.59357	14.7
301	-z	*endo*	-x	-x	-2757.60773	5.8

Table S16. Comparison of electronic activation energies between ground state **Conformer I** and transition state **TS II**.

Level of theory	E_{el} ground state	E_{el} TSII	ΔE
CPCM(MeCN)-M06-2X/def2-SVP	-2757.616984	-2757.554175	39.4
CPCM(MeCN)-M06-2X/def2-TZVP	-2759.859938	-2759.790947	43.3
CPCM(MeCN)-DLPNO-CCSD(T)/def2-TZVP// CPCM(MeCN)-M06-2X/def2-TZVP	-2755.610873	-2755.551461	37.3
CPCM(MeCN)-DLPNO-CCSD(T)/def2-TZVP// CPCM(MeCN)-M06-2X/def2-SVP	-2755.608146	-2755.547341	38.2
CPCM(MeCN)-B2PLYP/def2-SVP	-2756.230091	-2756.192816	23.4
CPCM(MeCN)-DLPNO-CCSD(T)/def2-TZVP// CPCM(MeCN)-B2PLYP/def2-SVP	-2755.608146	-2755.5721	22.6

Table S17. Thermodynamic quantities of the investigated intramolecular reaction of substrate **29**. Thermodynamic calculations were carried out at the CPCM(MeCN)-B2PLYP-D3BJ/def2-SVP level of theory (353 K / 1 M). Free energy G was calculated using the electronic energy of CPCM(MeCN)-DLPNO-CCSD(T)/def2-TZVP//CPCM(MeCN)-B2PLYP-D3BJ/def2-SVP level of theory via G = E_{el}(DLPNO-CCSD(T)) + [G(B2PLYP-D3BJ) − E el (B2PLYP-D3BJ)]

	E_{el} B2PLYP-D3BJ/def2-SVP [H_a]	H B2PLYP-D3BJ/def2-SVP [H_a]	G B2PLYP-D3BJ/def2-SVP [H_a]	S B2PLYP-D3BJ/def2-SVP [J K^{-1} mol^{-1}]	E_{el} DLPNO-CCSD(T)/def 2-TVP [Ha]	Free Energy G	ΔG
**SM 29**	-2342.90014	-2342.30631	-2342.42124	836.3	-2342.57667	-2342.09777	
**TS5**	-2342.87052	-2342.27957	-2342.39380	849.64	-2342.54808	-2342.07136	16.6
**TS7**	-2342.84660	-2342.25518	-2342.36695	831.33	-2342.53767	-2342.05802	24.9
**TS8**	-2342.84287	-2342.25134	-2342.36296	830.15	-2342.5319	-2342.05199	28.7
**TS9**	-2342.85849	-2342.26693	-2342.38089	847.64	-2342.54179	-2342.06419	21.1
**Inter4**	-2342.87472	-2342.28205	-2342.39582	846.16	-2342.55661	-2342.07771	12.6
**TS6**	-2342.87415	-2342.28266	-2342.39479	834	-2342.55431	-2342.07495	14.3
**Inter5**	-1794.83758	-1794.25755	-1794.36272	782.22	-1794.6242	-1794.14935	-34.1
**SO_2**	-548.09523	-548.08376	-548.11480	230.86	-547.983169	-548.002738	

Table S18. Thermodynamic quantities of the investigated intermolecular reaction between **Indole** and **TDO 9a**. Thermodynamic calculations were carried out at the CPCM(MeCN)-B2PLYP-D3BJ/def2-SVP level of theory (353 K / 1 M). Free energy G was calculated using the electronic energy of CPCM(MeCN)-DLPNO-CCSD(T)/def2-TZVP//CPCM(MeCN)-B2PLYP-D3BJ/def2-SVP level of theory via G = E_{el}(DLPNO-CCSD(T)) + [G(B2PLYP-D3BJ) − E el (B2PLYP-D3BJ)]

	E_{el} B2PLYP-D3BJ/def2-SVP [H_a]	H B2PLYP-D3BJ/def2-SVP [H_a]	G B2PLYP-D3BJ/def2-SVP [H_a]	S B2PLYP-D3BJ/def2-SVP [J K^{-1} mol^{-1}]	E_{el} DLPNO-CCSD(T)/def 2-TVP [Ha]	Free Energy G	ΔG
**Indole**	-363.18993	-363.05249	-363.08694	303.51	-363.14265	-353.03967	

**TDO 9a**	-2267.19314	-2266.84561	-2266.91350	598.22	-2266.76274	-2266.4831	
**TS1**	-2630.39034	-2629.90475	-2629.98511	708.08	-2629.89511	-2629.48988	20.6
**TS2**	-2630.38619	-2629.90457	-2629.98014	700.93	-2629.89069	-2629.48463	23.9
**TS3**	-2630.40250	-2629.91640	-2629.99497	692.02	-2629.90471	-2629.49989	14.4
**TS4**	-2630.42165	-2629.93540	-2630.01350	688.11	-2629.91931	-2629.51125	7.2
**Inter1**	-2630.40257	-2629.91550	-2629.99614	710.48	-2629.91320	-2629.50678	10.0
**Inter3**	-2630.42804	-2630.93991	-2630.01896	696.44	-2630.94044	-2629.53136	-5.4
**10a**	-2082.35357	-2081.87798	-2081.95186	650.87	-2081.95242	-2081.55072	-25.7
**SO_2**	-548.09523	-548.08376	-548.11480	230.86	-547.983169	-548.002738	

5. Coordinates

XYZ Geometries

SM 29

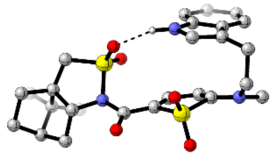

Electronic energy -2342.90014

Number of imaginary frequencies: 0

O	-4.29917689007490	2.41261867411410	-2.45207076595641
C	-4.38553650933363	1.31694545261033	-1.90716916326272
C	-3.26943736310291	0.69341817165916	-1.22483799055511
C	-3.18005315870365	-0.23226561117902	-0.21333977299881
C	-1.86843840368991	-0.61025846539967	0.20828724395835
C	-0.86016941708856	-0.06933300259467	-0.55853097245965
S	-1.63092120040869	1.23942298932696	-1.63232173647898
O	-1.38146103718787	1.14847826610980	-3.08224392653918
O	-1.26076836241111	2.49870975679489	-0.96153247167595
N	0.47275909551436	-0.12220392418078	-0.43580018813259
C	1.03321952248831	-0.71245921685886	0.77607782043765
C	1.28644716479299	-0.16456639743167	-1.65171968663251
C	1.34977889240476	-1.59589869095050	-2.24932181841224
C	-0.00412887798305	-2.23719786648318	-2.30602767862096
C	-1.07763876319097	-1.83248941069226	-3.08537658173647
N	-2.21684166410489	-2.49672098592729	-2.71035048595659
C	-1.92778524998933	-3.33821740172355	-1.66440567649524
C	-2.77074688900172	-4.18120696499225	-0.92278124320253
C	-2.19201326457816	-4.93142961618473	0.09798871631816
C	-0.80438917671310	-4.85282076329970	0.36927439820154
C	0.02764285775371	-4.01066504132890	-0.36482410738247
C	-0.52990055684184	-3.22426552065240	-1.39502123743210
H	0.84370318801286	0.51888121993778	-2.38614851452119
H	2.29324411655593	0.20178515788025	-1.40954953655567
H	2.02837237509983	-2.21322736192836	-1.64260395418524
H	1.80640637372129	-1.50799421253738	-3.24755820433291
H	-1.11485862473729	-1.07997347728167	-3.86655692968518
H	-3.15222215951152	-2.26253264680666	-3.04470310130817
H	-3.84071709749794	-4.22352172689979	-1.13192191629999

H	-2.81809100801526	-5.59438226745406	0.69872208791123
H	-0.38246200824286	-5.46388480848700	1.16981505935019
H	1.09661311828994	-3.96400349902676	-0.14485374591124
H	0.89815378294975	-1.80312616891962	0.80341048048591
H	0.53922975966015	-0.27161283932315	1.65126487708674
H	2.10456705020834	-0.48098599114801	0.80954932765569
H	-7.32865880944442	-1.11782411772290	-3.95159157280542
H	-7.91229641744129	-1.86702745750832	-2.41863321873269
H	-9.77719728340300	0.26691151918465	-3.68527890083246
C	-7.40819044402118	-0.99917219157117	-2.86305721358841
H	-8.50079540792464	1.28993613757560	-4.36227696364720
C	-8.98729322583599	0.98357459034518	-3.42606033278455
H	-10.71053724098055	0.02577579020022	-1.78604665866474
H	-9.84055437823007	-1.31377299436921	-1.01163834184413
O	-4.77192983352217	-1.31273291433432	-3.40115997837606
C	-10.00730520022727	-0.22582347536586	-0.98471404442933
C	-7.96295569601785	0.35171796033583	-2.46228075926458
S	-5.69490511842122	-1.04920328854092	-2.27978383543149
H	-10.62139686045978	2.11589561917196	-2.46770815521589
C	-9.53340833055263	2.18306212233329	-2.59809932662658
O	-5.55063819273031	-1.88400478728546	-1.08140999468259
C	-8.66741530081880	0.51490886859298	-1.07152452352149
H	-10.49456487831793	0.00787165059312	-0.02663036349292
H	-6.54393920476414	1.73886871939538	-3.40059504882525
C	-6.79017446069201	1.36093710143159	-2.39781147905804
H	-9.32076198933007	3.14893573799829	-3.07734462574421
H	-7.71537662317015	-0.99551834779854	0.17496060393767
N	-5.61883370353125	0.63630997540854	-1.87762209380889
C	-8.79276675877928	2.05269152797795	-1.24844021553184
C	-7.85359642913853	0.09460352662173	0.15829653277736
C	-7.32956218295844	2.48342538514720	-1.47873184259348
H	-8.41182250005549	0.37291803922805	1.06479864315174
H	-9.28131556466004	2.57880210575403	-0.41608562762590
H	-6.86247019217434	0.55357882090314	0.21671716524481
H	-7.25448192959958	3.46564407329789	-1.96327990241328
H	-6.75910743102468	2.52808088058477	-0.54278153116443
H	-4.06616453475146	-0.68377904813767	0.23058706388333
H	-1.69733049206386	-1.33478333818830	1.00134593703579

TS5

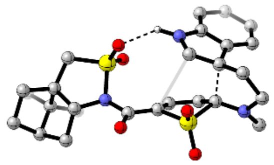

Electronic energy -2342.87052

Number of imaginary frequencies: 2

Imaginary frequencies: -267.10 cm^{-1} (and -25.7 cm^{-1})

O	-0.41036364498809	2.63970986528052	-1.28493199970108
C	-0.51198611434982	1.62776096363494	-0.58879157687774
C	0.55409653923365	1.01019174861497	0.12842478651759
C	0.56042716794574	0.17218185403428	1.27436213399560
C	1.78825191380412	-0.26271457322519	1.72490587034870
C	2.89570342471299	0.05591758693242	0.84440977616850
S	2.20674984043334	1.56969051402923	-0.10317742108504
O	2.59027040947742	1.66735740660572	-1.53365122956821
O	2.56117969892239	2.73047868228518	0.74438064039582
N	4.18861040383691	0.21635993864138	1.28666896401914
C	4.54592082957974	0.20621566676736	2.69198255030455
C	5.21841850035084	-0.07068511139313	0.29759997337555
C	4.50572616816462	-0.65260349146234	-0.93888795329718
C	3.16198947815224	-1.19353760295087	-0.47646851288389
C	1.97211451311802	-1.18163917730385	-1.30418534033756
N	1.17272878576856	-2.18515822462107	-0.96576308369990
C	1.77372630124572	-3.00751810894846	-0.00572771208519
C	1.28081852291478	-4.15779574297952	0.60780857580625
C	2.12753797207527	-4.79313513604991	1.52241942365629
C	3.41104579074650	-4.28803746976238	1.79880625719395
C	3.88302712609668	-3.12302906038339	1.17907916797767
C	3.05306433356937	-2.47863180381569	0.25956024441475
H	5.78779327197523	0.83292853628993	0.02527834112258
H	5.93509502097068	-0.78858766013023	0.72901865626643
H	5.10525223819667	-1.44939859877257	-1.39881509133858
H	4.33407010414165	0.12962898176657	-1.68550202117072
H	1.69639407570641	-0.47169538216832	-2.07735757227807
H	0.21714382070455	-2.25320985664839	-1.32859181231792
H	0.28159268941933	-4.53501570893207	0.38774010190127

H	1.78700702448570	-5.69899203026459	2.02676612483634
H	4.05030677142841	-4.81349375671270	2.51025377185703
H	4.87831942942820	-2.74200838424929	1.40957856597792
H	4.75845413702786	-0.80909720623688	3.07038258290446
H	3.73221204150924	0.64431250273253	3.28136005554770
H	5.44331399866100	0.82390798223057	2.83827752741896
H	-3.76439112077864	-0.72047568686578	-2.31115695116577
H	-4.28342006088208	-1.32744775123346	-0.69556983332426
H	-6.07821267432209	0.86635868927769	-2.00566283505970
C	-3.75821243839576	-0.52597497705364	-1.23072181581975
H	-4.77283584323168	1.75592979704752	-2.80498583261625
C	-5.22587314763507	1.53508965178760	-1.82873059666107
H	-6.91338527501511	0.79217879127912	-0.04935724103554
H	-6.09719656229198	-0.56023412879855	0.76001340465260
O	-1.19390342736784	-1.03701255808092	-1.93230285993786
C	-6.18586503564854	0.53678482178260	0.72888138915106
C	-4.19507575859503	0.88216421210077	-0.88661507181601
S	-2.01273053256345	-0.64626190843741	-0.76126497366868
H	-6.71883232010350	2.83275359558884	-0.84950519460696
C	-5.63819493693911	2.81375747391298	-1.04278546311704
O	-1.82679746926116	-1.45524189415994	0.45128543502299
C	-4.80441512099111	1.17156405170279	0.52790582074427
H	-6.60226745636832	0.85975360385092	1.69448553341340
H	-2.73727700348243	2.10770969445503	-1.97841408397315
C	-2.95067649894770	1.80232357839281	-0.94413245598365
H	-5.38762968065851	3.73478241556214	-1.58763582202065
H	-3.87274042115230	-0.33799613648967	1.79092806216049
N	-1.80724886507707	1.02541083330467	-0.43960985384045
C	-4.83049536180511	2.70266295626906	0.26967564972771
C	-3.95175592242307	0.75689998413158	1.73307500170296
C	-3.35668112433648	3.01074458071920	-0.06614281772079
H	-4.44492386346698	1.10328836879915	2.65384453538223
H	-5.23177090526552	3.30790937222459	1.09503023972616
H	-2.93513924928868	1.15955236833975	1.71869680136899
H	-3.24253871782842	3.95694519550697	-0.61118868907791
H	-2.72992929963666	3.06579824744203	0.83264790003671
H	-0.36936538886466	-0.10686058525740	1.76871912964992
H	1.91701289815821	-0.89801679993580	2.59958672333999

TS6

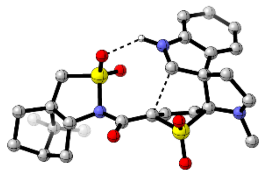

Electronic energy -2342.87415

Number of imaginary frequencies: 1

Imaginary frequencies: -109.3 cm^{-1}

O	-0.26015020216060	2.47228483973034	-1.33199962633317
C	-0.40554538647154	1.44747560896688	-0.66615885692957
C	0.64528489306365	0.78008786968020	0.05525255462284
C	0.55398049058570	0.11720999984037	1.33927363509535
C	1.74653977857401	-0.23869867536511	1.89276095846800
C	2.89659174145048	-0.02373876373497	0.98544935412840
S	2.25386696751005	1.55917171048760	0.06665484718193
O	2.81982073347838	1.73398760683531	-1.29504651080684
O	2.31683880756544	2.73932981076167	0.96131798900093
N	4.20908128576784	-0.00218246716616	1.47971023601716
C	4.55125521682502	0.89411267837692	2.56927248127047
C	5.14846307448027	-0.06381731554531	0.36081428088570
C	4.39710499066670	-0.80158187118931	-0.76739986867658
C	2.97697098998044	-1.08192965456381	-0.20770810545286
C	1.82383203009631	-0.97051344067027	-1.17388877213838
N	1.12569014309107	-2.10099304540659	-1.16242450008379
C	1.60920911781977	-3.01142669167801	-0.20849874874852
C	1.10187575167692	-4.25559608040224	0.15149894923052
C	1.81382602560122	-4.97000890624725	1.12664555305897
C	2.97918094791270	-4.44495696817155	1.70380062593809
C	3.46784366216884	-3.18193392082913	1.32713337944881
C	2.77211780240817	-2.46858880709272	0.35775573359880
H	5.43496089322724	0.95116864438604	0.02930307404103
H	6.06639866267571	-0.58265600698640	0.67471269883179
H	4.87860974342153	-1.74784812201910	-1.04080904262944
H	4.33072509387272	-0.17325212620456	-1.66097657807497
H	1.75901290190368	-0.27993010485377	-2.00943698180959
H	0.26987675961486	-2.20742570727194	-1.71310092497968
H	0.19040396922477	-4.64935204871024	-0.29924247978864
H	1.45249013908289	-5.95125172554921	1.43933897678692

H	3.51308763770976	-5.02502326157579	2.45836073854662
H	4.36267988051557	-2.76439811553432	1.78970271813938
H	5.51034480620165	0.58088733695333	3.00524915216679
H	3.78042867163938	0.84036262751610	3.34817434279388
H	4.64199543401279	1.94303815372030	2.23735072031941
H	-3.86864794488069	-0.89224300976972	-2.24059455755241
H	-4.23107137180604	-1.37428555088953	-0.54302828013065
H	-6.05051799482381	0.81949335435356	-1.89786139605822
C	-3.74447376611128	-0.62703811428865	-1.18285107228456
H	-4.76672123646907	1.64406093922705	-2.79524699008931
C	-5.17097933678991	1.46711437818321	-1.78903746205911
H	-6.76669691326531	0.81412834127420	0.09718053251336
H	-5.94521094117410	-0.50853094852003	0.94866233451207
O	-1.27754314040114	-1.11869677571942	-2.15807314173240
C	-6.00913001912981	0.58671269070375	0.85511929436624
C	-4.10902320822496	0.81329966297640	-0.88357407873689
S	-1.96206797156841	-0.77369127814072	-0.89037611608853
H	-6.56898624993259	2.83515997431766	-0.76677863114550
C	-5.50288218072834	2.78163473879962	-1.02382465070654
O	-1.64477962467965	-1.60899413362028	0.27425596863209
C	-4.62383299999286	1.17120524894005	0.55265956737610
H	-6.37054001239907	0.97762649600832	1.81778427507700
H	-2.66425216063433	1.94442349932169	-2.09772873273086
C	-2.83976625637067	1.68896608129847	-1.04219506578684
H	-5.26338841726484	3.67753463491254	-1.61362095665519
H	-3.61542967374731	-0.33076458426871	1.76681447844665
N	-1.71244636276131	0.88534110152490	-0.54816805517334
C	-4.62060700523958	2.69203802284817	0.24136445547283
C	-3.71363489896603	0.76383213054894	1.71809150918304
C	-3.16191299684541	2.94283822894371	-0.19314694504822
H	-4.17161259468317	1.09579634641947	2.66186657376124
H	-4.95290865254822	3.33897552246391	1.06568002923440
H	-2.70506928071054	1.18236326645507	1.66399636340267
H	-3.05575724883827	3.86200872939257	-0.78380641339405
H	-2.48160643381457	3.01825212554456	0.66400491117357
H	-0.40854611153743	-0.05556434371789	1.81951026096471
H	1.86661255114532	-0.72329583601016	2.86066798813689

TS7

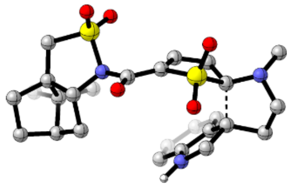

Electronic energy -2342.84660

Number of imaginary frequencies: 1

Imaginary frequencies: -54.1 cm^{-1}

O	-2.83292627997198	0.45950391388448	0.90886800301857
C	-2.29050155160782	-0.10524609089805	-0.04654471094082
C	-0.91490139814091	-0.18703132605254	-0.33776918780472
C	-0.18902539722039	-0.91081808076420	-1.34905682567376
C	1.13966939018399	-1.11941394738905	-1.13212209582075
C	1.61289818451587	-0.75870109285815	0.23071628754011
S	0.29846995408256	0.54584029490994	0.69122074550579
O	0.80761652737429	1.82790766572449	0.15895015862963
O	-0.05417532877417	0.54511808631092	2.13642672221081
N	2.90517252209918	-0.20916110327305	0.39199194274965
C	3.86147871528692	-0.32964865175981	-0.70164446487702
C	3.39075152551025	-0.56055005808837	1.71926261266597
C	2.97641893495643	-2.02621701645409	1.89222180336993
C	1.53939195556121	-2.06215792460905	1.33332215325147
C	0.53691975322554	-1.91998118855359	2.42657616341052
N	-0.39621046593445	-2.82781277151517	2.32813882405010
C	-0.15498064606678	-3.70475501454707	1.24300287070262
C	-0.91331093733588	-4.78800152058284	0.81370989705727
C	-0.39668187403159	-5.51865647331820	-0.26403776310108
C	0.82735200521109	-5.16593834254323	-0.85516177088836
C	1.56517578042074	-4.06130902417826	-0.40439445658822
C	1.06077071708340	-3.31824457138714	0.66082978780587
H	-0.69114969445024	-1.23800749018464	-2.25846948759897
H	1.78813257641755	-1.62843637966135	-1.84081950803797
H	4.47363981686200	-0.40416111592483	1.79474095820593
H	2.90024020082829	0.06802051373175	2.48000676113451
H	3.03278413463886	-2.37980137206091	2.93139004070015
H	3.60864466254006	-2.67721200665699	1.27205790333570
H	-1.17307582738396	-2.92142872396728	2.98370267141103

H	-1.85335864713250	-5.05715936842238	1.29582592890489
H	-0.95078234029154	-6.37892193205322	-0.64210983663583
H	1.21126410017859	-5.76278228247554	-1.68396826696867
H	2.51254174648856	-3.79712130726652	-0.87597163482663
H	4.15583756661332	-1.37268266986033	-0.92138145613863
H	4.76322291362508	0.23618593181528	-0.43553880198957
H	3.43974557299910	0.11336406852203	-1.61127124711278
H	-5.78478875989087	-0.17635904087727	-2.71409430890949
H	-5.02885957078182	-1.45903685023457	-3.72767570256552
H	-7.36320705848537	-2.31759935750453	-1.80456446252008
C	-5.05164271060270	-0.99319389038366	-2.73445126727775
H	-6.95680389380724	-1.21468239943164	-0.48068285739080
C	-6.63638675773556	-2.12596660939261	-1.00439288655335
H	-6.67159713774365	-4.19706566627915	-2.49924821513775
H	-5.43764964021970	-3.88481809967454	-3.73518095377006
O	-3.61589823081279	1.28259020845833	-2.36537455961250
C	-5.59686480426661	-4.24410637412432	-2.70657431245540
C	-5.21379089709686	-1.95219038563142	-1.57233097456984
S	-3.44711770115418	-0.17556275226908	-2.49261857753986
H	-7.14749655598855	-4.16957085250835	-0.34388134530784
C	-6.47654745348394	-3.34962673334770	-0.05510678989298
O	-2.46594703379796	-0.63845106317442	-3.48908900763672
C	-4.73278119017429	-3.43628639116164	-1.73007438493808
H	-5.29590653844433	-5.30191777673560	-2.67833608711392
H	-4.89638417325833	-0.62740830597512	0.15100107865462
C	-4.36296443673656	-1.42057499817208	-0.39358306831950
H	-6.70099966942804	-3.09705342679698	0.99087204568911
H	-3.11534977464134	-3.24332556309127	-3.17565635030412
N	-3.13092796639494	-0.87126671757179	-0.97085030313547
C	-4.99350326898261	-3.74533009643616	-0.23047681569560
C	-3.27611960548913	-3.64156880936720	-2.16350466553816
C	-4.14353636427405	-2.67526973848607	0.48638394110511
H	-3.06026129291975	-4.72039249052245	-2.19090262132720
H	-4.76395323632447	-4.77488229990406	0.08083339003366
H	-2.54601013496348	-3.16526538177100	-1.50415162034529
H	-4.47406490749549	-2.50780688980691	1.52055761381521
H	-3.07987615354617	-2.94126963593662	0.51448659378965
H	0.57863360418070	-1.24282472048222	3.27226634221337

TS8

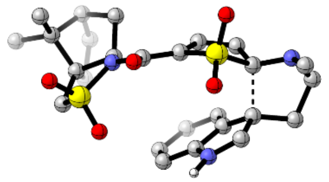

Electronic energy -2342.84287

Number of imaginary frequencies: 2

Imaginary frequencies: -259.3 cm^{-1} (-37.3 cm^{-1})

O	-2.70195924726757	1.48282703444918	1.82564291021231
C	-2.71500101982567	0.65562242465056	0.89832701802960
C	-1.53735149764132	0.16624171506978	0.27242858623875
C	-1.18221115834577	-0.59177099179476	-0.88980746737719
C	0.11817103949783	-1.00690151411690	-0.98004262962176
C	0.90371034482329	-0.73758634404773	0.23444389023685
S	-0.00752046726037	0.72191120376215	0.95658499941871
O	0.50020680001511	1.94178321428396	0.28513841849516
O	0.07269214985756	0.72546804339388	2.43463374275570
N	2.30250339916784	-0.62008657302258	0.10651913861500
C	3.01764128619450	0.10499461234258	1.15640551963030
C	2.77528053610115	-1.98521959347675	-0.08500984515423
C	2.09182994918687	-2.83683738756700	1.02105778911465
C	0.73142124201901	-2.18617974212079	1.33966200553321
C	0.54367451411519	-1.77099377747300	2.73711076905479
N	-0.64301481500190	-2.11280081954700	3.18080869373417
C	-1.37112720450121	-2.80628876900621	2.19384144395700
C	-2.65933327505974	-3.32473799476182	2.26137998384450
C	-3.10982228333505	-4.01982767633606	1.13270539550398
C	-2.28507499890158	-4.18559795838978	0.00747908987531
C	-0.99190886906439	-3.64362587073270	-0.03622370868993
C	-0.53308365866189	-2.93419395653608	1.07356552329720
H	-1.88563572974313	-0.78798288896222	-1.69392936481555
H	0.53803794557158	-1.52251739360185	-1.84098351512427
H	2.47204501315314	-2.34629670736293	-1.07650116790764
H	3.87020524231981	-2.02045329135148	-0.02840141166518
H	2.71757739348789	-2.84973467249243	1.92361206379787
H	1.95217332116803	-3.87735464058870	0.70182599122390

H	1.26595796276277	-1.28623330626823	3.38849891138316
H	-0.99945513819825	-1.88095185672381	4.10683748438321
H	-3.28643192770070	-3.17924077736466	3.13914135858305
H	-4.11346341283132	-4.44774805223924	1.13329023271829
H	-2.65982737999183	-4.74781169323003	-0.84964060841469
H	-0.36116249900228	-3.78483104360695	-0.91340940916233
H	4.07771614222314	0.15059436945492	0.87820394602881
H	2.94604143923548	-0.37176535973793	2.14995980189061
H	2.63367273420293	1.12734330846200	1.22506699006202
O	-5.76451821932677	1.14737563160314	2.20863622328211
H	-5.77942994874375	-2.27099748770165	1.52668551747672
H	-5.12675682622077	-3.23587432951510	-0.92399166540892
C	-6.08185916888343	-1.30778184512000	1.09581128178200
S	-5.02379491842466	-0.08294042329966	1.90692539018394
H	-3.64388746672413	-1.66880895170801	-0.49758774206721
C	-5.92000659779163	-2.52661218085420	-1.19346343792323
H	-7.11562507191181	-1.07129277785122	1.37840227185567
H	-6.88163387673579	-3.01639252175419	-0.99200776731832
N	-3.98578169266259	0.12436291568654	0.54072096349523
C	-4.29837130089221	-0.78519897886896	-0.56198719573080
C	-5.78494627847891	-1.22221223716347	-0.38364846285059
O	-4.29393757986168	-0.71449692672985	3.01803211135614
C	-5.81649830896938	-2.02362676208660	-2.66234450033795
H	-4.93585887788831	-2.43148604926149	-3.17889448648316
C	-4.29829002580623	-0.17028450306784	-1.99002347529403
H	-3.53688637403508	-0.62690621875789	-2.63501264908331
H	-6.69967140025140	-2.31100861105670	-3.24799246000496
C	-6.59164893833150	-0.20462639262477	-1.26452322320159
H	-8.22686832020815	-1.59276701244177	-1.83437078152882
C	-5.71376910569698	-0.48964699188899	-2.50718559543895
H	-8.58577826360478	-0.52815450521819	-0.45938388842649
C	-8.06752693236424	-0.58753890003671	-1.42890627511893
H	-4.09910253473256	0.90794002337522	-1.94934963803344
H	-7.18691236968501	1.40983228903074	0.06853942325521
C	-6.56524941714117	1.26336943553631	-0.82444938980210
H	-5.56549616268539	1.63795842719951	-0.58762306395873
H	-5.98736099583970	0.07210955459248	-3.41175726918568
H	-8.55800119965481	0.12655721098849	-2.10699807361133

H	-6.98441969921585	1.88178084558560	-1.63301571156385

TS9

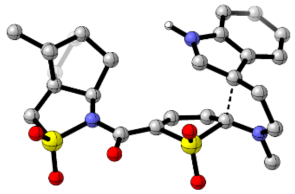

Electronic energy -2342.85849

Number of imaginary frequencies: 1

Imaginary frequencies: -313.9 cm^{-1}

O	0.24017264855583	3.14531239169493	-2.30582338099542
C	-0.47760242586646	2.15456409603390	-2.11609352084469
C	-0.05616428607552	1.05129191752866	-1.29273456318348
C	-0.72168687642212	0.05908487346447	-0.53871994761324
C	0.05096382399654	-0.77318775175488	0.25699650797952
C	1.46758379689219	-0.62346209913046	0.07138956567978
S	1.60219647027844	1.11700637878118	-0.69067748472555
O	2.61652295939948	1.26476586551578	-1.76245524923208
O	1.76074575222571	2.01141020812325	0.48254491561531
N	2.40481310990684	-0.92564657519843	1.03618995682851
C	2.19343579521531	-0.63234633960274	2.44102465042178
C	3.75938285599731	-0.92553094778389	0.51190507861875
C	3.66090725989116	-1.43941766798304	-0.94658879572355
C	2.19804364566710	-1.72612132725625	-1.26102829653650
C	1.54920080409066	-1.36183785137630	-2.49905145075617
N	0.55128791418212	-2.20690284836951	-2.74806529881998
C	0.52487784194285	-3.24996100221818	-1.81195966181985
C	-0.33738155787898	-4.34192153967254	-1.72045727915279
C	-0.10252304828795	-5.23612208365019	-0.67073558922534
C	0.95115929213897	-5.03242172540838	0.24041542660444
C	1.80139089627079	-3.92433904044161	0.13311724059707
C	1.58675289963995	-3.02821791072343	-0.91573315371521
H	-1.80542896643540	-0.04387147258374	-0.55104342620013
H	-0.36706323914463	-1.55818397194066	0.88529056716647
H	4.20035653129013	0.08755137001228	0.52838727878687

H	4.38739849969614	-1.57013208608774	1.14401409171773
H	4.23771951813205	-2.36463996367991	-1.07453729029629
H	4.05006340803189	-0.69218630530980	-1.64474906524577
H	1.76581242879950	-0.52297124683635	-3.15410984936126
H	-0.08274270013520	-2.12902140103327	-3.53985654271391
H	-1.15315291255608	-4.48683106531488	-2.42959318719090
H	-0.74743080270828	-6.10931574108252	-0.55834705200117
H	1.10743631381364	-5.75403523366697	1.04412940119739
H	2.61086787017530	-3.76836147456006	0.84712832227570
H	2.79930201044701	-1.32028010959392	3.04836200334712
H	1.13679363021257	-0.78019004546211	2.69165649828823
H	2.47281377966526	0.40448131928522	2.69294156056158
H	-4.65005854749319	3.29911565538406	-2.78747167761115
H	-4.32486483657760	3.49011374470812	-4.54892051036171
H	-5.99543950394479	1.00351466270523	-3.80734252626129
C	-4.00726744595373	2.99342265078876	-3.62335481238551
H	-5.26249433730206	0.72972110271563	-2.21953553713641
C	-5.08703168374674	0.65189679493820	-3.30140891502945
H	-5.54163426307974	0.50644274232794	-5.82643799127355
H	-4.76212017341599	1.98990900263974	-6.41412189624290
O	-2.45280234534929	4.53443159803463	-2.04367307156107
C	-4.58574685231647	0.93718552432496	-6.14439208274576
C	-3.86333006167143	1.48989676907270	-3.71942301381069
S	-2.37033202642939	3.65407811183360	-3.21523039421239
H	-5.42471618354665	-1.20847006553534	-4.44187677100460
C	-4.68262532463292	-0.78118205276177	-3.75469656460762
O	-1.69159344099442	4.16434835715653	-4.41074969493001
C	-3.47434982561653	0.83933977371001	-5.09231982349751
H	-4.27134857776007	0.41228287477488	-7.05866685161422
H	-3.14401550021043	0.91843508593254	-1.74598087812942
C	-2.72462323460537	1.04200099702163	-2.75512275291504
H	-4.58736817176695	-1.47328844766022	-2.90587709919615
N	-1.73373500884822	2.11742611250033	-2.73553892625566
C	-3.32121809667484	-0.56499801518927	-4.45379552341216
C	-2.19375031972035	1.35671614549759	-5.75758134466820
C	-2.26780451017807	-0.31091651657472	-3.35978922365413
H	-3.04257459157154	-1.36762958095992	-5.15143141467396
H	-1.25936327017981	-0.21380476525907	-3.77893647557916

H	-2.24865333485418	-1.11039220069498	-2.60804583216454
H	-2.35459350991448	2.36078046156390	-6.17172604971651
H	-1.93167171890710	0.68632589856768	-6.59039313878249
H	-1.33276142758171	1.41908612341888	-5.08578408339918

Inter4

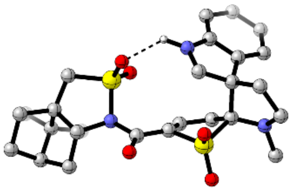

Electronic energy -2342.87472

Number of imaginary frequencies: 0

O	-0.30974890821799	2.60724057106427	-1.29765874522900
C	-0.42584702186638	1.60722843326496	-0.58411488670045
C	0.60898416187616	0.97603313446011	0.15597548978283
C	0.55838070693851	0.18792097475693	1.35900886143770
C	1.74959823290985	-0.28711081561670	1.82350448830151
C	2.88917039930580	-0.06675054319741	0.90163644467109
S	2.27679902337536	1.52903908953719	0.00266192866381
O	2.72440219042079	1.63706357015755	-1.41186482081458
O	2.59697664119353	2.68821926044909	0.87175284434678
N	4.19910972011509	-0.03008420252869	1.40838232922359
C	4.53411071852166	0.79541172606926	2.55371317359156
C	5.16000400519517	-0.08063540040616	0.31338852125757
C	4.43910810460758	-0.83619868296690	-0.82351345055490
C	3.03320028135693	-1.17152208400266	-0.27013821085443
C	1.88524301833090	-1.12827549153552	-1.22043393572417
N	1.13636240638786	-2.19328550860990	-1.07997020228181
C	1.65584594569607	-3.08022244342362	-0.11324363362741
C	1.14166865633968	-4.29500534007999	0.32500238033645
C	1.89475946286633	-4.97865714060471	1.28952946386173
C	3.10083681337381	-4.45014428678349	1.77593111761754
C	3.59250619064976	-3.21527013832474	1.32166138174811
C	2.85658264511594	-2.53062646856489	0.35994836238676
H	5.44249310388085	0.93486819321414	-0.02105819730201
H	6.07775187633241	-0.58732633438863	0.64792661150279

H	4.95601227973972	-1.76067937611770	-1.10669677197279
H	4.34507381076367	-0.20189323933345	-1.71063145706054
H	1.68687828998483	-0.38503725314205	-1.98615057255611
H	0.24423540523607	-2.28575796498846	-1.57900007951813
H	0.20035672474875	-4.68808718649719	-0.05987581556196
H	1.53680164445556	-5.93831902617792	1.66569514693522
H	3.66579159798374	-5.00932112332071	2.52362466392126
H	4.51981446376371	-2.79986518708884	1.71653349263986
H	5.45767523034997	0.41333403901442	3.01268510662235
H	3.72716913972161	0.73889152654857	3.29450958114193
H	4.68547136849711	1.85336844205204	2.28246119259713
H	-3.73682210894617	-0.74037868293125	-2.31469707026059
H	-4.18488637722823	-1.34321941066528	-0.67737071771240
H	-6.01816282314227	0.86061594278509	-1.96563761723480
C	-3.68625505138882	-0.54107627484095	-1.23644498477049
H	-4.72804790054361	1.76618470861098	-2.77149758388230
C	-5.16482278751644	1.52889105404063	-1.79165786726510
H	-6.81506495334884	0.74536789569755	-0.00067815256193
H	-5.98352087774535	-0.60159120842856	0.80160936870954
O	-1.15115734428308	-0.97257615300373	-2.06783906583704
C	-6.07775994543274	0.49499355067853	0.76995368182787
C	-4.11714067618669	0.86571363594423	-0.87590732795701
S	-1.91994284851659	-0.64794086020665	-0.84157741284122
H	-6.64542974056037	2.80511329446081	-0.76615629049677
C	-5.56856388545632	2.79467587024879	-0.98032553440555
O	-1.65990107896613	-1.50737528390924	0.32198723822830
C	-4.70125948375224	1.13594875795839	0.55291859789051
H	-6.48499812203332	0.81724927546146	1.73973788158074
H	-2.67265183210131	2.10398519425583	-1.97607076249535
C	-2.87386749026979	1.78829616682197	-0.94207103314000
H	-5.33331993815740	3.72456097844310	-1.51692372261243
H	-3.71372846462247	-0.39041459152645	1.75247971696083
N	-1.73063029603831	1.00514124567378	-0.45157214542882
C	-4.73567620275560	2.67009403011241	0.31520780794293
C	-3.82598451533924	0.70346395285044	1.73567520105760
C	-3.26943747305738	2.98691179217043	-0.04564225454843
H	-4.32067576195635	0.99958614848906	2.67293331862996
H	-5.12292909875312	3.26446133923600	1.15511473729432

H	-2.82215797018733	1.13663351367748	1.72789699849115
H	-3.17008103092574	3.93879484209483	-0.58379941823470
H	-2.62581163039893	3.03695961540191	0.84153637748696
H	-0.38878352982066	0.00689340342104	1.86667490641873
H	1.87152266628047	-0.87104008791087	2.73458228483619

Inter5

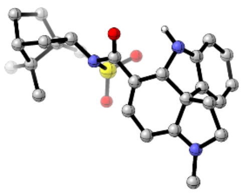

Electronic energy -1794.83758

Number of imaginary frequencies: 0

O	-0.32765256621556	0.71179395364127	-2.11106500930107
C	-0.39500086098013	0.43876667634917	-0.90803915226678
C	0.76487262645981	0.18509959690952	-0.08280219449935
C	0.76573392315520	0.41704971341776	1.27662760490045
C	1.90936708848881	0.28053892400162	2.11222481886831
C	3.06189688552157	-0.22984169502144	1.55133902608209
N	4.27985508174326	-0.39110182049383	2.09185730453828
C	4.61781208593569	-0.09404518403048	3.46613970377275
C	5.29140389866309	-0.79938205905749	1.10910119288888
C	4.58088136003440	-0.57232143616072	-0.22850827674315
C	3.09068654794230	-0.75689494874917	0.13773412134729
C	2.06401595902132	-0.11906597686024	-0.82273881353959
N	1.90611701523192	-1.16918081844986	-1.84645089115484
C	2.01032046664820	-2.38970260060574	-1.18484800836676
C	1.57957693437791	-3.65731377081056	-1.59074215672713
C	1.82636411038064	-4.74544732811218	-0.73948831124462
C	2.48673336736790	-4.58081839872970	0.48545841061562
C	2.92478431501147	-3.30457407140563	0.88249207894382
C	2.68282803253343	-2.22317363039408	0.04361657577674
H	6.19574860203143	-0.18428984036456	1.22194143710154
H	5.57368264598064	-1.85338835229037	1.26645967666021
H	4.91346752975084	-1.26020445695636	-1.01546117883442

H	4.74311963115291	0.46191509945592	-0.56710926853737
H	1.10767801803848	-1.03793365360598	-2.46184349418082
H	1.04838979502060	-3.79149551987105	-2.53479966036456
H	1.48533918259012	-5.73963139738106	-1.03681200651780
H	2.65718175918779	-5.44204063319807	1.13362200688695
H	3.43286450351842	-3.16464667460010	1.83933762006149
H	5.49391887140161	-0.68673184333664	3.75708763133702
H	3.78104180860202	-0.36077822132896	4.12509440836818
H	4.85082807987226	0.97470625831479	3.60300716248580
H	-4.04538471301860	-1.79802393482021	-0.47770037933128
H	-4.37998328268084	-1.17254346433170	1.17873610794801
H	-6.13943451989890	-0.01661949577808	-1.10729138853737
C	-3.86178728939523	-0.97981390584196	0.23091317232779
H	-4.89927797599167	-0.06324589198286	-2.37009512216489
C	-5.21367244502662	0.45939162659652	-1.45609907284017
H	-6.66449640546063	1.29775769746808	0.48407915423880
H	-5.85539217722220	0.64458598224484	1.92229574324533
O	-1.47843130339037	-2.23308079681775	-0.09063881707424
C	-5.85024781769713	1.47206493082606	1.19599737320531
C	-4.11135784366648	0.38194153655073	-0.38041718790251
S	-2.08452097469200	-1.07450791935286	0.58183930285911
H	-6.41630899848668	2.31032522573953	-1.45498520027653
C	-5.38461440509976	1.99318423480912	-1.65704906296491
O	-1.83055396859535	-0.91890375357584	2.01989422073589
C	-4.46610913605849	1.59436082438665	0.54720554515553
H	-6.08120836957114	2.39499059091855	1.74838379760612
H	-2.74129127920637	0.37963620024537	-2.09167965597615
C	-2.81407525818184	0.83525360245912	-1.09365953687781
H	-5.14113066169783	2.30750410456820	-2.68179471551775
H	-3.49128454018592	1.09693073490415	2.42957888421357
N	-1.67501664936715	0.37277109819399	-0.28786907169155
C	-4.39593458705647	2.59848327540096	-0.63595436560387
C	-3.47634872088076	1.89842905497425	1.67802391145668
C	-2.97085973881156	2.37302055032864	-1.18248046815982
H	-3.78168722274672	2.83069648794458	2.17704394369387
H	-4.60847414238466	3.64461095133290	-0.37250807918995
H	-2.44166255617125	2.01397405179674	1.34254548046406
H	-2.86063392623412	2.72810139395618	-2.21571232812718

H	-2.20909259614507	2.87577150696687	-0.57347410053455
H	-0.15623215432264	0.76915737822594	1.73914606375962
H	1.87237005835450	0.61166659872116	3.14944458608511
H	2.45579646482159	0.80005798446634	-1.28825130758143

SO_2

Electronic energy -548.09523

Number of imaginary frequencies: 0

O	1.05615888163100	0.03526215895022	-0.03875746040177
S	2.52396677001908	0.10450292693827	-0.11486160154546
O	3.11019111564991	1.01268898671151	-1.11307005695277

Indole

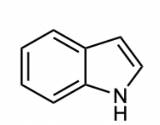

Electronic energy -363.189930913646

Number of imaginary frequencies: 0

H	1.30687382709994	-0.15952738657448	-0.02868986113612
C	2.39417025983428	-0.14054748559278	-0.02888753075840
C	3.22537669247292	-1.24118013492485	-0.02895806312182
N	4.53912032633724	-0.82980617202224	-0.02922568715281
C	4.58975261296108	0.54395007642876	-0.02933348294536
C	3.24273709795927	1.02047958680720	-0.02912504432886
C	3.01310011186630	2.41263482418705	-0.02918565865633
C	4.10443982240850	3.27737567036160	-0.02944649374755
C	5.43056720462370	2.78284248442999	-0.02964900205004
C	5.69143889461197	1.41453612626956	-0.02959638843865
H	2.98146095978681	-2.30119621293395	-0.02883730893677
H	5.34260739934497	-1.44669653295867	-0.02932489819400
H	1.99343780231232	2.80439283764951	-0.02902993152933
H	3.93876746946842	4.35662327677748	-0.02949546473809
H	6.26478449009581	3.48742499266673	-0.02985178400325
H	6.71314228231643	1.03028321372907	-0.02975340766264

TDO 9a

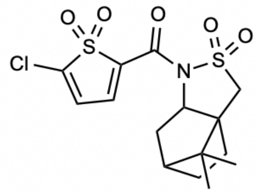

Electronic energy -2267.19314078595

Number of imaginary frequencies: 0

O	-1.95098896743387	2.58949168933545	0.62811013467412
C	-2.00041098915005	1.42084259205573	0.26291683361200
N	-3.21313542563368	0.79507001210055	0.03112728318592
C	-4.43367045787044	1.57810223036762	0.28747889338698
C	-4.75331012642911	2.70203601730894	-0.72785140951976
C	-6.18263030809969	2.34159604613412	-1.18029012982200
C	-7.12085083569983	2.52384483929819	0.03373407931809
C	-6.78350354146548	1.30223009287273	0.93709849577933
C	-5.64308291673859	0.61657689807515	0.15740995994920
C	-6.10493400214587	0.79864351804581	-1.33048370364204
C	-5.12505389467931	0.33886004819769	-2.41607782021023
C	-7.44712662426552	0.12218333262880	-1.63356776256662
C	-5.22608626277895	-0.75122243623024	0.65620156401302
S	-3.45872963355044	-0.92052824319941	0.31374716015179
O	-3.22268005468050	-1.66532432042049	-0.92737980137994
O	-2.70280812059558	-1.31039121026558	1.50609744490074
C	-0.75646255999539	0.66450683243226	0.03538909670319
C	-0.42120966791276	-0.39421195835094	-0.73485128774705
C	0.98528383447464	-0.81460725844983	-0.65887977233373
C	1.72415520948943	-0.06400017443006	0.17757334966188
S	0.71140875921145	1.26466434783533	0.89384256597926
O	1.18276104623162	2.54594228627720	0.36287770497079
O	0.56859887000125	1.08419283911623	2.34090328581213
H	-4.36156837743245	1.97250716285704	1.31178179569656
H	-4.04150016006143	2.69112203164020	-1.56205895724596
H	-4.69911663784681	3.69066079215194	-0.25428952660348
H	-6.50685230471503	2.87925108188859	-2.08241962371893
H	-8.17606250527351	2.51724825922050	-0.26925017665305
H	-6.93292156636775	3.47978524633899	0.54244585067747
H	-7.63985448413418	0.62572294457606	1.05409682095170
H	-6.44938038695683	1.58748304017496	1.94412498777302

H	-5.48124253598903	0.70655009171358	-3.39033549713382
H	-5.08890464782774	-0.75744530217685	-2.46444755135077
H	-4.09986789369817	0.69443226027932	-2.27480989345623
H	-8.26417689814266	0.43378385512537	-0.97380388704420
H	-7.34774546133609	-0.97135177474267	-1.55478994276407
H	-7.74706094576014	0.35084518389210	-2.66685321996666
H	-5.71716165900667	-1.60198829358295	0.16606989926136
H	-5.30302820065337	-0.84077419914459	1.74803605701934
H	-1.13242033235809	-0.92803410931002	-1.36389154507230
H	1.37147122843937	-1.65789732624218	-1.23005594101599
Cl	3.36817789783743	-0.15647800229468	0.59016908306898

TS1

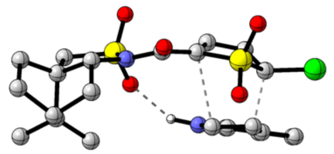

Electronic energy -2630.39034389692

Number of imaginary frequencies: 1

Imaginary frequencies: - 401.5cm^{-1}

O	-2.07632094994640	1.98880033051248	1.34928115323027
C	-2.14559809378646	0.97385963145183	0.65843938534061
N	-3.40066695064611	0.55558197759032	0.16218469640014
C	-4.54355951499378	1.44937868251578	0.41280104905547
C	-4.56021763870475	2.75199951212434	-0.42334464876704
C	-5.91640841517389	2.66449103334840	-1.15296849061909
C	-7.02744467223331	2.79793636933356	-0.08768210570605
C	-6.98754916701934	1.42687403647022	0.64798315924910
C	-5.81948792716961	0.70539399595465	-0.05379825113362
C	-5.99138848286814	1.16342691906889	-1.54336863521783
C	-4.89806059397162	0.72240110991088	-2.52418160746821
C	-7.32969586909206	0.73203272899461	-2.15512484784567
C	-5.65260703584469	-0.76666246719441	0.25982972178646
S	-3.87503331032249	-1.10546146182472	0.21135637243593
O	-3.48800537967738	-1.80236103767926	-1.02174677796911
O	-3.41704707789077	-1.70768874913631	1.47862228992192
C	-0.98636758267989	0.19995543183388	0.28616204704184

C	-0.70765128728978	-0.60643490806172	-0.81930264144870
C	0.59727207748274	-1.11352763583879	-0.90016002696552
C	1.36581871292588	-0.82647181834261	0.24800258088039
S	0.56180481272640	0.64568083336214	1.04699084088245
O	1.15478496935237	1.84716557549938	0.43581412861103
O	0.54653335200659	0.57225329219032	2.51792920661017
C	1.03190676416534	-2.18875450424020	1.71316082469959
C	-0.39267052326818	-2.14749490778958	1.84886190823190
N	-0.92900390109898	-3.08466037118716	1.06265448046517
C	0.06325993319801	-3.89387097493495	0.49889716907461
C	-0.06009503524920	-4.99919107600838	-0.34351160295166
C	1.12505991477526	-5.63697333357187	-0.72185307244061
C	2.37825228087664	-5.18173422333098	-0.26835224862607
C	2.48485827447744	-4.06790957140603	0.57161804732234
C	1.30962200538198	-3.42219201237056	0.97007019469580
H	-4.58592555334275	1.66070832671132	1.49109174830158
H	-3.71539979993771	2.77769970308442	-1.12251469825346
H	-4.48754894113884	3.63824617876963	0.22023049844979
H	-6.01234349644401	3.36882190858393	-1.99147553803155
H	-8.00779575623142	2.97557191569281	-0.54887541470750
H	-6.82875372833316	3.63892929283519	0.59140992830362
H	-7.92413115082930	0.86699765424679	0.52876621926356
H	-6.79419271617708	1.52013011722702	1.72547294819518
H	-5.06884013459866	1.21826992761518	-3.49165739868694
H	-4.94615493815122	-0.36236873240091	-2.69304174910079
H	-3.88175411488749	0.95921729385007	-2.19627831697880
H	-8.20668647477457	1.04115749898775	-1.57597625589453
H	-7.36289640123465	-0.36364734668559	-2.25885050110748
H	-7.42737115994344	1.16021255842288	-3.16369890591534
H	-6.12055482500930	-1.45807808136462	-0.45271453485905
H	-5.95000219752706	-1.01553135003871	1.28711578029004
H	0.97014506742000	-1.71355798562139	-1.72825145499894
H	-1.01339775587124	-1.49236332548908	2.44941543245273
H	-1.93647029714464	-3.18140719108886	0.93945500812770
H	-1.03492061358985	-5.34327727760015	-0.69094630971809
H	1.07660179201805	-6.50821443054153	-1.37743807294772
H	3.28158265247779	-5.71032119884017	-0.57748834800763
H	3.45896273371670	-3.71798529784369	0.91485276034567

H	1.69059316642142	-1.74017861470586	2.45500345147354
Cl	3.10159252745650	-0.74558116762071	0.17593324669282
H	-1.46332157268447	-0.82750278302968	-1.57234382186441

TS2

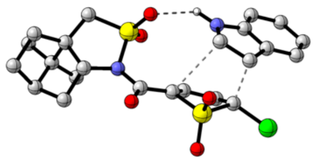

Electronic energy - 2630.38618819046

Number of imaginary frequencies: 1

Imaginary frequencies: - 411.5cm^{-1}

O	-1.60333440096813	2.57371613272880	-1.89461818497207
C	-1.73795123095126	1.68811942979193	-1.04865600435487
N	-3.03953548817356	1.30734191144587	-0.69205867738679
C	-4.17545943659987	2.02144828964222	-1.30136815924151
C	-4.27792850945218	1.96287613193811	-2.84698907703106
C	-5.69051523956638	1.39041511520638	-3.06891743075917
C	-6.71116051611174	2.43522460613174	-2.56416002192445
C	-6.59539606184023	2.33495467665976	-1.01520052937556
C	-5.46866516007197	1.29896107591647	-0.83451378534149
C	-5.76625311636929	0.27991126261179	-1.98921473530728
C	-4.74457372159936	-0.84727964725839	-2.18725148508463
C	-7.13965184099632	-0.39033620258042	-1.86397570875049
C	-5.25175838226595	0.77456209754492	0.56877929839697
S	-3.48255247750709	0.44959296875455	0.72995437925098
O	-3.17663895426867	-0.98366891111773	0.57956680060093
O	-2.93169932503948	1.12489961425912	1.91268161695478
C	-0.58455171482336	0.95675083103634	-0.55642248623035
C	-0.21536077383466	0.30922123606114	0.62495226308078
C	1.04120347981942	-0.31232857478931	0.61273524706558
C	1.63850254841999	-0.30659810423753	-0.66738014124239
S	0.88710811756073	1.13609434348565	-1.55904977128919
O	0.66307569324748	0.86752367224728	-2.98990756610785
O	1.67760788552654	2.32758941993668	-1.20844246086587
C	0.89820829411030	-1.77648728522907	-1.80991810424541

C	-0.51685810763798	-1.54553667347263	-1.73277883939304
N	-1.01665258866919	-2.26345452935383	-0.72062282035438
C	-0.04392658630893	-3.11631361582608	-0.18710074979294
C	-0.14882013092057	-4.04710462432940	0.84669335322898
C	0.99463915084842	-4.79848357090770	1.13589742515945
C	2.18993020416724	-4.62084467310029	0.41329777493260
C	2.28074096099649	-3.67587739153852	-0.61542674361347
C	1.14439589343285	-2.92316542662588	-0.92594248198778
H	-4.15853443522027	3.06083468215065	-0.94070453307061
H	-3.50255583915168	1.31460971699402	-3.26915576969523
H	-4.15092991306968	2.95954791765739	-3.28791480774061
H	-5.86963680814466	1.05042185663222	-4.09889491391575
H	-7.72865796708460	2.20217083437433	-2.90450007338419
H	-6.46579804813279	3.44085791232665	-2.93370542315146
H	-7.52788085981254	1.98649394693387	-0.55279922074697
H	-6.32764557682415	3.28796390611268	-0.53854145073211
H	-3.70484024992902	-0.50978954984757	-2.23292163613552
H	-4.97331374660457	-1.36989765213871	-3.12841244471381
H	-4.81835171168800	-1.58124551955714	-1.37313961642712
H	-7.17053217575845	-1.03272019610133	-0.97028958675881
H	-7.31224739493324	-1.03598800150166	-2.73767150482356
H	-7.97621481613523	0.31415156903347	-1.80137861612315
H	-5.76407021949263	-0.16762781469326	0.80339034415709
H	-5.46185827892833	1.52633825498551	1.34097625209811
H	1.48322443647942	-0.81170391258514	1.47290510199628
H	-1.95571818450683	-2.11779119244416	-0.33871453487676
H	-1.07851500134792	-4.17596741187828	1.40217963008303
H	0.95680969185726	-5.54141359628934	1.93461127818604
H	3.05962527963851	-5.23166382516617	0.66121444007999
H	3.21206015135378	-3.53556715856363	-1.16516254331830
H	1.46475070318522	-1.55379413712996	-2.71290628732100
H	-1.14619151560974	-0.92900669669654	-2.36571361552777
Cl	3.36203479313133	-0.45342255750705	-0.85890380492074
H	-0.85364077732290	0.28821503936738	1.50631114316456

TS3

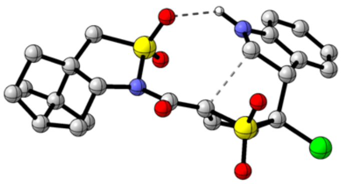

Electronic energy -2630.40249739315

Number of imaginary frequencies: 1

Imaginary frequencies: -39.9 cm^{-1}

O	-2.21313788213851	1.98112324106860	1.61742655392220
C	-2.20680133019355	1.00850560987378	0.86161195584187
N	-3.43755189300452	0.56657732032092	0.29636996777001
C	-4.61320580873689	1.42829914117897	0.48865688053828
C	-4.58898237577773	2.75080194479568	-0.31594124064521
C	-5.86913558216574	2.64953010587320	-1.17034829997537
C	-7.07586082705938	2.73576026126025	-0.20889674688686
C	-7.06897387541713	1.35171300365132	0.50417991686748
C	-5.82792214403387	0.66974591888391	-0.10470061473526
C	-5.87474605044545	1.15583599936110	-1.59389619807146
C	-4.68883660465915	0.74777977249409	-2.47702084945810
C	-7.14351905470802	0.70923661304222	-2.33078610665829
C	-5.65462847877258	-0.80911590774109	0.17509681055569
S	-3.87399373618916	-1.09162779392697	0.35704556165268
O	-3.30199694852853	-1.83052039924296	-0.77521935699908
O	-3.58752345066431	-1.63034695664692	1.70599784135817
C	-1.02825591789483	0.28805880493796	0.47365394036745
C	-0.70220849491582	-0.33926994328336	-0.78564218156601
C	0.57197890470856	-0.80944810858068	-0.87854982324590
C	1.30435418023643	-0.68564045930953	0.40644945504531
S	0.51629718668429	0.84766304310921	1.15351397919834
O	1.06440023403801	2.04729133136110	0.50008522528595
O	0.56413182798379	0.79168065213469	2.62858213662357
C	0.95885563605195	-1.83003221742977	1.43871416115594
C	-0.49240512410311	-1.77821162155737	1.84754071389861
N	-1.10693098504943	-2.85359975964117	1.39809544999198
C	-0.23583507247305	-3.72397987048700	0.71126445115838
C	-0.52560279762534	-4.93299168065085	0.08968296003918
C	0.55521926433129	-5.61816373405500	-0.48384072229136

C	1.85667648532002	-5.09946091492009	-0.42436271873194
C	2.12222649558089	-3.87199052836807	0.20592078913716
C	1.05976153155774	-3.18762342204402	0.78615722636979
H	-4.74923597307604	1.60950269319166	1.56495181058923
H	-3.68394015964227	2.81485261256875	-0.93251357793458
H	-4.59981434663989	3.62293966064153	0.35059629032126
H	-5.90464230306664	3.37044297931584	-1.99951790550486
H	-8.01415750000601	2.89906721938557	-0.75516449140778
H	-6.96127485205027	3.56826600396344	0.49948446493141
H	-7.97907036014450	0.77487207960157	0.29473190060371
H	-6.97288405286430	1.42935045560907	1.59597392083872
H	-4.80841012168311	1.20809159511560	-3.46932361841049
H	-4.67080925208281	-0.34273910353633	-2.61873483459279
H	-3.71398231565142	1.04392591185184	-2.07983189437206
H	-8.07508401966461	0.97436634208609	-1.81896534347735
H	-7.13534603575369	-0.38285901304059	-2.47117536672369
H	-7.16876712228795	1.16794856274662	-3.33027489246683
H	-5.99900345997524	-1.48117163941565	-0.62147133960360
H	-6.07756761833960	-1.11431542741794	1.14090597693380
H	1.02003171757953	-1.27592725229595	-1.75372085405850
H	-0.93166050472967	-1.14885377870392	2.61322863602378
H	-2.10885248061027	-2.99688021543446	1.55283069749983
H	-1.54281198543267	-5.32348751236017	0.05003691864640
H	0.37818334423698	-6.57289143047262	-0.98160832558065
H	2.67819081425677	-5.65910345825661	-0.87419340806973
H	3.13502002087654	-3.47037935092341	0.23909117888461
H	1.60527147777477	-1.67294872934910	2.31511574479679
Cl	3.06261549154842	-0.48958813500507	0.25540228622204
H	-1.43185007330769	-0.40901165272770	-1.59173898900189

TS4

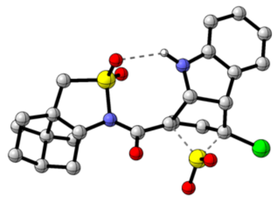

Electronic energy -2630.420333264291

Number of imaginary frequencies: 1

Imaginary frequencies: -217.4 cm^{-1}

O	-1.53546307930070	2.69444549241760	-1.76611261218499
C	-1.71792624169764	1.88717199441061	-0.86605046731356
N	-3.00075048660722	1.49148548059866	-0.53315317614974
C	-4.14513678886831	2.02157182685676	-1.29377653764869
C	-4.13656935608686	1.72534319975829	-2.81481413034382
C	-5.46901660630799	0.97837210763409	-3.02016120206714
C	-6.61575396476877	1.97837944968315	-2.75142377230171
C	-6.59585949907281	2.14417758287819	-1.20358133815267
C	-5.40185137550546	1.26003774202313	-0.79109858077538
C	-5.52455097571244	0.04925545549172	-1.77962636069889
C	-4.39982102468838	-0.99149888416421	-1.72310032248106
C	-6.84052805380199	-0.72244599817266	-1.62180339181919
C	-5.25193567546211	0.96917959208223	0.68882139852853
S	-3.47871845565915	0.93564658151763	1.04527066912063
O	-2.95460339938507	-0.42248710570925	1.24820294663845
O	-3.14277148128408	1.95998958707583	2.03952192104728
C	-0.53883645633753	1.18457298030429	-0.23955149584812
C	-0.20616932809100	1.23070350510020	1.14820030248936
C	1.09958309249160	0.90318830333320	1.46512521059774
C	1.94385090702135	0.57107172374080	0.36773862315933
S	1.25629829858265	2.33242640690746	-0.89700765945202
O	1.61270650376413	2.25278046000684	-2.32711130936754
O	1.32944635181095	3.64672397324423	-0.23676462120463
C	1.41603449212323	-0.40921962611191	-0.66882845523740
C	-0.08316437624293	-0.06477186347292	-1.01219723119526
N	-0.85853545315448	-1.25762795775173	-0.65500515975021
C	-0.02158152698664	-2.21242486570734	-0.08434189508685
C	-0.38820026651574	-3.45861070334989	0.43737730729439
C	0.62517940459408	-4.30395414177428	0.90896673947865
C	1.97385127733348	-3.92718335549071	0.84921536178415
C	2.33129394409094	-2.67254142425012	0.32950482640692
C	1.32689772914967	-1.82156185588920	-0.12369625608384
H	-4.22782893074948	3.09893163528283	-1.08628639156075
H	-3.27460621479435	1.10845814334994	-3.09266621846774
H	-4.08036903761559	2.65363372084680	-3.39708160630890
H	-5.53823498680030	0.46645906354967	-3.99031650489497

H	-7.58050833362462	1.58555796994894	-3.09798350503850
H	-6.44382765417731	2.93034721010636	-3.27288483851316
H	-7.52519244543725	1.79216390997103	-0.73796904615795
H	-6.43996592261961	3.18301103967113	-0.88191094910272
H	-4.57086265056145	-1.74162582521043	-2.50955225314266
H	-4.40852571892980	-1.52022902601449	-0.75892704415343
H	-3.39600724939931	-0.58385206536591	-1.87022973106978
H	-6.85548051799583	-1.25473180444370	-0.65815807889055
H	-6.91678922929898	-1.48013416635660	-2.41544822383265
H	-7.73657612777320	-0.09463916074907	-1.67274137056640
H	-5.65131936519300	-0.00020798334293	1.01439135619556
H	-5.63639634383389	1.77456436962184	1.32779705489481
H	1.51639458624381	1.10893471547455	2.45180789624709
H	-1.43620205300631	-3.76123779386407	0.46838986547697
H	0.35538248458493	-5.27850919841333	1.32134218340070
H	2.74737727113998	-4.60651615942817	1.21071063864386
H	3.37745889296411	-2.36563610302047	0.29477326059298
H	2.04772843378375	-0.34570879509153	-1.56551131810446
H	-0.17906716186306	0.14795738328215	-2.08685390439695
H	-1.73898020058178	-1.09467345161440	-0.16897360729182
H	-0.85306862618378	1.70994363674874	1.88176903256029
Cl	3.66799883729815	0.53363302644039	0.62730050059857

Inter1

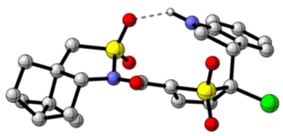

Electronic energy -2630.40256805221

Number of imaginary frequencies: 0

O	-2.14013387272038	1.92446330017397	1.63096525405126
C	-2.12081562051866	0.97374893977768	0.84619881762818
N	-3.35270520854360	0.52556530189498	0.27408169397827
C	-4.53406455405164	1.37547082446567	0.48187241744861
C	-4.53456643478441	2.69618113579377	-0.32607580991802
C	-5.82595670184743	2.58029182067830	-1.16187362977630

C	-7.01944177387744	2.65881316707323	-0.18361564743801
C	-6.98848088564588	1.27778818428874	0.53462688729701
C	-5.75054859457599	0.60453235079775	-0.09037089731467
C	-5.82400870372667	1.08510958448861	-1.58037410552184
C	-4.64744286042126	0.68707177216857	-2.48030762641446
C	-7.09918157821127	0.62400550086351	-2.29701807590091
C	-5.55889429223407	-0.87098060916199	0.19472153688924
S	-3.77201503499941	-1.13526299820587	0.34373619363386
O	-3.21934540902124	-1.87105264803009	-0.80108272235373
O	-3.45224445763268	-1.67778530012216	1.68447939222170
C	-0.94745100913429	0.27402814679707	0.43129371018205
C	-0.63084332165861	-0.37665778833132	-0.81593908133864
C	0.62887039107156	-0.88631935252975	-0.90499318518082
C	1.36666173722249	-0.78445081994899	0.38004357769071
S	0.59718456948802	0.74811503756163	1.15256989646890
O	1.20294459682961	1.94468076095181	0.54478164380860
O	0.61618880382008	0.65109552360828	2.62719116936813
C	1.04639574967965	-1.95687211963726	1.38994664797194
C	-0.39836417322601	-1.93510053709834	1.80686084816966
N	-1.01076318929799	-2.98604818712981	1.31939007309716
C	-0.13527166868463	-3.83713331314807	0.60903004154367
C	-0.42216357866779	-5.02845926217722	-0.04621534011861
C	0.66152346875829	-5.69149177201440	-0.63911482683750
C	1.96070403203672	-5.16827980092267	-0.56368753999221
C	2.22232432604292	-3.95853943870943	0.10085753634975
C	1.15678679667233	-3.29525948512936	0.70017274713806
H	-4.65539106153118	1.55856626346110	1.55951891394169
H	-3.63883390339736	2.76740874485019	-0.95548330219852
H	-4.54488687571685	3.57006781142839	0.33828849807351
H	-5.87991954683886	3.29779832021261	-1.99309287313527
H	-7.96698300408394	2.81042190119554	-0.71724575077994
H	-6.90300900011327	3.49535852232830	0.51966627610081
H	-7.89636462547275	0.69173865500397	0.34188246184183
H	-6.87607626076951	1.36090486451109	1.62446247639726
H	-4.78680796939250	1.14390574857231	-3.47173162674992
H	-4.61976966655331	-0.40343275833946	-2.61955868888711
H	-3.67022803290378	0.99435148850619	-2.09783186024100
H	-8.02555782915436	0.87837626137175	-1.77062440108415

H	-7.08048864645450	-0.46795911202720	-2.43695080446227
H	-7.14566688631896	1.08184712056149	-3.29611464994239
H	-5.91230318263952	-1.55106211212145	-0.59097854246213
H	-5.96145814575752	-1.17332881623371	1.17013975611939
H	1.06272840460568	-1.36946682604971	-1.77825230200223
H	-0.86708937727705	-1.28642689056775	2.53776297792612
H	-2.01510469873292	-3.13005565641449	1.46245357506379
H	-1.43777134733379	-5.42163810270637	-0.09637108847967
H	0.48940957363161	-6.63211160198272	-1.16441802752290
H	2.78450294579102	-5.71144381010424	-1.02924895152258
H	3.23339130850278	-3.55410056585457	0.14522084517362
H	1.69733454035574	-1.81275479768389	2.26542237696152
Cl	3.12593145915826	-0.58395272159068	0.21791072791420
H	-1.35915971964167	-0.43248984901332	-1.62451761327463

Inter2

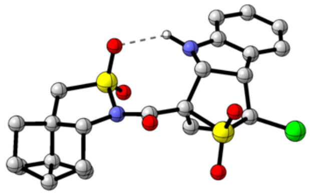

Electronic energy -2630.42843331772

Number of imaginary frequencies: 0

O	-1.48224094438771	2.27886463563303	-2.03985548246979
C	-1.66388699086433	1.41290770499082	-1.19677776245253
N	-2.94809254700717	1.11061650106205	-0.77812909249311
C	-4.07301037436107	1.82640227961645	-1.40891913550600
C	-4.23985361020288	1.59714081706035	-2.93154356506718
C	-5.67714883495256	1.04942661822570	-3.03279718301738
C	-6.64199420416408	2.18747021110525	-2.63052699928901
C	-6.46284725956888	2.28496038039374	-1.08727237418866
C	-5.37458549123418	1.22779421023424	-0.81288698699412
C	-5.75394007048520	0.08693916640311	-1.81835542946966
C	-4.78520619049998	-1.09949606246537	-1.89441162489224
C	-7.14621727330615	-0.50169246298940	-1.56004386199077
C	-5.13623212429080	0.86389503657135	0.63850525501652
S	-3.36288930231254	0.56616866500749	0.82404768672322
O	-3.02973399517180	-0.86034602225699	0.94465918774259

O	-2.78551179649003	1.49088951267539	1.80526705599032
C	-0.49498532373017	0.54751918738840	-0.76782792377207
C	-0.07289255634721	0.33520422217937	0.64877042792157
C	1.20816695712342	-0.08762629861237	0.73227484118510
C	1.84301137819815	-0.20995730906240	-0.60536644890168
S	1.12451424903039	1.35579237765731	-1.43057528323139
O	1.26117469977994	1.28042576976960	-2.89060728253031
O	1.47220836062056	2.59706144124377	-0.73425997251882
C	1.12246888060372	-1.26419543688872	-1.47665586719958
C	-0.38163472993301	-0.79868544915224	-1.57569191592826
N	-1.16092435910999	-1.89859279722775	-1.03081724558177
C	-0.32011426474147	-2.88232703430289	-0.52152195792727
C	-0.68293689030742	-4.04698929168149	0.16455333064913
C	0.33314385643968	-4.93813289081823	0.53757776251781
C	1.67644789440950	-4.68619739635049	0.22709909290890
C	2.03095953201490	-3.51124381406323	-0.45615923093713
C	1.02853588861803	-2.61661431646505	-0.81814460668409
H	-3.98137964887847	2.89453858940415	-1.16279768109449
H	-3.50075924566538	0.87920767152145	-3.30575682237248
H	-4.10511622181380	2.53274143276098	-3.48860225119341
H	-5.90374449430304	0.59187402733297	-4.00602676446371
H	-7.67956312527475	1.94776851996102	-2.89725300475882
H	-6.38255300622975	3.12701011594789	-3.13813898211006
H	-7.38771334896555	2.04482681083825	-0.54757190321782
H	-6.13368093656026	3.27785540132781	-0.75121508563627
H	-3.74692280387520	-0.82356487334220	-2.09911469733225
H	-5.11492298836052	-1.77616968362666	-2.69676783829851
H	-4.79957146363675	-1.67191568749925	-0.95553950257436
H	-7.15430298367634	-1.05178401481108	-0.60631682636678
H	-7.39243587584747	-1.22075226992929	-2.35501263773874
H	-7.94808894205938	0.24365276105066	-1.52470143320266
H	-5.63901035835957	-0.05097779823055	0.97856193653518
H	-5.33981634570848	1.69201272526635	1.32964940201269
H	1.75664582492615	-0.26112800835658	1.65743586514254
H	-1.99237697486039	-1.66858458284676	-0.48993680488539
H	-1.72851551589576	-4.25265682122819	0.39954183248770
H	0.06701257604291	-5.85052360956023	1.07545596187167
H	2.44799508175171	-5.39958530755669	0.52045850154165

H	3.07565887279728	-3.29650219165109	-0.68701437186856
H	1.59888214253599	-1.28112410048713	-2.46568015125556
H	-0.65659633920271	-0.58221226363485	-2.61792937216519
Cl	3.60240736327901	-0.29185351427741	-0.64181500310751
H	-0.69774380542713	0.56619451624591	1.50728022683961

Inter3

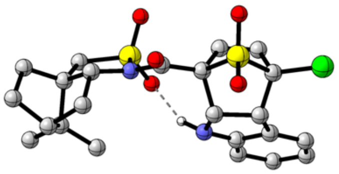

Electronic energy -2630.42803850249

Number of imaginary frequencies: 0

O	-2.10311916905650	1.42726009507237	1.67618970885282
C	-2.22392260160515	0.45676975647553	0.94201869608121
N	-3.45296831922342	0.15766610724423	0.37855378436576
C	-4.56472401961769	1.09248938644613	0.62753002010865
C	-4.38737152072424	2.50091740602558	0.00800419609255
C	-5.62609203384812	2.62537097581209	-0.90228940616107
C	-6.87074837605863	2.68890977089318	0.01106080237405
C	-7.01448245008939	1.22723389981313	0.52700308971809
C	-5.80132787329745	0.52659500178244	-0.11673969814239
C	-5.72971841814576	1.20819238495231	-1.52629720541358
C	-4.53589286945297	0.82052438688140	-2.40755116594820
C	-6.98922487389068	0.97483664931499	-2.36979556341264
C	-5.77272244452210	-0.98536812592606	-0.03760462905643
S	-4.04020226133816	-1.47136973389183	0.15498344037996
O	-3.47710686689135	-2.05187514988187	-1.06443998992458
O	-3.85760052310986	-2.18965195496959	1.42713217517557
C	-0.99345239440921	-0.32414479697356	0.53054604089979
C	-0.77243880940101	-0.63583558675381	-0.91223380319355
C	0.53366660282944	-0.83563854384733	-1.19364145542083
C	1.38737345403794	-0.67301253298892	0.01344718448821
S	0.52146539729208	0.82665645838954	0.81795601902412
O	0.50198464692964	2.02028777771747	-0.03183565504226
O	0.89952906265166	0.95214273245110	2.23180945130979
C	1.04235551257945	-1.71250806412904	1.10066107061113

C	-0.49892108558839	-1.53541014169230	1.40988065865343
N	-1.08562117867782	-2.79520186185392	1.02269719608085
C	-0.13489333115350	-3.71804326873038	0.64357260731543
C	-0.31815771588531	-5.05680802892892	0.27540615696938
C	0.81117761400613	-5.79938684196523	-0.09872063202368
C	2.09236358005451	-5.23201905713928	-0.10623536100051
C	2.26655765455543	-3.88965626886700	0.27300710420810
C	1.15244470446233	-3.14588395505246	0.64483226854419
H	-4.74354069100575	1.13900155408593	1.71196677646122
H	-3.45059873613188	2.56521811904495	-0.55869064959643
H	-4.35859955894726	3.27496760109995	0.78533339141976
H	-5.55741331183249	3.44974604724861	-1.62596562523286
H	-7.76259858151819	3.00481635371839	-0.54562234313204
H	-6.72617275185289	3.40575320340677	0.83138413452642
H	-7.95406586391757	0.76395492974628	0.19968922737042
H	-6.97329246595629	1.14870087484563	1.62207950853842
H	-4.55906317773691	1.42884452059938	-3.32397674878280
H	-4.60535307720137	-0.23421967500718	-2.71007400021414
H	-3.56111306081279	0.96623143180936	-1.93347366362990
H	-7.92250029706263	1.24987446558134	-1.86681539138508
H	-7.06142500507413	-0.08526222935305	-2.65818977306225
H	-6.92288810148040	1.56268346465790	-3.29705035577459
H	-6.14060841041481	-1.50632699011439	-0.93100433804312
H	-6.26259013030238	-1.37725739235645	0.86303594338502
H	0.95040582833473	-1.03509235243531	-2.18026433214298
H	-2.00725105480073	-3.05175773247071	1.35730886705988
H	-1.31292420019573	-5.50499858094292	0.27782223231151
H	0.68420240439147	-6.84462575011318	-0.38908273697394
H	2.95477448350429	-5.83192871118065	-0.40043122511415
H	3.26011427978722	-3.43847448250537	0.27939652788976
H	1.65191381473598	-1.49265021824993	1.98722648617679
Cl	3.11107716221698	-0.43439454237948	-0.26273310741095
H	-1.58181518830574	-0.65829788211384	-1.63854951691483
H	-0.66840564073248	-1.28800193570198	2.46828879105750

Product

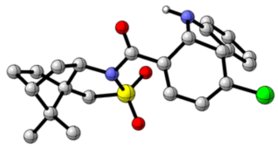

Electronic energy -2082.35356612243

Number of imaginary frequencies: 0

O	-2.06268118921431	0.53731442472695	2.06143221091886
C	-1.96391197490346	0.13485405915392	0.90674590901183
N	-3.06801049110406	0.15122400996363	0.05073350990182
C	-4.32267298596682	0.71173715068578	0.57002271891444
C	-4.37386243731965	2.25681849952209	0.65700517588593
C	-5.60195925911346	2.60132637830831	-0.21072610589128
C	-6.85431172625497	2.07153243013865	0.52237184233136
C	-6.75999747939553	0.53041082200826	0.32689725510152
C	-5.43785085998872	0.37101398360001	-0.45029173172572
C	-5.45847122831658	1.61621718051163	-1.40234177192915
C	-4.20211288628865	1.84973341660899	-2.24996012852213
C	-6.64643668219078	1.61271833543717	-2.37185645686040
C	-5.16051848429860	-1.00165985722900	-1.02580916482359
S	-3.36608876242728	-1.24668625699832	-0.96124857488274
O	-2.75925950519534	-1.07603359096174	-2.28417623529241
O	-3.02089862187619	-2.44791038054987	-0.19151062002234
C	-0.68575022606180	-0.35135997105421	0.35097860970664
C	-0.30988757140871	-0.04842803596745	-0.91656738075231
C	1.00293964907563	-0.39341247413467	-1.43718696803423
C	1.82322415791901	-1.17076831907565	-0.70226609829075
C	1.42969461879891	-1.74653719803672	0.62500481591081
C	0.28453180070301	-0.97509715506404	1.34035561768853
N	-0.33042598168098	-2.02534311138416	2.16754356856174
C	-0.17527846042441	-3.24105443711575	1.50915385704996
C	-0.85760345450308	-4.44514482952855	1.71399411527218
C	-0.49116965136523	-5.55506985942788	0.93892223391432
C	0.53389750971857	-5.47439035141760	-0.01296118343827
C	1.22227329097992	-4.26373216148065	-0.20418661280531
C	0.85849924729586	-3.15831219659015	0.55646211404689
H	-4.52920120161767	0.24459664559987	1.54457488358714

H	-3.45063895049162	2.69945385830893	0.26283829323328
H	-4.48884277287341	2.59369014839949	1.69552174120818
H	-5.66079904179164	3.66302053243745	-0.48951939863533
H	-7.77660409432668	2.47390481728671	0.08309838174257
H	-6.84605269722284	2.35679479091296	1.58372241152917
H	-7.60560571413573	0.13470840524048	-0.25030003997010
H	-6.72098805037914	-0.02404377893747	1.27463852810631
H	-4.30018939976210	2.81423076208512	-2.77082581601172
H	-4.10124150915021	1.06689640248470	-3.01423886101345
H	-3.27212081871718	1.86948699432502	-1.67402467126515
H	-6.66463820207024	2.55826850547141	-2.93382546444531
H	-7.62099209946204	1.49518857655839	-1.88550507372959
H	-6.53459111728289	0.79812916816623	-3.10401926971895
H	-5.45491192030454	-1.13930528147362	-2.07401902206109
H	-5.56905840064972	-1.80923136101143	-0.40419476056356
H	-1.24231946600386	-1.81034287490899	2.55964336021336
H	-1.66625851841263	-4.51130056494587	2.44397983320329
H	-1.02303901053040	-6.49868478292617	1.07874558683544
H	0.79783540634670	-6.35067684377483	-0.60725648006647
H	2.02416193785504	-4.19153831145730	-0.94084392686853
H	2.31371468653193	-1.75114882262220	1.28373576105946
Cl	3.38604355720049	-1.64729221050921	-1.29823821116337
H	0.67874819932071	-0.17602246336029	1.98706737761234
H	1.30461423076785	-0.03017688469387	-2.41963757001724
H	-0.98957420803012	0.49505065319562	-1.57180556664687

6. Crystal Structure Analysis of 10a

Figure S12. Solid state structure of **10a**. Displacement ellipsoid plots are drawn at 50% probability

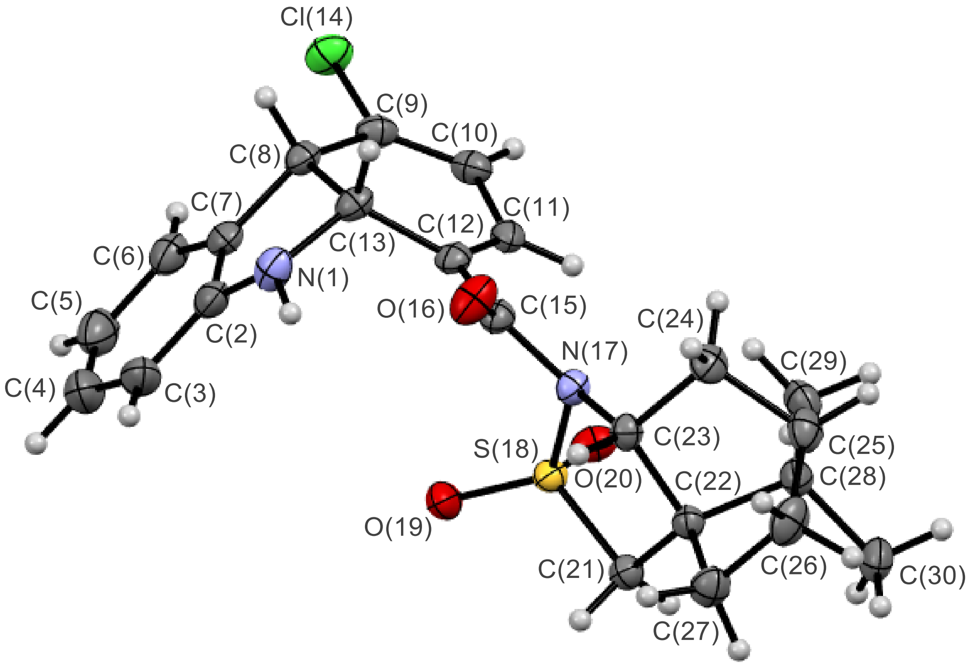

Low temperature single crystal X-ray diffraction data for **10a** were collected a using a Rigaku Oxford SuperNova diffractometer. Raw frame data were reduced using CrysAlisPro. All structures were solved using 'Superflip'[41] before refinement with CRYSTALS[42,43] as per the SI (CIF). Full refinement details are given in the Supporting Information CIF file; Crystallographic data has been deposited with the Cambridge Crystallographic Data Centre (CCDC 2408040) and can be obtained via www.ccdc.cam.ac.uk/data_request/cif.

Table S19. Crystal data and structure refinement for **10a**

CCDC identification code	2408040	
Empirical formula	C23 H25 Cl N2 O3 S	
Formula weight	444.98	
Temperature	150 K	
Wavelength	1.54184 Å	
Crystal system	Monoclinic	
Space group	I 2	
Unit cell dimensions	a = 13.2371(4)	α = 90°
	b = 7.9287(2)	β = 90.491(2)°
	c = 19.9752(6)	γ = 90°
Volume	2096.38(10) $Å^3$	
Z	4	
Crystal size	0.05 × 0.06 × 0.08 mm^3	
Reflections collected	15752	
Independent reflections	4152 [R(int) = 0.055]	
Completeness to theta = 74.798°	99.8%	
Refinement method	Full-matrix least-squares on F^2	
Data / restraints / parameters	4152 / 1 / 272	
Goodness-of-fit on F^2	1.0010	
Final R indices [I>2sigma(I)]	R1 = 0.0440, wR2 = 0.1110	
R indices (all data)	R1 = 0.0467, wR2 = 0.1151	
Absolute structure parameter	-0.013(11)	

Table S20. Bond lengths [Å] for **10a**

Atomic connectivity	Bond length / Å	Atomic connectivity	Bond length / Å
N(1)–C(2)	1.392(4)	S(18)–O(19)	1.4343(19)
N(1)–C(13)	1.480(3)	S(18)–O(20)	1.430(2)
N(1)–H(11)	0.838	S(18)–C(21)	1.792(3)
C(2)–C(3)	1.392(4)	C(21)–C(22)	1.515(3)
C(2)–C(7)	1.395(4)	C(21)–H(211)	0.959
C(3)–C(4)	1.393(5)	C(21)–H(212)	0.967
C(3)–H(31)	0.927	C(22)–C(23)	1.546(4)
C(4)–C(5)	1.376(6)	C(22)–C(27)	1.544(4)
C(4)–H(41)	0.939	C(22)–C(28)	1.569(3)
C(5)–C(6)	1.403(5)	C(23)–C(24)	1.548(4)
C(5)–H(51)	0.929	C(23)–H(231)	0.959
C(6)–C(7)	1.379(4)	C(24)–C(25)	1.545(4)
C(6)–H(61)	0.920	C(24)–H(241)	0.973
C(7)–C(8)	1.526(3)	C(24)–H(242)	0.966
C(8)–C(9)	1.497(4)	C(25)–C(26)	1.541(4)
C(8)–C(13)	1.564(3)	C(25)–C(28)	1.551(4)
C(8)–H(81)	0.978	C(25)–H(251)	0.972
C(9)–C(10)	1.330(4)	C(26)–C(27)	1.553(4)
C(9)–C(l14)	1.730(3)	C(26)–H(261)	0.967
C(10)–C(11)	1.448(4)	C(26)–H(262)	0.976
C(10)–H(101)	0.917	C(27)–H(272)	0.958
C(11)–C(12)	1.350(4)	C(27)–H(271)	0.965
C(11)–H(111)	0.935	C(28)–C(29)	1.527(4)
C(12)–C(13)	1.514(4)	C(28)–C(30)	1.545(4)
C(12)–C(15)	1.471(4)	C(29)–H(293)	0.964
C(13)–H(131)	0.968	C(29)–H(292)	0.954
C(15)–O(16)	1.221(4)	C(29)–H(291)	0.958
C(15)–N(17)	1.402(4)	C(30)–H(303)	0.957
N(17)–S(18)	1.712(2)	C(30)–H(302)	0.956
N(17)–C(23)	1.485(3)	C(30)–H(301)	0.965

Table S21. Bond angles [°] for **10a**:

Atomic connectivity	Bond angle / °	Atomic connectivity	Bond angle / °
C(2)–N(1)–C(13)	108.0(2)	C(22)–C(21)–H(211)	112.5
C(2)–N(1)–H(11)	117.4	S(18)–C(21)–H(212)	107.2
C(13)–N(1)–H(11)	118.3	C(22)–C(21)–H(212)	108.9
N(1)–C(2)–C(3)	127.3(3)	H(211)–C(21)–H(212)	111.0
N(1)–C(2)–C(7)	111.9(2)	C(21)–C(22)–C(23)	108.0(2)
C(3)–C(2)–C(7)	120.8(3)	C(21)–C(22)–C(27)	116.2(2)
C(2)–C(3)–C(4)	117.4(3)	C(23)–C(22)–C(27)	104.5(2)
C(2)–C(3)–H(31)	121.6	C(21)–C(22)–C(28)	120.3(2)
C(4)–C(3)–H(31)	121.0	C(23)–C(22)–C(28)	104.3(2)
C(3)–C(4)–C(5)	122.1(3)	C(27)–C(22)–C(28)	102.1(2)
C(3)–C(4)–H(41)	119.1	C(22)–C(23)–N(17)	106.9(2)
C(5)–C(4)–H(41)	118.8	C(22)–C(23)–C(24)	103.5(2)
C(4)–C(5)–C(6)	120.4(3)	N(17)–C(23)–C(24)	114.5(2)
C(4)–C(5)–H(51)	120.5	C(22)–C(23)–H(231)	109.9
C(6)–C(5)–H(51)	119.1	N(17)–C(23)–H(231)	110.2
C(5)–C(6)–C(7)	118.0(3)	C(24)–C(23)–H(231)	111.4
C(5)–C(6)–H(61)	120.7	C(23)–C(24)–C(25)	102.3(2)
C(7)–C(6)–H(61)	121.2	C(23)–C(24)–H(241)	111.7
C(2)–C(7)–C(6)	121.4(3)	C(25)–C(24)–H(241)	111.4
C(2)–C(7)–C(8)	107.2(2)	C(23)–C(24)–H(242)	110.7
C(6)–C(7)–C(8)	131.3(3)	C(25)–C(24)–H(242)	111.2
C(7)–C(8)–C(9)	115.0(2)	H(241)–C(24)–H(242)	109.4
C(7)–C(8)–C(13)	102.3(2)	C(24)–C(25)–C(26)	108.2(2)
C(9)–C(8)–C(13)	113.4(2)	C(24)–C(25)–C(28)	102.4(2)
C(7)–C(8)–H(81)	108.5	C(26)–C(25)–C(28)	102.4(2)
C(9)–C(8)–H(81)	108.9	C(24)–C(25)–H(251)	113.4
C(13)–C(8)–H(81)	108.3	C(26)–C(25)–H(251)	113.6

C(8)–C(9)–C(10)	123.9(2)	C(28)–C(25)–H(251)	115.7
C(8)–C(9)–Cl(14)	115.23(19)	C(25)–C(26)–C(27)	103.7(2)
C(10)–C(9)–Cl(14)	120.8(2)	C(25)–C(26)–H(261)	111.0
C(9)–C(10)–C(11)	119.8(3)	C(27)–C(26)–H(261)	111.1
C(9)–C(10)–H(101)	119.9	C(25)–C(26)–H(262)	112.6
C(11)–C(10)–H(101)	120.3	C(27)–C(26)–H(262)	110.2
C(10)–C(11)–C(12)	122.6(3)	H(261)–C(26)–H(262)	108.3
C(10)–C(11)–H(111)	118.7	C(22)–C(27)–C(26)	102.2(2)
C(12)–C(11)–H(111)	118.7	C(22)–C(27)–H(272)	110.0
C(11)–C(12)–C(13)	120.7(2)	C(26)–C(27)–H(272)	111.6
C(11)–C(12)–C(15)	121.9(2)	C(22)–C(27)–H(271)	109.3
C(13)–C(12)–C(15)	116.0(2)	C(26)–C(27)–H(271)	112.8
C(8)–C(13)–C(12)	113.4(2)	H272–C27–H271	110.6
C(8)–C(13)–N(1)	102.4(2)	C(25)–C(28)–C(22)	92.2(2)
C(12)–C(13)–N(1)	113.8(2)	C(25)–C(28)–C(29)	113.9(2)
C(8)–C(13)–H(131)	109.8	C(22)–C(28)–C(29)	116.9(2)
C(12)–C(13)–H(131)	108.3	C(25)–C(28)–C(30)	114.7(2)
N(1)–C(13)–H(131)	109.0	C(22)–C(28)–C(30)	112.2(2)
C(12)–C(15)–O(16)	121.6(3)	C(29)–C(28)–C(30)	106.9(2)
C(12)–C(15)–N(17)	118.8(2)	C(28)–C(29)–H(293)	109.6
O(16)–C(15)–N(17)	119.4(2)	C(28)–C(29)–H(292)	107.8
C(15)–N(17)–S(18)	119.06(17)	H(293)–C(29)–H(292)	109.6
C(15)–N(17)–C(23)	116.6(2)	C(28)–C(29)–H(291)	109.8
S(18)–N(17)–C(23)	111.03(17)	H(293)–C(29)–H(291)	110.1
N(17)–S(18)–O(19)	108.81(12)	H(292)–C(29)–H(291)	109.8
N(17)–S(18)–O(20)	109.82(11)	C(28)–C(30)–H(303)	108.5
O(19)–S(18)–O(20)	117.49(12)	C(28)–C(30)–H(302)	109.4
N(17)–S(18)–C(21)	95.95(11)	H(303)–C(30)–H(302)	109.0
O(19)–S(18)–C(21)	111.83(12)	C(28)–C(30)–H(301)	110.3

O(20)–S(18)–C(21)	110.78(13)	H(303)–C(30)–H(301)	110.4
S(18)–C(21)–C(22)	106.47(18)	H(302)–C(30)–H(301)	109.2
S(18)–C(21)–H(211)	110.6		

7. References

1 Nenajdenko, V. G., Moiseev, A. M. & Balenkova, E. S. A novel method for the oxidation of thiophenes. Synthesis of thiophene 1,1-dioxides containing electron-withdrawing substituents. *Russ. Chem. Bull.* **53**, 2241-2247 (2004).

2 Azzouzi, A., Perrin, B., Sinibaldi, M.-E., Gramain, J.-C. & Lavaud, C. Stereoselective preparation of tri and tetracyclic amines as potential intermediates in Aspidosperma alkaloid synthesis. *Tetrahedron Lett.* **34**, 5451-5454 (1993).

3 Beemelmanns, C. & Reissig, H.-U. A Short Formal Total Synthesis of Strychnine with a Samarium Diiodide Induced Cascade Reaction as the Key Step. *Angew. Chem. Int. Ed.* **49**, 8021-8025 (2010).

4 Hong, A. Y. & Vanderwal, C. D. A Synthesis of Alsmaphorazine B Demonstrates the Chemical Feasibility of a New Biogenetic Hypothesis. *J. Am. Chem. Soc.* **137**, 7306-7309 (2015).

5 Rawal, V. H. & Michoud, C. A general solution to the synthesis of 2-azabicyclo[3.3.1]nonane unit of Strychnos alkaloids. *Tetrahedron Lett.* **32**, 1695-1698 (1991).

6 Huh, C. W., Bechle, B. M. & Warmus, J. S. Development of a scalable synthetic route towards a 2,2,6-trisubstituted chiral morpholine via stereoselective hydroalkoxylation. *Tetrahedron Lett.* **59**, 1808-1812 (2018).

7 Park, J. & Chen, D. Y. K. A Desymmetrization-Based Total Synthesis of Reserpine. *Angew. Chem. Int. Ed.* **57**, 16152-16156 (2018).

8 Jones, S. B., Simmons, B., Mastracchio, A. & MacMillan, D. W. C. Collective synthesis of natural products by means of organocascade catalysis. *Nature* **475**, 183-188 (2011).

9 Martin, D. B. C. & Vanderwal, C. D. Efficient Access to the Core of the Strychnos, Aspidosperma and Iboga Alkaloids. A Short Synthesis of Norfluorocurarine. *J. Am. Chem. Soc.* **131**, 3472-3473 (2009).

10 Hong, A. Y. & Vanderwal, C. D. A sequential cycloaddition strategy for the synthesis of Alsmaphorazine B traces a path through a family of Alstonia alkaloids. *Tetrahedron* **73**, 4160-4171 (2017).

11 Bergman, J. A., Hahne, K., Hrycyna, C. A. & Gibbs, R. A. Lipid and sulfur substituted prenylcysteine analogs as human Icmt inhibitors. *Bioorg. Med. Chem. Lett.* **21**, 5616-5619 (2011).

12 Liu, C. *et al.* Gold(i)-catalyzed pathway-switchable tandem cycloisomerizations to indolizino[8,7-b]indole and indolo[2,3-a]quinolizine derivatives. *Chem. Commun.* **55**, 14418-14421 (2019).

13 Neese, F. The ORCA program system. *Wiley Interdiscip. Rev. Comput. Mol. Sci.* **2**, 73-78. Full details of computational methods are described in the Supporting Information (2012).

14 Neese, F. Software update: the ORCA program system, version 4.0. *Wiley Interdiscip. Rev. Comput. Mol. Sci.* **8**, e1327 (2018).

15 Neese, F., Wennmohs, F., Hansen, A. & Becker, U. Efficient, approximate and parallel Hartree–Fock and hybrid DFT calculations. A 'chain-of-spheres' algorithm for the Hartree–Fock exchange. *Chem. Phys.* **356**, 98-109 (2009).

16 Stoychev, G. L., Auer, A. A. & Neese, F. Automatic Generation of Auxiliary Basis Sets. *J. Chem. Theory Comput.* **13**, 554-562 (2017).

17 Zhao, Y. & Truhlar, D. G. The M06 suite of density functionals for main group thermochemistry, thermochemical kinetics, noncovalent interactions, excited states, and

transition elements: two new functionals and systematic testing of four M06-class functionals and 12 other functionals. *Theor. Chem. Acc.* **120**, 215-241 (2008).

18 Grimme, S. Semiempirical hybrid density functional with perturbative second-order correlation. *J. Chem. Phys.* **124**, 034108 (2006).

19 Young, T. A., Martí-Centelles, V., Wang, J., Lusby, P. J. & Duarte, F. Rationalizing the Activity of an "Artificial Diels-Alderase": Establishing Efficient and Accurate Protocols for Calculating Supramolecular Catalysis. *J. Am. Chem. Soc.* **142**, 1300-1310 (2020).

20 Sterling, A. J., Dürr, A. B., Smith, R. C., Anderson, E. A. & Duarte, F. Rationalizing the diverse reactivity of [1.1.1]propellane through σ–π-delocalization. *Chem. Sci.* **11**, 4895-4903 (2020).

21 Penocchio, E., Piccardo, M. & Barone, V. Semiexperimental Equilibrium Structures for Building Blocks of Organic and Biological Molecules: The B2PLYP Route. *J. Chem. Theory Comput.* **11**, 4689-4707 (2015).

22 Linder, M. & Brinck, T. On the method-dependence of transition state asynchronicity in Diels–Alder reactions. *Phys. Chem. Chem. Phys.* **15**, 5108-5114 (2013).

23 Jr., T. H. D. Gaussian basis sets for use in correlated molecular calculations. I. The atoms boron through neon and hydrogen. *J. Chem. Phys.* **90**, 1007-1023 (1989).

24 Pracht, P., Bohle, F. & Grimme, S. Automated exploration of the low-energy chemical space with fast quantum chemical methods. *Phys. Chem. Chem. Phys.* **22**, 7169-7192 (2020).

25 Klopman, G. Solvations: a semi-empirical procedure for including solvation in quantum mechanical calculations of large molecules. *Chem. Phys. Lett.* **1**, 200-202 (1967).

26 Bannwarth, C., Ehlert, S. & Grimme, S. GFN2-xTB—An Accurate and Broadly Parametrized Self-Consistent Tight-Binding Quantum Chemical Method with Multipole Electrostatics and Density-Dependent Dispersion Contributions. *J. Chem. Theory Comput.* **15**, 1652-1671 (2019).

27 Barone, V. & Cossi, M. Quantum Calculation of Molecular Energies and Energy Gradients in Solution by a Conductor Solvent Model. *J. Phys. Chem. A* **102**, 1995-2001 (1998).

28 Grimme, S., Ehrlich, S. & Goerigk, L. Effect of the damping function in dispersion corrected density functional theory. *J. Comput. Chem.* **32**, 1456-1465 (2011).

29 Liakos, D. G., Sparta, M., Kesharwani, M. K., Martin, J. M. & Neese, F. Exploring the Accuracy Limits of Local Pair Natural Orbital Coupled-Cluster Theory. *J. Chem. Theory Comput.* **11**, 1525-1539 (2015).

30 Grimme, S. Supramolecular binding thermodynamics by dispersion-corrected density functional theory. *Chem. Eur. J.* **18**, 9955-9964 (2012).

31 Young, T. duartegroup/otherm. (2020).

32 Batatia, I., Kovács, D. P., Simm, G. N. C., Ortner, C. & Csányi, G. MACE: Higher Order Equivariant Message Passing Neural Networks for Fast and Accurate Force Fields. *Adv. Neural Inf. Process. Syst.* **35**, 11423 (2022).

33 Batatia, I., Kovács, D. P., Simm, G. N. C., Ortner, C. & Csányi, G. MACE: Higher Order Equivariant Message Passing Neural Networks for Fast and Accurate Force Fields. *arXiv*, 2205.06643 (2022).

34 Young, T. A. *et al.* mlp-train package. https://figshare.com/articles/software/mlp-train_package/25816864 (2024).

35 Barducci, A., Bussi, G. & Parrinello, M. Well-Tempered Metadynamics: A Smoothly Converging and Tunable Free-Energy Method. *Phys. Rev. Lett.* **100**, 020603 (2008).

36 Zhang, H., Juraskova, V. & Duarte, F. Modeling Chemical Processes in Explicit Solvents with Machine Learning Potentials. *Nat. Commun.* **15**, 6114 (2024).

37 Bonomi, M. *et al.* PLUMED: A portable plugin for free-energy calculations with molecular dynamics. *Comp. Phys. Commun.* **180**, 1961-1972 (2009).
38 Tribello, G. A., Bonomi, M., Branduardi, D., Camilloni, C. & Bussi, G. PLUMED 2: New feathers for an old bird. *Comp. Phys. Commun.***185**, 604-613 (2014).
39 Hjorth Larsen, A. *et al.* The atomic simulation environment—a Python library for working with atoms. *J. Phys. Condens. Matter* **29**, 273002 (2017).
40 Park, K. H. K., Frank, N., Duarte, F. & Anderson, E. A. Collective Synthesis of Illudalane Sesquiterpenes via Cascade Inverse Electron Demand (4 + 2) Cycloadditions of Thiophene S,S-Dioxides. *J. Am. Chem. Soc.* **144**, 10017-10024 (2022).
41 Palatinus, L. & Chapuis, G. SUPERFLIP - a computer program for the solution of crystal structures by charge flipping in arbitrary dimensions. *J. Appl. Cryst.* **40**, 786-790 (2007).
42 Parois, P., Cooper, R. I. & Thompson, A. L. Crystal structures of increasingly large molecules: meeting the challenges with CRYSTALS software. *Chem Cent J* **9**, 30 (2015).
43 Cooper, R. I., Thompson, A. L. & Watkin, D. J. CRYSTALS enhancements: dealing with hydrogen atoms in refinement. *J. Appl. Cryst.* **43**, 1100-1107 (2010).

8. Copies of NMR Spectra

(*Z*)-*N*-(2-(1*H*-indol-3-yl)ethyl)-2-iodobut-2-en-1-amine, **11** (NMR spectra in $CDCl_3$)

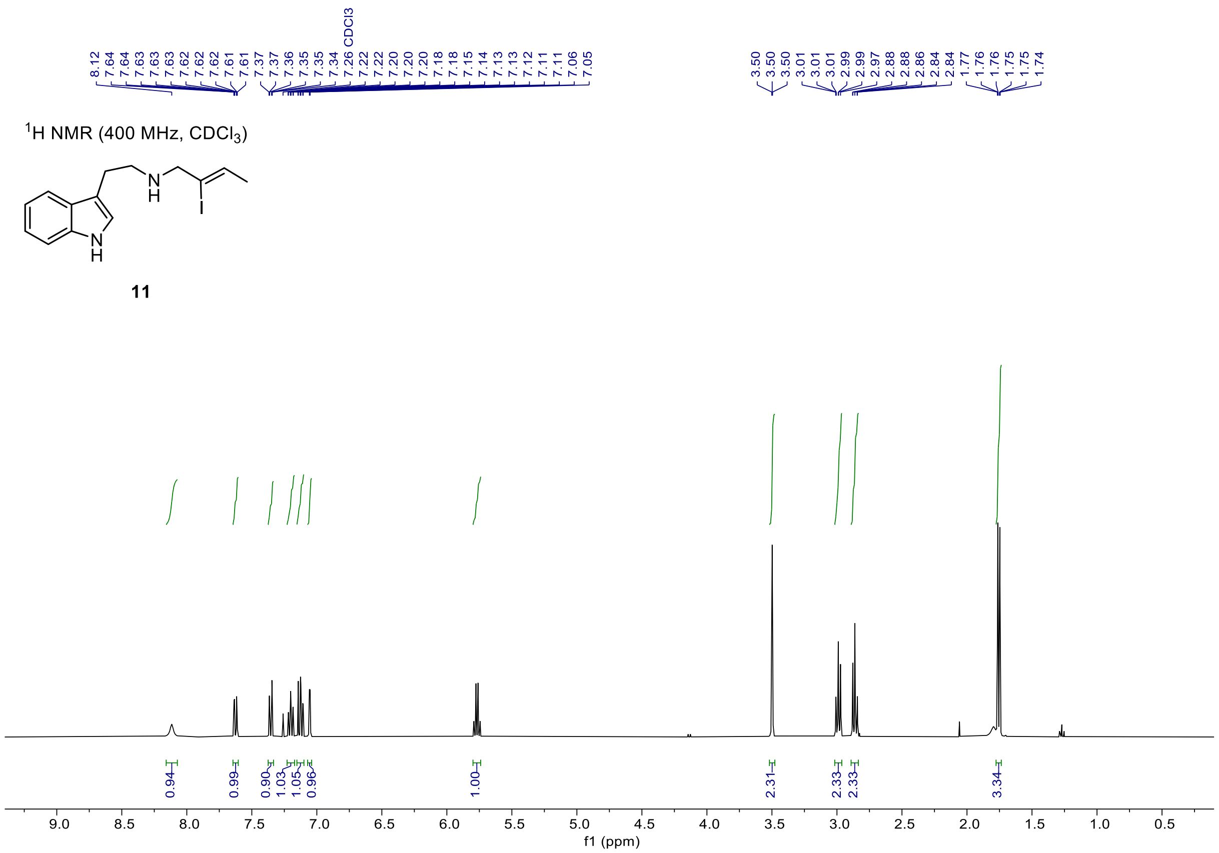

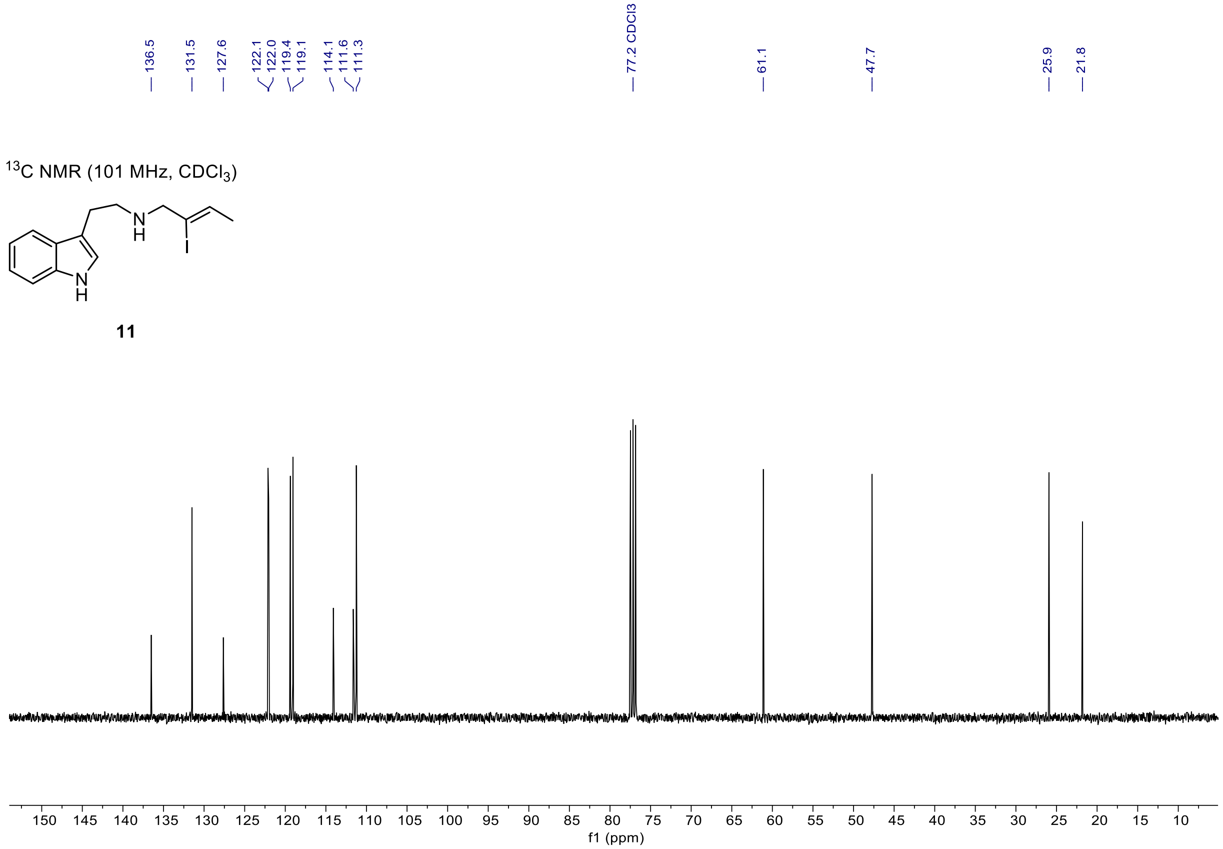

Methyl 5-chlorothiophene-2-carboxylate 1,1-dioxide, **9e** (NMR spectra in $CDCl_3$)

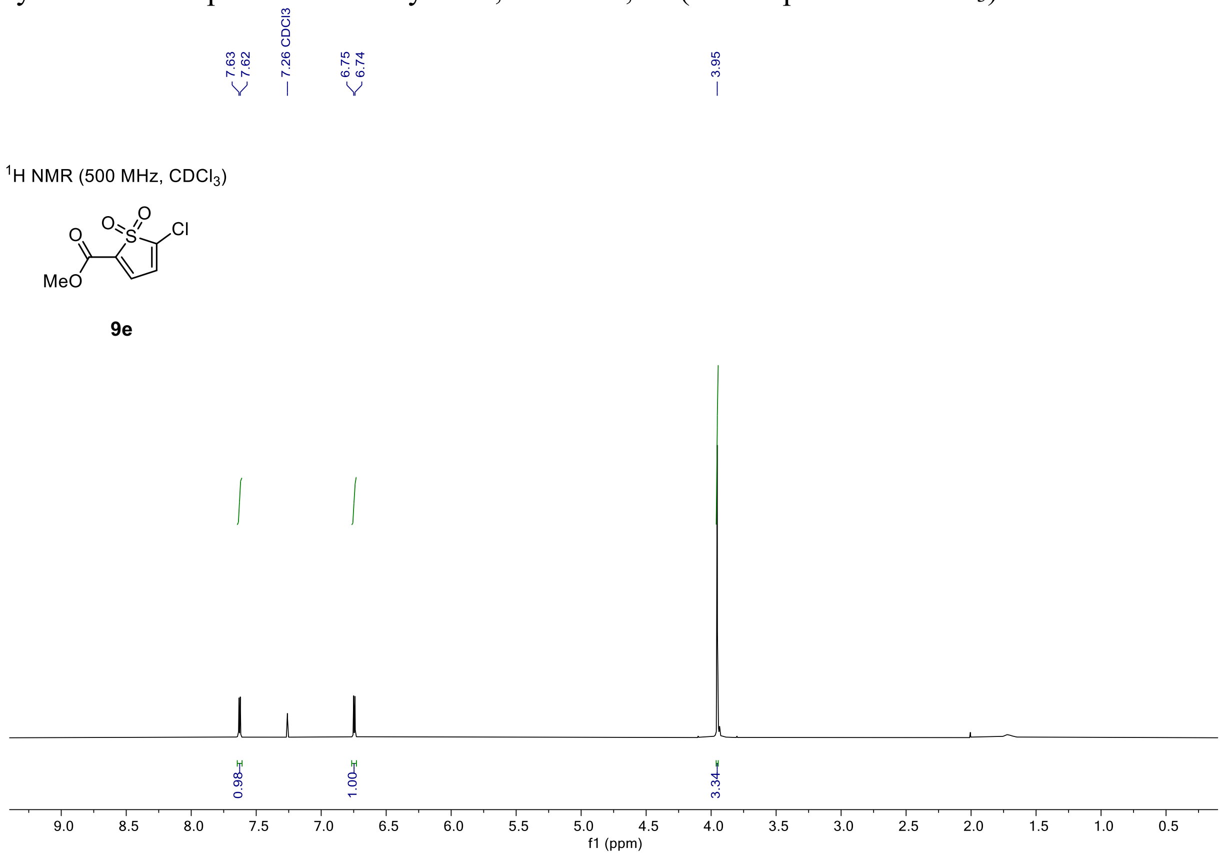

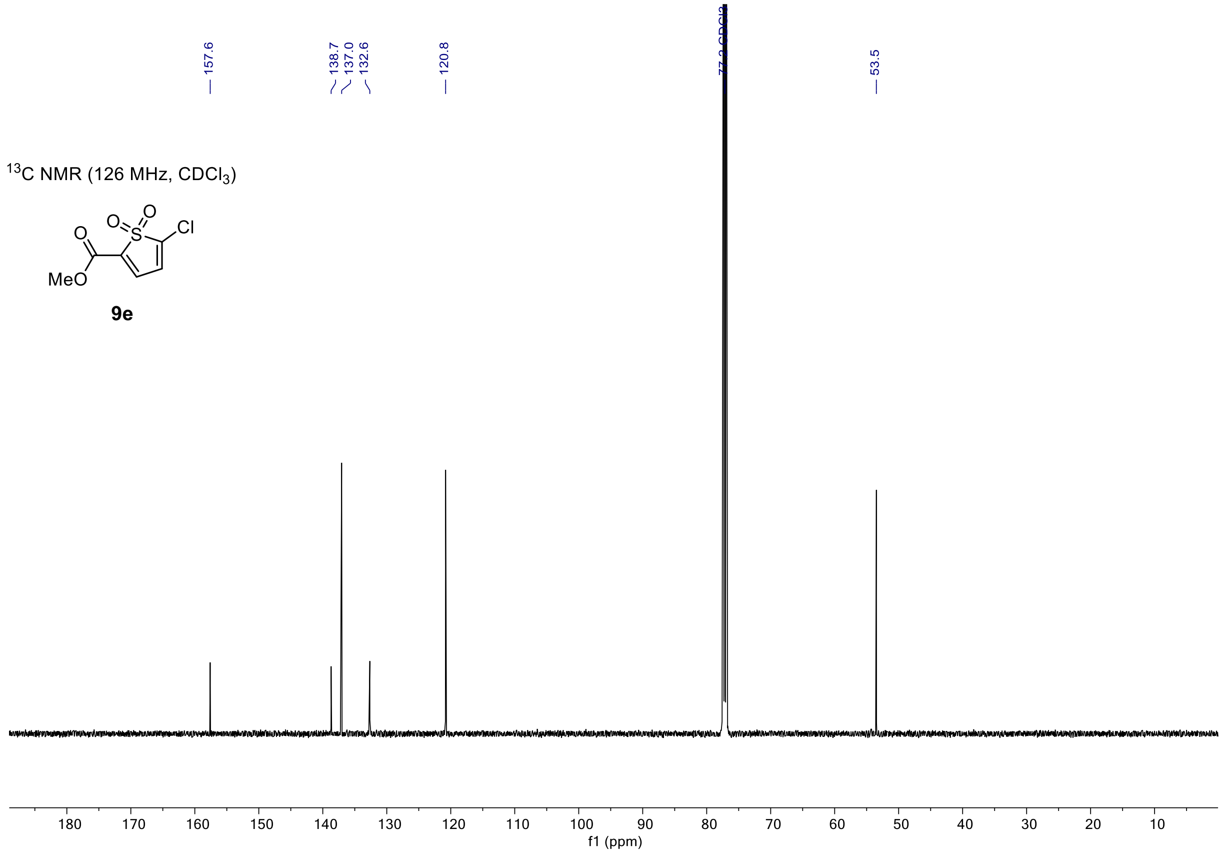

Methyl (3a*S*,6a*R*,11b*S*)-3-((*Z*)-2-iodobut-2-en-1-yl)-2,3,3a,4,6a,7-hexahydro-1*H*-pyrrolo[2,3-*d*]carbazole-6-carboxylate, **S2** (NMR spectra in $CDCl_3$)

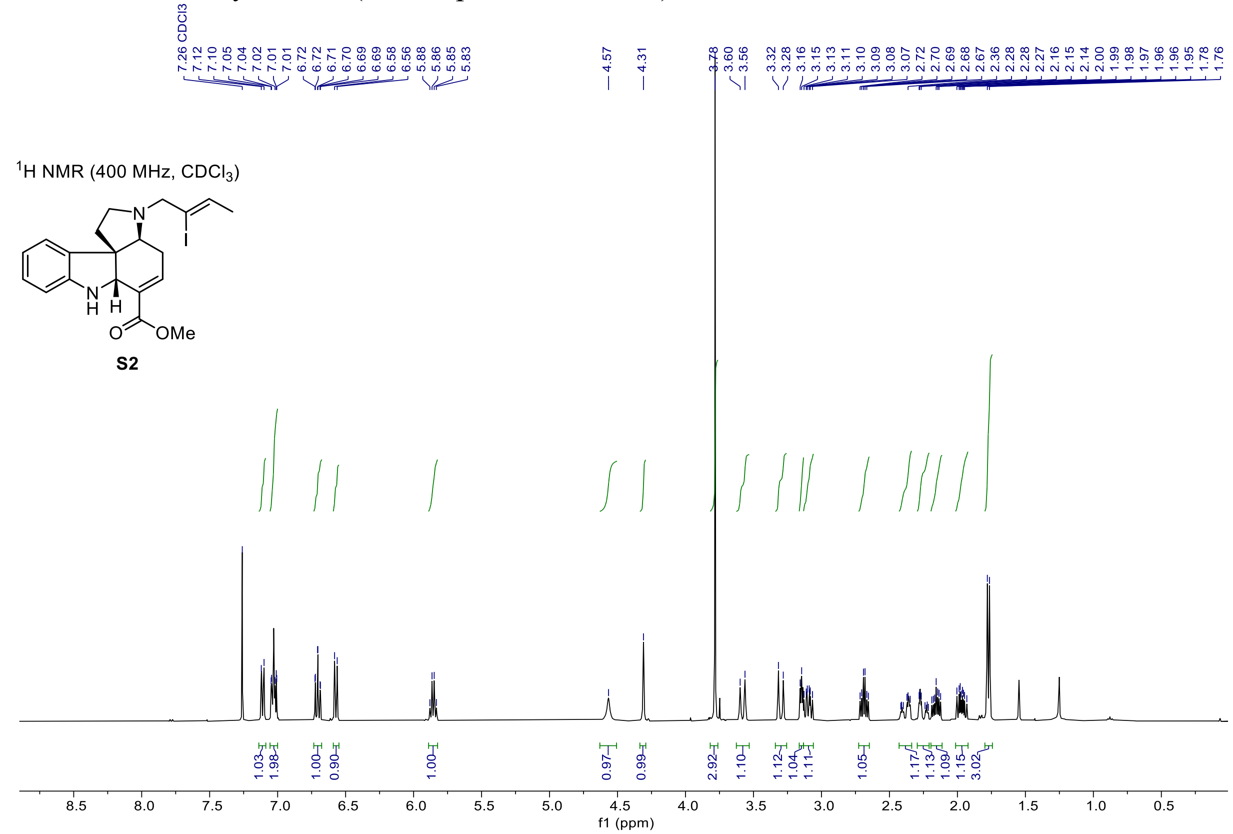

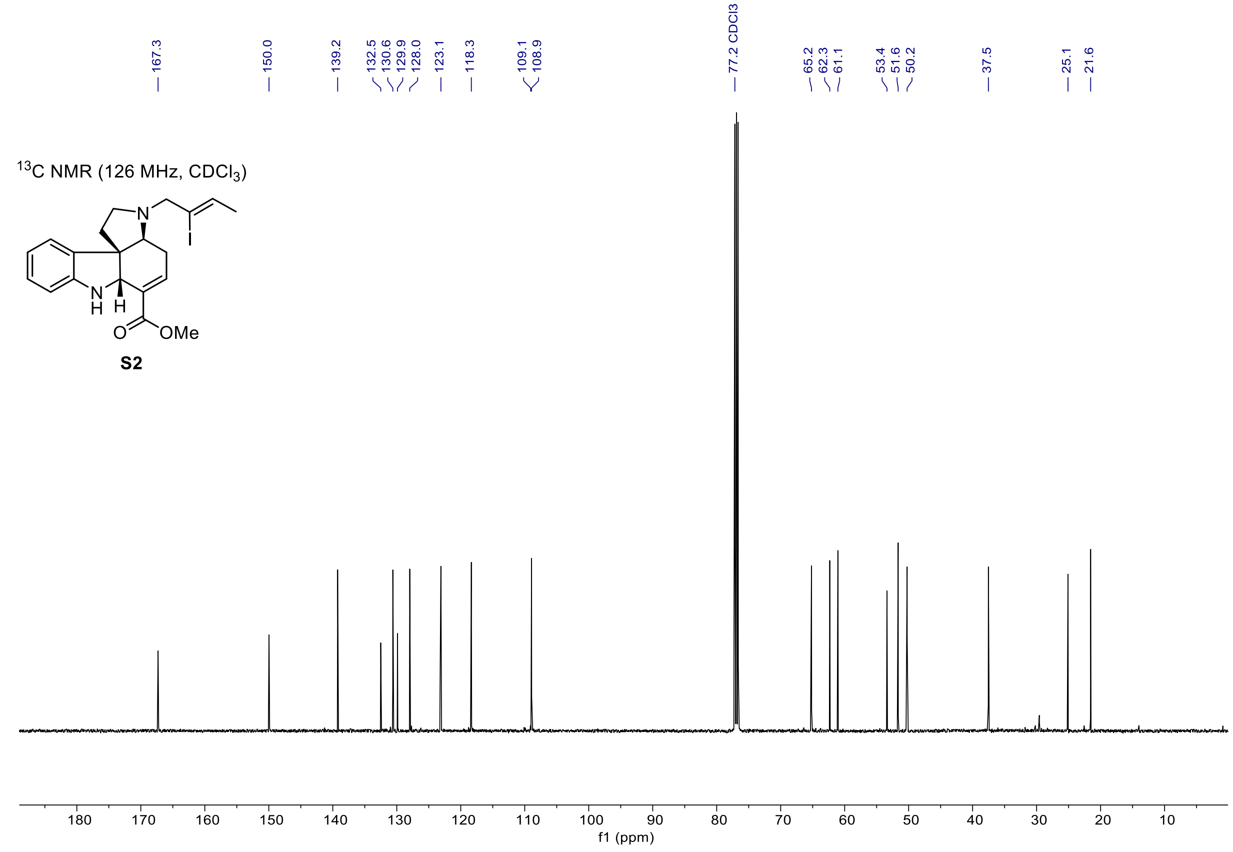

Methyl (3a*R*,6a*R*,11b*S*)-3-((*Z*)-2-iodobut-2-en-1-yl)-2,3,3a,4,6a,7-hexahydro-1*H*-pyrrolo[2,3-*d*]carbazole-6-carboxylate, **S2'** (Spectra in $CDCl_3$)

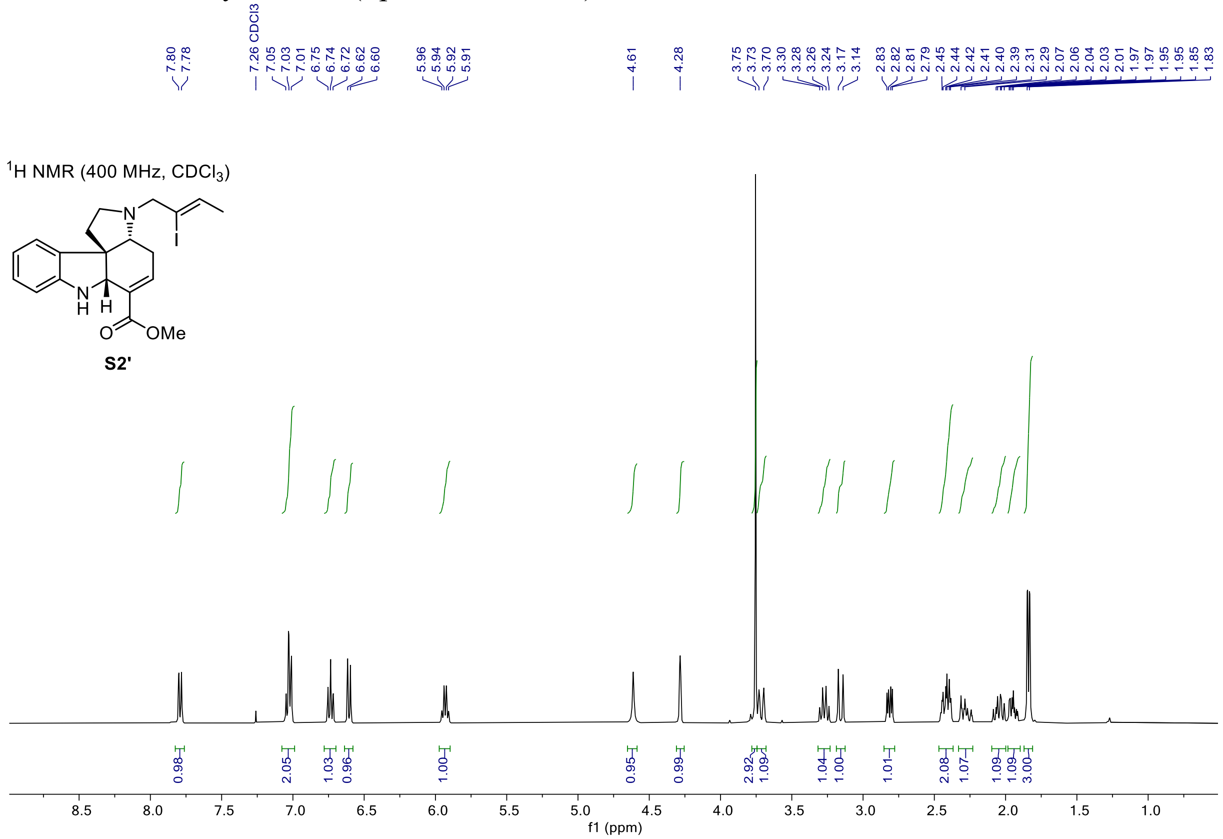

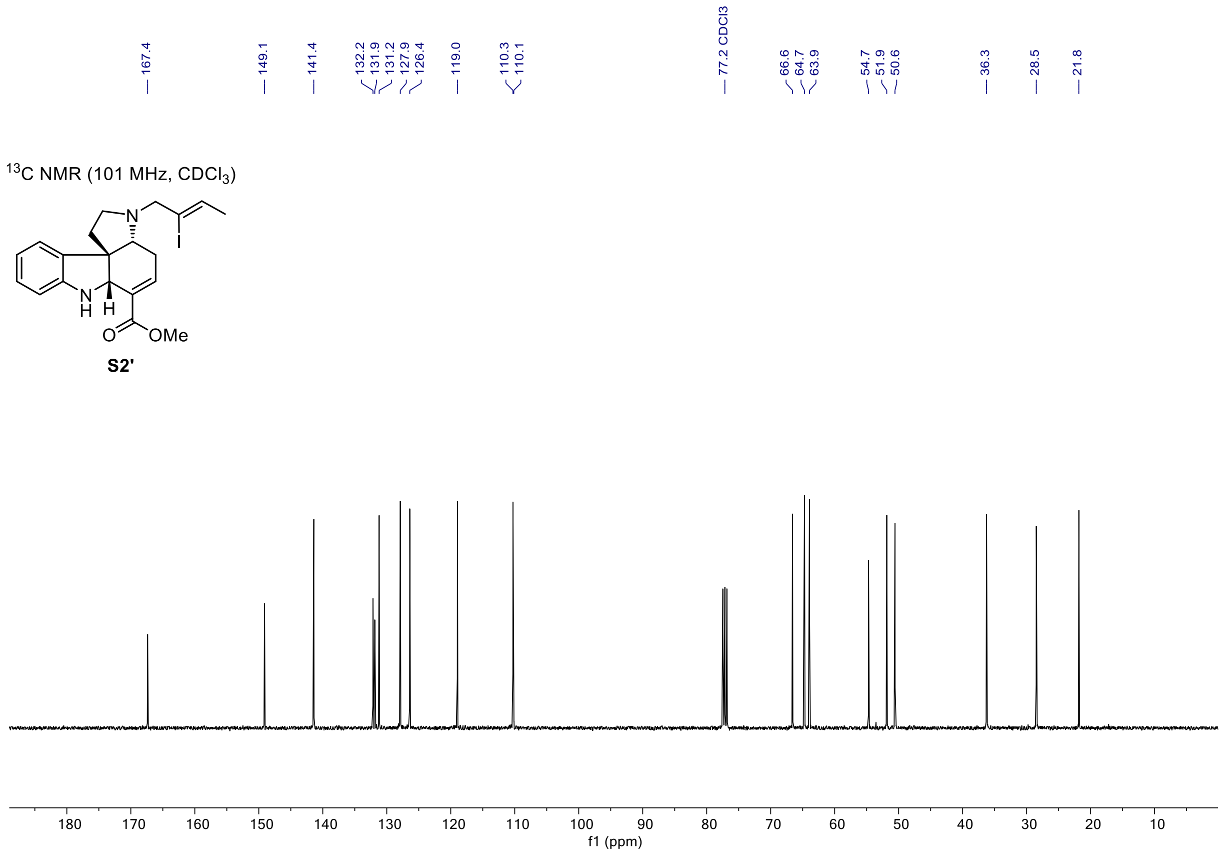

(±)-Methyl (3aS,5S,11*b*R,E)-1*2*-ethylidene-1,2,3a,4,5,7-hexahydro-3,5-ethanopyrrolo[2,3-d]ca*r*bazole-6-carboxylate, **rac-akuammicine** (Spectra in $CDCl_3$)

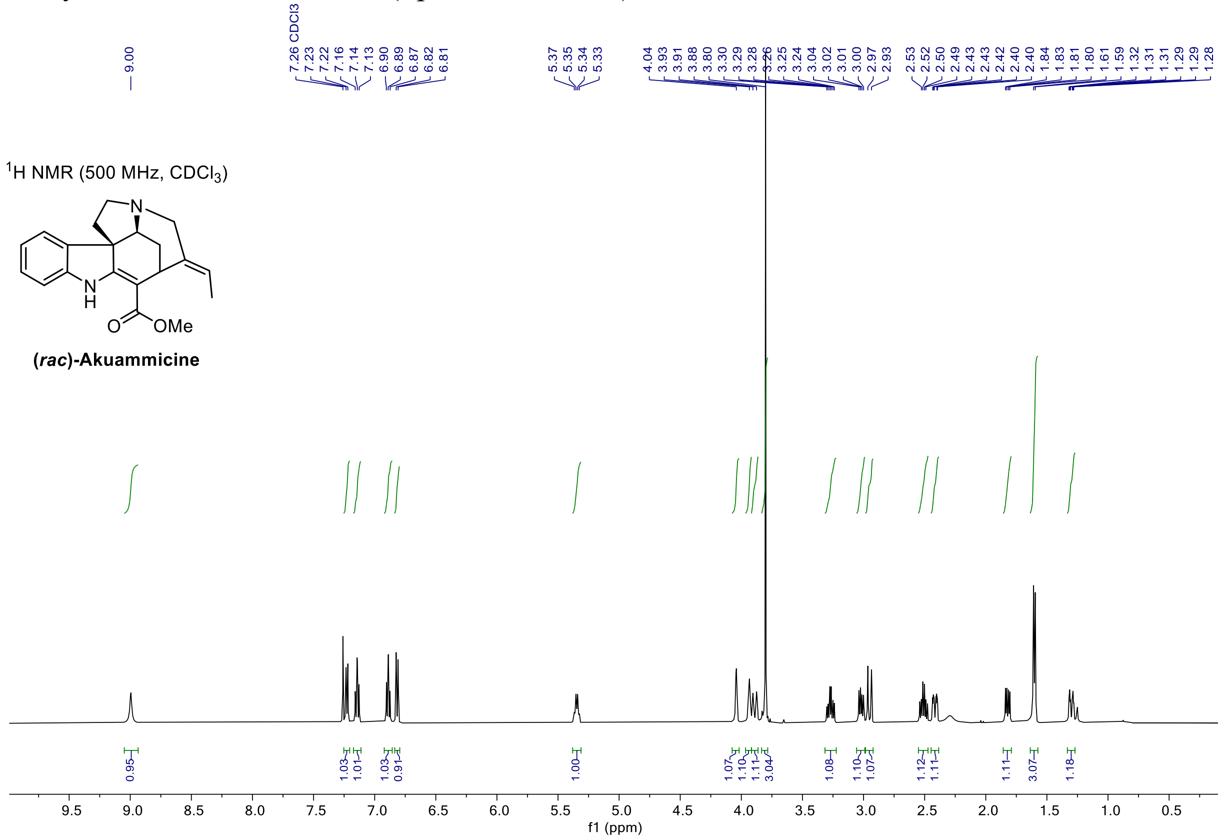

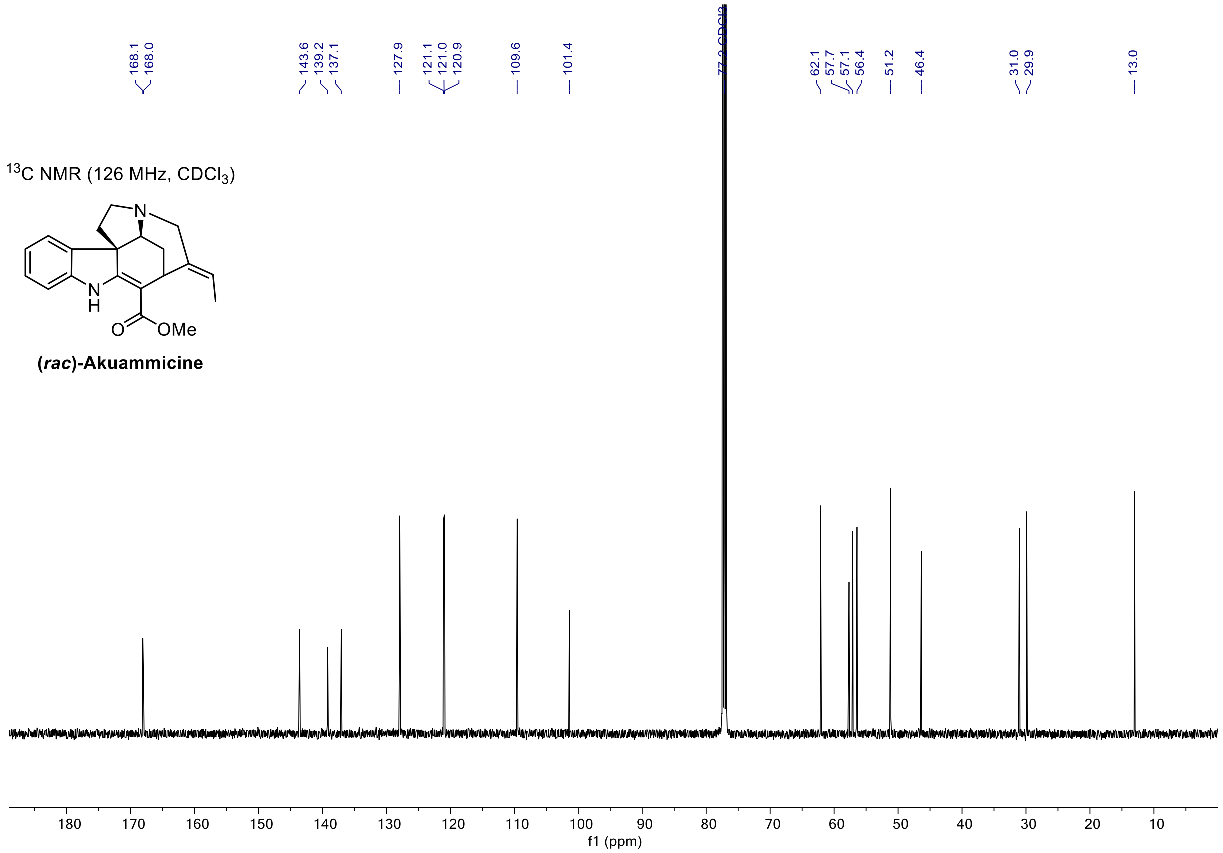

(5-Chlorothiophen-2-yl)((3a*S*,6*R*,7a*R*)-8,8-dimethyl-2,2-dioxidotetrahydro-3*H*-3a,6-methanobenzo[*c*]isothiazol-1(4*H*)-yl)methanone, **S3** (Spectra in $CDCl_3$)

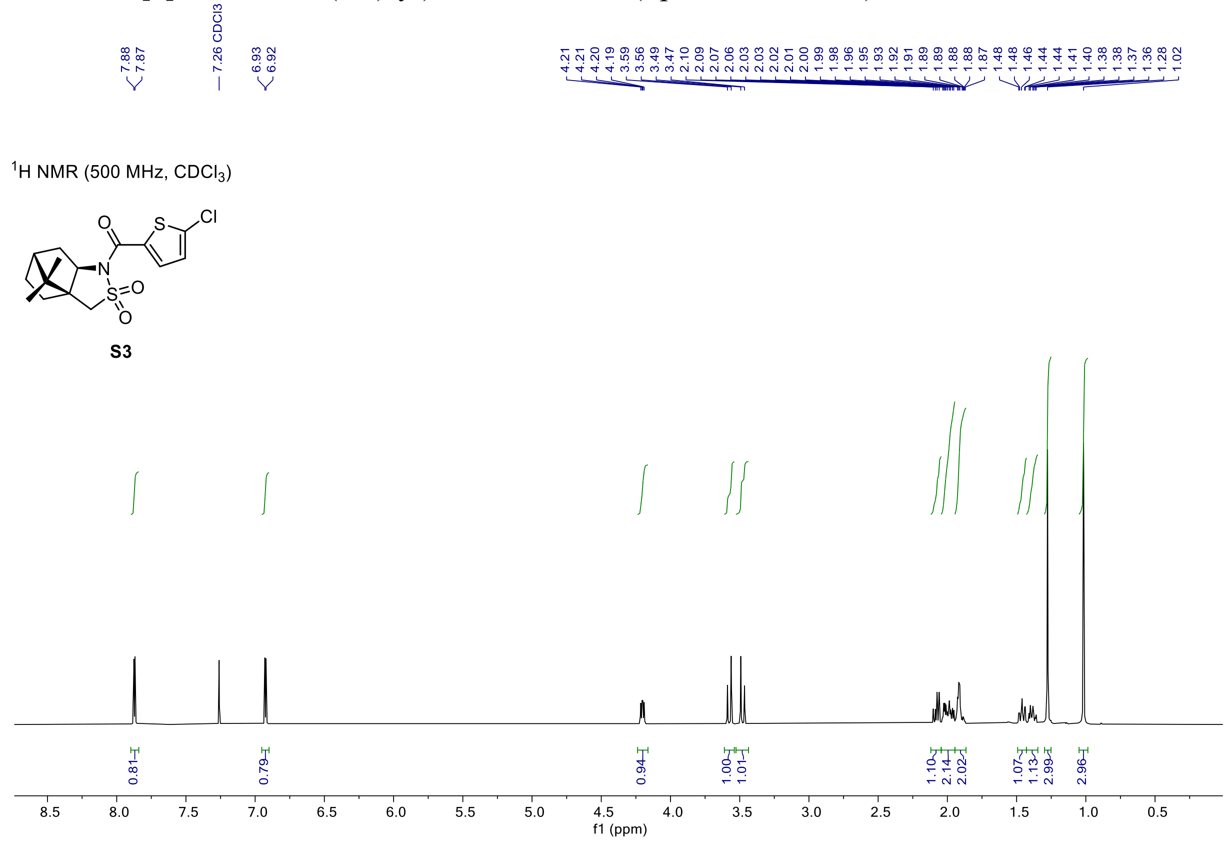

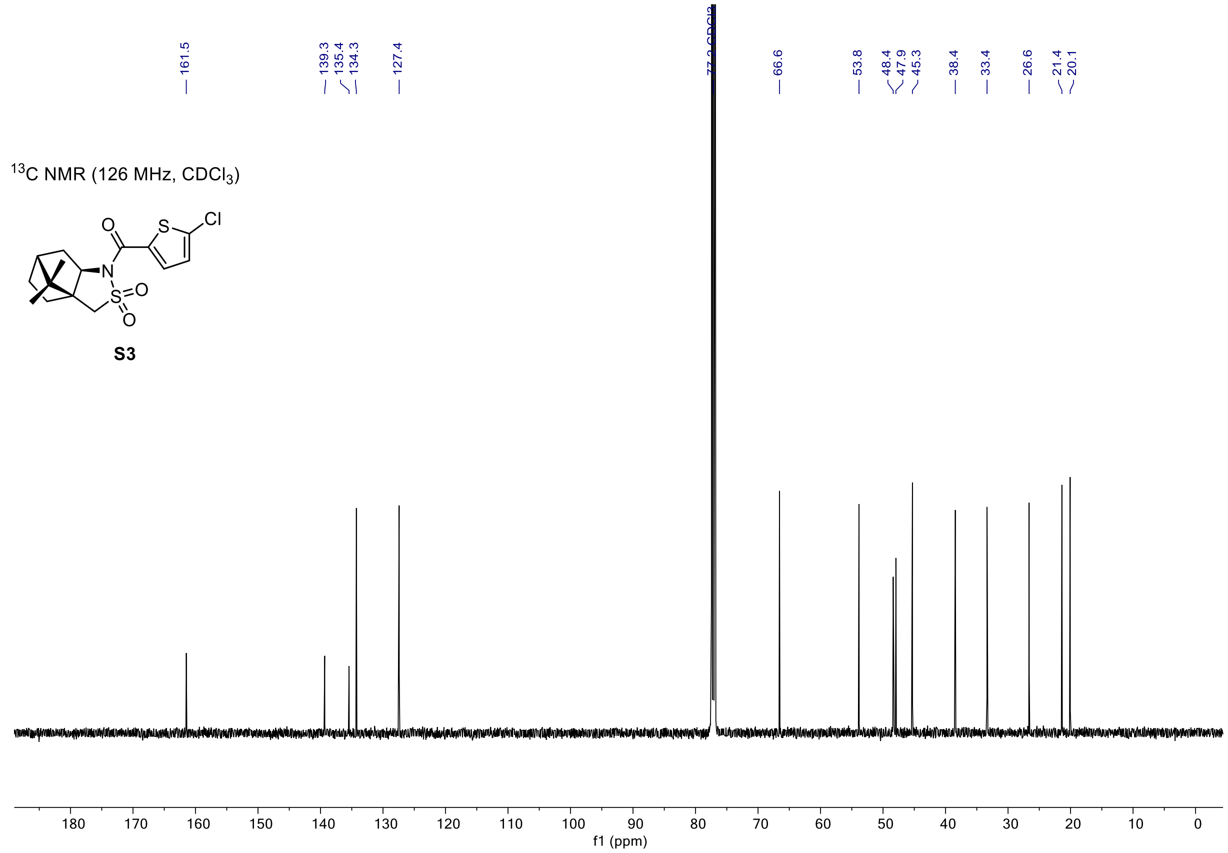

(5-Chloro-1,1-dioxidothiophen-2-yl)((3a*S*,6*R*,7a*R*)-8,8-dimethyl-2,2-dioxidotetrahydro-3*H*-3a,6-methanobenzo[*c*]isothiazol-1(4*H*)-yl)methanone, **9a** (Spectra in $CDCl_3$)

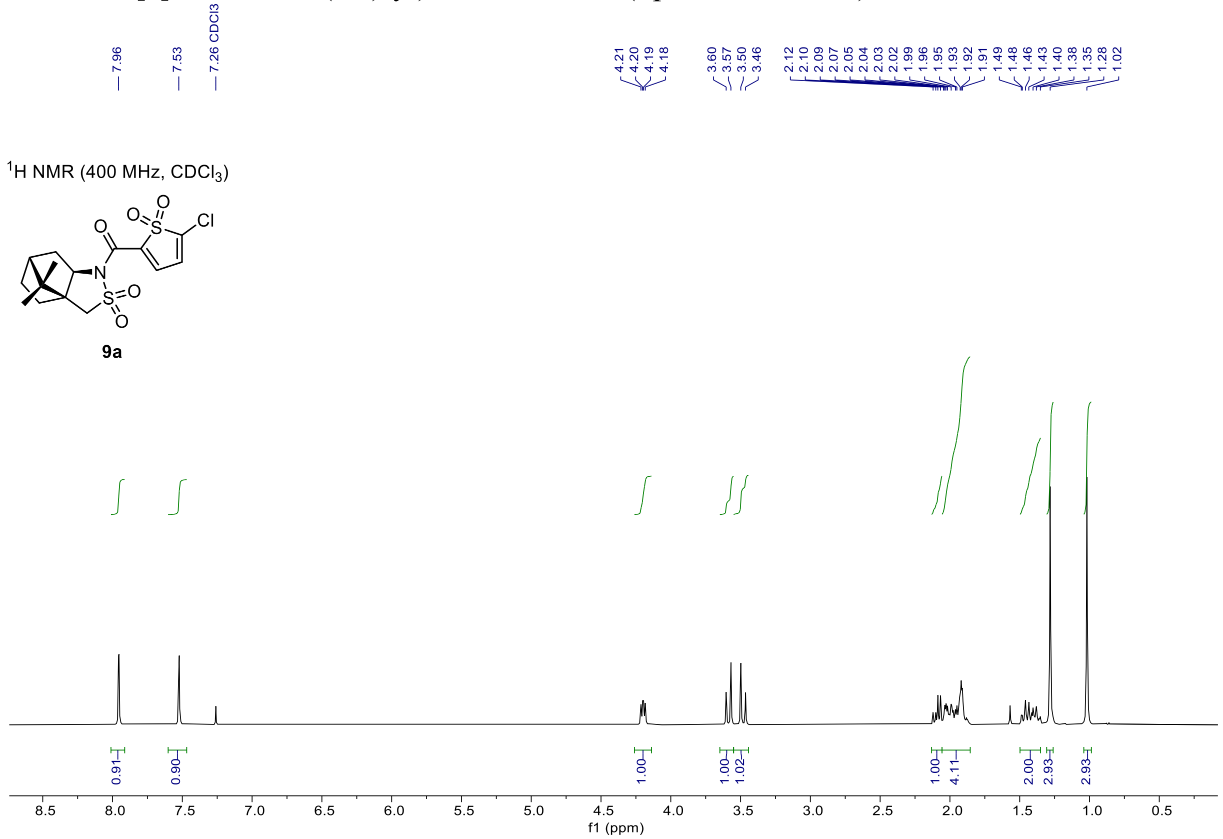

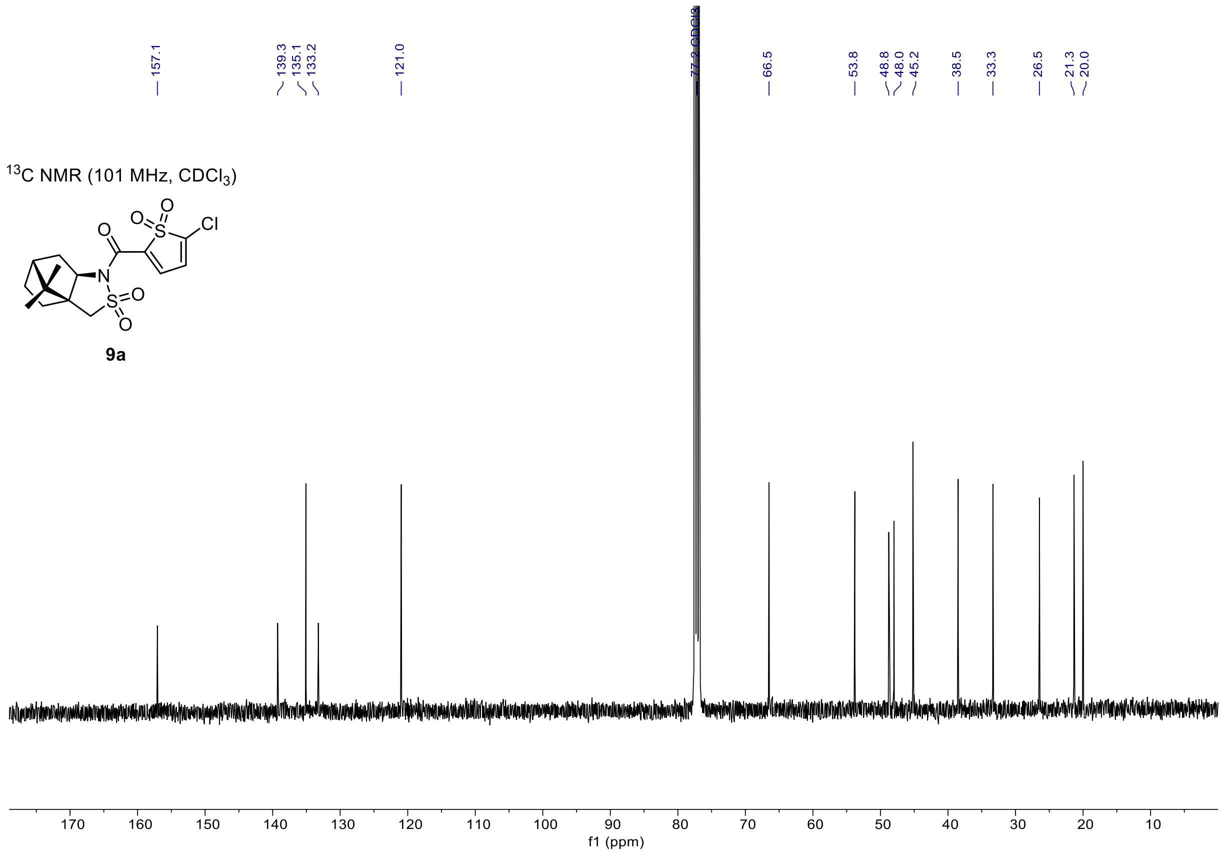

((3a*S*,6*R*,7a*R*)-8,8-Dimethyl-2,2-dioxidotetrahydro-3*H*-3a,6-methanobenzo[*c*]isothiazol-1(4*H*)-yl)((3a*S*,6a*R*,11b*S*)-3-((*Z*)-2-iodobut-2-en-1-yl)-2,3,3a,4,6a,7-hexahydro-1*H*-pyrrolo[2,3-*d*]carbazol-6-yl)methanone, **14** (Spectra in $CDCl_3$)

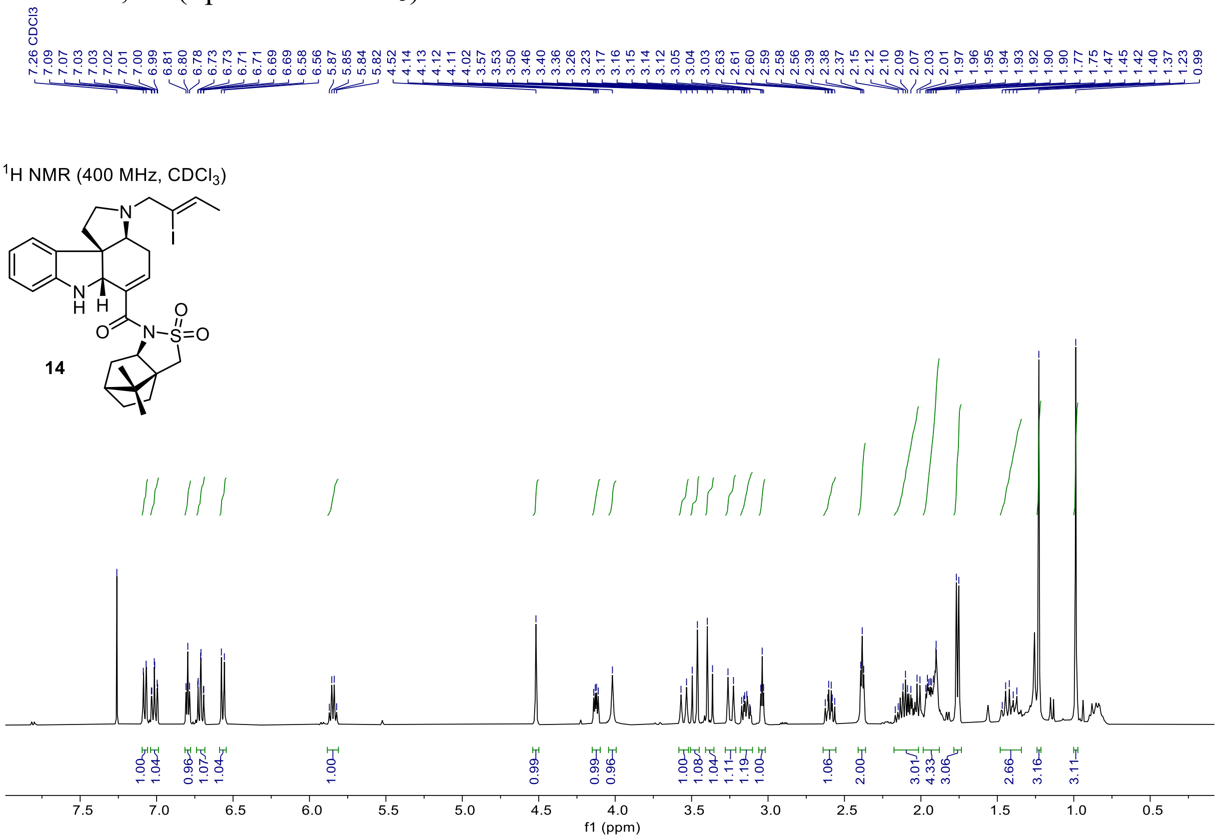

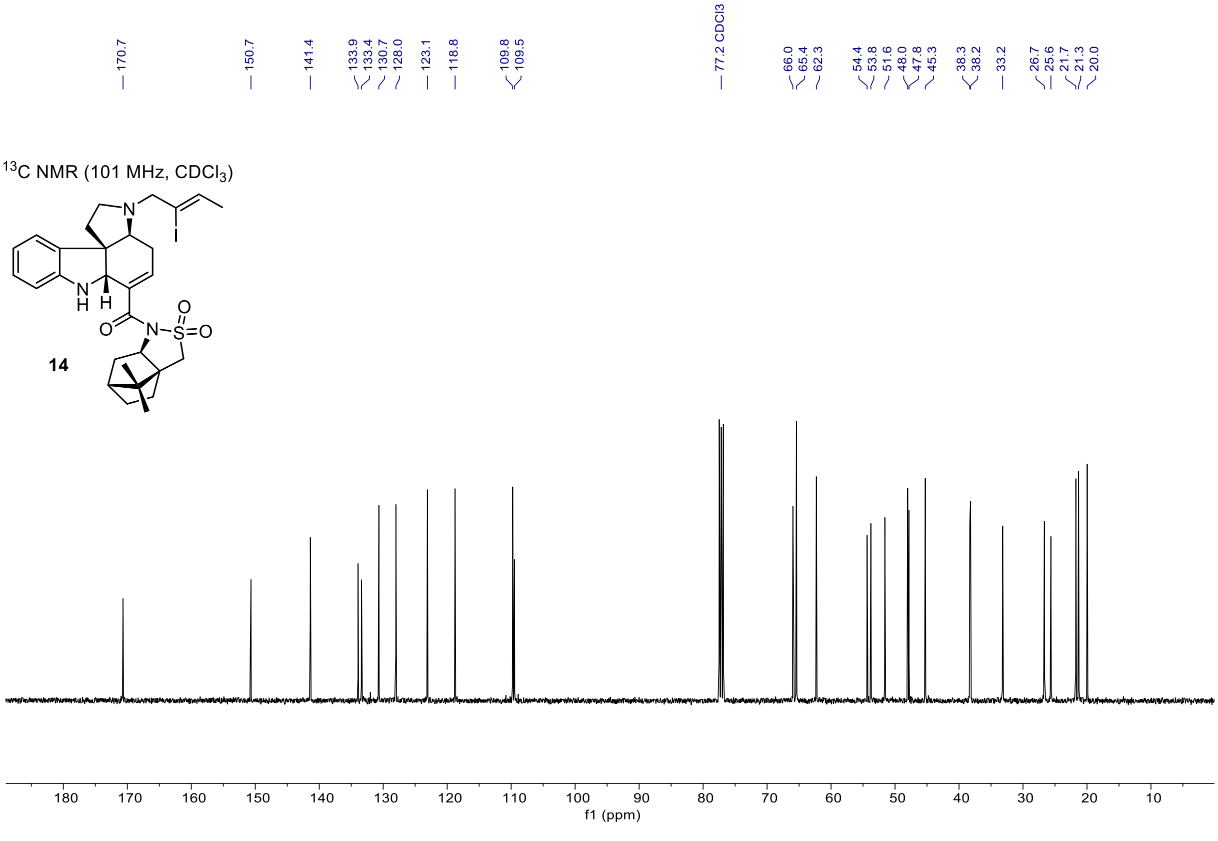

((3a*S*,6*R*,7a*R*)-8,8-Dimethyl-2,2-dioxidotetrahydro-3*H*-3a,6-methanobenzo[*c*]isothiazol-1(4*H*)-yl)((3a*R*,6a*R*,11b*S*)-3-((*Z*)-2-iodobut-2-en-1-yl)-2,3,3a,4,6a,7-hexahydro-1*H*-pyrrolo[2,3-*d*]carbazol-6-yl)methanone, **14'** (Spectra in $CDCl_3$)

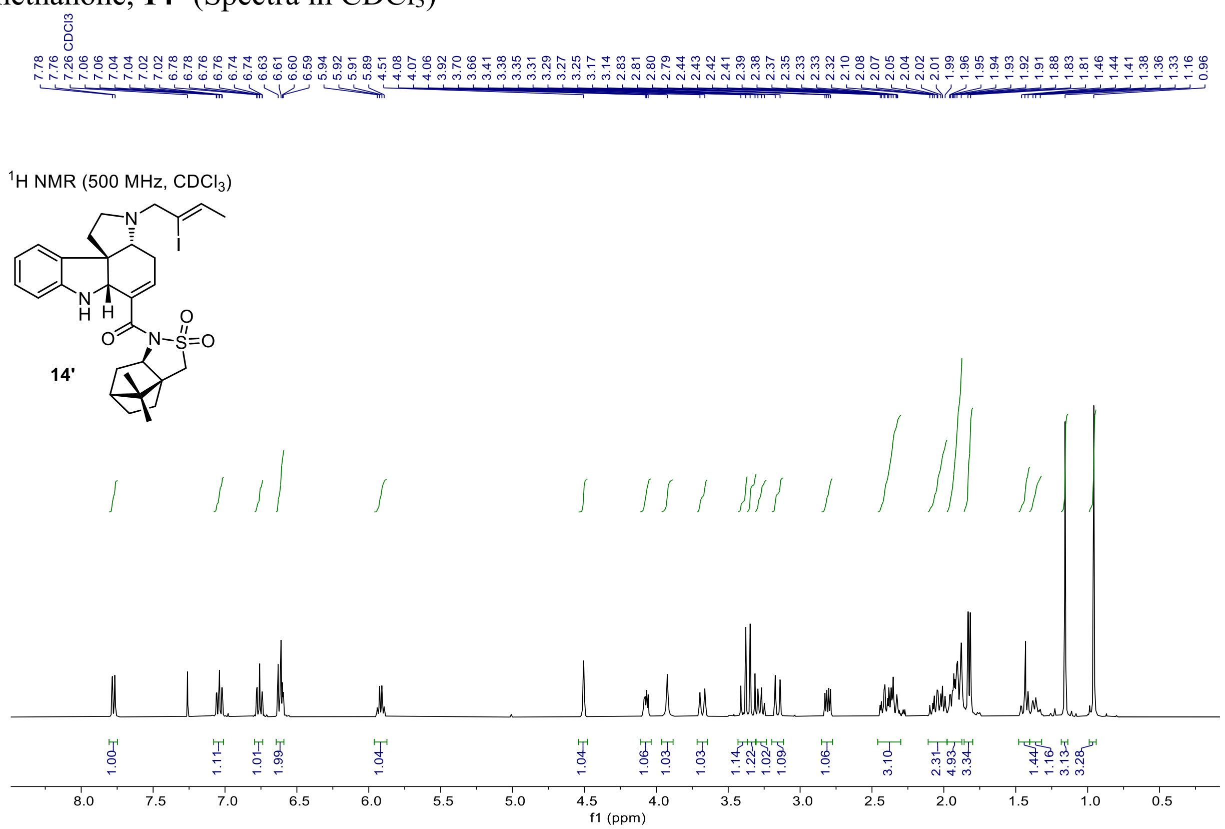

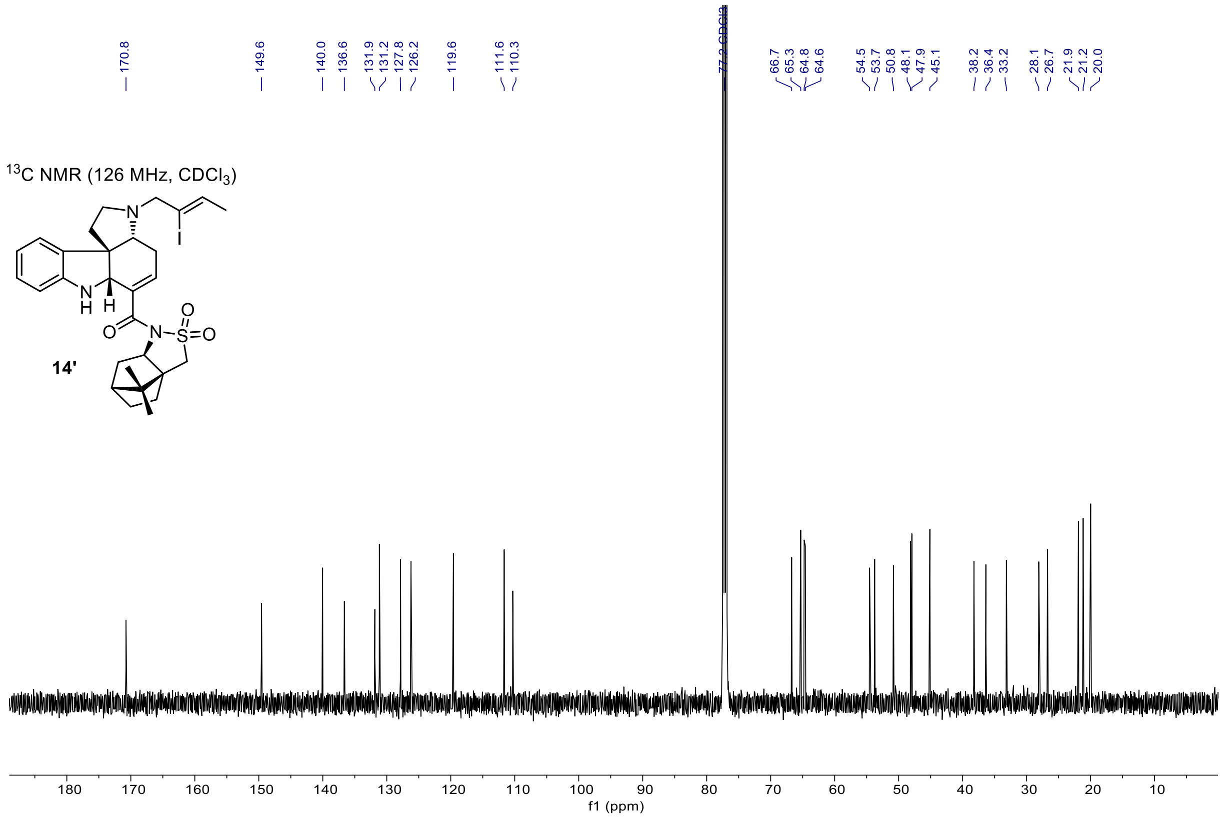

(4-Bromothiophen-2-yl)((3a*S*,6*R*,7a*R*)-8,8-dimethyl-2,2-dioxidotetrahydro-3*H*-3a,6-methanobenzo[*c*]isothiazol-1(4*H*)-yl)methanone, **17** (Spectra in $CDCl_3$)

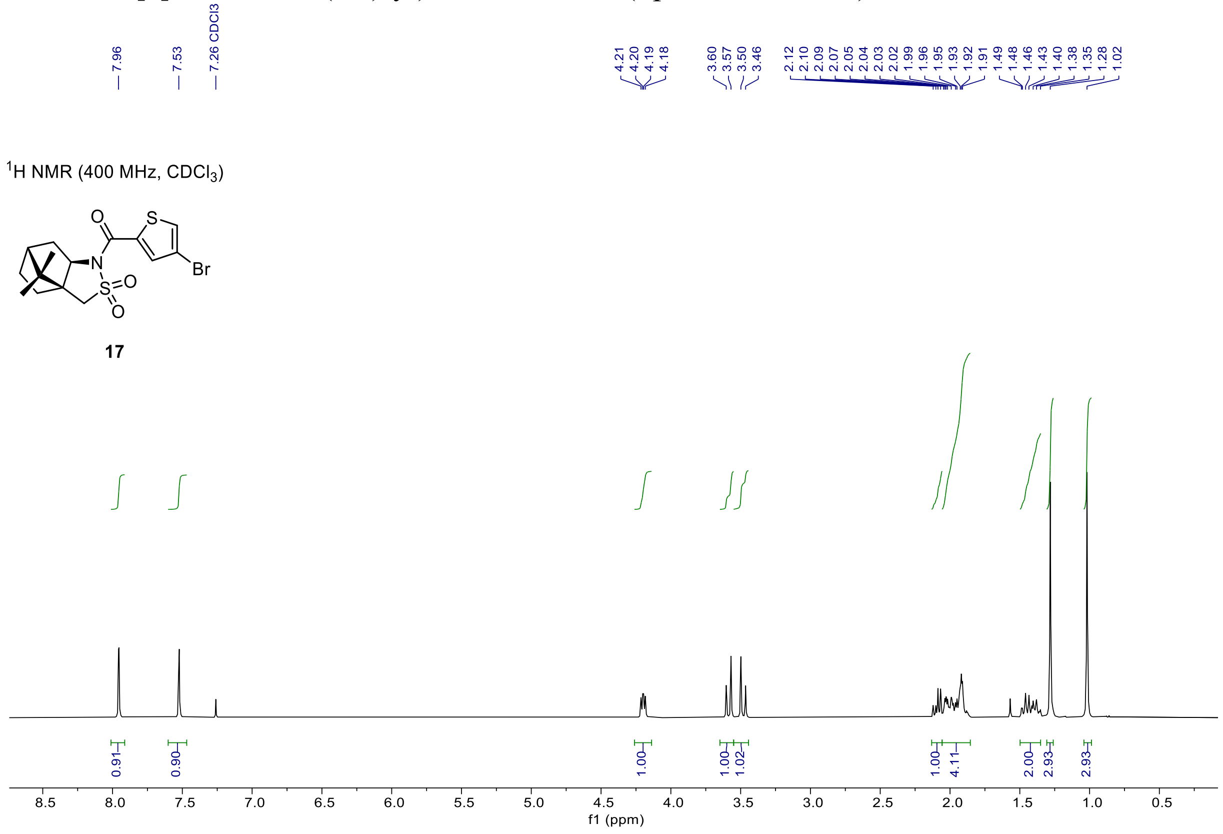

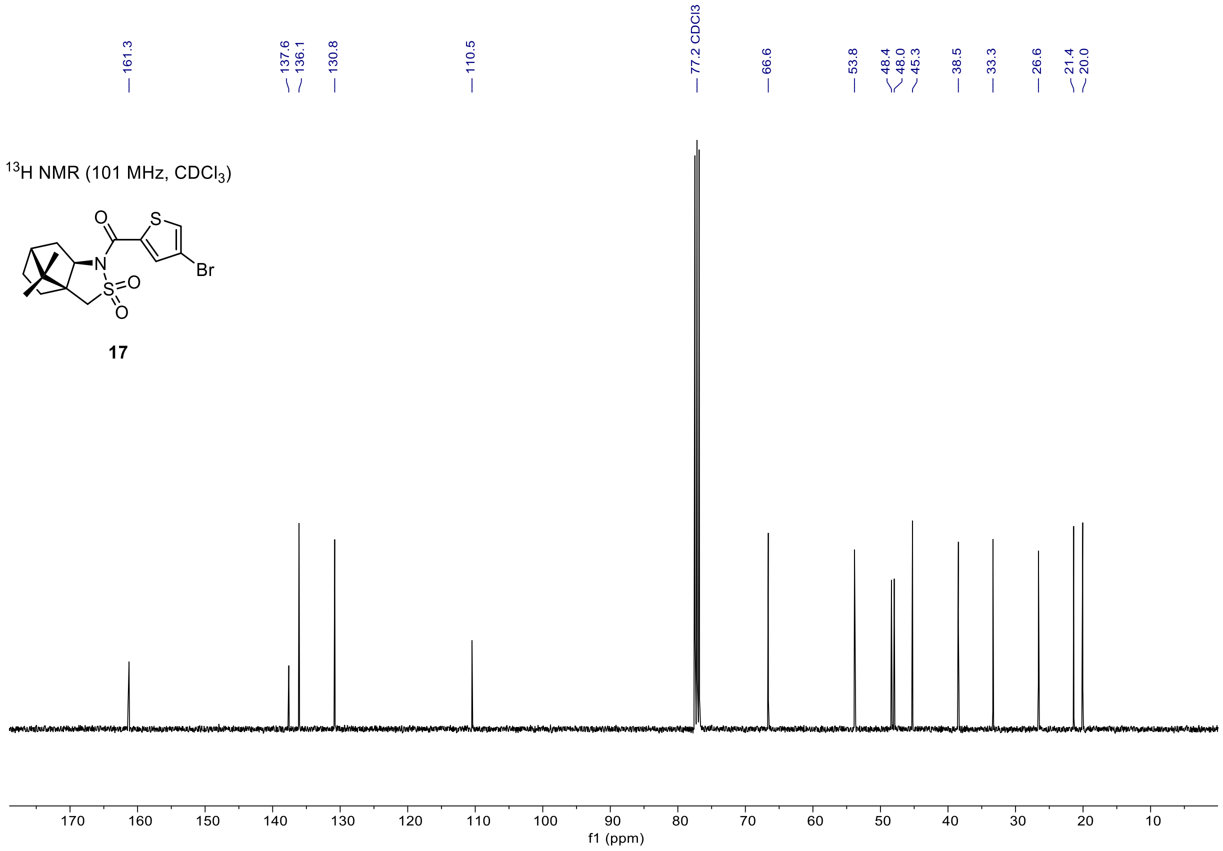

((3a*R*,6a*R*,11b*S*)-4-Bromo-2,3,3a,4,6a,7-hexahydro-1*H*-pyrrolo[2,3-*d*]carbazol-6-yl)((3a*S*,6*R*,7a*R*)-8,8-dimethyl-2,2-dioxidotetrahydro-3*H*-3a,6-methanobenzo[*c*]isothiazol-1(4*H*)-yl)methanone, **19** (Spectra in $CDCl_3$)

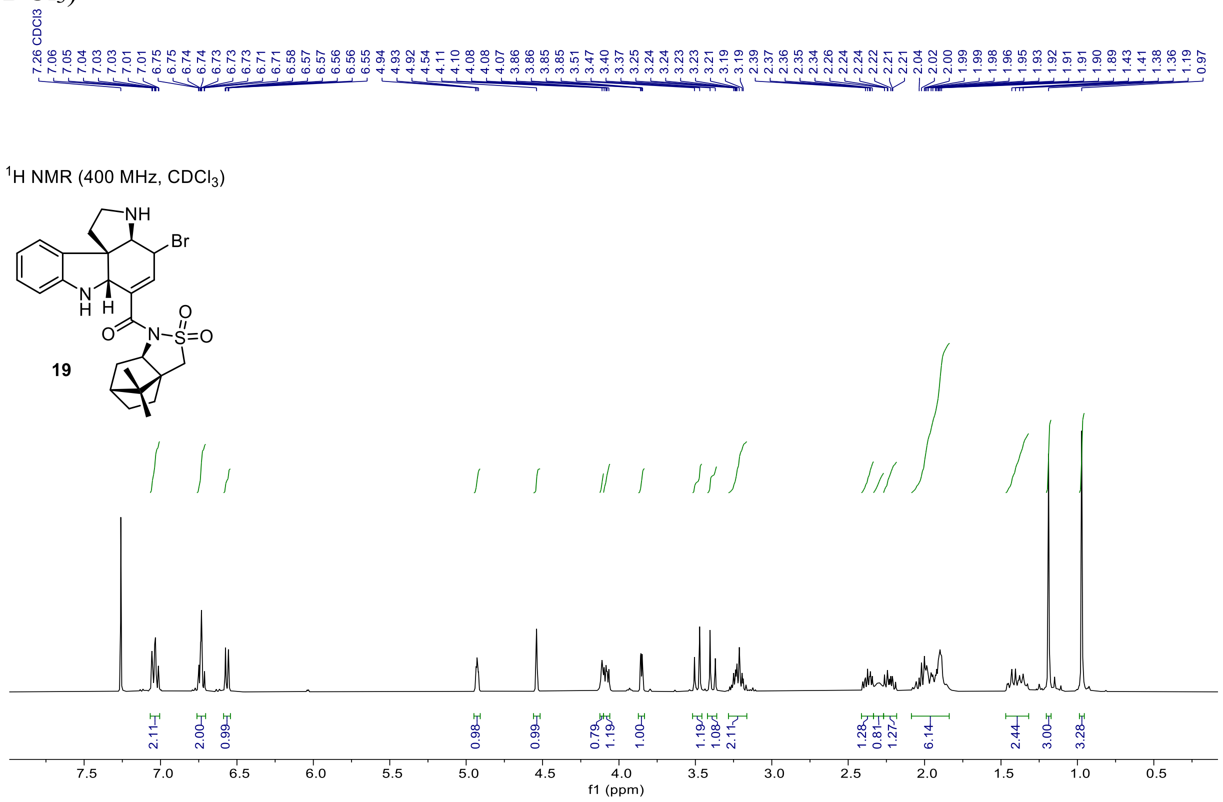

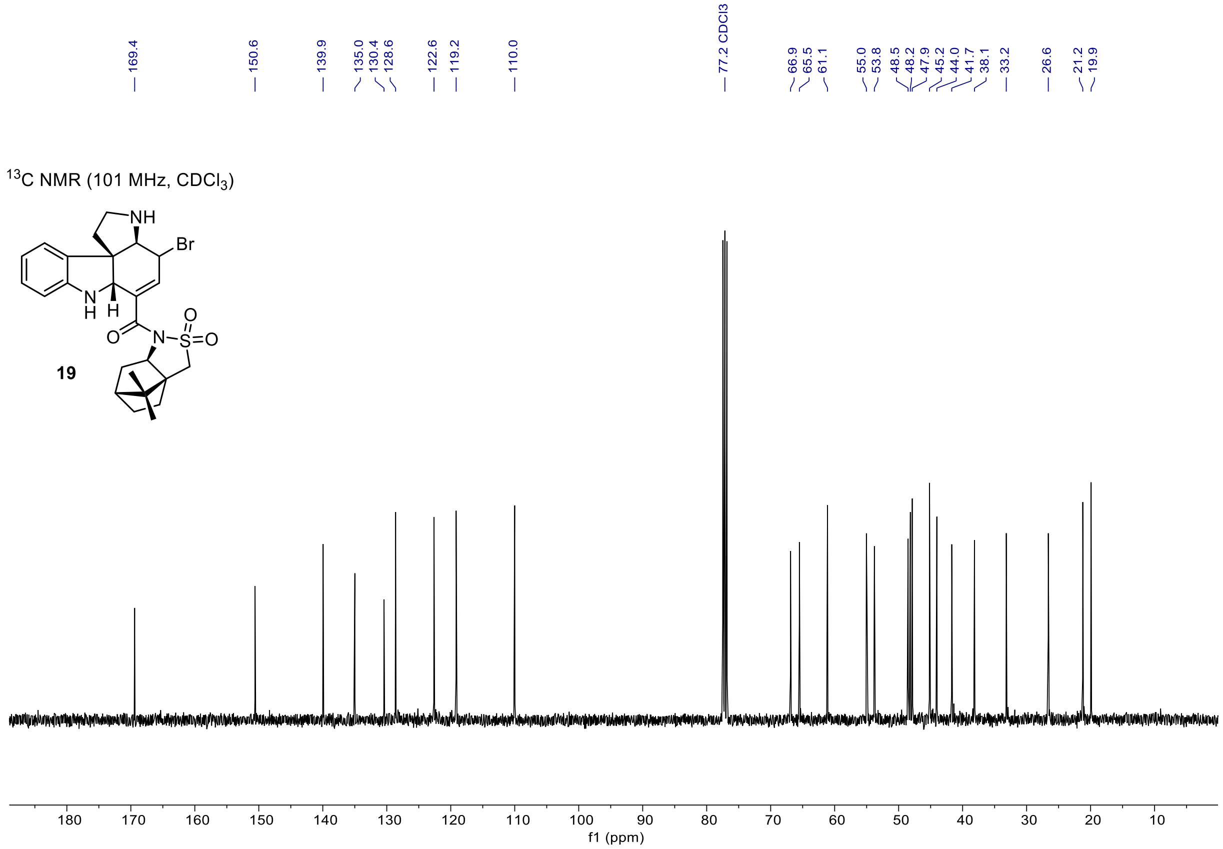

(3a*S*,6a*R*,11b*S*)-3-((*Z*)-2-Iodobut-2-en-1-yl)-2,3,3a,4,6a,7-hexahydro-1*H*-pyrrolo[2,3-*d*]carbazole-6-carbaldehyde, **21** (Spectra in $CDCl_3$)

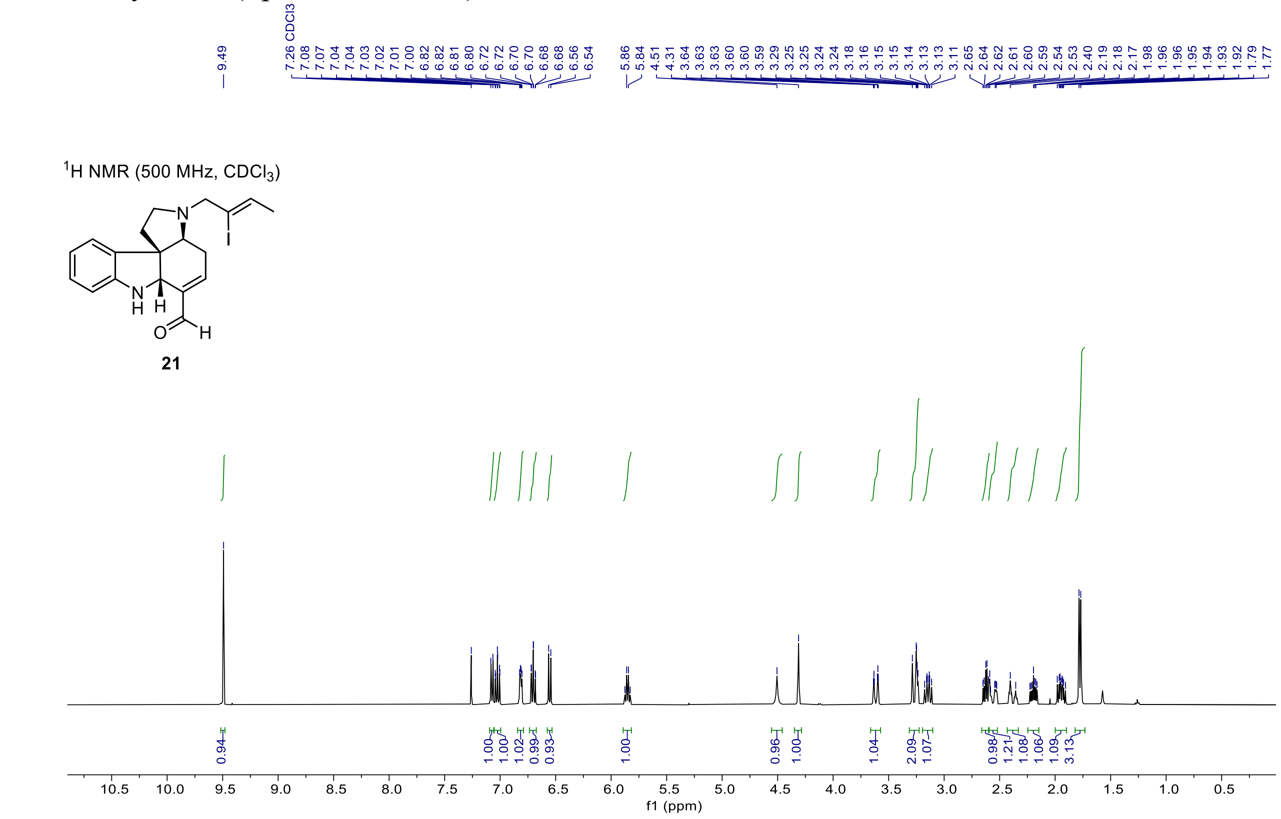

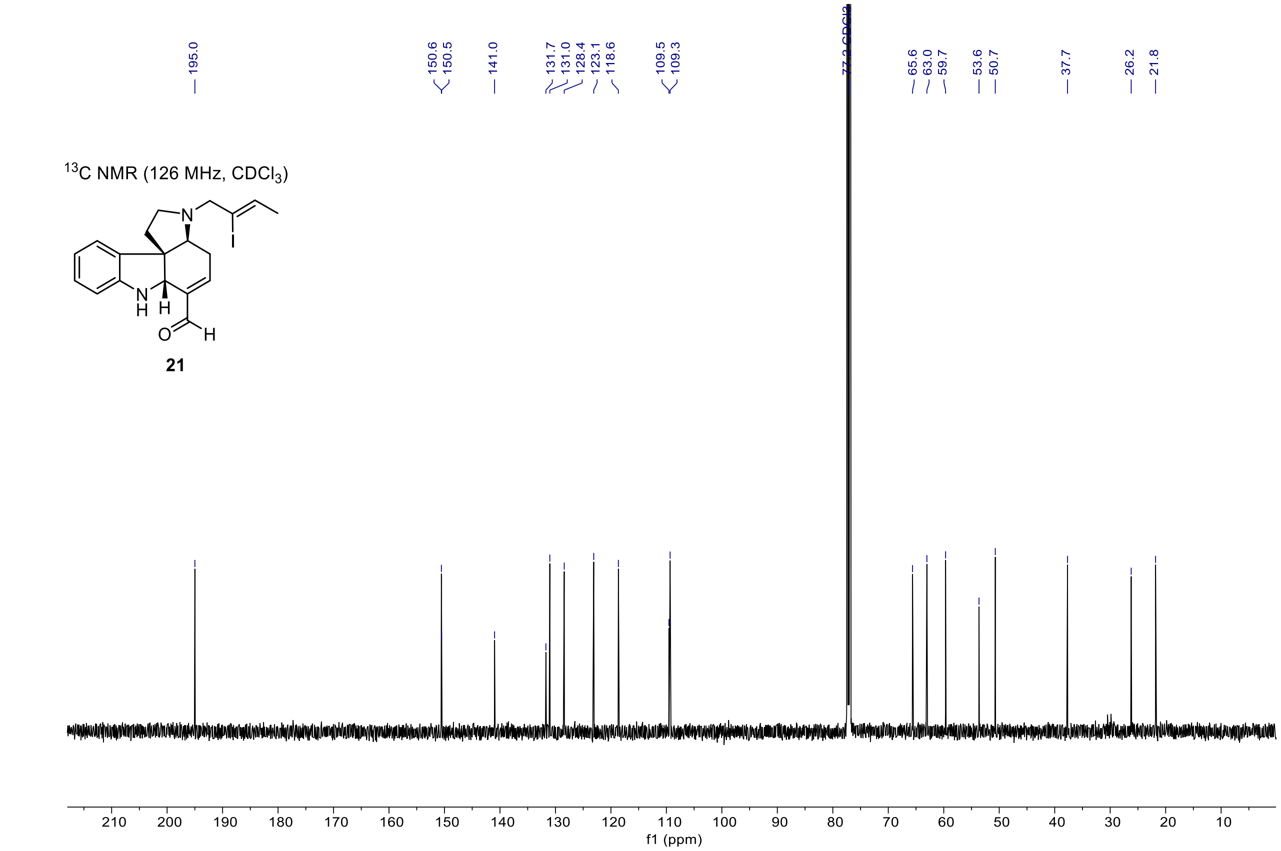

(3aS,5S,*11*bR,*E*)-12-Ethylidene-1,2,3a,4,5,7-hexahydro-3,5-ethanopyrrolo[2,3-d]*c*arbazole-6-carbaldehyde, **(–)-Norfluorocurarine** (Spectra in $CDCl_3$)

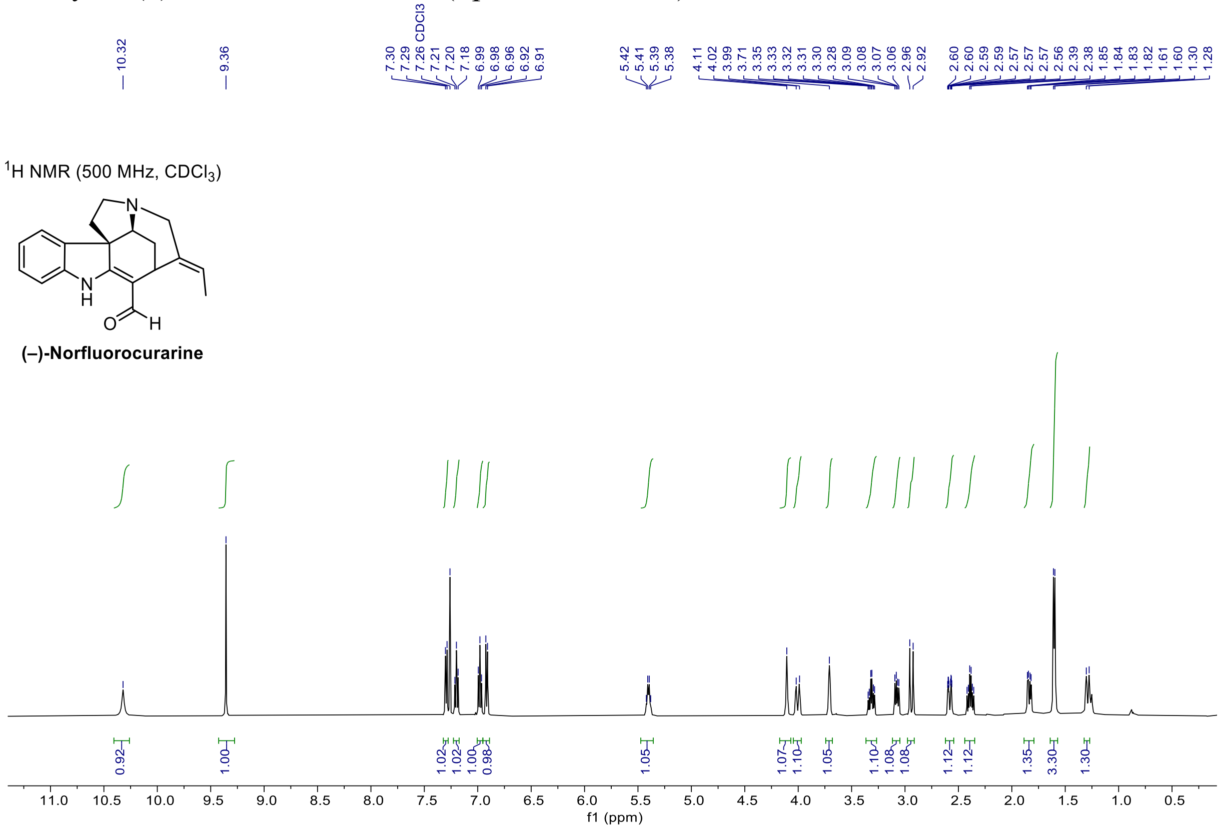

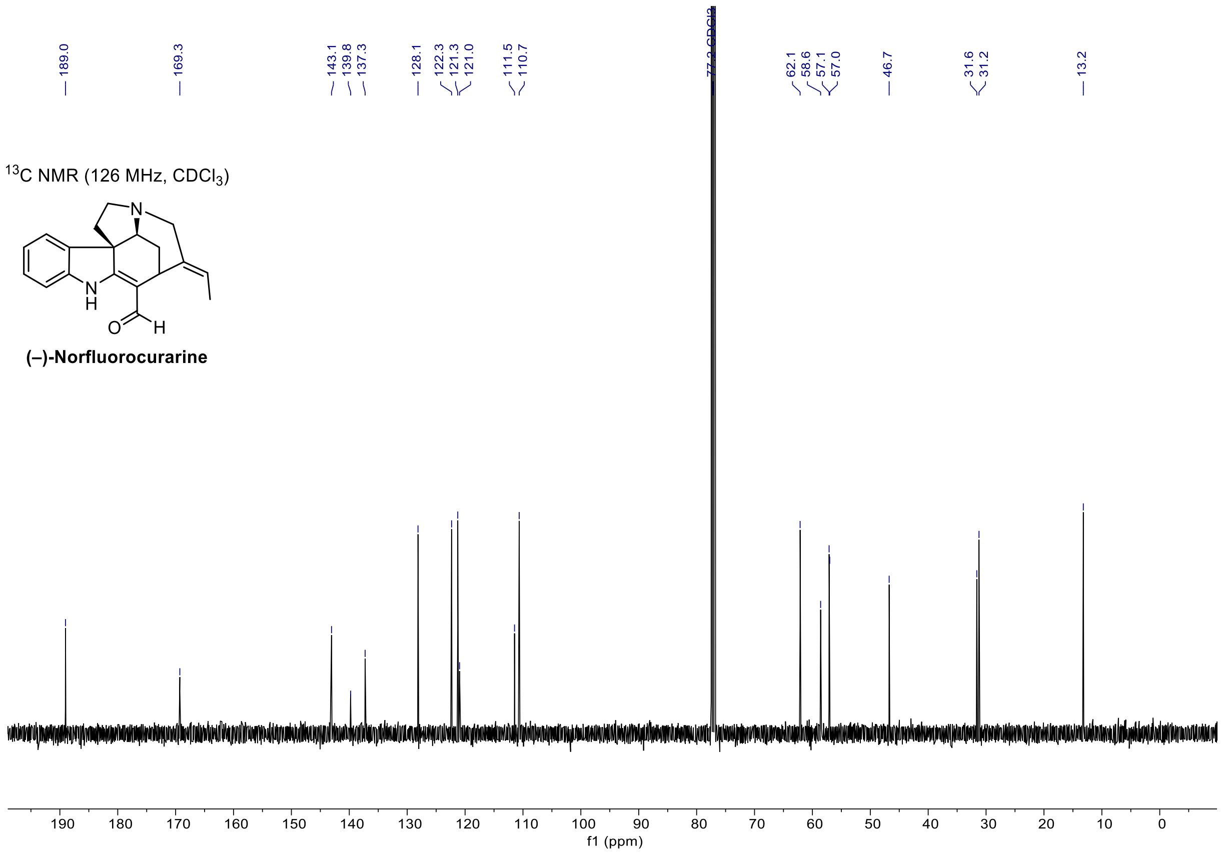

Methyl (3a*S*,5*R*,11b*R*,12*R*)-12-acetyl-12-hydroxy-1,2,3a,4,5,7-hexahydro-5,3-ethanopyrrolo[2,3-*d*]carbazole-6-carboxylate, **(–)-Lagumicine** (Spectra in $CDCl_3$)

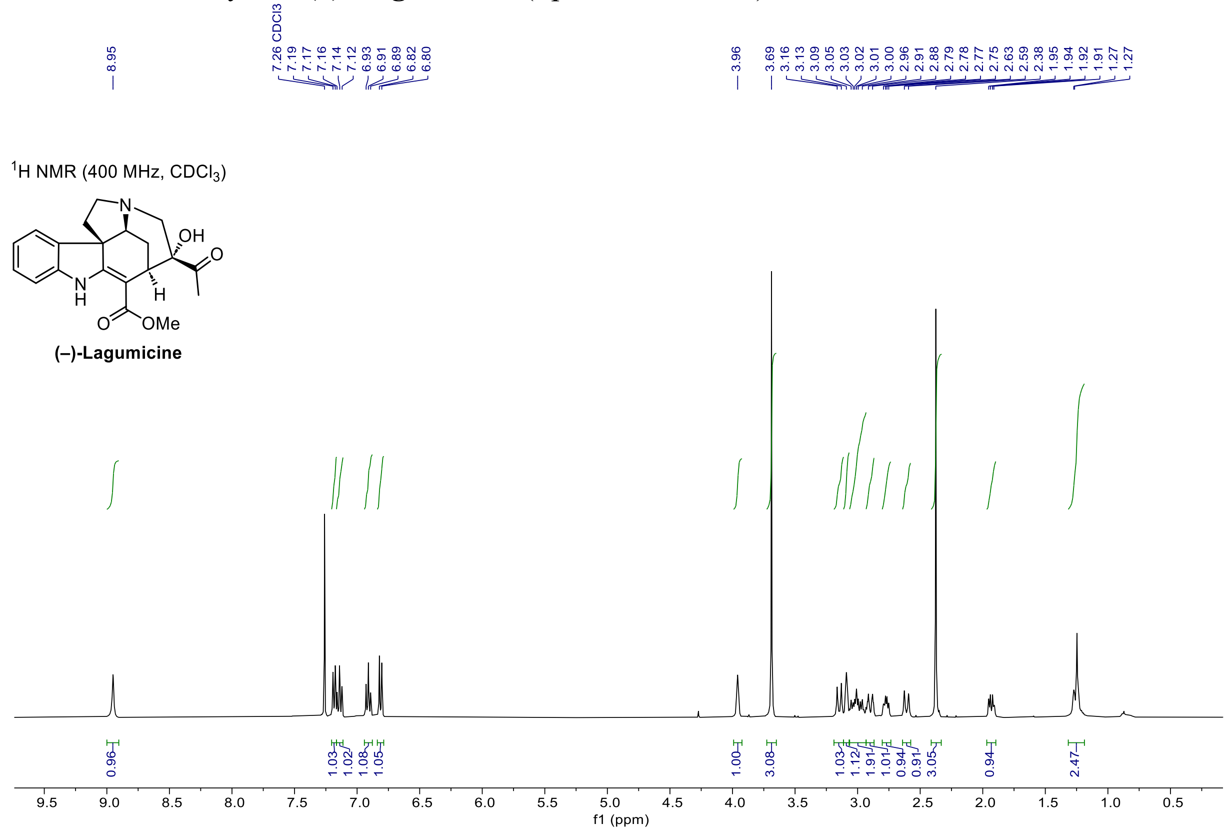

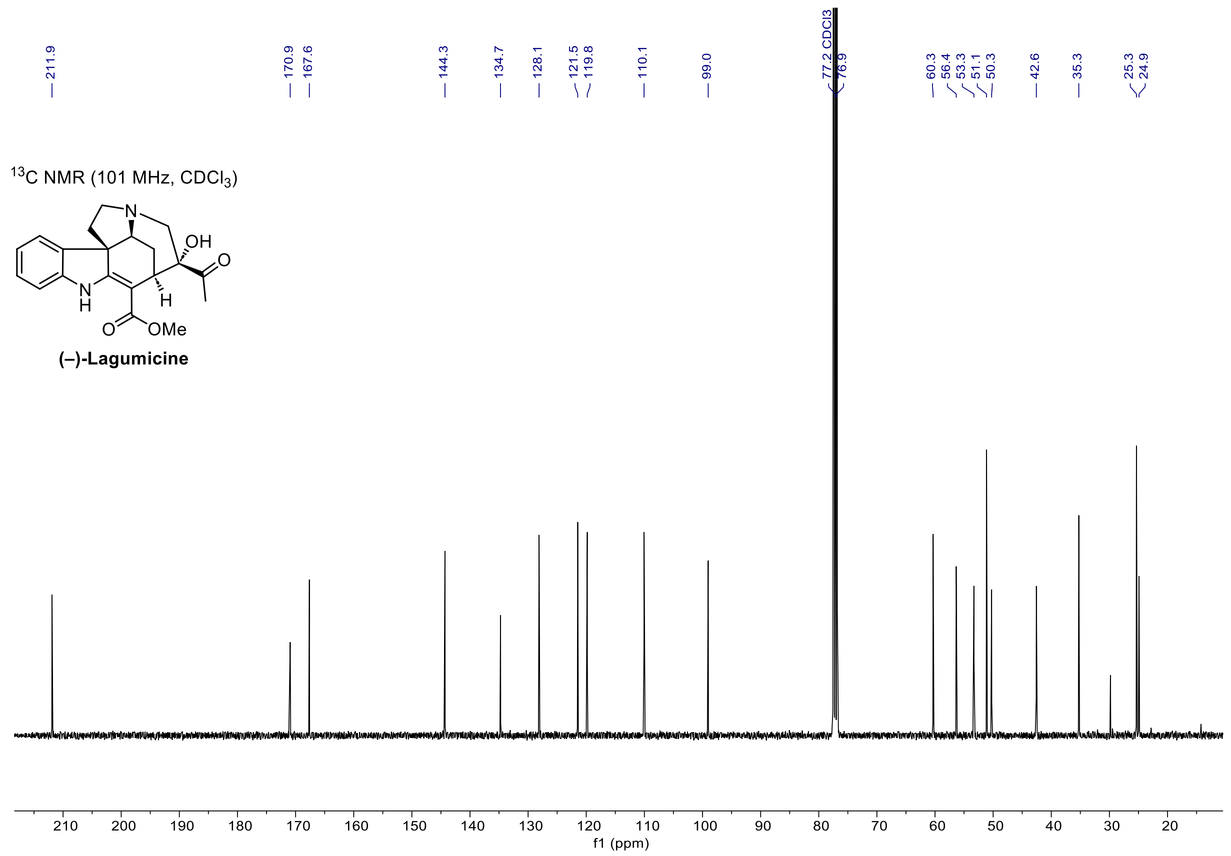

Methyl (3aS,5S,11bR,12S)-12-acetyl-1,2,3a,4,5,7-hexahydro-5,3-ethanopyrrolo[2,3-d]carbazole-6-carboxylate, **(–)-Alstolucine B** (Spectra in $CDCl_3$)

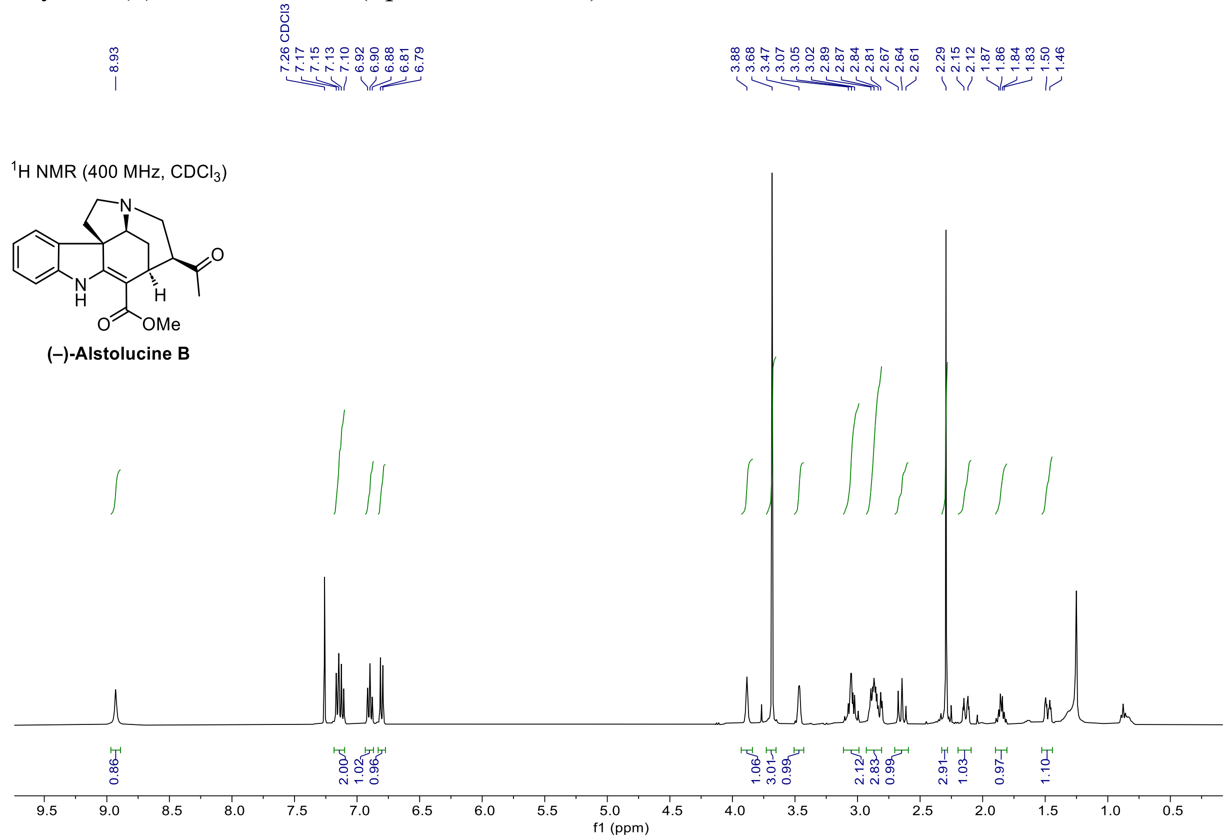

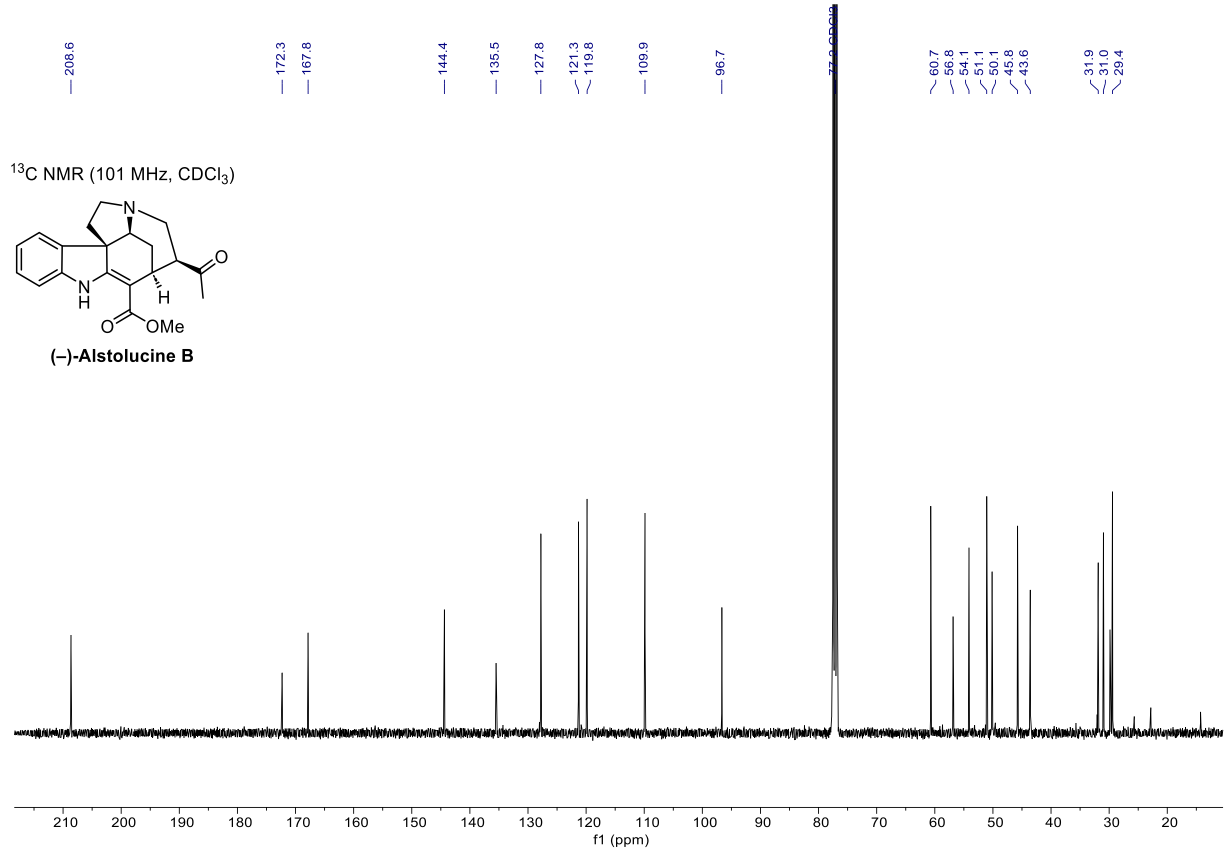

Methyl (3aS,5S,*1*1bR,*1*2R)-12-acetyl-1,2,3a,4,5,7-hexahydro-5,3-ethanopyrrolo[2,3-d]*c*arbazole-6-carboxylate, **(–)-Alstolucine F** (Spectra in $CDCl_3$)

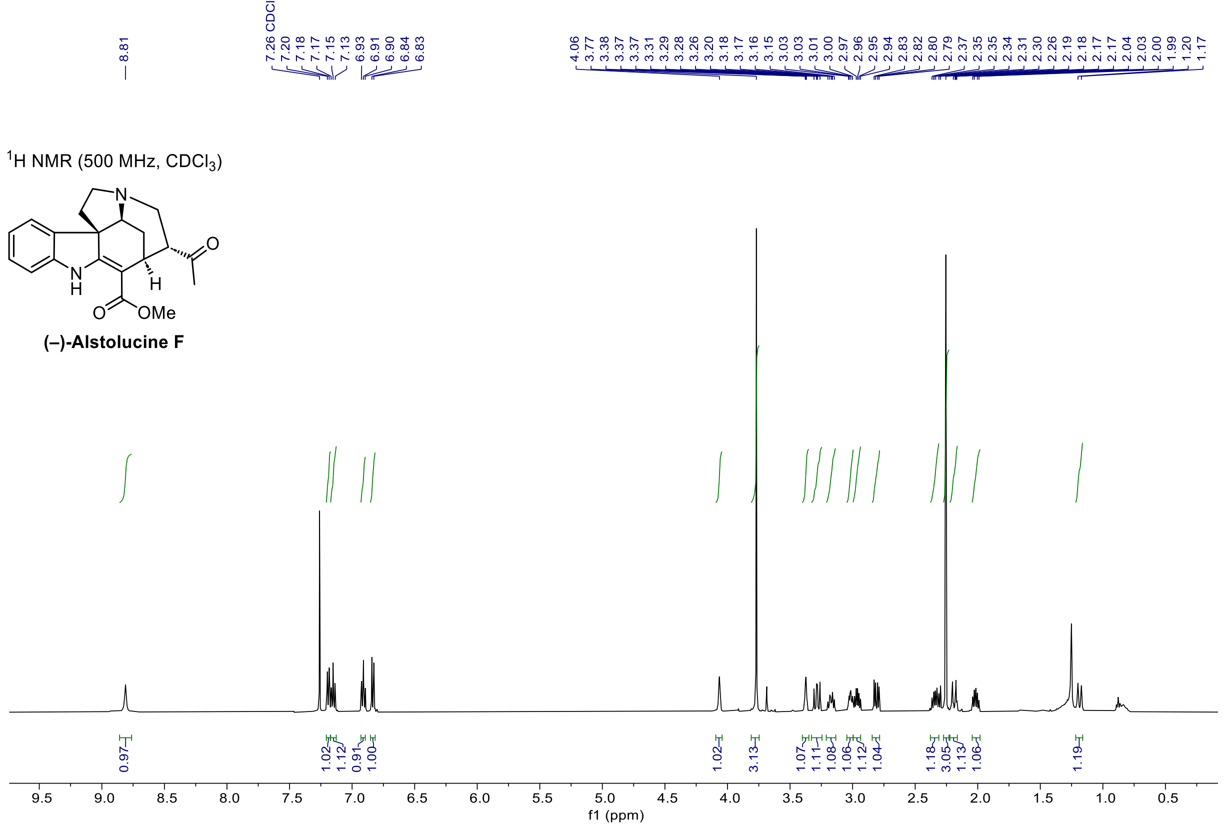

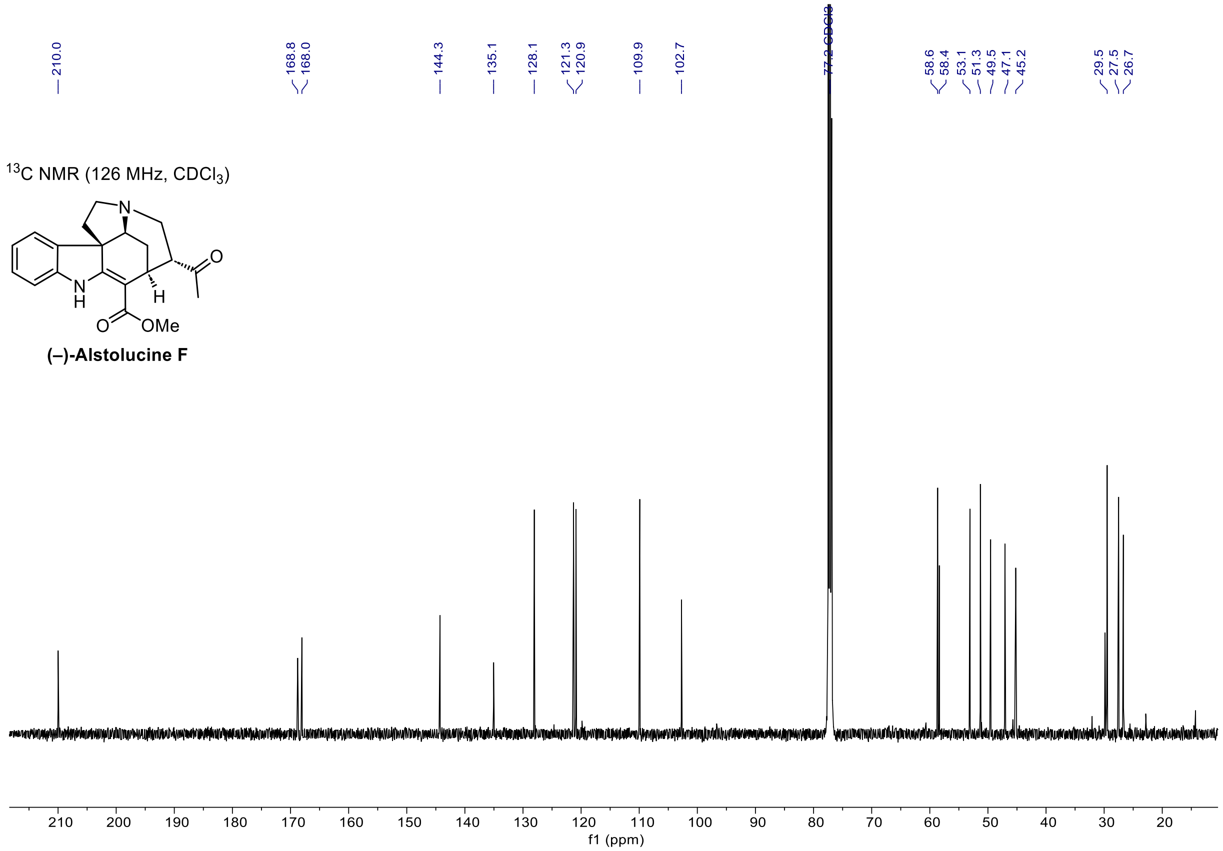

Methyl (3aS,5S,*1*1bR,*1*2S)-12-((S)-1-hydroxyethyl)-1,2,3a,4,5,7-hexahydro-5,3-ethanopyrrolo[2,3-d]*c*arbazole-6-carboxylate, **(–)-Echitamidine** (Spectra in $CDCl_3$)

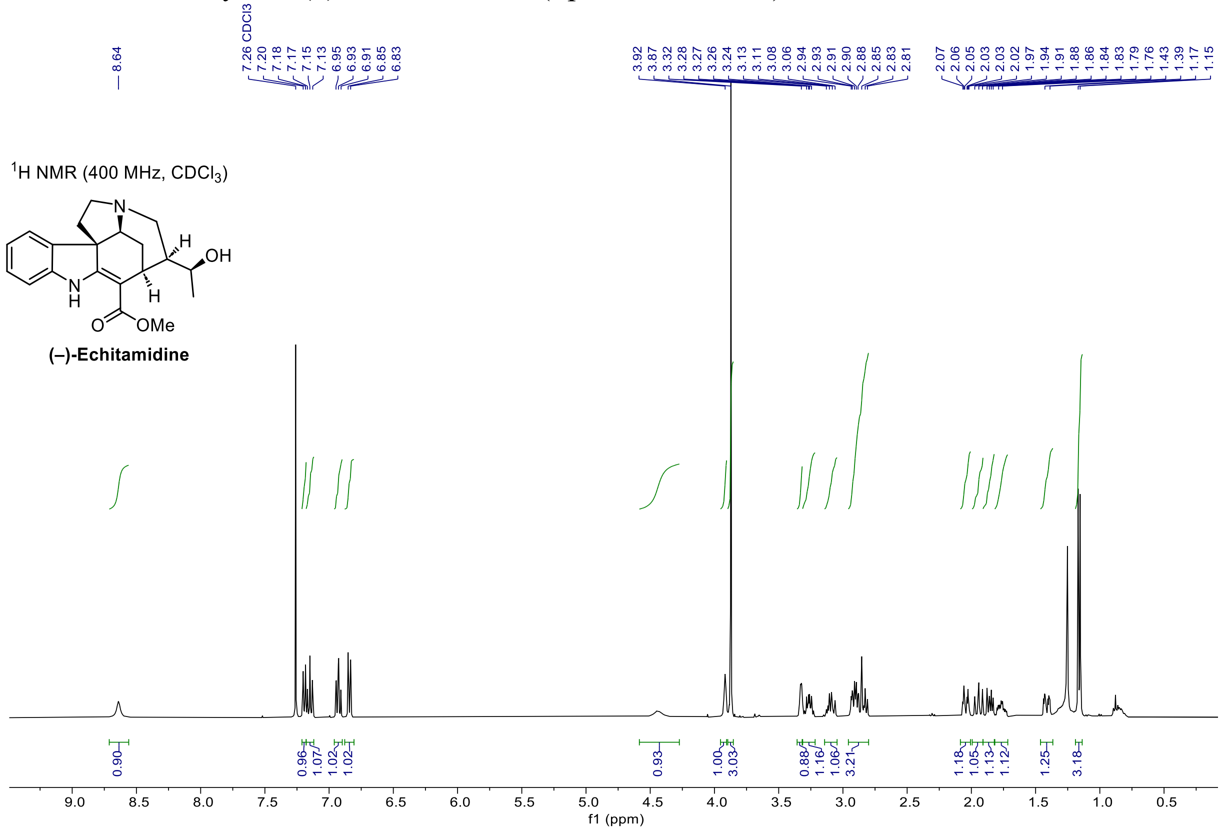

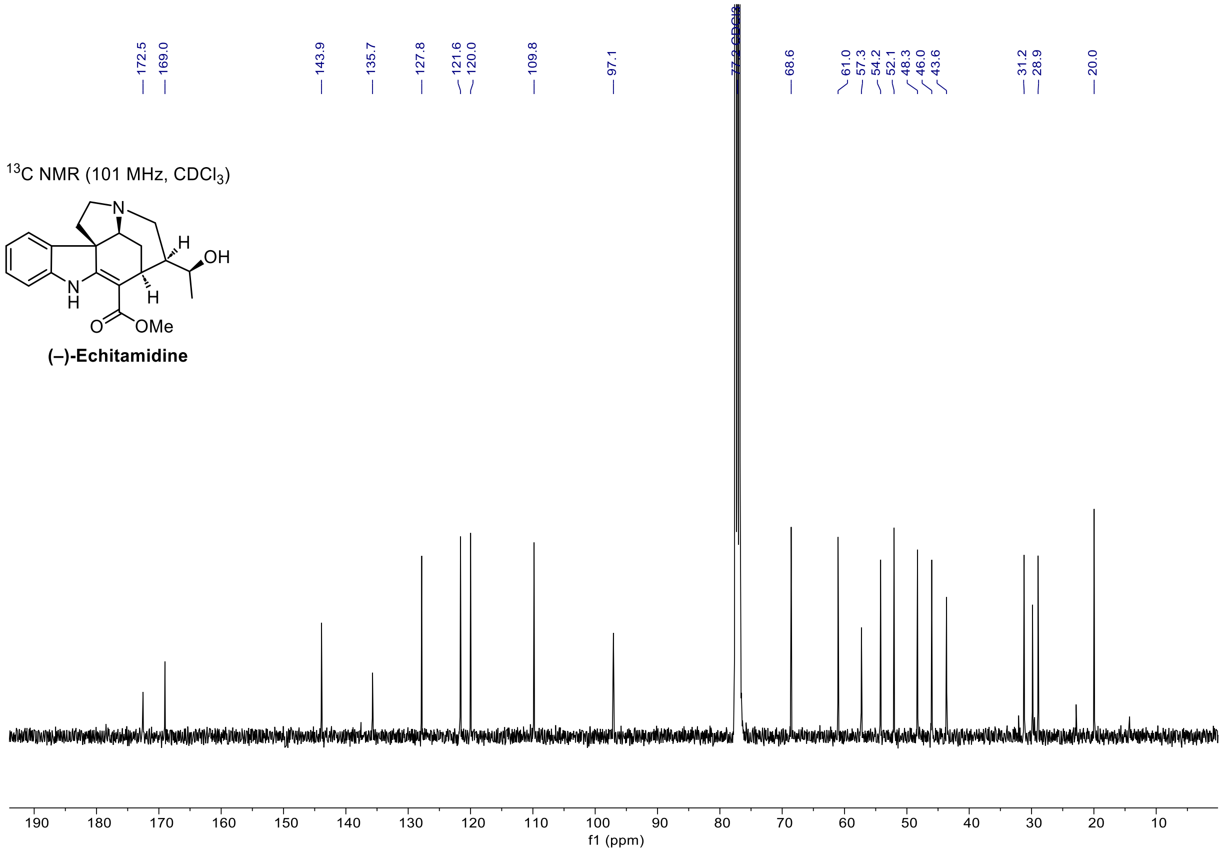

(*Z*)-4-Bromo-3-iodobut-2-en-1-yl benzoate, **22** (Spectra in $CDCl_3$)

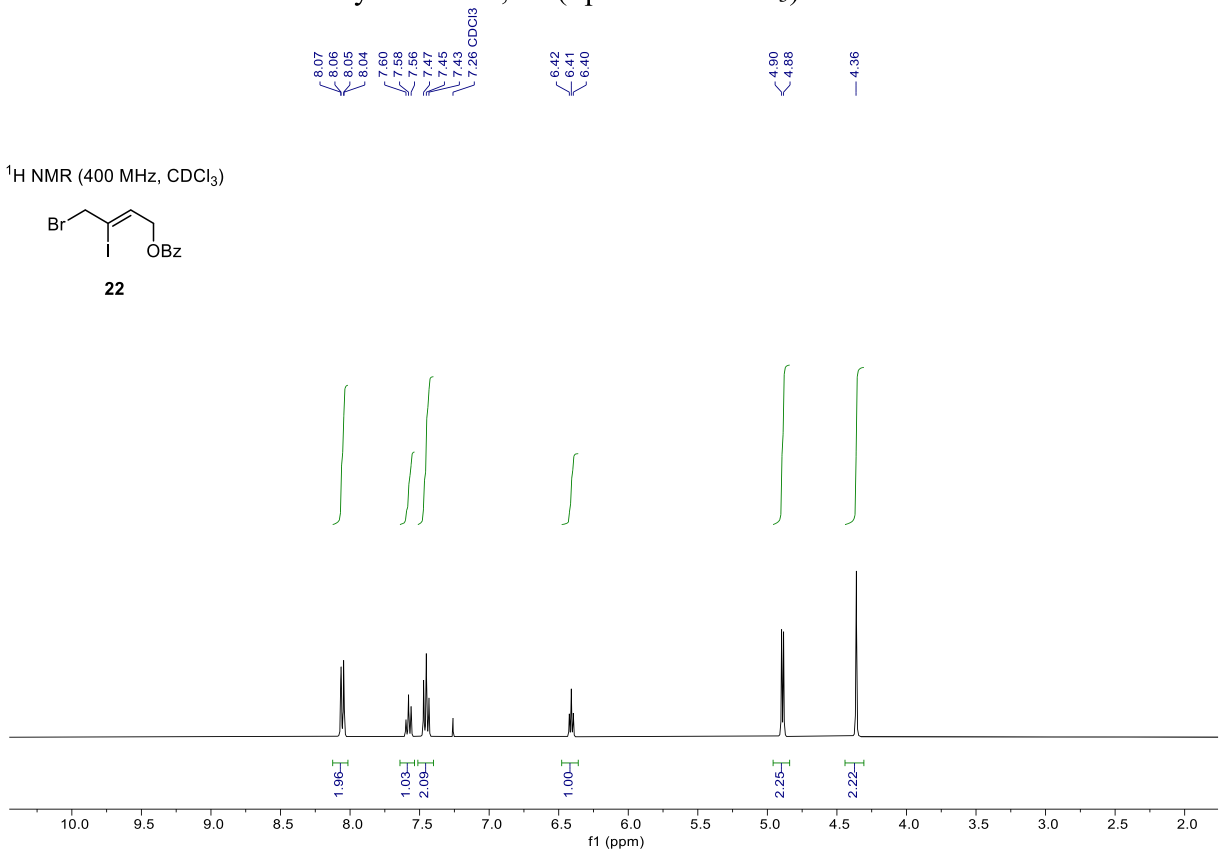

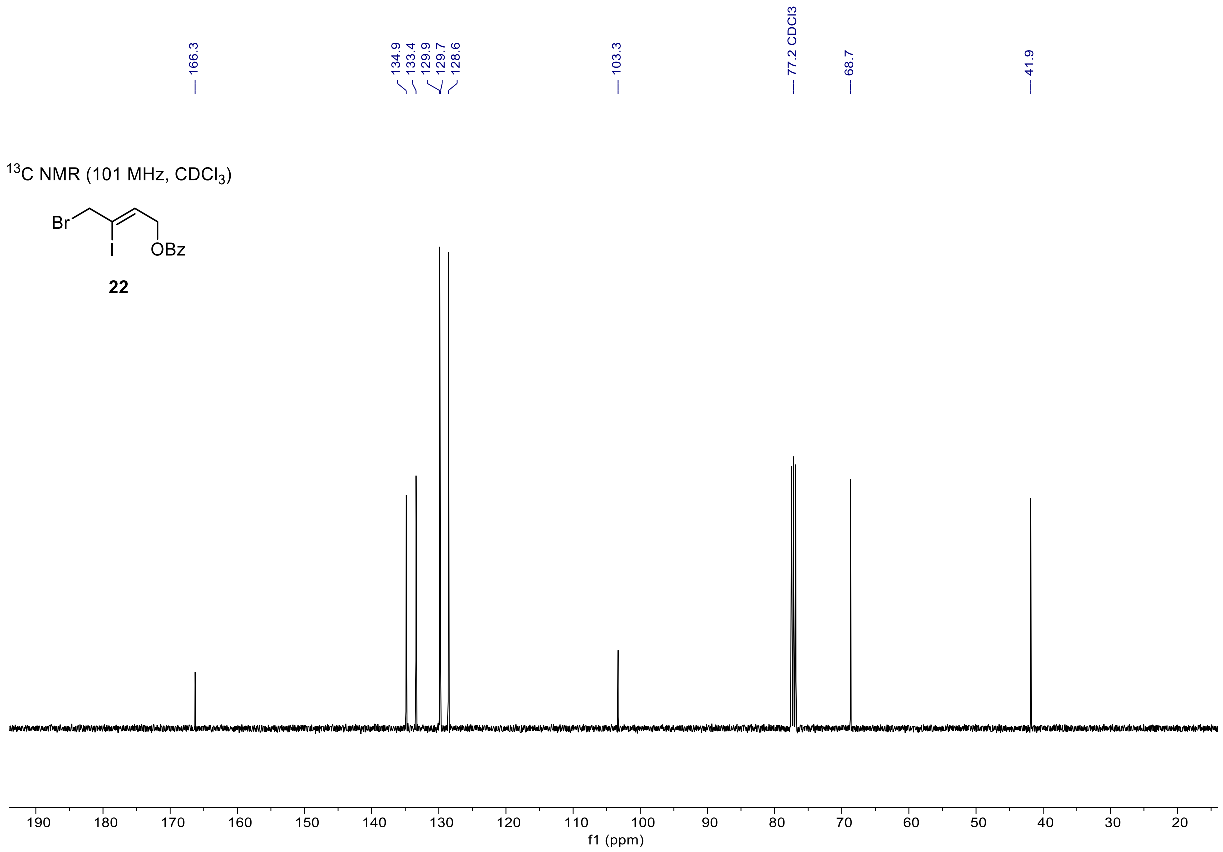

(*Z*)-3-iodo-4-((2-(1-(4-methoxybenzyl)-1*H*-indol-3-yl)ethyl)amino)but-2-en-1-yl benzoate, **S8**
(Spectra in $CDCl_3$)

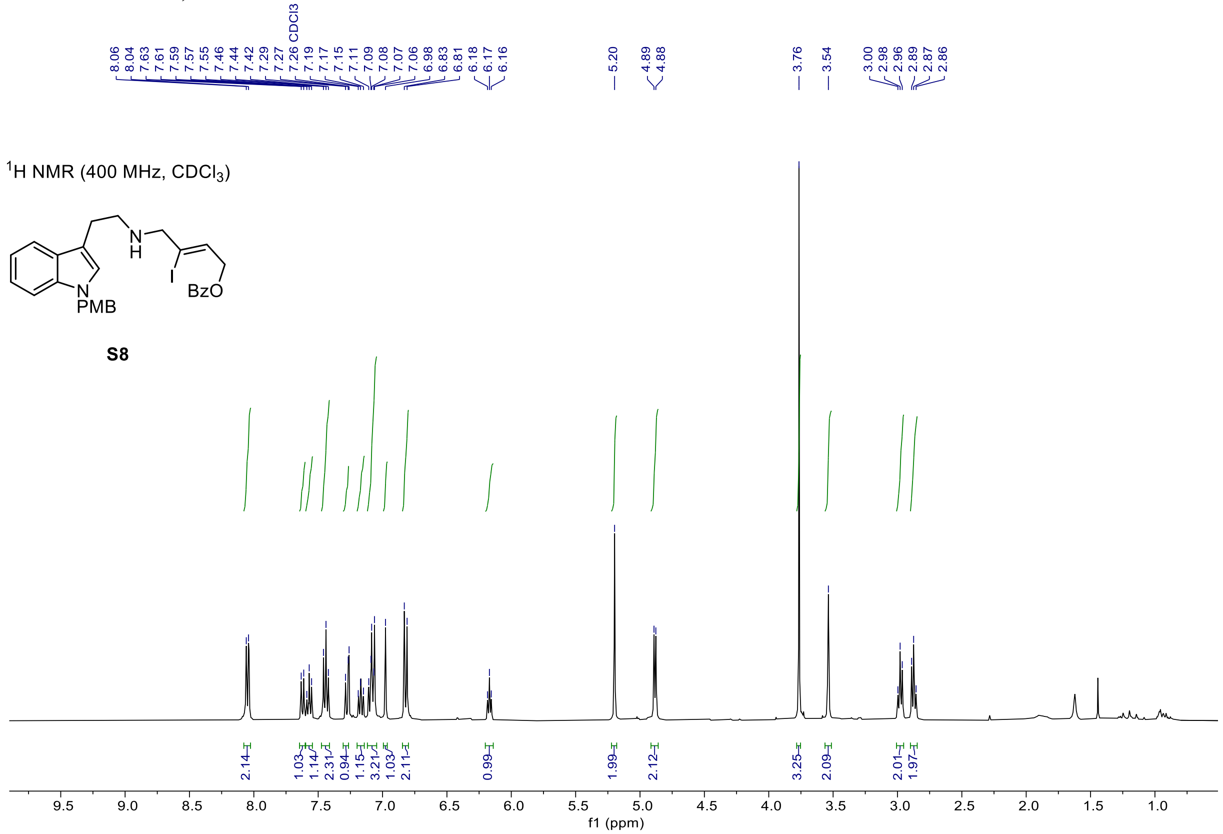

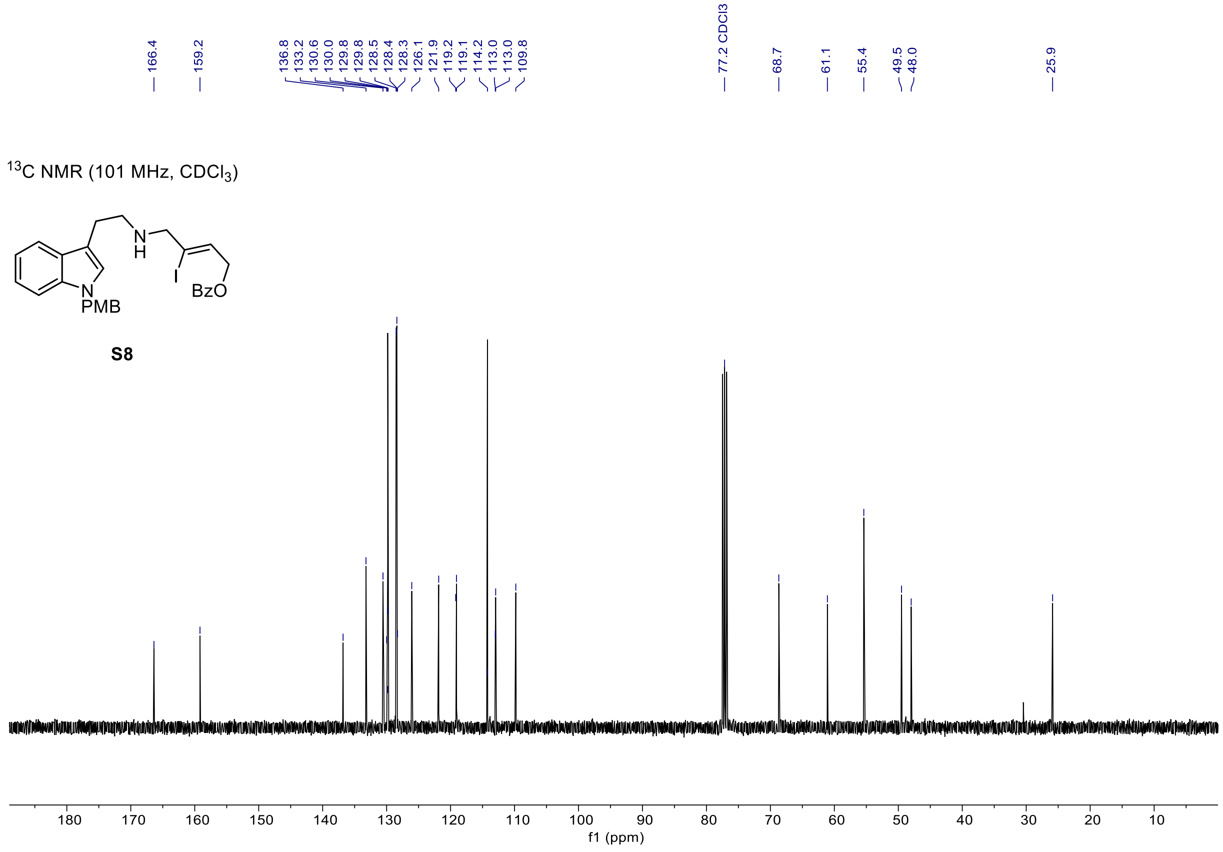

(*Z*)-4-((3a*S*,6a*R*,11b*R*)-6-((3a*S*,6*R*,7a*R*)-8,8-Dimethyl-2,2-dioxidohexahydro-3*H*-3a,6-methanobenzo[*c*]isothiazole-1-carbonyl)-7-(4-methoxybenzyl)-1,2,3a,4,6a,7-hexahydro-3*H*-pyrrolo[2,3-*d*]carbazol-3-yl)-3-iodobut-2-en-1-yl benzoate, **23** (Spectra in $CDCl_3$)

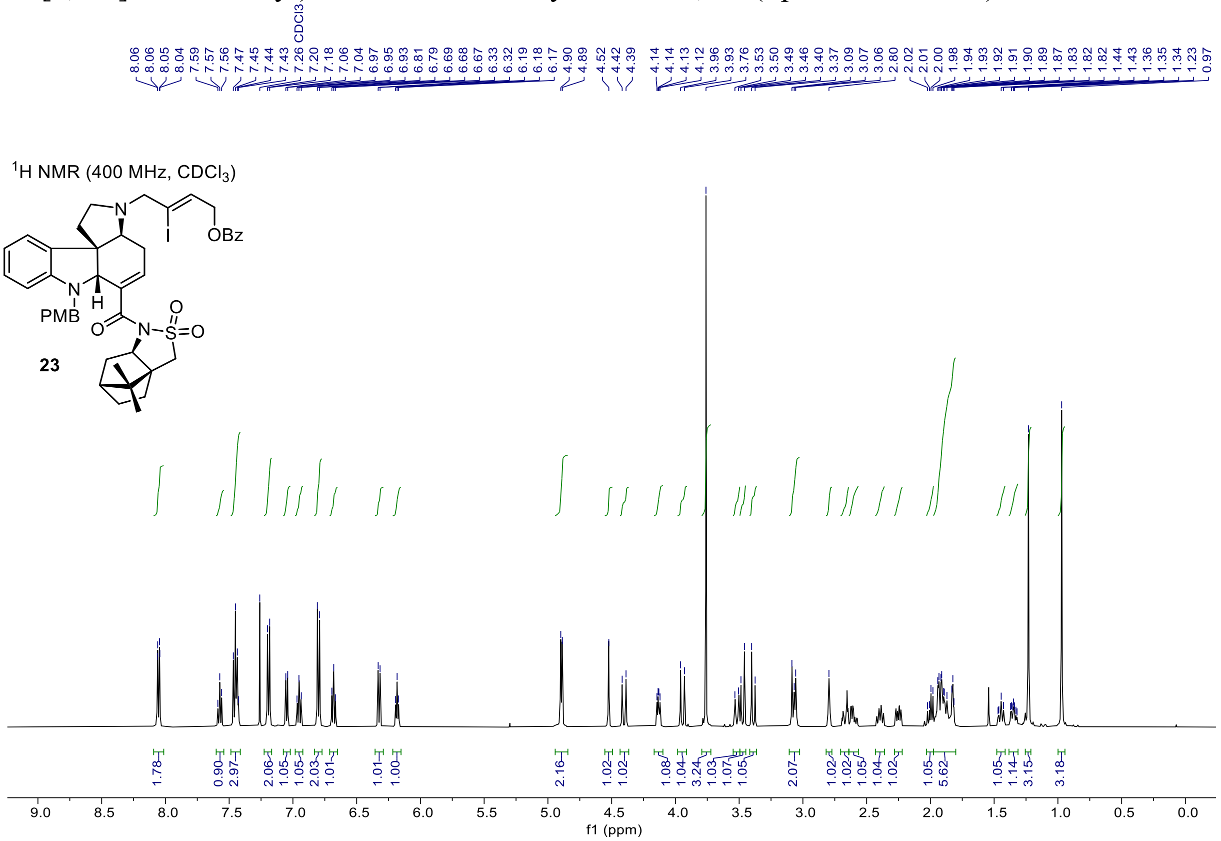

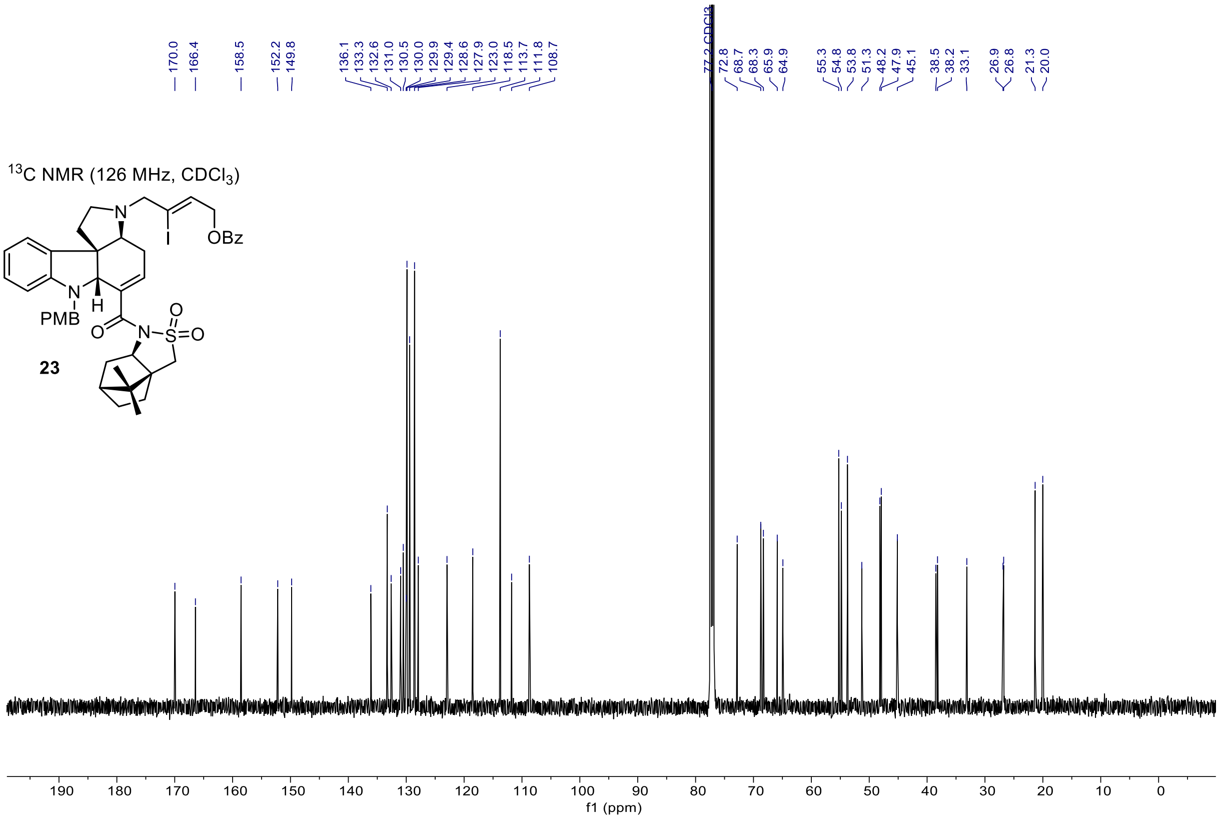

(*Z*)-4-((3a*R*,6a*R*,11b*R*)-6-((3a*S*,6*R*,7a*R*)-8,8-Dimethyl-2,2-dioxidohexahydro-3*H*-3a,6-methanobenzo[*c*]isothiazole-1-carbonyl)-7-(4-methoxybenzyl)-1,2,3a,4,6a,7-hexahydro-3*H*-pyrrolo[2,3-*d*]carbazol-3-yl)-3-iodobut-2-en-1-yl benzoate, **23’** (Spectra in $CDCl_3$)

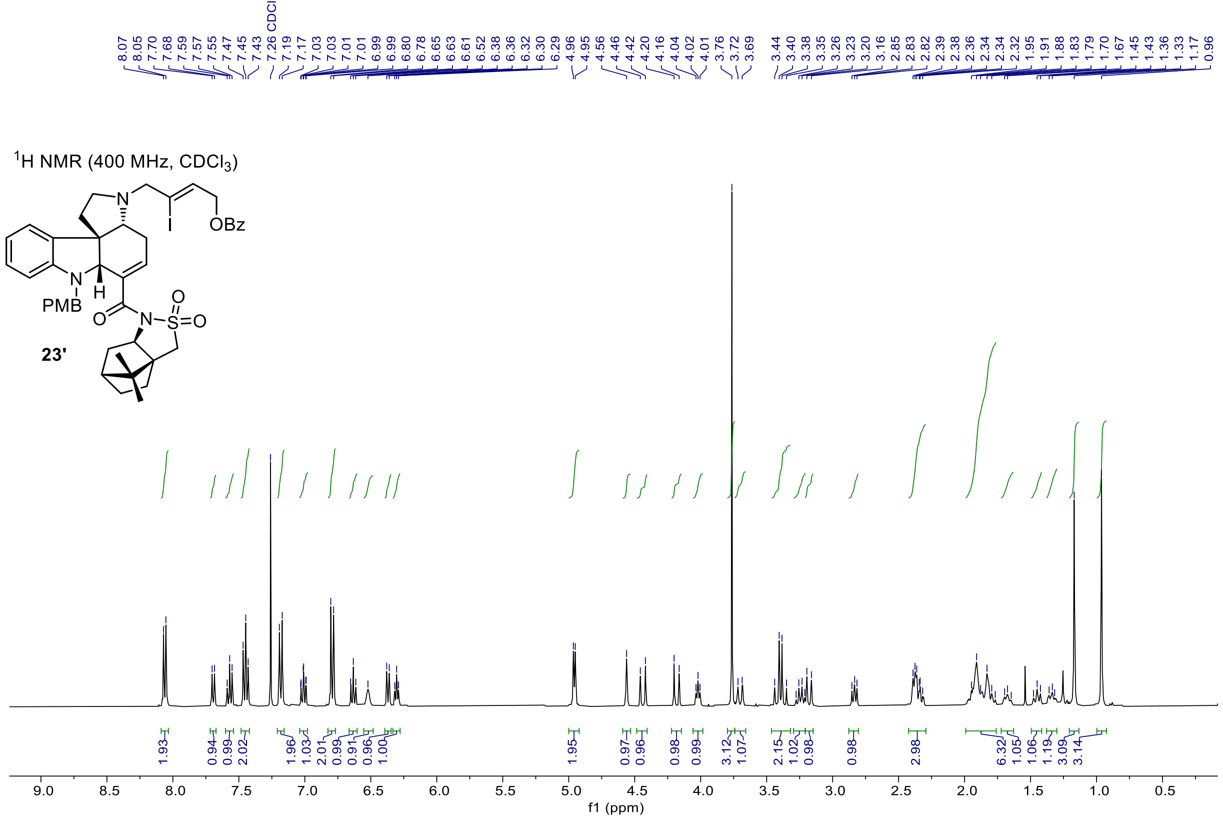

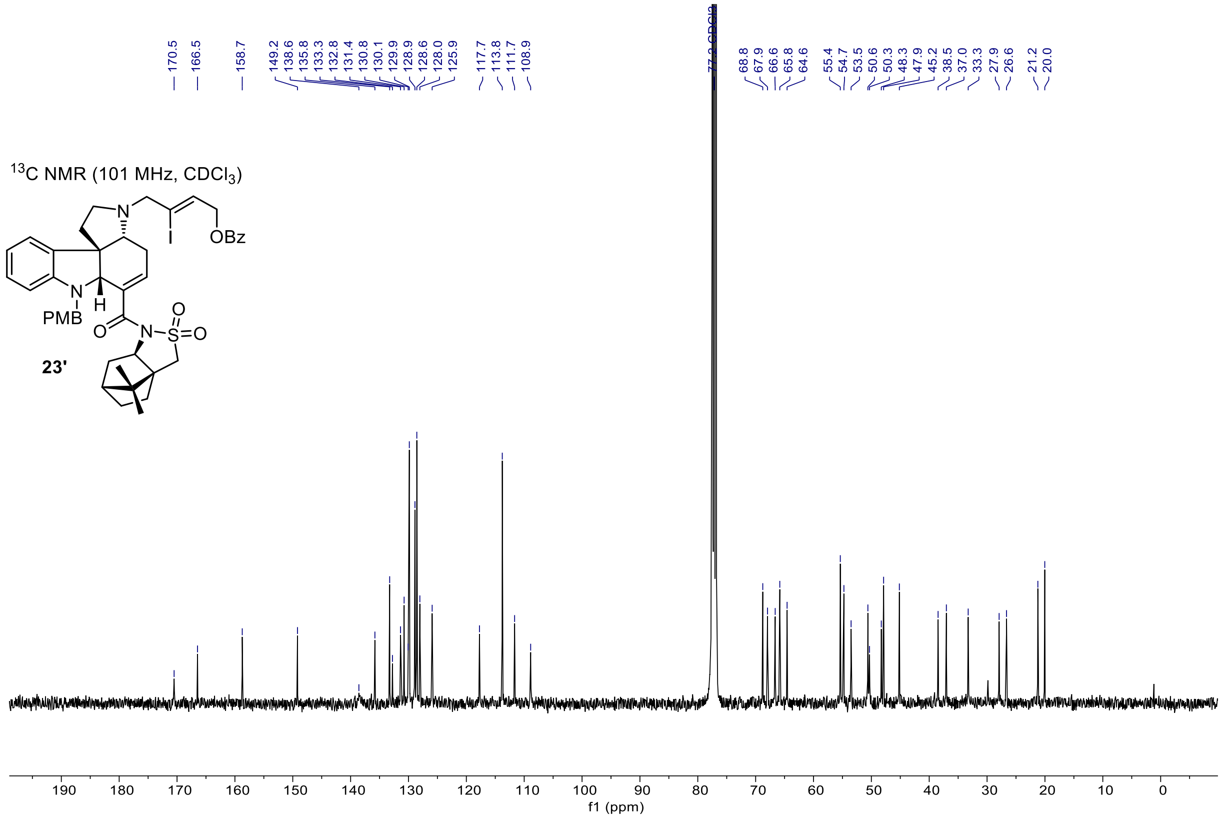

(*Z*)-4-((3a*S*,6a*R*,11b*S*)-6-((3a*S*,6*R*,7a*R*)-8,8-Dimethyl-2,2-dioxidohexahydro-3*H*-3a,6-methanobenzo[*c*]isothiazole-1-carbonyl)-1,2,3a,4,6a,7-hexahydro-3*H*-pyrrolo[2,3-*d*]carbazol-3-yl)-3-iodobut-2-en-1-yl benzoate, **S9** (Spectra in $CDCl_3$)

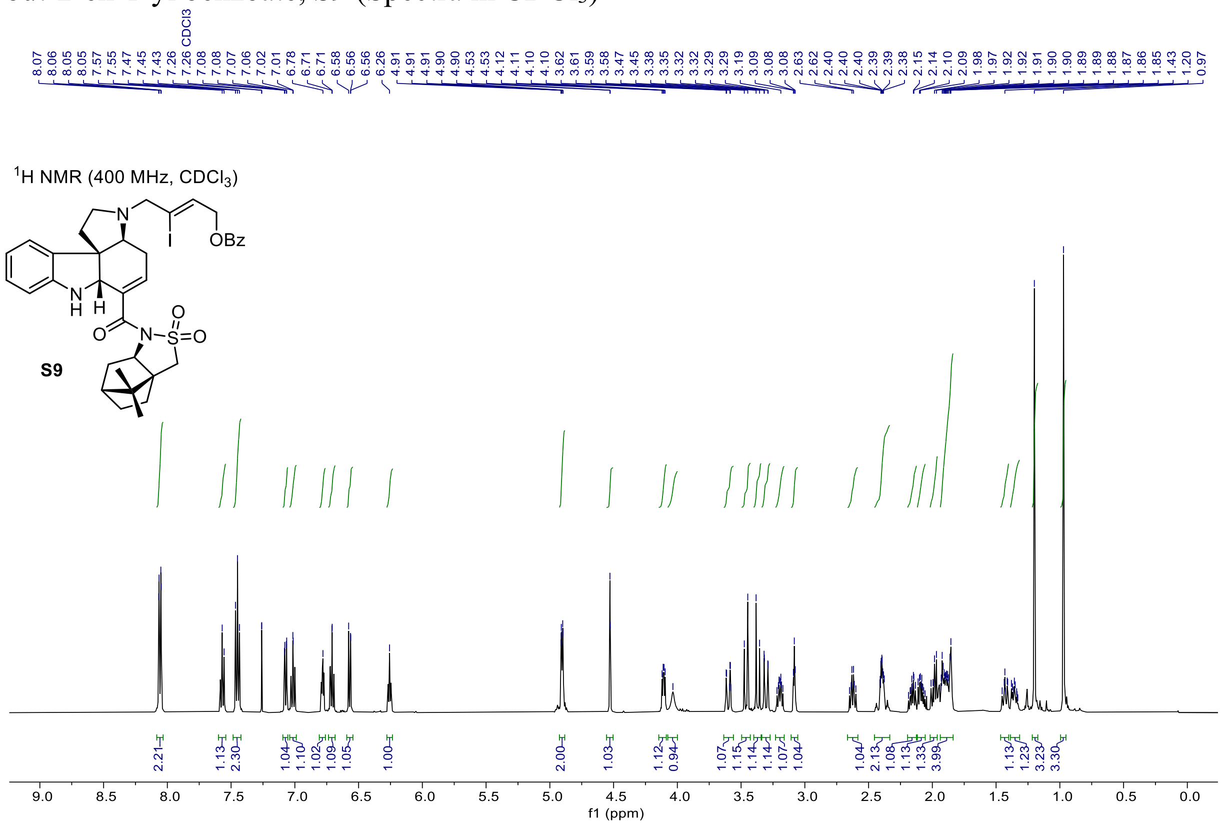

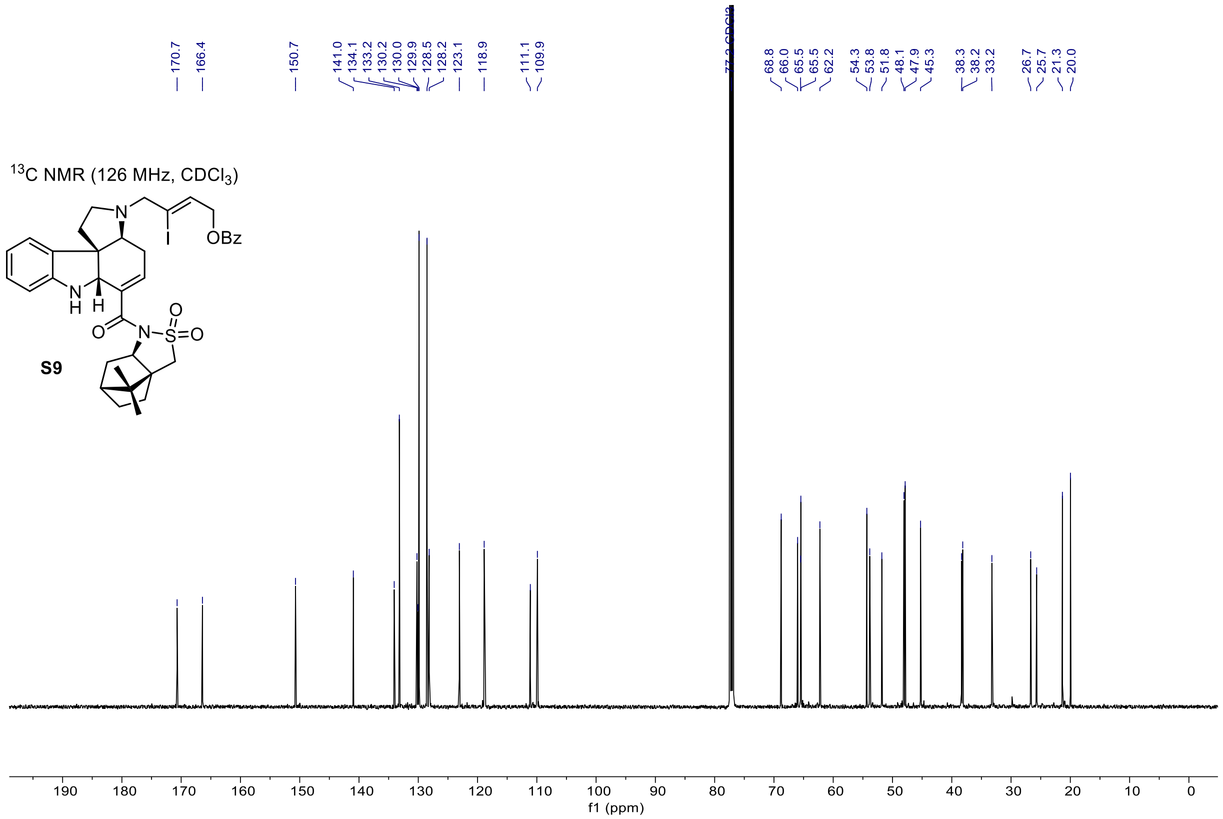

(*Z*)-4-((3a*S*,6a*R*,11b*R*)-6-(Hydroxymethyl)-7-(4-methoxybenzyl)-1,2,3a,4,6a,7-hexahydro-3*H*-pyrrolo[2,3-*d*]carbazol-3-yl)-3-iodobut-2-en-1-ol, **24** (Spectra in $CDCl_3$)

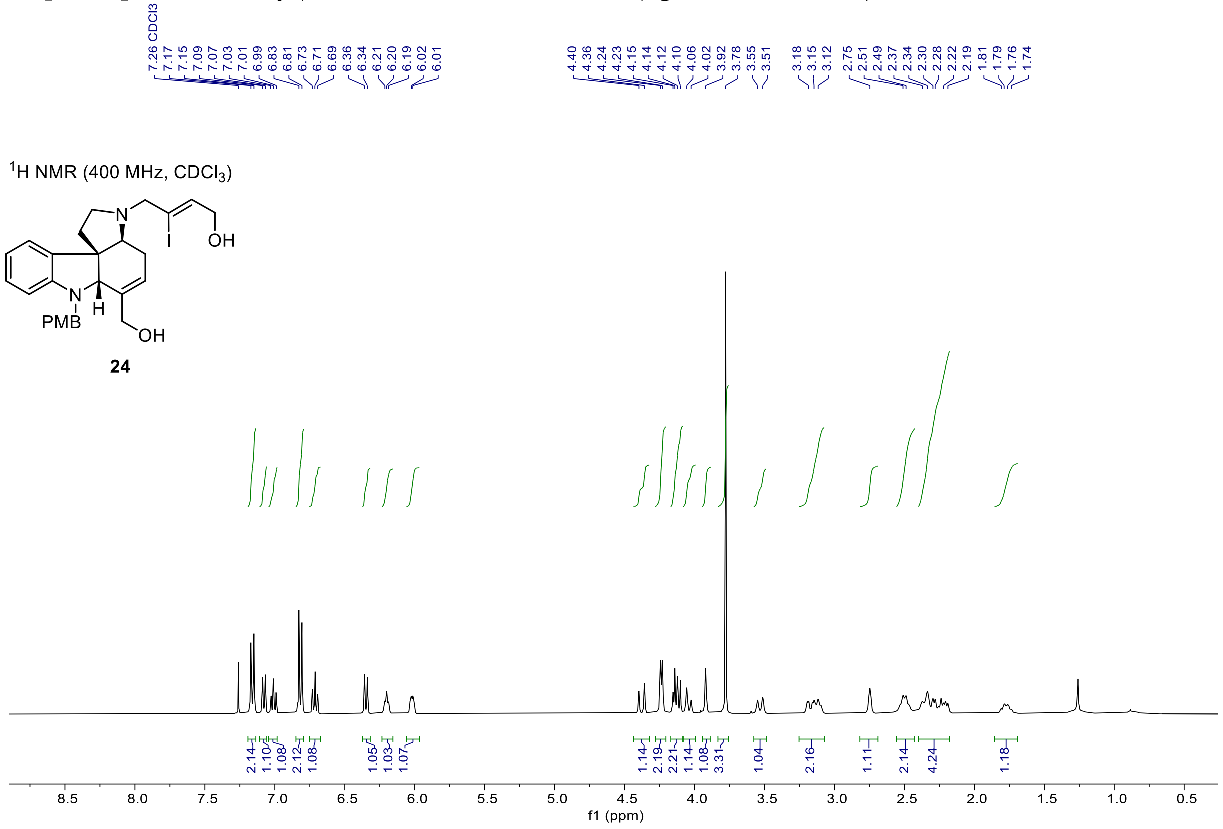

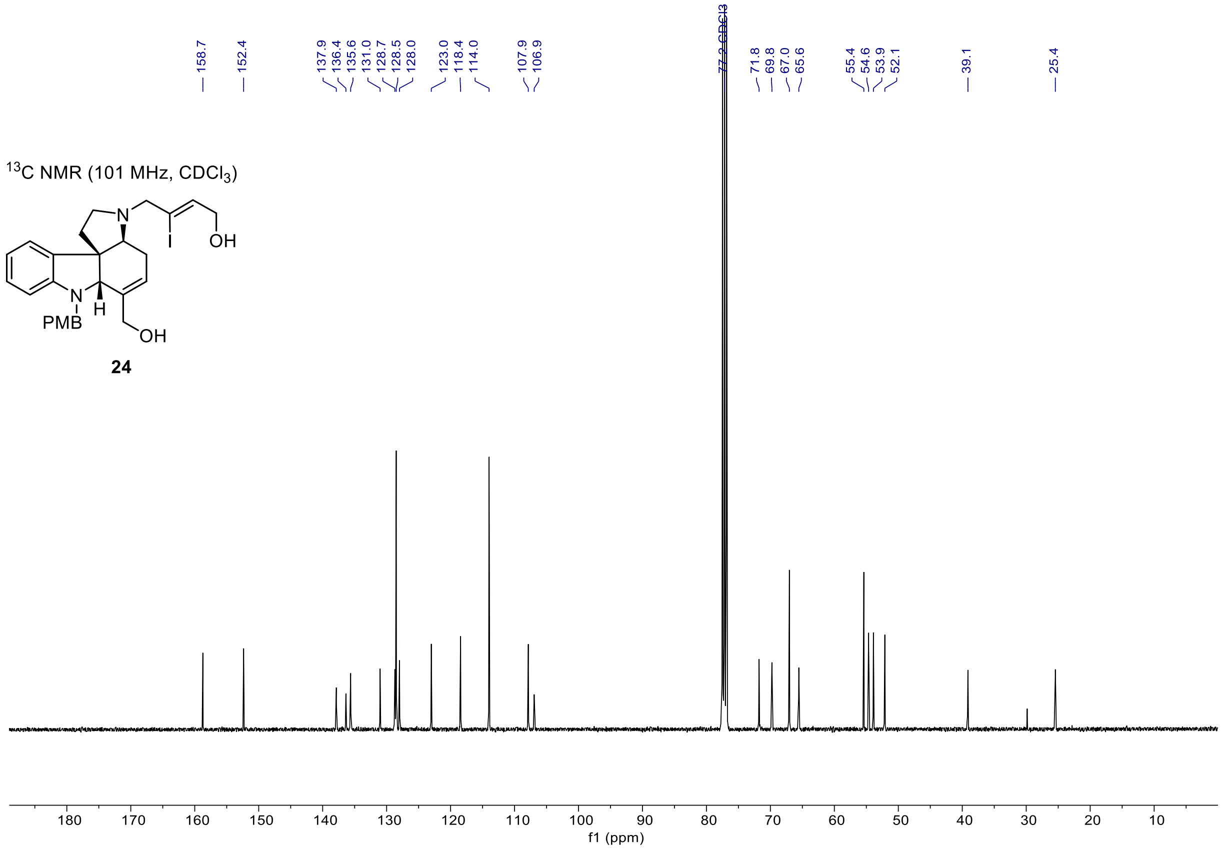

(4b*R*,7a*S*,8a*R*,13*R*,13a*R*,13b*S*)-14-(4-Methoxybenzyl)-5,6,7a,8,8a,11,13,13a,13b,14-decahydro-7,9-methanooxepino[3,4-*a*]pyrrolo[2,3-*d*]carbazol-13-ol, **S10** (Spectra in $CDCl_3$)

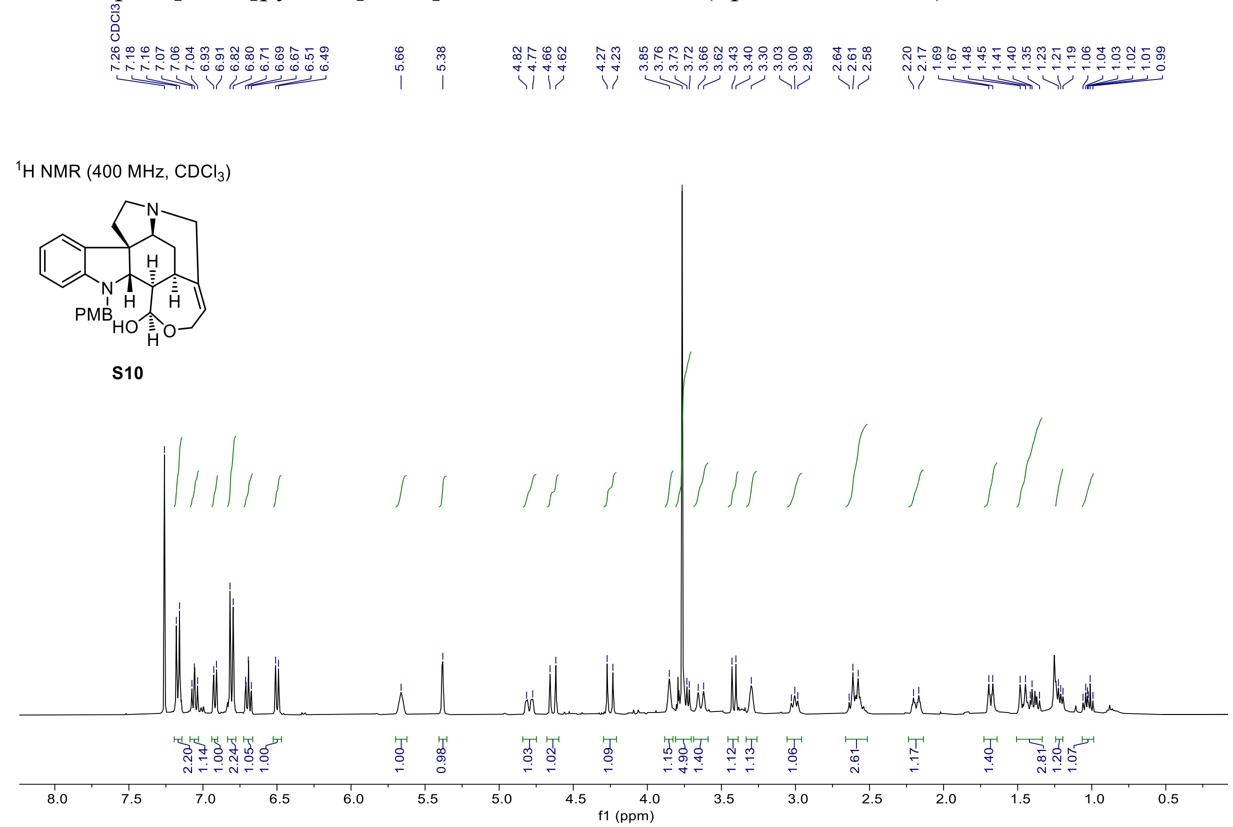

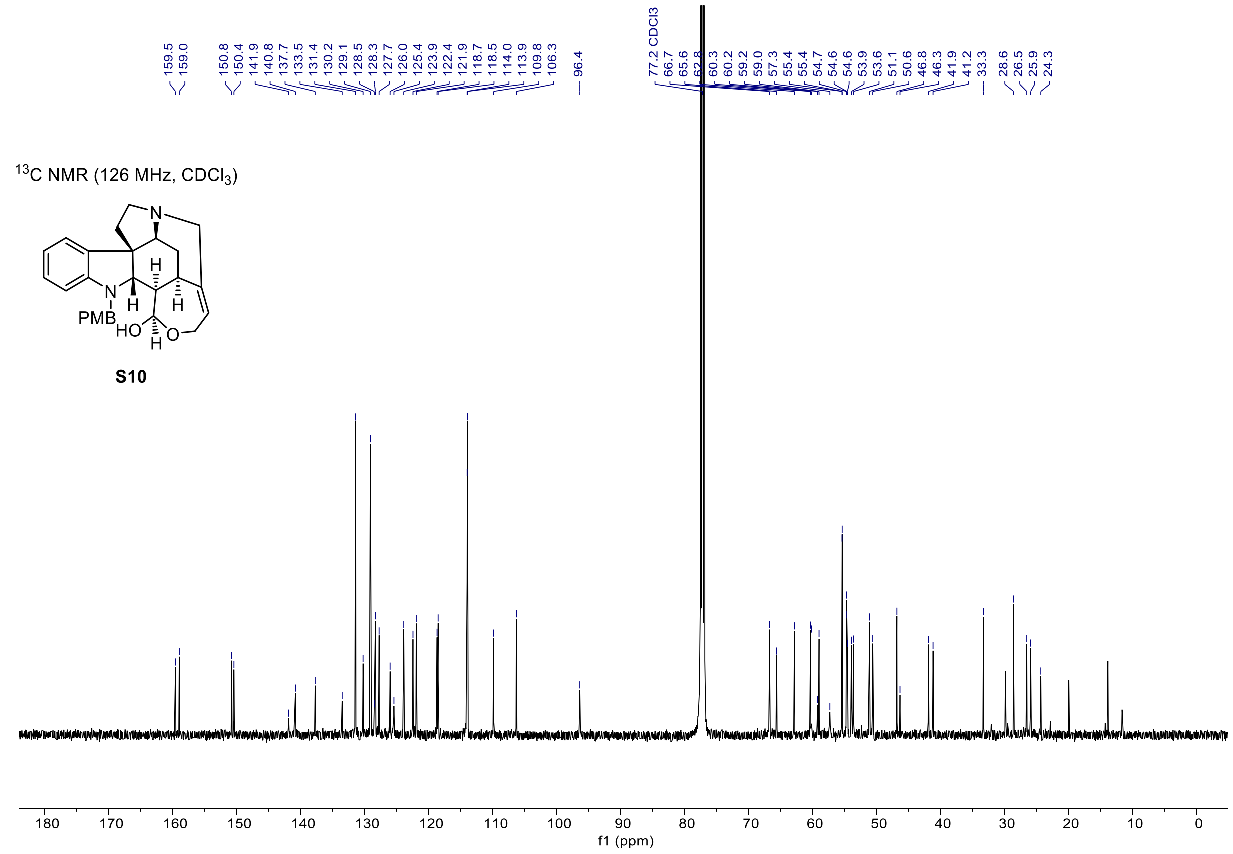

(4aR,*4*a1R,*5*aS,*8*aR,*8*a1S,*1*5aS)-2,4a,4a1,5,5a,7,8,8a1,15,15a-Decahydro-14H-*4*,6-methanoindolo[3,2,1-ij]*o*xepino[2,3,4-de]*p*yrrolo[2,3-h]*q*uinolin-14-one, **(–)-Strychnine** (Spectra in $CDCl_3$)

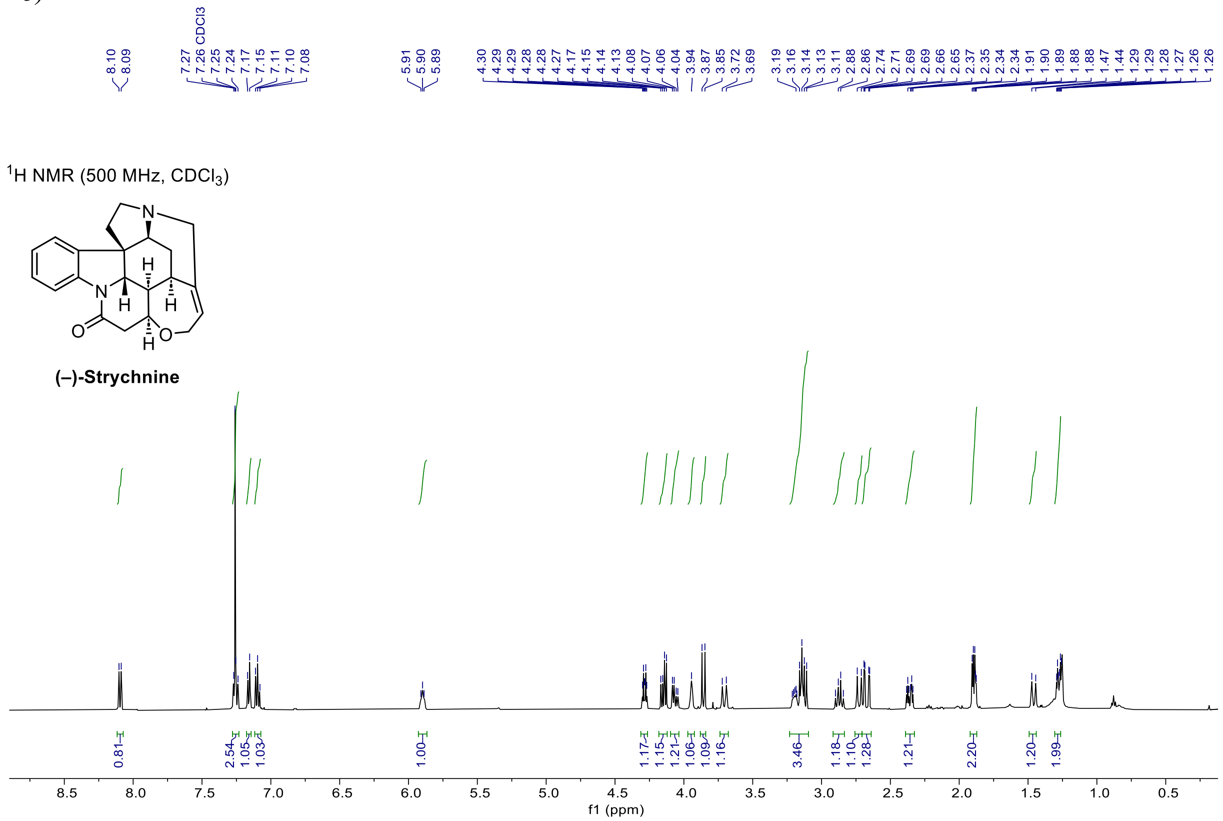

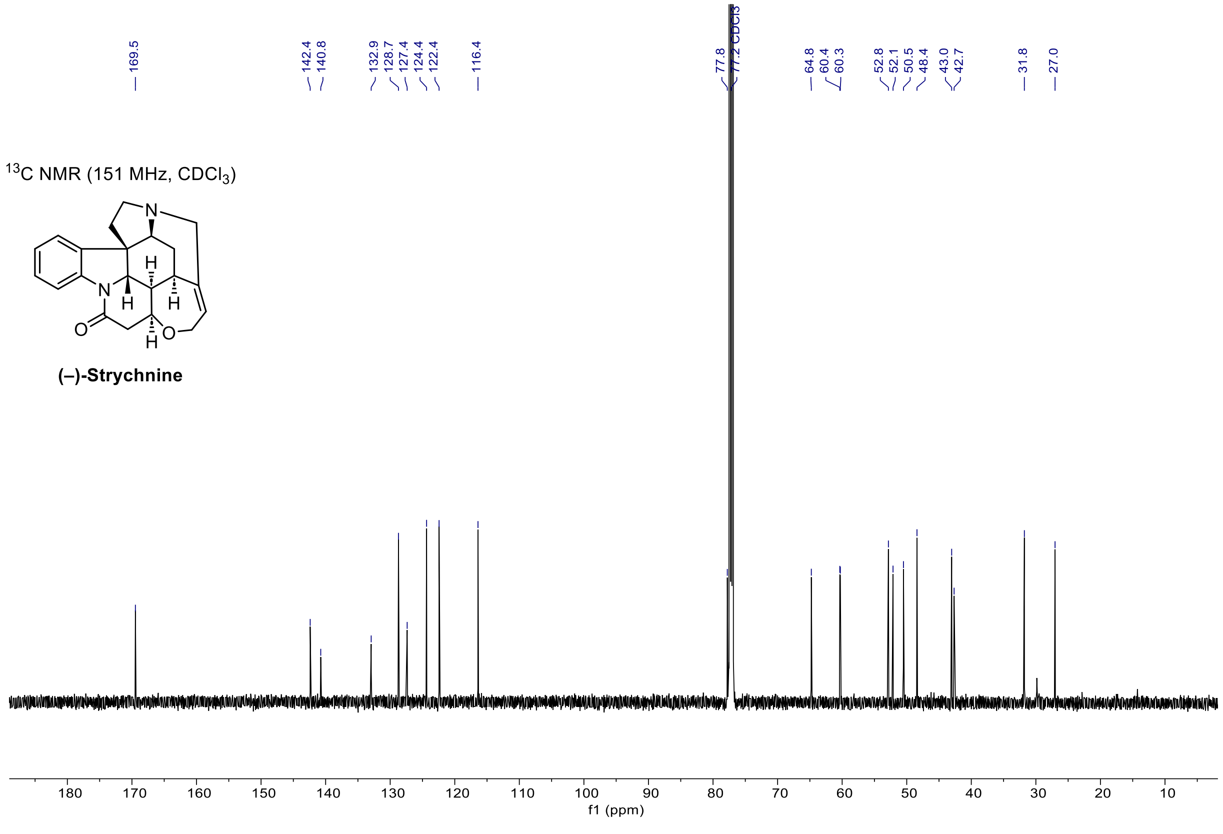

2-Iodo-4,5-dimethoxy-*N*-(4-methoxybenzyl)aniline, **26** (Spectra in $CDCl_3$)

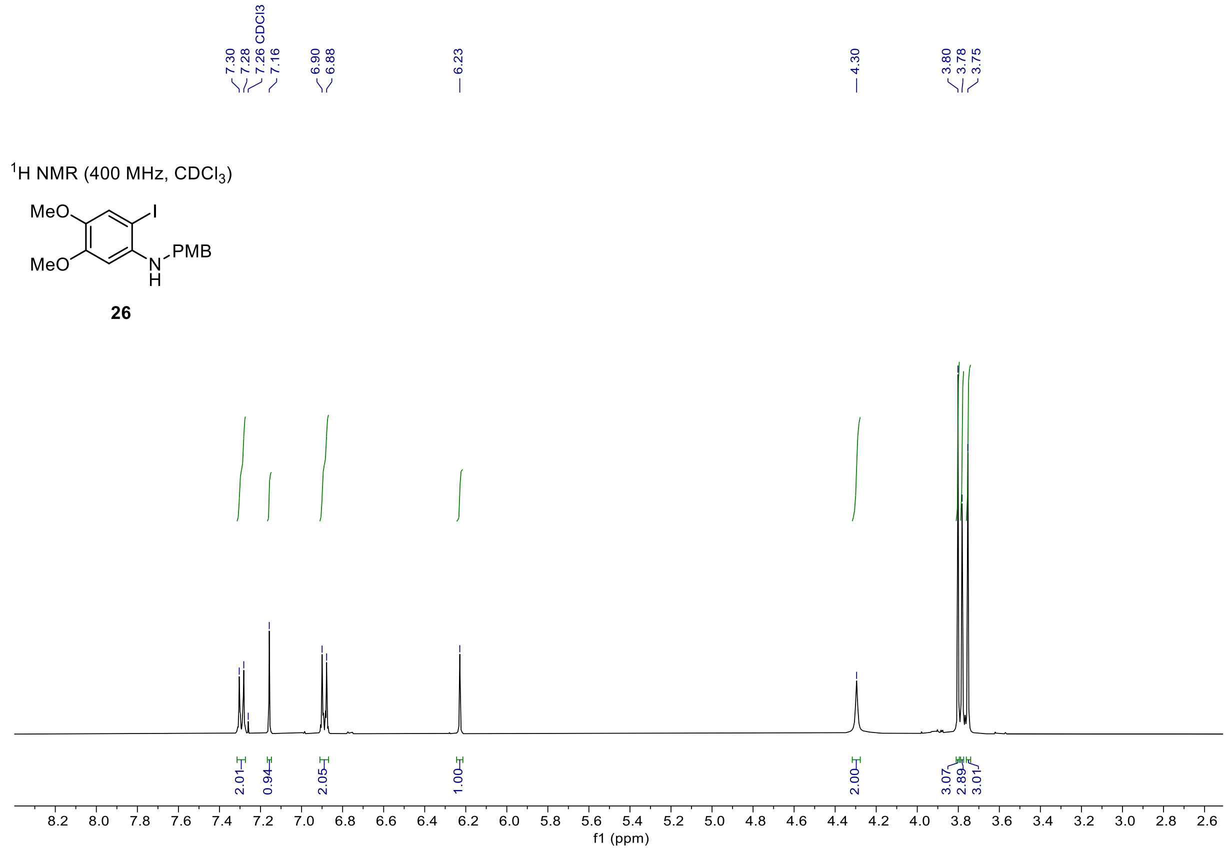

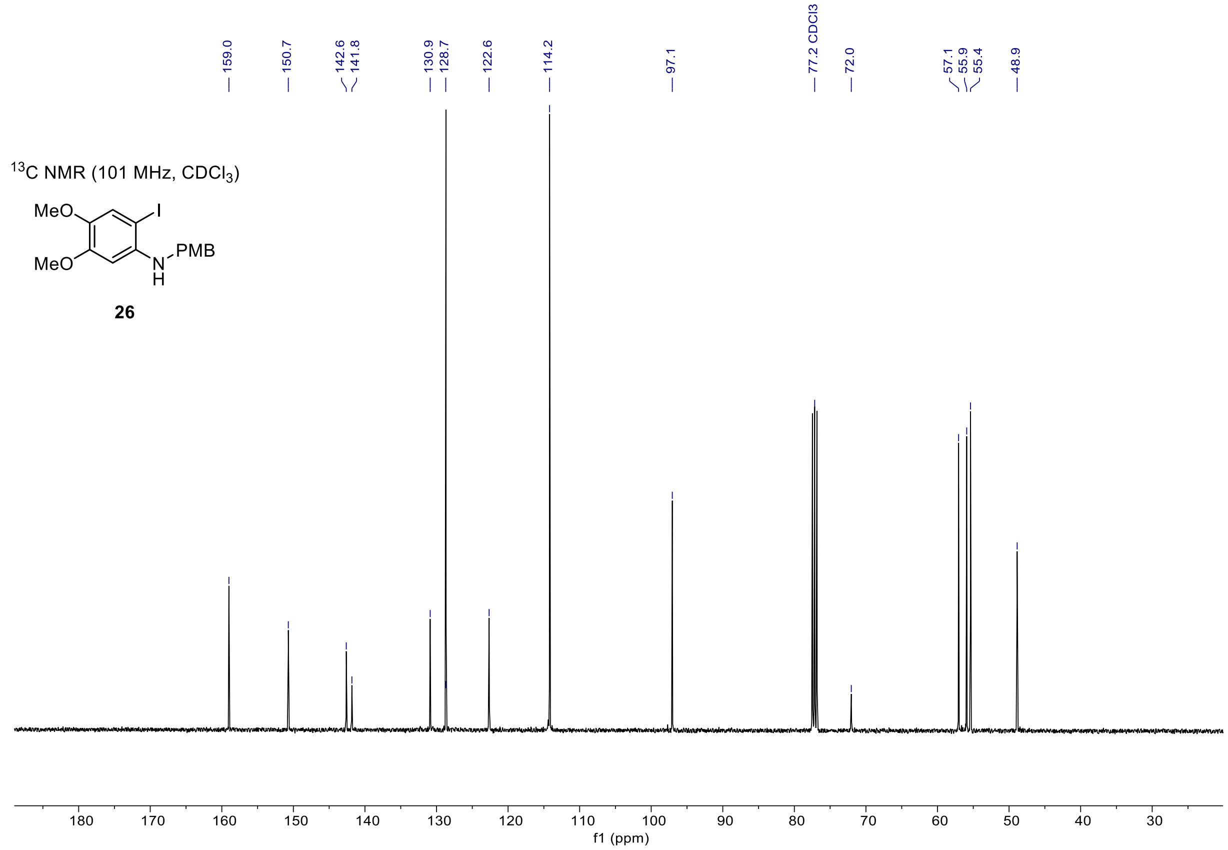

tert-Butyl (4-(trimethylsilyl)but-3-yn-1-yl)carbamate, **27** (Spectra in $CDCl_3$)

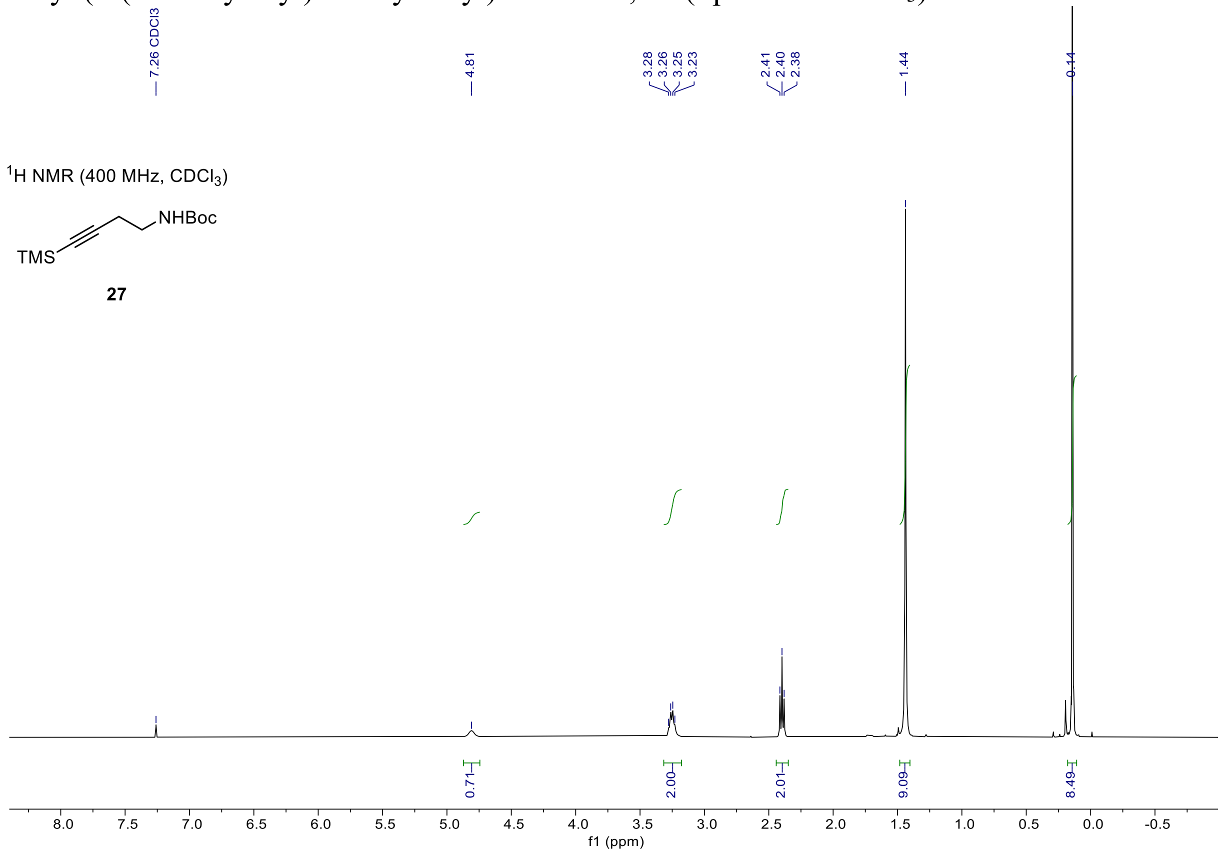

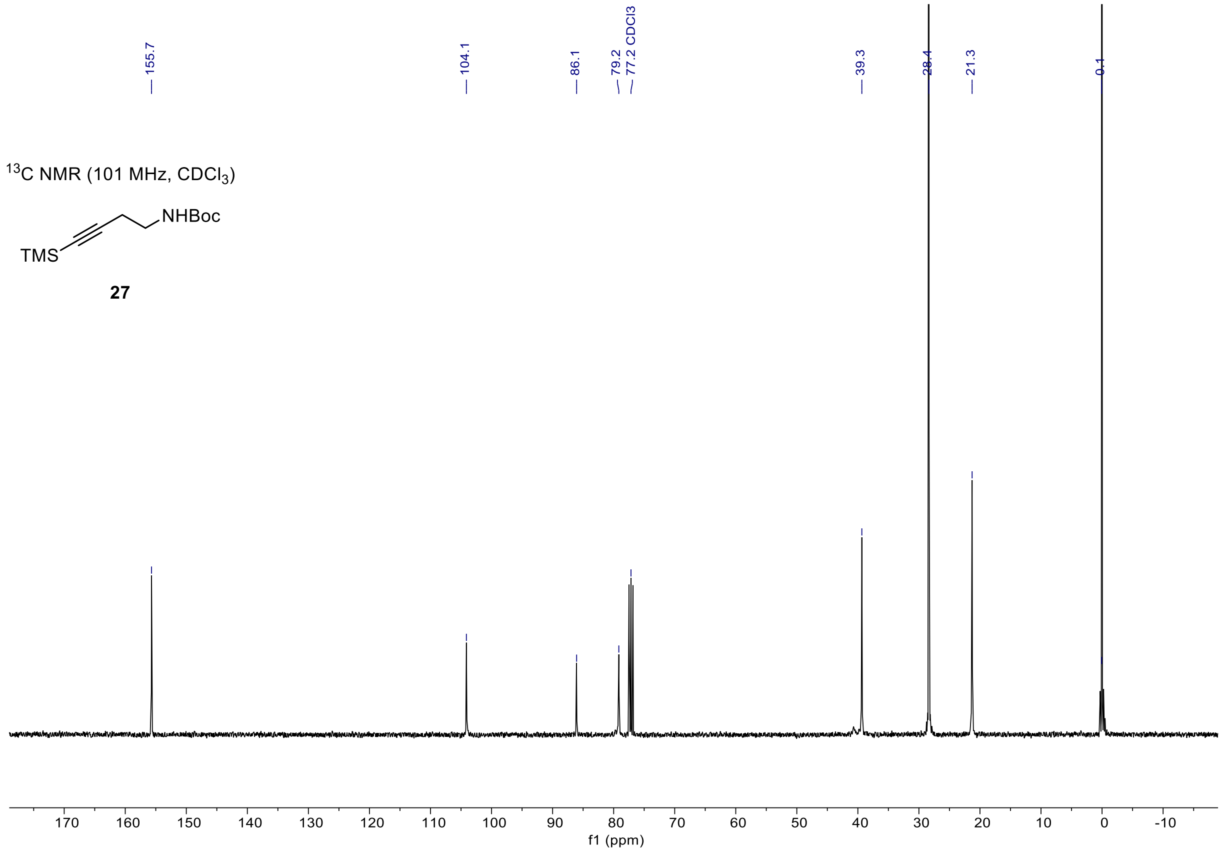

2-(5,6-Dimethoxy-1-(4-methoxybenzyl)-1*H*-indol-3-yl)ethan-1-amine, **25** (Spectra in $CDCl_3$)

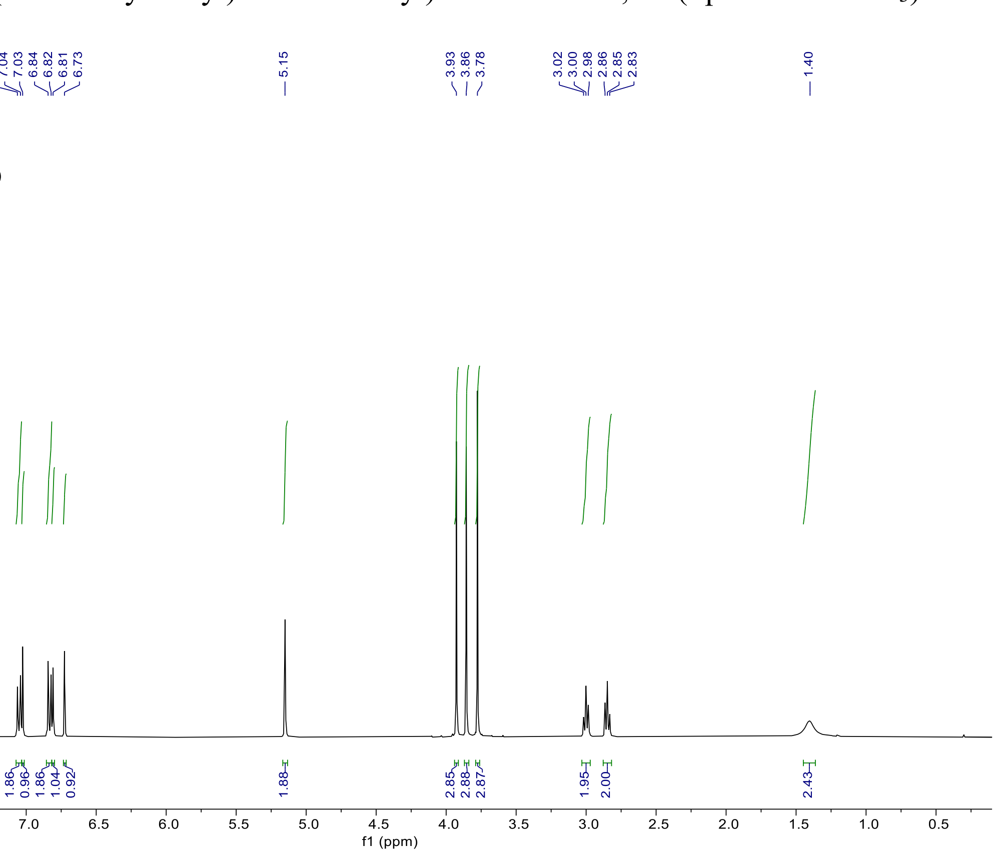

^{1}H NMR (400 MHz, $CDCl_3$)

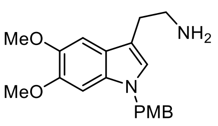

25

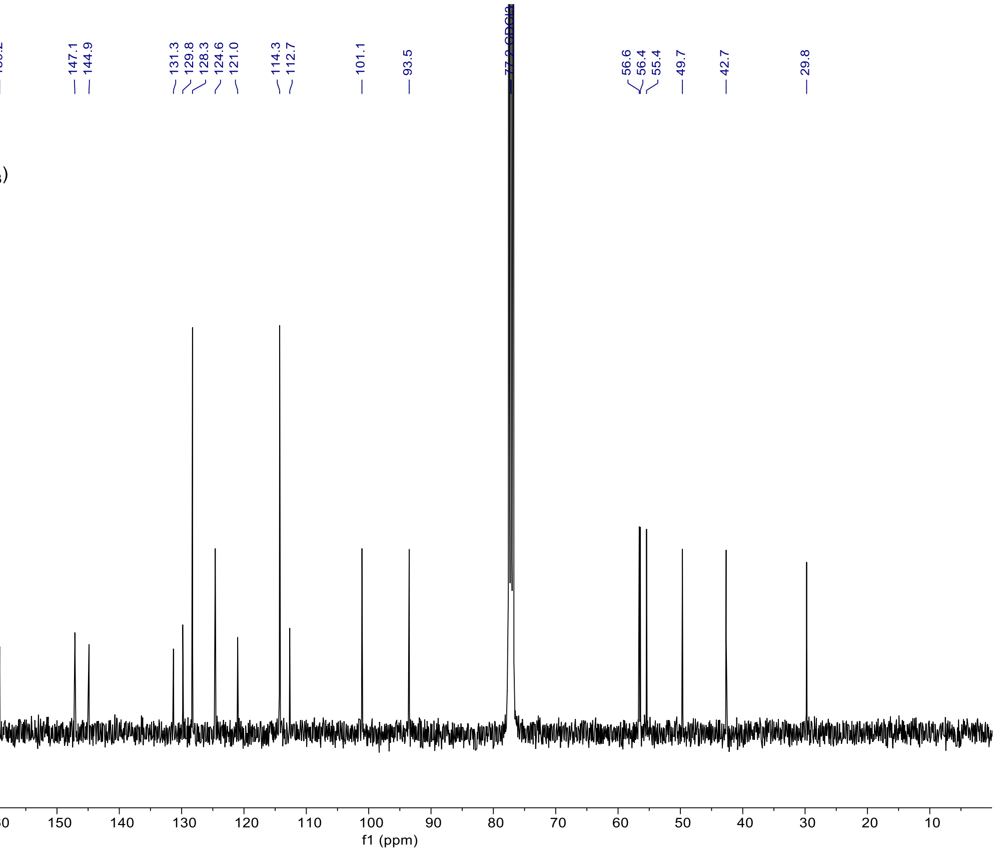

^{13}C NMR (101 MHz, $CDCl_3$)

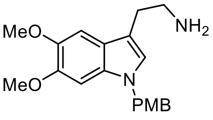

25

(*Z*)-4-((2-(5,6-Dimethoxy-1-(4-methoxybenzyl)-1*H*-indol-3-yl)ethyl)amino)-3-iodobut-2-en-1-yl benzoate, **S14** (Spectra in $CDCl_3$)

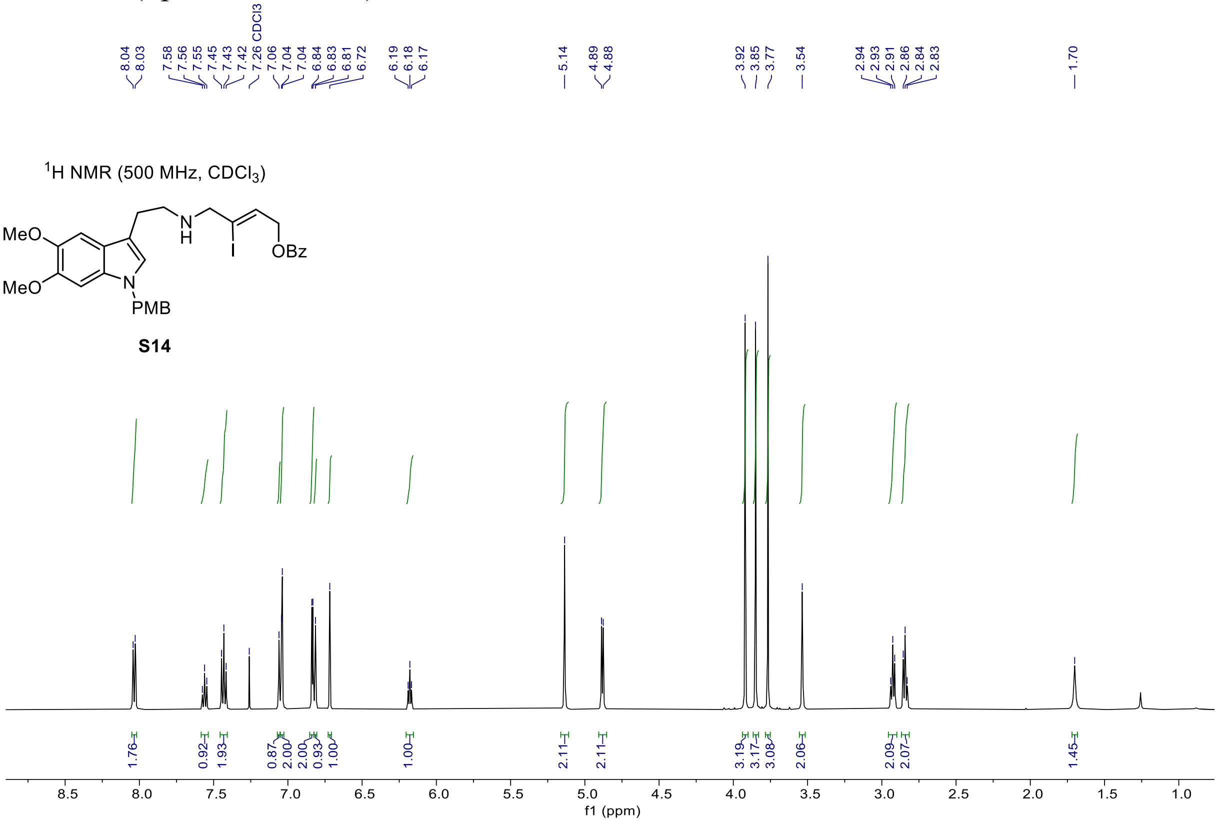

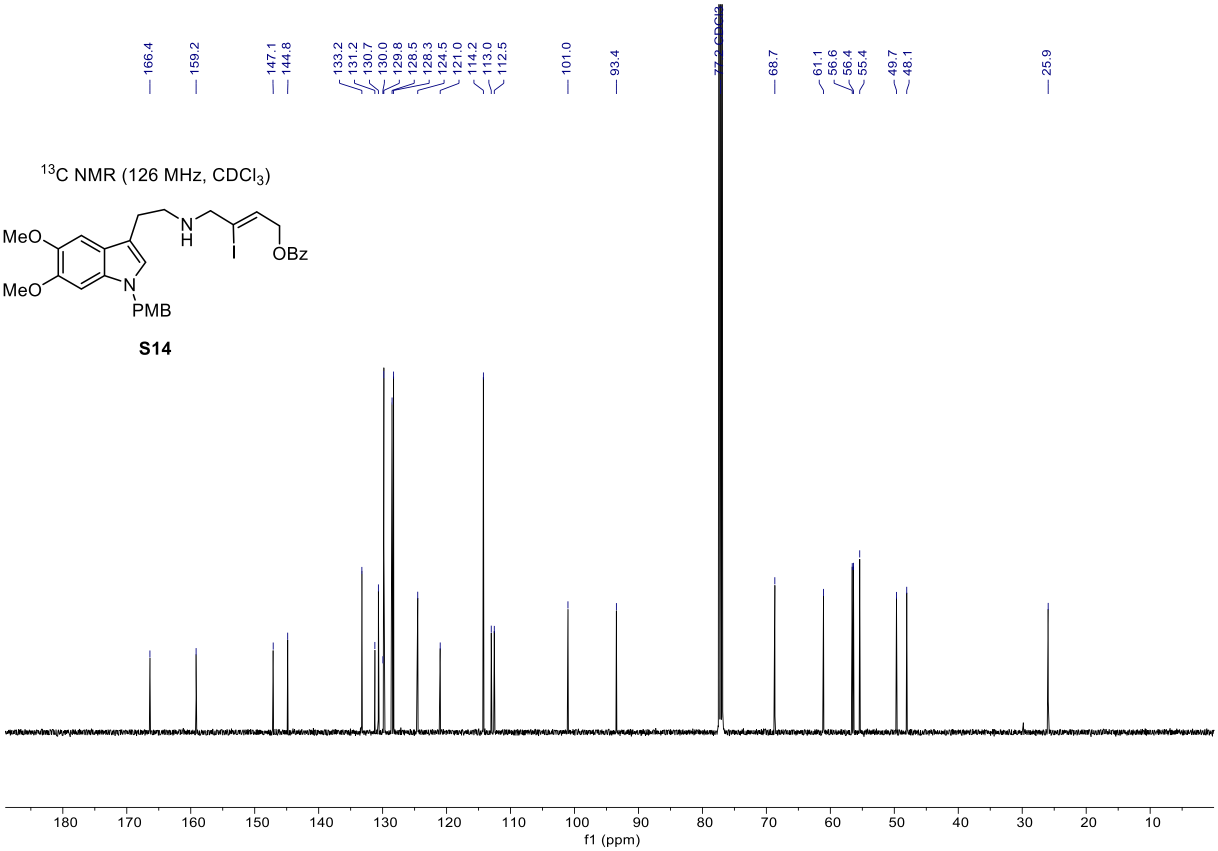

(*Z*)-4-((3a*S*,6a*R*,11b*R*)-6-((3a*S*,6*R*,7a*R*)-8,8-Dimethyl-2,2-dioxidohexahydro-3*H*-3a,6-methanobenzo[*c*]isothiazole-1-carbonyl)-9,10-dimethoxy-7-(4-methoxybenzyl)-1,2,3a,4,6a,7-hexahydro-3*H*-pyrrolo[2,3-*d*]carbazol-3-yl)-3-iodobut-2-en-1-yl benzoate, **28** (Spectra in $CDCl_3$)

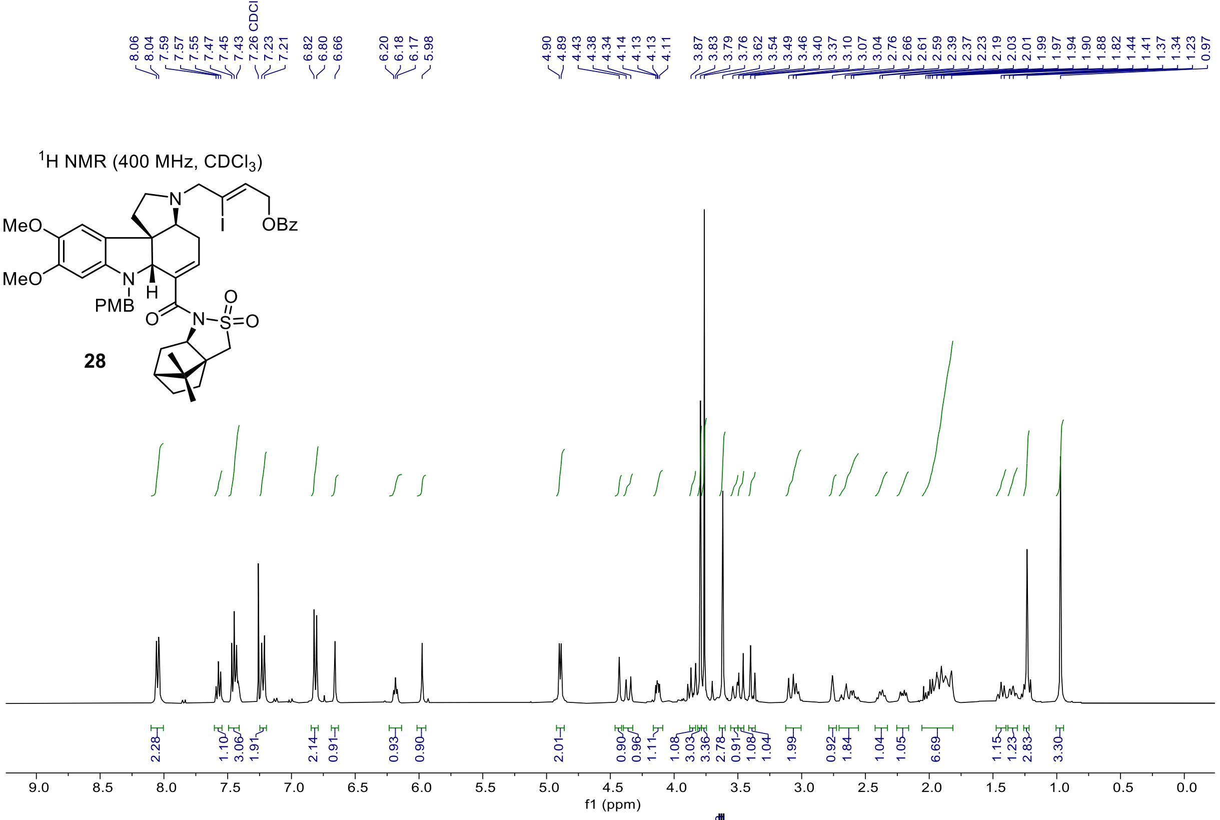

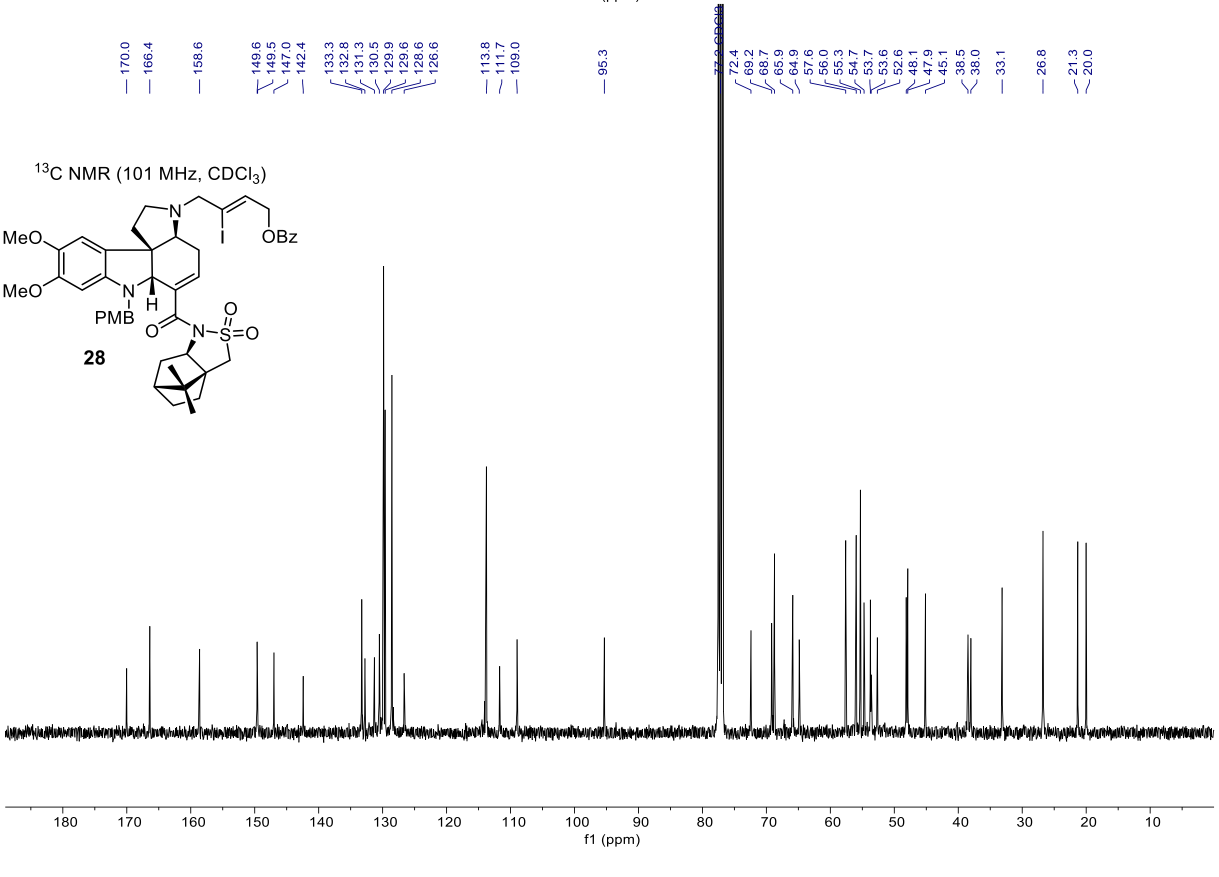

(*Z*)-4-((3a*R*,6a*R*,11b*R*)-6-((3a*S*,6*R*,7a*R*)-8,8-Dimethyl-2,2-dioxidohexahydro-3*H*-3a,6-methanobenzo[*c*]isothiazole-1-carbonyl)-9,10-dimethoxy-7-(4-methoxybenzyl)-1,2,3a,4,6a,7-hexahydro-3*H*-pyrrolo[2,3-*d*]carbazol-3-yl)-3-iodobut-2-en-1-yl benzoate, **28'** (Spectra in $CDCl_3$)

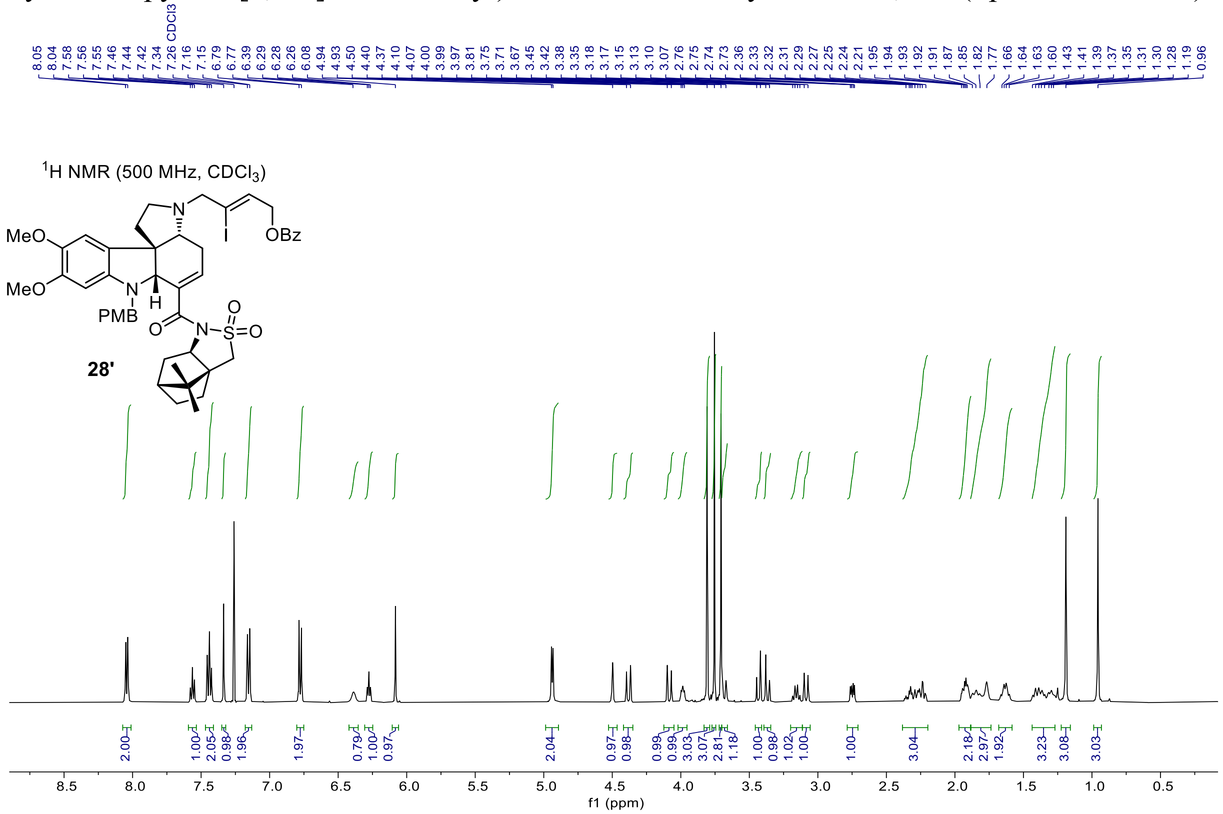

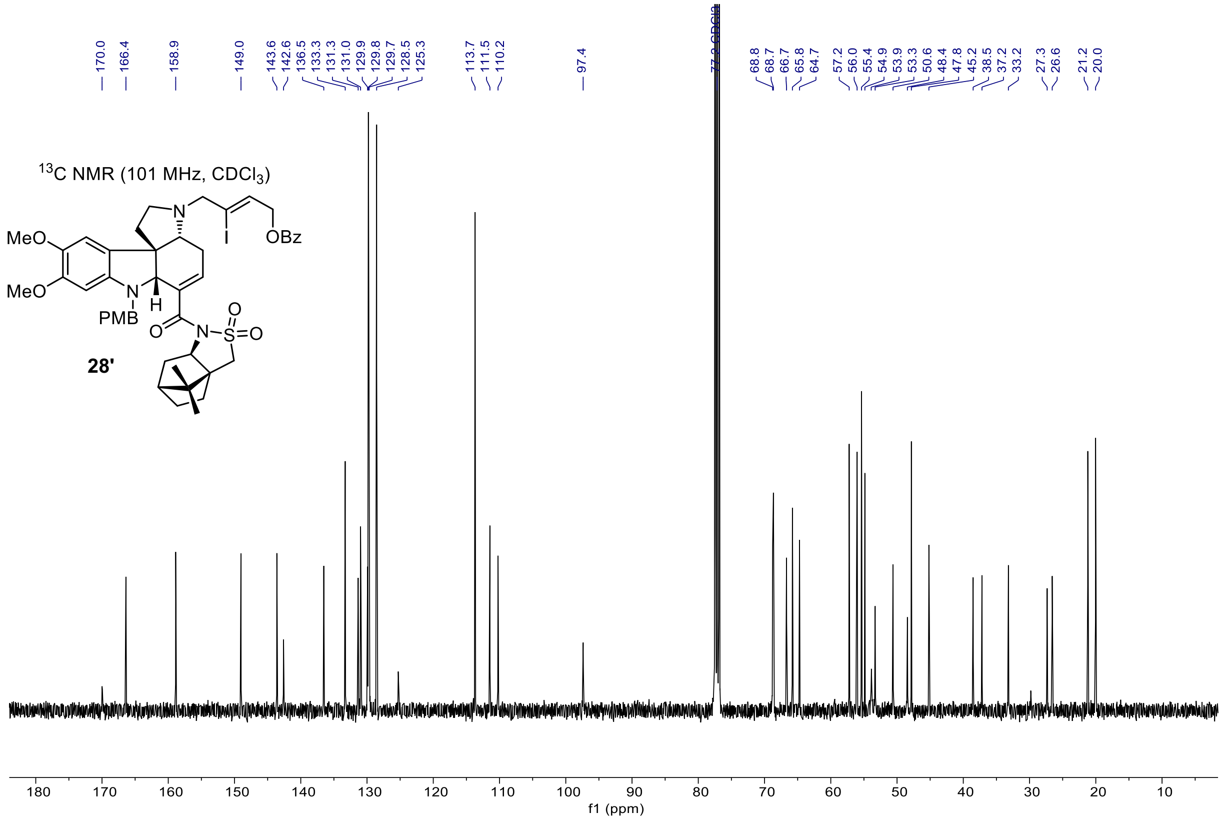

(*Z*)-4-((3a*S*,6a*R*,11b*R*)-6-(Hydroxymethyl)-7-(4-methoxybenzyl)-1,2,3a,4,6a,7-hexahydro-3*H*-pyrrolo[2,3-*d*]carbazol-3-yl)-3-iodobut-2-en-1-ol, **S16** (Spectra in $CDCl_3$)

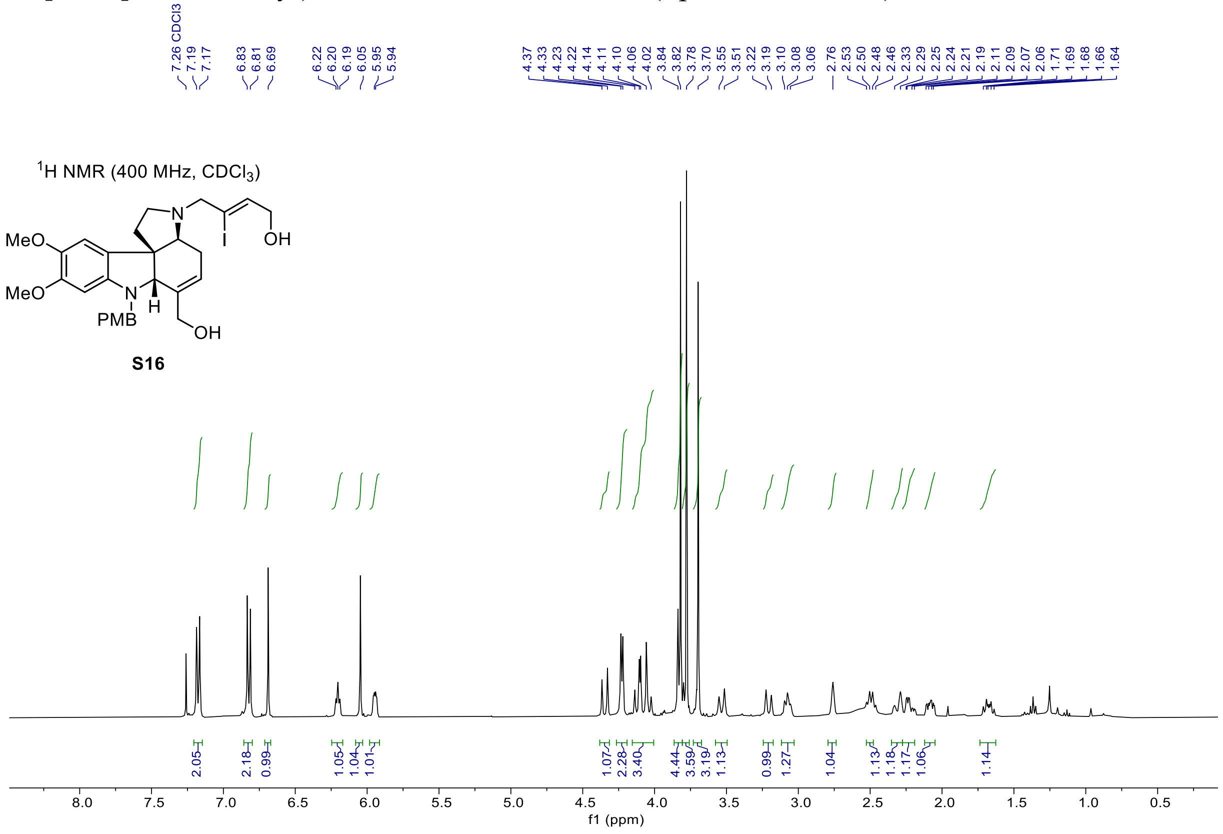

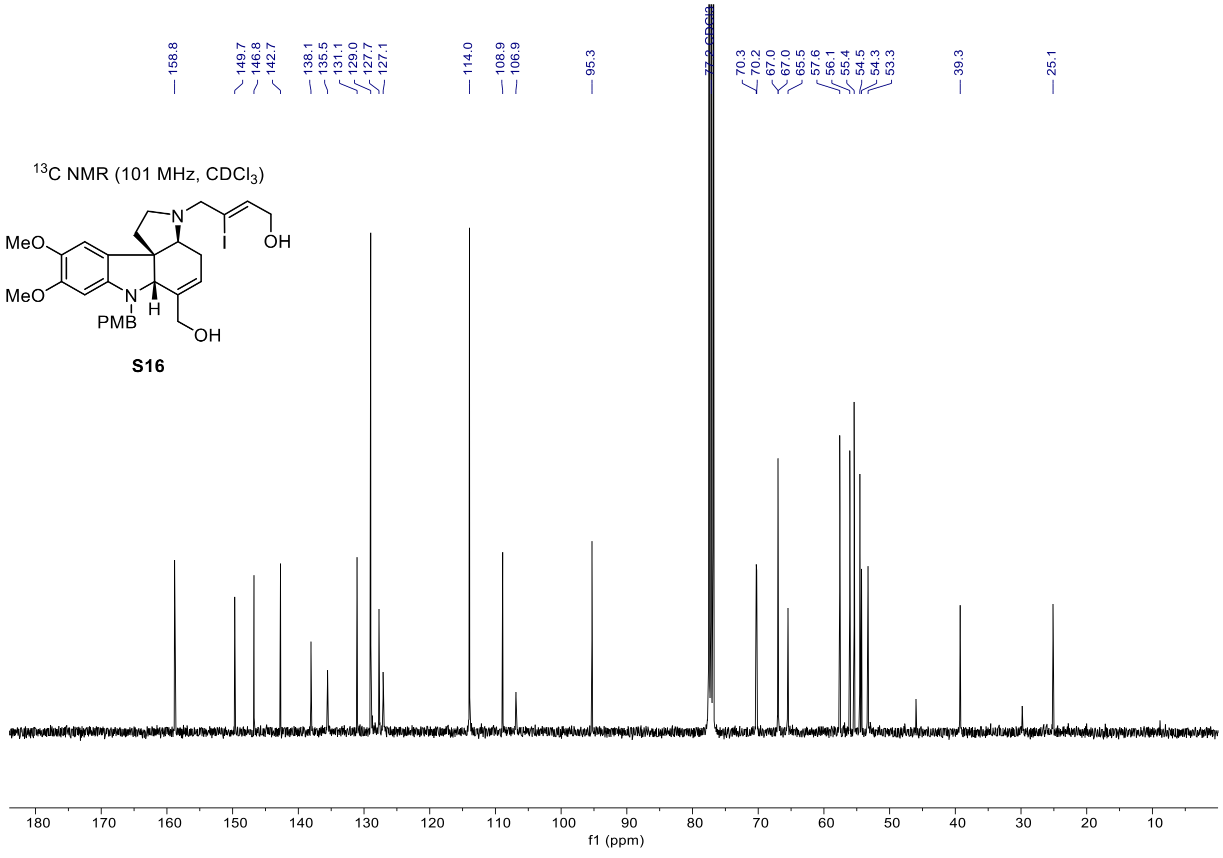

(4aR,*4*a1R,*5*aS,*8*aR,*8*a1S,*15*aS)-10,11-Dimethoxy-2,4a,4a1,5,5a,7,8,8a1,15,15a-decahydro-14H-*4*,6-methanoindolo[3,2,1-ij]*o*xepino[2,3,4-de]*p*yrrolo[2,3-h]*q*uinolin-14-one, **(–)-Brucine** (Spectra in $CDCl_3$)

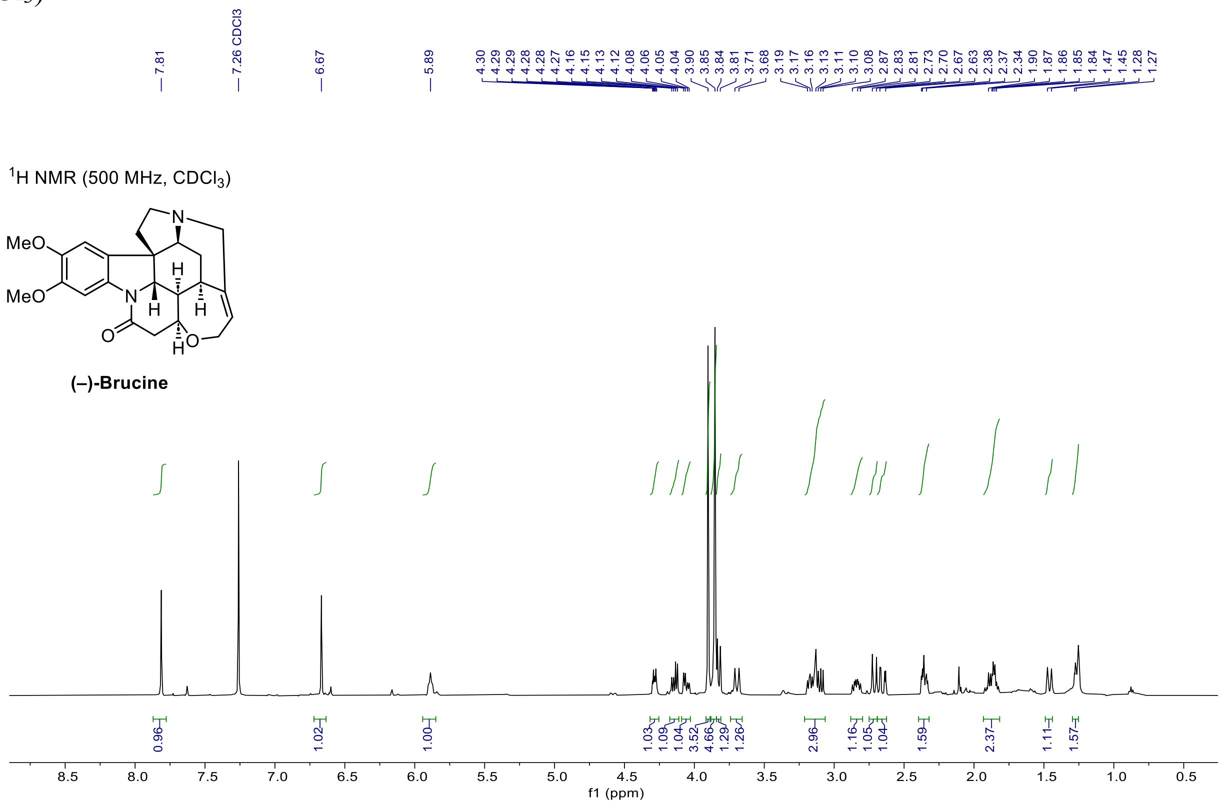

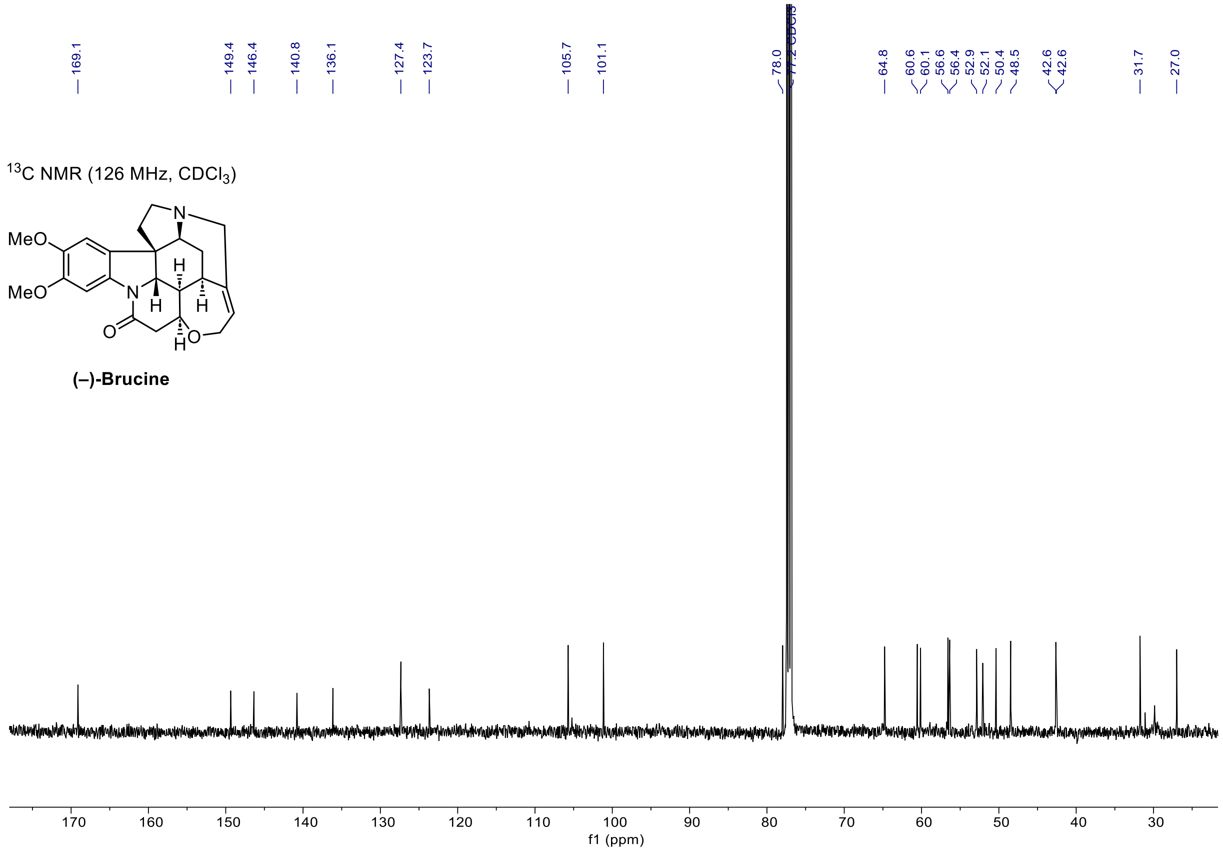

Methyl 5-((2-(1*H*-indol-3-yl)ethyl)(benzyl)amino)thiophene-2-carboxylate 1,1-dioxide, **S19** (Spectra in $CDCl_3$)

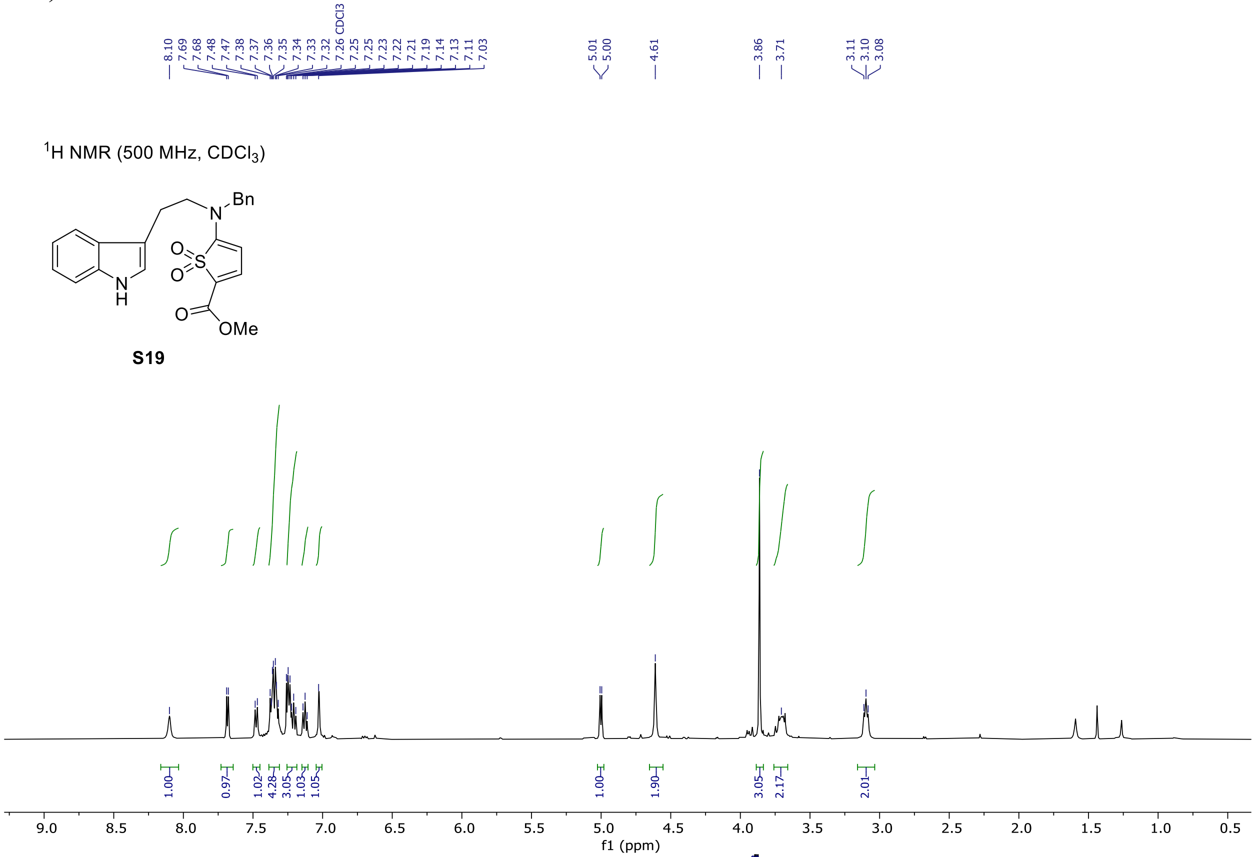

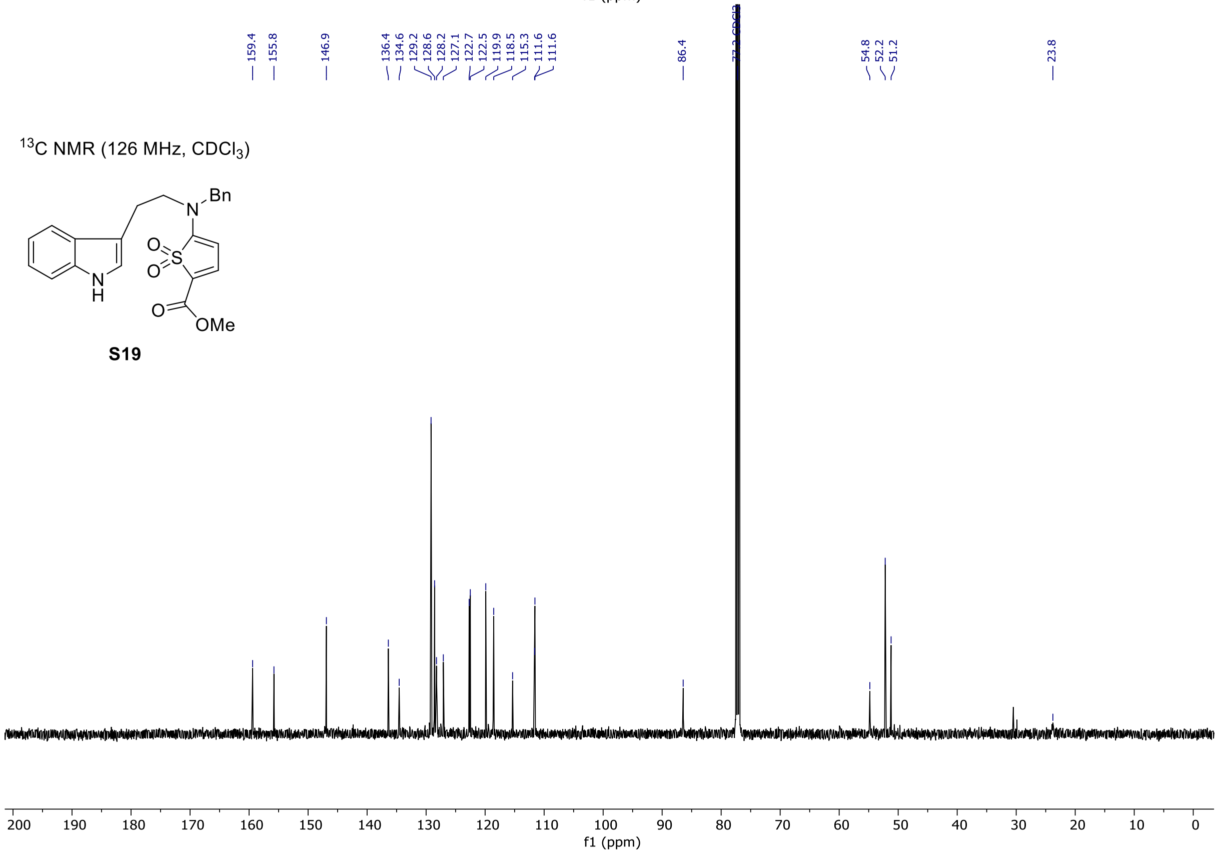

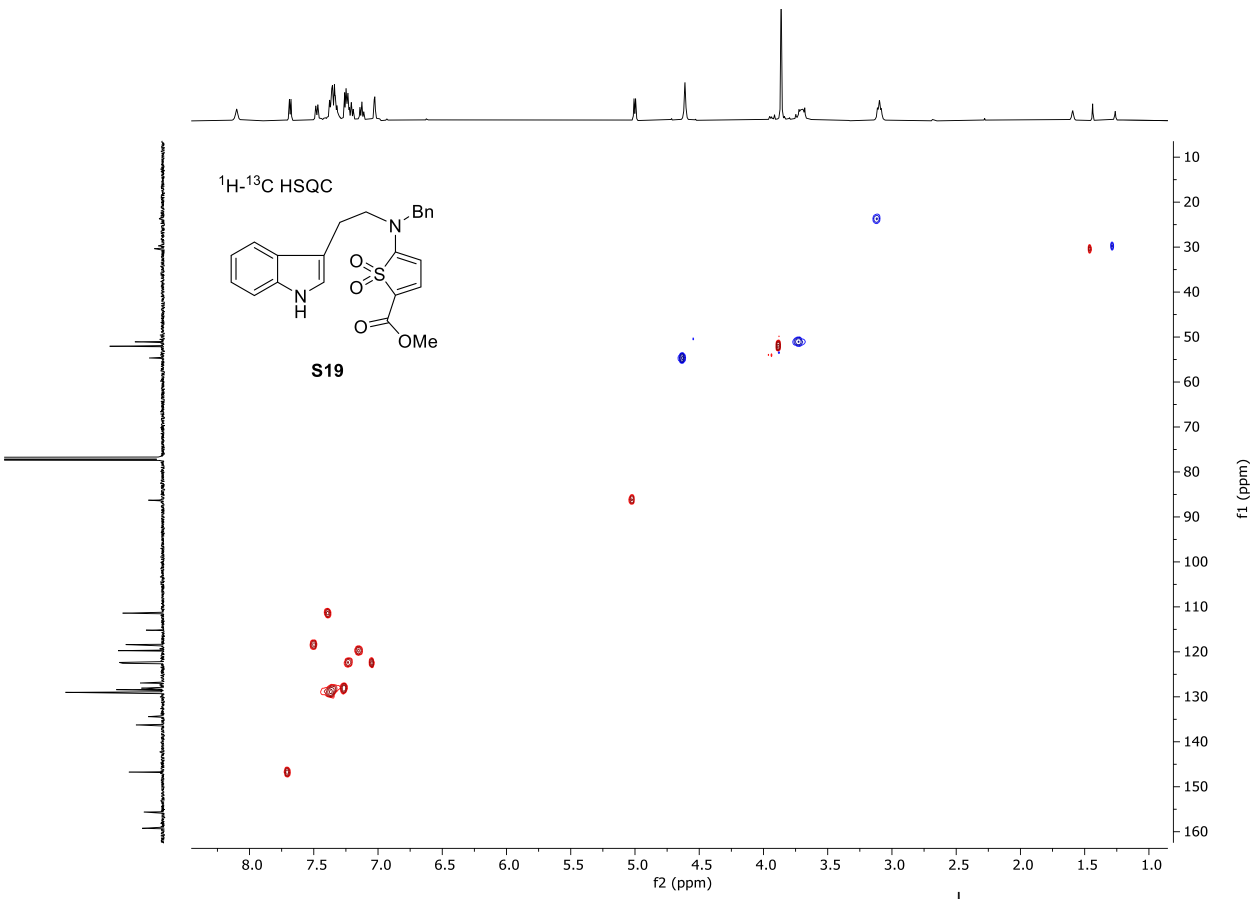
^{1}H-^{13}C HSQC
S19
f1 (ppm)
f2 (ppm)

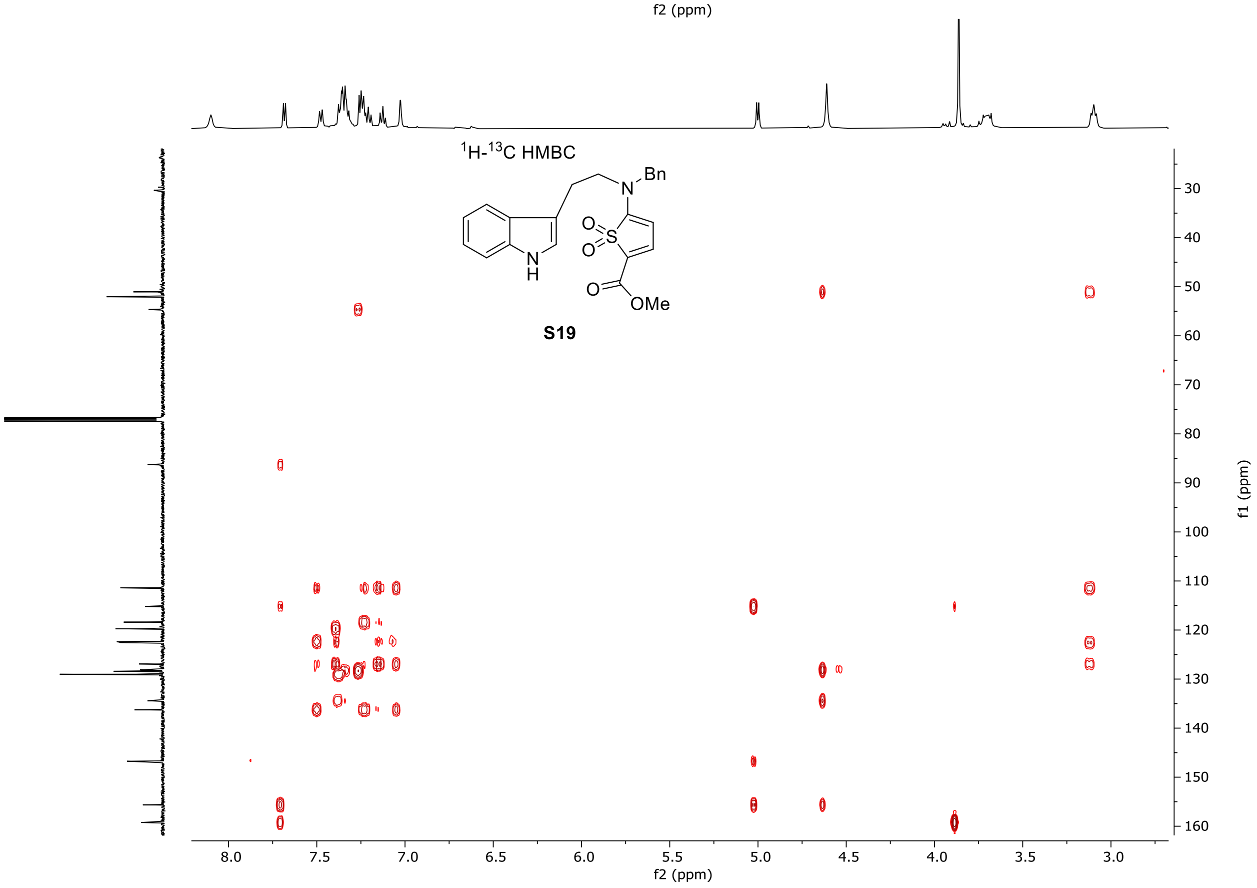
^{1}H-^{13}C HMBC
S19
f1 (ppm)
f2 (ppm)

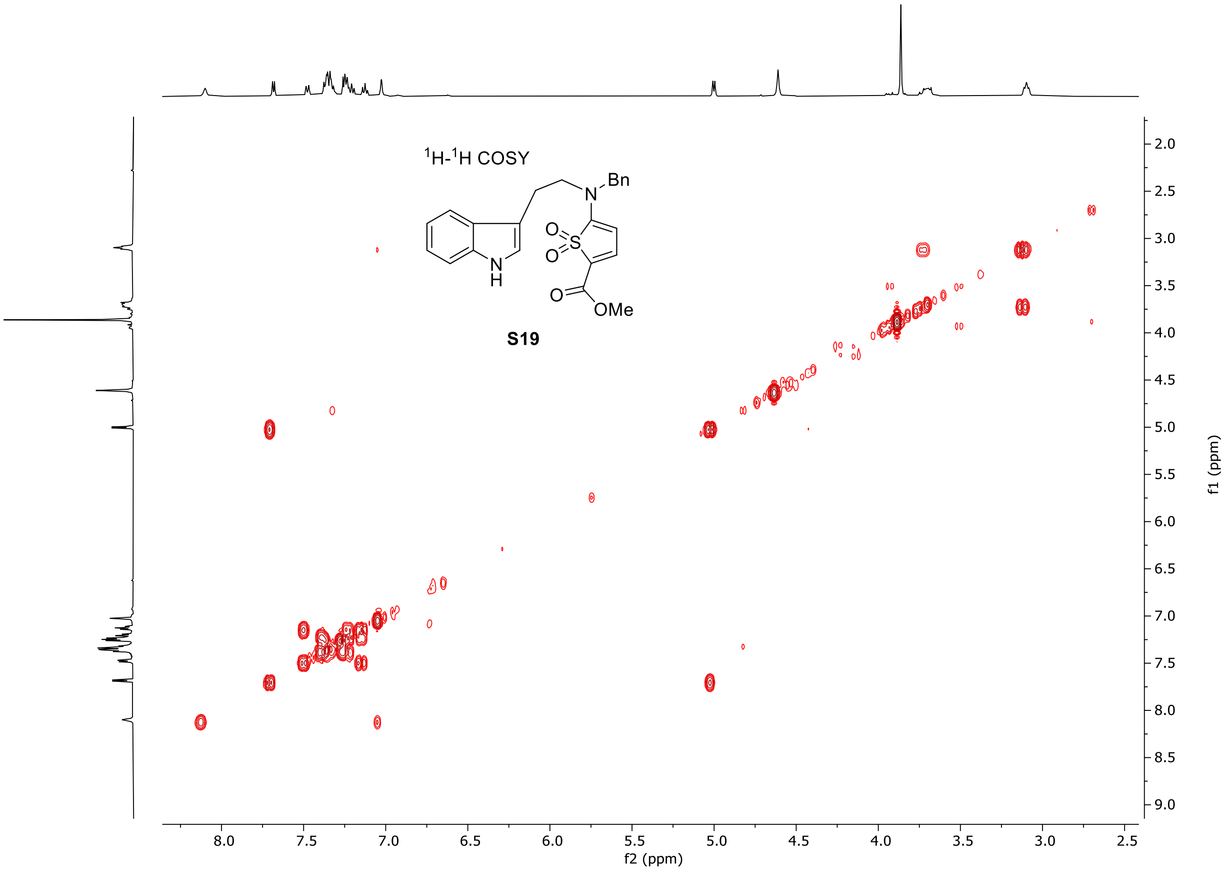

1H-1H COSY
S19
Bn
N
O
S
O
N
H
O
OMe
f1 (ppm)
f2 (ppm)

Methyl (6a*R*,11b*S*)-3-benzyl-2,3,6a,7-tetrahydro-1*H*-pyrrolo[2,3-*d*]carbazole-6-carboxylate, **S20** (Spectra in $CDCl_3$)

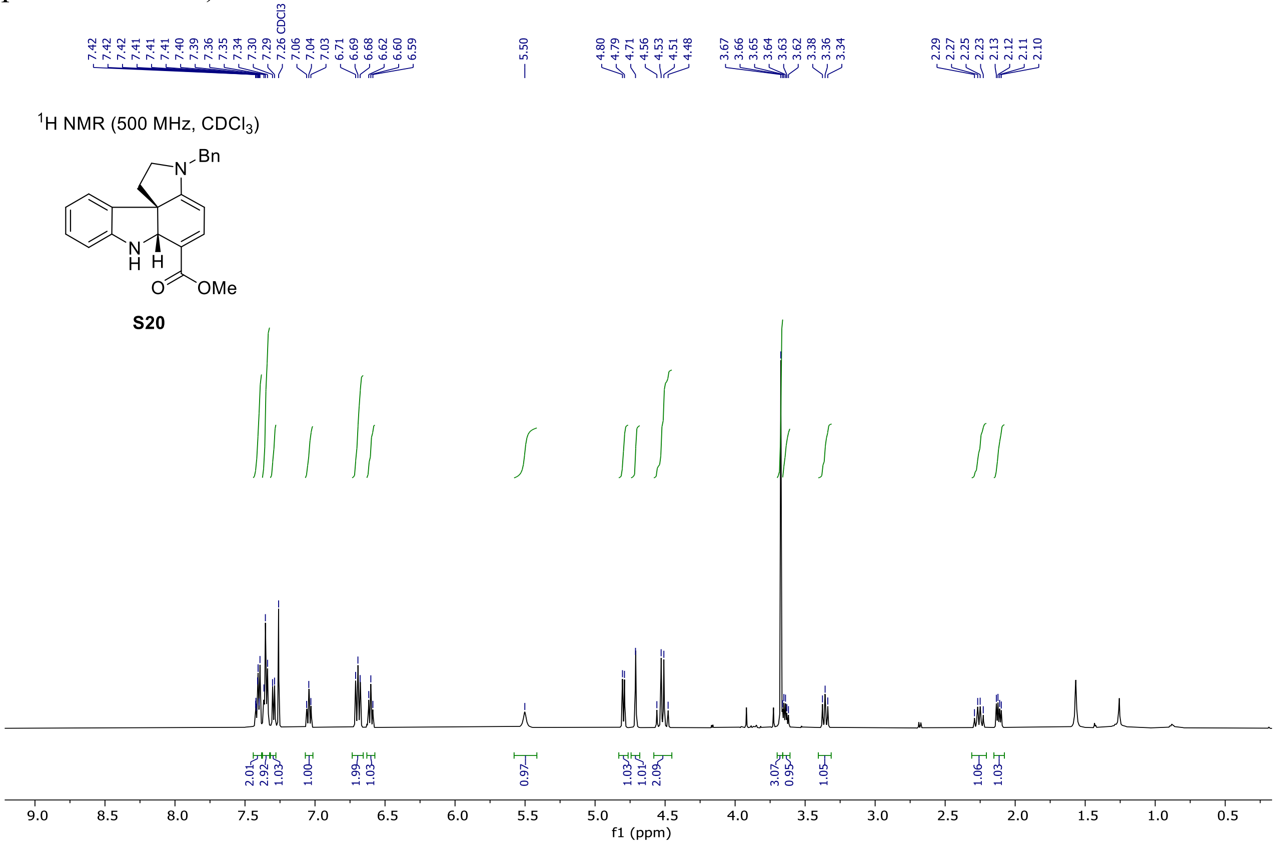

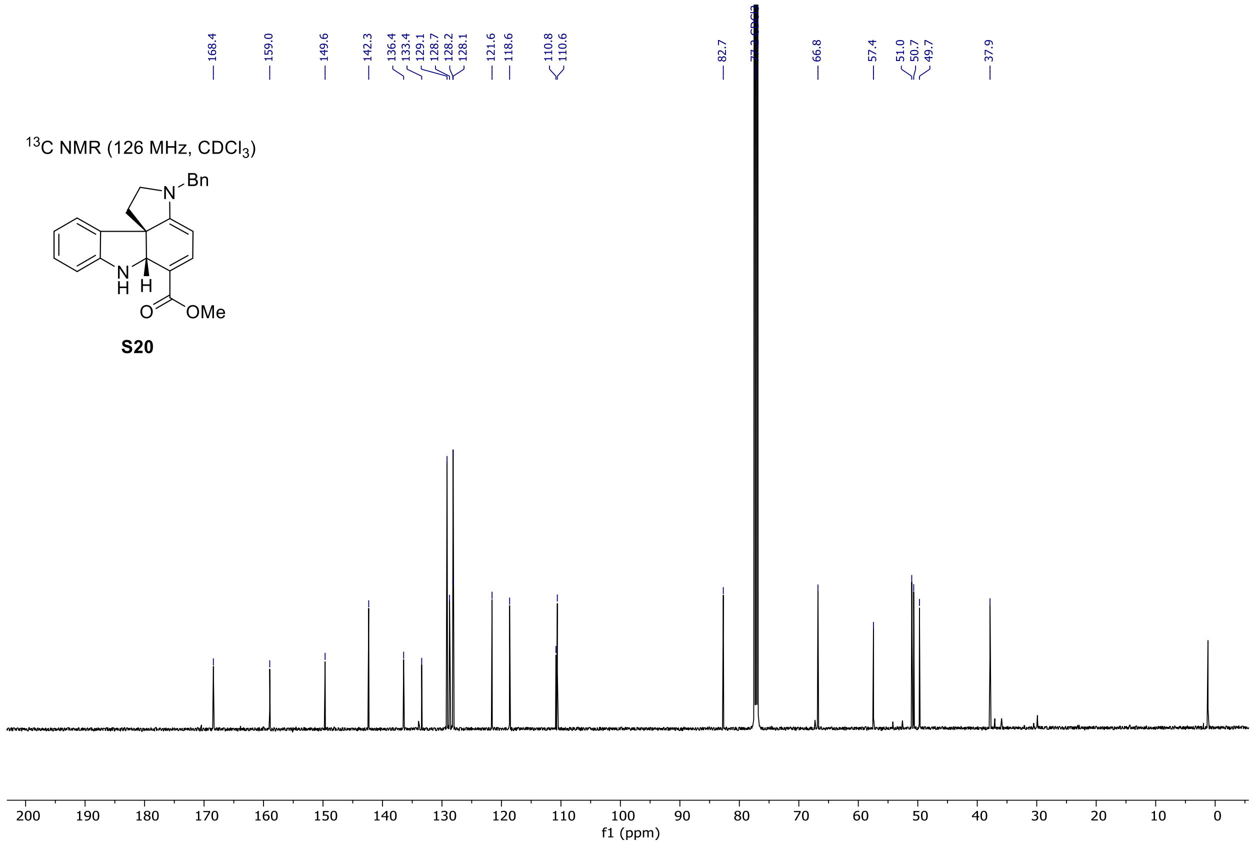

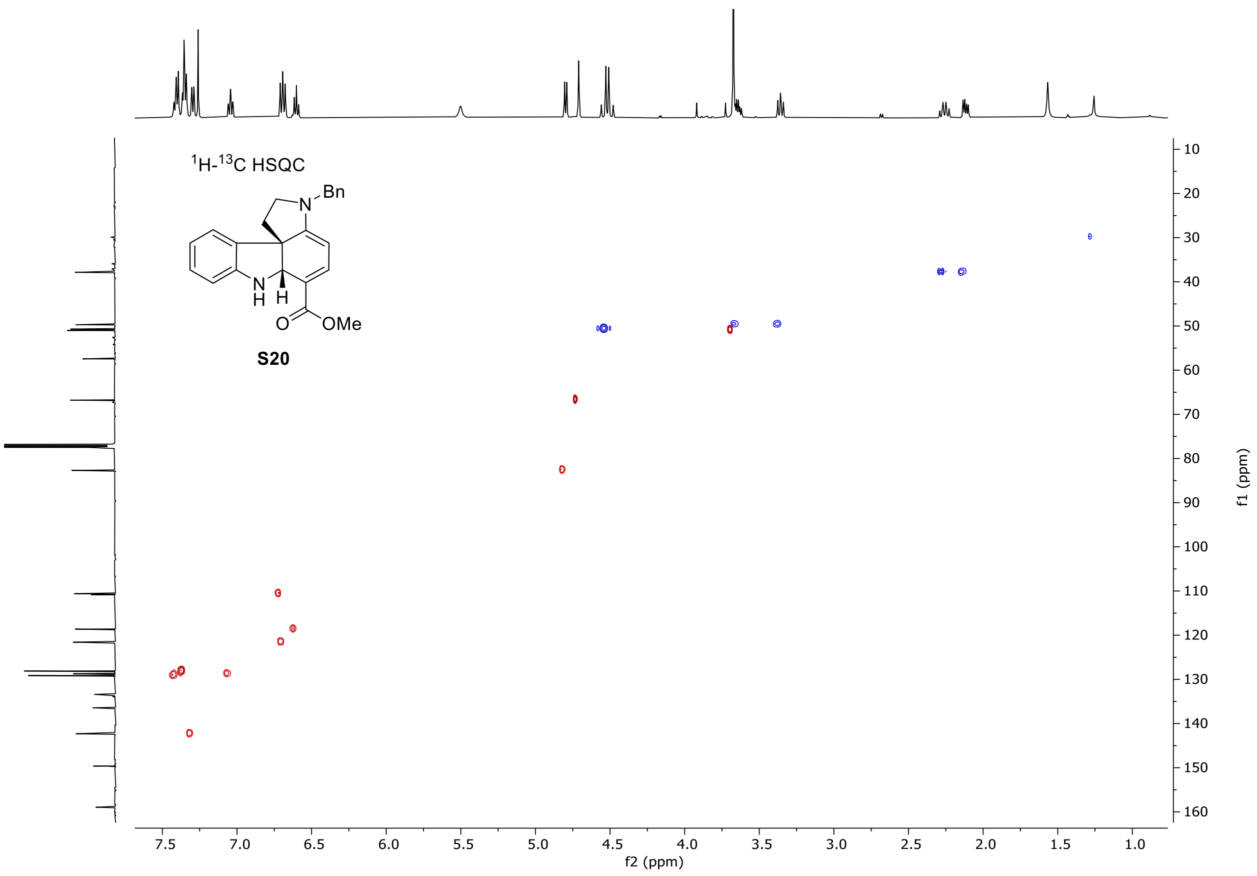

1H-13C HSQC
N
Bn
N
H
H
O
OMe
S20
f2 (ppm)
f1 (ppm)

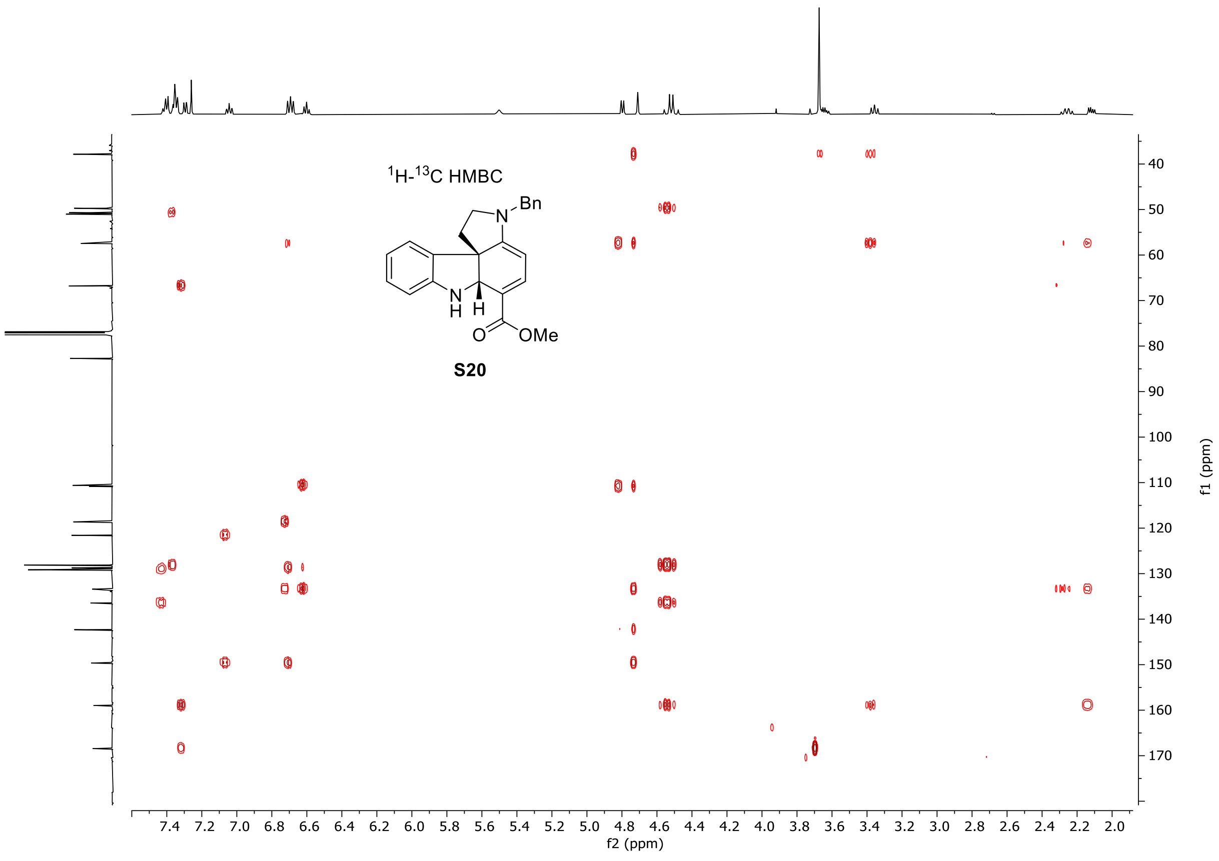

1H-13C HMBC
N
Bn
N
H
H
O
OMe
S20
f2 (ppm)
f1 (ppm)

tert-Butyl (*Z*)-(2-(1*H*-indol-3-yl)ethyl)(2-iodobut-2-en-1-yl)carbamate, **S21** (Spectra in $CDCl_3$)

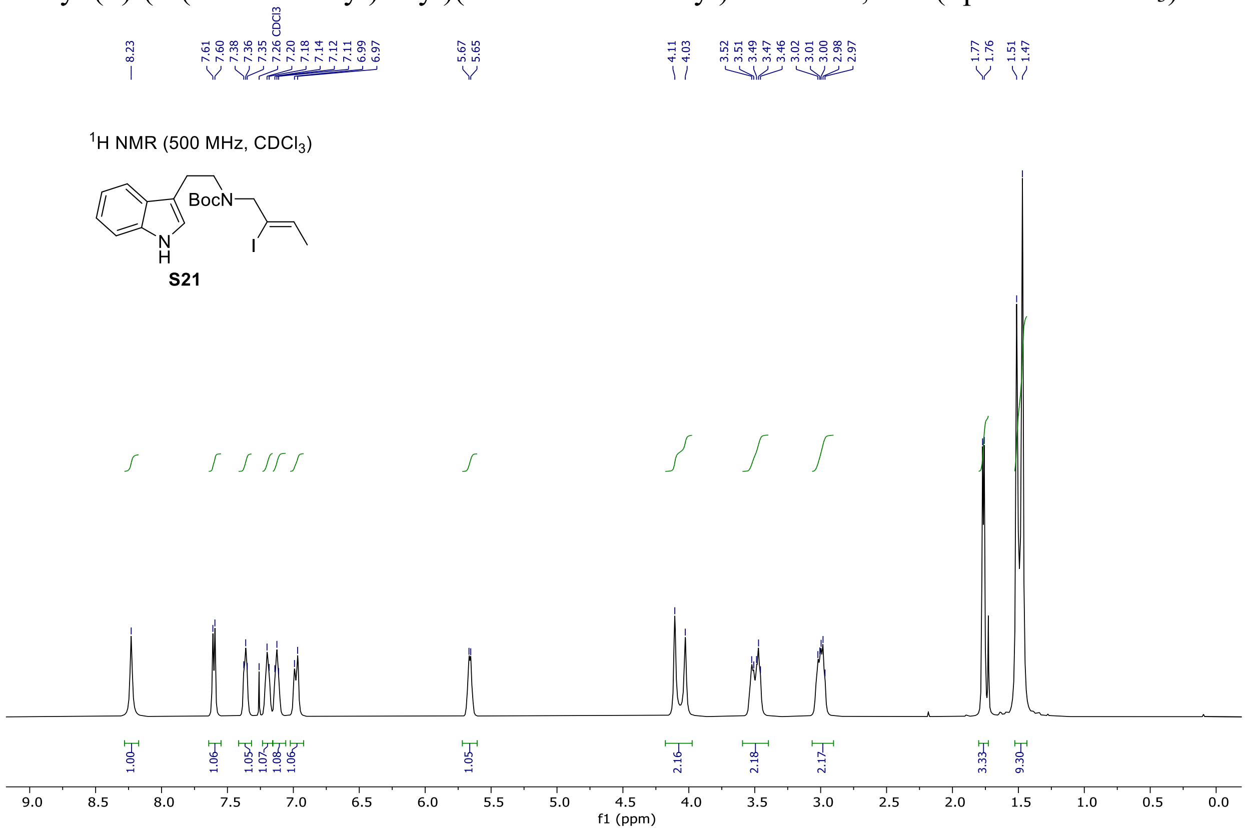

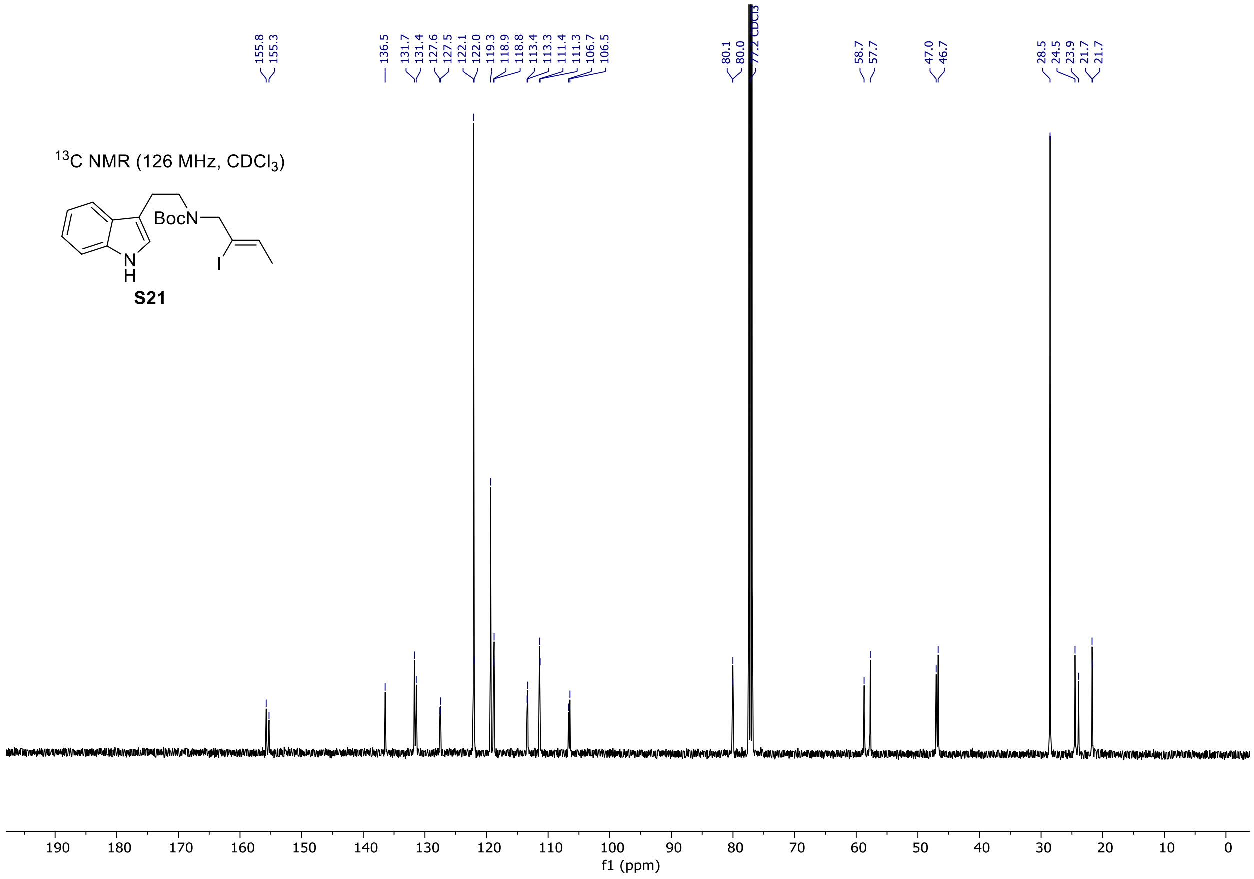

tert-Butyl (((4a*R*,9a*S*)-4-chloro-1-((3a*S*,6*R*,7a*R*)-8,8-dimethyl-2,2-dioxidohexahydro-3*H*-3a,6-methanobenzo[*c*]isothiazole-1-carbonyl)-9,9a-dihydro-4a*H*-carbazol-4a-yl)methyl)((*Z*)-2-iodobut-2-en-1-yl)carbamate, **S22** (Spectra in $CDCl_3$)

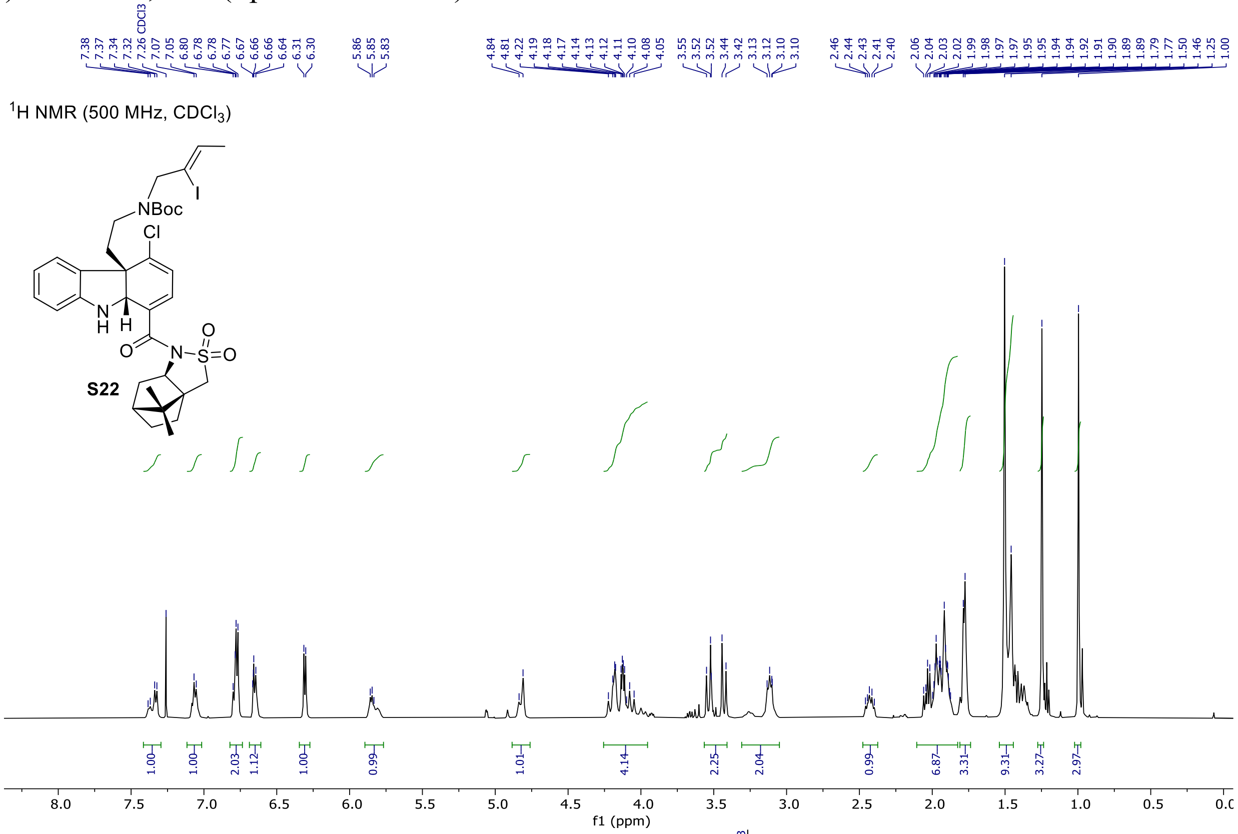

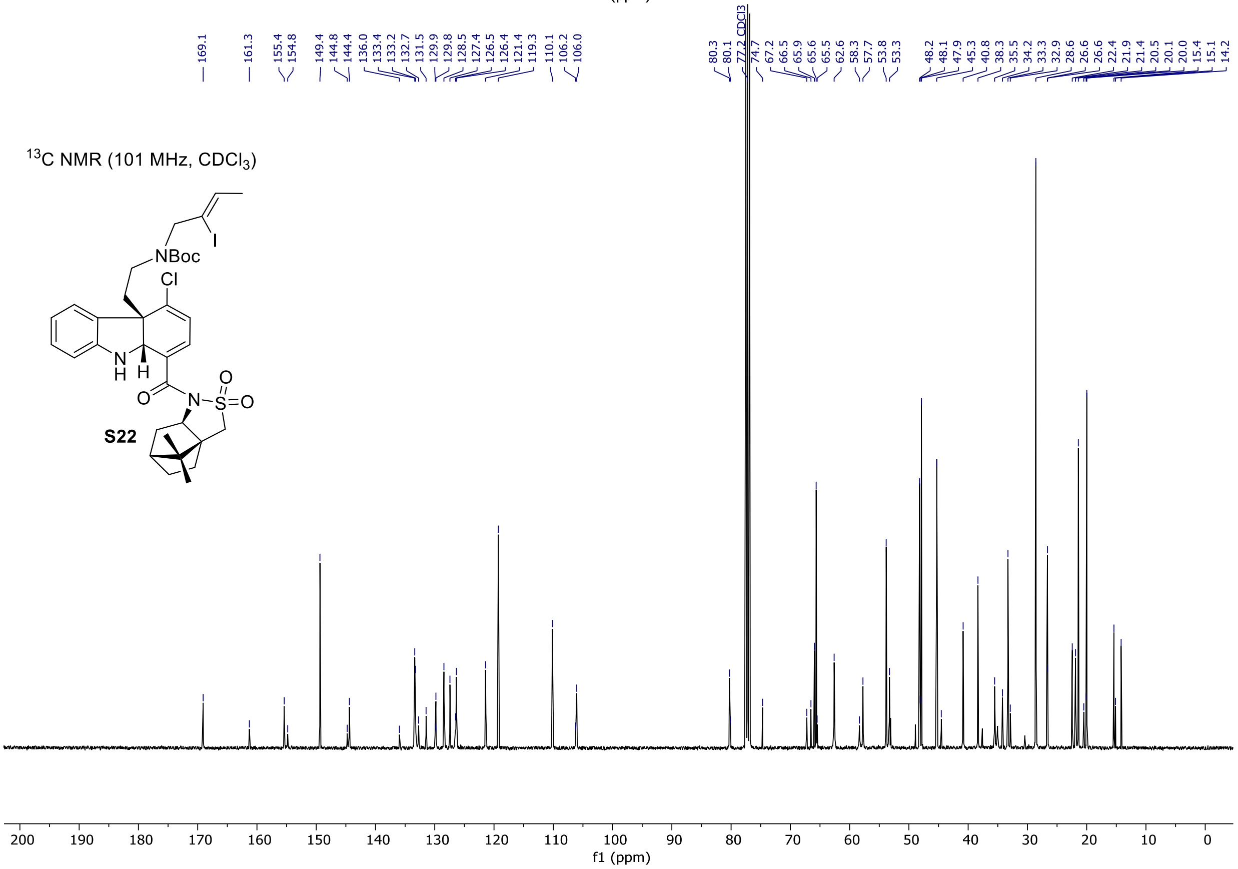

((3a*S*,6*R*,7a*R*)-8,8-Dimethyl-2,2-dioxidotetrahydro-3*H*-3a,6-methanobenzo[*c*]isothiazol-1(4*H*)-yl)((6a*R*,11b*S*)-3-((*Z*)-2-iodobut-2-en-1-yl)-2,3,6a,7-tetrahydro-1*H*-pyrrolo[2,3-*d*]carbazol-6-yl)methanone, **13** (Spectra in $CDCl_3$)

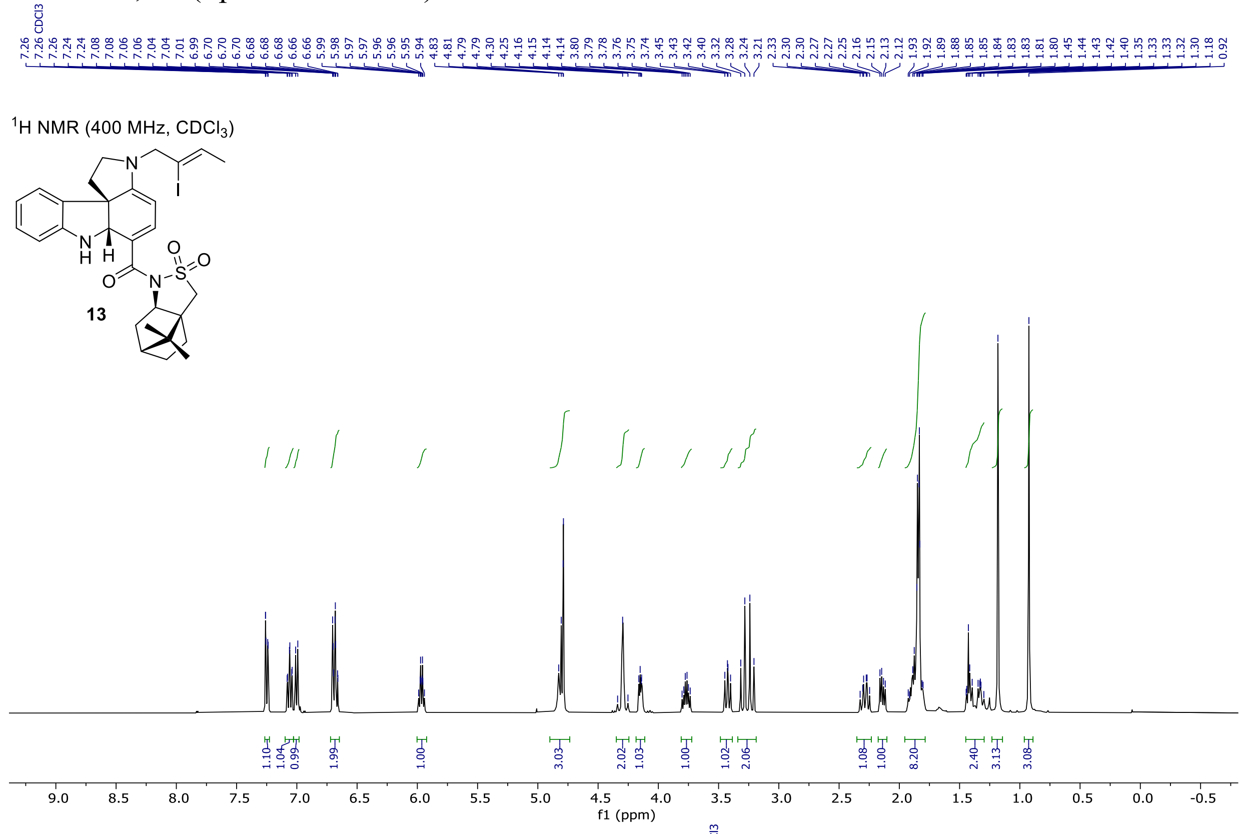

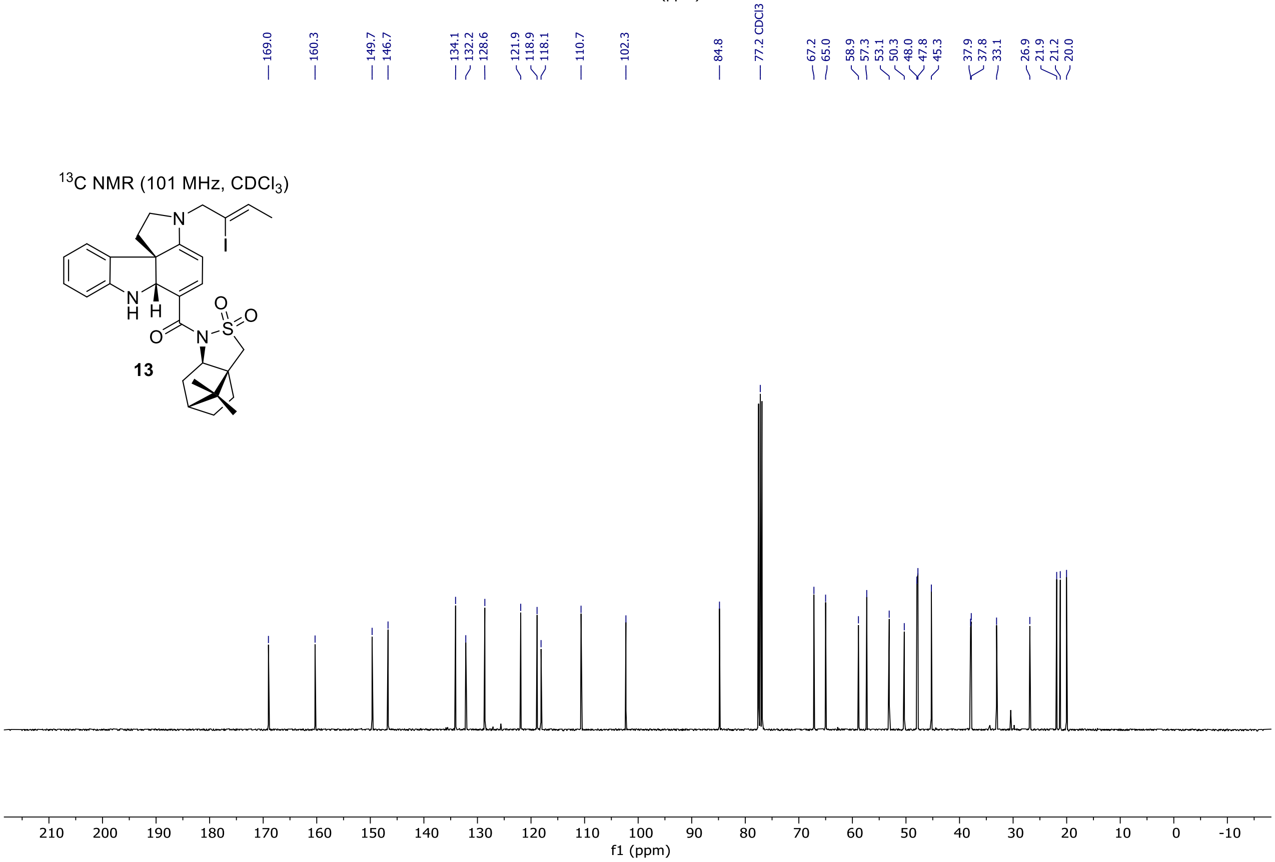

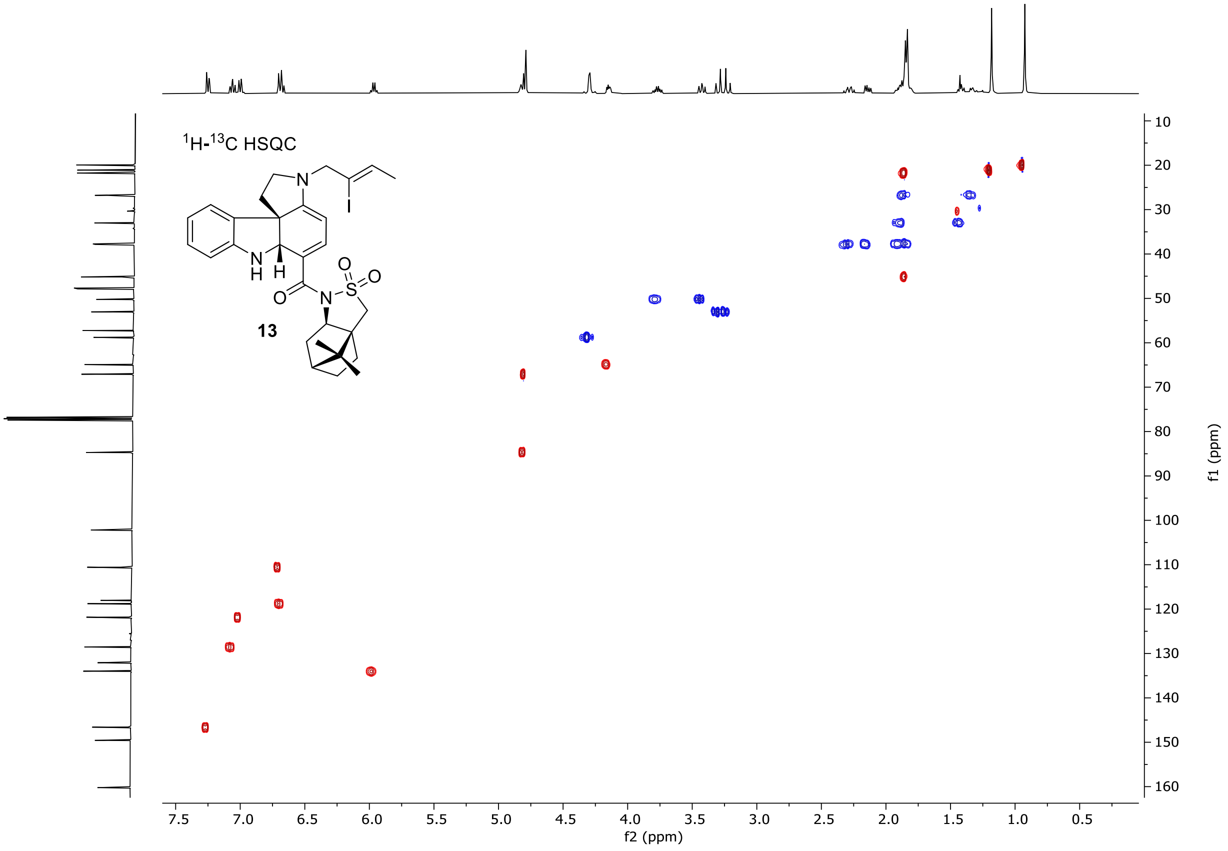

1H-13C HSQC
13
f1 (ppm)
f2 (ppm)

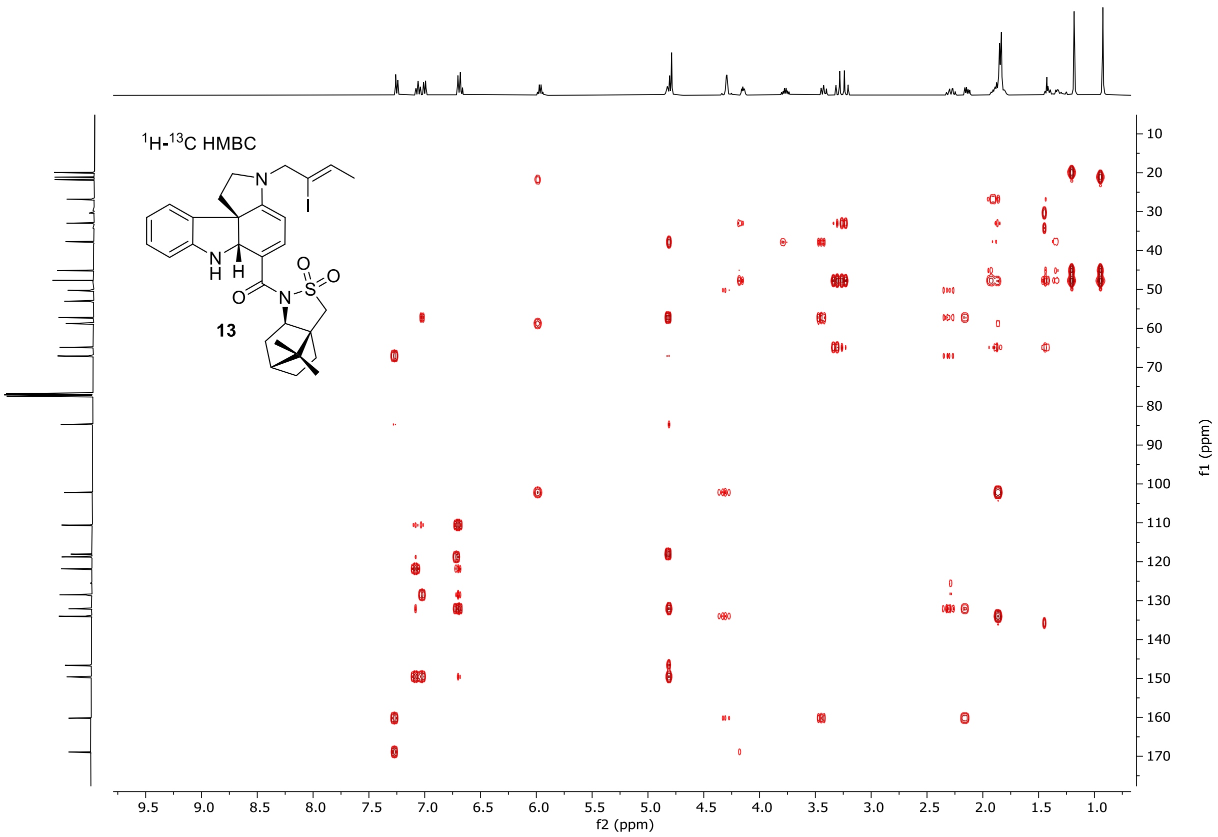

1H-13C HMBC
13
f1 (ppm)
f2 (ppm)

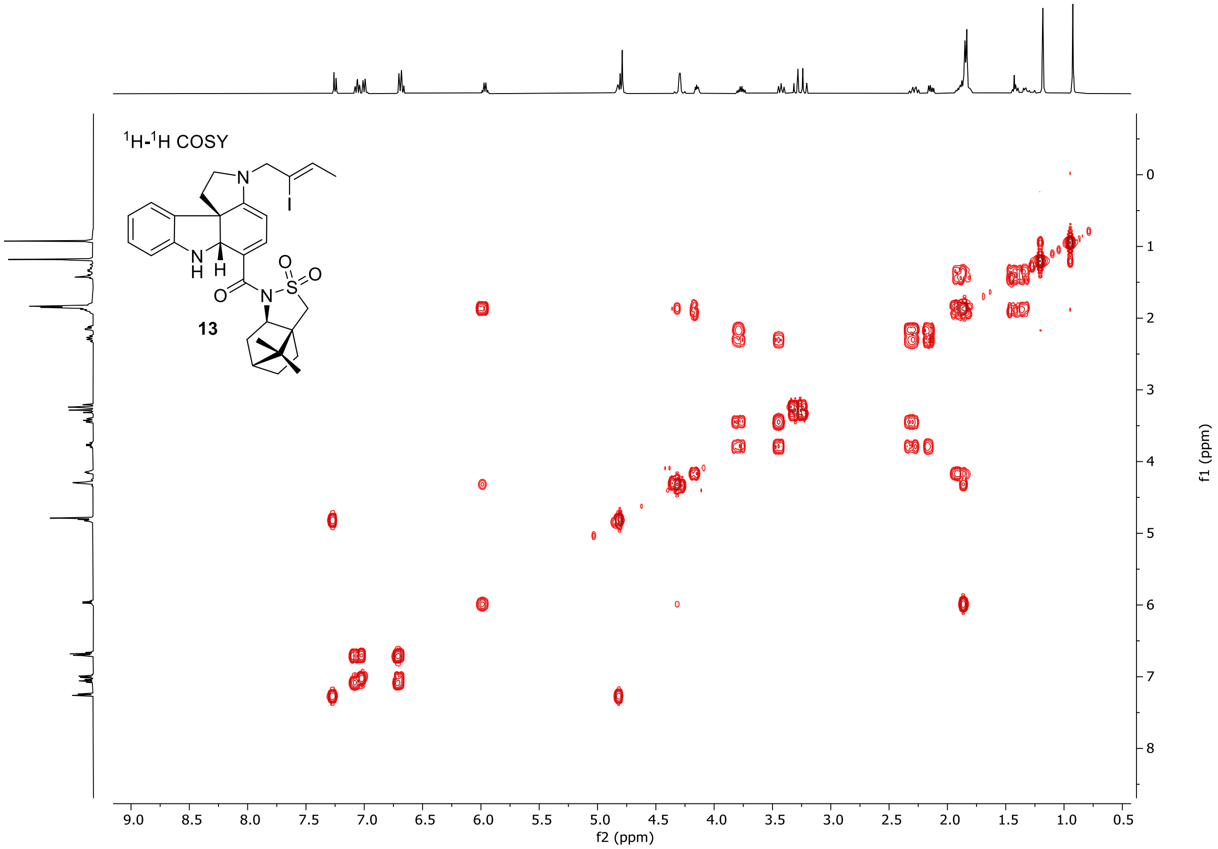

1H-1H COSY
13
f2 (ppm)
f1 (ppm)

(3-((2-(1*H*-Indol-3-yl)ethyl)((*Z*)-2-iodobut-2-en-1-yl)amino)-5-chloro-1,1-dioxido-2,3-dihydrothiophen-2-yl)((3a*S*,6*R*,7a*R*)-8,8-dimethyl-2,2-dioxidotetrahydro-3*H*-3a,6-methanobenzo[*c*]isothiazol-1(4*H*)-yl)methanone, **S23** (Spectra in $CDCl_3$)

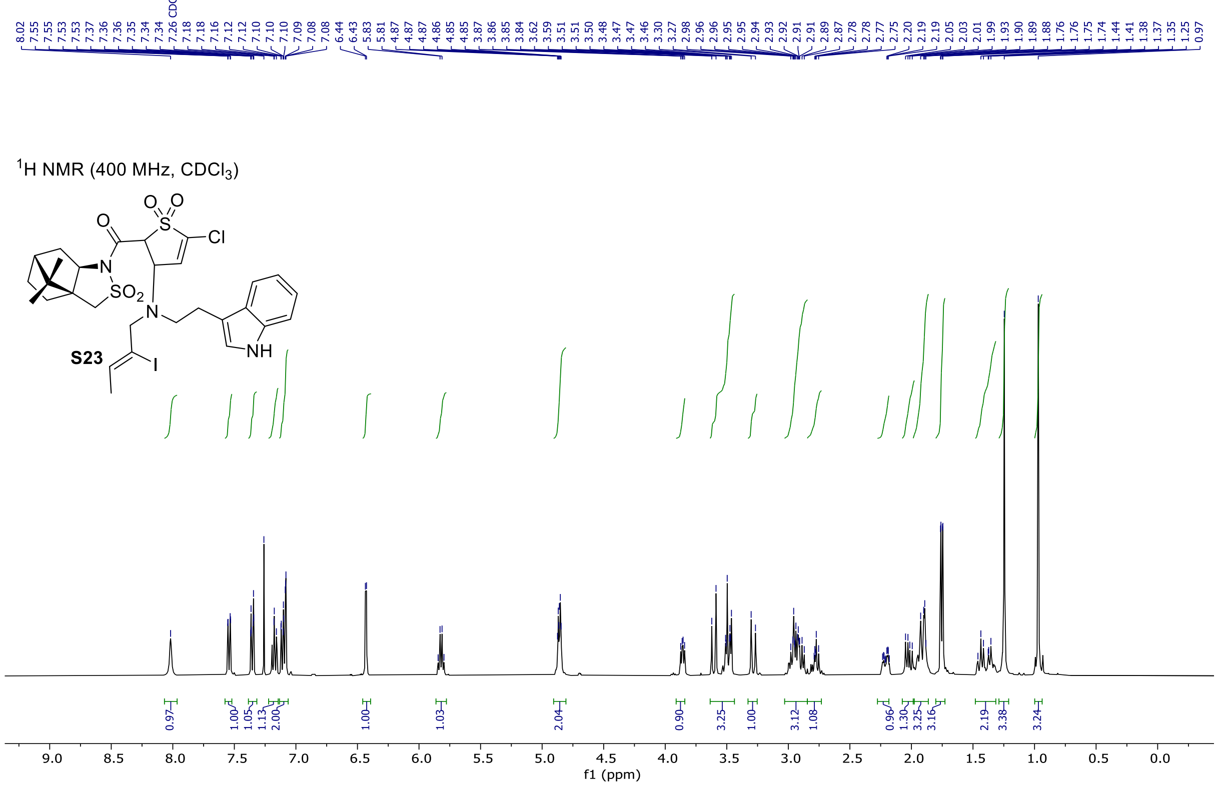

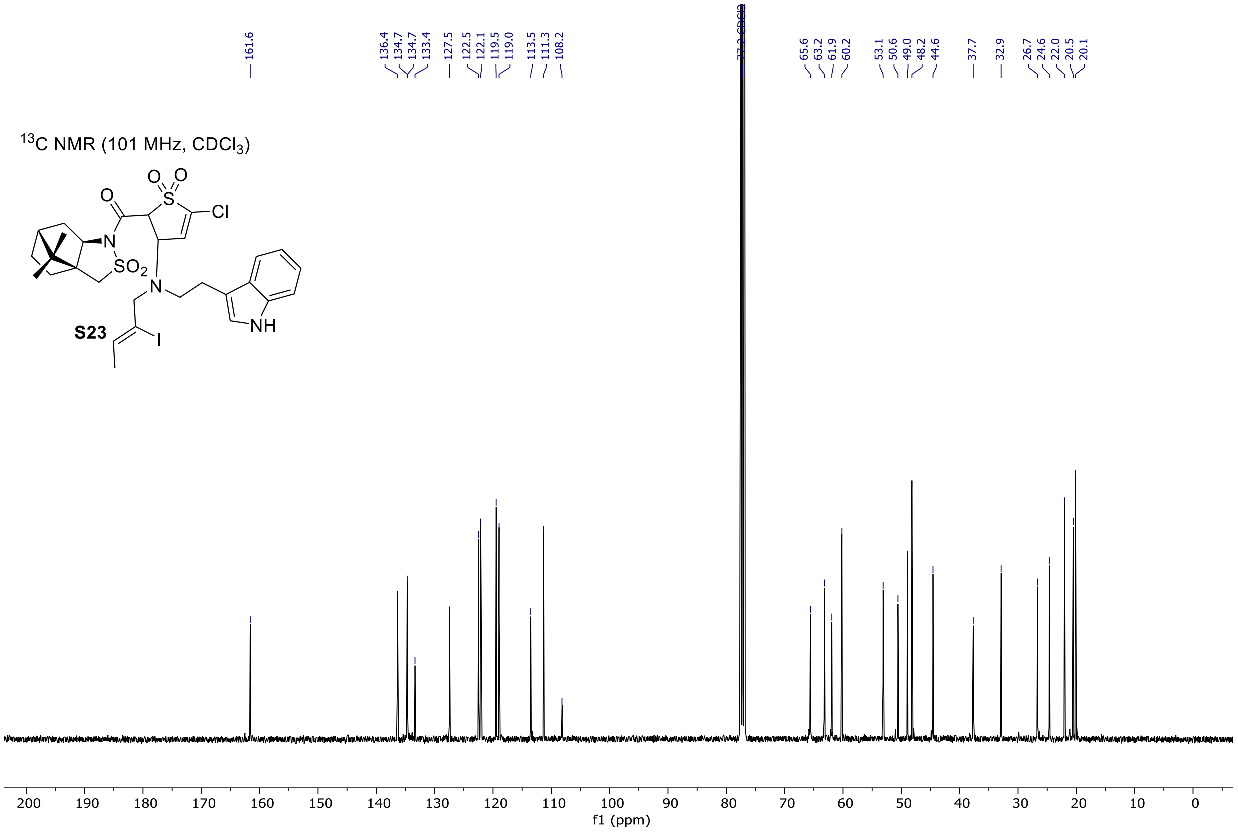

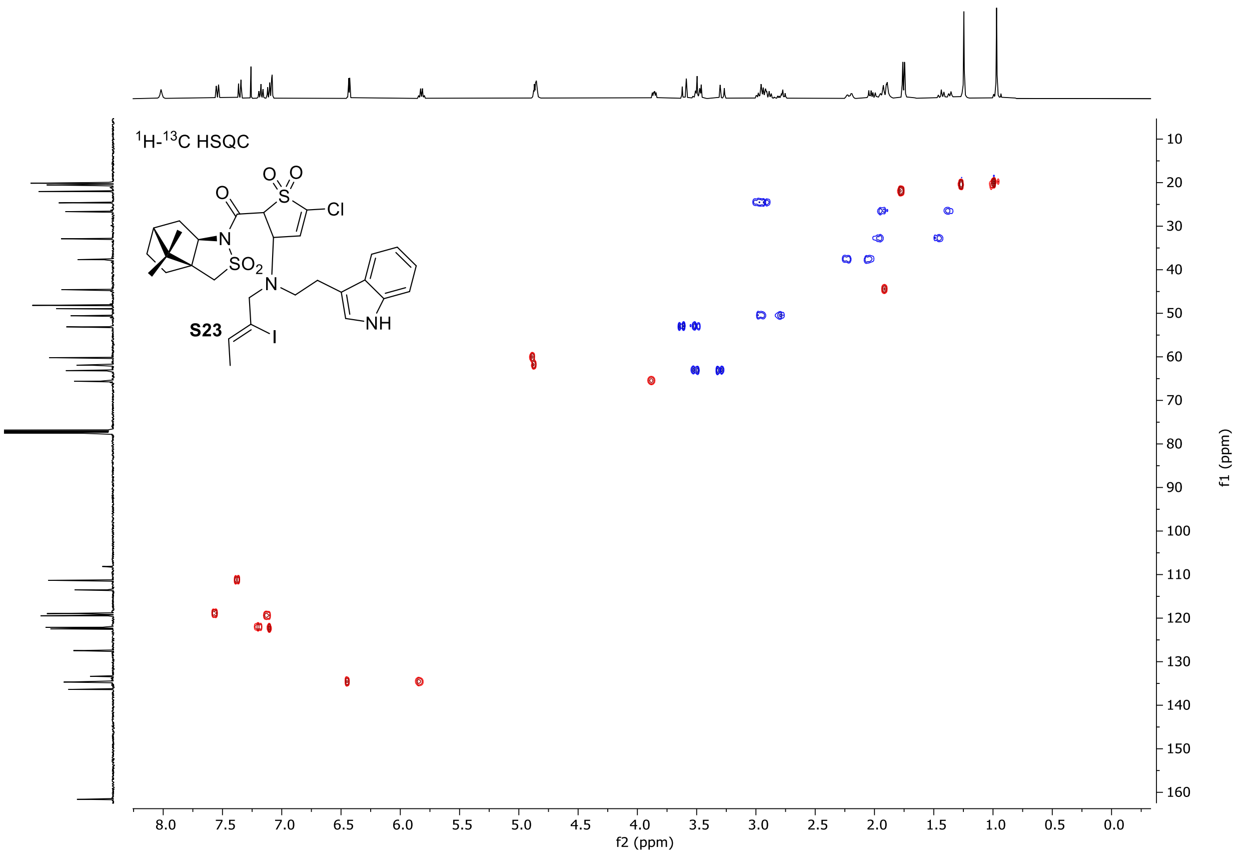

1H-13C HSQC
S23
f2 (ppm)
f1 (ppm)

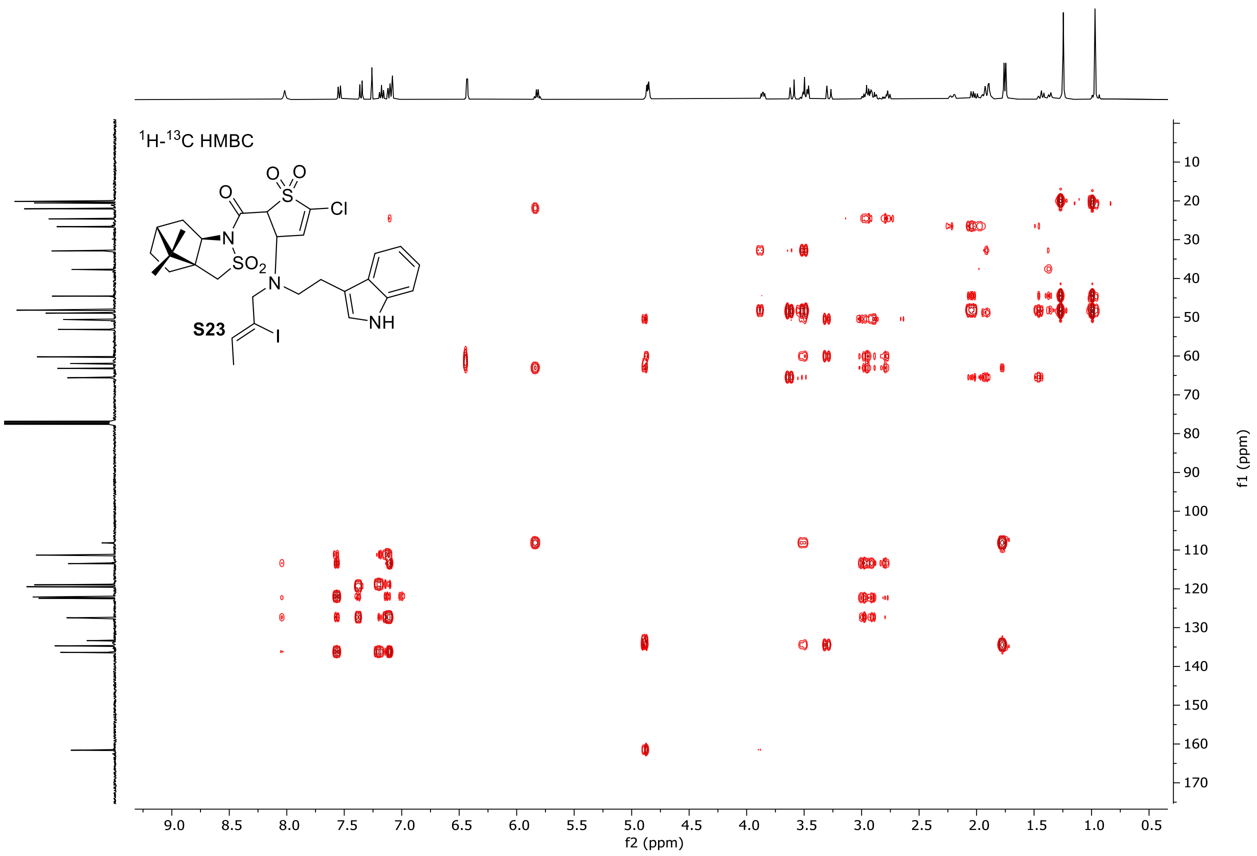

1H-13C HMBC
S23
f2 (ppm)
f1 (ppm)

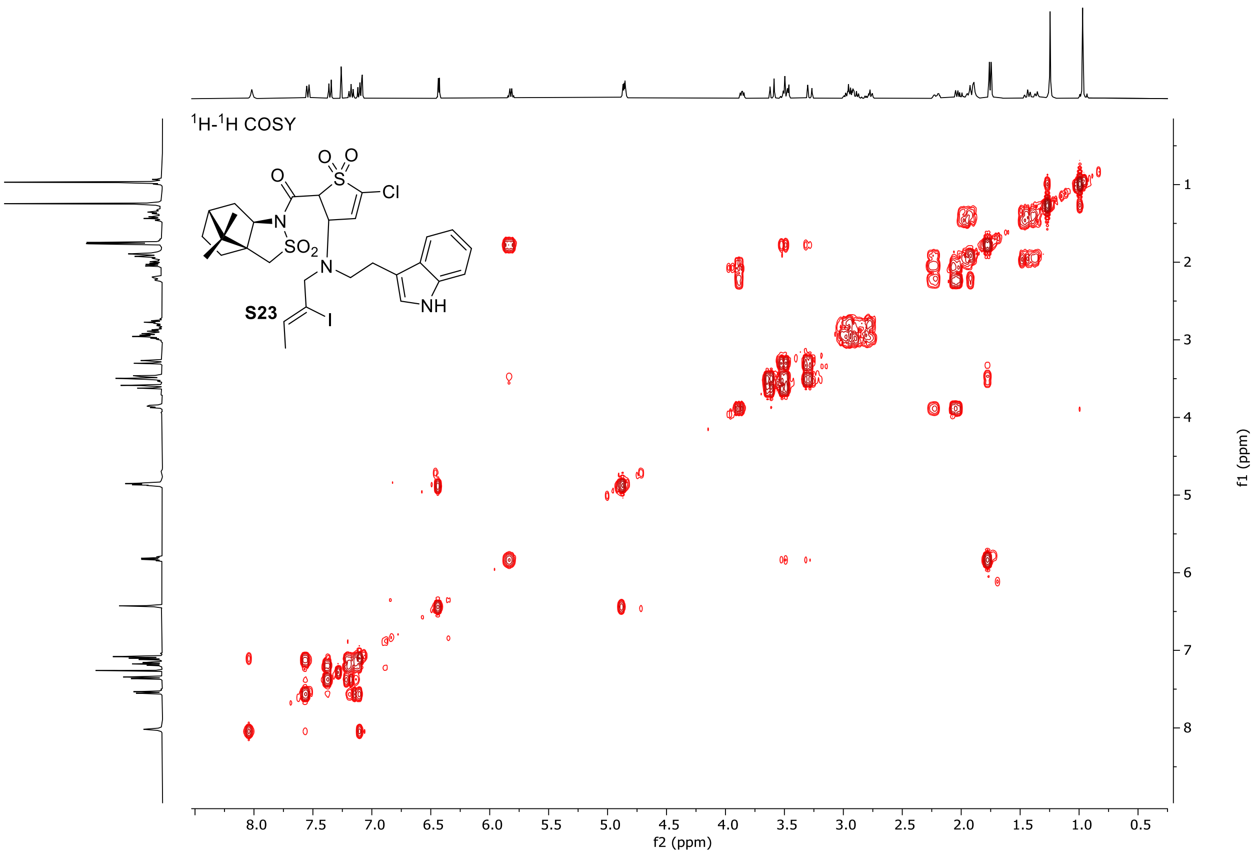

1H-1H COSY
S23
Cl
NH
I
f1 (ppm)
f2 (ppm)

((4a*S*,9a*R*)-4-Chloro-4a,9a-dihydro-9*H*-carbazol-1-yl)((3a*S*,6*R*,7a*R*)-8,8-dimethyl-2,2-dioxidotetrahydro-3*H*-3a,6-methanobenzo[*c*]isothiazol-1(4*H*)-yl)methanone, **10a** (Spectra in $CDCl_3$)

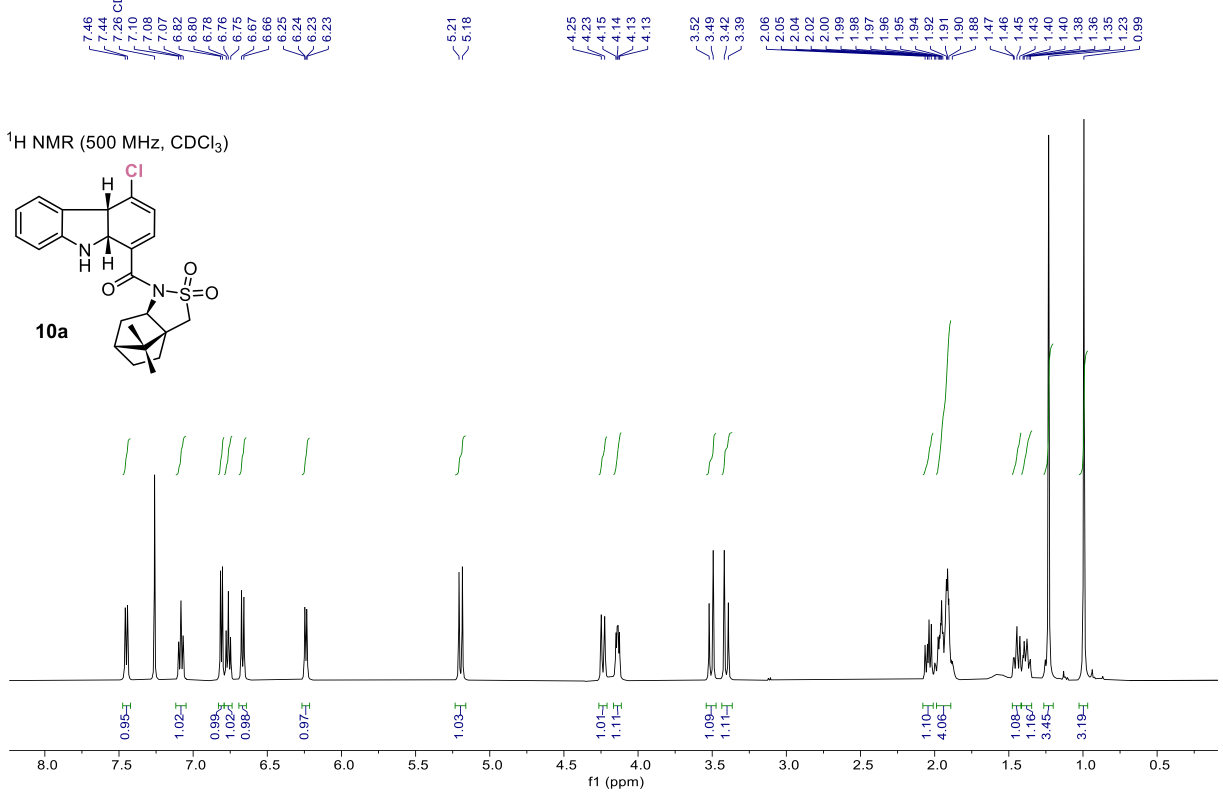

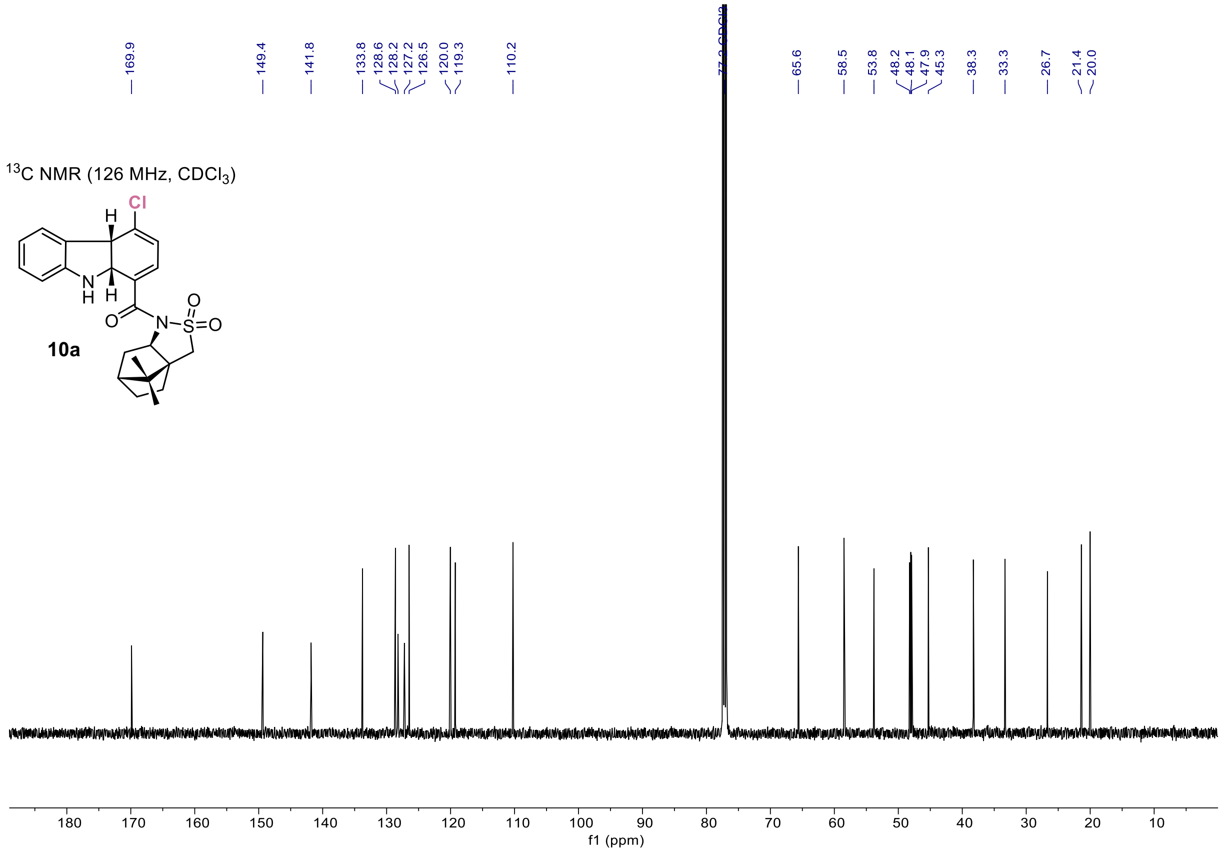

((4a*S*,9a*R*)-4-Chloro-8-methyl-4a,9a-dihydro-9*H*-carbazol-1-yl)((3a*S*,6*R*,7a*R*)-8,8-dimethyl-2,2-dioxidotetrahydro-3*H*-3a,6-methanobenzo[*c*]isothiazol-1(4*H*)-yl)methanone, **10b** (Spectra in $CDCl_3$)

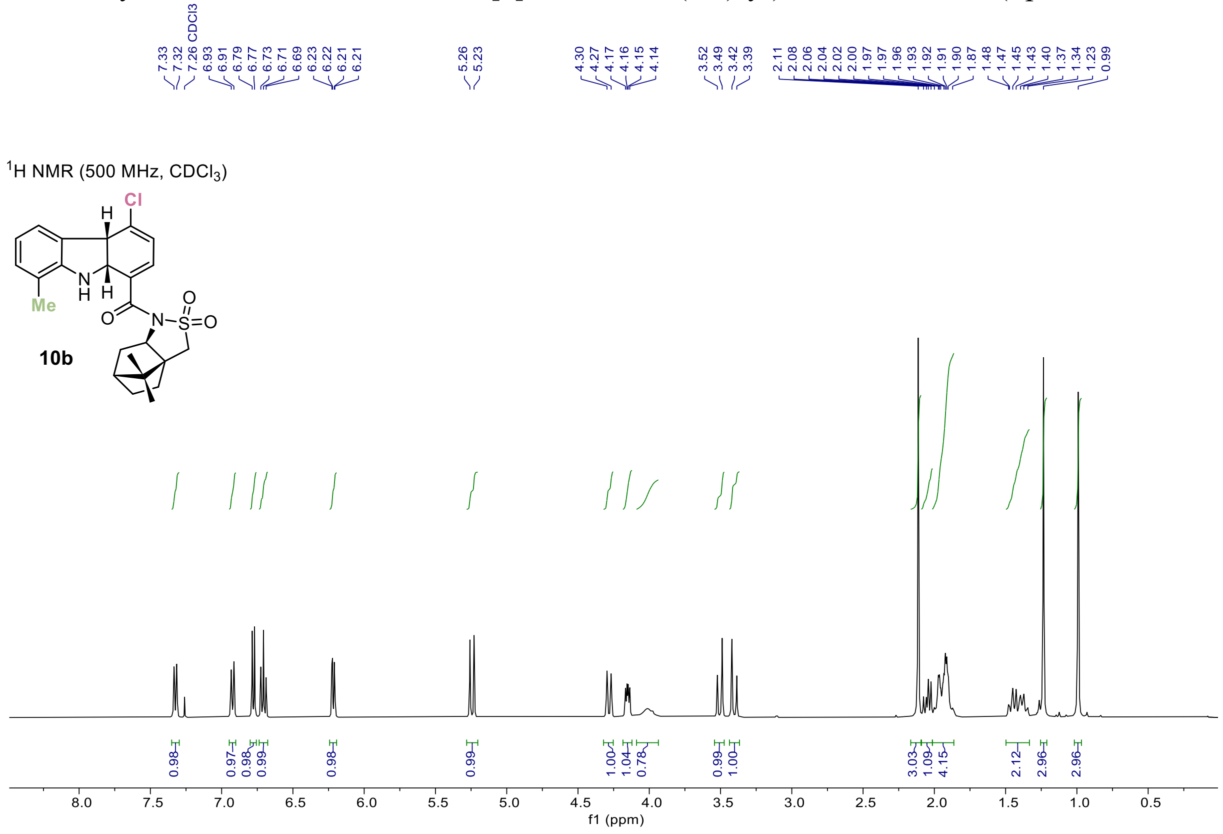

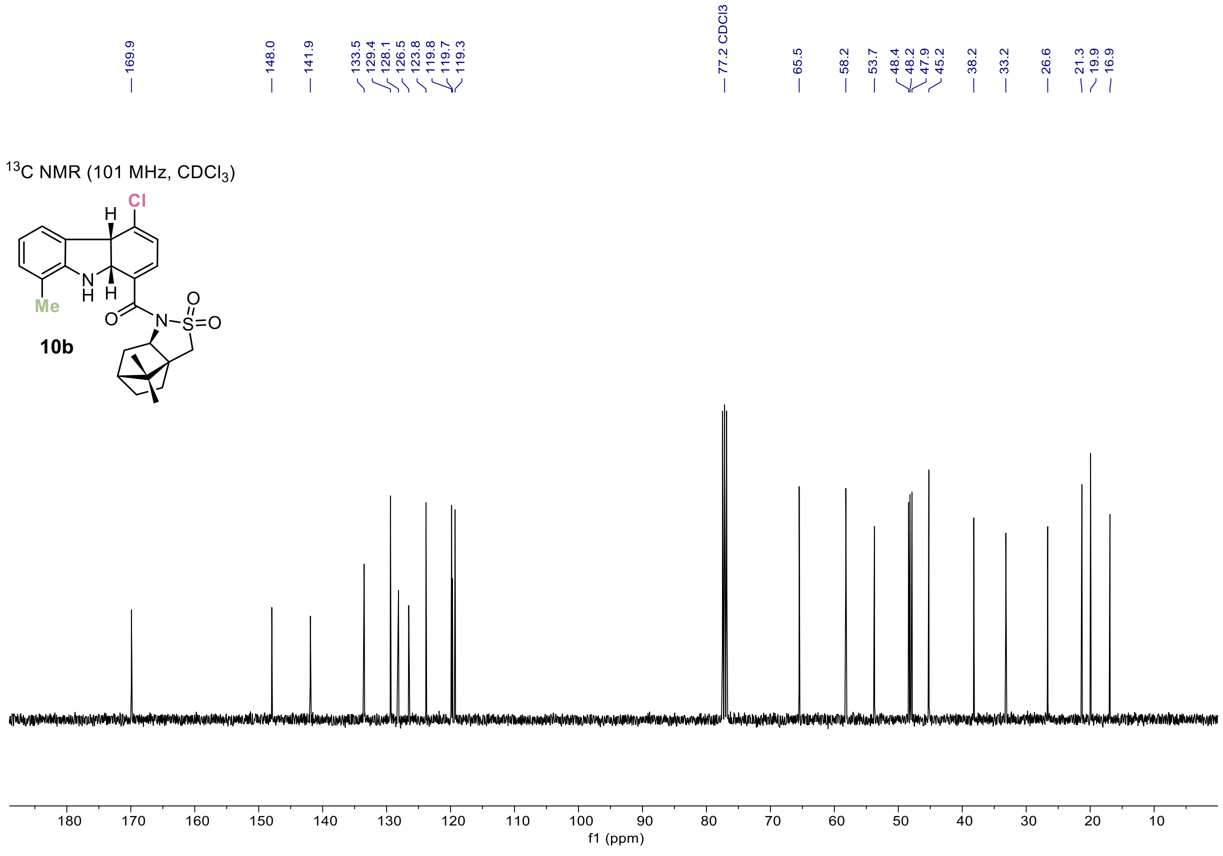

((4a*S*,9a*R*)-4-Chloro-6-(4,4,5,5-tetramethyl-1,3,2-dioxaborolan-2-yl)-4a,9a-dihydro-9*H*-carbazol-1-yl)((3a*S*,6*R*,7a*R*)-8,8-dimethyl-2,2-dioxidotetrahydro-3*H*-3a,6-methanobenzo[*c*]isothiazol-1(4*H*)-yl)methanone, **10c** (Spectra in $CDCl_3$)

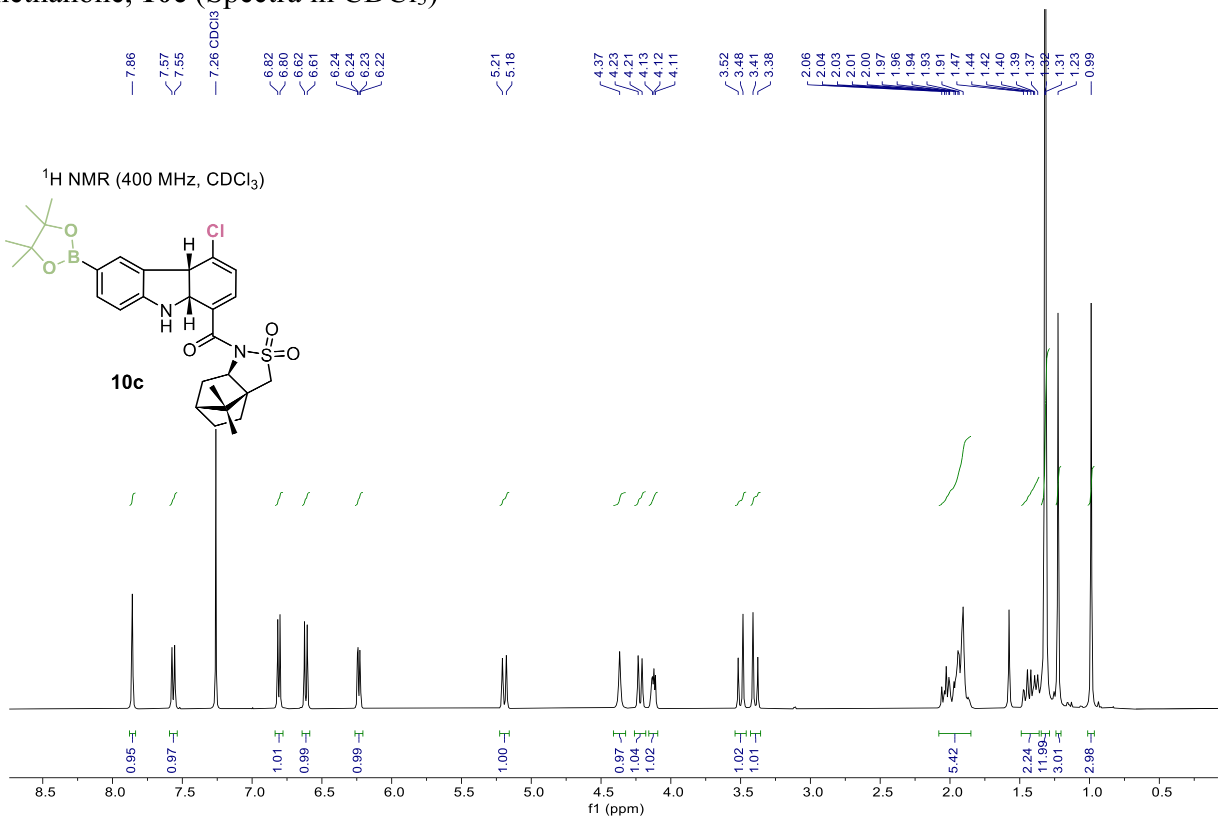

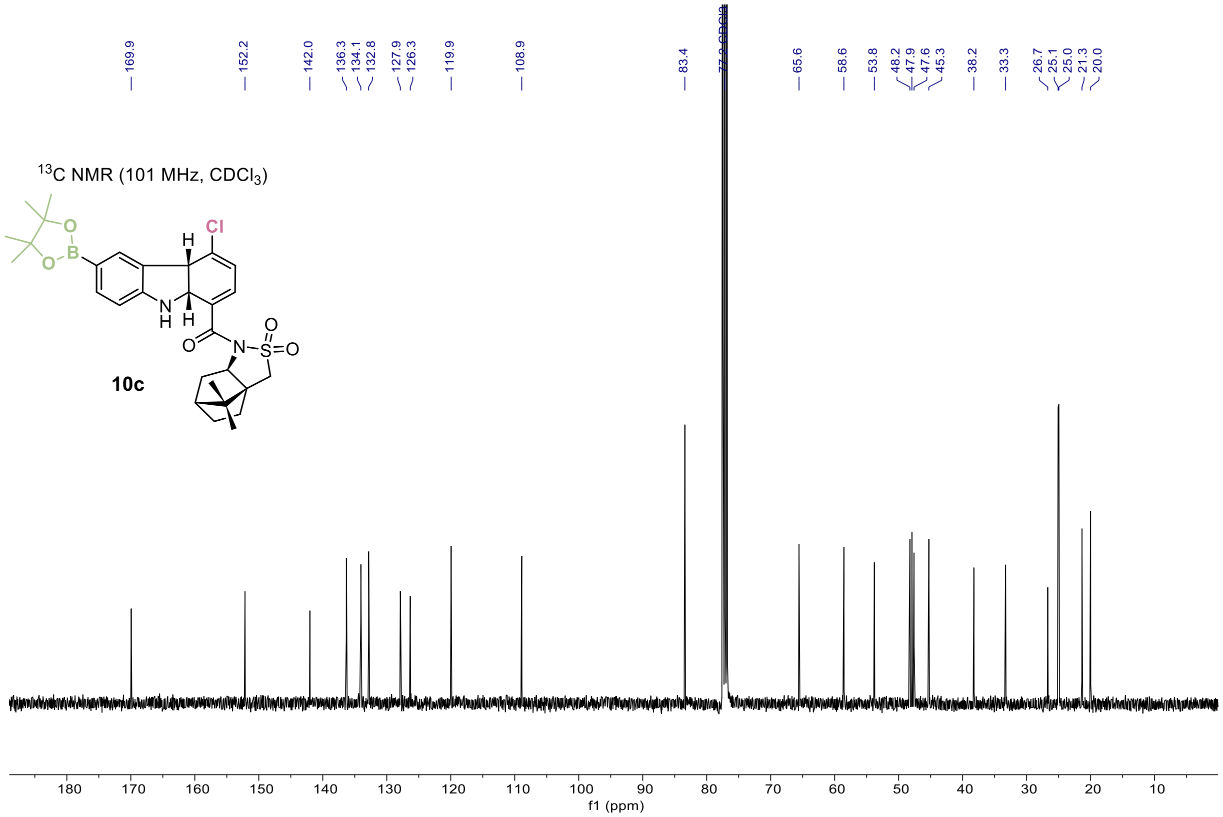

((4a*S*,9a*R*)-4-Chloro-8-iodo-4a,9a-dihydro-9*H*-carbazol-1-yl)((3a*S*,6*R*,7a*R*)-8,8-dimethyl-2,2-dioxidotetrahydro-3*H*-3a,6-methanobenzo[*c*]isothiazol-1(4*H*)-yl)methanone, **10d** (Spectra in $CDCl_3$)

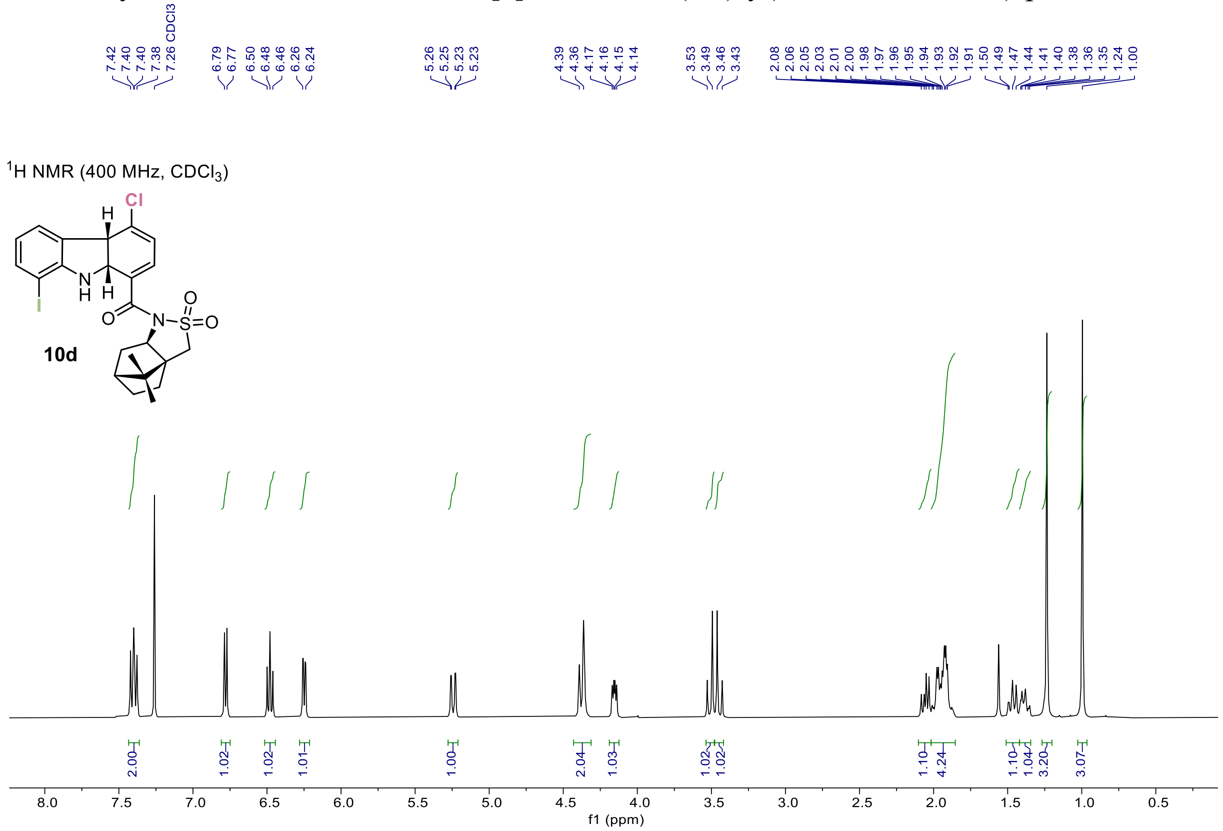

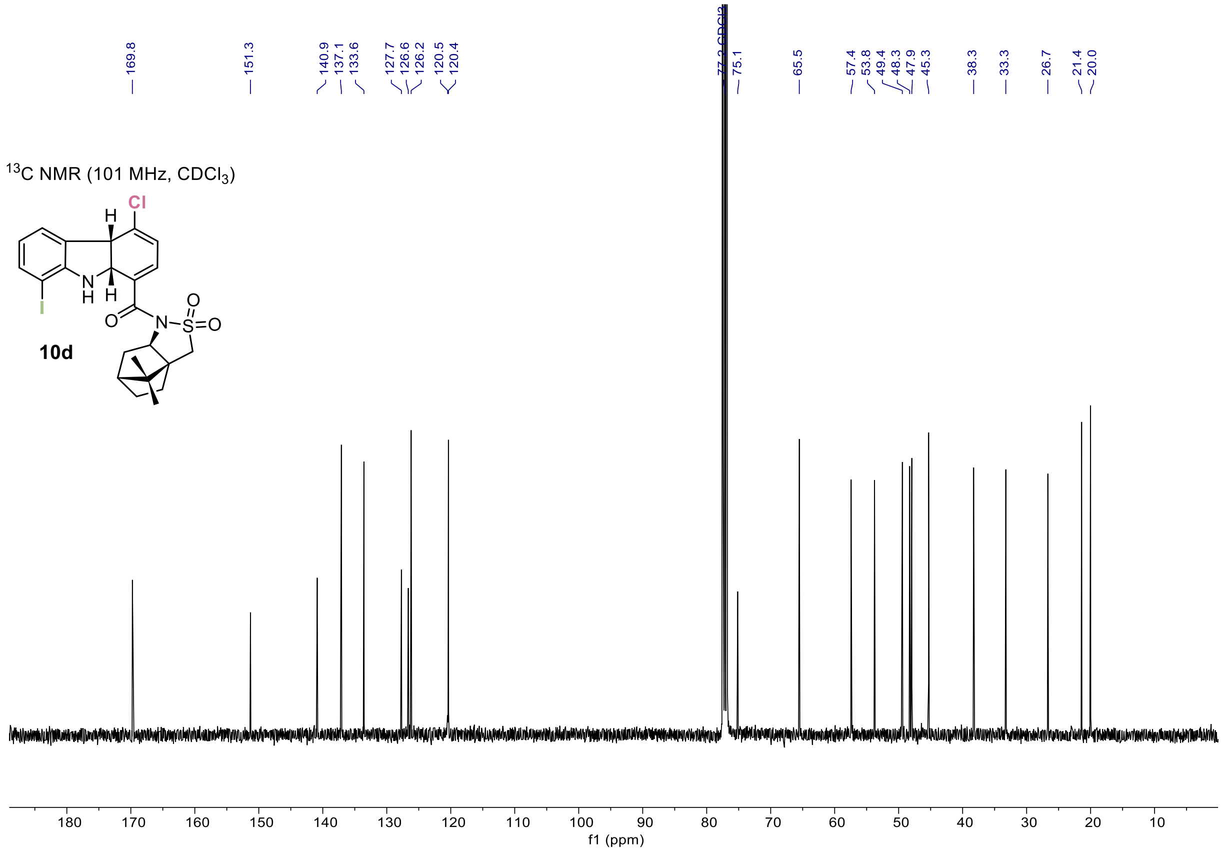

((4a*S*,9a*R*)-4-Chloro-6-nitro-4a,9a-dihydro-9*H*-carbazol-1-yl)((3a*S*,6*R*,7a*R*)-8,8-dimethyl-2,2-dioxidotetrahydro-3*H*-3a,6-methanobenzo[*c*]isothiazol-1(4*H*)-yl)methanone, **10e** (Spectra in $CDCl_3$)

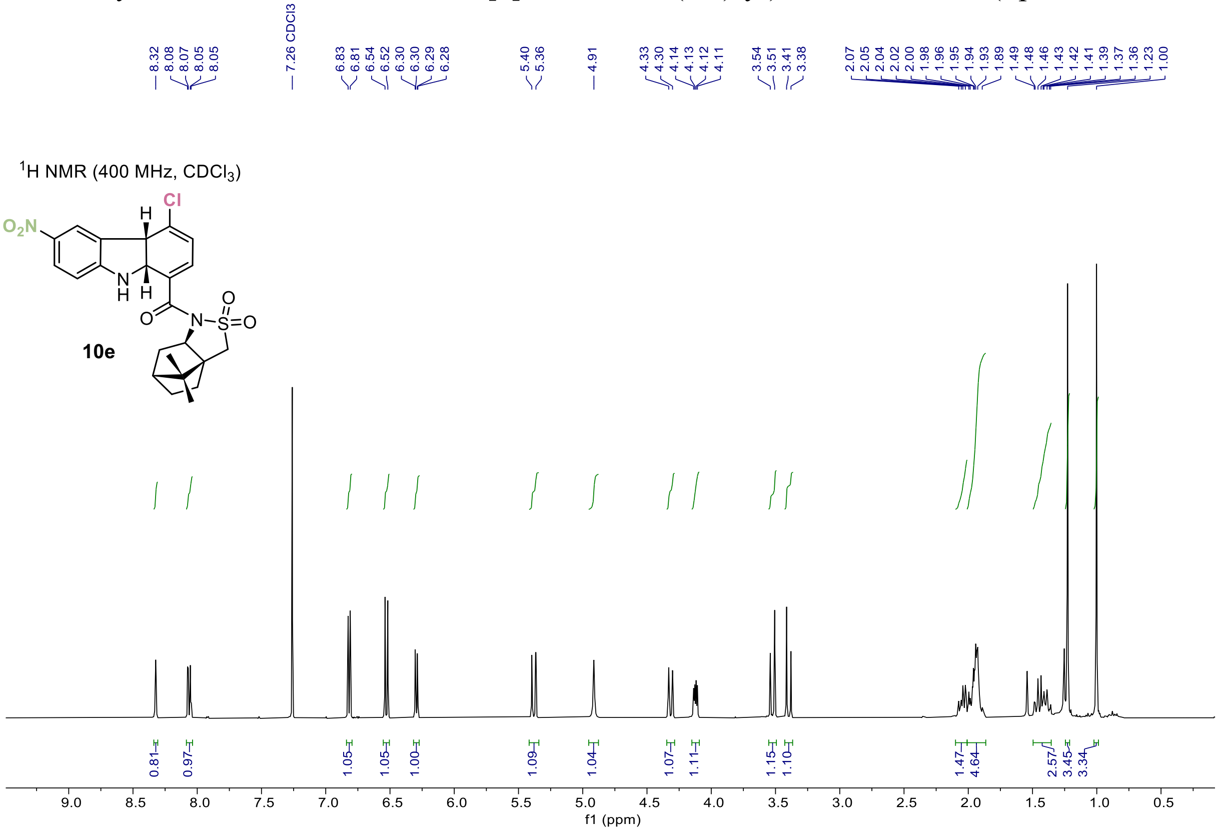

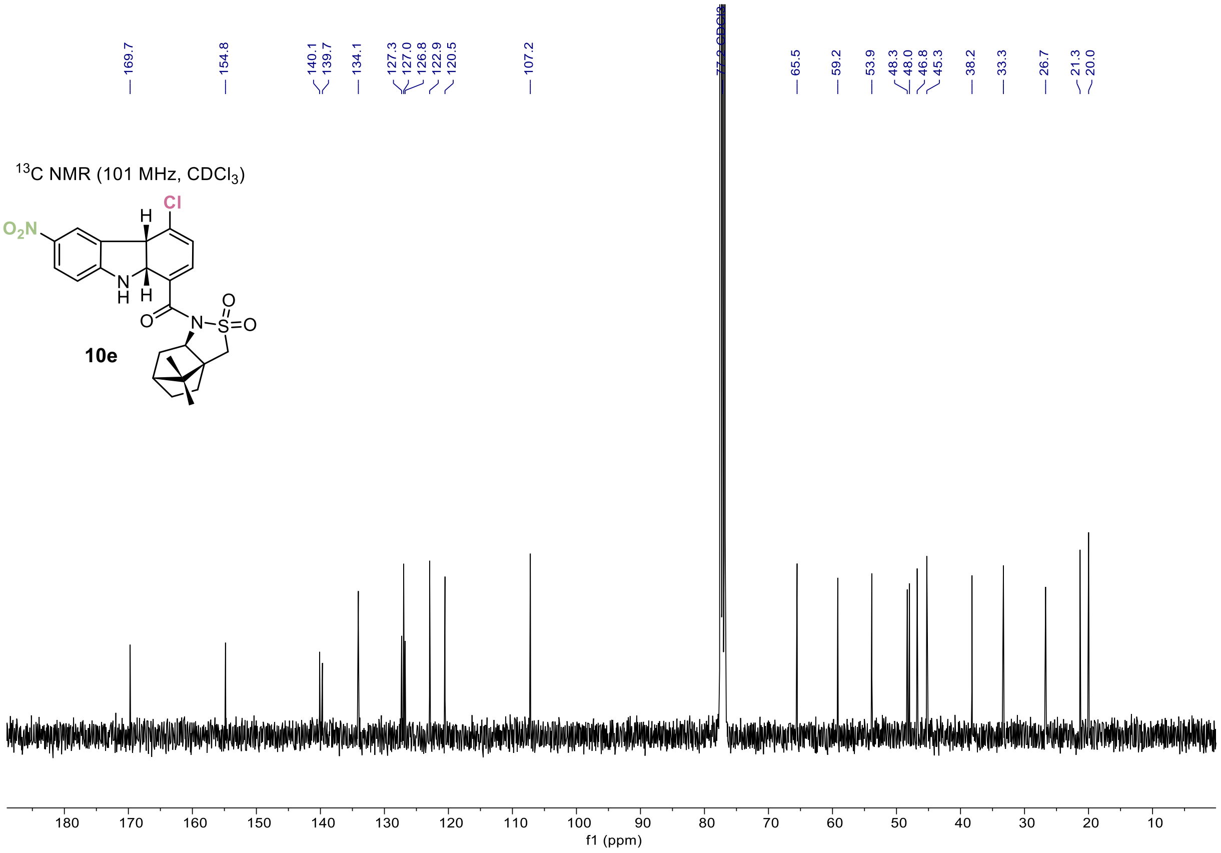

(4b*S*,8a*R*)-5-Chloro-8-((3a*S*,6*R*,7a*R*)-8,8-dimethyl-2,2-dioxidohexahydro-3*H*-3a,6-methanobenzo[*c*]isothiazole-1-carbonyl)-4b,8a-dihydro-9*H*-carbazole-3-carbonitrile, **10f** (Spectra in $CDCl_3$)

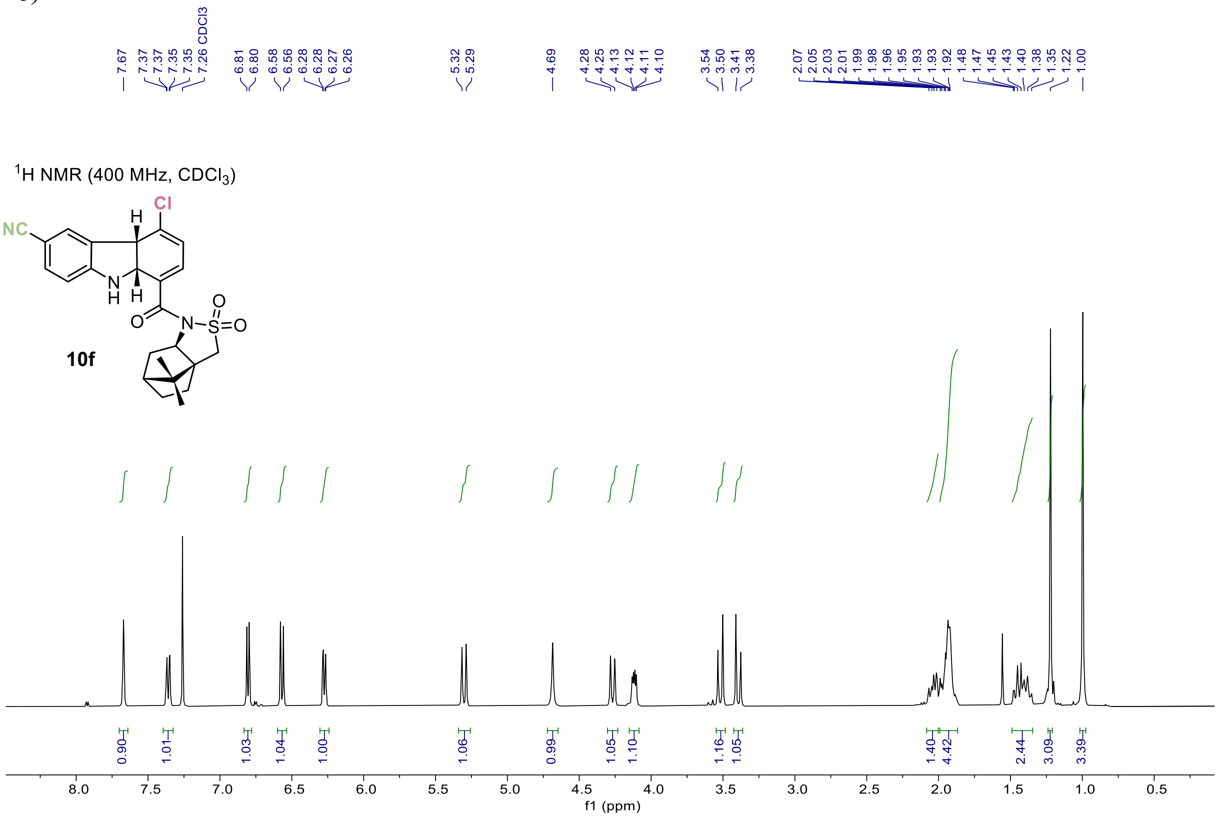

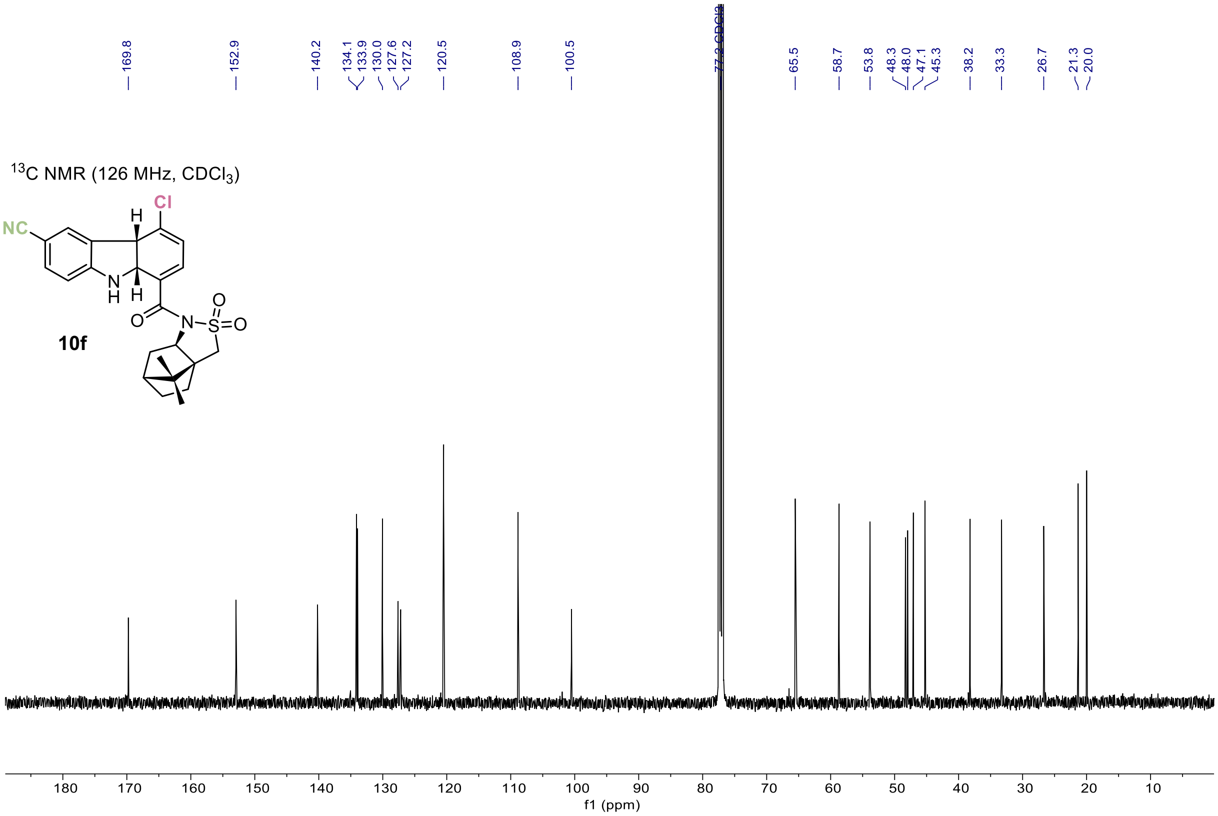

((4a*S*,9a*R*)-4-Chloro-7-fluoro-4a,9a-dihydro-9*H*-carbazol-1-yl)((3a*S*,6*R*,7a*R*)-8,8-dimethyl-2,2-dioxidotetrahydro-3*H*-3a,6-methanobenzo[*c*]isothiazol-1(4*H*)-yl)methanone, **10g** (Spectra in $CDCl_3$)

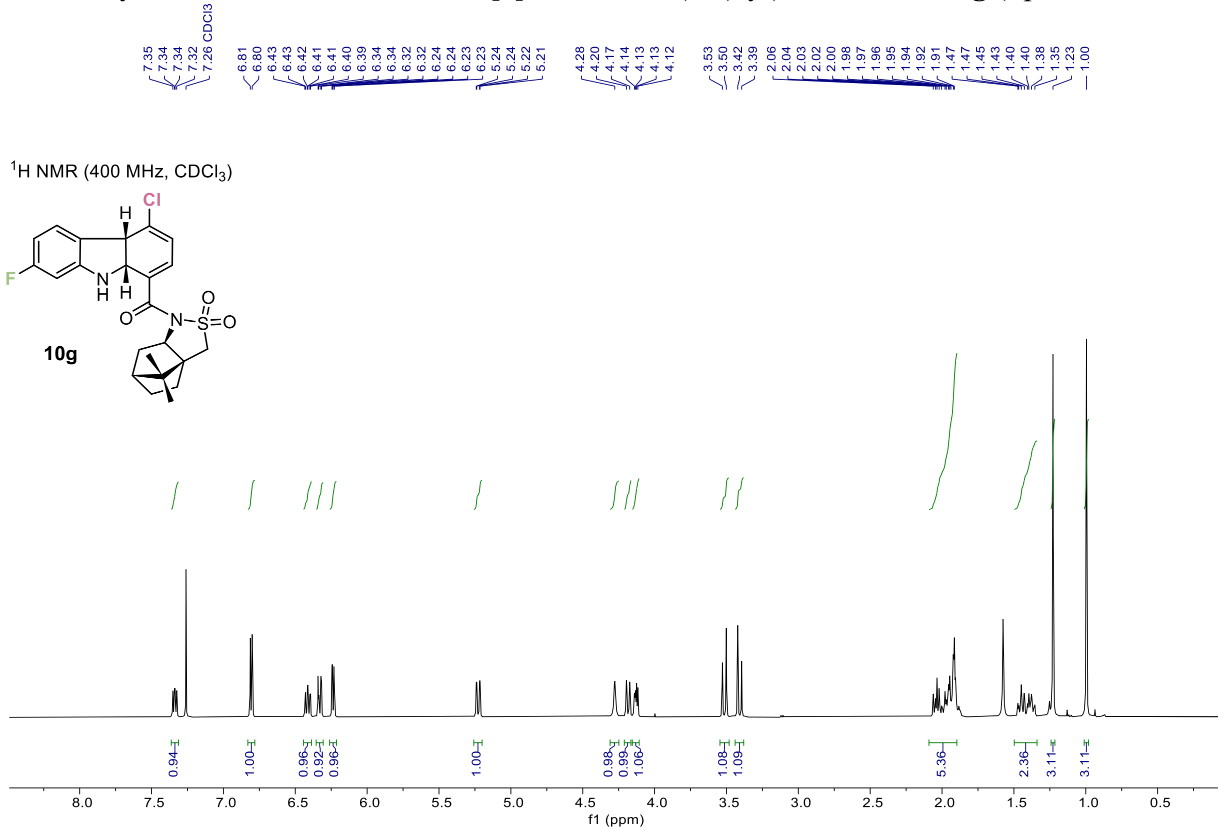

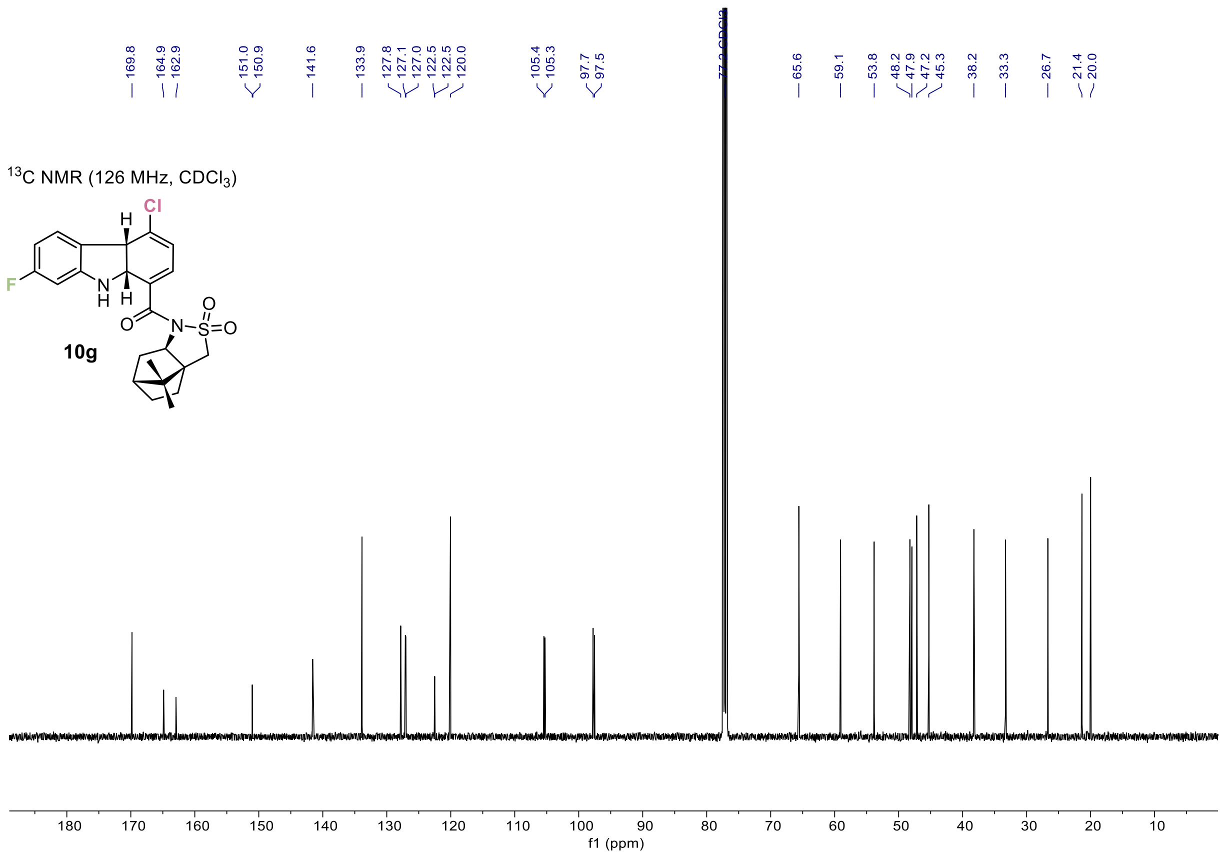

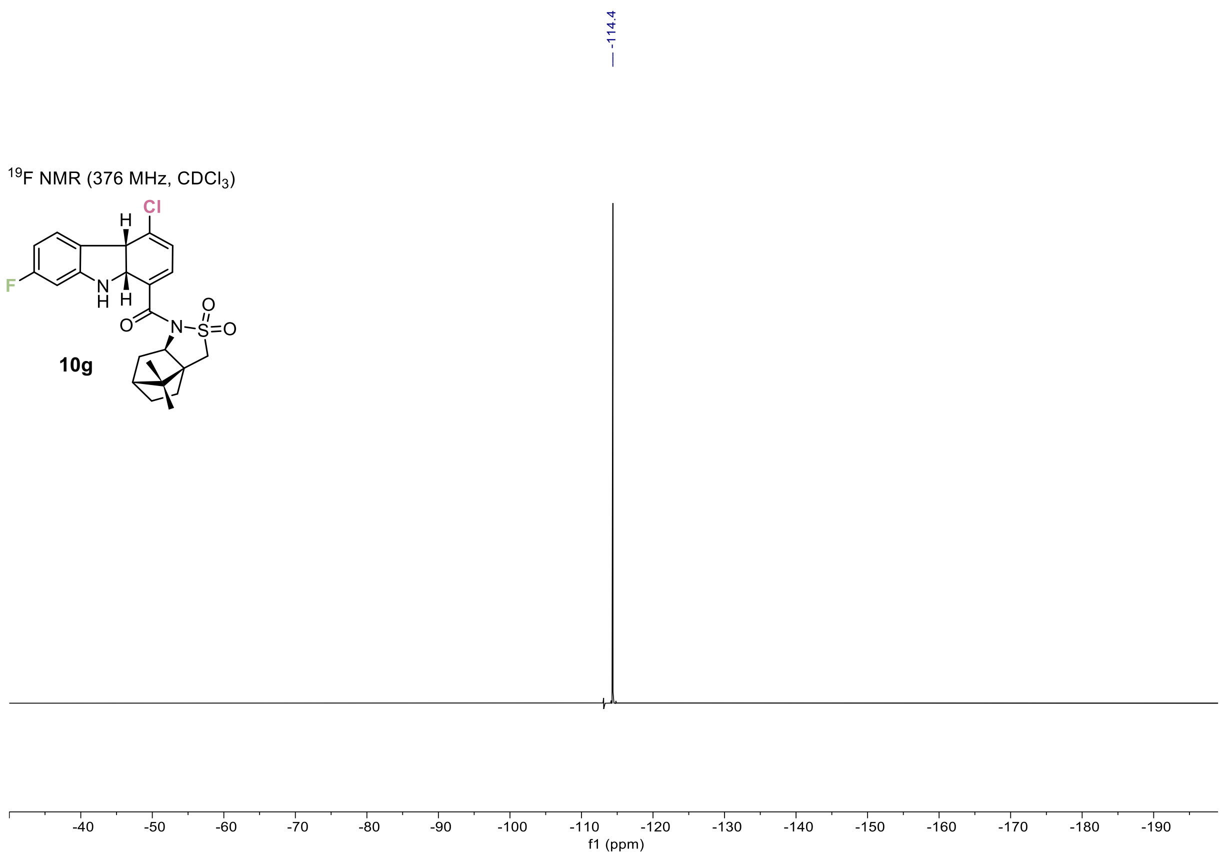
-114.4
^{19}F NMR (376 MHz, CDCl$_3$)
Cl
H
F
N
H
H
O
N
S
O
O
10g
-40
-50
-60
-70
-80
-90
-100
-110
-120
-130
-140
-150
-160
-170
-180
-190
f1 (ppm)

((4a*S*,9a*R*)-4-Chloro-5-fluoro-4a,9a-dihydro-9*H*-carbazol-1-yl)((3a*S*,6*R*,7a*R*)-8,8-dimethyl-2,2-dioxidotetrahydro-3*H*-3a,6-methanobenzo[*c*]isothiazol-1(4*H*)-yl)methanone, **10h** (Spectra in $CDCl_3$)

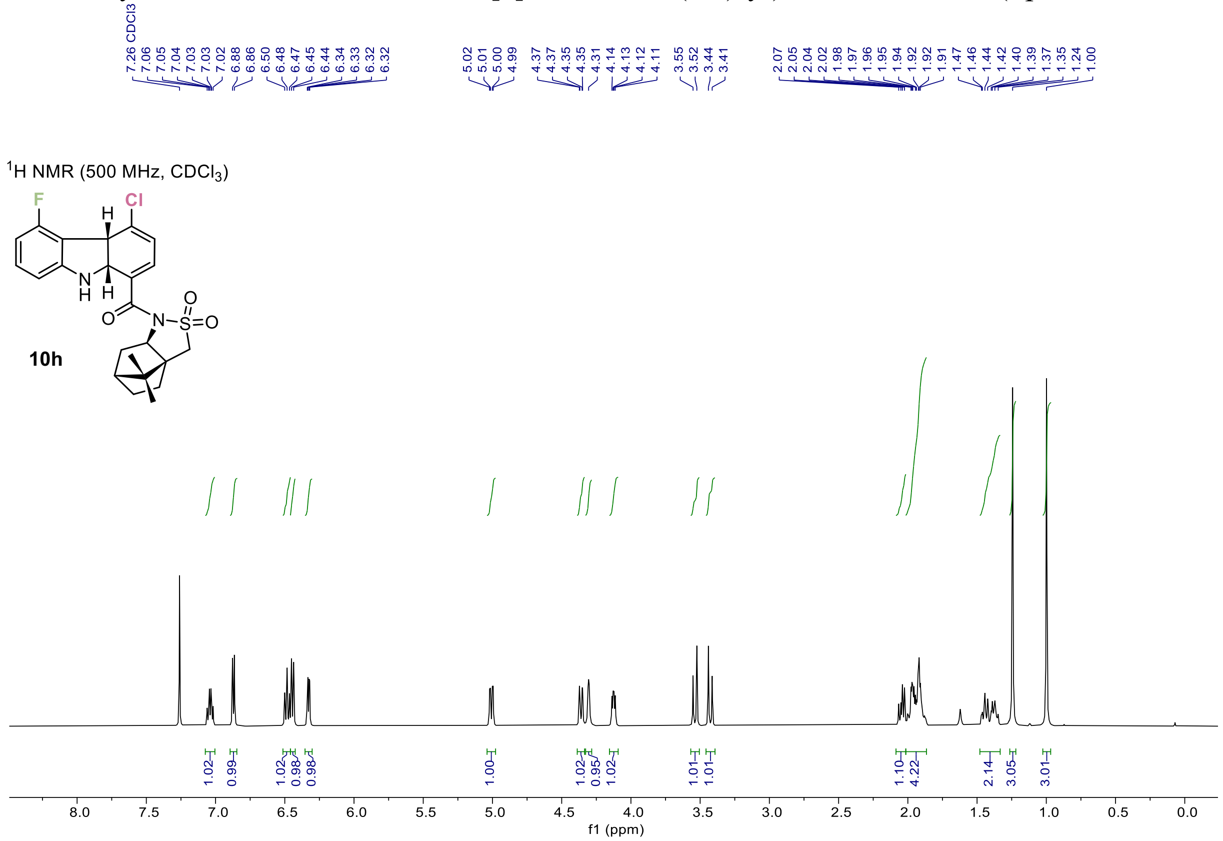

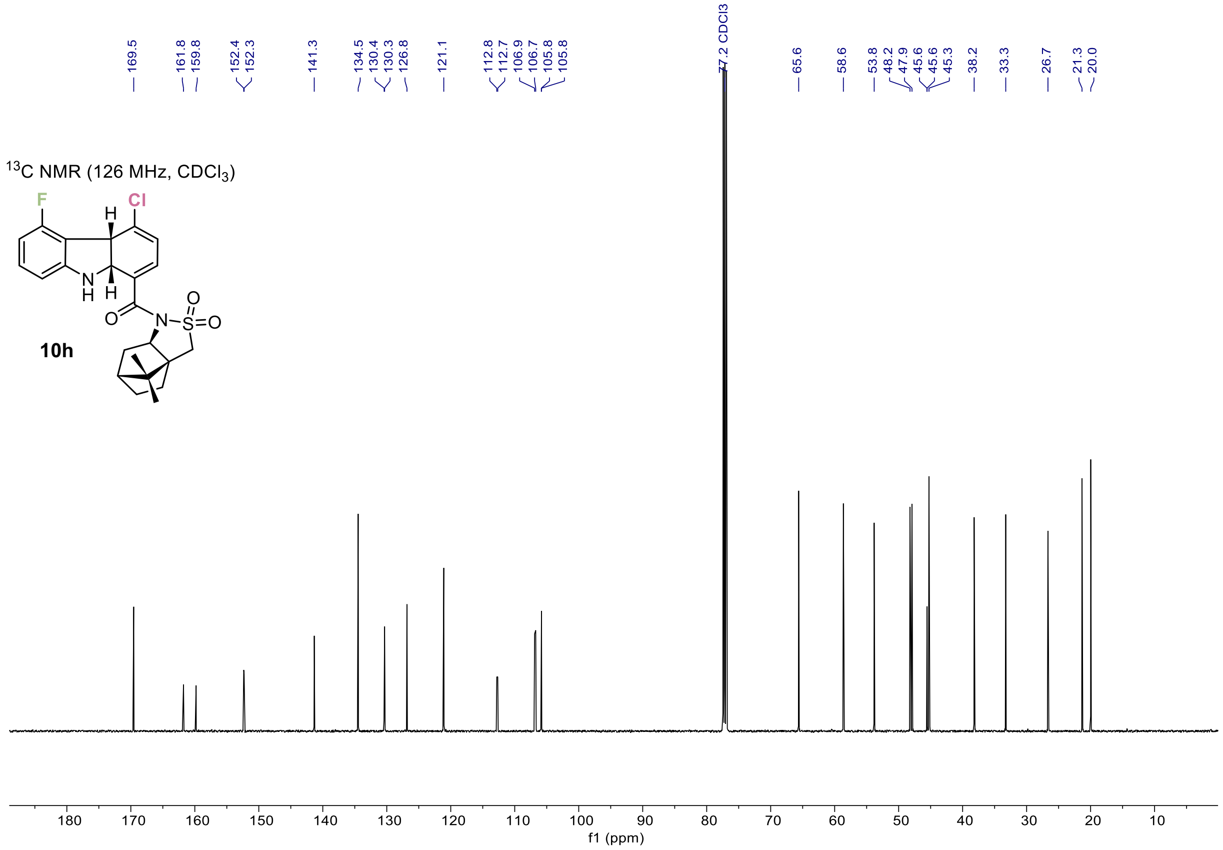

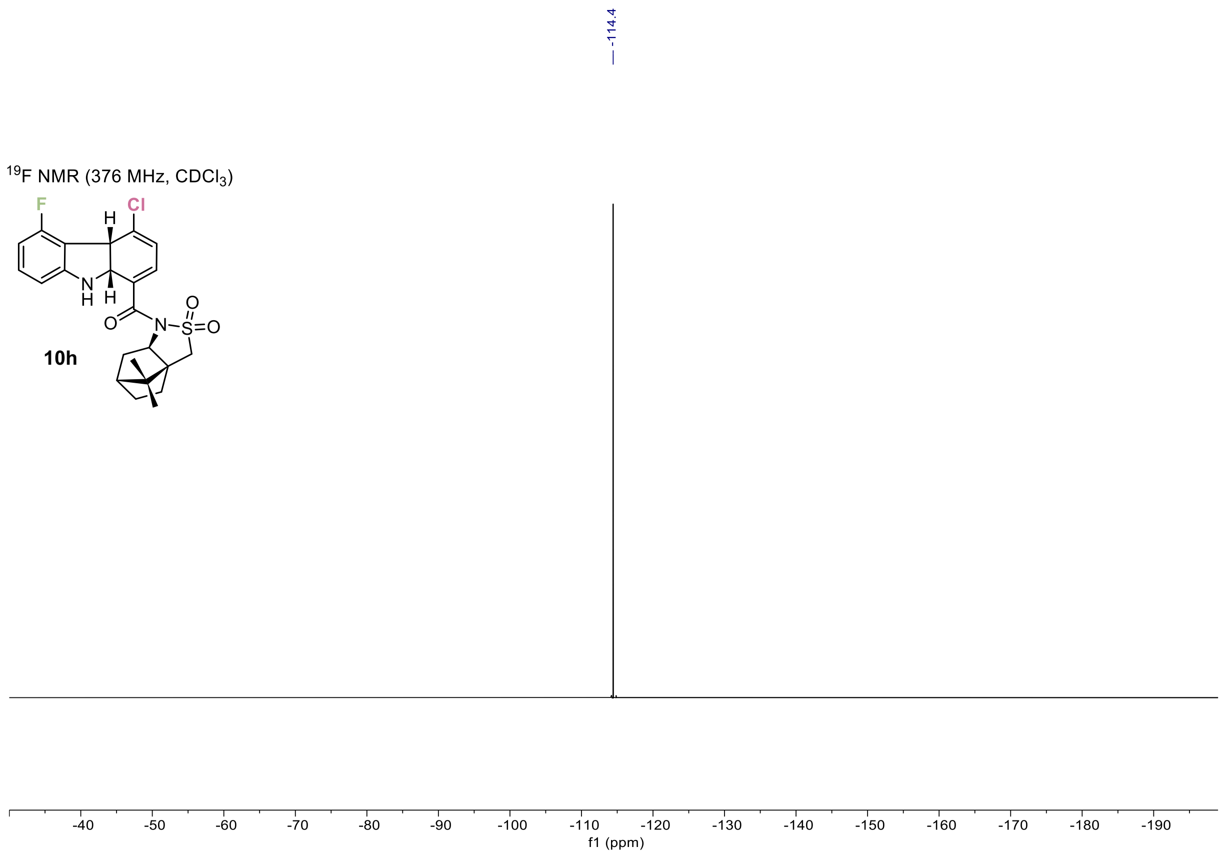
-114.4
^{19}F NMR (376 MHz, CDCl$_3$)
F
Cl
H
N
H
H
O
N
S
O
O
10h
-40
-50
-60
-70
-80
-90
-100
-110
-120
-130
-140
-150
-160
-170
-180
-190
f1 (ppm)

Methyl (4b*S*,8a*R*)-5-chloro-8-((3a*S*,6*R*,7a*R*)-8,8-dimethyl-2,2-dioxidohexahydro-3*H*-3a,6-methanobenzo[*c*]isothiazole-1-carbonyl)-4b,8a-dihydro-9*H*-carbazole-2-carboxylate, **10i** (Spectra in $CDCl_3$)

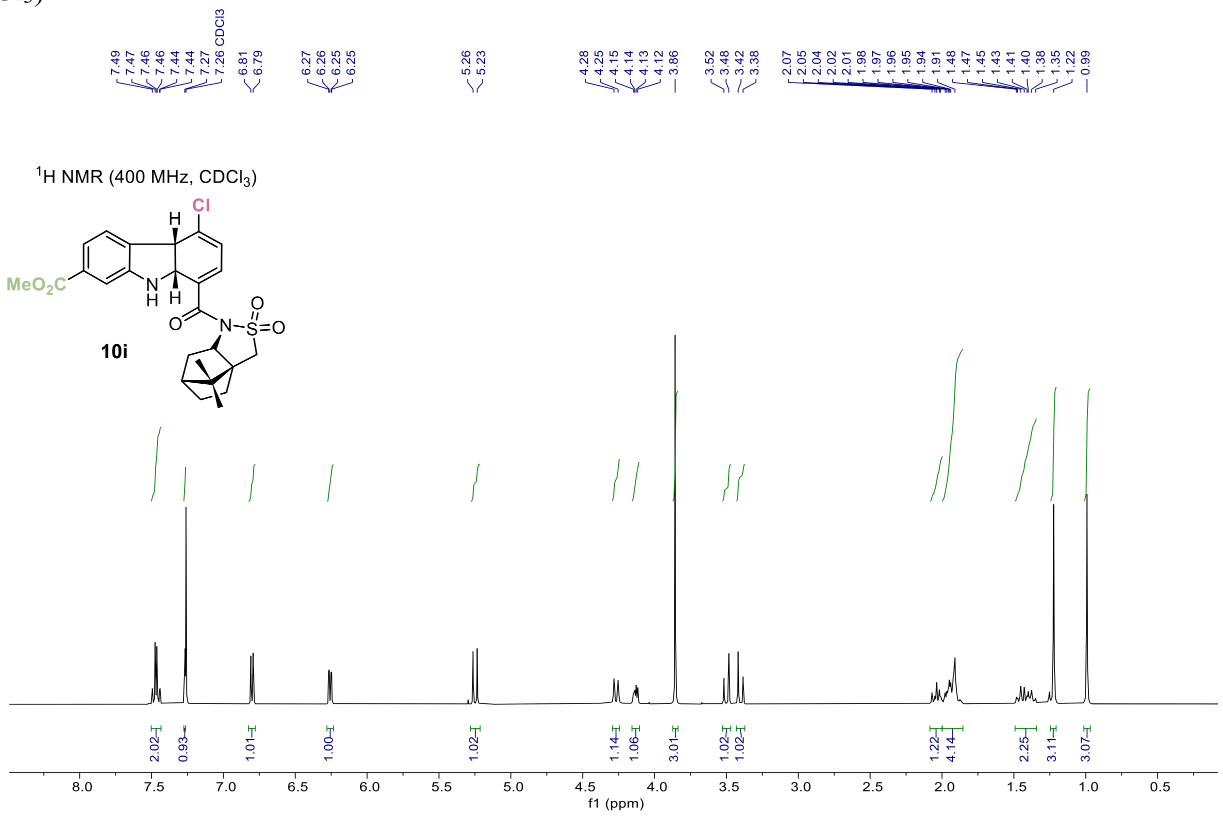

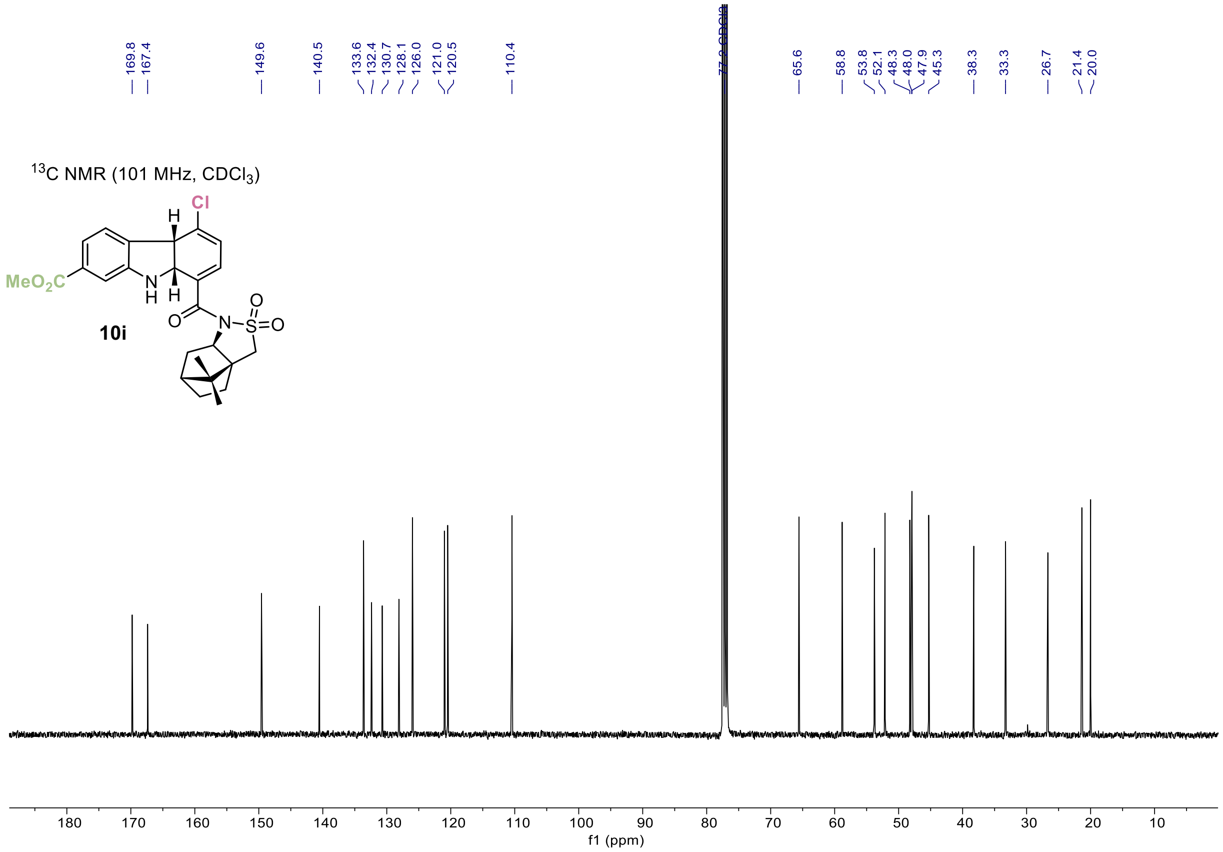

((4a*S*,9a*R*)-4-Chloro-7-methoxy-4a,9a-dihydro-9*H*-carbazol-1-yl)((3a*S*,6*R*,7a*R*)-8,8-dimethyl-2,2-dioxidotetrahydro-3*H*-3a,6-methanobenzo[*c*]isothiazol-1(4*H*)-yl)methanone, **10j** (Spectra in $CDCl_3$)

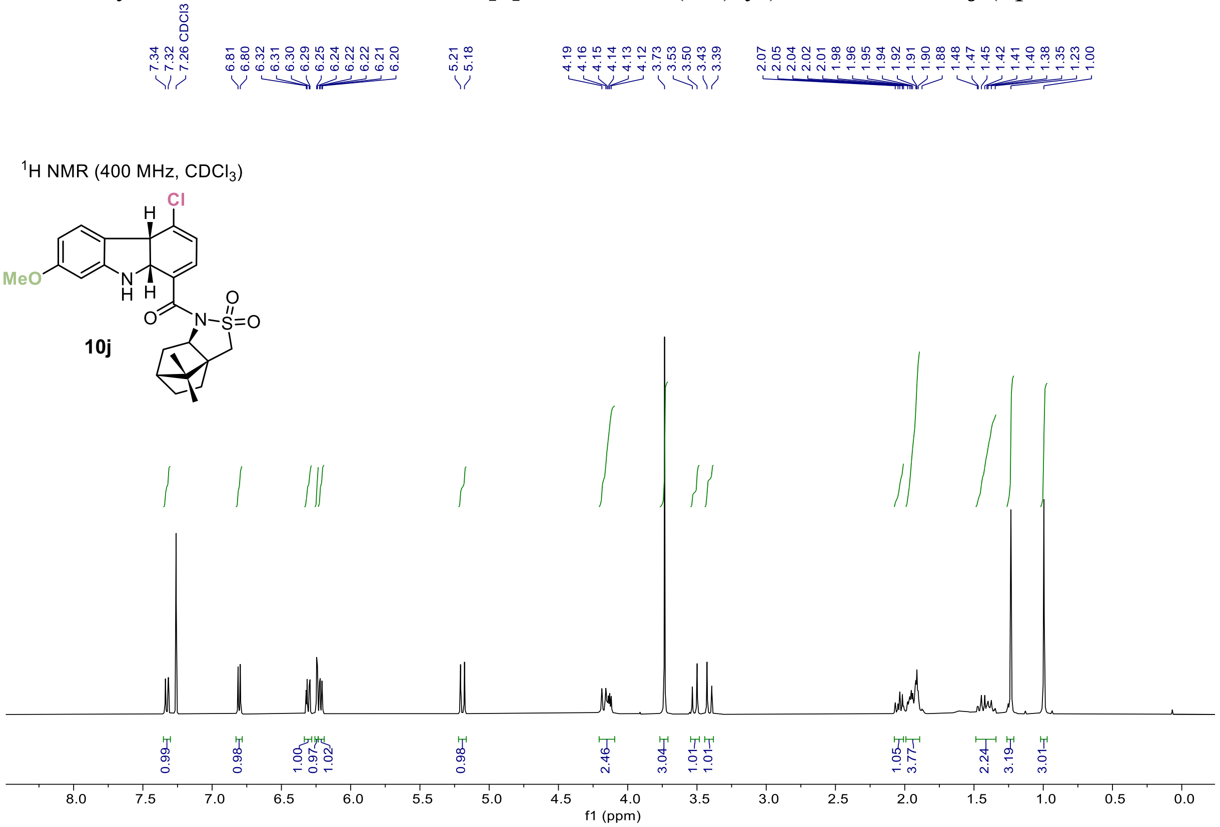

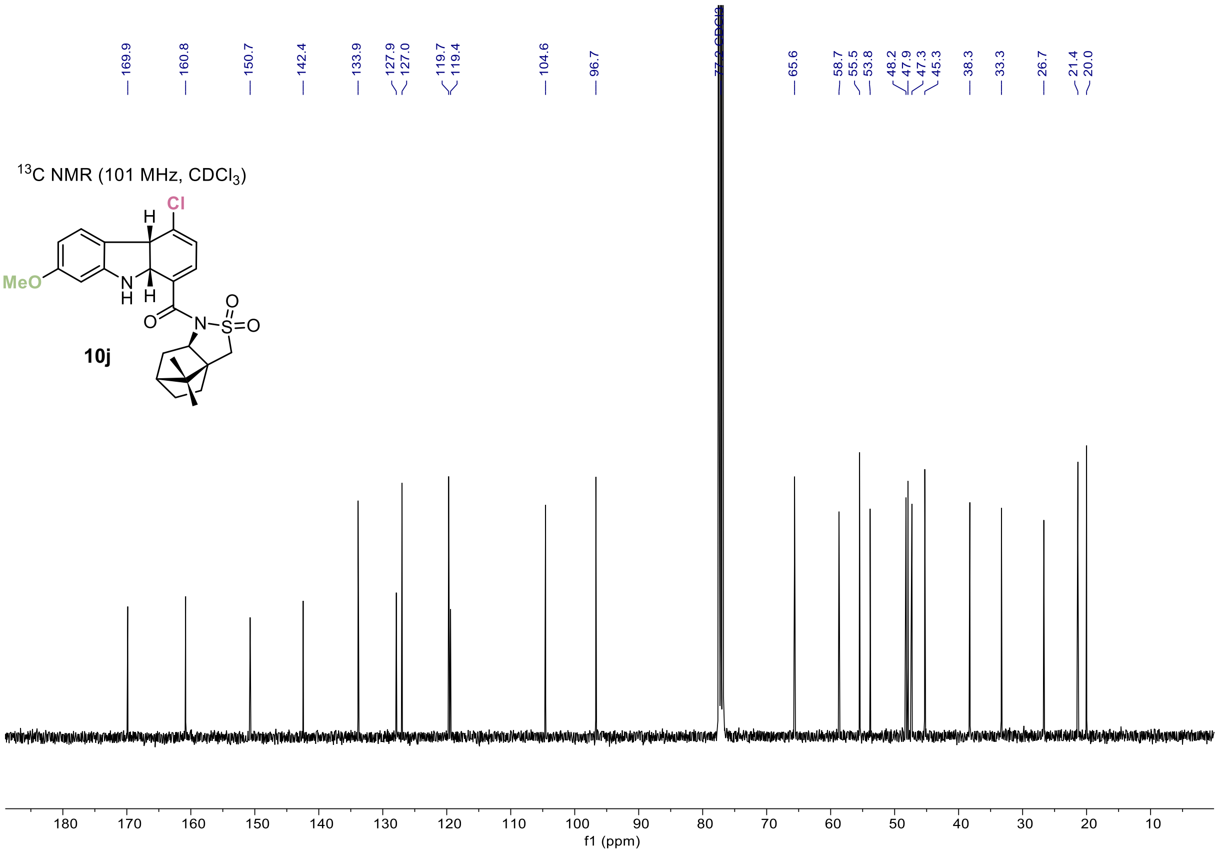

((4a*S*,9a*R*)-4-Chloro-6,7-dimethoxy-4a,9a-dihydro-9*H*-carbazol-1-yl)((3a*S*,6*R*,7a*R*)-8,8-dimethyl-2,2-dioxidotetrahydro-3*H*-3a,6-methanobenzo[*c*]isothiazol-1(4*H*)-yl)methanone, **10k** (Spectra in $CDCl_3$)

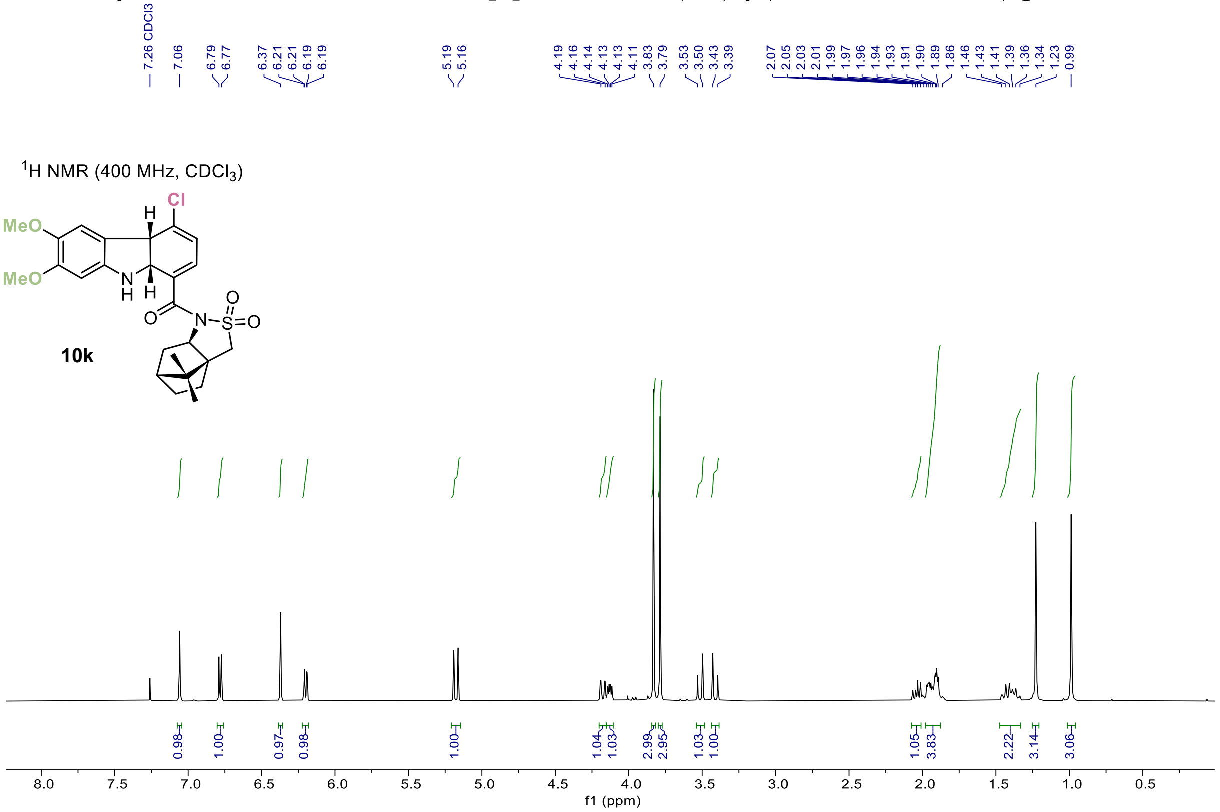

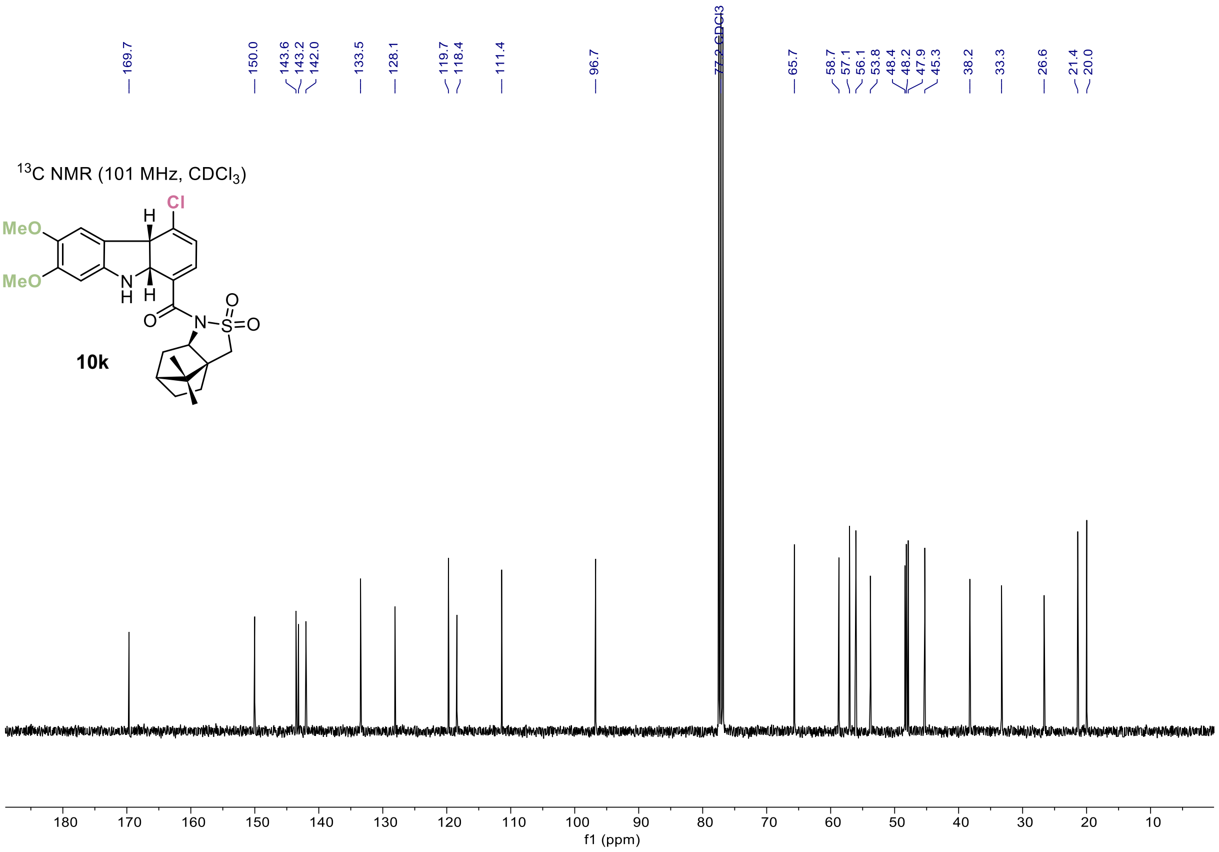

((4a*S*,9a*R*)-4-Chloro-9-methyl-4a,9a-dihydro-9*H*-carbazol-1-yl)((3a*S*,6*R*,7a*R*)-8,8-dimethyl-2,2-dioxidotetrahydro-3*H*-3a,6-methanobenzo[*c*]isothiazol-1(4*H*)-yl)methanone, **10l** (Spectra in $CDCl_3$)

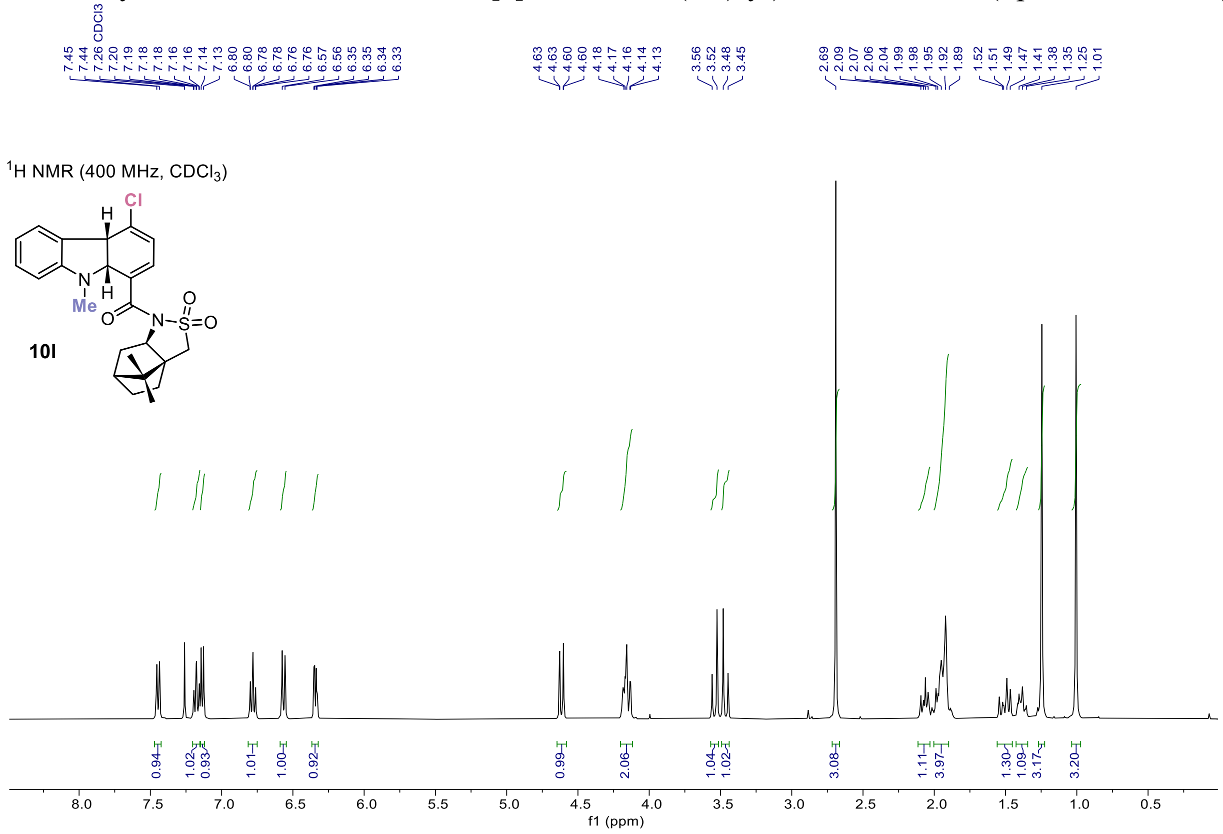

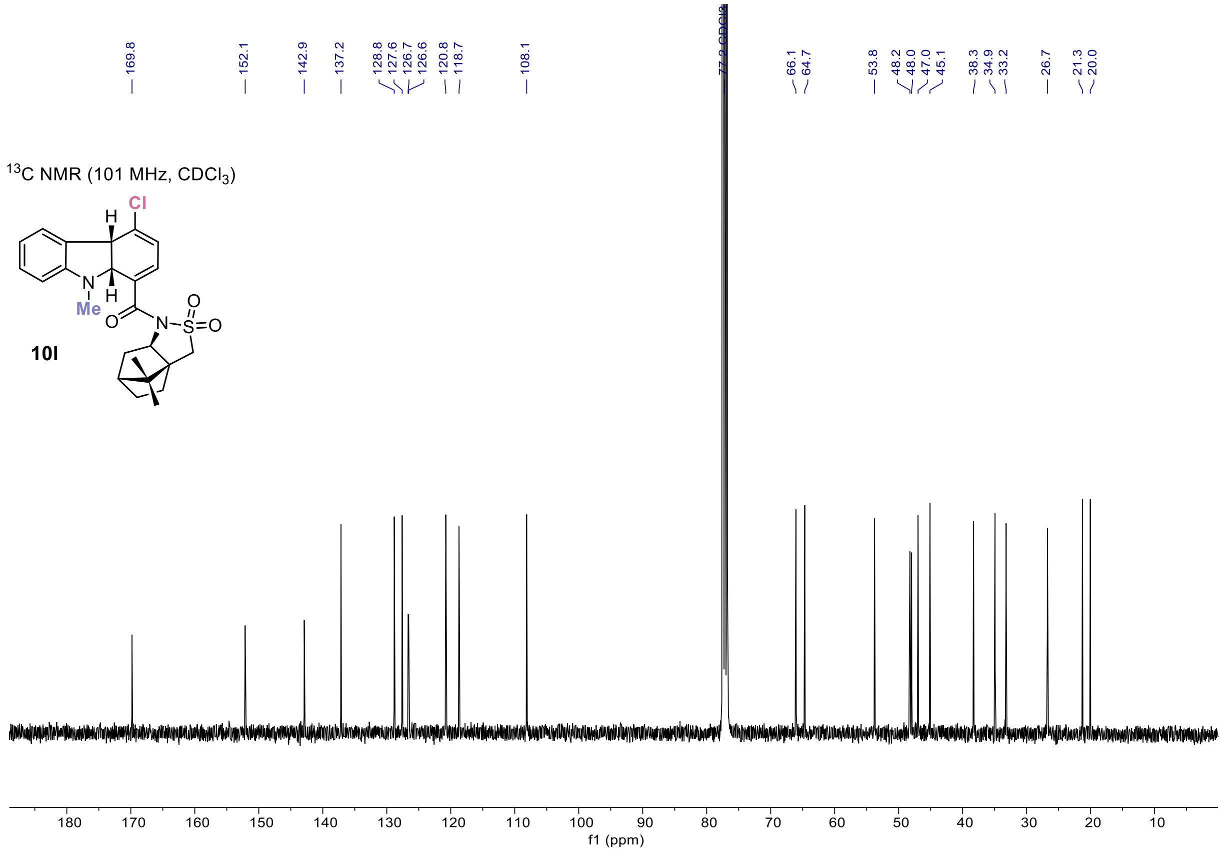

((4a*S*,9a*R*)-9-Benzyl-4-chloro-4a,9a-dihydro-9*H*-carbazol-1-yl)((3a*S*,6*R*,7a*R*)-8,8-dimethyl-2,2-dioxidotetrahydro-3*H*-3a,6-methanobenzo[*c*]isothiazol-1(4*H*)-yl)methanone, **10m** (Spectra in $CDCl_3$)

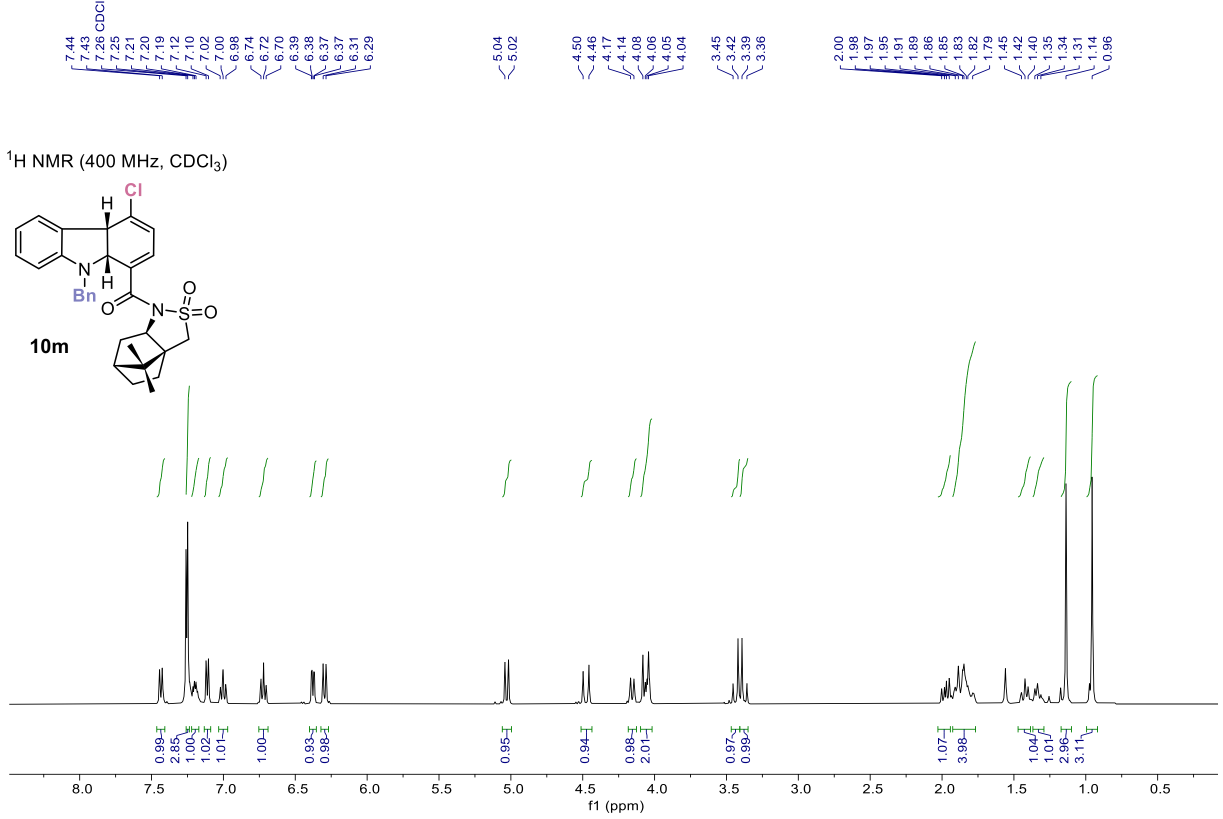

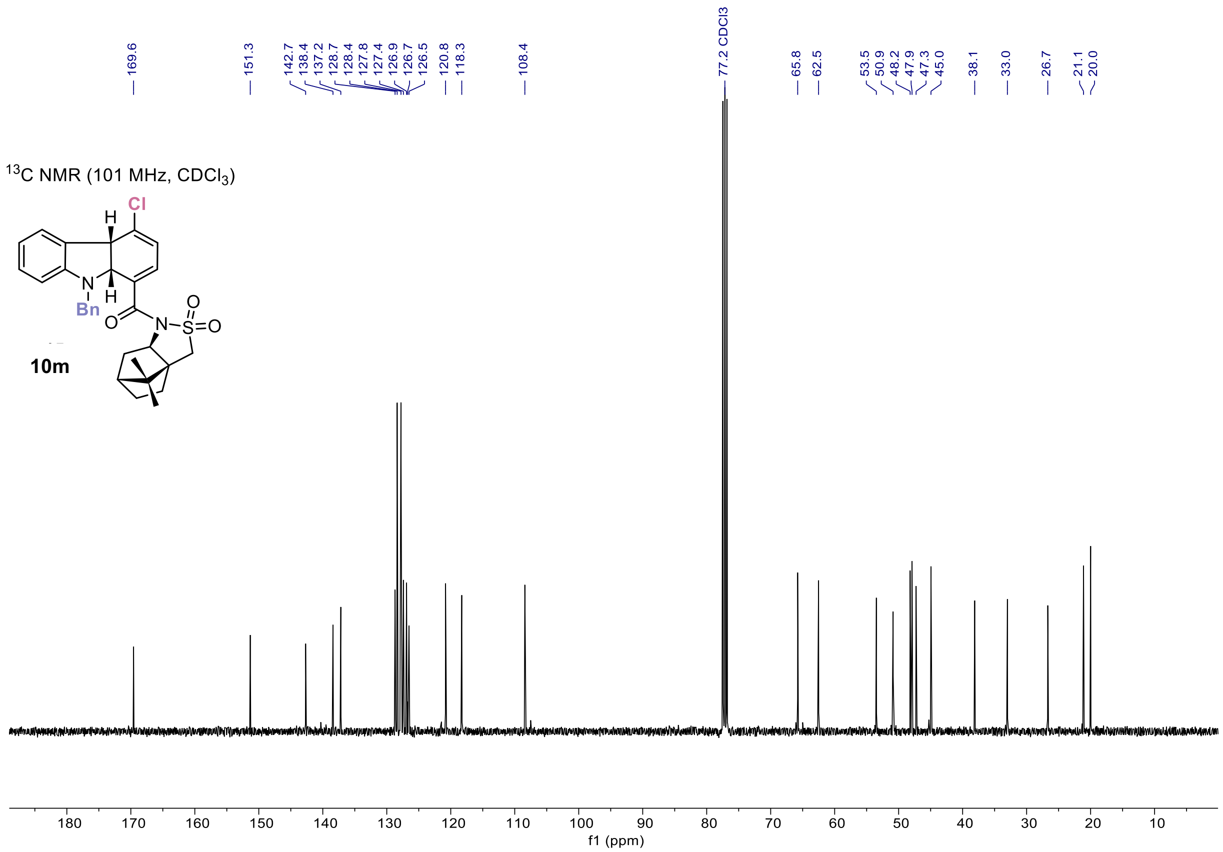

((3a*S*,6*R*,7a*R*)-8,8-Dimethyl-2,2-dioxidotetrahydro-3*H*-3a,6-methanobenzo[*c*]isothiazol-1(4*H*)-yl)(5-methylthiophen-2-yl)methanone, **S25** (Spectra in $CDCl_3$)

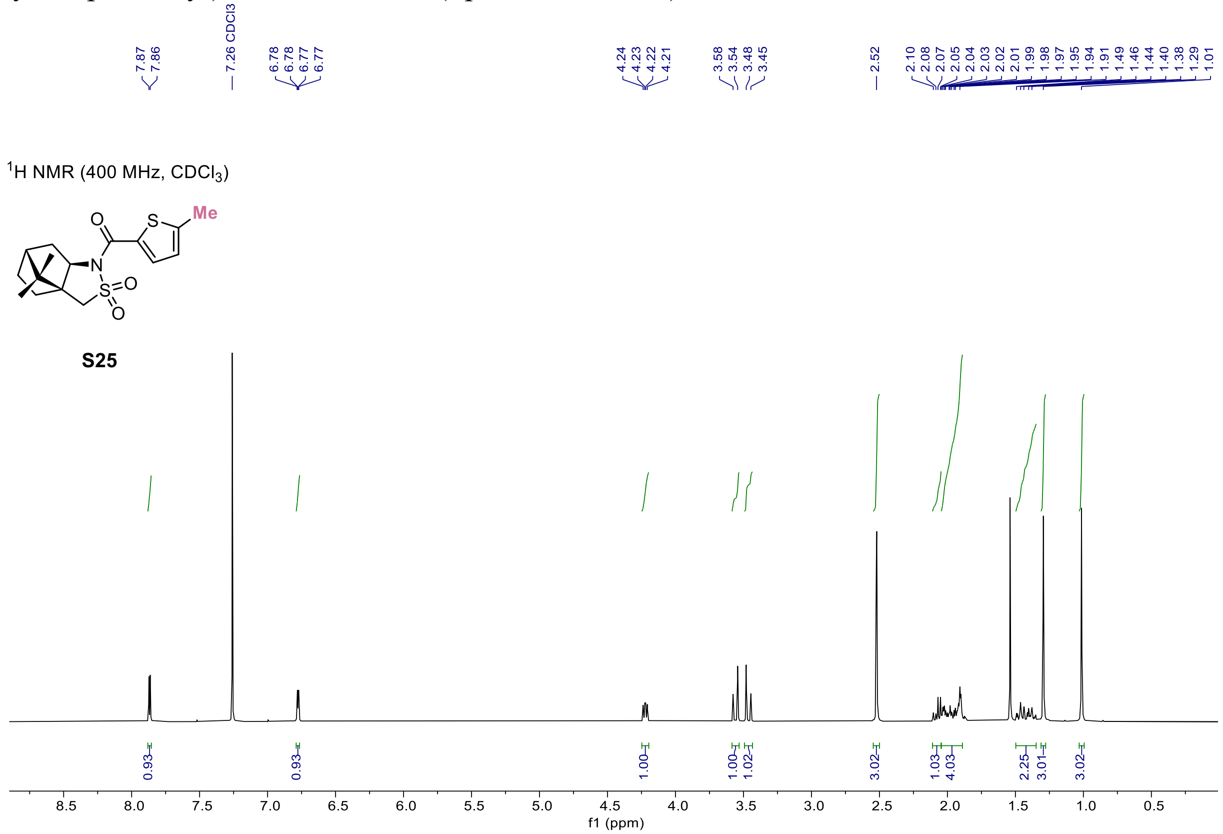

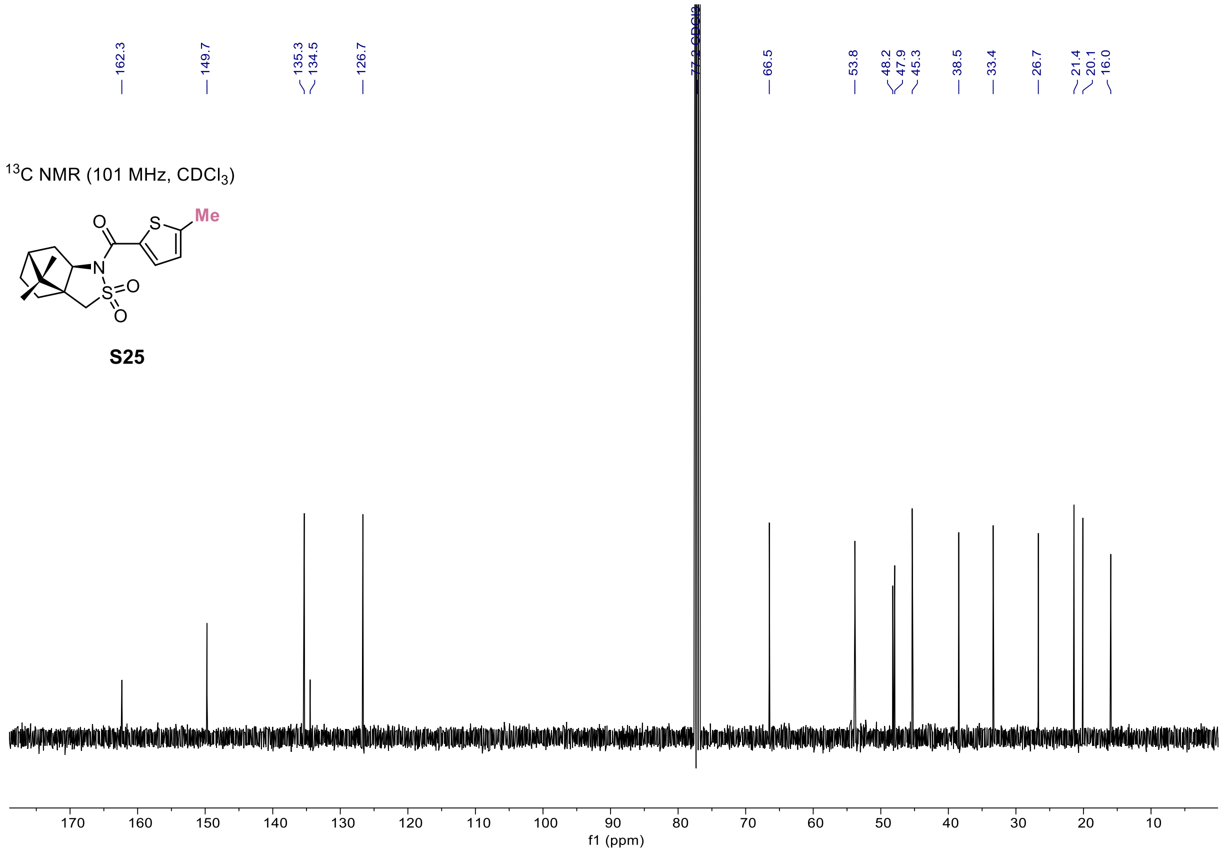

((3aS,6R,7aR)-8,8-Dimethyl-2,2-dioxidotetrahydro-3H-3a,6-methanobenzo[c]isothiazol-1(4H)-yl)(5-methyl-1,1-dioxidothiophen-2-yl)methanone, **9b** (Spectra in $CDCl_3$)

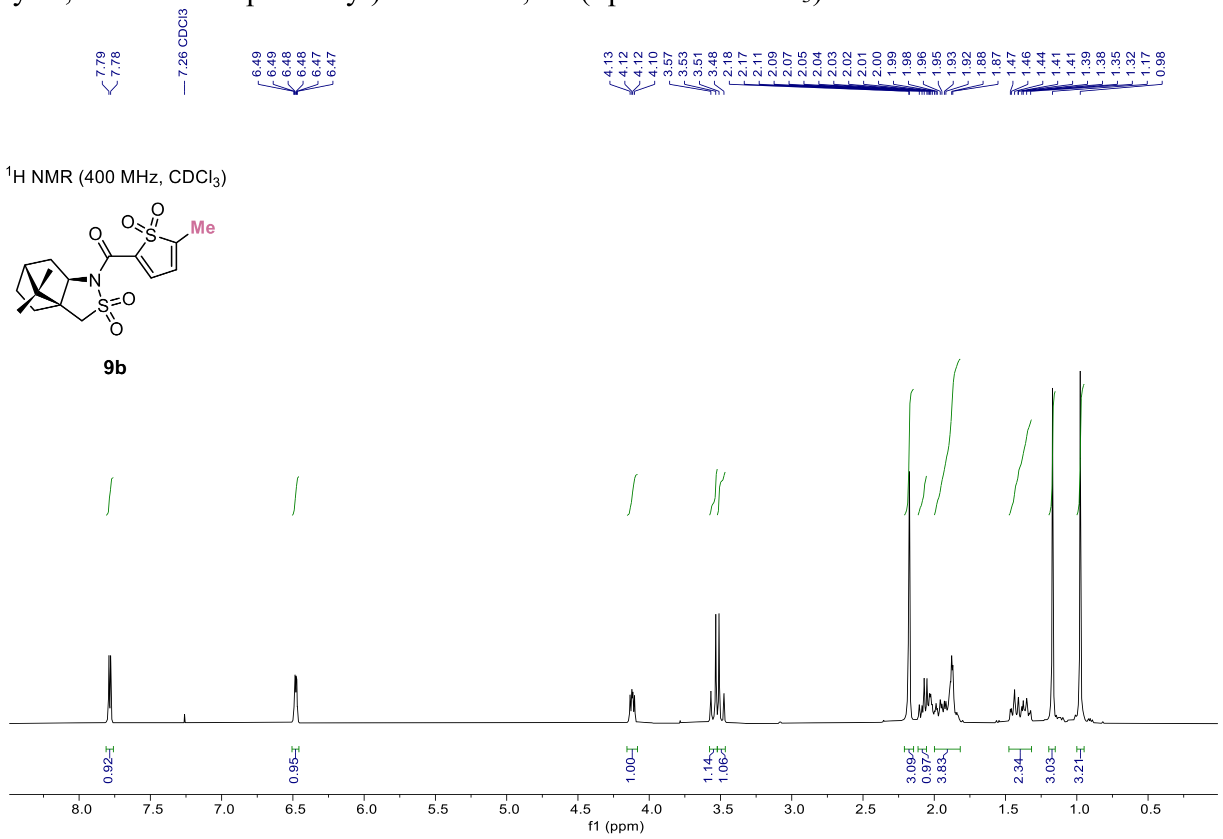

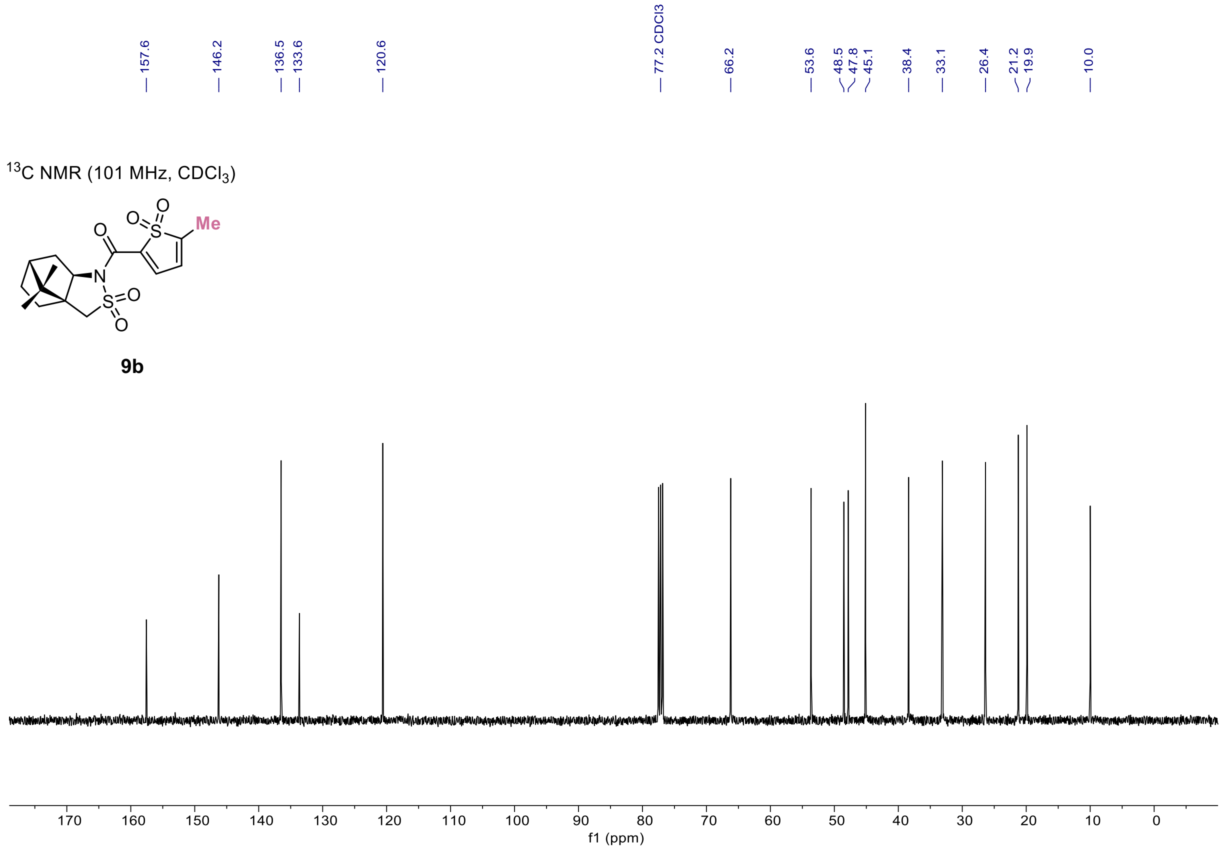

((3a*S*,6*R*,7a*R*)-8,8-Dimethyl-2,2-dioxidotetrahydro-3*H*-3a,6-methanobenzo[*c*]isothiazol-1(4*H*)-yl)((4a*S*,9a*R*)-4-methyl-4a,9a-dihydro-9*H*-carbazol-1-yl)methanone, **10n** (Spectra in $CDCl_3$)

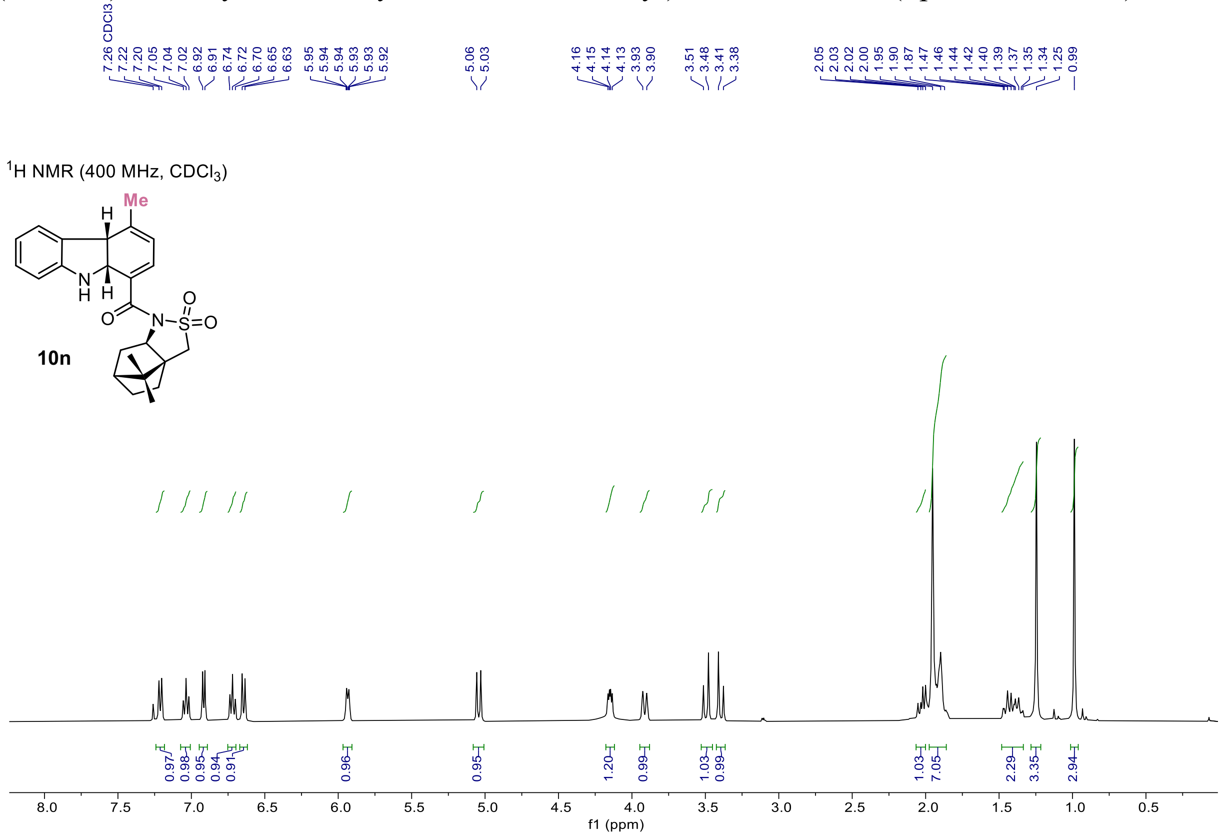

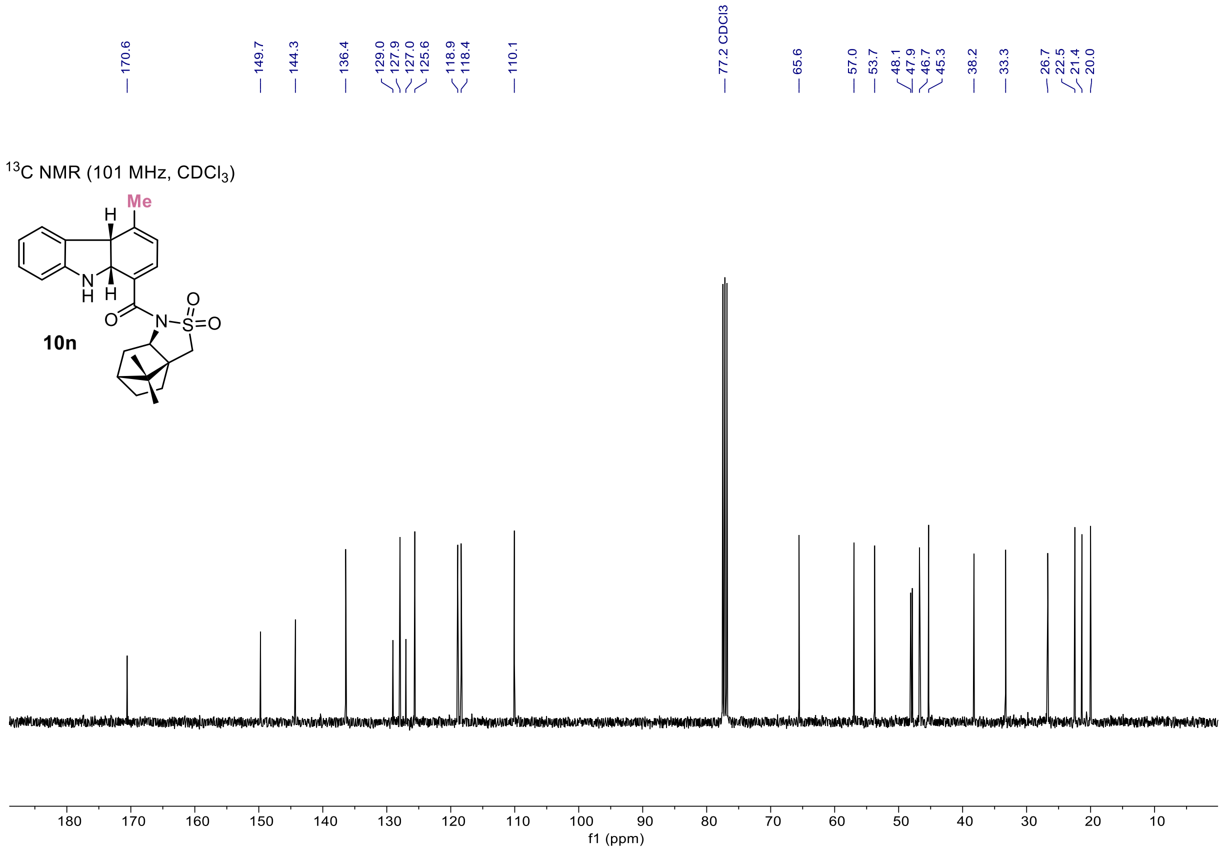

(5,6-Dihydro-4*H*-cyclopenta[*b*]thiophen-2-yl)((3a*S*,6*R*,7a*R*)-8,8-dimethyl-2,2-dioxidotetrahydro-3*H*-3a,6-methanobenzo[*c*]isothiazol-1(4*H*)-yl)methanone, **S26** (Spectra in $CDCl_3$)

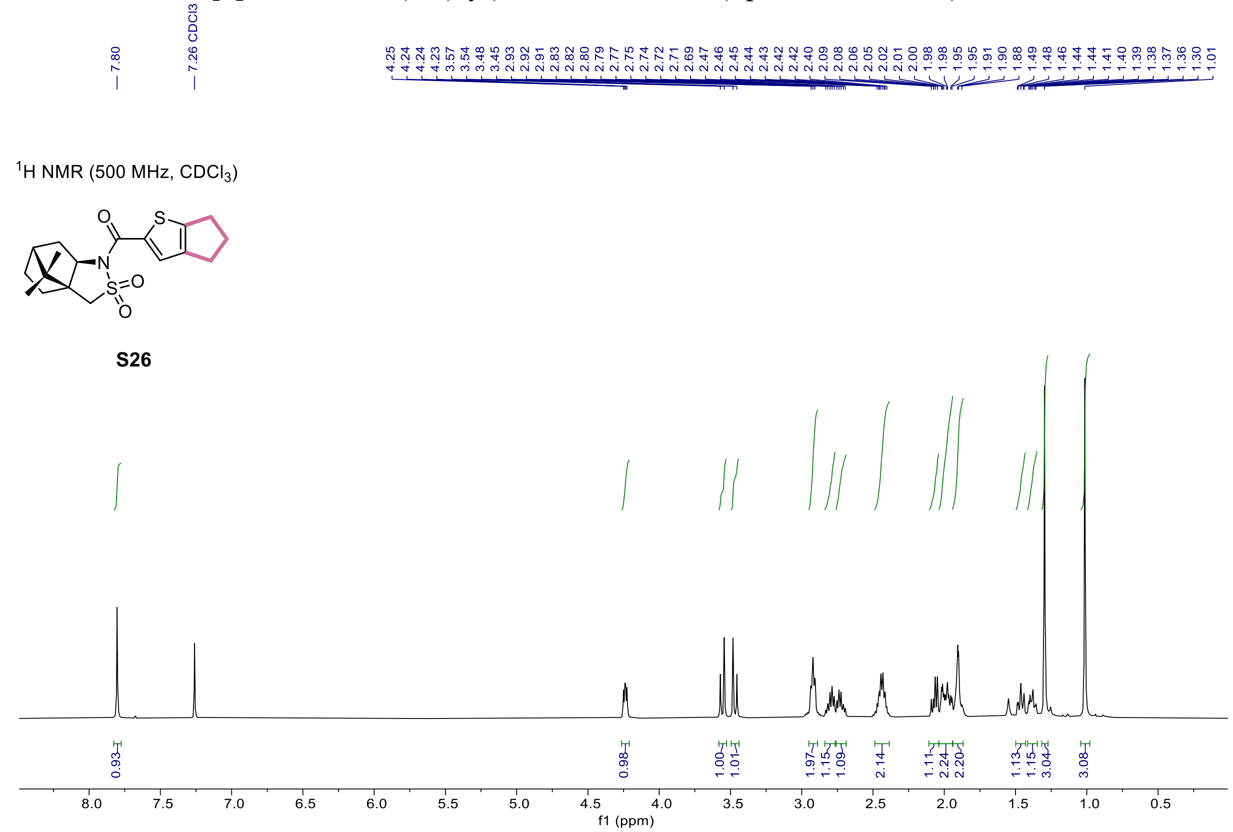

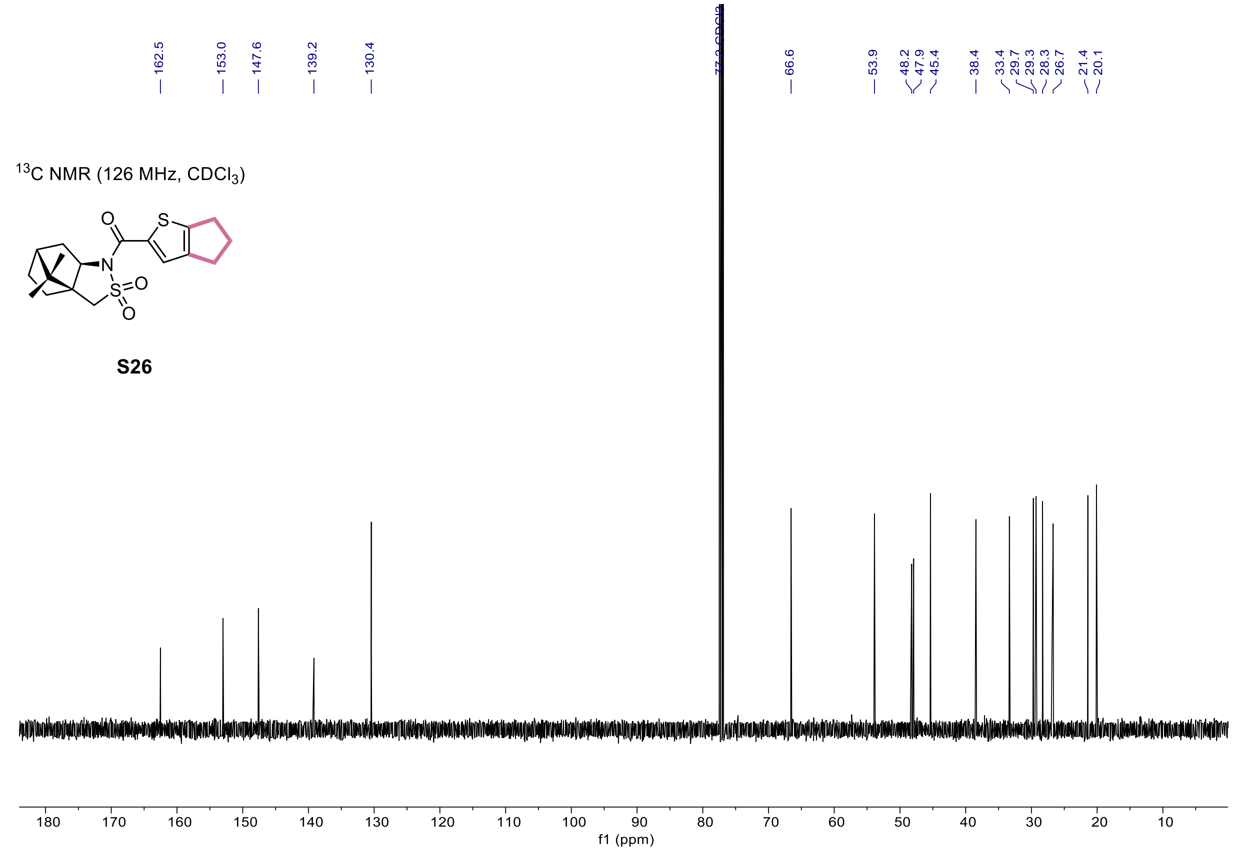

((3a*S*,6*R*,7a*R*)-8,8-Dimethyl-2,2-dioxidotetrahydro-3*H*-3a,6-methanobenzo[*c*]isothiazol-1(4*H*)-yl)(1,1-dioxido-5,6-dihydro-4*H*-cyclopenta[*b*]thiophen-2-yl)methanone, **9c** (Spectra in $CDCl_3$)

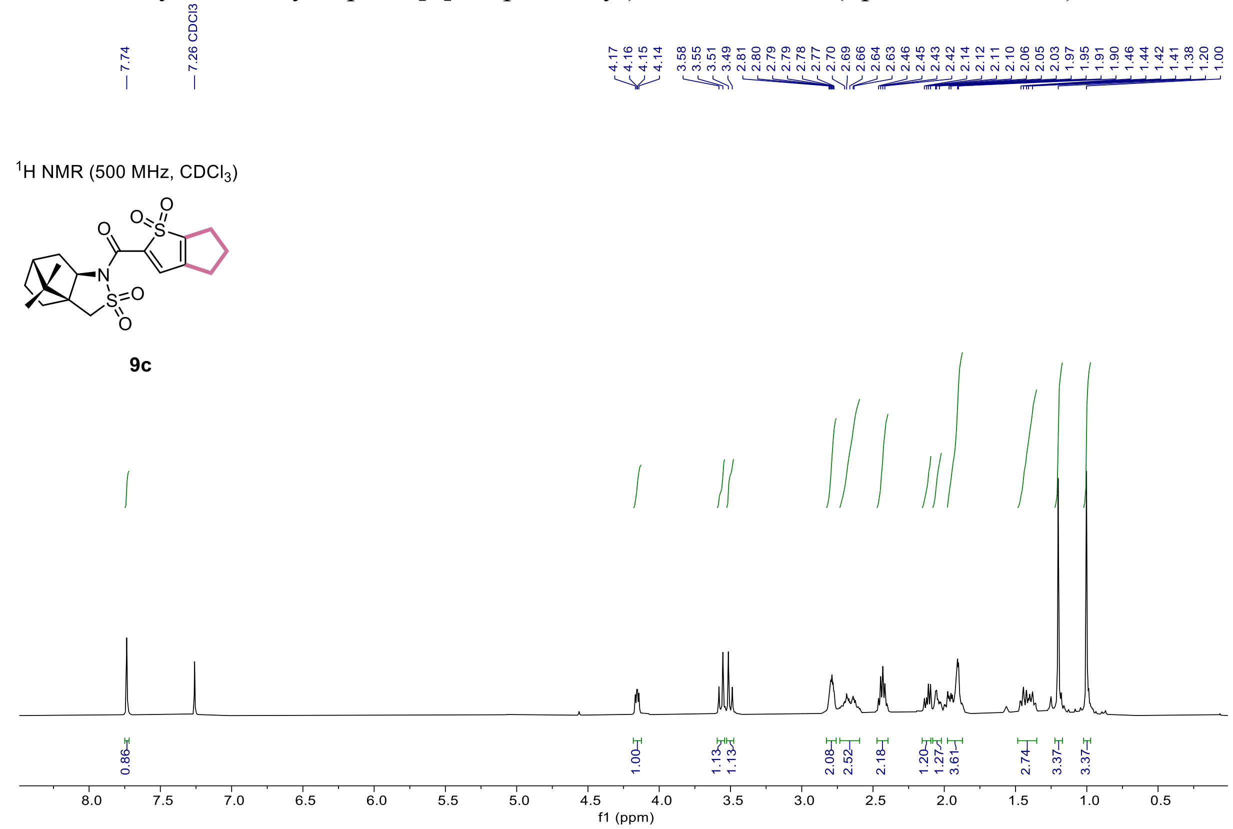

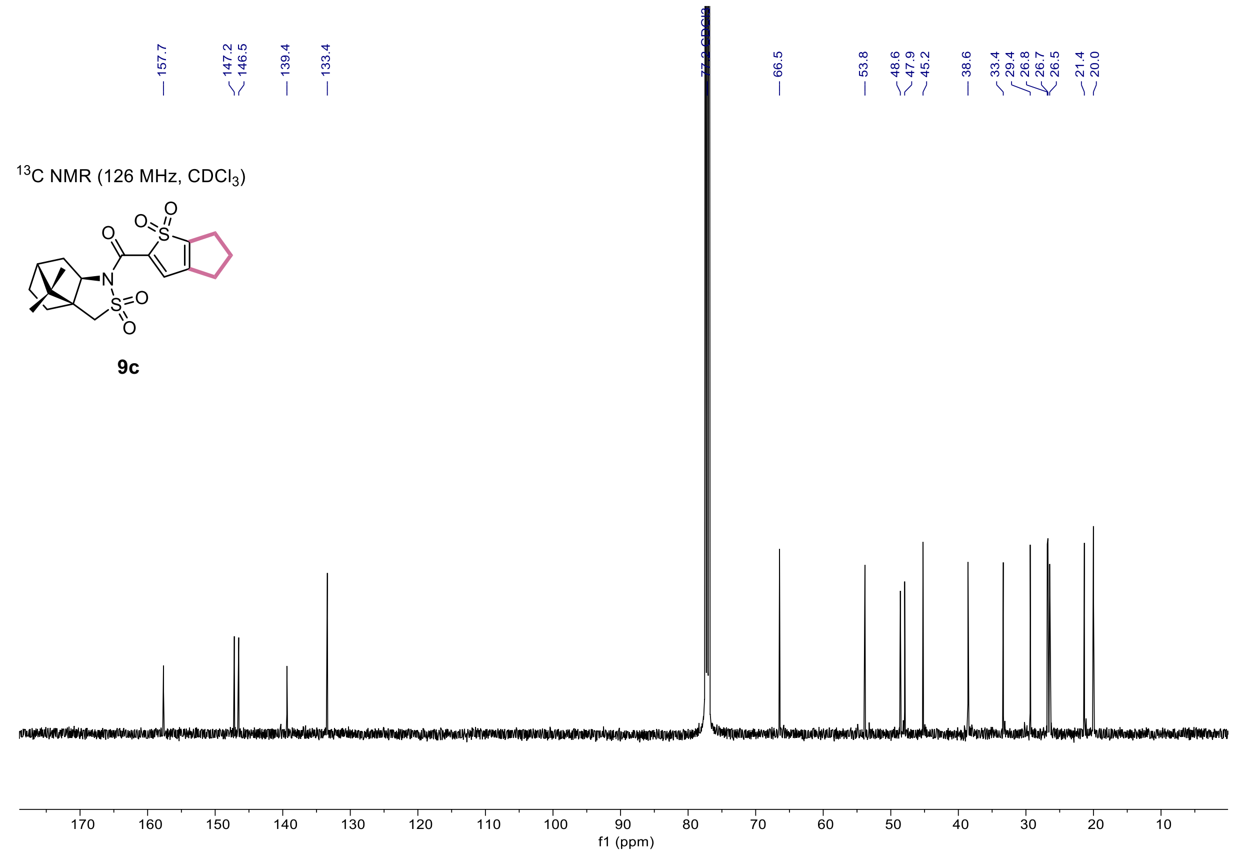

((3a*S*,6*R*,7a*R*)-8,8-Dimethyl-2,2-dioxidotetrahydro-3*H*-3a,6-methanobenzo[*c*]isothiazol-1(4*H*)-yl)((5a*R*,10b*S*)-1,2,3,5a,6,10b-hexahydrocyclopenta[*c*]carbazol-5-yl)methanone, **10o** (Spectra in $CDCl_3$)

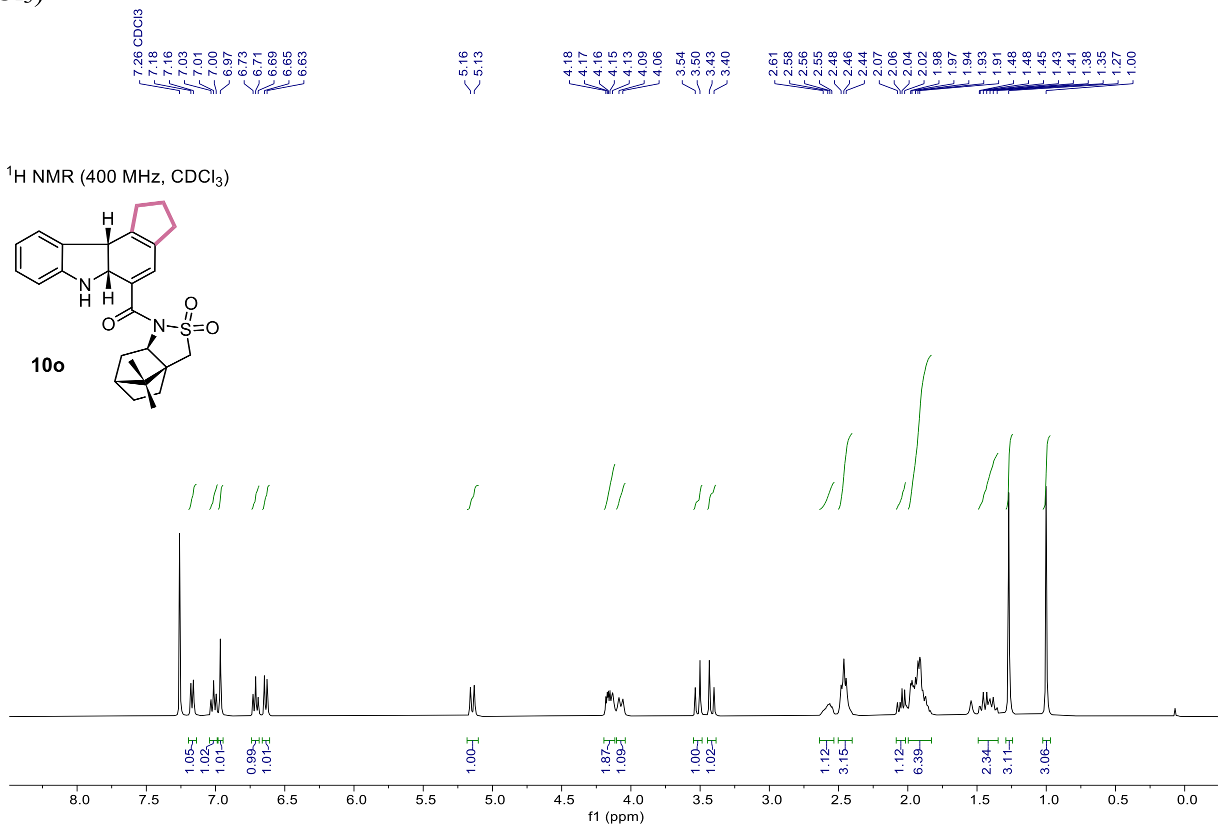

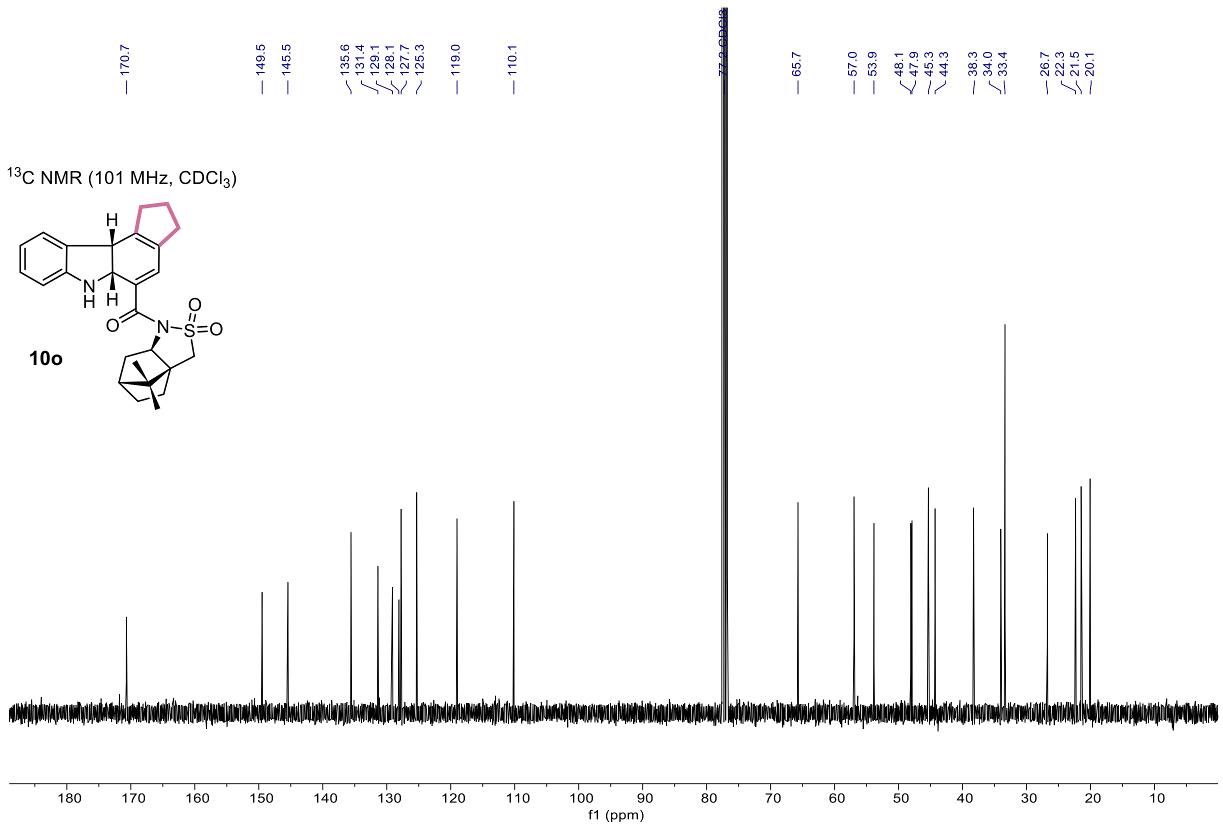

((4a*S*,9a*R*)-3-Bromo-4a,9a-dihydro-9*H*-carbazol-1-yl)((3a*S*,6*R*,7a*R*)-8,8-dimethyl-2,2-dioxidotetrahydro-3*H*-3a,6-methanobenzo[*c*]isothiazol-1(4*H*)-yl)methanone, **10p** (Spectra in $CDCl_3$)

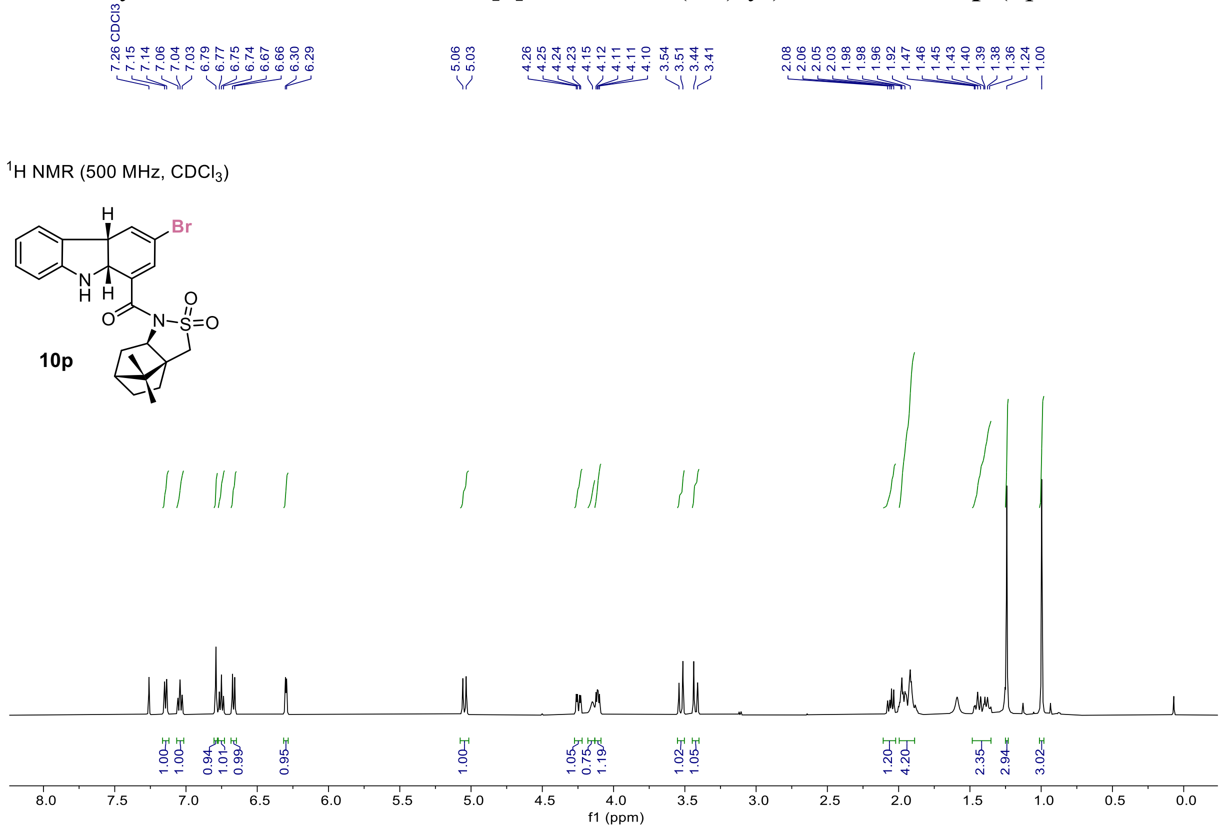

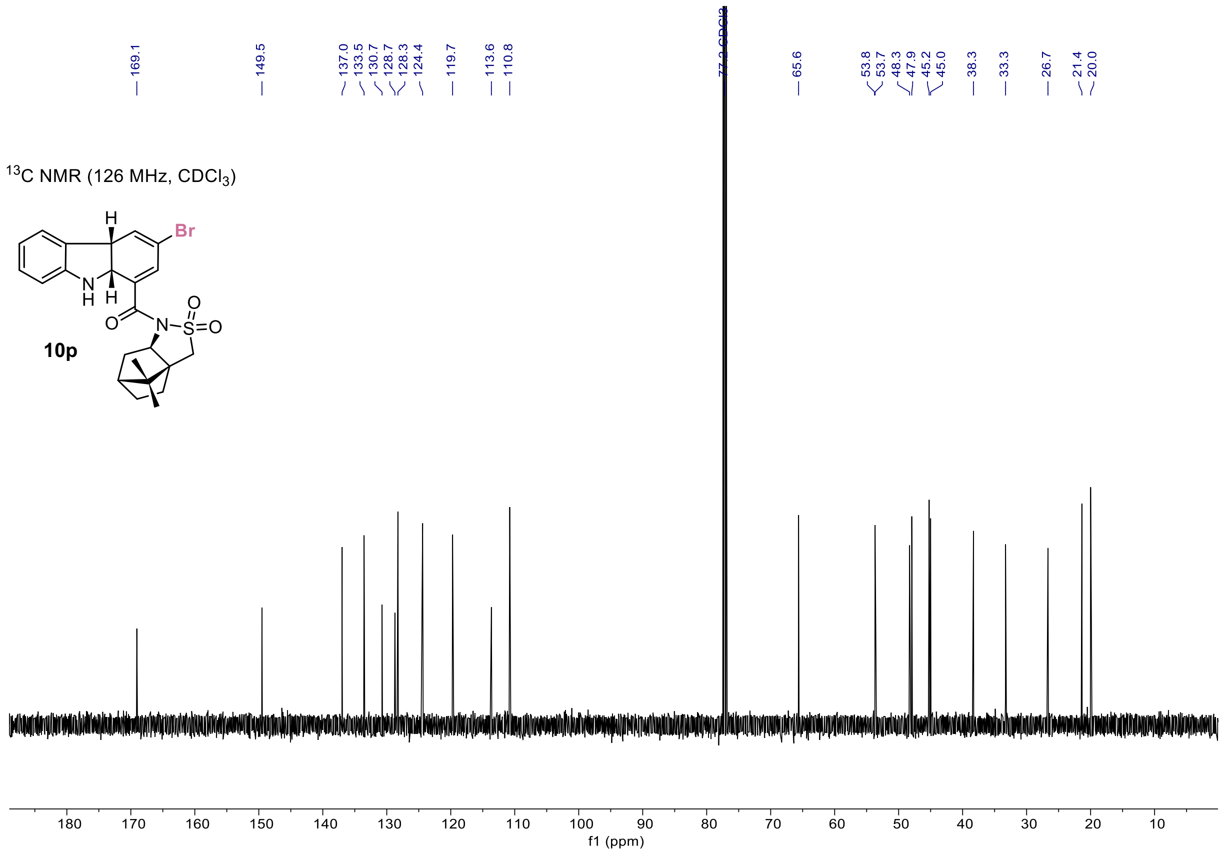

((4a*S*,9a*R*)-3-Bromo-4a,9a-dihydro-9*H*-carbazol-1-yl)((3a*S*,6*R*,7a*R*)-8,8-dimethyl-2,2-dioxidotetrahydro-3*H*-3a,6-methanobenzo[*c*]isothiazol-1(4*H*)-yl)methanone, **10q** (Spectra in $CDCl_3$)

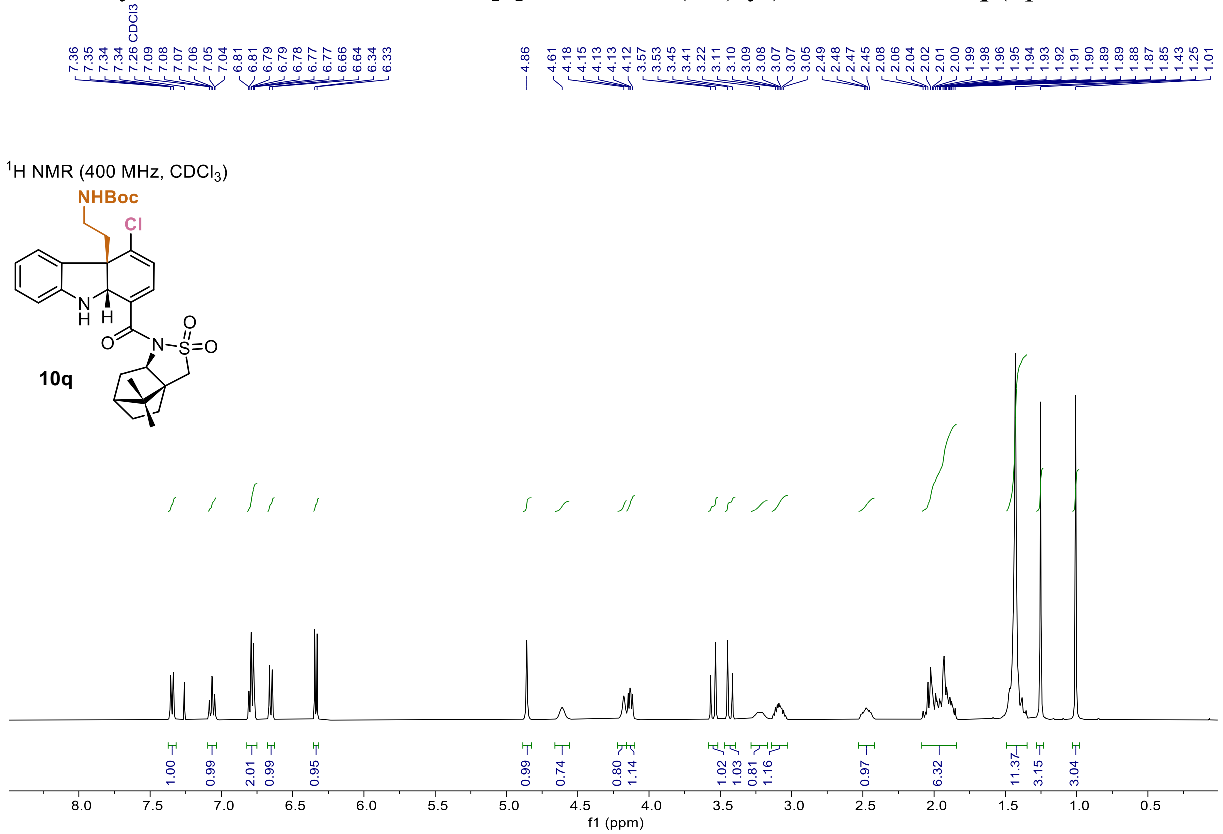

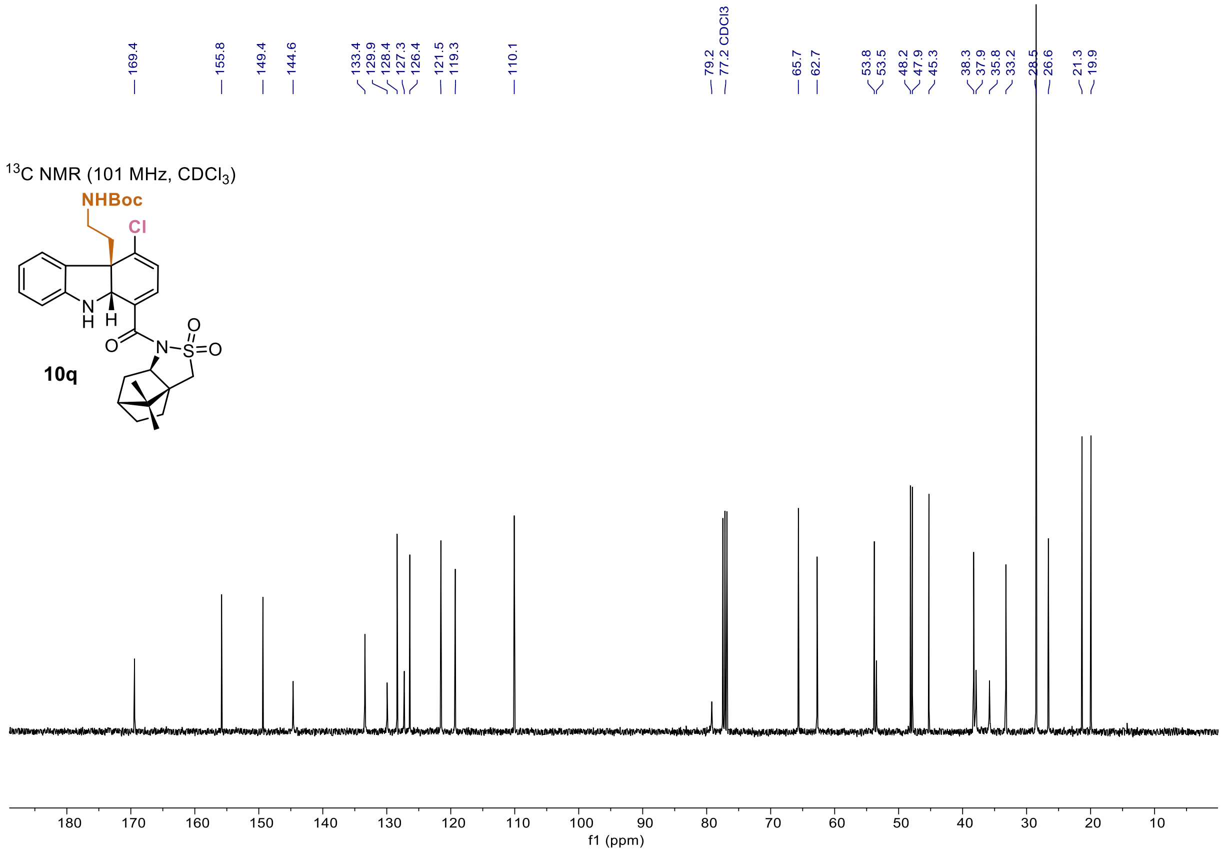

N-(2-((4a*S*,9a*R*)-1-((3a*S*,6*R*,7a*R*)-8,8-Dimethyl-2,2-dioxidohexahydro-3*H*-3a,6-methanobenzo[*c*]isothiazole-1-carbonyl)-9,9a-dihydro-4a*H*-carbazol-4a-yl)ethyl)acetamide, **10r** (Spectra in $CDCl_3$)

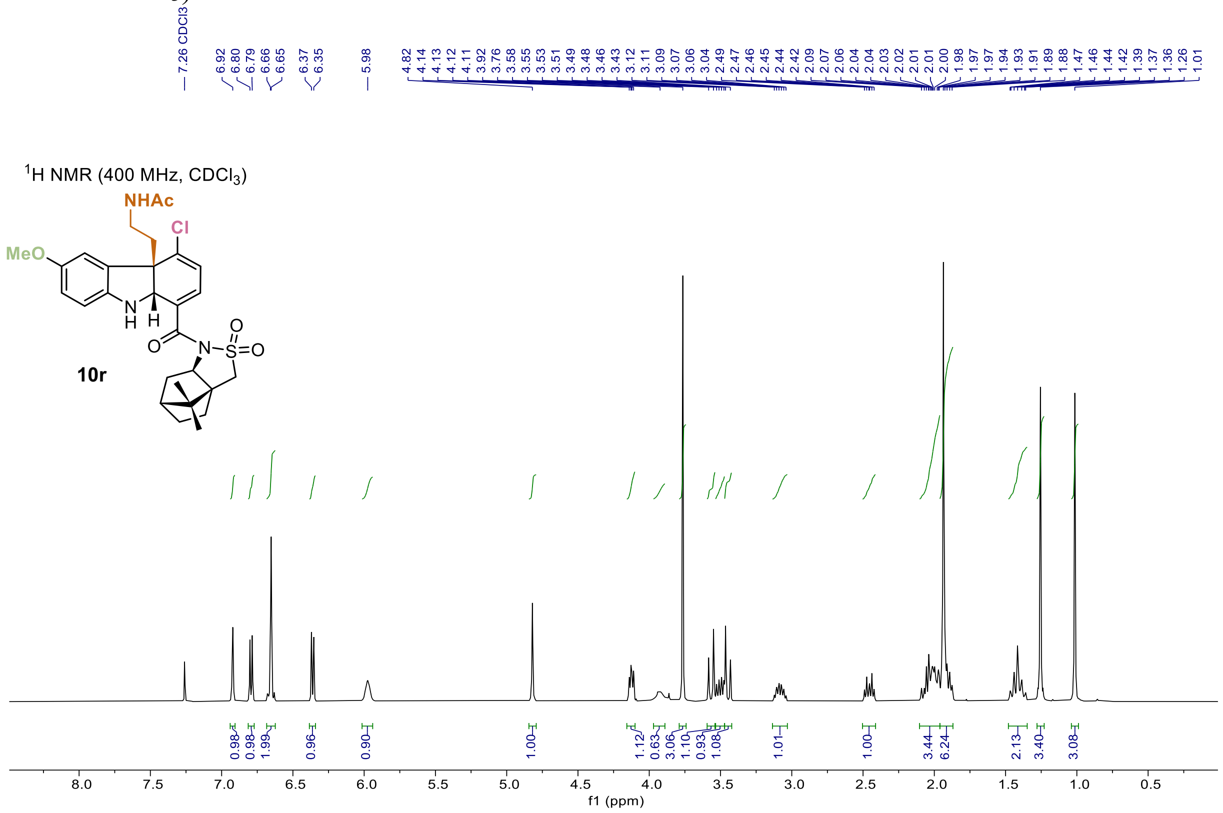

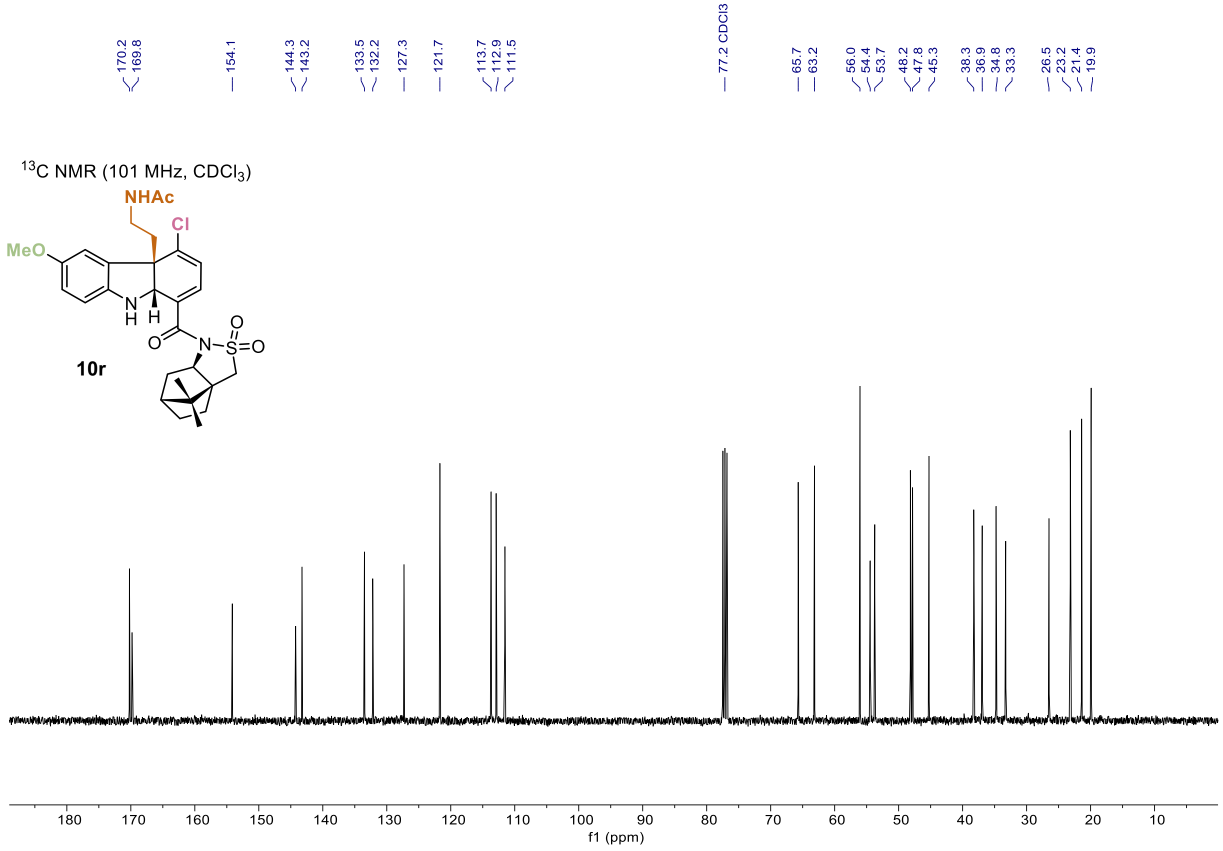

Supplement: Supplementary file 1 — Supplementary Figs. 1–11, Schemes 1–5, Tables 1–18, experimental procedures, computational details and copies of NMR spectra. [file 41557_2025_2041_MOESM1_ESM.pdf]
